# Enantioselective Intramolecular C–H Alkylation Catalyzed by a Nonsymmetric Chiral Cobalt Porphyrin

**DOI:** 10.1021/jacs.5c19047

**Published:** 2025-12-19

**Authors:** Christoph Buchelt, Stefan Breitenlechner, Julian Zuber, Stefan M. Huber, Thorsten Bach

**Affiliations:** † School of Natural Sciences, Department of Chemistry and Catalysis Research Center (CRC), 163254 Technische Universität München, Lichtenbergstr. 4, 85747 Garching, Germany; ‡ Faculty of Chemistry and Biochemistry, 9142 Ruhr University Bochum, Universitätsstr. 150, 44801 Bochum, Germany

## Abstract

Upon catalysis (1 mol %) by a chiral cobalt porphyrin, quinazolinones with a tethered diazo alkane precursor underwent an enantioselective C–H alkylation at carbon atom C4. Formation of five-, six-, and seven-membered rings was successfully accomplished (27 examples, 72–99% yield, 47–99% *ee*). Experimental and computational studies suggest that the catalyst recruits the substrates by two-point hydrogen bonding. After formation of a cobalt-entangled carbon-centered radical, a sequence of hydrogen abstraction and homolytic substitution is precisely orchestrated by the chiral confinement of the cobalt porphyrin.

## Graphical abstract

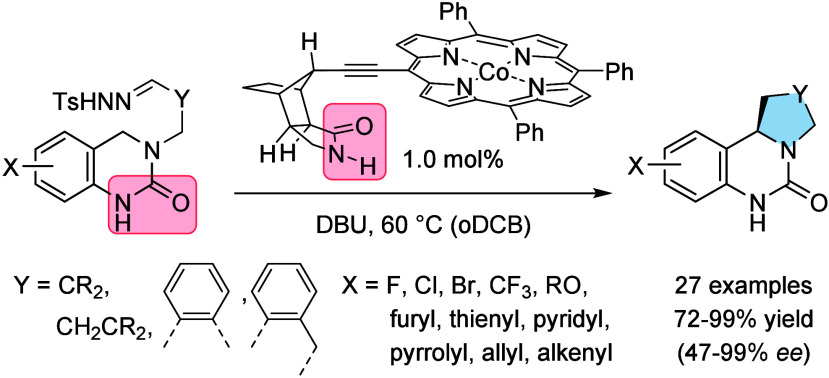

The porphyrin ligand is of critical relevance for several biological processes, including oxygen transport (hemoglobin)^1^ and monooxygenation (heme proteins).^2^ Its stability and the perfect square planar coordination of a given metal atom have led the synthetic community to investigate metal porphyrin complexes as versatile catalysts for a broad range of applications.^3^ While the planarity of the ligand core is advantageous for coordination, it poses a significant challenge if enantioselective processes are to be addressed. A possible solution relies on aryl groups that are attached to the porphyrin^4^ core and are decorated by chiral substituents. A chiral pocket is created that invites binding of a given substrate with a distinct preference for an enantiotopic face or group. Many of the devised porphyrin ligands are *C*_2_- or *D*_2_-symmetric, limiting the number of possible diastereomeric transition states.

Our group has taken an approach to enantioselective metal porphyrin-catalyzed reactions that relies on ligands which are nonsymmetric but display a noncovalent hydrogen bonding motif to recruit the substrate. Chiral Mn- and Fe-porphyrin complexes of this type had been previously used to achieve enantioselective oxygenation^5^ and amination^6^ reactions. In the context of enantioselective C–C bond formation, we identified Co-porphyrin complexes as possible catalysts. Their outstanding activity for the alkylation of hydrocarbons^7^ has been established by the groups of de Bruin^8^ and Zhang.^9^ The latter group successfully applied *D*_2_-symmetric Co-porphyrin complexes such as compound **3** to the intramolecular alkylation of *tert*-butoxycarbonyl­(Boc)-protected amines **1** (Scheme 1).^10^ 1,1-Elimination of toluenesulfinic acid (TsH) from the substrate generates a diazo compound which upon dediazotation delivers a cobalt-entangled, carbon-centered radical.^3a,3b,11,12^ Hydrogen atom transfer (HAT) is facilitated by the low bond dissociation energy of the C–H bond adjacent to the nitrogen atom. Ring closure occurs by homolytic substitution of the cobalt fragment that re-enters the catalytic cycle.

**Scheme 1:**
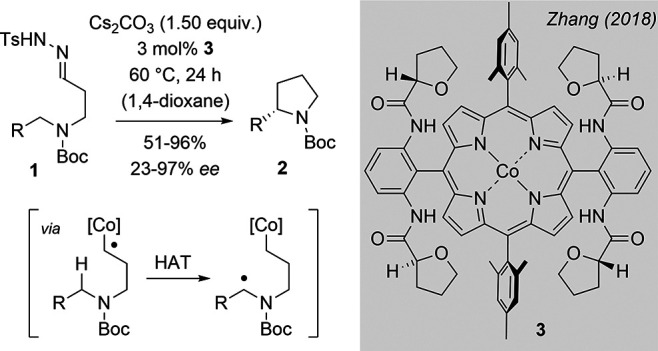
Enantioselective Cobalt-Catalyzed Intramolecular C–H Alkylation to 2-Substituted Pyrrolidines^10^

In the present study,^13^ we have now probed the application of nonsymmetric chiral Co-porphyrin complexes to an enantioselective alkylation reaction. 3-Substituted dihydroquinazolinones,^14^ e.g. **4a**, were found to react in an intramolecular alkylation upon catalysis by the achiral tetraphenylporphyrin (TPP) cobalt complex (Table 1). With DBU as the base and 1,2-dichlorobenzene (oDCB) as the solvent,^13^ the racemic product *rac*-**5a** was obtained in 85% yield (entry 1). Four chiral cobalt complexes **6** were investigated in an enantioselective approach toward the product (entries 2–5), and we found triphenyl-substituted complex **6b** to deliver the highest enantiomeric excess (*ee*). Further optimization was performed by varying the catalyst loading (entry 6), the amount of base (entries 7, 9), and the temperature (entries 8, 10). The most notable effect was observed when lowering the base loading to 2.0 equiv (entry 9). The desired product was obtained in 88% yield with 95% *ee*.

**Table 1:**
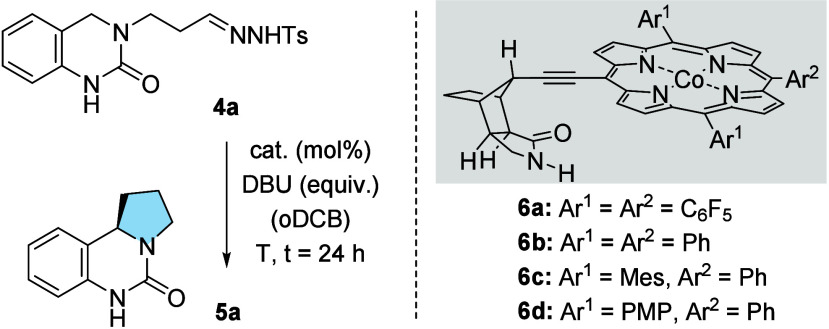
Optimization of the Enantioselective Alkylation^a^

no.	cat.	mol %^b^	equiv^c^	*T* (°C)	yield (%)^d^	*ee* (%)^e^
1	Co(TPP)	1.0	2.5	60	85	–
2	**6a**	1.0	2.5	60	82	81
3	**6b**	1.0	2.5	60	72	92
4	**6c**	1.0	2.5	60	77	69
5	**6d**	1.0	2.5	60	73	86
6	**6b**	0.5	2.5	60	99	84
7	**6b**	1.0	1.5	60	93	87
8	**6b**	1.0	1.5	50	51	92
9	**6b**	1.0	2.0	60	88	95
10	**6b**	1.0	2.0	55	36	97

a The reactions were performed on a 100 μmol scale (*c* = 20 mM).

b Amount (mol %) of catalyst.

c Equivalents of base.

d Yield of isolated product.

e Determined by chiral HPLC analysis. oDCB = 1,2-dichlorobenzene; DBU = 1,8-diazabicyclo[5.4.0]­undec-7-ene. Mes = 2,4,6-trimethylphenyl, PMP = 4-methoxyphenyl.

The conditions established in the optimization experiments were subsequently applied to several 3,4-dihydroquinazolin-2-ones **4** with an appropriately tethered side chain (Scheme 2). The absolute configuration of the resulting products was proven by taking 5-bromoquinazolinone **4b** into the enantioselective alkylation reaction. Product **5b** (72%, 91% *ee*) was crystalline and allowed its configuration to be established by single-crystal X-ray diffraction (anomalous dispersion). The configuration of all other products was assigned by analogy. Moving the bromine atom around the benzo ring of the quinazolinone led to minor deviations in yield and enantioselectivity (products **5c**, **5d**), except for a substitution in the 8-position (**5e**) for which the *ee* decreased to 47%. Other halogenated substrates carrying fluorine or chlorine substituents gave excellent results (73–99% yield, 95–99% *ee*) in the alkylation reaction (products **5f**–**5h**). Double bonds were compatible with the reaction (products **5i** and **5j**), and an 8-alkylated substrate reacted also well (product **5k**). Since methoxy substitution showed the same reaction pattern as seen for bromine (products **5l**, **5m**), it appears as if binding of the substrate to the catalyst lactam binding site is only hampered by electronegative substituents at position C-8 but not by a less polar methyl group. Heterocyclic rings were fully compatible with the reaction conditions, as seen for furan (product **5n**), thiophene (product **5o**), pyrrole (product **5p**), and pyridine (product **5q**). A potential Michael acceptor in the substrate (product **5r**) or an electron rich arene ring (product **5s**) turned out to be well tolerated. The reaction of 5-azaquinazolin-2-one delivered the desired product **5t** in high yield but with lower enantioselectivity (81% *ee*) than the carbon analogue **5a** (95% *ee*). Remarkably, substrate **4u** with a geminal dimethyl-substituted carbon atom in the linker reacted somewhat sluggishly with diminished enantioselectivity. Although performed with far fewer substrates than in the five-membered ring series, six-membered ring formation was feasible and resulted in the successful reaction to product **5aa** to **5ac** with very good results (82–99% yield, 94–98% *ee*). For seven-membered ring formation, the geometric constraints for ring closure do not seem to be perfectly met, and products **5ba** to **5bc** were obtained in a lower enantioselectivity (74–78% *ee*). A full list of substrates is given in the Supporting Information. The reaction is amenable to scale up, as was shown for the transformation **4a** → **5a**. If performed on a 1.0 mmol scale, product **5a** was isolated in 93% yield and with 95% *ee*.

**Scheme 2:**
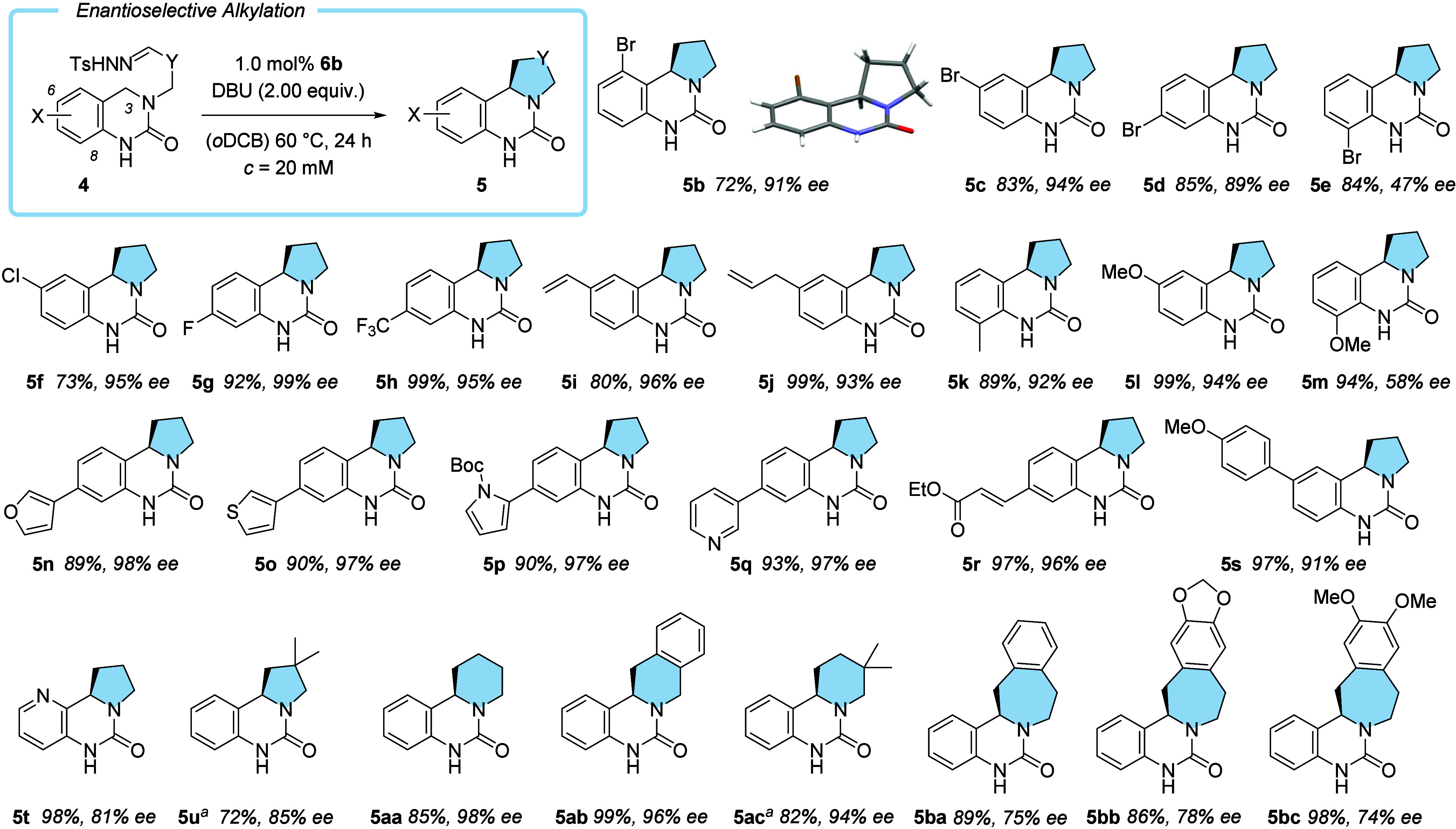
Scope of the Enantioselective Cobalt-Catalyzed Intramolecular C–H Alkylation (100 μmol Scale)

The reactions were performed on a 100 μmol scale employing chiral cobalt porphyrin **6b** as the catalyst.
a The substrate concentration was *c* = 10 mM.

A key feature of the catalyst design is the lactam binding site at the octahydro-1*H*-4,7-methanoisoindol-1-one skeleton of the catalyst. It has been proposed that a given lactam substrate binds at this position via two hydrogen bonds.^15^ While the binding behavior of the related 1,5,7-trimethyl-3-azabicyclo[3.3.1]­nonan-2-one skeleton was previously investigated,^16^ no studies had been carried out with catalysts displaying the former skeleton. Since paramagnetic compounds lead to peak broadening, ^1^H NMR titration studies were performed with diamagnetic zinc complex **7** which should display the same binding features as cobalt complex **6b**. To allow for comparison with previous experiments, we chose quinolone **8** as the binding partner. The quinolone binds to an 1,5,7-trimethyl-3-azabicyclo[3.3.1]­nonan-2-one host with an association constant *K*_a_ = 835 ± 39 L mol^–1^ (298 K, toluene-*d*_8_) which is lower than the dimerization constant of the quinolone *K*_dim_ = 2001 ± 133 L mol^–1^. Self-association (dimerization) of the enantiopure host was shown to be marginal.^16^ Likewise, homochiral zinc porphyrin complex **7** was found by dilution experiments to show no detectable self-association by hydrogen bonding. Remarkably, the association of quinolone **8** to cobalt complex **7** (Scheme 3A) is thermodynamically favored over its dimerization with *K*_a_ = 3340 ± 142 L mol^–1^ (298 K, toluene-*d*_8_). Consequently, together with the negligible self-association of the complex, the driving force for association of a given lactam substrate with the catalyst is high.

**Scheme 3:**
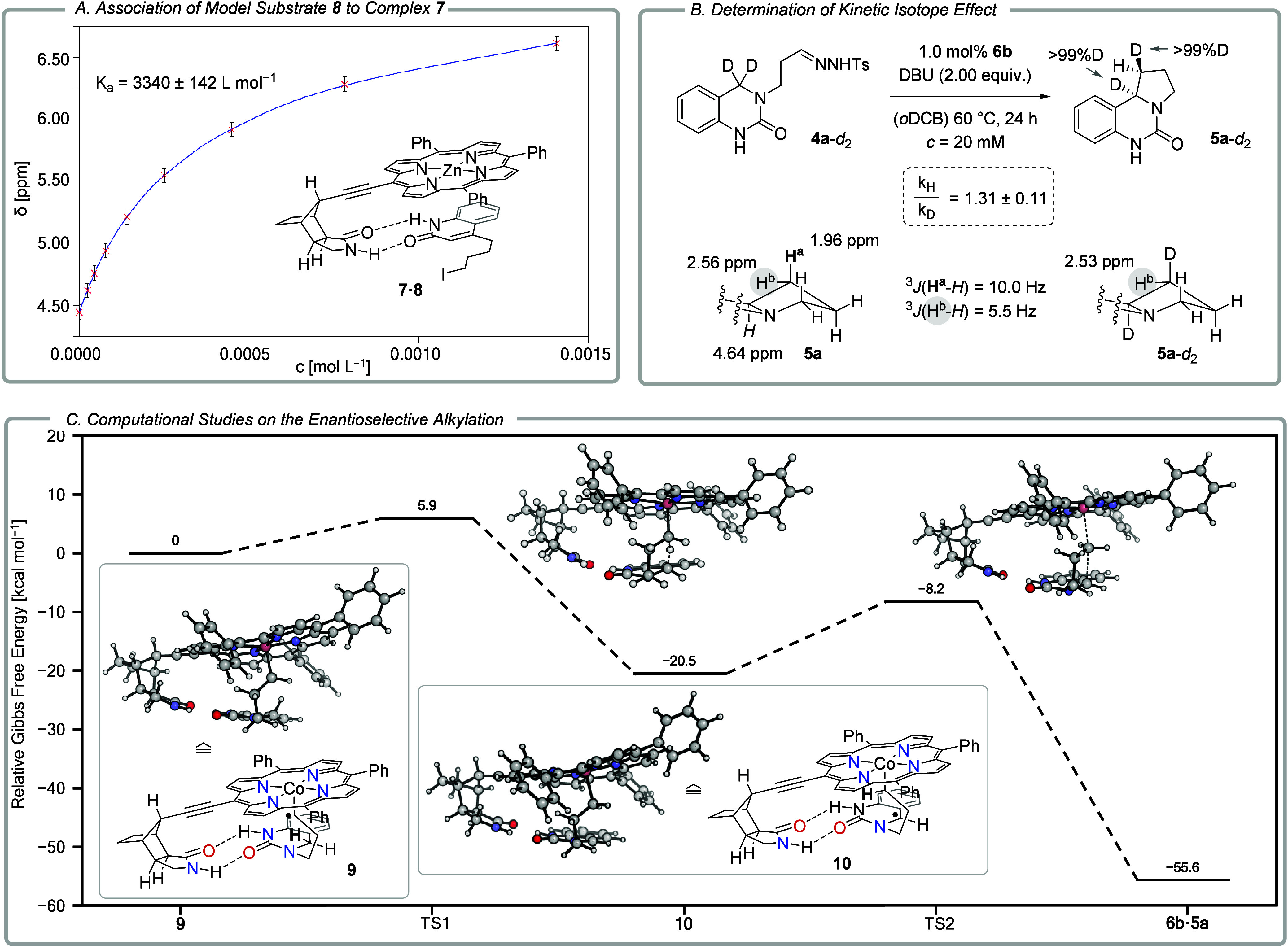
Mechanistic Experiments and Computational Studies on the Enantioselective C–H Alkylation Reaction

The kinetic isotope effect (KIE) of the catalytic reaction was determined by subjecting deuterated substrate **4a**-*d*_2_ to the conditions of the alkylation reaction. The initial rate was compared to the rate measured for the nondeuterated substrate **4a**.^17^ All measurements were done in triplicate and resulted in a small KIE k_H_/k_D_ of only 1.31 (±0.11). Deuterium incorporation into product **5a**-*d*_2_ was complete, and the relative configuration could be elucidated by NMR studies. The deuterium atom resides in the pseudoaxial position of the former hydrogen atom H^a^ (Scheme 3B). For comparison the KIE of the racemic reaction, catalyzed by Co­(TPP), was also determined. A value of k_H_/k_D_ = 1.17 (±0.03) was obtained. In contrast to the reaction catalyzed by **6b**, the racemic reaction was not diastereoselective. The racemic product *rac*-**5a**-*d*_2_ was obtained as a mixture of doubly deuterated diastereoisomers.

Based on experimental precedence,^3,11^ it is assumed that the reaction commences with 1,1-elimination of TsH by the base to form a diazoalkane. Dediazotation is mediated by the cobalt complex and leads to cobalt-entangled radical **9** (Scheme 3C). The further course of the reaction was explored by DFT calculations. To this end, the HAT and ring closure step were studied by the D3-dispersion-corrected^18^ M06L functional^19^ combined with the def2-TZVPD^20^ basis set for cobalt and the def2-TZVP^21^ basis set for all other atoms (see the Supporting Information for details). Originally, doublet and quartet multiplicities were investigated, but as expected, the former was at least 10 kcal mol^–1^ more stable for all species. In complex **9**, two strong hydrogen bonds (with O–H distances of 1.81 and 1.82 Å) are observed, and the carbon-centered radical is ideally positioned to abstract the *pro-(R)*-hydrogen atom at carbon atom C4 of the substrate (C···H distance: 2.65 Å). Abstraction occurs from the *Re* face of the radical center, which is inconsequential for the reaction of **4a** but is relevant for the reaction outcome with **4a**-*d*_2_ as the substrate. In the latter case a stereogenic center is created in α-position to the cobalt atom. The HAT process is exergonic by ΔG ≅ −20 kcal mol^–1^ and proceeds over a low barrier of about 6 kcal mol^–1^. In the transition state (**TS1**), the hydrogen atom is closer to the original carbon atom (d_C–H_ = 1.24 Å) than to the former radical center (d_C–H_ = 1.48 Å). The subsequent ring closure is decisive for the absolute and relative configuration. It features a relatively “early” transition state **TS2** (d_C–C_ = 2.42 Å), in line with the more than 35 kcal mol^–1^ gained in ΔG toward the product. The barrier is once again low, with ΔG^‡^ ≅ 12 kcal mol^–1^. The approach of the prostereogenic radical center at the quinazolinone C4 carbon atom can occur only from its *Re* face, establishing the absolute configuration of the product.

From the results obtained with deuterated substrate **4a**-*d*_2_, the homolytic substitution (S_H_2)^22^ at the metal center appears to occur with inversion of configuration. The hydrogen atom (marked in bold) ends up with high diastereoselectivity at a single position of the product (*vide supra*). The fact that achiral Co­(TPP) promotes the racemic reaction without any notable diastereoselectivity underpins the steric constraints exerted by the chiral catalyst in the hydrogen-bonded array. Regarding the catalytic cycle, a crude estimate of the KIE for **TS1** via the *Kinisot* software^23^ yielded a value of k_H_/k_D_ = 3.7 (or 4.6 including tunneling), that is far higher than the experimental value. The HAT step is thus unlikely to be turnover limiting. Since the subsequent substitution step also involves a low-lying transition state (**TS2**), it is most likely that the turnover is limited by the Co-mediated dediazotation to radical **9**. This assumption aligns with previous studies on the rate-determining step in Co-catalyzed cyclopropanation reactions.^24^ Scheme 4 summarizes the mechanistic picture that evolves from the experimental and computational studies.

**Scheme 4:**
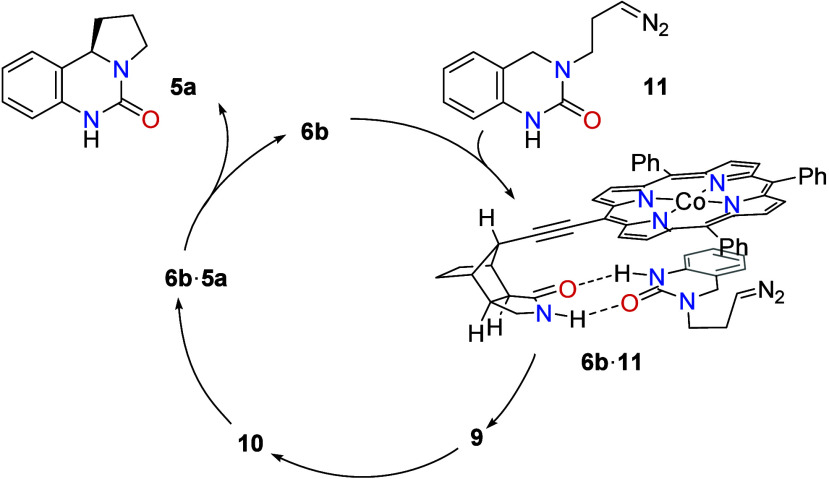
Catalytic Cycle for the Enantioselective C–H Alkylation Mediated by Co-Porphyrin Complex **6b**

Diazo compound **11** is formed from precursor **4a** by 1,1-elimination. The yellow color of the compound was instantaneously visible if the elimination was performed in the absence of the catalyst. Coordination to complex **6b** is favored for any free quinazolin-2-one as indicated by the association constant *K*_a_ for **7**·**8**. However, coordination is reversible and allows for an exchange of quinazolin-2-ones. In other words, the 1,1-elimination can occur within complex **6b**·**4a**, or complex **6b**·**11** may form from compound **6b** liberated by product release. Dediazotation expels a nitrogen molecule (N_2_) and delivers the cobalt-entangled radical that is competent to initiate a rapid intramolecular HAT. The ensuing intermediate **10** undergoes stereoselective homolytic substitution delivering product **5a** enantioselectively.

In summary, it has been shown that the chiral confinement within a hydrogen-bonded substrate–catalyst assembly is suited to induce high enantioselectivity in a Co-catalyzed C–H alkylation reaction. A wide array of N3–C4 annulated quinazolinones can be accessed by this method in high yields. Notable mechanistic features of the reaction include the low KIE observed upon deuteration at carbon atom C4 and the high facial diastereoselectivity recorded for the reaction of **4a**-*d*_2_. In addition, the association of lactam substrates to porphyrin complexes with an octahydro-1*H*-4,7-methanoisoindol-1-one backbone has been studied for the first time by NMR titration experiments.

## Supplementary Material



## Data Availability

The data that supports the findings of this study are available in the Supporting Information of this article. Primary research data are openly available in the repository RADAR4Chem with the DOI: 10.22000/yycc3s2ycgxb2sv0.

## Notes

^1^H and ^13^C NMR spectra for all new compounds; materials and methods, experimental procedures, analytical data, HPLC traces of products **5** (racemates, enantioenriched products), mechanistic experiments, and DFT calculations. (PDF)
