# Supplementary material for: Enantioselective Intramolecular C–H Alkylation Catalyzed by a Nonsymmetric Chiral Cobalt Porphyrin
Source: J Am Chem Soc. 2025 Dec 19;148(1):93–8. doi: 10.1021/jacs.5c19047 (PMC12814358; doi:10.1021/jacs.5c19047)
Supplement: Supplementary file 1 [file ja5c19047_si_001.pdf]

Supporting Information

Enantioselective Intramolecular C–H Alkylation Catalyzed by a Non-symmetric Chiral Cobalt Porphyrin

Christoph Buchelt,[a] Stefan Breitenlechner,[a] Julian Zuber,[a] Stefan M. Huber,[b] and Thorsten Bach[a]*

[a]*Technical University Munich, School of Natural Sciences, Department of Chemistry and Catalysis Research Center, Lichtenbergstrasse 4, 85747 Garching, Germany.*

[b]*Faculty of Chemistry and Biochemistry, Ruhr University Bochum, Universitätsstr. 150, 44801 Bochum, Germany*

*** Corresponding Author**

Email: thorsten.bach@ch.tum.de

Table of Contents

1. General Remarks 3

2. Preparation of Catalysts 5

3. Preparation of Hydrazones 21

4. Optimization of the Reaction Conditions with Hydrazone 4a 60

5. Enantioselective Catalysis 61

6. Deuteration Experiments 80

7. Crystallographic Data 88

8. Computational Studies 96

9. NMR Titration Experiments 141

10. NMR Spectra of New Compounds 144

11. HPLC Traces 252

12. References 282

1. General Remarks

All experiments were performed in flame-dried glassware under argon atmosphere and under anhydrous conditions using *Schlenk* techniques unless otherwise stated.

Solvents and reagents: Dry dichloromethane (CH_2Cl_2) and tetrahydrofuran (THF) were obtained from an MBraun MB-SPS 800 solvent purification system. Dry dimethylformamide (DMF), dimethylsulfoxide (DMSO), ethanol (EtOH), *o*-dichlorobenzene (*o*DCB), methanol (MeOH), and toluene (PhMe) were obtained from either Sigma-Aldrich or Acros in the highest available purity (>99%, extra dry over molecular sieves) and used without further purification. Technical solvents used for aqueous workup and purification by column chromatography [acetone (ac), dichloromethane (CH_2Cl_2), diethyl ether (Et_2O), ethyl acetate (EtOAc), hexane, methanol (MeOH), pentane] were distilled prior to use. Commercially available chemicals were obtained from BLDPharm, Sigma Aldrich and TCI Europe and used as received unless otherwise stated.

Catalyst synthesis: The employed Cobalt(II) porphyrin catalysts were synthesized according to literature procedures or purchased from Sigma Aldrich. All analytical data were in agreement with the reported data.

Chromatography: Thin layer chromatography (TLC) was performed on pre-coated glass-backed Merck Kieselgel 60 F254 plates with visualization effected with ultra-violet irradiation (λ = 254, 366 nm) and/or staining using potassium permanganate (KMnO4) solution prepared from potassium permanganate (3.00 g), potassium carbonate (20.0 g) and 5% aqueous sodium hydroxide solution (5.00 mL) in water (300 mL). Flash column chromatography was performed on silica 60 (Merck, 230–400 mesh) with the indicated eluent mixtures. Automated flash column chromatography was performed on a Büchi C-815 Flash chromatography instrument for purification of all products unless otherwise noted. In all cases, Biotage® pre-packed silica cartridges (Biotage® Sfär Silica D Duo 60 µm 10 g, manufacturer number FSRD-0445-0050, FSRD-0445-0025, FSRD-0445-0010, FSRD-0445-0005) were used in combination UV detection at 265, 280 and 320 nm.

High Performance Liquid Chromatography: High Performance Liquid Chromatography (HPLC) was performed using a Thermo Fisher Ultimate 3000 device equipped with one of the following chiral stationary phases [ChiralPak AD-H (250 × 4.6 mm), ChiralPak IC (250 × 4.6 mm), ChiralPak AS-H (250 × 4.6 mm), 6 ChiralPak IA (250 × 4.6 mm), Chiralcel OJ-RH (150 × 4.6 mm) or Chiralcel OD-RH (150 × 4.6 mm), Daicel Chemical Industries] with LPG 3400SD Pump, WPS3000SL Autosampler and a DAD 3000 photodiode array (detection at λ = 215 nm or λ = 210). Racemic samples *rac*-**5** were prepared according to a previous reported protocol[1] starting from hydrazones **4** as follows: **4** (100 µmol, 1.00 equiv.) and Co(TPP) (1.00 µmol, 1.0 mol%) were dissolved in *o*DCB (5.0 mL, 20 mM), and DBU (250 µmol, 2.50 equiv.) was added. The reaction mixture was stirred at 60 °C for 24 h, whereupon the mixture was subjected to flash column chromatography (CH_2Cl_2/ac = 9/1 → 1/1) to yield quinazolin-2-ones *rac*-**5**.

NMR spectroscopy: ^{1}H NMR spectra were recorded on Bruker AVHD-300, AVHD-400 or AVHD-500 spectrometers at 303 K operating at 300 MHz, 400 MHz and 500 MHz, respectively. Data is reported in the following manner: chemical shift [in parts per million (ppm) relative to residual $CHCl_3$ (δ_H = 7.26 ppm) or DMSO-*d*5 (δ_H = 2.50 ppm)], number of protons, multiplicity and coupling constant *J* (measured in Hz to the nearest 0.1 Hz). The multiplicity of a signal is indicated as: s-singlet, bs-broad singlet, d-doublet, t-triplet, q-quartet, quint-quintet, m-multiplet, or combinations of

these. Apparent multiplets which occur because of coupling constant equality between magnetically non-equivalent protons are marked as virtual (*virt.*). ^{13}C NMR spectra were recorded on Bruker AVHD-400 or AVHD-500 spectrometers at 303 K operating at 101 MHz and 126 MHz respectively with proton decoupling. The chemical shift [in parts per million (ppm)] is reported relative to residual $CHCl_3$ (δ_C = 77.16 ppm) or DMSO-d_5 (δ_C = 39.52 ppm). ^{19}F NMR spectra were recorded on a Bruker AVHD-400 or AVHD-500 spectrometers at 303 K operating at 376 MHz and 476 MHz and are given without reference. Spectra are reported based on appearance, not on theoretical multiplicities derived from structural information.

Mass Spectroscopy (ESI): High-resolution mass spectra (HRMS) were recorded on a Thermo Finnigan LTQ FT (HRMS-ESI) with each value obtained within 7 ppm of the calculated mass.

UV/Vis Spectroscopy: UV/Vis spectroscopy was performed on a Perkin Elmer Lambda 365+ UV-Vis spectrometer using a Hellma precision cell (quartz SUPRASIL®) with a pathlength of 1 mm.

Melting points (m.p.): Determined using a Kofler heating bar designed by L. Kofler (Reichert) without correction, with range quoted to the nearest whole number

Infrared spectroscopy (IR): Spectra were recorded on a Perkin Elmer Frontier Optica+SP10 spectrometer by ATR technique. The signal intensity is assigned using the following abbreviations: vs (very strong), s (strong), m (medium), w (weak).

Specific rotation: Specific rotations were determined using a Bellingham+Stanley ADP440+ polarimeter with a 0.5 cm cuvette at λ = 589 nm (Na-D-line) at 298 K. Specific rotation is reported as followed: $[\alpha]_D^T$ in 10^{-1} grad cm^2 g^{-1} (c was defined as g per 100 mL solvent).

2. Preparation of Catalysts

2.1. Di(1H-pyrrol-2-yl)methane (SI-1)

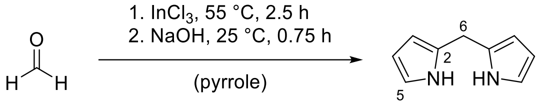

According to a modified procedure by *Frost et al.*,[2] a solution of paraformaldehyde (751 mg, 25.0 mmol, 1.00 equiv.) in freshly distilled pyrrole (173 mL, 168 g, 100 equiv.) was degassed with a stream of argon for 10 min. The solution was heated to 55 °C and $InCl_3$ (553 mg, 2.50 mmol, 0.10 equiv.) was added in one portion. The resulting suspension was stirred at 55 °C for 2.5 h. After cooling to room temperature, powdered NaOH (3.00 g, 75.0 mmol, 3.00 equiv.) was added, and the mixture was stirred for 0.75 h. The solids were filtered, washed with pyrrole (2 × 10 mL), and the filtrate was concentrated under reduced pressure. The crude product was subjected to flash column chromatography (pentane/CH_2Cl_2/EtOAc = 14/5/1 → 7/2/1) to yield dipyrromethene **SI-1** (2.41 g, 16.5 mmol, 66%) as a colorless solid.

***R*f:** 0.41 (pentane/CH_2Cl_2/EtOAc = 7/2/1) [UV, $KMnO_4$].

^{1}H NMR ($CDCl_3$, 500 MHz, 300 K): 3.99 (s, 2 H, H-6), 6.04 (m, 2 H, H-5), 6.15 (*virt.* q, $^3J \approx {}^3J'$ = 2.8 Hz, 2 H, H-4), 6.67 (*virt.* td, $^3J \approx {}^3J'$ = 2.8 Hz, ^4J = 1.6 Hz, 2 H, H-3), 7.90 (br s, 1 H, NH).

^{13}C NMR (126 MHz, $CDCl_3$): δ [ppm] = 129.2 (C-5), 117.4 (C-2), 108.5 (C-4), 106.5 (C-3), 26.5 (C-6).

Spectral data matched those reported in the literature.[2]

2.2. 5,15-Diphenylporphyrin (SI-2b)

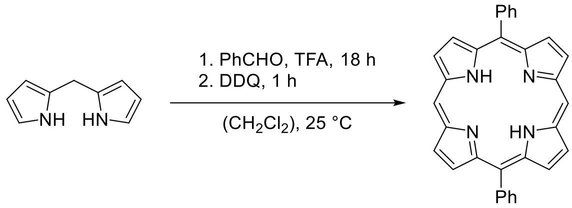

According to a modified procedure by *Schissler et al.*,[3] a solution of dipyrromethene **SI-1** (400 mg, 2.74 mmol, 1.00 equiv.) in CH_2Cl_2 (550 mL) was degassed with a stream of argon for 10 min. Benzaldehyde (0.29 mL, 298 mg, 2.80 mmol, 1.03 equiv.) and TFA (0.13 mL, 189 mg, 1.66 mmol, 0.61 equiv.) were added sequentially, and the reaction mixture was stirred for 18 h in the dark at room temperature. The mixture was oxidized using DDQ (621 mg, 2.74 mmol, 1.00 equiv.) and stirred for 1 h. The reaction mixture was neutralized with NEt_3 (0.23 mL, 168 mg, 1.66 mmol, 0.61 equiv.) and filtered over neutral aluminium oxide. The filtrate was conventrated under reduced pressure. The crude product was purified by flash column chromatography (hex/CH_2Cl_2 = 1/0 → 2/3) to yield porphyrin **SI-2b** (218 mg, 472 µmol, 35%) as a purple solid.

***R*f:** 0.41 (Hex/CH_2Cl_2 = 3/2) [vis: violett].

^{1}H NMR ($CDCl_3$, 500 MHz, 300 K): δ [ppm] = –3.11 (s, 2 H, NH), 7.79 – 7.84 (m, 6 H, H-3', H-4'), 8.23 – 8.35 (m, 4 H, H-2'), 9.09 (d, ^3J = 4.5 Hz, 4 H, H-2), 9.41 (d, ^3J = 4.5 Hz, 4 H, H-3), 10.33 (s, 2 H, H_{meso}).

^{13}C NMR ($CDCl_3$, 126 MHz, 300 K): δ [ppm] = 147.3 (C-4), 145.3 (C-1), 141.5 (C-1'), 135.0 (C-2'), 131.8 (C-3), 131.2 (C-2), 127.9 (C-4'), 127.1 (C-3'), 119.2 (C-5), 105.4 (d, C_{meso}).

UV-Vis (CH_2Cl_2): λ (nm) = 301 (ε = 28100 $cm^{-1}M^{-1}$), 406 (ε = 834900 $cm^{-1}M^{-1}$), 502 (ε = 37300 $cm^{-1}M^{-1}$), 535 (ε = 11400 $cm^{-1}M^{-1}$), 575 (ε = 12100 $cm^{-1}M^{-1}$), 630 (ε = 3900 $cm^{-1}M^{-1}$).

Spectral data matched those reported in the literature.[3]

2.3. 5,15-Bis(4-methoxyphenyl)porphyrin (SI-2d)

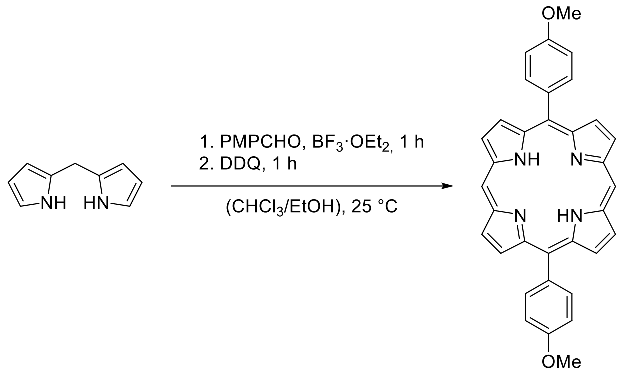

According to a modified procedure by *Banfi et al.*,[4] a solution of dipyrromethene **SI-1** (837 mg, 5.73 mmol, 1.00 equiv.) and 4-methoxyphenylbenzaldehyde (0.73 mL, 818 mg, 6.01 mmol, 1.05 equiv) in $CHCl_3$ (573 mL) and EtOH (11.5 mL) was degassed with a stream of argon for 10 min. The flask was wrapped in tinfoil and $BF_3{\cdot}OEt_2$ (0.40 mL, 447 mg, 3.15 mmol, 0.55 equiv.) was added dropwise. The reaction mixture was stirred for 1 h in the dark at room temperature, and DDQ (1.95 mg, 8.59 mmol, 1.50 equiv.) was added in one portion. After 1 h, the reaction mixture was neutralized with NEt_3 (0.44 mL, 319 mg, 3.15 mmol, 0.55 equiv.) and filtered over neutral aluminium oxide. The filtrate was conventrated under reduced pressure. Due to the pure solubility of the porphyrin, the crude product was directly subjected to the next step.

2.4. 5,10,15-Triphenylporphyrin (SI-3b)

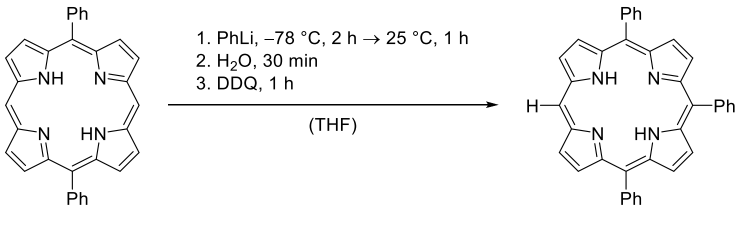

According to a modified procedure by *Schissler et al.*,[3] PhLi (1.9 M in THF, 4.80 mL, 9.13 mmol, 15.0 equiv.) was added to a solution of **SI-2b** (281 mg, 608 µmol, 1.00 equiv.) in THF (135 mL) at 0 °C dropwise, and the reaction mixture was stirred at 0 °C for 2 h. The reaction was quenched by addition of H_2O (0.79 mL, 789 mg, 43.8 mmol, 72.0 equiv.), and the resulting green solution was stirred at room temperature for 0.5 h. DDQ (552 mg, 2.43 mmol, 4.00 equiv.) was added in one portion, and the mixture was stirred for 1 h. The solids were filtered off and washed with CH_2Cl_2 (3 × 50 mL) and

the filtrate was concentrated under reduced pressure. The crude product was purified by flash column chromatography (pentane/CH_2Cl_2 = 1/0 → 2/3) to yield porphyrin **SI-3b** (230 mg, 427 µmol, 70%) as a purple solid.

***R*f:** 0.46 (Hex/CH_2Cl_2 = 3/2) [vis: braun-violett].

^{1}H NMR ($CDCl_3$, 500 MHz, 300 K): δ [ppm] = –2.99 (s, 2 H, NH), 7.72 – 7.83 (m, 9 H, H_{Ar}), 8.20 – 8.24 (m, 2 H, H_{Ar}), 8.24 – 8.27 (m, 4 H, H_{Ar}), 8.87 (d, ^3J = 4.7 Hz, 2 H, H-2†), 8.91 (d, ^3J = 4.7 Hz, 2 H, H-3†), 9.03 (d, ^3J = 4.6 Hz, 2 H, H-7*), 9.35 (d, ^3J = 4.6 Hz, 2 H, H-8*), 10.23 (s, 1 H, H-20),

† assignment is interconvertible

^{13}C NMR ($CDCl_3$, 126 MHz, 300 K): δ [ppm] = 142.7 (2C), 141.9 (2C), 134.8, 134.6, 131.6 (3C), 130.9, 127.0 (3C), 126.7, 120.7 (2C), 119.8 (2C), 105.0 (C-20).

UV-Vis (CH_2Cl_2): λ (nm) = 412 (ε = 570200 $cm^{-1}M^{-1}$), 508 (ε = 26300 $cm^{-1}M^{-1}$), 542 (ε = 8600 $cm^{-1}M^{-1}$), 582 (ε = 8700 $cm^{-1}M^{-1}$), 637 (ε = 4400 $cm^{-1}M^{-1}$).

Spectral data matched those reported in the literature.[3]

2.5. 5,15-Bis(4-methoxyphenyl)-10-phenylporphyrin (SI-3d)

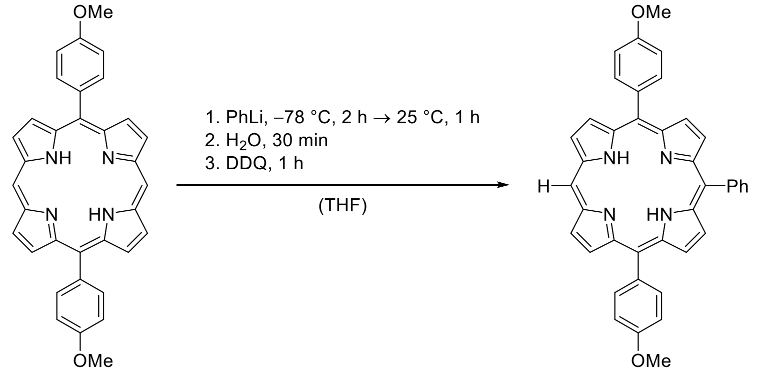

According to a modified procedure by *Plunkett et al.*,[5] PhLi (1.9 M in THF, 1.46 mL, 2.77 mmol, 15.0 equiv.) was added to a solution of **SI-2d** (96.6 mg, 184 µmol, 1.00 equiv.) in THF (41 mL) at 0 °C dropwise, and the reaction mixture was stirred at 0 °C for 2 h. The reaction was quenched by addition of H_2O (0.24 mL, 240 mg, 13.3 mmol, 72.0 equiv.), and the resulting green solution was stirred at room temperature for 0.5 h. DDQ (168 mg, 739 µmol, 4.00 equiv.) was added in one portion, and the mixture was stirred for 1 h. The solids were filtered off and washed with CH_2Cl_2 (3 × 10 mL) and the filtrate was concentrated under reduced pressure. The crude product was purified by flash column chromatography (hex/CH_2Cl_2 = 3/2 → 0/1) to yield porphyrin **SI-3d** (230 mg, 427 µmol, 70%) as a purple solid.

***R*f:** 0.23 (Hex/CH_2Cl_2 = 3/2) [vis: braun-violett].

^{1}H NMR ($CDCl_3$, 500 MHz, 300 K): δ [ppm] = –2.97 (s, 2 H, NH), 4.12 (s, 6H, OCH_3), 7.27 – 7.38 (m, 7 H, H_{Ar}), 7.73 – 7.77 (m, 3H, H_{Ar}), 8.15 – 8.17 (m, 4 H, H_{Ar}), 8.86 (d, ^3J = 4.7 Hz, 2H, H_{Ar}), 8.93 (d, ^3J = 4.7 Hz, 2H, H_{Ar}), 9.05 (d, ^3J = 4.6 Hz, 2H, H_{Ar}), 9.34 (d, ^3J = 4.6 Hz, 2H, H_{Ar}), 10.21 (s, 1H, H-20).

^{13}C NMR ($CDCl_3$, 126 MHz, 300 K): δ [ppm] = 159.5, 142.8, 135.9, 134.6, 134.3, 128.7, 128.1, 128.1, 127.4, 126.7, 126.7, 120.6, 119.5, 114.3, 112.5, 104.9, 55.8.

IR (ATR): ṽ [cm^{-1}] = 3312 (w), 2947 (w, C–H), 2916 (w, C–H), 2854 (w, C–H), 1610 (w, C=C), 1598 (w, C=C), 1558 (w, C=C), 1441 (w), 1249 (w, C–O), 1223 (w), 1194 (w), 1148 (w, C–O), 1046 (s), 797 (vs, C–H), 782 (m, C–H), 740 (m, C–H), 720 (m, C–H), 702 (m, C–H).

UV-Vis (CH_2Cl_2): λ (nm) = 412 (ε = 435700 $cm^{-1}M^{-1}$), 507 (ε = 23300 $cm^{-1}M^{-1}$), 540 (ε = 7200 $cm^{-1}M^{-1}$), 584 (ε = 8500 $cm^{-1}M^{-1}$), 638 (ε = 4500 $cm^{-1}M^{-1}$).

Spectral data matched those reported in the literature.[5]

2.6. 5,10,15-Triphenylporphyrin zinc(II) (SI-4b)

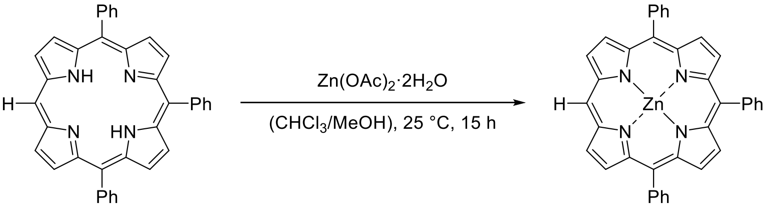

According to a modified procedure by *Frost et al.*,[2] $Zn(OAc)_2 \cdot 2H_2O$ (419 mg, 1.91 mmol, 5.00 equiv.) was added to a solution of **SI-3b** (206 mg, 382 µmol, 1.00 equiv.) in $CHCl_3$ (45 mL) and MeOH (22 mL), and the reaction mixture was stirred at room temperature for 15 h. The solvent was removed under reduced pressure, and the crude product was purified by flash column chromatography (pentane/CH_2Cl_2 = 1/0 → 1/4) to yield porphyrin **SI-4b** (218 mg, 362 µmol, 95%) as a pink solid.

***R*f:** 0.35 (pentane/CH_2Cl_2 = 3/2) [vis: pink].

^{1}H NMR ($CDCl_3$, 500 MHz, 300 K): δ [ppm] = 7.72 – 7.83 (m, 9 H, H_{Ph}), 8.20 – 8.27 (m, 6 H, H_{Ph}), 8.98 (d, ^{3}J = 4.6 Hz, 2 H, H-2†), 9.00 (d, ^{3}J = 4.6 Hz, 2 H, H-3†), 9.08 (d, ^{3}J = 4.4 Hz, 2 H, H-7‡), 9.37 (d, ^{3}J = 4.4 Hz, 2 H, H-8‡), 10.22 (s, 1 H, H-20).

†,‡ assignment is interconvertible

^{13}C NMR ($CDCl_3$, 126 MHz, 300 K): δ [ppm] = 150.3 (2C, C-4 und C-6†), 150.0 (C-9†), 149.9 (C-1), 143.0 (C_{Ph}), 142.8 (C_{Ph}), 134.7 (2C, C_{Ph}), 134.6 (C_{Ph}), 132.8 (C-7‡), 132.2 (C-2**†**), 132.0 (C-3**†**), 131.9 (C-8‡), 127.6 (C_{Ph}), 126.8 (C_{Ph}), 126.6 (C_{Ph}), 121.6 (C-5⸸), 120.7 (C-10⸸), 106.0 (C-20).

†,‡,**†**,⸸ assignment is interconvertible

UV-Vis (CH_2Cl_2): λ (nm) = 308 (ε = 15700 $cm^{-1}M^{-1}$), 350 (ε = 12100 $cm^{-1}M^{-1}$), 413 (ε = 456600 $cm^{-1}M^{-1}$), 541 (ε = 19000 $cm^{-1}M^{-1}$).

2.7. 5,15-Bis(4-methoxyphenyl)-10-phenylporphyrin zinc(II) (SI-4d)

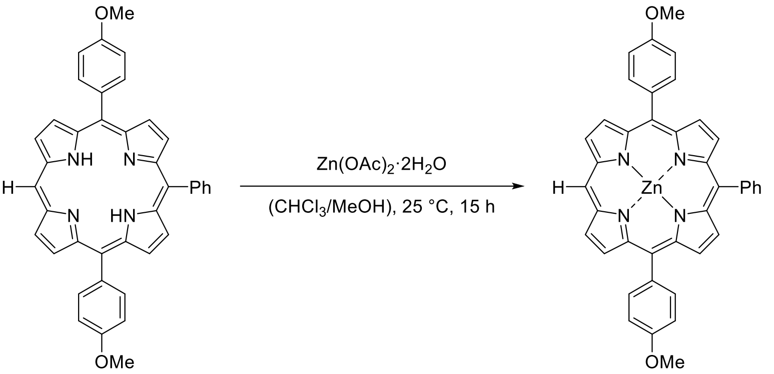

According to a modified procedure by *Frost et al.*,[2] $Zn(OAc)_2·2H_2O$ (73.3 mg, 334 µmol, 5.00 equiv.) was added to a solution of **SI-3d** (40.0 mg, 66.8 µmol, 1.00 equiv.) in $CHCl_3$ (6.7 mL) and MeOH (3.3 mL), and the reaction mixture was stirred at room temperature for 15 h. The solvent was removed under reduced pressure, and the crude product was purified by flash column chromatography (pentane/CH_2Cl_2 = 3/2 → 0/1) to yield porphyrin **SI-4d** (21.8 mg, 32.9 µmol, 49%) as a pink solid.

***R*f:** 0.40 (pentane/CH_2Cl_2 = 1/1) [vis: pink].

^{1}H NMR ($CDCl_3$, 500 MHz, 300 K): δ [ppm] = 4.11 (s, 6H, OCH_3), 7.28 – 7.34 (m, 4H, H_{Ar}), 7.73 – 7.80 (m, 3H, H_{Ar}), 8.14 – 8.16 (m, 4 H, H_{Ar}), 8.21 – 8.23 (m, 2H, H_{Ar}), 8.97 (d, ^3J = 4.6 Hz, 2H, H_{Ar}), 9.02 (d, ^3J = 4.6 Hz, 2H, H_{Ar}), 9.12 (d, ^3J = 4.4 Hz, 2H, H_{Ar}), 9.39 (d, ^3J = 4.4 Hz, 2H, H_{Ar}), 10.23 (s, 1H, H-20).

^{13}C NMR ($CDCl_3$, 126 MHz, 300 K): δ [ppm] = 159.4, 150.7, 150.6, 150.0, 149.9, 143.1, 135.6, 135.2, 134.6, 132.8, 132.1, 132.0, 131.8, 128.6, 128.1, 128.1, 127.7, 127.6, 126.6, 121.5, 120.5, 112.3, 106.0, 55.7.

IR (ATR): ṽ [cm^{-1}] = 3027 (m), 3002 (w), 2960 (m, C–H), 2930 (m, C–H), 2836 (m, C–H), 1603 (s, C=N), 1574 (m, C=C), 1524 (m, C=C), 1501 (s, C=C), 1439 (s), 1246 (vs, C–O), 1067 (s, C–O), 1048 (m), 861 (s), 849 (s), 802 (vs), 795 (vs), 784 (vs), 775 (vs, C–H), 752 (s, C–H), 741 (vs, C–H), 725 (vs, C–H).

UV-Vis (CH_2Cl_2): λ (nm) = 415 (ε = 484800 $cm^{-1}M^{-1}$), 543 (ε = 24100 $cm^{-1}M^{-1}$), 579 (ε = 7000 $cm^{-1}M^{-1}$).

2.8. 5-Bromo-10,15,20-triphenylporphyrinzinc(II) (SI-5b)

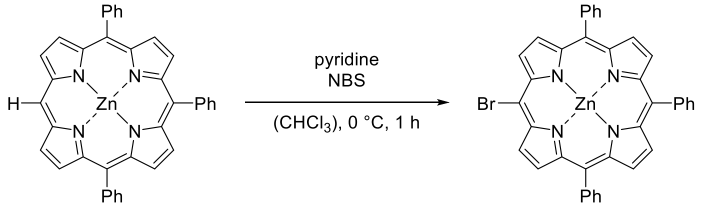

According to a modified procedure by *Frost et al.*,[2] pyridine (0.98 mL, 96.4 mg, 1.22 mmol, 3.57 equiv.) and NBS (60.8 mg, 341 µmol, 1.00 equiv.) were added to a solution of **SI-4b** (206 mg, 341 µmol, 1.00 equiv.) in $CHCl_3$ (68 mL) at 0 °C, and the reaction mixture was stirred for 1 h. The reaction was quenched by addition of acetone (35 mL), and the solvents were removed under reduced pressure. The crude product was purified by flash column chromatography

(Hex/EtOAc = 4/1) to yield porphyrin **SI-5b** (63.8 mg, 93.7 µmol, 27%) as a purple solid. Due to strong signal broadening, no distinct NMR signals were detected. Therefore, the crude material was used directly in the next step.

***R*f:** 0.31 (CH_2Cl_2/MeOH = 49/1) [vis: purple].

UV-Vis (CH_2Cl_2): λ (nm) = 308 (ε = 15700 $cm^{-1}M^{-1}$), 350 (ε = 12100 $cm^{-1}M^{-1}$), 413 (ε = 456600 $cm^{-1}M^{-1}$), 541 (ε = 19000 $cm^{-1}M^{-1}$).

2.9. 5-Bromo-10,20-dimesityl-15-phenylporphyrinzinc(II) (SI-5c)

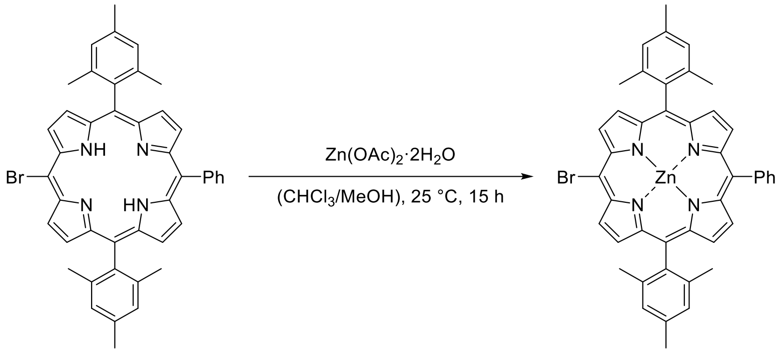

According to a modified procedure by *Frost et al.*,[2] $Zn(OAc)_2 \cdot 2H_2O$ (109 mg, 499 µmol, 5.00 equiv.) was added to a solution of 5-bromo-10,20-dimesityl-15-phenylporphyrin[6] (70.0 mg, 99.8 µmol, 1.00 equiv.) in $CHCl_3$ (12 mL) and MeOH (6 mL), and the reaction mixture was stirred at room temperature for 15 h. The solvent was removed under reduced pressure, and the crude product was purified by flash column chromatography (pentane/CH_2Cl_2 = 1/0 → 1/4) to yield porphyrin **SI-5c** (218 mg, 362 µmol, 95%) as a pink solid. Due to strong signal broadening, no distinct NMR signals were detected. Therefore, the crude material was used directly in the next step.

***R*f:** 0.39 (pentane/CH_2Cl_2 = 3/2) [vis: pink].

IR (ATR): ṽ [cm^{-1}] = 3067 (w), 2921 (w, C–H), 2868 (w, C–H), 1646 (m, C=N), 1610 (m, C=C), 1599 (m, C=C), 1454 (m), 1251 (m), 1093 (m, C–Br), 1049 (m), 1022 (m), 858 (m), 840 (m), 812 (m), 753 (s, C–H), 705 (s, C–H), 667 (vs).

UV-Vis (CH_2Cl_2): λ (nm) = 422 (ε = 473700 $cm^{-1}M^{-1}$), 553 (ε = 21500 $cm^{-1}M^{-1}$), 590 (ε = 7100 $cm^{-1}M^{-1}$), 652 (ε = 5800 $cm^{-1}M^{-1}$).

2.10. 5-Bromo-10,20-bis(4-methoxyphenyl)-15-phenylporphyrin zinc(II) (SI-5d)

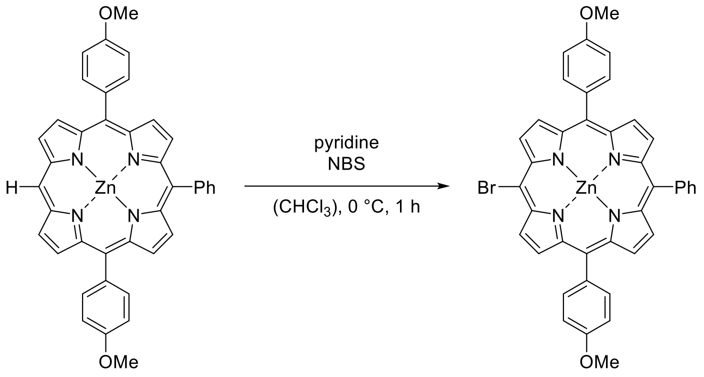

According to a modified procedure by *Frost et al.*,[2] pyridine (0.01 mL, 8.96 mg, 113 µmol, 3.57 equiv.) and NBS (5.65 mg, 31.7 µmol, 1.00 equiv.) were added to a solution of **SI-4d** (21.0 mg, 31.7 µmol, 1.00 equiv.) in $CHCl_3$ (3.2 mL) at 0 °C, and the reaction mixture was stirred for 1 h. The reaction was quenched by addition of acetone (10 mL), and the solvents were removed under reduced pressure. The crude product was purified by flash column chromatography (pentane/CH_2Cl_2 = 7/3 → 2/3) to yield porphyrin **SI-5d** (22.5 mg, 30.4 µmol, 95%) as a purple solid. Due to strong signal broadening, no distinct NMR signals were detected. Therefore, the crude material was used directly in the next step.

***R*f:** 0.45 (pentane/CH_2Cl_2 = 1/1) [vis: purple].

IR (ATR): ṽ [cm^{-1}] = 3002 (w), 2956 (w, C–H), 2932 (w, C–H), 2906 (w, C–H), 2833 (w, C–H), 1603 (w, C=N), 1573 (w, C=C), 1505 (w, C=C), 1465 (w), 1247 (s, C–O), 1207 (w), 1179 (s, C–O), 1074 (w, C–Br), 802 (s), 792 (s), 785 (vs, C–H), 756 (s, C–H), 740 (s, C–H), 728 (s, C–H), 719 (s, C–H), 702 (s), 678 (s), 667 (s).

UV-Vis (CH_2Cl_2): λ (nm) = 423 (ε = 408900 $cm^{-1}M^{-1}$), 553 (ε = 18600 $cm^{-1}M^{-1}$), 593 (ε = 7400 $cm^{-1}M^{-1}$).

2.11. 5-((3aS,4R,7S,7aR,8R)-8-Ethynyl)octahydro-1H-4,7-methanoisoindol-1-on-9-yl)-10,15,20-triphenylporphyrin zinc(II) (7)

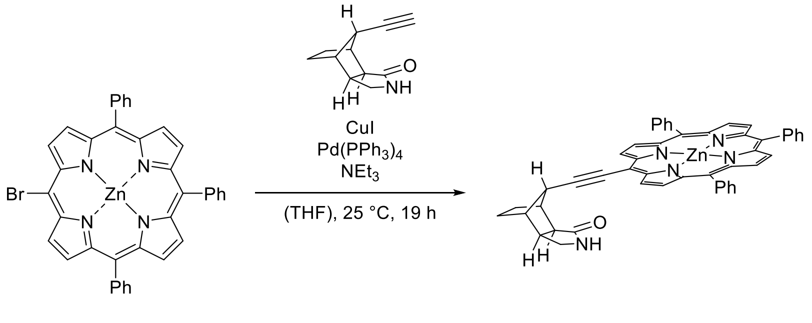

According to a modified procedure by *Burg et al.*,[7] porphyrin **SI-5b** (200 mg, 293 µmol, 1.00 equiv.) and (3a*S*,4*R*,7*S*,7a*R*,8*R*)-8-ethynyloctahydro-1*H*-4,7-methanoisoindol-1-one[8] (61.6 mg, 352 µmol, 1.20 equiv.) were dissolved in THF (17 mL) and NEt_3 (17 mL), and the mixture was degassed by freeze-pump-thaw cycles (3×). CuI (16.8 mg, 88.1 µmol, 30 mol%) and $Pd(PPh_3)_4$ (50.9 mg, 44.1 µmol, 15 mol%) were added sequentially, and the reaction mixture was stirred at room temperature for 19 h. The solvents were removed under reduced pressure, and the residue was dissolved in CH_2Cl_2 (25 mL). The organic layer was washed with H_2O (25 mL), dried over Na_2SO_4, filtered, and the solvent

was removed under reduced pressure. The crude product was purified by flash column chromatography (CH_2Cl_2/MeOH = 1/0 → 97/3) to yield porphyrin **SI-6b** (184 mg, 237 µmol, 81%) as a teal solid.

***R*f:** 0.31 (CH_2Cl_2/MeOH = 49/1) [vis: teal].

^{1}H NMR ($CDCl_3$, 500 MHz, 300 K): δ [ppm] = 1.42 – 1.52 (m, 2 H), 1.77 – 1.87 (m, 2 H), 2.45 – 2.59 (m, 2 H), 2.70 – 2.82 (m, 1 H), 3.06 – 3.14 (m, 2 H), 3.36 – 3.48 (m, 1 H), 3.77 – 3.89 (m, 1 H), 5.10 (br s, 1 H, NH), 7.64 – 7.82 (m, 9 H, H_{Ph}), 8.00 (br s, 2 H), 8.11 – 8.12 (m, 6 H, H_{Ph}), 8.55 (br s, 2 H), 8.84 (d, ^3J = 4.6 Hz, 2 H), 8.85 (d, ^3J = 4.6 Hz, 2 H).

^{13}C NMR ($CDCl_3$, 126 MHz, 300 K): δ [ppm] = 207.2, 178.3, 152.5, 150.4, 150.0, 149.8, 143.0, 142.8, 134.6 (2C), 134.5, 133.0, 132.1, 131.9, 130.9, 128.5, 128.4, 128.2, 127.6, 126.7, 126.6, 122.3, 121.7, 99.7, 94.0, 85.2, 50.6, 47.3, 44.6, 41.8, 39.9, 31.1, 28.9, 28.8.

IR (ATR): $\tilde{\nu}$ [cm^{-1}] = 3054 (w), 2953 (m, C–H), 2878 (w, C–H), 1669 (s, C=O), 1597 (m, C=C), 1586 (m), 1440 (s), 1340 (s), 1202 (s), 1069 (s), 1002 (vs), 993 (vs), 796 (C–H), 751 (s, C–H), 723 (s, C–H), 701 (vs), 663 (w).

UV-Vis (CH_2Cl_2): λ (nm) = 312 (ε = 15200 cm^{-1}M^{-1}), 428 (ε = 355500 cm^{-1}M^{-1}), 560 (ε = 14800 cm^{-1}M^{-1}), 599 (ε = 8500 cm^{-1}M^{-1}), 662 (ε = 1600 cm^{-1}M^{-1}).

2.12. 5-((3aS,4R,7S,7aR,8R)-8-Ethynyl)octahydro-1H-4,7-methanoisoindol-1-on-9-yl)-10,20-dimesityl-15-phenylporphyrin zinc(II) (SI-6c)

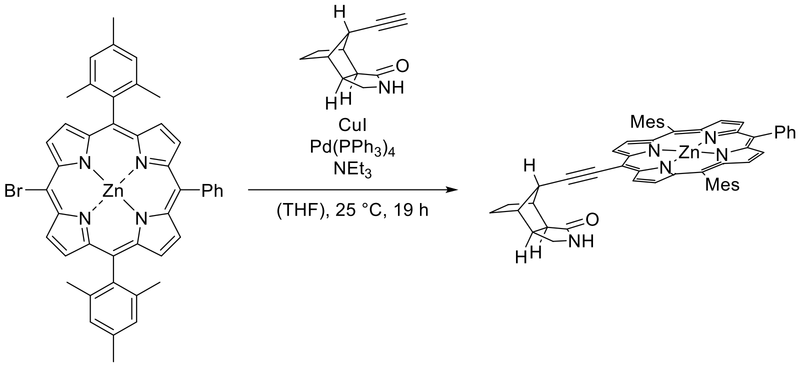

According to a modified procedure by *Burg et al.*,[7] porphyrin **SI-5c** (59.0 mg, 77.1 µmol, 1.00 equiv.) and (3a*S*,4*R*,7*S*,7a*R*,8*R*)-8-ethynyloctahydro-1*H*-4,7-methanoisoindol-1-one[8] (16.2 mg, 92.5 µmol, 1.20 equiv.) were dissolved in THF (4.5 mL) and NEt_3 (4.5 mL), and the mixture was degassed by freeze-pump-thaw cycles (3×). CuI (4.41 mg, 23.1 µmol, 30 mol%) and $Pd(PPh_3)_4$ (13.4 mg, 11.6 µmol, 15 mol%) were added sequentially, and the reaction mixture was stirred at room temperature for 19 h. The solvents were removed under reduced pressure, and the residue was dissolved in CH_2Cl_2 (25 mL). The organic layer was washed with H_2O (25 mL), dried over Na_2SO_4, filtered, and the solvent was removed under reduced pressure. The crude product was purified by flash column chromatography (CH_2Cl_2/MeOH = 99/1 → 24/1) to yield porphyrin **SI-6c** (66.3 mg, 77.1 µmol, >99%) as a teal solid. The crude product was directly subjected to the next step.

***R*f:** 0.55 (CH_2Cl_2/MeOH = 9/1) [vis: teal].

IR (ATR): ṽ [cm^{-1}] = 2948 (w, C–H), 2916 (w, C–H), 2876 (w, C–H), 1667 (s, C=O), 1610 (w, C=N), 1597 (w, C=C), 1574 (w, C=C), 1547 (w, C=C), 1437 (s), 1283 (s), 1059 (s), 830 (s), 795 (vs), 755 (s, C–H), 746 (s, C–H), 718 (vs, C–H).

UV-Vis (CH_2Cl_2): λ (nm) = 429 (ε = 578600 cm^{-1}M^{-1}), 560 (ε = 23100 cm^{-1}M^{-1}), 601 (ε = 15600 cm^{-1}M^{-1}).

2.13. 5-((3aS,4R,7S,7aR,8R)-8-Ethynyl)octahydro-1H-4,7-methanoisoindol-1-on-9-yl)-10,20-bis(4-methoxyphenyl)-15-phenylporphyrin zinc(II) (SI-6d)

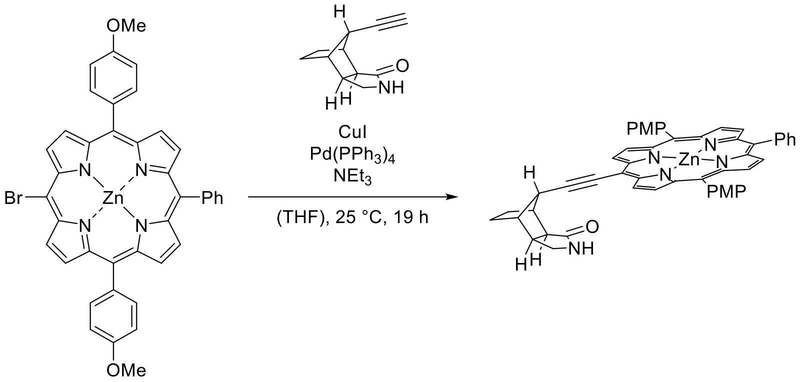

According to a modified procedure by *Burg et al.*,[7] porphyrin **SI-5d** (161 mg, 217 µmol, 1.00 equiv.) and (3a*S*,4*R*,7*S*,7a*R*,8*R*)-8-ethynyloctahydro-1*H*-4,7-methanoisoindol-1-one[8] (45.7 mg, 261 µmol, 1.20 equiv.) were dissolved in THF (13 mL) and NEt_3 (13 mL), and the mixture was degassed by freeze-pump-thaw cycles (3×). CuI (12.4 mg, 65.2 µmol, 30 mol%) and $Pd(PPh_3)_4$ (37.6 mg, 32.6 µmol, 15 mol%) were added sequentially, and the reaction mixture was stirred at room temperature for 19 h. The solvents were removed under reduced pressure, and the residue was dissolved in CH_2Cl_2 (25 mL). The organic layer was washed with H_2O (25 mL), dried over Na_2SO_4, filtered, and the solvent was removed under reduced pressure. The crude product was purified by flash column chromatography (CH_2Cl_2/MeOH = 99/1 → 97/3) to yield porphyrin **SI-6d** (179 mg, 215 µmol, >99%) as a teal solid. The crude product was directly subjected to the next step.

***R*f:** 0.31 (CH_2Cl_2/MeOH = 99/1) [vis: teal].

IR (ATR): ṽ [cm^{-1}] = 2951 (s, C–H), 2917 (s, C–H), 2878 (s, C–H), 2837 (m, C–H), 1690 (s), 1670 (vs, C=O), 1604 (s, C=N), 1573 (m, C=C), 1523 (s, C=C), 1466 (s), 1245 (vs, C–O), 1202 (s), 1119 (s, C–O), 834 (m), 808 (vs), 795 (vs), 756 (s, C–H), 730 (vs, C–H), 715 (vs, C–H).

UV-Vis (CH_2Cl_2): λ (nm) = 430 (ε = 403100 cm^{-1}M^{-1}), 562 (ε = 17900 cm^{-1}M^{-1}), 603 (ε = 13700 cm^{-1}M^{-1}).

2.14. 5-((3aS,4R,7S,7aR,8R)-8-Ethynyl)octahydro-1H-4,7-methanoisoindol-1-on-9-yl)-10,15,20-triphenylporphyrin (SI-7b)

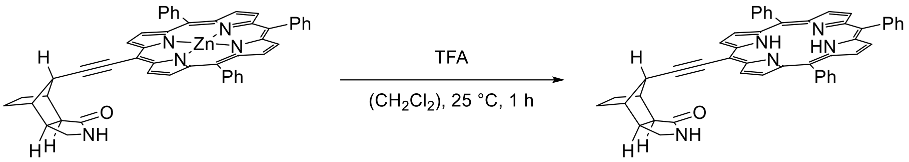

According to a modified procedure by *Burg et al.*,[7] TFA (1.09 mL, 1.62 g, 14.2 mmol, 100 equiv.) was added dropwise to a solution of porphyrin **SI-6b** (110 mg, 142 µmol, 1.00 equiv.) in CH_2Cl_2 (71 mL), and the reaction mixture was stirred at room temperature for 1 h. The reactions was quenched by addition of sat. $NaHCO_3$ solution (pH = 7), and the aqueous layer was extracted with CH_2Cl_2 (3 × 25 mL). The organic layer was dried over Na_2SO_4, filtered, and the solvent was removed under reduced pressure. The crude product was purified by flash column chromatography (CH_2Cl_2/MeOH = 1/0 → 97/3) to yield porphyrin **SI-7b** (101 mg, 142 µmol, >99%) as a purple solid.

$\boldsymbol{R_f}$**:** 0.15 (pentane/ac = 3/2) [vis: purple].

^{1}H NMR ($CDCl_3$, 500 MHz, 300 K): δ [ppm] = –2.50 (s, 2H, NH), 1.34 – 1.43 (m, 1 H), 1.82 – 1.94 (m, 2 H), 2.28 – 2.37 (m, 1 H), 2.58 – 2.65 (m, 1 H), 2.81 – 2.86 (m, 1 H), 2.96 – 3.02 (m, 1 H), 3.17 (s, 1 H), 3.49 (t, ^3J = 9.7 Hz, 1 H), 3.87 – 3.93 (m, 1 H), 5.06 (br s, 1 H, NH), 7.70 – 7.80 (m, 9 H), 8.10 – 8.22 (m, 6 H), 8.83 – 8.87 (m, 4 H), 8.93 (d, ^3J = 4.0 Hz, 2 H), 9.58 (d, ^3J = 4.0 Hz, 2 H).

^{13}C NMR ($CDCl_3$, 126 MHz, 300 K): δ [ppm] = 152.5, 150.4, 150.0, 149.8, 143.0, 142.8, 134.6, 134.5, 133.1, 132.1, 132.0, 131.9, 130.9, 128.5, 128.4, 127.6, 126.7, 126.7, 122.3, 121.7, 50.2, 47.4, 44.7, 41.8, 39.9, 31.1, 28.9, 28.8.

IR (ATR): ṽ [cm^{-1}] = 3055 (w), 2955 (s, C–H), 2926 (s, C–H), 1669 (vs, C=O), 1597 (w, C=C), 1486 (m), 1440 (m), 1341 (m), 1204 (m), 1068 (m), 1002 (vs), 994 (vs), 796 (vs), 751 (s, C–H), 717 (s, C–H), 701 (vs), 662 (w).

UV-Vis (CH_2Cl_2): λ (nm) = 313 (ε = 16800 $cm^{-1}M^{-1}$), 428 (ε = 534900 $cm^{-1}M^{-1}$), 560 (ε = 20300 $cm^{-1}M^{-1}$), 599 (ε = 13900 $cm^{-1}M^{-1}$).

HRMS (ESI): calculated for $C_{49}H_{38}N_5O^+$ $[M+H]^+$: 712.3071; found: 712.3060.

2.15. 5-((3aS,4R,7S,7aR,8R)-8-Ethynyl)octahydro-1H-4,7-methanoisoindol-1-on-9-yl)-10,20-dimesityl-15-phenylporphyrin (SI-7c)

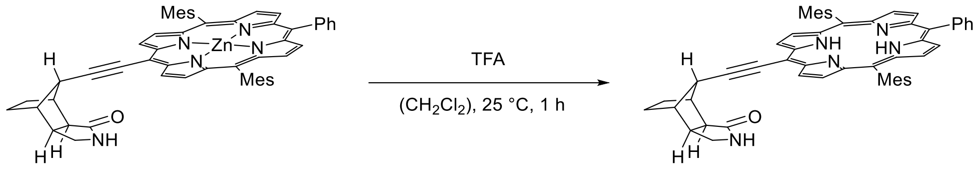

According to a modified procedure by *Burg et al.*,[7] TFA (0.33 mL, 488 mg, 4.28 mmol, 100 equiv.) was added dropwise to a solution of porphyrin **SI-6c** (36.8 mg, 42.8 µmol, 1.00 equiv.) in CH_2Cl_2 (21 mL), and the reaction mixture was stirred at room temperature for 2 h. The reactions was quenched by addition of sat. $NaHCO_3$ solution (pH = 7), and the aqueous layer was extracted with CH_2Cl_2 (3 × 25 mL). The organic layer was dried over Na_2SO_4, filtered, and the solvent was removed under reduced pressure. The crude product was purified by flash column chromatography (CH_2Cl_2/MeOH = 99/1 → 49/1) to yield porphyrin **SI-7b** (32.1 mg, 40.3 µmol, 94%) as a purple solid.

***R_f*:** 0.59 (CH_2Cl_2/ac = 19/1) [vis: purple].

^{1}H NMR ($CDCl_3$, 500 MHz, 300 K): δ [ppm] = –2.35 (bs, 2H, NH), 1.43 (dd, ^2J = 14.8 Hz, ^3J = 6.0 Hz, 1H), 1.48 – 1.52 (m, 1H), 1.82 (s, 6H, CH_3), 1.84 (s, 6H, CH_3), 1.91 – 1.99 (m, 2H), 2.63 (s, 6H, CH_3), 2.74 – 2.80 (m, 2H), 2.93 (d, ^3J = 3.7 Hz, 1H), 3.27 (s, 1H), 3.36 (d, ^3J = 3.7 Hz, 1H), 3.62 – 3.66 (m, 1H), 4.01 – 4.04 (m, 1H), 5.26 (bs, 1H, NH), 7.28 (s, 4H, H_{Ar}), 7.69 – 7.77 (m, 3H, H_{Ar}), 8.17 – 8.19 (m, 2H, H_{Ar}), 8.61 (d, ^3J = 4.7 Hz, 2H, H_{Ar}), 8.71 (d, ^3J = 4.7 Hz, 2H, H_{Ar}), 8.75 (d, ^3J = 4.7 Hz, 2H, H_{Ar}), 9.62 (d, ^3J = 4.7 Hz, 2H, H_{Ar}).

^{13}C NMR ($CDCl_3$, 126 MHz, 300 K): δ [ppm] = 179.0, 141.9, 139.4, 139.4, 138.2, 137.9, 134.5, 132.3, 132.2, 128.7, 128.6, 127.9, 127.9, 126.8, 120.8, 119.1, 99.4, 95.7, 84.5, 50.6, 47.7, 47.0, 44.8, 42.3, 40.1, 29.1, 28.9, 21.8, 21.7, 21.6.

IR (ATR): ṽ [cm^{-1}] = 2944 (m, C–H), 2918 (m, C–H), 2871 (w, C–H), 1701 (s), 1667 (s, C=O), 1610 (m, C=N), 1558 (w, C=C), 1515 (w, C=C), 1440 (m), 1253 (m), 967 (m), 826 (m), 798 (vs), 756 (m, C–H), 741 (s, C–H), 718 (vs, C–H), 702 (vs), 657 (m).

UV-Vis (CH_2Cl_2): λ (nm) = 427 (ε = 326200 $cm^{-1}M^{-1}$), 526 (ε = 14100 $cm^{-1}M^{-1}$), 564 (ε = 17600 $cm^{-1}M^{-1}$), 603 (ε = 6900 $cm^{-1}M^{-1}$), 661 (ε = 8400 $cm^{-1}M^{-1}$).

HRMS (ESI): calculated for $C_{55}H_{50}N_5O^+$ $[M+H]^+$: 796.4010; found: 796.4003.

2.16. 5-((3aS,4R,7S,7aR,8R)-8-Ethynyl)octahydro-1H-4,7-methanoisoindol-1-on-9-yl)-10,20-bis(4-methoxyphenyl)-15-phenylporphyrin (SI-7d)

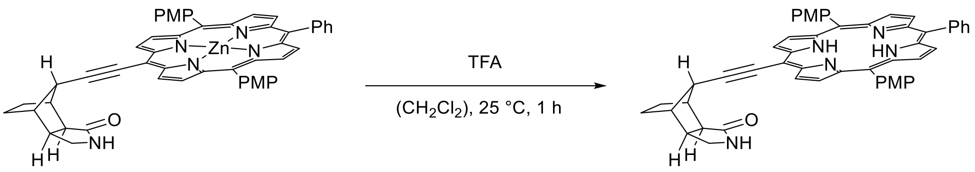

According to a modified procedure by *Burg et al.*,[7] TFA (0.46 mL, 684 mg, 6.00 mmol, 100 equiv.) was added dropwise to a solution of porphyrin **SI-6d** (50.1 mg, 60.0 µmol, 1.00 equiv.) in CH_2Cl_2 (30 mL), and the reaction mixture was stirred at room temperature for 2 h. The reactions was quenched by addition of sat. $NaHCO_3$ solution (pH = 7), and the aqueous layer was extracted with CH_2Cl_2 (3 × 25 mL). The organic layer was dried over Na_2SO_4, filtered, and the solvent was removed under reduced pressure. The crude product was purified by flash column chromatography (CH_2Cl_2/ac = 19/1 → 4/1) to yield porphyrin **SI-6c** (19.8 mg, 27.4 µmol, 43%) as a purple solid.

***R*f:** 0.21 (CH_2Cl_2/ac = 23/2) [vis: purple].

^{1}H NMR ($CDCl_3$, 500 MHz, 300 K): δ [ppm] = –2.48 (bs, 2H, NH), 1.42 – 1.51 (m, 2H), 1.82 (s, 6H, CH_3), 1.91 – 2.00 (m, 2H), 2.73 – 2.78 (m, 2H), 2.94 (d, ^3J = 3.7 Hz, 1H), 3.29 (s, 1H), 3.39 (d, ^3J = 3.7 Hz, 1H), 3.59 – 3.62 (m, 1H), 3.98 – 4.00 (m, 1H), 4.11 (s, 6H, OCH_3), 5.14 (bs, 1H, NH), 7.28 – 7.30 (m, 4H, H_{Ar}), 7.71 – 7.78 (m, 3H, H_{Ar}), 8.16 – 8.18 (m, 2H, H_{Ar}), 8.75 (d, ^3J = 4.7 Hz, 2H, H_{Ar}), 8.79 (d, ^3J = 4.7 Hz, 2H, H_{Ar}), 8.95 (d, ^3J = 4.7 Hz, 2H, H_{Ar}), 9.68 (d, ^3J = 4.7 Hz, 2H, H_{Ar}).

^{13}C NMR ($CDCl_3$, 126 MHz, 300 K): δ [ppm] = 179.0, 159.6, 142.2, 135.7, 134.6, 134.3, 126.8, 121.5, 120.7, 112.4, 99.9, 96.0, 84.8, 55.8, 50.6, 47.7, 46.9, 44.8, 42.3, 40.1, 29.1, 28.9.

IR (ATR): ṽ [cm^{-1}] = 3316 (w, N–H), 2958 (w, C–H), 2931 (w, C–H), 2900 (w, C–H), 1648 (vs, C=O), 1602 (s, C=N), 1573 (w, C=C), 1558 (w, C=C), 1515 (m, C=C), 1458 (m), 1304 (w), 1243 (vs, C–O), 1121 (w, C–O), 981 (w), 970 (vs), 917 (w), 815 (s), 808 (s), 796 (vs), 784 (vs, C–H), 752 (s, C–H), 739 (s, C–H), 725 (vs), 702 (vs).

UV-Vis (CH_2Cl_2): λ (nm) = 428 (ε = 379500 cm^{-1}M^{-1}), 529 (ε = 16800 cm^{-1}M^{-1}), 567 (ε = 22300 cm^{-1}M^{-1}), 604 (ε = 8200 cm^{-1}M^{-1}), 663 (ε = 10600 cm^{-1}M^{-1}).

HRMS (ESI): calculated for $C_{51}H_{42}N_5O_3^+$ [M+H]$^+$: 772.3282; found: 772.3275.

2.17. 5-((3a*S*,4*R*,7*S*,7a*R*,8*R*)-8-Ethynyloctahydro-1*H*-4,7-methanoisoindol-1-on)-10,15,20-tris(pentafluorphenyl)porphyrin cobalt(II) (6a)

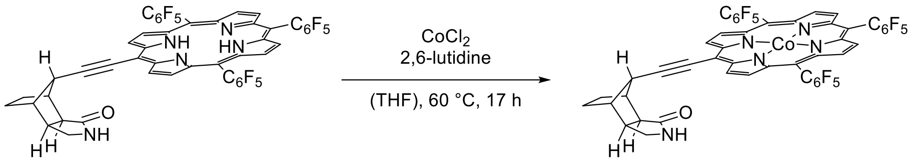

According to a modified procedure by *Wang et al.*,[9] 2,6-lutidine (0.04 mL, 32.8 mg, 306 µmol, 10.0 equiv.), and anhydrous $CoCl_2$ (99.2 mg, 764 µmol, 25.0 equiv.) was added to a solution of 5-((3a*S*,4*R*,7*S*,7a*R*,8*R*)-8-ethynyloctahydro-1*H*-4,7-methanoisoindol-1-on)-10,15,20- tris(pentafluorphenyl)porphyrin[7] (30.0 mg, 30.6 µmol, 1.00 equiv.) in THF (3.0 mL) and the reaction mixture was heated to 60 °C for 17 h. The solvent was removed under reduced pressure, and the residue was dissolved in CH_2Cl_2 (25 mL). The organic layer was washed with H_2O (25 mL), dried over Na_2SO_4, filtered, and the solvent was removed under reduced pressure. The crude product was purified by flash column chromatography (pentane/ac = 9/1 → 7/3) to yield porphyrin **6a** (25.8 mg, 24.8 µmol, 81%) as a red solid.

***R*f:** 0.24 (pentane/ac = 7/3) [vis: rot].

EA (CHN): calculated for $C_{49}H_{20}CoF_{15}N_5O$ (%): C 56.66, H 1.94, N 6.74; found (%): C 53.29, H 2.61, N 6.17.

IR (ATR): $\tilde{\nu}$ [cm^{-1}] = 2927 (m), 2856 (w), 1665 (s, C=O), 1519 (vs, C=C), 1491 (vs, C=C), 1351 (m), 1079 (m, C–F), 1059 (m), 987 (vs), 939 (s), 762 (m), 702 (m).

UV-Vis (CH_2Cl_2): λ (nm) = 270 (ε = 30000 cm^{-1}M^{-1}), 317 (ε = 23200 cm^{-1}M^{-1}), 419 (ε = 431400 cm^{-1}M^{-1}), 539 (ε = 23200 cm^{-1}M^{-1}).

HRMS (ESI): calculated for $C_{49}H_{21}CoF_{15}N_5O^+$ [M+H]$^+$: 1039.0833; found: 1039.0833.

2.18. 5-((3aS,4R,7S,7aR,8R)-8-Ethynyl)octahydro-1H-4,7-methanoisoindol-1-on-9-yl)-10,15,20-triphenylporphyrin cobalt(II) (6b)

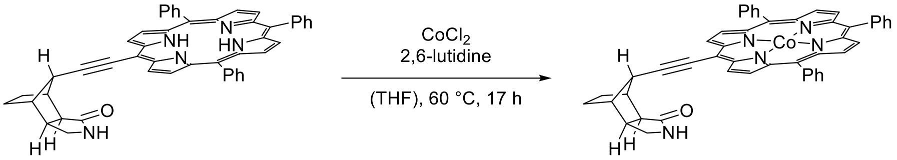

According to a modified procedure by *Wang et al.*,[9] 2,6-lutidine (0.15 mL, 143 mg, 1.33 mmol, 10.0 equiv.), and anhydrous $CoCl_2$ (433 mg, 3.34 mmol, 25.0 equiv.) was added to a solution of porphyrin **SI-7b** (95.0 mg, 133 µmol, 1.00 equiv.) in THF (13 mL) and the reaction mixture was heated to 60 °C for 17 h. The solvent was removed under reduced pressure, and the residue was dissolved in CH_2Cl_2 (25 mL). The organic layer was washed with H_2O (25 mL), dried over Na_2SO_4, filtered, and the solvent was removed under reduced pressure. The crude product was purified by flash column chromatography (CH_2Cl_2/ac = 9/1) to yield porphyrin **6b** (60.9 mg, 79.2 µmol, 59%) as a red solid.

***R*f:** 0.60 (CH_2Cl_2/ac = 9/1) [vis: red].

EA (CHN): calculated for $C_{49}H_{35}CoN_5O$ (%): C 76.55, H 4.59, N 9.11; found (%): C 74.31, H 4.58, N 8.63.

IR (ATR): $\tilde{\nu}$ [cm^{-1}] = 3053 (w, N–H), 2947 (w, C–H), 2876 (w, C–H), 1695 (s, C=O), 1598 (w, C=C), 1491 (w), 1441 (w), 1352 (w), 1301 (w), 1279 (w), 1261 (w), 1205 (w), 1177 (w), 1071 (w), 1005 (s), 834 (m), 796 (s), 752 (m, C–H), 736 (w, C–H), 700 (vs), 669 (w), 655 (w).

UV-Vis (CH_2Cl_2): λ (nm) = 422 (ε = 110400 $cm^{-1}M^{-1}$), 549 (ε = 13700 $cm^{-1}M^{-1}$).

HRMS (ESI): calculated for $C_{49}H_{36}N_5O^+$ $[M+H]^+$: 769.2246; found: 769.2198.

2.19. 5-((3aS,4R,7S,7aR,8R)-8-Ethynyl)octahydro-1H-4,7-methanoisoindol-1-on-9-yl)-10,20-dimesityl-15-phenylporphyrin cobalt(II) (6c)

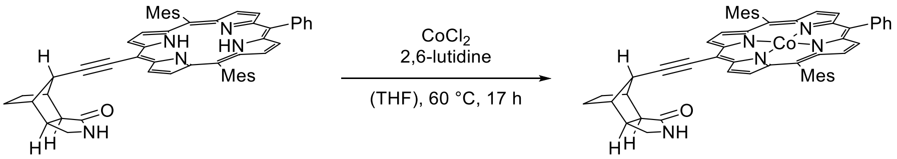

According to a modified procedure by *Wang et al.*,[9] 2,6-lutidine (0.04 mL, 40.4 mg, 377 µmol, 10.0 equiv.), and anhydrous $CoCl_2$ (122 mg, 942 µmol, 25.0 equiv.) was added to a solution of porphyrin **SI-7c** (30.0 mg, 37.7 µmol, 1.00 equiv.) in THF (3.8 mL) and the reaction mixture was heated to 60 °C for 17 h. The solvent was removed under reduced pressure, and the residue was dissolved in CH_2Cl_2 (25 mL). The organic layer was washed with H_2O (25 mL), dried over Na_2SO_4, filtered, and the solvent was removed under reduced pressure. The crude product was purified by flash column chromatography (CH_2Cl_2/ac = 19/1 → 93/7) to yield porphyrin **6c** (26.3 mg, 30.8 µmol, 82%) as a red solid.

***R_f*:** 0.54 (CH_2Cl_2/ac = 19/1) [vis: red].

EA (CHN): calculated for $C_{55}H_{47}CoN_5O$ (%): C 77.45, H 5.55, N 8.21; found (%): C 74.63, H 5.35, N 7.60.

IR (ATR): $\tilde{\nu}$ [cm^{-1}] = 3200 (w, N–H), 2963 (w, C–H), 2945 (w, C–H), 2919 (w, C–H), 2872 (w, C–H), 1666 (s, C=O), 1611 (w, C=N), 1596 (w, C=C), 1574 (w, C=C), 1544 (w, C=C), 1535 (w, C=C), 1456 (m), 1182 (w), 953 (w), 834 (m), 798 (vs), 757 (m, C–H), 741 (m, C–H), 732 (m, C–H), 724 (m), 711 (vs), 703 (vs).

UV-Vis (CH_2Cl_2): λ (nm) = 422 (ε = 316700 cm^{-1}M^{-1}), 540 (ε = 19300 cm^{-1}M^{-1}), 572 (ε = 10100 cm^{-1}M^{-1}).

HRMS (ESI): calculated for $C_{55}H_{48}CoN_5O^+$ [M+H]$^+$: 853.3185; found: 853.3145.

2.20. 5-((3aS,4R,7S,7aR,8R)-8-Ethynyl)octahydro-1H-4,7-methanoisoindol-1-on-9-yl)-10,20-bis(4-methoxyphenyl)-15-phenylporphyrin cobalt(II) (6d)

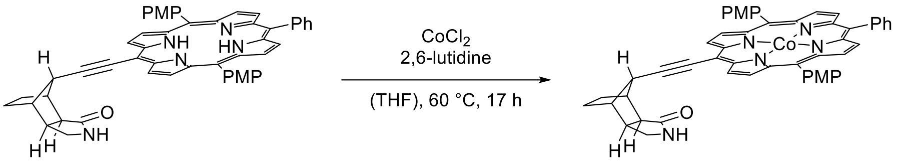

According to a modified procedure by *Wang et al.*,[9] 2,6-lutidine (0.04 mL, 36.7 mg, 342 µmol, 10.0 equiv.), and anhydrous $CoCl_2$ (111 mg, 855 µmol, 25.0 equiv.) was added to a solution of porphyrin **SI-7d** (26.4 mg, 34.2 µmol, 1.00 equiv.) in THF (3.4 mL) and the reaction mixture was heated to 60 °C for 17 h. The solvent was removed under reduced pressure, and the residue was dissolved in CH_2Cl_2 (25 mL). The organic layer was washed with H_2O (25 mL), dried over Na_2SO_4, filtered, and the solvent was removed under reduced pressure. The crude product was purified by flash column chromatography (CH_2Cl_2/ac = 19/1 → 93/7) to yield porphyrin **6d** (22.5 mg, 27.2 µmol, 79%) as a red solid.

***R*f:** 0.47 (CH_2Cl_2/ac = 19/1) [vis: red].

EA (CHN): calculated for $C_{51}H_{39}CoN_5O_3$ (%): C 73.91, H 4.74, N 8.45; found (%): C 73.89, H 4.58, N 8.25.

IR (ATR): ṽ [cm^{-1}] = 2955 (w, C–H), 2922 (w, C–H), 2851 (w, C–H), 1605 (s, C=O), 1573 (w, C=C), 1525 (w, C=C), 1509 (w, C=C), 1456 (w), 1327 (w), 1247 (s, C–O), 1106 (m), 1067 (m, C–O), 811 (m), 796 (vs), 788 (s, C–H), 755 (m, C–H), 745 (w), 724 (w), 717 (m).

UV-Vis (CH_2Cl_2): λ (nm) = 422 (ε = 86800 cm^{-1}M^{-1}), 456 (ε = 68400 cm^{-1}M^{-1}), 559 (ε = 17700 cm^{-1}M^{-1}).

HRMS (ESI): calculated for $C_{53}H_{43}CoN_6O_3^+$ [M+MeCN+H]$^+$: 870.2723; found: 870.2729.

3. Preparation of Hydrazones

3.1. General Procedure 1 (GP 1): Synthesis of 3,4-Dihydroquinazolinones

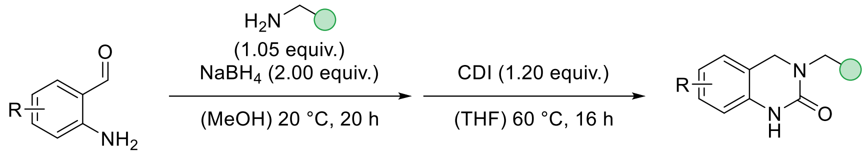

According to a modified procedure by *Pechulis et al.*,[10] the corresponding amines (1.05 equiv.) were added to a solution of 2-aminobenzaldehydes (1.00 equiv.) in MeOH (500 mM) and the corresponding solution was stirred at 20 °C for 4 h. $NaBH_4$ (2.00 equiv.) was added carefully to the reaction mixture while the temperature was maintained using a water bath. The mixture was further stirred for 16 h before addition of H_2O (5 mL). The organic solvent was removed under reduced pressure and the residue was redissolved in CH_2Cl_2 (30 mL) and H_2O (30 mL). The aqueous layer was washed with CH_2Cl_2 (2 × 30 mL) and the combined organic layers were dried over Na_2SO_4, and filtered. The solvent was removed under reduced pressure to obtain crude diamines.

According to a modified procedure by *Thanigaimalai et al.*,[11] the crude products were redissolved in THF (150 mM), and CDI (1.20 equiv.) was added in one portion. The mixture was stirred at 60 °C for 16 h. After cooling to room temperature, the organic layer was washed with sat. LiOH solution (50 mL), aqueous citric acid solution (10 wt%, 50 mL), H_2O (50 mL) and brine (40 mL). The organic layer was dried over Na_2SO_4, filtered and the solvent was removed under reduced pressure. The crude products were subjected to automated flash column chromatography (CH_2Cl_2/MeOH = 49/1 → 19/1) to yield free 3,4-dihydroquinazolinones.

*3.1.1. 3-(3,3-Diethoxypropyl)-3,4-dihydroquinazolin-2(1H)-one (**SI-8a**)*

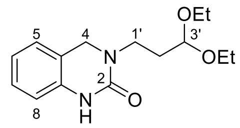

Following GP 1 starting from 2-aminobenzaldehyde (6.31 g, 25.0 mmol), compound **SI-8a** was obtained as a colorless solid (4.78 g, 17.2 mmol, 69%).

***R*f:** 0.42 (CH_2Cl_2/ac = 4/1) [UV, $KMnO_4$].

^{1}H NMR (500 MHz, $CDCl_3$): δ [ppm] = 1.20 (t, ^3J = 7.1 Hz, 6H, CH_3), 1.92 – 2.00 (m, 2H, H-2'), 3.48 – 3.55 (m, 4H, H-1', OCH_2^a), 3.67 (dq, ^2J = 9.3 Hz, ^3J = 7.1 Hz, 2H, OCH_2^b), 4.46 (s, 2H, H-4), 4.59 (t, ^3J = 5.6 Hz, 1H, H-3'), 6.64 (dd, ^3J = 7.6 Hz, ^4J = 1.9 Hz, 1H, H-8), 6.82 (bs, 1H, NH), 6.93 (*virt.* td, $^3J \approx {}^3J$ = 7.6 Hz, ^4J = 1.9 Hz, 1H, H-6), 7.04 (d, ^3J = 7.6 Hz, 1H, H-5), 7.15 (*virt.* td, $^3J \approx {}^3J$ = 7.6 Hz, ^4J = 1.4 Hz, 1H, H-7).

^{13}C NMR (126 MHz, $CDCl_3$): δ [ppm] = 154.2 (C-2), 137.0 (C-8a), 128.3 (C-7), 125.7 (C-5), 122.1 (C-6), 117.9 (C-4a), 113.5 (C-8), 101.4 (C-3'), 61.7 (OCH_2), 49.1 (C-4), 43.7 (C-1'), 31.5 (C-2'), 15.5 (CH_3).

HRMS (+ESI): calc. for $C_{15}H_{22}N_2NaO_3$ $[M+Na]^+$: 301.1523; found: 301.1523.

IR (ATR): $\tilde{\nu}$ [cm^{-1}] = 3209 (w, N–H), 2973 (w, C–H), 2930 (w, C–H), 2906 (w, C–H), 2877 (w, C–H), 1668 (vs, C=O), 1603 (s, C=C), 1516 (w, C=C), 1480 (s), 1376 (m), 1213 (m), 1120 (vs, C–O), 932 (m), 795 (m), 761 (vs, C–H), 751 (vs, C–H), 727 (s, C–H), 704 (vs), 664 (m), 655 (m).

m.p. = 85-87 °C.

*3.1.2. 5-Bromo-3-(3,3-diethoxypropyl)-3,4-dihydroquinazolin-2(1H)-one (**SI-8b**)*

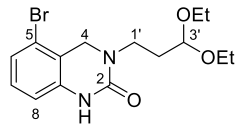

Following GP 1 starting from 2-amino-3-bromobenzaldehyde (1.66 g, 5.00 mmol), compound **SI-8b** was obtained as a colorless solid (327 mg, 916 µmol, 18%).

***R*f:** 0.24 (CH_2Cl_2/ac = 9/1) [UV, $KMnO_4$].

^{1}H NMR (500 MHz, $CDCl_3$): δ [ppm] = 1.21 (t, ^3J = 7.0 Hz, 6H, CH_3), 1.95 – 2.02 (m, 2H, H-2'), 3.49 – 3.57 (m, 4H, H-1', OCH_2^a), 3.68 (dq, ^2J = 9.3 Hz, ^3J = 7.0 Hz, 2H, OCH_2^b), 4.49 (s, 2H, H-4), 4.61 (t, ^3J = 5.6 Hz, 1H, H-3'), 7.01 (*virt.* t, $^3J \approx {}^3J$ = 7.8 Hz, 1H, H-7), 7.17 (bs, 1H, NH), 6.99 (d, ^3J = 7.8 Hz, 1H, H-5), 7.38 (dd, ^3J = 7.8 Hz, ^4J = 1.3 Hz, 1H, H-7).

^{13}C NMR (126 MHz, $CDCl_3$): δ [ppm] = 153.4 (C-2), 135.2 (C-8a), 131.5 (C-7), 124.8 (C-5), 122.8 (C-6), 119.5 (C-4a), 107.8 (C-8), 101.4 (C-3'), 61.8 (OCH_2), 49.2 (C-4), 43.6 (C-1'), 31.4 (C-2'), 15.5 (CH_3).

HRMS (+ESI): calc. for $C_{15}H_{21}BrN_2NaO_3$ $[M+Na]^+$: 379.0628; found: 379.0628.

IR (ATR): $\tilde{\nu}$ [cm^{-1}] = 3193 (w, N–H), 2967 (w, C–H), 2899 (w, C–H), 1678 (vs, C=O), 1607 (w, C=C), 1591 (s, C=C), 1515 (m, C=C), 1446 (m), 1312 (m), 1217 (w), 1127 (m, C–O), 1047 (s, C–Br), 801 (m), 765 (vs, C–H), 745 (s, C–H), 726 (m).

m.p. = 87-89 °C.

*3.1.3. 6-Bromo-3-(3,3-diethoxypropyl)-3,4-dihydroquinazolin-2(1H)-one (**SI-8c**)*

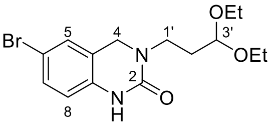

Following GP 1 starting from 2-amino-5-bromobenzaldehyde (1.66 g, 5.00 mmol), compound **SI-8c** was obtained as a colorless solid (1.33 g, 3.71 mmol, 74%).

***R*f:** 0.13 (CH_2Cl_2/ac = 9/1) [UV, $KMnO_4$].

^{1}H NMR (500 MHz, $CDCl_3$): δ [ppm] = 1.20 (t, ^3J = 7.0 Hz, 6H, CH_3), 1.92 – 1.98 (m, 2H, H-2'), 3.47 – 3.55 (m, 4H, H-1', OCH_2^a), 3.67 (dq, ^2J = 9.2 Hz, ^3J = 7.0 Hz, 2H, OCH_2^b), 4.43 (s, 2H, H-4), 4.58 (t, ^3J = 5.6 Hz, 1H, H-3'), 6.75 (d, ^3J = 8.4 Hz, 1H, H-8), 7.16 (d, ^4J = 2.1 Hz, 1H, H-5), 7.25 (dd, ^3J = 8.4 Hz, ^4J = 2.1 Hz, 1H, H-7), 7.40 (bs, 1H, NH).

^{13}C NMR (126 MHz, $CDCl_3$): δ [ppm] = 154.1 (C-2), 136.3 (C-8a), 131.2 (C-7), 128.5 (C-5), 119.9 (C-4a), 115.2 (C-8), 114.0 (C-6), 101.3 (C-3'), 61.7 (OCH_2), 48.6 (C-4), 43.7 (C-1'), 31.4 (C-2'), 15.5 (CH_3).

HRMS (+ESI): calc. for $C_{15}H_{21}BrN_2NaO_3$ $[M+Na]^+$: 379.0628; found: 379.0630.

IR (ATR): $\tilde{\nu}$ [cm^{-1}] = 3198 (w, N–H), 2974 (w, C–H), 2928 (w, C–H), 2900 (w, C–H), 1667 (vs, C=O), 1612 (w), 1601 (w, C=C), 1515 (s, C=C), 1472 (m), 1343 (w), 1276 (w), 1114 (s, C–O), 1064 (vs, C–Br), 945 (w818 (vs), 800 (m), 742 (m, C–H), 706 (w).

m.p. = 132-134 °C.

*3.1.4. 7-Bromo-3-(3,3-diethoxypropyl)-3,4-dihydroquinazolin-2(1H)-one (**SI-8d**)*

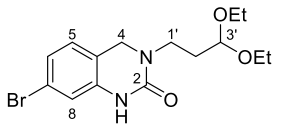

Following GP 1 starting from 2-amino-4-bromobenzaldehyde (6.63 g, 20.0 mmol), compound **SI-8d** was obtained as a colorless solid (6.50 g, 18.2 mmol, 91%).

***R*f:** 0.19 (CH_2Cl_2/ac = 9/1) [UV, $KMnO_4$].

^{1}H NMR (500 MHz, $CDCl_3$): δ [ppm] = 1.20 (t, ^3J = 7.1 Hz, 6H, CH_3), 1.93 – 1.99 (m, 2H, H-2'), 3.48 – 3.55 (m, 4H, H-1', OCH_2^a), 3.67 (dq, ^2J = 9.4 Hz, ^3J = 7.1 Hz, 2H, OCH_2^b), 4.41 (s, 2H, H-4), 4.59 (t, ^3J = 5.6 Hz, 1H, H-3'), 6.85 (d, ^4J = 1.8 Hz, 1H, H-8), 6.89 (d, ^3J = 8.0 Hz, 1H, H-5), 7.04 (dd, ^3J = 8.0 Hz, ^4J = 1.8 Hz, 1H, H-6), 7.55 (bs, 1H, NH).

^{13}C NMR (126 MHz, $CDCl_3$): δ [ppm] = 154.1 (C-2), 138.6 (C-8a), 127.0 (C-5), 124.8 (C-6), 121.6 (C-4a), 116.8 (C-7), 116.5 (C-8), 101.4 (C-3'), 61.8 (OCH_2), 48.7 (C-4), 43.7 (C-1'), 31.4 (C-2'), 15.5 (CH_3).

HRMS (+ESI): calc. for $C_{15}H_{21}BrN_2NaO_3$ $[M+Na]^+$: 379.0628; found: 379.0630.

IR (ATR): $\tilde{\nu}$ [cm^{-1}] = 3334 (w, N–H), 2975 (w, C–H), 2931 (w, C–H), 2878 (w, C–H), 1662 (m, C=O), 1601 (m, C=C), 1517 (m, C=C), 1455 (m), 1316 (m), 1158 (m), 1120 (m, C–O), 1060 (vs, C–Br), 882 (m), 787 (m, C–H), 768 (m, C–H), 759 (m, C–H), 700 (m).

m.p. = 100-102 °C.

*3.1.5. 8-Bromo-3-(3,3-diethoxypropyl)-3,4-dihydroquinazolin-2(1H)-one (**SI-8e**)*

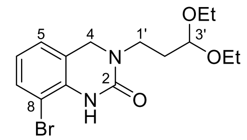

Following GP 1 starting from 2-amino-3-bromobenzaldehyde (1.66 g, 5.00 mmol), compound **SI-8e** was obtained as a colorless solid (1.20 g, 3.35 mmol, 67%).

***R*f:** 0.44 (CH_2Cl_2/ac = 4/1) [UV, $KMnO_4$].

^{1}H NMR (500 MHz, $CDCl_3$): δ [ppm] = 1.20 (t, ^3J = 7.0 Hz, 6H, CH_3), 1.93 – 1.98 (m, 2H, H-2'), 3.47 – 3.55 (m, 4H, H-1', OCH_2^a), 3.67 (dq, ^2J = 9.4 Hz, ^3J = 7.0 Hz, 2H, OCH_2^b), 4.47 (s, 2H, H-4), 4.58 (t, ^3J = 5.6 Hz, 1H, H-3'), 6.81 (*virt.* t, $^3J \approx {}^3J$ = 7.8 Hz, 1H, H-6), 6.89 (bs, 1H, NH), 6.99 (d, ^3J = 7.8 Hz, 1H, H-5), 7.38 (dd, ^3J = 7.8 Hz, ^4J = 1.3 Hz, 1H, H-7).

^{13}C NMR (126 MHz, $CDCl_3$): δ [ppm] = 153.4 (C-2), 135.2 (C-8a), 131.5 (C-7), 124.8 (C-5), 122.8 (C-6), 119.5 (C-4a), 107.8 (C-8), 101.4 (C-3'), 61.8 (OCH_2), 49.2 (C-4), 43.6 (C-1'), 31.4 (C-2'), 15.5 (CH_3).

HRMS (+ESI): calc. for $C_{15}H_{21}BrN_2NaO_3$ $[M+Na]^+$: 379.0628; found: 379.0628.

IR (ATR): $\tilde{\nu}$ [cm^{-1}] = 3193 (m, N–H), 2967 (m, C–H), 2899 (m, C–H), 1678 (vs, C=O), 1607 (m, C=C), 1591 (s, C=C), 1515 (s, C=C), 1446 (s), 1312 (s), 1217 (m), 1127 (s, C–O), 1047 (vs, C–Br), 876 (m), 765 (vs, C–H), 745 (s, C–H), 726 (s, C–H).

m.p. = 50-52 °C.

3.1.6. 6-Chloro-3-(3,3-diethoxypropyl)-3,4-dihydroquinazolin-2(1H)-one ***(SI-8f)***

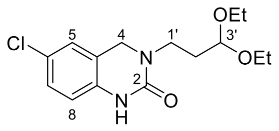

Following GP 1 starting from 2-amino-5-chlorobenzaldehyde (1.00 g, 6.43 mmol), compound **SI-8f** was obtained as a yellow solid (601 mg, 1.92 mmol, 35%).

***R*f:** 0.18 (CH_2Cl_2/ac = 9/1) [UV, $KMnO_4$].

^{1}H NMR (500 MHz, $CDCl_3$): δ [ppm] = 1.20 (t, ^3J = 7.1 Hz, 6H, CH_3), 1.92 – 1.98 (m, 2H, H-2'), 3.47 – 3.55 (m, 4H, H-1', OCH_2^a), 3.67 (dq, ^2J = 9.5 Hz, ^3J = 7.1 Hz, 2H, OCH_2^b), 4.43 (s, 2H, H-4), 4.59 (t, ^3J = 5.6 Hz, 1H, H-3'), 6.62 (dd, ^3J = 8.4 Hz, 1H, H-8), 7.02 (d, ^4J = 2.1 Hz, 1H, H-5), 7.11 (dd, ^3J = 8.4 Hz, ^4J = 2.1 Hz, 1H, H-7), 7.51 (bs, 1H, NH).

^{13}C NMR (126 MHz, $CDCl_3$): δ [ppm] = 154.2 (C-2), 135.9 (C-8a), 128.3 (C-7), 126.8 (C-6), 125.6 (C5), 119.4 (C-4a), 114.8 (C-8), 101.3 (C-3'), 61.7 (OCH_2), 48.7 (C-4), 43.7 (C-1'), 31.4 (C-2'), 15.5 (CH_3).

HRMS (+ESI): calc. for $C_{15}H_{21}ClN_2NaO_3$ $[M+Na]^+$: 335.1133; found: 335.1133.

IR (ATR): $\tilde{\nu}$ [cm^{-1}] = 3196 (w, N–H), 2974 (w, C–H), 2930 (w, C–H), 2909 (w, C–H), 2886 (w, C–H), 2863 (w, C–H), 1662 (m, C=O), 1603 (m, C=C), 1520 (w, C=C), 1499 (w, C=C), 1472 (m), 1343 (m), 1255 (m), 1122 (m, C–O), 1070 (m, C–Cl), 886 (m), 758 (s, C–H), 744 (s, C–H), 702 (vs), 655 (vs).

m.p. = 139-141 °C.

3.1.7. 3-(3,3-Diethoxypropyl)-7-fluoro-3,4-dihydroquinazolin-2(1H)-one ***(SI-8g)***

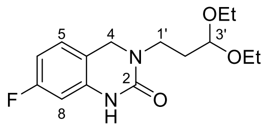

Following GP 1 starting from 2-amino-4-fluorobenzaldehyde (1.00 g, 7.19 mmol), compound **SI-8g** was obtained as a pale-yellow solid (426 mg, 1.75 mmol, 24%).

***R*f:** 0.22 (CH_2Cl_2/ac = 9/1) [UV, $KMnO_4$].

^{1}H NMR (500 MHz, $CDCl_3$): δ [ppm] = 1.20 (t, ^3J = 7.1 Hz, 6H, CH_3), 1.93 – 1.99 (m, 2H, H-2'), 3.48 – 3.55 (m, 4H, H-1', OCH_2^a), 3.67 (dq, ^2J = 9.3 Hz, ^3J = 7.1 Hz, 2H, OCH_2^b), 4.42 (s, 2H, H-4), 4.59 (t, ^3J = 5.6 Hz, 1H, H-3'), 6.41 (dd, $^3J_{H–F}$ = 8.8 Hz, $^4J_{H–H}$ = 2.4 Hz, 1H, H-8), 6.62 (*virt.* td, $^3J_{H–F} \approx {}^3J_{H–H}$ = 8.8 Hz,, $^4J_{H–H}$ = 2.4 Hz, 1H, H-6), 6.97 (dd, $^3J_{H–H}$ = 8.8 Hz, $^4J_{H–F}$ = 5.7 Hz, 1H, H-5), 7.25 (bs, 1H, NH).

^{13}C NMR (126 MHz, $CDCl_3$): δ [ppm] = 162.7 (d, $^1J_{C–F}$ = 244.9 Hz, C-7), 154.0 (C-2), 138.6 (d, $^3J_{C–F}$ = 10.7 Hz, C-8a), 127.0 (d, $^3J_{C–F}$ = 9.7 Hz, C-5), 113.5 (d, $^4J_{C–F}$ = 3.0 Hz, C-4a), 108.6 (d, ^2J = 22.0 Hz, C-6), 101.4 (C-3'), 101.0 (d, ^2J = 25.7 Hz, C-8), 61.7 (OCH_2), 48.5 (C-4), 43.7 (C-1'), 31.4 (C-2'), 15.5 (CH_3).

^{19}F NMR (471 MHz, $CDCl_3$): δ [ppm] = −113.7 (*virt.* td, $^3J_{H–F} \approx {}^3J_{H–F}$ = 8.8 Hz, $^4J_{H–F}$ = 5.7 Hz, 1F).

HRMS (+ESI): calc. for $C_{15}H_{21}FN_2NaO_3$ $[M+Na]^+$: 319.1428; found: 319.1426.

IR (ATR): $\tilde{\nu}$ [cm^{-1}] = 3337 (w, N–H), 2977 (w, C–H), 2953 (w, C–H), 2934 (w, C–H), 2884 (w, C–H), 2821 (w, C–H), 1669 (vs, C=O), 1620 (vs, C=C), 1532 (w, C=C), 1499 (s, C=C), 1447 (w), 1321 (w), 1211 (w), 1117 (m, C–O), 1055 (vs), 854 (vs), 777 (m, C–H), 755 (m, C–H), 700 (w).

m.p. = 103-105 °C.

*3.1.8. 3-(3,3-Diethoxypropyl)-7-(trifluoromethyl)-3,4-dihydroquinazolin-2(1H)-one (**SI-8h**)*

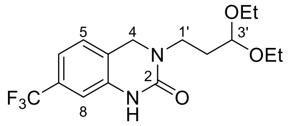

Following a modified GP 1 starting from 2-nitro-4-trifluoromethylbenzaldehyde (2.00 g, 9.13 mmol), the intermediate nitrobenzaldehyde was reduced with Zn dust (8.83 g, 135 mmol, 15.0 equiv.) and NH_4Cl (2.41 g, 45.0 mmol, 5.00 equiv.) in $EtOH/H_2O$ (2/1, 125 mM) at 60 °C for 16 h. The crude product was subjected to carbonylation with CDI to yield compound **SI-8h** as a colorless solid (1.33 g, 3.84 mmol, 49%).

***R*f:** 0.51 (CH_2Cl_2/ac = 4/1) [UV, $KMnO_4$].

^{1}H NMR (500 MHz, $CDCl_3$): δ [ppm] = 1.20 (t, ^3J = 7.0 Hz, 6H, CH_3), 1.94 – 2.00 (m, 2H, H-2'), 3.48 – 3.56 (m, 4H, H-1', OCH_2^a), 3.67 (dq, ^2J = 9.3 Hz, ^3J = 7.0 Hz, 2H, OCH_2^b), 4.51 (s, 2H, H-4), 4.59 (t, ^3J = 5.5 Hz, 1H, H-3'), 6.93 (s, 1H, H-8), 7.14 (d, ^3J = 7.9 Hz, 1H, H-5), 7.18 (d, ^3J = 7.9 Hz, 1H, H-6), 7.80 (bs, 1H, NH).

^{13}C NMR (126 MHz, $CDCl_3$): δ [ppm] = 154.1 (C-2), 137.8 (C-8a), 130.8 (q, $^2J_{C-F}$ = 32.7 Hz, C-7) 126.2 (C-6), 123.9 (q, $^1J_{C-F}$ = 272.4 Hz, CF_3), 121.5 (C-4a), 118.7 (q, $^3J_{C-F}$ = 4.1 Hz, C-6), 110.5 (q, $^3J_{C-F}$ = 3.8 Hz, C-8), 101.4 (C-3'), 61.8 (OCH_2), 48.8 (C-4), 43.7 (C-1'), 31.4 (C-2'), 15.5 (CH_3).

^{19}F NMR (471 MHz, $CDCl_3$): δ [ppm] = −62.8 (s, CF_3).

HRMS (+ESI): calc. for $C_{14}H_{21}N_3NaO_3$ $[M+Na]^+$: 369.1396; found: 369.1398.

IR (ATR): $\tilde{\nu}$ [cm^{-1}] = 3112 (w, N–H), 2978 (w, C–H), 2936 (w, C–H), 2900 (w, C–H), 2882 (w, C–H), 1667 (vs, C=O), 1611 (m, C=C), 1543 (w, C=C), 1534 (w, C=C), 1504 (m, C=C), 1409 (s), 1338 (vs, C–F), 1244 (s), 1115 (vs, C–O), 1005 (s), 883 (m), 793 (m), 748 (m, C–H), 723 (s, C–H), 653 (w).

m.p. = 129-131 °C.

*3.1.9. 3-(3,3-Diethoxypropyl)-8-methyl-3,4-dihydroquinazolin-2(1H)-one (**SI-8k**)*

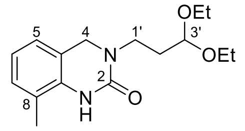

Following GP 1 starting from 2-amino-3-methylbenzaldehyde (250 mg, 1.85 mmol), compound **SI-8k** was obtained as a pale-yellow solid (147 mg, 504 µmol, 27%).

***R*f:** 0.49 (CH_2Cl_2/ac = 4/1) [UV, $KMnO_4$].

^{1}H NMR (500 MHz, $CDCl_3$): δ [ppm] = 1.20 (t, ^3J = 7.1 Hz, 6H, CH_3), 1.93 – 1.99 (m, 2H, H-2'), 2.19 (s, 3H, C-8-CH_3), 3.48 – 3.55 (m, 4H, H-1', OCH_2^a), 3.67 (dq, ^2J = 9.4 Hz, ^3J = 7.1 Hz, 2H, OCH_2^b), 4.45 (s, 2H, H-4), 4.59 (t, ^3J = 5.6 Hz, 1H, H-3'), 6.39 (bs, 1H, NH), 6.85 (*virt.* t, $^3J \approx {}^3J$ = 7.4 Hz, 1H, H-6), 6.90 (d, ^3J = 7.4 Hz, 1H, H-5), 7.39 (d, ^3J = 7.4 Hz, 1H, H-7).

^{13}C NMR (126 MHz, $CDCl_3$): δ [ppm] = 154.1 (C-2), 135.3 (C-8a), 129.6 (C-7), 123.5 (C-5), 121.7 (C-6), 121.0 (C-8), 117.5 (C-4a), 101.4 (C-3'), 61.7 (OCH_2), 49.1 (C-4), 43.7 (C-1'), 31.5 (C-2'), 16.6 (C-8-CH_3), 15.5 (CH_3).

HRMS (+ESI): calc. for $C_{16}H_{24}N_2NaO_3$ $[M+Na]^+$: 315.1679; found: 315.1681.

IR (ATR): $\tilde{\nu}$ [cm^{-1}] = 3185 (w, N–H), 2967 (w, C–H), 2917 (w, C–H), 2873 (w, C–H), 2839 (w, C–H), 1655 (vs, C=O), 1608 (w, C=C), 1577 (w, C=C), 1511 (w, C=C), 1499 (w, C=C), 1446 (m), 1343 (w), 1244 (w), 1105 (s, C–O), 1027 (w), 814 (s), 797 (w), 789 (w, C–H), 755 (w, C–H), 747 (w, C–H), 711 (w).

m.p. = 89-91 °C.

*3.1.10. 3-(3,3-Diethoxypropyl)-6-methoxy-3,4-dihydroquinazolin-2(1H)-one (**SI-8l**)*

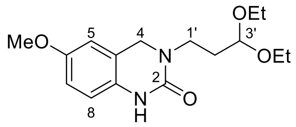

Following a modified GP 1 starting from 5-methoxy-2-nitrobenzaldehyde (1.30 g, 7.20 mmol), the intermediate nitrobenzaldehyde was reduced with Zn dust (7.06 g, 108 mmol, 15.0 equiv.) and NH_4Cl (1.93 g, 36.0 mmol, 5.00 equiv.) in EtOH/H_2O (2/1, 125 mM) at 60 °C for 16 h. The crude product was subjected to carbonylation with CDI to yield compound **SI-8l** as a colorless solid (1.06 g, 3.43 mmol, 48%).

***R*f:** 0.31 (CH_2Cl_2/ac = 9/1) [UV, $KMnO_4$].

^{1}H NMR (500 MHz, $CDCl_3$): δ [ppm] = 1.20 (t, ^3J = 7.1 Hz, 6H, CH_3), 1.93 – 1.99 (m, 2H, H-2'), 3.48 – 3.55 (m, 4H, H-1', OCH_2^a), 3.67 (dq, ^2J = 9.4 Hz, ^3J = 7.1 Hz, 2H, OCH_2^b), 3.76 (s, 3H, OCH_3), 4.43 (s, 2H, H-4), 4.59 (t, ^3J = 5.6 Hz, 1H, H-3'), 6.59 (d, ^4J = 2.5 Hz, 1H, H-5), 6.61 (d, ^3J = 8.7 Hz, 1H, H-8), 6.71 (dd, ^3J = 8.7 Hz, ^4J = 2.5 Hz, 1H, H-7), 7.19 (bs, 1H, NH).

^{13}C NMR (126 MHz, $CDCl_3$): δ [ppm] = 154.9 (C-2), 154.6 (C-6), 130.8 (C-8a), 118.8 (C-4a), 114.5 (C-5), 113.7 (C-7), 111.1 (C-8), 101.4 (C-3'), 61.6 (OCH_2), 55.8 (OCH_3), 49.2 (C-4), 43.6 (C-1'), 31.4 (C-2'), 15.5 (CH_3).

HRMS (+ESI): calc. for $C_{16}H_{24}N_2NaO_4$ $[M+Na]^+$: 331.1628; found: 331.1624.

IR (ATR): $\tilde{\nu}$ [cm^{-1}] = 3202 (w, N–H), 2976 (m, C–H), 2933 (w, C–H), 2906 (w, C–H), 2871 (w, C–H), 1659 (vs, C=O), 1616 (w, C=C), 1508 (vs, C=C), 1433 (m), 1314 (m), 1244 (vs, C–O), 1122 (vs, C–O), 943 (m), 826 (s), 768 (w, C–H), 709 (w).

m.p. = 99-101 °C.

*3.1.11. 3-(3,3-Diethoxypropyl)-8-methoxy-3,4-dihydroquinazolin-2(1H)-one (**SI-8m**)*

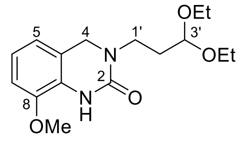

Following GP 1 starting from 2-amino-3-methoxylbenzaldehyde (1.00 g, 6.62 mmol), compound **SI-8m** was obtained as a pale-yellow oil (646 mg, 2.09 mmol, 37%).

***R*f:** 0.27 (CH_2Cl_2/ac = 4/1) [UV, $KMnO_4$].

^{1}H NMR (500 MHz, $CDCl_3$): δ [ppm] = 1.20 (t, ^3J = 7.1 Hz, 6H, CH_3), 1.92 – 1.98 (m, 2H, H-2'), 3.47 – 3.55 (m, 4H, H-1', OCH_2^a), 3.66 (dq, ^2J = 9.3 Hz, ^3J = 7.1 Hz, 2H, OCH_2^b), 3.84 (s, 3H, OCH_3), 4.46 (s, 2H, H-4), 4.58 (t, ^3J = 5.6 Hz, 1H, H-3'), 6.64 (d, ^3J = 7.9 Hz, 1H, H-5), 6.73 (d, ^3J = 7.9 Hz, 1H, H-7), 6.83 (bs, 1H, NH), 6.88 (*virt.* t, $^3J \approx ^3J$ = 7.9 Hz, 1H, H-6).

^{13}C NMR (126 MHz, $CDCl_3$): δ [ppm] = 153.7 (C-2), 145.1 (C-8), 126.7 (C-8a), 121.7 (C-6), 118.1 (C-4a), 117.4 (C-5), 109.3 (C-7), 101.4 (C-3'), 61.7 (OCH_2), 55.8 (OCH_3), 49.1 (C-4), 43.7 (C-1'), 31.5 (C-2'), 15.5 (CH_3).

HRMS (+ESI): calc. for $C_{16}H_{24}N_2NaO_4$ $[M+Na]^+$: 331.1628; found: 331.1628.

IR (ATR): $\tilde{\nu}$ [cm^{-1}] = 3450 (w, N–H), 2973 (w, C–H), 2931 (w, C–H), 2880 (w, C–H), 1659 (vs, C=O), 1605 (m, C=C), 1502 (s, C=C), 1452 (s), 1378 (s), 1256 (vs, C–O), 1124 (s, C–O), 958 (s), 764 (s, C–H), 727 (s, C–H), 710 (s, C–H).

*3.1.12. 3-(3,3-Diethoxypropyl)-3,4-dihydropyrido[2,3-d]pyrimidin-2(1H)-one (**SI-8t**)*

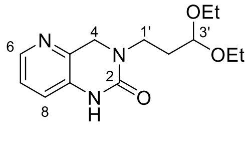

Following GP 1 starting from 2-amino-picolinaldehyde (1.20 g, 4.74 mmol), compound **SI-8t** was obtained as a colorless solid (980 mg, 3.52 mmol, 74%).

***R*f:** 0.15 (CH_2Cl_2/ac = 9/1) [UV, $KMnO_4$].

^{1}H NMR (500 MHz, $CDCl_3$): δ [ppm] = 1.20 (t, ^3J = 7.0 Hz, 6H, CH_3), 1.96 – 2.02 (m, 2H, H-2'), 3.48 – 3.57 (m, 4H, H-1', OCH_2^{a}), 3.67 (dq, ^2J = 9.3 Hz, ^3J = 7.0 Hz, 2H, OCH_2^{b}), 4.59 (s, 2H, H-4), 4.61 (t, ^3J = 5.7 Hz, 1H, H-3'), 6.99 (dd, ^3J = 8.1 Hz, ^4J = 1.4 Hz, 1H, H-8), 7.09 (dd, ^3J = 8.1 Hz, ^3J = 4.8 Hz, 1H, H-7), 7.83 (bs, 1H, NH), 8.14 (dd, ^3J = 4.8 Hz, ^4J = 1.4 Hz, 1H, H-6).

^{13}C NMR (126 MHz, $CDCl_3$): δ [ppm] = 153.7 (C-2), 142.3 (C-6), 138.7 (C-4a), 133.3 (C-8a), 123.2 (C-7), 120.3 (C-8), 101.2 (C-3'), 61.5 (OCH_2), 51.3 (C-4), 43.8 (C-1'), 31.2 (C-2'), 15. (CH_3).

HRMS (+ESI): calc. for $C_{14}H_{21}N_3NaO_3$ $[M+Na]^+$: 302.1475; found: 302.1473.

IR (ATR): $\tilde{\nu}$ [cm^{-1}] = 3341 (w, N–H), 2971 (m, C–H), 2930 (m, C–H), 2898 (m, C–H), 1679 (s, C=O), 1610 (m, C=C), 1598 (m, C=C), 1516 (s, C=C), 1459 (s), 1381 (m), 1253 (m), 1118 (s, C–O), 1024 (s), 885 (m), 795 (vs), 757 (s, C–H), 744 (m, C–H), 730 (s, C–H), 719 (s).

m.p. = 125-127 °C.

*3.1.13. 3-(3-Hydroxy-2,2-dimethylpropyl)-3,4-dihydroquinazolin-2(1H)-one (**SI-8u**)*

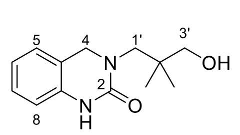

Following GP 1 starting from 2-aminobenzaldehyde (1.21 g, 10.0 mmol), compound **SI-8u** was obtained as a colorless solid (350 mg, 1.49 mmol, 15%).

***R*f:** 0.12 (CH_2Cl_2/ac = 9/1) [UV, $KMnO_4$].

^{1}H NMR (500 MHz, $CDCl_3$): δ [ppm] = 0.98 [s, 6H, $(CH_3)_2$], 3.23 (s, 2H, H-3'), 3.29 (s, 2H, H-1'), 4.54 (s, 2H, H-4), 6.76 (dd, ^3J = 7.6 Hz, ^4J = 1.1 Hz, 1H, H-8) 7.00 (*virt.* td, $^3J \approx {}^3J$ = 7.6 Hz, ^4J = 1.1 Hz, 1H, H-6),, 7.06 (d, ^3J = 7.6 Hz, 1H, H-5), 7.23 (*virt.* td, $^3J \approx {}^3J$ = 7.6 Hz, ^4J = 1.5 Hz, 1H, H-7), 7.37 (bs, 1H, NH).

^{13}C NMR (126 MHz, $CDCl_3$): δ [ppm] = 156.7 (C-2), 136.8 (C-8a), 128.7 (C-7), 125.5 (C-5), 122.6 (C-6), 117.8 (C-4a), 113.8 (C-8), 68.0 (C-3'), 55.1 (C-1'), 52.4 (C-4), 38.3 (C-2'), 23.4 [$(CH_3)_2$].

HRMS (+ESI): calc. for $C_{13}H_{19}N_2O_2$ $[M+H]^+$: 235.1441; found: 235.1442.

IR (ATR): $\tilde{\nu}$ [cm^{-1}] = 3382 (w. N–H), 3197 (w, O–H), 2964 (w, C–H), 2949 (w, C–H), 2914 (w, C–H), 2869 (w, C–H), 2847 (w, C–H), 1645 (vs, C=O), 1606 (m, C=C), 1523 (w, C=C), 1508 (w, C=C), 1476 (w), 1363 (w), 1242 (w), 1051 (s), 863 (w), 782 (w, C–H), 759 (m, C–H), 744 (vs, C–H), 711 (m).

m.p. = 189-191 °C.

*3.1.14. 3-(3,3-Diethoxypropyl)-3,4-dihydropyrido[2,3-d]pyrimidin-2(1H)-one (**SI-8v**)*

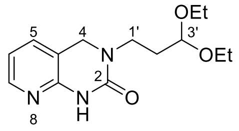

F ollowing GP 1 starting from 2-amino-nicotinaldehyde (611 mg, 5.00 mmol), compound **SI-8v** was obtained as a colorless solid (529 mg, 1.89 mmol, 37%).

***R*f:** 0.03 (CH_2Cl_2/ac = 9/1) [UV, $KMnO_4$].

^{1}H NMR (500 MHz, $CDCl_3$): δ [ppm] = 1.20 (t, ^3J = 7.1 Hz, 6H, CH_3), 1.93 – 1.99 (m, 2H, H-2’), 3.47 – 3.55 (m, 4H, H-1’, OCH_2^a), 3.67 (dq, ^2J = 9.3 Hz, ^3J = 7.1 Hz, 2H, OCH_2^b), 4.47 (s, 2H, H-4), 4.59 (t, ^3J = 5.6 Hz, 1H, H-3’), 6.88 (dd, ^3J = 7.4 Hz, ^3J = 5.0 Hz, 1H, H-6), 7.35 (dd, ^3J = 7.4 Hz, ^4J = 1.7 Hz, 1H, H-5), 7.41 (bs, 1H, NH), 8.15 (dd, ^3J = 5.0 Hz, ^4J = 1.7 Hz, 1H, H-7).

^{13}C NMR (126 MHz, $CDCl_3$): δ [ppm] = 153.5 (C-2), 150.1 (C-8a), 147.6 (C-7), 133.8 (C-5), 117.9 (C-6), 113.0 (C-4a), 101.4 (C-3’), 61.8 (OCH_2), 48.0 (C-4), 43.5 (C-1’), 31.4 (C-2’), 15.5 (CH_3).

HRMS (+ESI): calc. for $C_{14}H_{21}N_3NaO_3$ [M+Na]$^+$: 302.1475; found: 302.1478.

IR (ATR): $\tilde{\nu}$ [cm^{-1}] = 3201 (w, N–H), 2976 (w, C–H), 2931 (w, C–H), 2899 (w, C–H), 2881 (w, C–H), 2855 (w, C–H), 2808 (w, C–H), 1672 (s, C=O), 1608 (s, C=N), 1497 (s, C=C), 1455 (s, C=C), 1440 (s, C=C), 1428 (s), 1246 (m, C–O), 1110 (s, C–O), 892 (m), 779 (s, C–H), 749 (s, C–H), 726 (m).

m.p. = 109-111 °C.

*3.1.15. 2-(2-Oxo-1,4-dihydroquinazolin-3(2H)-yl)benzonitrile (**SI-8w**)*

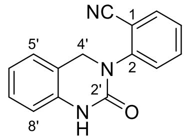

Following the carbonylation protocol of GP 1 starting from **SI-12w** (670 mg, 3.00 mmol), compound **SI-8w** was obtained as a pale-yellow solid (278 mg, 1.11 mmol, 37%).

***R*f:** 0.56 (CH_2Cl_2/ac = 4/1) [UV, $KMnO_4$].

^{1}H NMR (500 MHz, $CDCl_3$): δ [ppm] = 4.88 (s, 2H, H-4’), 6.77 (dd, ^3J = 7.7 Hz, ^4J = 1.2 Hz, 1H, H-8’), 7.01 (*virt.* td, $^3J \approx {}^3J$ = 7.7 Hz, ^4J = 1.2 Hz, 1H, H-6’), 7.09 (dd, ^3J = 7.7 Hz, ^4J = 1.5 Hz, 1H, H-5’), 7.13 (bs, 1H, NH), 7.23* (*virt.* td, $^3J \approx {}^3J$ = 7.7 Hz, ^4J = 1.4 Hz, 1H, H-7’), 7.42 (*virt.* td, $^3J \approx {}^3J$ = 7.8 Hz, ^4J = 1.2 Hz, 1H, H-5), 7.50 (dd, ^3J = 7.8 Hz, ^4J = 1.2 Hz, 1H, H-3), 7.68 (*virt.* td, $^3J \approx {}^3J$ = 7.8 Hz, ^4J = 1.6 Hz, 1H, H-4), 7.74 (dd, ^3J = 7.8 Hz, ^3J = 1.6 Hz, 1H, H-6).

*overlaps with residual solvent signal

^{13}C NMR (126 MHz, $CDCl_3$): δ [ppm] = 153.4 (C-2’), 144.8 (C-2), 136.4 (C-8a’), 134.1 (C-4), 133.9 (C-6), 128.8 (C-7’), 127.9 (C-3), 127.8 (C-5), 125.6 (C-5’), 122.8 (C-6’), 117.8 (C-4a’), 116.7 (CN), 114.4 (C-8’), 112.5 (C-1), 52.0 (C-4’).

HRMS (+ESI): calc. for $C_{15}H_{12}N_3O$ $[M+H]^+$: 250.0975; found: 250.0975.

IR (ATR): $\tilde{\nu}$ [cm^{-1}] = 3205 (w, NH), 2960 (w, C–H), 2919 (w, C–H), 2873 (w, C–H), 2231 (w, C≡N), 1667 (vs, C=O), 1600 (m, C=C), 1575 (w, C=C), 1463 (s), 1297 (m), 1195 (w), 1008 (w), 798 (w), 775 (s, C–H), 746 (vs, C–H), 733 (m), 706 (w).

m.p. = >230 °C.

*3.1.16. 3-(4,4-Diethoxybutyl)-3,4-dihydroquinazolin-2(1H)-one (**SI-8aa**)*

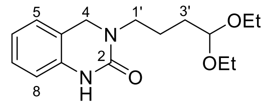

Following GP 1 starting from 2-aminobenzaldehyde (2.13 g, 8.00 mmol), compound **SI-8aa** was obtained as a colorless solid (1.79 g, 6.11 mmol, 76%).

***R*f:** 0.45 (Hex/EtOAc = 2/3) [UV, $KMnO_4$].

^{1}H NMR (500 MHz, $CDCl_3$): δ [ppm] = 1.20 (t, ^3J = 7.1 Hz, 6H, CH_3), 1.64 – 1.75 (m, 4H, H-2’, H-3’), 3.44 – 3.53 (m, 4H, H-1’, $OCH_2{}^a$), 3.64 (dq, ^2J = 9.5 Hz, ^3J = 7.1 Hz, 2H, $OCH_2{}^b$), 4.45 (s, 2H, H-4), 4.52 (t, ^3J = 5.3 Hz, 1H, H-4’), 6.65 – 6.69 (m, 1H, H-8), 6.93 (*virt.* td, $^3J \approx {}^3J$ = 7.5 Hz, ^4J = 1.1 Hz, 1H, H-6), 7.03 (d, ^3J = 7.5 Hz, 1H, H-5), 7.08 – 7.18 (m, 2H, H-7, NH).

^{13}C NMR (126 MHz, $CDCl_3$): δ [ppm] = 154.2 (C-2), 137.0 (C-8a), 128.4 (C-7), 125.7 (C-5), 122.1 (C-6), 117.8 (C-4a), 113.5 (C-8), 102.8 (C-4’), 61.5 (OCH_2), 48.7 (C-4), 47.0 (C-1’), 31.0 (C-2’), 22.3 (C-3’), 15.5 (CH_3).

HRMS (+ESI): calc. for $C_{16}H_{24}N_2NaO_3$ $[M+Na]^+$: 315.1679; found: 315.1677.

IR (ATR): $\tilde{\nu}$ [cm^{-1}] = 3204 (w, N–H), 2971 (w, C–H), 2929 (w, C–H), 2869 (w, C–H), 1660 (vs, C=O), 1608 (s, C=C), 1522 (w, C=C), 1512 (w, C=C), 1457 (s), 1341 (s), 1217 (s), 1116 (s, C–O), 1000 (m), 843 (m), 793 (m), 753 (vs, C–H), 726 (m, C–H), 714 (m).

m.p. = 77-79 °C.

*3.1.17. 3-(2-(1,3-Dioxolan-2-yl)benzyl)-3,4-dihydroquinazolin-2(1H)-one (**SI-8ab**)*

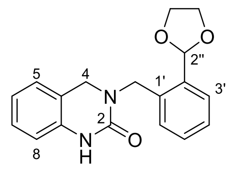

Following GP 1 starting from 2-aminobenzaldehyde (678 mg, 5.60 mmol) and **SI-10** (1.10 g, 6.16 mmol, 1.10 equiv.), compound **SI-8ab** was only obtained as a crude product (1.32 g), which was used in the next step without further purification.

*3.1.18. 3-(4,4-Dimethoxy-2,2-dimethylbutyl)-3,4-dihydroquinazolin-2(1H)-one (**SI-8ac**)*

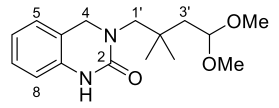

Following GP 1 starting from 2-aminobenzaldehyde (1.00 g, 8.25 mmol) and 4,4-dimethoxy-2,2-dimethylbutan-1-amine[12] (1.54 mL, 1.40 g, 8.67 mmol), compound **SI-8ac** was obtained as a colorless solid (1.54 g, 5.26 mmol, 64%).

***R*f:** 0.25 (CH_2Cl_2/ac = 9/1) [UV, $KMnO_4$].

^{1}H NMR (500 MHz, $CDCl_3$): δ [ppm] = 1.00 [s, 6H, $(CH_3)_2$], 1.62 (d, ^3J = 5.2 Hz, 2H, H-3'), 3.30 (s, 2H, H-1'), 3.31 [s, 6H, $(OCH_3)_2$], 4.45 (s, 2H, H-4), 4.54 (t, ^3J = 5.2 Hz, 1H, H-4'), 6.69 (d, ^3J = 7.8 Hz, ^4J = 1.1 Hz, 1H, H-8), 6.93 (*virt.* td, $^3J \approx {}^3J$ = 7.5 Hz, ^4J = 1.1 Hz, H-6), 7.04 (d, ^3J = 7.5 Hz, 1H, H-5), 7.14 – 7.21 (m, 2H, H-7, NH).

^{13}C NMR (126 MHz, $CDCl_3$): δ [ppm] = 156.2 (C-2), 137.7 (C-8a), 128.4 (C-7), 125.4 (C-5), 122.0 (C-6), 118.8 (C-4a), 113.4 (C-8), 102.6 (C-4'), 57.9 (C-1'), 52.5 [$(OCH_3)_2$], 51.8 (C-4), 42.4 (C-3'), 35.5 (C-2'), 26.0 [$(CH_3)_2$].

HRMS (+ESI): calc. for $C_{16}H_{24}N_2NaO_3$ $[M+Na]^+$: 315.1679; found: 315.1677.

IR (ATR): $\tilde{\nu}$ [cm^{-1}] = 3200 (w, N–H), 2957 (m, C–H), 2934 (m, C–H), 2916 (m, C–H), 2830 (w, C–H), 1662 (s, C=O), 1606 (m, C=C), 1506 (m, C=C), 1459 (m), 1226 (m, C–O), 1119 (s, C–O), 1006 (w), 860 (m), 768 (m, C–H), 746 (vs, C–H), 728 (s).

m.p. = 85-87 °C.

*3.1.19. 3-(2-(1,3-Dioxolan-2-yl)phenethyl)-3,4-dihydroquinazolin-2(1H)-one (**SI-8ba**)*

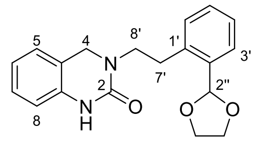

Following GP 1 starting from 2-aminobenzaldehyde (527 mg, 4.35 mmol) and 2-(2-(1,3-dioxolan-2-yl)phenyl)ethan-1-amine[1] (883 mg, 4.57 mmol), compound **SI-8ba** was obtained as a pale-yellow solid (426 mg, 1.31 mmol, 29%).

***R*f:** 0.24 (CH_2Cl_2/ac = 9/1) [UV, $KMnO_4$].

^{1}H NMR (500 MHz, $CDCl_3$): δ [ppm] = 3.08 – 3.11 (m, 2H, H-7'), 3.65 – 3.68 (m, 2H, H-8'), 4.03 – 4.10 (m 2H, H^a-4'', H^a-5''), 4.13 – 4.20 (m, 2H, H^b-4'', H^b-5''), 4.33 (s, 2H, H-4), 6.08 (2, 1H, H-2''), 6.66 (dd, ^3J = 7.6 Hz, ^4J = 1.0 Hz, 1H, H-8), 6.89 (bs, 1H, NH), 6.91 (*virt.* td, $^3J \approx {}^3J$ = 7.6 Hz, ^4J = 1.0 Hz, 1H, H-6), 6.95 (dd, ^3J = 7.6 Hz, ^4J = 1.6 Hz, 1H, H-5), 7.15 (*virt.* td, $^3J \approx {}^3J$ = 7.6 Hz, ^4J = 1.6 Hz, 1H, H-7), 7.24 – 7.28* (m, 3H, H-4', H-5', H-6'), 7.57 – 7.59 (m, 1H, H-3').

*overlaps with residual solvent signal

^{13}C NMR (126 MHz, $CDCl_3$): δ [ppm] = 154.2 (C-2), 137.9 (C-1'), 137.1 (C-8a), 135.5 (C-2'), 130.7(C-4'†), 129.4 (C-5'), 128.3 (C-7), 126.8 (C-3'), 126.7 (C-6'†), 125.6 (C-5), 122.0 (C-6), 117.9 (C-4a), 113.5 (C-8), 102.0 (C-2''), 65.4 (C-4'', C-5''), 49.7 (C-4), 49.6 (C-8'), 30.5 (C-7').

† assignment is interconvertible

HRMS (+ESI): calc. for $C_{19}H_{21}N_2O_3$ $[M+H]^+$: 315.1547; found: 315.1544.

IR (ATR): $\tilde{\nu}$ [cm^{-1}] = 3203 (m, N–H), 2984 (m, C–H), 2887 (m, C–H), 1664 (s, C=O), 1606 (s, C=C), 1514 (s, C=C), 1498 (s, C=C), 1453 (s), 1300 (s), 1226 (s, C–O), 1112 (s, C–O), 942 (s), 812 (s), 754 (vs, C–H), 733 (s, C–H), 719 (s), 711 (s).

m.p. = 147-149 °C.

*3.1.20. 3-(2-(Benzo[d][1,3]dioxol-5-yl)ethyl)-3,4-dihydroquinazolin-2(1H)-one (**SI-8bb**)*

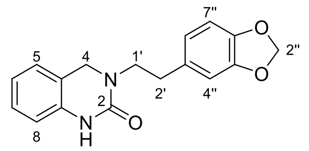

Following GP 1 starting from 2-aminobenzaldehyde (424 mg, 3.50 mmol) and homopiperonylamine[1] (607 mg, 3.68 mmol), compound **SI-8bb** was obtained as a colorless solid (760 mg, 2.57 mmol, 72%).

***R*f:** 0.25 (CH_2Cl_2/ac = 9/1) [UV, $KMnO_4$].

^{1}H NMR (500 MHz, $CDCl_3$): δ [ppm] = 2.84 – 2.89 (m, 2H, H-2’), 3.58 – 3.63 (m, 2H, H-1’), 4.35 (s, 2H, H-4), 5.92 (s, 2H, H-2’’), 6.67 (dd, ^3J = 7.6 Hz, ^4J = 1.1 Hz, 1H, H-8), 6.69 (dd, ^3J = 7.9 Hz, ^4J = 1.6 Hz, 1H, H-6’’), 6.73 (d, ^3J = 7.9 Hz, 1H, H-7“), 6.75 (d, ^4J = 1.6 Hz, 1H, H-4“), 6.89 (bs, 1H, NH), 6.92 (*virt.* td. $^3J \approx {}^3J$ = 7.6 Hz, ^4J = 1.1 Hz, 1H, H-6), 6.97 (d, ^3J = 7.6 Hz, 1H, H-5), 7.16 (*virt.* td, $^3J \approx {}^3J$ = 7.6 Hz, ^4J = 1.5 Hz, 1H, H-7).

^{13}C NMR (126 MHz, $CDCl_3$): δ [ppm] = 154.1 (C-2), 147.8 (C-3a’’), 146.2 (C-7a’’), 137.0 (C-8a), 132.9 (C-5’’), 128.4 (C-7), 125.6 (C-5), 122.1 (C-6), 121.9 (C-6’’), 117.7 (C-4a), 113.6 (C-8), 109.4 (C-4’’), 108.5 (C-7’’), 101.0 (C-2’’), 49.6 (C-1’), 49.6 (C-4), 33.6 (C-2’).

HRMS (+ESI): calc. for $C_{17}H_{17}N_2O_3$ $[M+H]^+$: 297.1234; found: 297.1236.

IR (ATR): $\tilde{\nu}$ [cm^{-1}] = 3204 (w, N–H), 2980 (w, C–H), 2921 (w, C–H), 2881 (w, C–H), 2856 (w, C–H), 1662 (m, C=O), 1604 (m, C=C), 1519 (m, C=C), 1505 (m, C=C), 1441 (m), 1318 (m), 1237 (m, C–O), 1118 (m, C–O), 975 (s), 858 (s), 781 (s, C–H), 751 (s, C–H), 745 (s, C–H), 730 (s), 706 (vs).

m.p. = 183-185 °C.

*3.1.21. 3-(3,4-Dimethoxyphenethyl)-3,4-dihydroquinazolin-2(1H)-one (**SI-8bc**)*

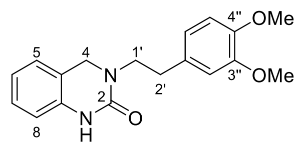

Following GP 1 starting from 2-aminobenzaldehyde (1.21 g, 10.0 mmol), compound **SI-8c** was obtained as a colorless solid (1.90 g, 6.08 mmol, 61%).

***R*f:** 0.24 (CH_2Cl_2/ac = 9/1) [UV, $KMnO_4$].

^{1}H NMR (500 MHz, $CDCl_3$): δ [ppm] = 2.89 (t, ^3J = 8.2 Hz, 2H, H-2’), 3.64 (t, ^3J = 8.2 Hz, 2H, H-1’), 3.77 (s, 3H, C-3’’-OCH_3), 3.85 (s, 3H, C-4’’-OCH_3), 4.31 (s, 2H, H-4), 6.66 (d, ^3J = 8.0 Hz, 1H, H-8), 6.75 (d, ^4J = 1.4 Hz, 1H, H-2’’), 6.77 – 6.81 (m, 2H, H-, H-5’’, H-6’’), 6.86 (bs, 1H, NH), 6.89 – 6.96 (m, 2H, H-5, H-6), 7.15 (ddd, ^3J = 8.0 Hz, ^3J = 6.7 Hz, ^4J = 2.1 Hz, 1H, H-7).

^{13}C NMR (126 MHz, $CDCl_3$): δ [ppm] = 154.1 (C-2), 149.0 (C-3''), 147.7 (C-4''), 137.0 (C-8a), 131.7 (C-1''), 128.4 (C-7), 125.6 (C-5), 122.2 (C-6), 120.8 (C-6''), 117.9 (C-4a), 113.5 (C-8), 112.1 (C-2''), 111.4 (C-5''), 56.0 (C-4''-O*C*H_3), 55.9 (C-3''-O*C*H_3), 49.6 (C-4), 49.6 (C-1'), 33.6 (C-2').

HRMS (+ESI): calc. for $C_{18}H_{21}N_2O_3$ $[M+H]^+$: 313.1547; found: 313.1546.

IR (ATR): $\tilde{\nu}$ [cm^{-1}] = 3204 (w, N–H), 2985 (w, C–H), 2958 (w, C–H), 2925 (w, C–H), 2859 (w, C–H), 2841 (w, C–H), 1659 (m, C=O), 1606 (m, C=C), 1591 (m, C=C), 1513 (m, C=C), 1500 (m, C=C), 1467 (m), 1336 (m), 1258 (m, C–O), 1245 (m, C–O), 1107 (m C–O), 925 (s), 804 (s), 771 (s, C–H), 753 (vs, C–H), 712 (vs).

m.p. = 148-150 °C.

*3.1.22. 3-(2,2-Diethoxyethyl)-3,4-dihydroquinazolin-2(1H)-one (**SI-8ca**)*

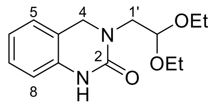

Following GP 1 starting from 2-aminobenzaldehyde (6.31 g, 25.0 mmol), compound **SI-8ca** was obtained as a colorless solid (4.78 g, 17.2 mmol, 69%).

***R*f:** 0.51 (Hex/EtOAc = 2/3) [UV, $KMnO_4$].

^{1}H NMR (500 MHz, $CDCl_3$): δ [ppm] = 1.21 (t, ^3J = 7.0 Hz, 6H, CH_3), 3.50 (d, ^3J = 5.5 Hz, 2H, H-1'), 3.57 (dq, ^2J = 9.4 Hz, ^3J = 7.0 Hz, 2H, $OCH_2{}^a$), 3.76 (dq, ^2J = 9.3 Hz, ^3J = 7.1 Hz, 2H, $OCH_2{}^b$), 4.60 (s, 2H, H-4), 4.73 (t, ^3J = 5.6 Hz, 1H, H-2'), 6.67 (dd, ^3J = 7.6 Hz, 1H, H-8), 6.93 (*virt.* td, $^3J \approx {}^3J$ = 7.6 Hz, ^4J = 1.2 Hz, 1H, H-6), 7.02 (d, ^3J = 7.6 Hz, ^4J = 1.5 Hz, 1H, H-5), 7.13 – 7.17 (*m*, 1H, NH, H-7).

^{13}C NMR (126 MHz, $CDCl_3$): δ [ppm] = 154.7 (C-2), 137.0 (C-8a), 128.2 (C-7), 125.5 (C-5), 122.1 (C-6), 118.3 (C-4a), 113.6 (C-8), 102.1 (C-2'), 63.8 (OCH_2), 50.9 (C-4†), 50.8 (C-1'†), 15.6 (CH_3).

† assignment is interconvertible

154.2 (C-2), 137.0 (C-8a), 128.3 (C-7), 125.7 (C-5), 122.1 (C-6), 117.9 (C-4a), 113.5 (C-8), 101.4 (C-3'), 61.7 (OCH_2), 49.1 (C-4), 43.7 (C-1'), 31.5 (C-2'), 15.5 (CH_3).

HRMS (+ESI): calc. for $C_{14}H_{20}N_2NaO_3$ $[M+Na]^+$: 287.1366; found: 287.1363.

IR (ATR): $\tilde{\nu}$ [cm^{-1}] = 3202 (w, NH), 2975 (w, C–H), 2921 (w, C–H), 2877 (w, C–H), 1662 (vs, C=O), 1605 (m, C=C), 1519 (w, C=C), 1457 (m), 1292 (s), 1110 (s, C–O), 895 (w), 763 (s, C–H), 746 (vs, C–H), 727 (m, C–H), 701 (s).

m.p. = 109-111 °C.

3.2. General Procedure 2 (*GP 2*): Cross-Coupling of Quinazolin-2-one Bromides

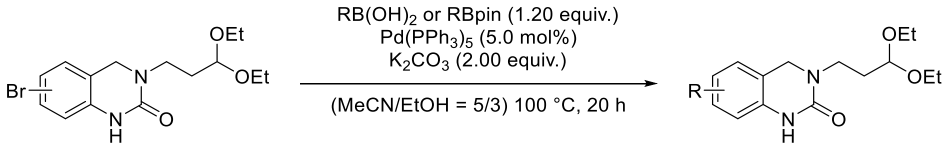

According to a modified procedure by *Cortez et al.*,[13] the corresponding boronic acid or its pinacol ester (1.20 equiv.), K_2CO_3 (2.00 equiv.) and $Pd(PPh_3)_4$ (5.0 mol%) was added to a solution of bromide (1.00 equiv.) in MeCN/EtOH (5/3, 200 mM). The reaction mixture was stirred at 100 °C for 20 h and cooled to room temperature. The mixture was diluted with EtOAc (100 mL) and quenched by addition of sat. NH_4Cl solution (100 mL). The organic layer was washed with brine (100 mL), dried over Na_2SO_4 and filtered, and the solvent was removed under reduced pressure. The crude products were subjected to automated flash column chromatography (CH_2Cl_2/MeOH = 49/1 → 19/1) to yield cross-coupled 3,4-dihydroquinazolinones.

*3.2.1. 3-(3,3-Diethoxypropyl)-6-vinyl-3,4-dihydroquinazolin-2(1H)-one (**SI-8i**)*

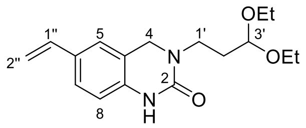

Following GP2 starting from **SI-8c** (1.07 g, 3.00 mmol) and using vinyltrifluoroborate as coupling reagent, compound **SI-8i** was obtained as colorless solid (417 mg, 1.37 mmol, 46%).

***R*f:** 0.36 (CH_2Cl_2/ac = 4/1) [UV, $KMnO_4$].

^{1}H NMR (500 MHz, $CDCl_3$): δ [ppm] = 1.20 (t, ^3J = 7.0 Hz, 6H, CH_3), 1.93 – 1.99 (m, 2H, H-2'), 3.48 – 3.53 (m, 4H, H-1', OCH_2^a), 3.67 (dq, ^2J = 9.4 Hz, ^3J = 7.0 Hz, 2H, OCH_2^b), 4.46 (s, 2H, H-4), 4.59 (t, ^3J = 5.6 Hz, 1H, H-3'), 5.15 (d, ^3J = 10.9 Hz, ^2J = 0.9 Hz, 1H, H-2''$_{trans}$), 5.61 (dd, ^3J = 17.6 Hz, ^2J = 0.9 Hz, 1H, H-2''$_{cis}$), 6.58 – 6.66 (m, 2H, H-8, H-1''), 6.69 (bs, 1H, NH), 7.10 (s, 1H, H-5), 7.21 (dd, ^3J = 8.1 Hz, ^4J = 1.9 Hz, 1H, H-7).

^{13}C NMR (126 MHz, $CDCl_3$): δ [ppm] = 153.9 (C-2), 136.6 (C-8a), 136.0 (C-1''), 131.9 (C-6), 126.4 (C-7), 123.5 (C-5), 118.0 (C-4a), 113.6 (C-8), 112.4 (C-2''), 101.4 (C-3'), 61.7 (OCH_2), 49.0 (C-4), 43.7 (C-1'), 31.4 (C-2'), 15.5 (CH_3).

HRMS (+ESI): calc. for $C_{17}H_{24}N_2NaO_3$ $[M+Na]^+$: 327.1679; found: 327.1676.

IR (ATR): $\tilde{\nu}$ [cm^{-1}] = 3197 (w, N–H), 2973 (w, C–H), 2929 (w, C–H), 2895 (w, C–H), 1670 (vs, C=O), 1615 (s, C=C), 1603 (vs, C=C), 1508 (vs, C=C), 1451 (s), 1255 (m, C–O), 1125 (s, C–O), 827 (vs), 788 (s, C–H), 754 (s, C–H), 739 (s, C–H).

m.p. = 142-144 °C.

*3.2.2. 6-Allyl-3-(3,3-diethoxypropyl)-3,4-dihydroquinazolin-2(1H)-one (**SI-8j**)*

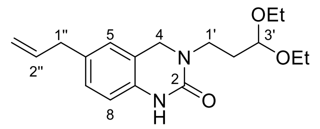

Following GP2 starting from **SI-8c** (1.00 g, 2.80 mmol) and using allyltributylstannane as coupling reagent, compound **SI-8j** was obtained as colorless solid (544 mg, 1.71 mmol, 61%).

***R*f:** 0.37 (CH_2Cl_2/ac = 4/1) [UV, $KMnO_4$].

^{1}H NMR (500 MHz, $CDCl_3$): δ [ppm] = 1.20 (t, ^3J = 7.0 Hz, 6H, CH_3), 1.92 – 1.99 (m, 2H, H-2'), 3.30 (*virt.* dt, ^3J = 6.7 Hz, $^4J \approx {}^4J$ = 1.5 Hz, 2H, H-1''), 3.47 – 3.53 (m, 4H, H-1', OCH_2^a), 3.67 (dq, ^2J = 9.4 Hz, ^3J = 7.0 Hz, 2H, OCH_2^b), 4.43 (s, 2H, H-4), 4.59 (t, ^3J = 5.6 Hz, 1H, H-3'), 5.03 – 5.09 (m, 2H, H-3''), 5.92 (ddt, ^3J = 17.5 Hz, ^3J = 9.5 Hz, ^3J = 6.7 Hz, 1H, H-2''), 6.57 (d, ^3J = 8.0 Hz, 1H, H-8), 6.66 (bs, 1H, NH), 6.86 (s, 1H, H-5), 6.97 (dd, ^3J = 8.0 Hz, ^4J = 1.9 Hz, 1H, H-7).

^{13}C NMR (126 MHz, $CDCl_3$):* δ [ppm] = 154.5 (C-2), 137.6 (C-2'''), 135.1 (C-8a), 133.9 (C-6), 128.5 (C-7), 125.7 (C-5), 117.8 (C-4a), 116.0 (C-3'''), 113.6 (C-8), 101.4 (C-3'), 61.7 (OCH_2), 49.1 (C-4), 43.7 (C-1'), 39.6 (C-1'''), 31.4 (C-2'), 15.5 (CH_3).

*compound is prone to hydrolysis in solution and contains the aldehyde as impurity

HRMS (+ESI): calc. for $C_{18}H_{16}N_2NaO_3$ $[M+Na]^+$: 341.1836; found: 341.1833.

IR (ATR): $\tilde{\nu}$ [cm^{-1}] = 3208 (w, N–H), 2976 (w, C–H), 2930 (w, C–H), 2882 (w, C–H), 1660 (vs, C=O), 1637 (s, C=C), 1609 (s, C=C), 1510 (m, C=C), 1475 (m), 1276 (m), 1108 (s, C–O), 821 (s), 760 (s, C–H), 730 (m, C–H).

m.p. = 71-73 °C.

*3.2.3. 3-(3,3-Diethoxypropyl)-7-(furan-3-yl)-3,4-dihydroquinazolin-2(1H)-one (**SI-8n**)*

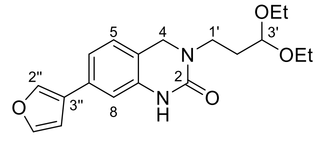

Following GP2 starting from **SI-8d** (500 mg, 1.40 mmol) and using furan-3-ylboronic acid pinacol ester as coupling reagent, compound **SI-8n** was obtained as colorless solid (402 mg, 1.17 mmol, 83%).

***R*f:** 0.43 (CH_2Cl_2/ac = 4/1) [UV, $KMnO_4$].

^{1}H NMR (500 MHz, $CDCl_3$):* δ [ppm] = 1.17 (t, ^3J = 7.0 Hz, 6H, CH_3), 1.96 – 2.00 (m, 2H, H-2'), 3.49 – 3.55 (m, 4H, H-1', OCH_2^a), 3.67 (dq, ^2J = 9.3 Hz, ^3J = 7.0 Hz, 2H, OCH_2^b), 4.47 (s, 2H, H-4), 4.60 (t, ^3J = 5.6 Hz, 1H, H-3'), 6.65 (dd, ^3J = 1.7 Hz, ^4J = 0.9 Hz, 1H, H-4''), 6.76 (d, ^4J = 1.6 Hz, 1H, H-8), 7.03 (d, ^3J = 7.8 Hz, 1H, H-5), 7.06 (dd, ^3J = 7.8 Hz, ^4J = 1.6 Hz, 1H, H-6), 7.13 (bs, 1H, NH), 7.47 (*virt.* t, $^3J \approx {}^4J$ = 1.7 Hz, 1H, H-5''), 7.70 (dd, ^4J = 1.7 Hz, ^4J = 0.9 Hz, 1H, H-2'').

^{13}C NMR (126 MHz, $CDCl_3$):* δ [ppm] = 154.4 (C-2), 143.9 (C-5''), 138.8 (C-2''), 137.4 (C-8a), 132.8 (C-7), 126.1 (C-5), 125.9 (C-3''), 119.7 (C-6), 116.5 (C-4a), 110.8 (C-8), 108.9 (C-4''), 101.4 (C-3'), 61.7 (OCH_2), 48.9 (C-4'), 43.8 (C-1'), 31.4 (C-2'), 15.5 (CH_3).

*compound is prone to hydrolysis in solution and contains the aldehyde as impurity

HRMS (+ESI): calc. for $C_{19}H_{24}N_2NaO_4$ $[M+Na]^+$: 367.1628; found: 367.1627.

IR (ATR): $\tilde{\nu}$ [cm^{-1}] = 3211 (w, N–H), 2976 (m, C–H), 2932 (m, C–H), 2898 (m, C–H), 2877 (m, C–H), 1661 (s, C=O), 1628 (m, C=C), 1598 (m, C=C), 1505 (s, C=C), 1445 (s), 1240 (m, C–O), 1106 (m, C–O), 872 (m), 778 (s, C–H), 759 (s, C–H), 725 (m), 703 (m).

m.p. = 128-130 °C.

*3.2.4. 3-(3,3-Diethoxypropyl)-7-(thiophen-3-yl)-3,4-dihydroquinazolin-2(1H)-one (**SI-8o**)*

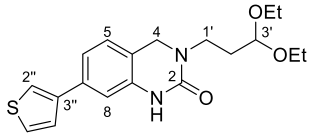

Following GP2 starting from **SI-8d** (500 mg, 1.40 mmol) and using thiophen-3-ylboronic acid as coupling reagent, compound **SI-8o** was obtained as colorless solid (446 mg, 1.23 mmol, 88%).

***R*f:** 0.49 (CH_2Cl_2/ac = 4/1) [UV, $KMnO_4$].

^{1}H NMR (400 MHz, $CDCl_3$):* δ [ppm] = 1.17 (t, ^3J = 7.0 Hz, 6H, CH_3), 1.90 – 1.95 (m, 2H, H-2’), 3.45 – 3.53 (m, 4H, H-1’, OCH_2^a), 3.67 (dq, ^2J = 9.4 Hz, ^3J = 7.0 Hz, 2H, OCH_2^b), 4.47 (s, 2H, H-4), 4.57 (t, ^3J = 5.6 Hz, 1H, H-3’), 6.91 (d, ^4J = 1.7 Hz, 1H, H-8), 7.09 (d, ^3J = 7.8 Hz, 1H, H-5), 7.18 (bs, 1H, NH), 7.19 (dd, ^3J = 7.8 Hz, ^4J = 1.7 Hz, 1H, H-6), 7.37 (dd, ^3J = 5.0 Hz, ^4J = 1.4 Hz, 1H, H-4’’), 7.41 (dd, ^3J = 5.0 Hz, ^4J = 2.9 Hz, 1H, H-5’’), 7.47 (dd, ^4J = 2.9 Hz, ^4J = 1.4 Hz, 1H, H-2’’).

^{13}C NMR (101 MHz, CD_2Cl_2):* δ [ppm] = 154.3 (C-2), 141.9 (C-3’’), 138.1 (C-8a), 136.2 (C-7), 126.8 (C-5’’), 126.5 (C-5†), 126.4 (C-4’’†), 121.0 (C-2’’), 120.3 (C-6), 117.3 (C-4a), 111.4 (C-8), 101.7 (C-3’), 61.8 (OCH_2), 49.1 (C-4), 43.9 (C-1’), 31.8 (C-2’), 15.6 (CH_3).

*compound is prone to hydrolysis in solution and contains the aldehyde as impurity

† assignment is interconvertible

HRMS (+ESI): calc. for $C_{14}H_{21}N_3NaO_3S$ $[M+Na]^+$: 383.1400; found: 383.1399.

IR (ATR): $\tilde{\nu}$ [cm^{-1}] = 3196 (w, N–H), 2974 (w, C–H), 2929 (w, C–H), 2894 (w, C–H), 2875 (w, C–H), 1661 (vs, C=O), 1626 (s, C=C), 1601 (s, C=C), 1548 (w, C=C), 1516 (s, C=C), 1455 (s), 1128 (s, C–O), 1026 (s), 871 (s), 774 (vs, C–H), 750 (vs, C–H), 706 (s).

m.p. = 144-146 °C.

*3.2.5. tert-Butyl 2-(3-(3,3-diethoxypropyl)-2-oxo-1,2,3,4-tetrahydroquinazolin-7-yl)-1H-pyrrole-1-carboxylate (**SI-8p**)*

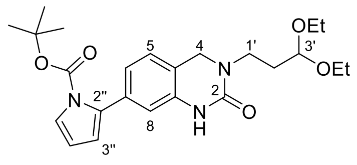

Following GP2 starting from **SI-8d** (500 mg, 1.40 mmol) and using [1-(*tert*-butoxycarbonyl)-1H-pyrrol-2-yl]boronic acid as coupling reagent, compound **SI-8p** was obtained as colorless solid (486 mg, 1.10 mmol, 78%).

***R*f:** 0.28 (CH_2Cl_2/ac = 4/1) [UV, $KMnO_4$].

^{1}H NMR (500 MHz, $CDCl_3$):* δ [ppm] = 1.20 (t, ^3J = 7.0 Hz, 6H, CH_3), 1.42 [s, 9H, $(CH_3)_3$], 1.94 – 1.99 (m, 2H, H-2'), 3.48 – 3.56 (m, 4H, H-1', OCH_2^a), 3.68 (dq, ^2J = 9.3 Hz, ^3J = 7.0 Hz, 2H, OCH_2^b), 4.48 (s, 2H, H-4), 4.59 (t, ^3J = 5.6 Hz, 1H, H-3'), 6.17 (dd. ^3J = 3.3 Hz, ^4J = 1.8 Hz, 1H, H-3''), 6.21 (*virt.* t, $^3J \approx {}^3J$ = 3.3 Hz, 1H, H-4''), 6.55 (bs, 1H, NH), 6.62 (d, ^4J = 1.6 Hz, 1H, H-8), 6.93 (dd, ^3J = 7.8 Hz, ^4J = 1.6 Hz, 1H, H-6), 7.02 (d, ^3J = 7.8 Hz, 1H, H-5), 7.31 (dd, ^3J = 3.3 Hz, ^4J = 1.8 Hz, 1H, H-5'').

^{13}C NMR (126 MHz, CD_2Cl_2): * δ [ppm] = 162.3 (NCOO), 151.4 (C-2), 149.2, 141.3, 138.3 (C-8a), 133.4, 127.5, 124.8, 124.3, 116.3, 114.7, 111.2, 101.5 (C-3'), 84.7 [*C*$(CH_3)_3$], 61.3 (OCH_2), 49.1 (C-4), 43.8 (C-1'), 32.0 (C-2'), 27.8 [C(*C*$H_3)_3$], 15.5 (CH_3).

*compound is prone to hydrolysis in solution and contains the aldehyde as impurity

HRMS (+ESI): calc. for $C_{24}H_{33}N_3NaO_5$ $[M+Na]^+$: 466.2312; found: 466.2312.

IR (ATR): $\tilde{\nu}$ [cm^{-1}] = 3230 (w, N–H), 2976 (m, C–H), 2933 (m, C–H), 2881 (m, C–H), 1734 (s, C=O), 1710 (m), 1659 (s, C=O), 1626 (m, C=C), 1596 (s, C=C), 1523 (m, C=C), 1443 (m), 1310 (s, C–O), 1142 (vs, C–O), 843 (m), 772 (m, C–H), 756 (m, C–H), 729 (s, C–H).

m.p. = 97-99 °C.

*3.2.6. 3-(3,3-Diethoxypropyl)-7-(pyridin-3-yl)-3,4-dihydroquinazolin-2(1H)-one (**SI-8q**)*

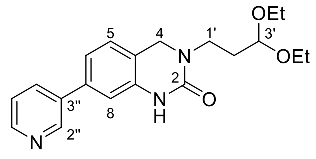

Following GP2 starting from **SI-8d** (500 mg, 1.40 mmol) and using pyridin-3-ylboronic acid as coupling reagent, compound **SI-8q** was obtained as colorless solid (330 mg, 928 µmol, 66%).

***R*f:** 0.16 (CH_2Cl_2/ac = 3/2) [UV, $KMnO_4$].

^{1}H NMR (500 MHz, $CDCl_3$): δ [ppm] = 1.20 (t, ^3J = 7.0 Hz, 6H, CH_3), 1.95 – 2.01 (m, 2H, H-2'), 3.48 – 3.57 (m, 4H, H-1', OCH_2^a), 3.67 (dq, ^2J = 9.3 Hz, ^3J = 7.0 Hz, 2H, OCH_2^b), 4.52 (s, 2H, H-4), 4.60 (t, ^3J = 5.6 Hz, 1H, H-3'), 6.86 (*virt.* t, $^4J \approx {}^5J$ = 1.1Hz, 1H, H-8), 7.13 – 7.16 (m, 2H, H-5, H-6), 7.28 (bs, 1H, NH), 7.36 (ddd, ^3J = 7.9 Hz, ^3J = 4.8 Hz, ^4J = 0.9 Hz, 1H, H-5''), 7.83 (ddd, ^3J = 7.9 Hz, ^4J = 2.4 Hz, ^4J = 1.6 Hz, 1H, H-4''), 8.60 (dd, ^3J = 4.8 Hz, ^4J = 1.6 Hz, 1H, H-6''), 8.81 (dd, ^4J = 2.4 Hz, ^4J = 0.9 Hz, 1H, H-2'').

^{13}C NMR (126 MHz, $CDCl_3$): δ [ppm] = 154.2 (C-2), 148.9 (C-6''), 148.2 (C-2''), 138.2 (C-7), 137.9 (C-8a), 136.1 (C-3''), 134.5 (C-4''), 126.4 (C-5), 123.8 (C-5''), 120.9 (C-6), 117.8 (C-4a), 112.1 (C-8), 101.4 (C-3'), 61.7 (OCH_2), 48.8 (C-4), 43.7 (C-1'), 31.4 (C-2'), 15.5 (CH_3).

HRMS (+ESI): calc. for $C_{20}H_{25}N_3NaO_3$ $[M+Na]^+$: 378.1788; found: 378.1788.

IR (ATR): $\tilde{\nu}$ [cm^{-1}] = 3337 (w, N–H), 2976 (w, C–H), 2938 (w, C–H), 2902 (w, C–H), 2878 (w, C–H), 1668 (vs, C=O), 1626 (w, C=N), 1598 (s, C=C), 1568 (w, C=C), 1534 (w, C=C), 1497 (s, C=C), 1475 (s), 1307 (s), 1112 (s, C–O), 931 (s), 799 (s), 769 (s, C–H), 708 (s).

m.p. = 124-126 °C.

*3.2.7. Ethyl (E)-3-(3-(3,3-diethoxypropyl)-2-oxo-1,2,3,4-tetrahydroquinazolin-7-yl)acrylate (**SI-8r**)*

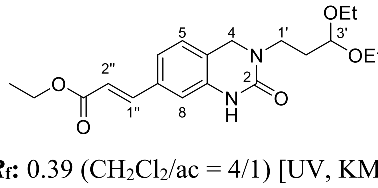

Following GP2 starting from **SI-8d** (1.00 g, 2.80 mmol) and using 2-(ethoxycarbonyl)vinylboronic acid pinacol ester as coupling reagent, compound **SI-8r** was obtained as colorless solid (1.00 g, 2.67 mmol, 95%).

***R*f:** 0.39 (CH_2Cl_2/ac = 4/1) [UV, $KMnO_4$].

^{1}H NMR (500 MHz, $CDCl_3$): δ [ppm] = 1.20 [t, ^3J = 7.1 Hz, 6H, $(CH_3)_2$], 1.33 (t, ^3J = 7.2 Hz, 3H, CH_3), 1.93 – 2.00 (m, 2H, H-2’), 3.47 – 3.56 [m, 4H, H-1’, $(OCH_2{}^a)_2$], 3.67 [dq, ^2J = 9.4 Hz, ^3J = 7.1 Hz, 2H, $(OCH_2{}^b)_2$], 4.26 (q, ^3J = 7.2 Hz, 2H, OCH_2), 4.48 (s, 2H, H-4), 4.59 (t, ^3J = 5.6 Hz, 1H, H-3’), 6.38 (d, ^3J = 16.0 Hz, 1H, H-2’’), 6.79 (d, ^4J = 1.6 Hz, 1H, H-8), 7.05 (d, ^3J = 7.8 Hz, 1H, H-5), 7.08 (bs, 1H, NH), 7.10 (dd, ^3J = 7.8 Hz, ^4J = 1.6 Hz, 1H, H-7), 7.59 (d, ^3J = 16.0 Hz. 1H, H-1’’).

^{13}C NMR (126 MHz, $CDCl_3$): δ [ppm] = 167.0 (COO), 154.1 (C-2), 143.8 (C-1’’), 137.6 (C-8a), 134.8 (C-7), 126.2 (C-5), 122.0 (C-6), 120.0 (C-4a), 118.9 (C-2"), 112.6 (C-8), 101.4 (C-3’), 61.7 (OCH_2), , 60.8 ($COOCH_2$), 48.9 (C-4), 43.7 (C-1’), 31.4 (C-2’), 15.5 (CH_3), 14.5 ($COOCH_2CH_3$).

HRMS (+ESI): calc. for $C_{20}H_{28}N_2NaO_5$ $[M+Na]^+$: 399.1890; found: 399.1888.

IR (ATR): $\tilde{\nu}$ [cm^{-1}] = 3213 (w, N–H), 2976 (w, C–H), 2931 (w, C–H), 2899 (w, C–H), 1710 (s, C=O), 1660 (vs, C=O), 1641 (vs, C=C), 1626 (s, C=C), 1593 (m, C=C), 1562 (w, C=C), 1528 (w, C=C), 1368 (m), 1216 (w), 1124 (s, C–O), 856 (m), 778 (m, C–H), 755 (m, C–H), 747 (m, C–H), 727 (m, C–H).

m.p. = 106-108 °C.

*3.2.8. 3-(3,3-Diethoxypropyl)-6-(4-methoxyphenyl)-3,4-dihydroquinazolin-2(1H)-one (**SI-8s**)*

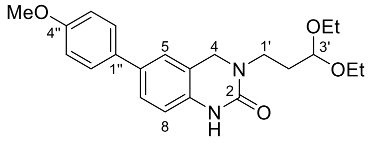

Following GP2 starting from **SI-8c** (1.00 g, 2.80 mmol) and using 4-methoxyphenylboronic acid as coupling reagent, compound **SI-8s** was obtained as brown solid (563 mg, 1.46 mmol, 52%).

***R*f:** 0.51 (CH_2Cl_2/ac = 4/1) [UV, $KMnO_4$].

^{1}H NMR (500 MHz, $CDCl_3$): δ [ppm] = 1.20 (t, ^3J = 7.1 Hz, 6H, CH_3), 1.95 – 2.01 (m, 2H, H-2’), 3.48 – 3.57 (m, 4H, H-1’, $OCH_2{}^a$), 3.68 (dq, ^2J = 9.4 Hz, ^3J = 7.1 Hz, 2H, $OCH_2{}^b$), 3.85 (s, 3H, OCH_3), 4.52 (s, 2H, H-4), 4.61 (t, ^3J = 5.6 Hz, 1H, H-3’), 6.71 (d, ^3J = 8.2 Hz, 1H, H-8), 6.92 (bs, 1H, NH), 6.94 – 6.98 (m, 2H, H-3’’), 7.22 (d, ^4J = 2.0 Hz, 1H, H-5), 7.34 (dd, ^3J = 8.2 Hz, ^4J = 2.0 Hz, 1H, H-7), 7.42 – 7.47 (m, 2H, H-2’’).

^{13}C NMR (126 MHz, $CDCl_3$): δ [ppm] = 159.0 (C-4’’), 154.2 (C-2), 135.8 (C-8a), 135.0 (C-7), 133.1 (C-1’’), 127.8 (C-2’’), 126.7 (C-7), 123.9 (C-5), 118.2 (C-4a), 114.4 (C-3’’), 113.9 (C-8), 101.4 (C-3’), 61.7 (OCH_2), 55.5 (OCH_3), 49.2 (C-4), 43.7 (C-1’), 31.5 (C-2’), 15.5 (CH_3).

HRMS (+ESI): calc. for $C_{22}H_{28}N_2NaO_4$ $[M+Na]^+$: 407.1941; found: 407.1939.

IR (ATR): $\tilde{\nu}$ [cm^{-1}] = 3185 (w, N–H), 2967 (w, C–H), 2917 (w, C–H), 2873 (w, C–H), 2839 (w, C–H), 1655 (vs, C=O), 1608 (s, C=C), 1577 (w, C=C), 1511 (s, C=C), 1454 (s), 1317 (s), 1244 (vs, C–O), 1105 (s, C–O), 1027 (s), 834 (m), 797 (s), 789 (s, C–H), 755 (s, C–H), 747 (s, C–H), 727 (m), 711 (m).

m.p. = 139-141 °C.

3.3. General Procedure 3 (*GP 3*): Synthesis of Hydrazones by Deprotection of Acetals

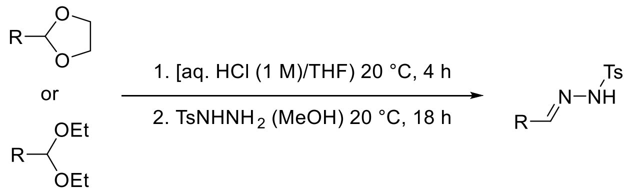

According to a modified procedure by *Buchelt et al.*,[1] The corresponding acetal (1.00 equiv.) was dissolved in THF (500 mM) and aq. HCl (1 M, 500 mM) and stirred at 20 °C for 4 h. Afterwards, the reaction mixture was quenched by addition of sat. $NaHCO_3$ solution (pH = 7) and the aqueous layer was extracted with CH_2Cl_2 (3 × 20 mL). The combined organic layers were dried over Na_2SO_4, filtered, and the solvent was removed under reduced pressure to obtain the crude aldehydes.

The crude aldehydes were redissolved in MeOH (1 M) and *p*-toluenesulfonyl hydrazide (1.00 equiv.) was added. The reaction mixture was stirred at 20 °C for 18 h. The resulting precipitate was collected and washed with little amounts of cold MeOH and Et_2O to obtain hydrazones as colorless solids.

The hydrazones are typically (*E*)-configured, but varying amounts of the respective (*Z*)-isomer are visible in the NMR spectra.

*3.3.1. N'-(3-(2-Oxo-1,4-dihydroquinazolin-3(2H)-yl)propylidene)-4-methylbenzenesulfonohydrazide (**4a**)*

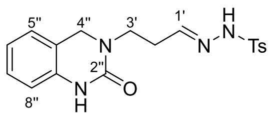

Following GP 3 starting from **SI-8a**, compound **4a** was obtained as a colorless solid (1.05 g, 2.82 mmol, 79%).

***R*f:** 0.28 (CH_2Cl_2/ac = 7/3) [UV, $KMnO_4$].

^{1}H NMR (500 MHz, DMSO-*d*$_6$): δ [ppm] = 2.28 (s, 3H, CH_3), 2.39 (td, ^3J = 6.8 Hz, ^3J = 5.3 Hz, 2h, H-2'), 3.39 (t, ^3J = 6.8 Hz, 2H, H-3'), 4.28 (s, 2H, H-4''), 6.75 (dd, ^3J = 7.6 Hz, ^4J = 1.2 Hz, 1H, H-8''), 6.86 (*virt.* td, $^3J \approx {}^3J$ = 7.6 Hz, ^4J = 1.2 Hz, 1H, H-6''), 7.00 (d, ^3J = 7.6 Hz, 1H, H-5''), 7.12 (*virt.* td, $^3J \approx {}^3J$ = 7.6 Hz, ^4J = 1.5 Hz, 1H, H-7''), 7.25 – 7.30 (m, 3H, H-3, H-1'), 7.62 – 7.66 (m, 2H, H-2), 9.13 (bs, 1H, NH), 11.04 (bs, 1H, NNH).

^{13}C NMR (126 MHz, DMSO-*d*$_6$): δ [ppm] = 153.5 (C-2''), 149.5 (C-1'), 143.2 (C-4), 137.6 (C-8a''), 136.3 (C-1), 129.6 (C-3), 127.8 (C-7''), 127.0 (C-2), 125.5 (C-5''), 121.0 (C-6''), 117.9 (C-4a''), 113.2 (C-8''), 47.8 (C-4''), 43.3 (C-3'), 30.2 (C-2'), 21.0 (CH_3).

HRMS (+ESI): calc. for $C_{18}H_{21}N_4O_3S$ $[M+H]^+$: 373.1329; found: 373.1330.

IR (ATR): $\tilde{\nu}$ [cm^{-1}] = 3358 (w, N–H), 2865 (w, C–H), 2839 (w, C–H), 2822 (w, C–H), 2761 (w, C–H), 1649 (s, C=O), 1604 (m, C=C), 1516 (w, C=C), 1504 (w, C=C), 1476 (s), 1329 (s, RSO_2N), 1165 (s, RSO_2N), 1019 (w), 872 (w), 760 (s, C–H), 749 (vs, C–H), 720 (m, C–H), 706 (m).

m.p. = >216 °C (decomposition).

*3.3.2. N'-(3-(5-Bromo-2-oxo-1,4-dihydroquinazolin-3(2H)-yl)propylidene)-4-methylbenzenesulfonohydrazide (**4b**)*

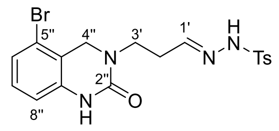

Following GP 3 starting from **SI-8b**, compound **4b** was obtained as a colorless solid (200 mg, 444 µmol, 65%).

***R*f:** 0.42 (CH_2Cl_2/ac = 7/3) [UV, $KMnO_4$].

^{1}H NMR (500 MHz, DMSO-*d*$_6$): δ [ppm] = 2.27 (s, 3H, CH_3), 2.40 (td, ^3J = 6.9 Hz, ^3J = 4.2 Hz, 2H, H-2'), 3.42 (t, ^3J = 6.9 Hz, 2H, H-3'), 4.29 (s, 2H, H-4''), 6.75 (dd, ^3J = 7.9 Hz, ^4J = 1.3 Hz, 1H, H-8''), 7.08 (*virt. t*, $^3J \approx {}^3J$ = 7.9 Hz, 1H, H-7''), 7.12 (dd, ^3J = 7.9 Hz, ^4J = 1.3 Hz, 1H, H-6''), 7.24 – 7.29 (m, 3H, H-3, H-1'), 7.61 – 7.65 (m, 2H, H-2), 9.35 (bs, 1H, NH), 11.07 (bs, 1H, NNH).

^{13}C NMR (126 MHz, DMSO-*d*$_6$): δ [ppm] = 152.5 (C-2''), 149.4 (C-1'), 143.2 (C-4), 139.1 (C-8a''), 136.3 (C-1), 129.7 (C-7''), 129.5 (C-3), 127.0 (C-2), 124.3 (C-6''), 120.4 (C-5''), 117.0 (C-4a''), 112.7 (C-8''), 48.9 (C-4''), 43.4 (C-1'), 23.0 (C-2'), 21.0 (CH_3).

HRMS (+ESI): calc. for $C_{18}H_{20}BrN_4O_3S$ $[M+H]^+$: 451.0434; found: 451.0427.

IR (ATR): $\tilde{\nu}$ [cm^{-1}] = 3345 (w), 2869 (w, C–H), 2814 (w, C–H), 2756 (w, C–H), 1667 (s, C=O), 1592 (m, C=C), 1503 (w, C=C), 1444 (m), 1322 (m, RSO_2N), 1156 (vs, RSO_2N), 988 (w), 815 (m), 799 (w), 769 (vs, C–H), 750 (m, C–H), 718 (m), 704 (m).

m.p. = >203 °C (decomposition).

*3.3.3. N'-(3-(6-Bromo-2-oxo-1,4-dihydroquinazolin-3(2H)-yl)propylidene)-4-methylbenzenesulfonohydrazide (**4c**)*

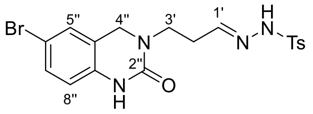

Following GP 3 starting from **SI-8c**, compound **4c** was obtained as a colorless solid (223 mg, 495 µmol, 50%).

***R*f:** 0.37 (CH_2Cl_2/ac = 7/3) [UV, $KMnO_4$].

^{1}H NMR (500 MHz, DMSO-*d*$_6$): δ [ppm] = 2.29 (s, 3H, CH_3), 2.35 – 2.40 (m, 2H, H-2'), 3.37 (t, ^3J = 6.8 Hz, 2H, H-3'), 4.26 (s, 2H, H-4''), 6.71 (d, ^3J = 8.5 Hz, 1H, H-8''), 7.20 (d, ^4J = 2.2 Hz, 1H, H-5''), 7.25 (d, ^3J = 5.3 Hz, 1H, H-1'), 7.27 – 7.32 (m, 3H, H-3, H-6''), 7.62 – 7.66 (m, 2H, H-2), 9.28 (bs, 1H, NH), 11.05 (bs, 1H, NNH).

^{13}C NMR (126 MHz, DMSO-*d*$_6$): δ [ppm] = 153.1 (C-2''), 149.4 (C-1'), 143.2 (C-4), 137.1 (C-8a''), 136.3 (C-1), 130.5 (C-7''), 129.5 (C-3), 128.1 (C-5''), 127.1 (C-2), 120.4 (C-4a''), 115.2 (C-8''), 112.1 (C-6''), 47.2 (C-4''), 43.3 (C-3'), 30.2 (C-2'), 21.0 (CH_3).

HRMS (+ESI): calc. for $C_{18}H_{20}BrN_4O_3S$ $[M+H]^+$: 451.0434; found: 451.0432.

IR (ATR): $\tilde{\nu}$ [cm^{-1}] = 3332 (w, N–H), 2937 (w, C–H), 2873 (w, C–H), 2838 (w, C–H), 1654 (vs, C=O), 1602 (w, C=C), 1501 (w, C=C), 1336 (m, RSO_2N), 1162 (vs, RSO_2N), 1061 (m, C–Br), 887 (w), 751 (m, C–H), 725 (w, C–H), 706 (m).

m.p. = >190 °C (decomposition).

*3.3.4. N'-(3-(7-Bromo-2-oxo-1,4-dihydroquinazolin-3(2H)-yl)propylidene)-4-methylbenzenesulfonohydrazide **(4d)***

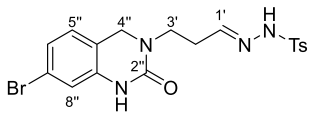

Following GP 3 starting from **SI-8d**, compound **4d** was obtained as a colorless solid (1.59 g, 3.52 mmol, 70%).

***R*f:** 0.42 (CH_2Cl_2/ac = 7/3) [UV, $KMnO_4$].

^{1}H NMR (500 MHz, DMSO-d_6): δ [ppm] = 2.28 (s, 3H, CH_3), 2.35 – 2.41 (m, 2H, H-2’), 3.36 – 3.44* (m, 2H, H-3’), 4.24 (s, 2H, H-4’’), 6.92 (d, ^4J = 2.0 Hz, 1H, H-8’’), 6.96 (d, ^3J = 8.1 Hz, 1H, H-5‘‘), 7.04 (dd, ^3J = 8.1 Hz, ^4J = 2.0 Hz, 1H, H-6’’), 7.23 – 7.28 (m, 3H, H-3, H-1’), 7.60 – 7.64 (m, 2H, H-2), 9.26 (bs, 1H, NH), 11.06 (bs, 1H, NNH).

* signal overlaps with residual water.

^{13}C NMR (126 MHz, DMSO-d_6): δ [ppm] = 153.0 (C-2‘‘), 149.3 (C-1’), 143.1 (C-4), 139.4 (C-8a’’), 136.3 (C-1), 129.5 (C-3), 127.5 (C-5’’), 127.0 (C-2), 123.5 (C-6’’), 120.2 (C-7’’), 117.3 (C-4a’’), 115.5 (C-8’’), 47.3 (C-4’’), 43.3 (C-3’), 30.2 (C-2’), 21.0 (CH_3).

HRMS (+ESI): calc. for $C_{18}H_{20}BrN_4O_3S$ $[M+H]^+$: 451.0434; found: 451.0437.

IR (ATR): $\tilde{\nu}$ [cm^{-1}] = 3347 (w, N–H), 2870 (w, C–H), 1666 (vs, C=O), 1599 (m, C=C), 1507 (w, C=C), 1442 (m), 1323 (m, RSO_2N), 1158 (vs, RSO_2N), 1050 (m, C–Br), 924 (w), 799 (s), 769 (m, C–H), 753 (m, C–H), 723 (m, C–H), 704 (m).

m.p. = >169 °C (decomposition).

*3.3.5. N'-(3-(8-Bromo-2-oxo-1,4-dihydroquinazolin-3(2H)-yl)propylidene)-4-methylbenzenesulfonohydrazide **(4e)***

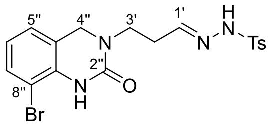

Following GP 3 starting from **SI-8e**, compound **4e** was obtained as a colorless solid (252 mg, 557 µmol, 56%).

***R*f:** 0.60 (CH_2Cl_2/ac = 7/3) [UV, $KMnO_4$].

^{1}H NMR (500 MHz, DMSO-d_6): δ [ppm] = 2.26 (s, 3H, CH_3), 2.38 – 2.43 (m, 2H, H-2’), 3.41 (t, ^3J = 6.8 Hz, 2H, H-3’), 4.30 (s, 2H, H-4’’), 6.87 (*virt.* t, $^3J \approx {}^3J$ = 7.8 Hz, 1H, H-6’’), 7.06 (d, ^3J = 7.8 Hz, 1H, H-5‘‘), 7.24 – 7.29 (m, 3H, H-3, H-1’), 7.44 (d, ^3J = 7.8 Hz, ^4J = 1.3 Hz, 1H, H-7‘’), 7.62 – 7.65 (m, 2H, H-2), 7.95 (bs, 1H, NH), 11.05 (bs, 1H, NNH).

^{13}C NMR (126 MHz, DMSO-d_6): δ [ppm] = 153.0 (C-2’’), 149.3 (C-1’), 143.2 (C-4), 136.3 (C-1), 135.2 (C-8a’’), 131.3 (C-7’’), 129.5 (C-3), 127.1 (C-2), 125.1 (C-5’’), 122.8 (C-6’’), 120.7 (C-4a’’), 106.8 (C-8’’), 47.8 (C-4’’), 43.4 (C-3’), 30.2 (C-2’), 21.0 (CH_3).

HRMS (+ESI): calc. for $C_{18}H_{20}BrN_4O_3S$ $[M+H]^+$: 451.0434; found: 451.0433.

IR (ATR): $\tilde{\nu}$ [cm^{-1}] = 3268 (w, N–H), 2870 (w, C–H), 1646 (s, C=O), 1608 (w, C=C), 1598 (w, C=C), 1508 (m, C=C), 1453 (m), 1329 (m, RSO_2N), 1163 (vs, RSO_2N), 1021 (w), 839 (w), 754 (s, C–H), 705 (s).

m.p. = >173 °C (decomposition).

*3.3.6. N'-(3-(6-Chloro-2-oxo-1,4-dihydroquinazolin-3(2H)-yl)propylidene)-4-methylbenzenesulfonohydrazide (**4f**)*

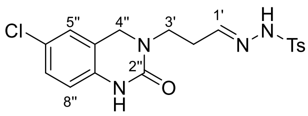

Following GP 3 starting from **SI-8f**, compound **4f** was obtained as a colorless solid (298 mg, 733 µmol, 73%).

***R*f:** 0.37 (CH_2Cl_2/ac = 7/3) [UV, $KMnO_4$].

^{1}H NMR (500 MHz, DMSO-*d*$_6$): δ [ppm] = 2.29 (s, 3H, CH_3), 2.35 – 2.40 (m, 2H, H-2'), 3.38 (t, ^3J = 6.8 Hz, 2H, H-3'), 4.26 (s, 2H, H-4''), 6.76 (d, ^3J = 8.5 Hz, 1H, H-8''), 7.08 (d, ^4J = 2.4 Hz, 1H, H-5''), 7.18 (dd, ^3J = 8.5 Hz, ^4J = 2.4 Hz, 1H, H-7''), 7.25 (t, ^3J = 5.4 Hz, 1H, H-1'), 7.27 – 7.30 (m, 2H, H-3), 7.62 – 7.66 (m, 2H, H-2), 9.27 (bs, 1H, NH), 11.05 (bs, 1H, NNH).

^{13}C NMR (126 MHz, DMSO-*d*$_6$): δ [ppm] = 153.2 (C-2''), 149.4 (C-1'), 143.2 (C-4), 136.7 (C-8a''), 136.3 (C-1), 129.5 (C-3), 127.6 (C-7''), 127.1 (C-2), 125.3 (C-5''), 124.5 (C-6''), 120.0 (C-4a''), 114.8 (C-8''), 47.3 (C-4''), 43.3 (C-3'), 30.2 (C-2'), 21.0 (CH_3).

HRMS (+ESI): calc. for $C_{18}H_{20}ClN_4O_3S$ $[M+H]^+$: 407.0939; found: 407.0942.

IR (ATR): $\tilde{\nu}$ [cm^{-1}] = 3339 (w, N–H), 2936 (w, C–H), 2870 (w, C–H), 2838 (w, C–H), 1652 (s, C=O), 1612 (w, C=C), 1598 (w, C=C), 1504 (w, C=C), 1445 (m), 1336 (m, RSO_2N), 1163 (s, RSO_2N), 1060 (m, C–Cl), 889 (w), 816 (s), 752 (m, C–H), 706 (m).

m.p. = >200 °C (decomposition).

*3.3.7. N'-(3-(7-Fluoro-2-oxo-1,4-dihydroquinazolin-3(2H)-yl)propylidene)-4-methylbenzenesulfonohydrazide (**4g**)*

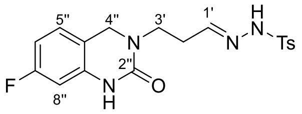

Following GP 3 starting from **SI-8g**, compound **4g** was obtained as a colorless solid (194 mg, 496 µmol, 37%).

***R*f:** 0.38 (CH_2Cl_2/ac = 7/3) [UV, $KMnO_4$].

^{1}H NMR (500 MHz, DMSO-*d*$_6$): δ [ppm] = 2.29 (s, 3H, CH_3), 2.38 (td, ^3J = 6.8 Hz, ^3J = 5.3 Hz, 2H, H-2'), 3.38 (t, ^3J = 6.9 Hz, 2H, H-3'), 4.25 (s, 2H, H-4''), 6.53 (dd, $^3J_{H\text{–}F}$ =10.3 Hz, ^4J = 2.6 Hz, 1H, H-8''), 6.68 (*virt.* t, $^3J_{H\text{–}F} \approx {}^3J_{H\text{–}H}$ = 8.7 Hz, 1H, H-6''), 7.03 (dd, ^3J = 8.7 Hz, $^4J_{H\text{–}F}$ = 6.1 Hz, 1H, H-5''), 7.25 (t, ^3J = 5.3 Hz, 1H, H-1'), 7.26 – 7.30 (m, 2H, H-3), 7.61 – 7.65 (m, 2H, H-2), 9.26 (bs, 1H, NH), 11.04 (bs, 1H, NNH).

^{13}C NMR (126 MHz, DMSO-*d*$_6$): δ [ppm] = 161.70 (d, $^1J_{C\text{–}F}$ = 240.9 Hz, C-7''), 153.0 (C-2''), 149.4 (C-1'), 143.1 (C-4), 139.34 (d, $^2J_{C\text{–}F}$ = 11.2 Hz, C-8a''), 136.3 (C-1), 129.5 (C-3), 127.21 (d, $^3J_{C\text{–}F}$ = 9.8 Hz, C-5''), 127.1 (C-2), 114.1 (d, $^4J_{C\text{–}F}$ = 2.7 Hz, C-4a''), 107.4 (d, $^2J_{C\text{–}F}$ = 21.6 Hz, C-6''), 100.1 (d, $^2J_{C\text{–}F}$ = 25.8 Hz, C-8''), 47.2 (C-4''), 43.3 (C-3'), 30.2 (C-2'), 21.0 (CH_3).

^{19}F NMR (471 MHz, $CDCl_3$): δ [ppm] = −114.8 (ddd, $^3J_{H\text{–}F}$ =10.3 Hz, $^3J_{H\text{–}F}$ = 8.7 Hz, $^4J_{H\text{–}F}$ = 6.1 Hz, 1F).

HRMS (+ESI): calc. for $C_{18}H_{20}FN_4O_3S$ $[M+H]^+$: 391.1235; found: 391.1234.

IR (ATR): $\tilde{\nu}$ [cm^{-1}] = 3368 (w, N–H), 2932 (w, C–H), 2865 (w, C–H), 2841 (w, C–H), 1654 (m, C=O), 1617 (m, C=C), 1522 (w, C=C), 1447 (m), 1330 (m, RSO_2N), 1261 (m, C–F), 1162 (vs, RSO_2N), 1061 (m), 871 (m), 797 (m), 757 (m, C–H), 740 (m, C–H), 705 (m).

m.p. = >202 °C (decomposition).

*3.3.8. N'-(3-(2-Oxo-7-(trifluoromethyl)-1,4-dihydroquinazolin-3(2H)-yl)propylidene)-4-methylbenzenesulfonohydrazide (**4h**)*

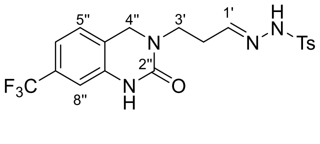

Following GP 3 starting from **SI-8h**, compound **4h** was obtained as a colorless solid (729 mg, 1.65 mmol, 83%).

***R*f**: 0.67 (CH_2Cl_2/ac = 7/3) [UV, $KMnO_4$].

^{1}H NMR (500 MHz, DMSO-d_6): δ [ppm] = 2.25 (s, 3H, CH_3), 2.40 (td, ^3J = 6.7 Hz, ^3J = 5.3 Hz, 2H, H-2'), 3.40 (t, ^3J = 6.7 Hz, 2H, H-3'), 4.35 (s, 2H, H-4''), 7.05 (d, ^4J = 1.7 Hz, 1Hz, H-8''), 7.19 – 7.28 (m, 5H, H-3, H-1', H-5'', H-6''), 7.61 – 7.65 (m, 2H, H-2), 9.41 (bs, 1H, NH), 11.05 (bs, 1H, NNH).

^{13}C NMR (126 MHz, DMSO-d_6): δ [ppm] = 152.9 (C-4''), 149.4 (C-1'), 143.1 (C-4), 138.5 (C-8a''), 136.3 (C-1), 129.5 (C-3), 128.53 (q, $^2J_{C\text{–}F}$ = 31.8 Hz, C-7''), 127.0 (C-2), 126.7 (C-5''), 124.1 (d, $^1J_{C\text{–}F}$ = 272.0 Hz, CF_3), 122.4 (C-4a''), 117.5 (q, $^3J_{C\text{–}F}$ = 4.0 Hz, C-6''), 109.4 (q, $^3J_{C\text{–}F}$ = 4.2 Hz, C-6''), 47.5 (C-4''), 43.3 (C-3'), 30.2 (C-2'), 20.9 (CH_3).

^{19}F NMR (471 MHz, DMSO-d_6): δ [ppm] = −61.25 (s, 3F).

HRMS (+ESI): calc. for $C_{19}H_{20}F_3N_4O_3S$ $[M+H]^+$: 441.1203; found: 441.1194.

IR (ATR): $\tilde{\nu}$ [cm^{-1}] = 3294 (w, N–H), 2925 (w, C–H), 2882 (w, C–H), 2840 (w, C–H), 1667 (s, C=O), 1610 (w, C=C), 1529 (w, C=C), 1446 (w), 1330 (s, RSO_2N), 1164 (vs, RSO_2N), 1110 (s, C–F), 950 (m), 814 (s), 788 (w, C–H), 755 (m, C–H), 744 (m, C–H), 720 (m), 706 (m).

m.p. = >213 °C (decomposition).

*3.3.9. N'-(3-(2-Oxo-6-vinyl-1,4-dihydroquinazolin-3(2H)-yl)propylidene)-4-methylbenzenesulfonohydrazide (**4i**)*

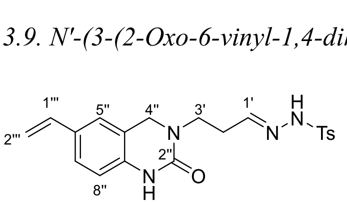

Following GP 3 starting from **SI-8i**, compound **4i** was obtained as a colorless solid (254 mg, 637 µmol, 52%).

***R*f**: 0.43 (CH_2Cl_2/ac = 7/3) [UV, $KMnO_4$].

^{1}H NMR (500 MHz, DMSO-d_6): δ [ppm] = 2.27 (s, 3H, CH_3), 2.36 – 2.43 (m, 2H, H-2'), 3.39 (t, ^3J = 6.8 Hz, 2H, H-3'), 4.28 (s, 2H, H-4''), 5.11 (dd, ^3J = 10.9 Hz, ^2J = 1.1 Hz, 1H, H-2'''$_{trans}$), 5.64 (dd, ^3J = 17.6 Hz, ^2J = 1.1 Hz, 1H, H-2'''$_{cis}$), 6.62 (dd, ^3J = 17.6 Hz, ^3J = 10.9 Hz, 1H, H-1''), 6.73 (d, ^3J = 8.2 Hz, 1H, H-8''), 7.13 (d, ^4J = 1.9 Hz, 1H, H-5''), 7.23 – 7.29 (m, 4H, H-3, H-1', H-7''), 7.62 – 7.65 (m, 2H, H-2), 9.22 (bs, 1H, NH), 11.04 (bs, 1H, NNH).

^{13}C NMR (126 MHz, DMSO-*d*$_6$): δ [ppm] = 153.3 (C-2''), 149.5 (C-1'), 143.2 (C-4), 137.4 (C-8a''), 136.3 (C-1†), 136.2 (C-1'''†), 130.3 (C-8a''), 129.6 (C-3), 127.0 (C-2), 125.9 (C-7''), 123.3 (C-5''), 118.0 (C-4a''), 113.4 (C-8''), 111.8 (C-2'''), 47.7 (C-4''), 43.4 (C-3'), 30.2 (C-2'), 21.0 (CH_3).

† assignment is interconvertible

HRMS (+ESI): calc. for $C_{20}H_{23}N_4O_3S$ $[M+H]^+$: 399.1485; found: 399.1480.

IR (ATR): $\tilde{\nu}$ [cm^{-1}] = 3339 (w, N–H), 2870 (w, C–H), 2834 (w, C–H), 1653 (s, C=O), 1619 (w, C=C), 1600 (w, C=C), 1520 (w, C=C), 1447 (m), 1322 (m, RSO_2N), 1164 (vs, RSO_2N), 1019 (w), 817 (m), 765 (w, C–H), 753 (m, C–H), 723 (w, C–H), 706 (m).

m.p. = >182 °C (decomposition).

*3.3.10. N'-(3-(6-Allyl-2-oxo-1,4-dihydroquinazolin-3(2H)-yl)propylidene)-4-methylbenzenesulfonohydrazide (**4j**)*

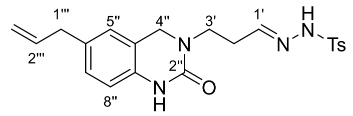

Following GP 3 starting from **SI-8j**, compound **4j** was obtained as a colorless solid (443 mg, 1.07 mmol, 79%).

***R*f**: 0.55 (CH_2Cl_2/ac = 7/3) [UV, $KMnO_4$].

^{1}H NMR (500 MHz, DMSO-*d*$_6$): δ [ppm] = 2.28 (s, 3H, CH_3), 2.35 – 2.41 (m, 2H, H-2'), 3.26 (d, ^3J = 6.7 Hz, 2H, H-1'''), 3.38 (t, ^3J = 6.8 Hz, 2H, H-3'), 4.25 (s, 2H, H-4''), 5.00 – 5.08 (m, 2H, H-3'''$_{trans}$, H-3'''$_{cis}$), 5.90 (ddt, ^3J = 16.8 Hz, ^3J = 9.9 Hz, ^3J = 6.7 Hz, 1H, H-2'''), 6.69 (d, ^3J = 8.0 Hz, 1H, H-8''), 6.82 (d, ^4J = 2.0 Hz, 1H, H-5''), 6.94 (dd, ^3J = 8.0 Hz, ^4J = 2.0 Hz, 1H, H-7''), 7.24 – 7.30 (m, 3H, H-3, H-1'), 7.62 – 7.66 (m, 2H, H-2), 9.07 (bs, 1H, NH), 11.02 (bs, 1H, NNH).

^{13}C NMR (126 MHz, DMSO-*d*$_6$): δ [ppm] = 153.5 (C-2''), 149.6 (C-1'), 143.2 (C-4), 138.0 (C-2'''), 136.3 (C-1), 135.8 (C-8a''), 132.2 (C-6''), 129.6 (C-3), 127.8 (C-6''), 127.1 (C-2), 125.4 (C-5''), 117.9 (C-4a''), 115.6 (C-3'''), 113.3 (C-8''), 47.8 (C-4''), 43.4 (C-3'), 30.2 (C-2'), 21.0 (CH_3).

HRMS (+ESI): calc. for $C_{21}H_{25}N_4O_3S$ $[M+H]^+$: 413.1642; found: 413.1637.

IR (ATR): $\tilde{\nu}$ [cm^{-1}] = 3354 (w, N–H), 2934 (w, C–H), 2860 (w, C–H), 2837 (w, C–H), 1648 (m, C=O), 1606 (w, C=C), 1516 (w, C=C), 1447 (m), 1326 (m, RSO_2N), 1159 (vs, RSO_2N), 998 (w), 818 (s), 774 (w, C–H), 754 (m, C–H), 706 (s).

m.p. = >184 °C (decomposition).

*3.3.11. N'-(3-(8-Methyl-2-oxo-1,4-dihydroquinazolin-3(2H)-yl)propylidene)-4-methylbenzenesulfonohydrazide (**4k**)*

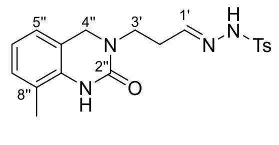

Following GP 3 starting from **SI-8k**, compound **4k** was obtained as a colorless solid (104 mg, 269 µmol, 57%).

***R*f:** 0.30 (CH_2Cl_2/ac = 7/3) [UV, $KMnO_4$].

¹H NMR (500 MHz, DMSO-*d*6): δ [ppm] = 2.17 (s, 3H, C-8''-CH_3), 2.29 (s, 3H, CH_3), 2.40 (td, ^3J = 6.8 Hz, ^3J = 5.2 Hz, 2H, H-2'), 3.41 (t, ^3J = 6.8 Hz, 2H, H-3'), 4.27 (s, 2H, H-4''), 6.80 (*virt. t*, $^3J \approx ^3J$ = 7.4 Hz, 1H, H-6''), 6.86 (d, ^3J = 7.4 Hz, 1H, H-5''), 6.98 (d, ^3J = 7.4 Hz, 1H, H-7''), 7.26 – 7.31 (m, 3H, H-3, H-1'), 7.63 – 7.67 (m, 2H, H-2), 8.41 (bs, 1H, NH), 11.04 (bs, 1H, NNH).

¹³C NMR (126 MHz, DMSO-*d*6): δ [ppm] = 153.8 (C-2''), 149.5 (C-1'), 143.2 (C-4), 136.3 (C-1), 135.6 (C-8a''), 129.5 (C-3), 129.2 (C-7''), 127.1 (C-2), 123.2 (C-5''), 121.7 (C-8''), 121.0 (C-6''), 118.1 (C-4a''), 47.9 (C-4''), 43.4 (C-3'), 30.2 (C-2'), 21.0 (CH_3), 17.1 (C-8''-*C*H_3).

HRMS (+ESI): calc. for $C_{19}H_{23}N_4O_4S$ $[M+H]^+$: 387.1485; found: 387.1478.

IR (ATR): $\tilde{\nu}$ [cm^{-1}] = 3345 (w, N–H), 3294 (w, N–H), 3229 (w), 2956 (w, C–H), 2867 (w, C–H), 1651 (vs, C=O), 1616 (m, C=C), 1599 (w, C=C), 1505 (m, C=C), 1449 (m), 1323 (m, RSO_2N), 1159 (vs, RSO_2N), 1051 (m), 953 (w), 816 (m), 779 (s, C–H), 767 (m, C–H), 727 (m, C–H), 705 (m).

m.p. = >163 °C (decomposition).

*3.3.12. N'-(3-(6-Methoxy-2-oxo-1,4-dihydroquinazolin-3(2H)-yl)propylidene)-4-methylbenzenesulfonohydrazide (**4l**)*

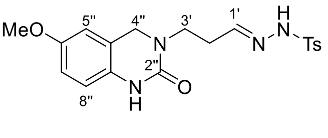

Following GP 3 starting from **SI-8l**, compound **4l** was obtained as a colorless solid (463 mg, 1.15 mmol, 57%).

***R*f:** 0.31 (CH_2Cl_2/ac = 7/3) [UV, $KMnO_4$].

¹H NMR (500 MHz, DMSO-*d*6): δ [ppm] = 2.29 (s, 3H, CH_3), 2.34 – 2.40 (m, 2H, H-2'), 3.38 (t, ^3J = 6.9 Hz, 2H, H-3'), 3.68 (s, 3H, OCH_3), 4.25 (s, 2H, H-4''), 6.62 (d, ^4J =2.7 Hz, 1H, H-5''), 6.68 (d, ^3J = 8.6 Hz, 1H, H-8''), 6.73 (dd, ^3J = 8.6 Hz, ^4J = 2.7 Hz, 1H, H-7''), 7.26 (d, ^3J = 5.3 Hz, 1H, H-1'), 7.27 – 7.31 (m, 2H, H-3), 7.62 – 7.66 (m, 2H, H-2), 8.96 (bs, 1H, NH), 11.03 (bs, 1H, NNH).

¹³C NMR (126 MHz, DMSO-*d*6): δ [ppm] = 153.9 (C-6''), 153.6 (C-2''), 149.5 (C-1'), 143.2 (C-4), 136.3 (C-1), 131.2 (C-8a''), 129.6 (C-3), 127.1 (C-2), 118.9 (C-4a''), 114.1 (C-8''), 113.4 (C-7''), 110.8 (C-5''), 55.3 (OCH_3), 48.0 (C-4''), 43.3 (C-3') , 30.2 (C-2'), 21.0 (CH_3).

HRMS (+ESI): calc. for $C_{19}H_{23}N_4O_4S$ $[M+H]^+$: 403.1435; found: 403.1429.

IR (ATR): $\tilde{\nu}$ [cm^{-1}] = 3169 (w, N–H), 3100 (w, N–H), 2947 (w, C–H), 2917 (w, C–H), 1659 (s, C=O), 1606 (w, C=C), 1500 (s, C=C), 1454 (m), 1323 (s, RSO_2N), 1248 (s, C–O), 1162 (vs, RSO_2N), 1097 (m), 820 (s), 751 (m, C–H).

m.p. = >192 °C (decomposition).

*3.3.13. N'-(3-(8-Methoxy-2-oxo-1,4-dihydroquinazolin-3(2H)-yl)propylidene)-4-methylbenzenesulfonohydrazide (**4m**)*

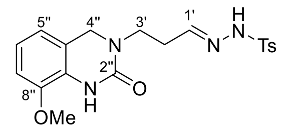

Following GP 3 starting from **SI-8m**, compound **4m** was obtained as a colorless solid (539 mg, 1.34 mmol, 66%).

***R*f:** 0.30 (CH_2Cl_2/ac = 7/3) [UV, $KMnO_4$].

¹H NMR (500 MHz, DMSO-d_6): δ [ppm] = 2.27 (s, 3H, CH_3), 2.36 – 2.42 (m, 2H, H-2’), 3.39 (t, ^3J = 6.8 Hz, 2H, H-3’), 3.78 (s, 3H, OCH_3), 4.28 (s, 2H, H-4’’), 6.63 (dd, ^3J = 5.2 Hz, ^4J = 3.7 Hz, 1H, H-6’’), 6.83 – 6.89 (m, 2H, H-5’’, H-7’’), 7.06 (d, ^3J = 7.8 Hz, 1H, H-5’‘), 7.23 – 7.31 (m, 3H, H-3, H-1’), 7.60 – 7.66 (m, 2H, H-2), 7.92 (bs, 1H, NH), 11.04 (bs, 1H, NNH).

¹³C NMR (126 MHz, DMSO-d_6): δ [ppm] = 153.1 (C-2’’), 149.5 (C-1’), 144.9 (C-8’’), 143.2 (C-4), 136.3 (C-1), 129.5 (C-3), 127.1 (C-2), 126.4 (C-8a’’), 121.4 (C-6’’), 118.5 (C-4a’’), 117.4 (C-5’’), 109.9 (C-7’’), 55.8 (OCH_3), 47.8 (C-4’’), 43.4 (C-3’), 30.2 (C-2’), 21.0 (CH_3).

HRMS (+ESI): calc. for $C_{19}H_{23}N_4O_4S$ $[M+H]^+$: 403.1435; found: 403.1429.

IR (ATR): $\tilde{\nu}$ [cm^{-1}] = 3237 (w, N–H), 2933 (w, C–H), 1639 (s, C=O), 1606 (w, C=C), 1598 (w, C=C), 1510 (s, C=C), 1455 (m), 1332 (m, RSO_2N), 1254 (m, C–O), 1161 (vs, RSO_2N), 911 (m), 811 (m), 763 (s, C–H), 724 (m, C–H).

m.p. = >171 °C (decomposition).

*3.3.14. N'-(3-(7-(Furan-3-yl)-2-oxo-1,4-dihydroquinazolin-3(2H)-yl)propylidene)-4-methylbenzenesulfonohydrazide (**4n**)*

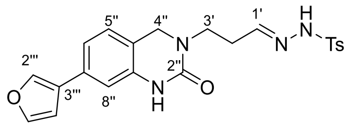

Following GP 3 starting from **SI-8n**, compound **4n** was obtained as a colorless solid (294 mg, 669 µmol, 62%).

***R*f**: 0.61 (CH_2Cl_2/ac = 7/3) [UV, $KMnO_4$].

¹H NMR (500 MHz, DMSO-d_6): δ [ppm] = 2.25 (s, 3H, CH_3), 2.40 (td, ^3J = 6.8 Hz, ^3J = 5.0 Hz, 2H, H-2’), 3.40 (t, ^3J = 6.8 Hz, 2H, H-3’), 4.28 (s, 2H, H-4’’), 6.78 (dd, ^3J = 1.9 Hz, ^3J = 0.9 Hz, 1H, H-4‘‘‘), 6.92 (d, ^4J = 1.7 Hz, 1H, H-8’’), 7.02 (d, ^3J = 7.8 Hz, 1H, H-5’’), 7.12 (dd, ^3J = 7.8 Hz, ^4J = 1.7 Hz, 1H, H-6’‘), 7.24 – 7.29 (m, 3H, H-3, H-1’), 7.62 – 7.65 (m, 2H, H-2), 7.74 (dd, ^3J = 1.9 Hz, ^3J = 1.6 Hz, 1H, H-5’’’), 8.04 (dd, ^3J = 1.6 Hz, ^3J = 0.9 Hz, 1H, H-2’’’), 9.13 (bs, 1H, NH), 11.05 (bs, 1H, NNH).

¹³C NMR (126 MHz, DMSO-d_6): δ [ppm] = 153.4 (C-2’’), 149.5 (C-1’), 144.5 (C-5’’’), 143.2 (C-4), 139.1 (C-2’’’), 138.1 (C-7’’), 136.3 (C-1), 131.5 (C-8a’’), 129.6 (C-3), 127.0 (C-2), 126.0 (C-5’’), 125.6 (C-3’’’), 118.6 (C-6’’), 116.7 (C-4a’’), 110.1 (C-8’’), 108.7 (C-4’’’), 47.6 (C-4’’), 43.4 (C-3’), 30.2 (C-2’), 21.0 (CH_3).

HRMS (+ESI): calc. for $C_{22}H_{23}N_4O_4S$ $[M+H]^+$: 439.1435; found: 439.1431.

IR (ATR): $\tilde{\nu}$ [cm^{-1}] = 3354 (w, N–H), 2868 (w, C–H), 1667 (s, C=O), 1633 (w, C=C), 1607 (w, C=C), 1595 (w, C=C), 1505 (w, C=C), 1444 (m), 1314 (m, RSO_2N), 1154 (vs, RSO_2N), 873 (s), 788 (s, C–H), 752 (m, C–H), 725 (m, C–H).

m.p. = >216 °C (decomposition).

*3.3.15. N'-(3-(2-Oxo-7-(thiophen-3-yl)-1,4-dihydroquinazolin-3(2H)-yl)propylidene)-4-methylbenzenesulfonohydrazide (**4o**)*

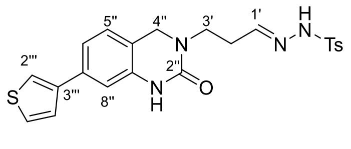

Following GP 3 starting from **SI-8o**, compound **4o** was obtained as a colorless solid (317 mg, 697 µmol, 66%).

***R*f**: 0.66 (CH_2Cl_2/ac = 7/3) [UV, $KMnO_4$].

^{1}H NMR (500 MHz, DMSO-*d*$_6$): δ [ppm] = 2.25 (s, 3H, CH_3), 2.40 (td, ^3J = 6.8 Hz, ^3J = 5.3 Hz, 2H, H-2’), 3.41 (t, ^3J = 6.8 Hz, 2H, H-3’), 4.29 (s, 2H, H-4’’), 7.03 – 7.06 (m, 2H, H-5’’, H-8’’), 7.21 (dd, ^3J = 7.8 Hz, ^4J = 1.8 Hz, 1H, H-6’’), 7.25 – 7.29 (m, 3H, H-3, H-1’), 7.40 (dd, ^3J = 5.1 Hz, ^3J = 1.4 Hz, 1H, H-4’’’), 7.62 – 7.66 (m, 3H, H-2, H-5’’’), 7.71 (dd, ^3J = 3.0 Hz, ^3J = 1.4 Hz, 1H, H-2’’’), 9.16 (bs, 1H, NH), 11.05 (bs, 1H, NNH).

^{13}C NMR (126 MHz, DMSO-*d*$_6$): δ [ppm] = 153.4 (C-2’’), 149.5 (C-1’), 143.2 (C-4), 141.2 (C-3’’’), 138.1 (C-8a’’), 136.3 (C-1), 134.8 (C-7’’), 129.6 (C-3), 127.3 (C-5’’’), 127.0 (C-2), 126.1 (C-5’’), 126.0 (C-4’’’), 120.9 (C-2’’’), 119.1 (C-6’’), 116.8 (C-4a’’), 110.7 (C-8’’), 47.6 (C-4’’), 43.4 (C-3’), 30.2 (C-2’), 21.0 (CH_3).

HRMS (+ESI): calc. for $C_{22}H_{23}N_4O_3S_2$ $[M+H]^+$: 455.1201; found: 455.1206.

IR (ATR): $\tilde{\nu}$ [cm^{-1}] = 3367 (w, N–H), 2865 (w, C–H), 2756 (w, C–H), 1648 (s, C=O), 1596 (m, C=C), 1510 (w, C=C), 1449 (m), 1319 (m, RSO_2N), 1157 (s, RSO_2N), 1009 (w), 820 (m), 782 (s, C–H), 761 (s, C–H), 721 (m), 707 (m).

m.p. = >222 °C (decomposition).

*3.3.16. tert-Butyl 2-(2-oxo-3-(3-(2-tosylhydrazineylidene)propyl)-1,2,3,4-tetrahydroquinazolin-7-yl)-1H-pyrrole-1-carboxylate (**4p**)*

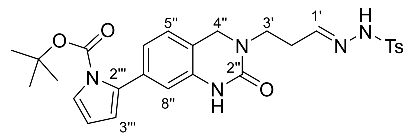

Following GP 3 starting from **SI-8p**, compound **4p** was obtained as a colorless solid (283 mg, 527 µmol, 51%).

***R*f**: 0.70 (CH_2Cl_2/ac = 7/3) [UV, $KMnO_4$].

^{1}H NMR (500 MHz, DMSO-*d*$_6$): δ [ppm] = 1.34 [s, 9H, $(CH_3)_3$], 2.30 (s, 3H, CH_3), 2.40 (td, ^3J = 6.9 Hz, ^3J = 5.2 Hz, 2H, H-2’), 3.40 (t, ^3J = 6.8 Hz, 2H, H-3’), 4.32 (s, 2H, H-4’’), 6.19 (dd, ^3J = 3.3 Hz, ^4J = 1.8 Hz, 1H, H-3‘’’), 6.25 (*virt.* t, $^3J \approx {}^3J$ = 3.3 Hz, 1H, H-4‘‘‘), 6.69 (d, ^4J = 1.7 Hz, 1H, H-8’’), 6.83 (dd, ^3J = 7.8 Hz, ^4J = 1.7 Hz, 1H, H-6’’), 7.00 (d, ^3J = 7.8 Hz, 1H, H-5’’), 7.27 (t, ^3J = 5.2 Hz, 1H, H-1‘), 7.30 – 7.33 (m, 3H, H-3, H-5’‘‘), 7.64 – 7.67 (m, 2H, H-2), 9.21 (bs, 1H, NH), 11.04 (bs, 1H, NNH).

^{13}C NMR (126 MHz, DMSO-*d*$_6$): δ [ppm] = 153.5 (C-2’’), 149.5 (C-1’), 148.7 (COO), 143.2 (C-4), 137.0 (C-8a’’), 136.3 (C-1), 134.2 (C-2’’’), 133.2 (C-7’’), 129.6 (C-3), 127.1 (C-2), 124.8 (C-5’’’), 122.7 (C-5’’), 121.6 (C-6’’), 116.9 (C-4a’’), 114.2 (C-3’’’), 113.7 (C-8’’), 110.9 (C-4’’’), 83.7 [*C*$(CH_3)_3$], 47.7 (C-4’’), 43.4 (C-3’), 30.2 (C-2’), 27.2 [C(*C*$H_3)_3$], 21.0 (CH_3).

HRMS (+ESI): calc. for $C_{27}H_{32}N_5O_5S$ $[M+H]^+$: 538.2119; found: 538.2116.

IR (ATR): $\tilde{\nu}$ [cm^{-1}] = 3367 (w, N–H), 2875 (w, C–H), 1742 (s, C=O), 1657 (s, C=O), 1600 (m, C=C), 1475 (s), 1319 (s, RSO_2N), 1145 (vs, RSO_2N), 949 (m), 807 (s), 771 (s, C–H), 751 (s, C–H), 727 (vs, C–H), 710 (s).

m.p. = >198 °C (decomposition).

*3.3.17. N'-(3-(2-Oxo-7-(pyridin-3-yl)-1,4-dihydroquinazolin-3(2H)-yl)propylidene)-4-methylbenzenesulfonohydrazide **(4q)***

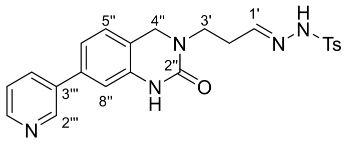

Following GP 3 starting from **SI-8q**, compound **4q** was obtained as a colorless solid (245 mg, 545 µmol, 68%).

***R*f**: 0.08 (CH_2Cl_2/ac = 7/3) [UV, $KMnO_4$].

^{1}H NMR (500 MHz, DMSO-d_6): δ [ppm] = 2.25 (s, 3H, CH_3), 2.37 – 2.45 (m, 2H, H-2’), 3.42 (t, ^3J = 6.8 Hz, 2H, H-3’), 4.34 (s, 2H, H-4’’), 7.04 (d, ^4J = 1.7 Hz, 1H, H-8‘‘), 7.14 (d, ^3J = 7.9 Hz, 1H, H-5‘‘), 7.22 (dd, ^3J = 7.9 Hz, ^4J = 1.7 Hz, 1H, H-6’’), 7.26 – 7.30 (m, 3H, H-3, H-1’), 7.49 (ddd, ^3J = 8.0 Hz, ^3J = 4.8 Hz, ^5J = 0.9 Hz, 1H, H-5’’’), 7.63 – 7.66 (m, 2H, H-2), 7.96 (ddd, ^3J = 8.0 Hz, ^4J = 2.4 Hz, ^4J = 1.6 Hz, 1H, H-4‘‘‘), 8.57 (dd, ^3J = 4.8 Hz, ^4J = 1.6 Hz, 1H, H-6‘‘‘), 8.78 (dd, ^4J = 2.4 Hz, ^5J = 0.9 Hz, 1H, H-2’’’), 9.25 (bs, 1H, NH), 11.06 (bs, 1H, NNH).

^{13}C NMR (126 MHz, DMSO-d_6): δ [ppm] = 153.4 (C-2’’), 149.5 (C-1’), 148.6 (C-6’’’), 147.4 (C-2’’’), 143.2 (C-4), 138.4 (C-8a’’), 136.7 (C-1), 136.1 (C-3’’’), 135.5 (C-7’’), 134.1 (C-4’’’), 129.6 (C-3), 127.0 (C-2), 126.4 (C-5’’), 124.1 (C-5’’’), 119.7 (C-6’’), 118.0 (C-4a’’), 111.4 (C-8’’), 47.6 (C-4’’), 43.4 (C-3’), 30.3 (C-2’), 21.0 (CH_3).

HRMS (+ESI): calc. for $C_{23}H_{24}N_5O_3S$ $[M+H]^+$: 450.1594; found: 450.1593.

IR (ATR): $\tilde{\nu}$ [cm^{-1}] = 3364 (w, N–H), 2871 (w, C–H), 2823 (w, C–H), 1649 (vs, C=O), 1598 (m, C=C), 1447 (m), 1324 (s, RSO_2N), 1160 (vs, RSO_2N), 952 (m), 814 (m), 799 (s), 782 (m, C–H), 756 (m, C–H), 738 (m, C–H), 716 (m), 705 (s).

m.p. = >209 °C (decomposition).

*3.3.18. Ethyl (E)-3-(2-oxo-3-(3-(2-tosylhydrazineylidene)propyl)-1,2,3,4-tetrahydroquinazolin-7-yl)acrylate **(4r)***

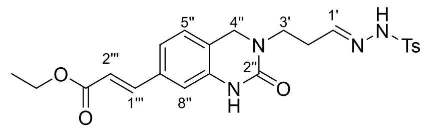

Following GP 3 starting from **SI-8r**, compound **4r** was obtained as a colorless solid (523 mg, 1.11 mmol, 40%).

***R*f**: 0.74 (CH_2Cl_2/ac = 7/3) [UV, $KMnO_4$].

^{1}H NMR (500 MHz, DMSO-d_6): δ [ppm] = 1.26 (t, ^3J = 7.1 Hz, 3H, OCH_2CH_3), 2.26 (s, 3H, CH_3), 2.36 – 2.42 (m, 2H, H-2’), 3.39 (t, ^3J = 6.8 Hz, 2H, H-3’), 4.18 (q, ^3J = 7.1 Hz, 2H, OCH_2), 4.31 (s, 2H, H-4’’), 6.43 (d, ^3J = 16.0 Hz, 1H, H-2‘‘‘), 6.96 (d, ^4J = 1.7 Hz, 1H, H-8’’), 7.06 (d, ^3J = 7.8 Hz, 1H, H-5’’), 7.23 – 7.28 (m, 4H, H-3, H-1’, H-6’’), 7.53 (d, ^3J = 16.0 Hz, 1H, H-1’’’), 7.61 – 7.65 (m, 2H, H-2), 9.23 (bs, 1H, NH), 11.05 (bs, 1H, NNH).

^{13}C NMR (126 MHz, DMSO-d_6): δ [ppm] = 166.1 (COO), 153.2 (C-2’’), 149.4 (C-1’), 144.1 (C-1’’’), 143.2 (C-4), 138.2 (C-8a’’), 136.3 (C-1), 133.5 (C-7’’), 129.5 (C-3), 127.0 (C-2), 126.2 (C-5’’), 121.2 (C-6’’), 120.5 (C-4a’’), 117.9 (C-2’’’), 112.3 (C-8), 60.2 (OCH_2), 47.7 (C-4’’), 43.3 (C-3’), 30.2 (C-2’), 21.0 (CH_3), 14.3 (OCH_2CH_3).

HRMS (+ESI): calc. for $C_{27}H_{27}N_4O_5S$ $[M+H]^+$: 471.1697; found: 471.1692.

IR (ATR): $\tilde{\nu}$ [cm^{-1}] = 3345 (w, N–H), 2887 (w, C–H), 1710 (m, C=O), 1652 (s, C=O), 1637 (m, C=C), 1597 (m, C=C), 1526 (w, C=C), 1435 (w), 1327 (m, RSO_2N), 1270 (s, C–O), 1155 (vs, RSO_2N), 979 (m), 814 (m), 748 (m, C–H), 732 (w), 720 (w), 704 (m).

m.p. = >196 °C (decomposition).

*3.3.19. N'-(3-(6-(4-Methoxyphenyl)-2-oxo-1,4-dihydroquinazolin-3(2H)-yl)propylidene)-4-methylbenzenesulfonohydrazide (**4s**)*

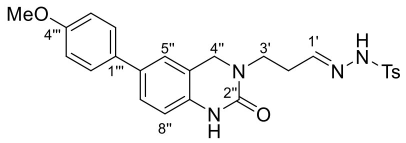

Following GP 3 starting from **SI-8s**, compound **4s** was obtained as a colorless solid (365 mg, 763 µmol, 64%).

***R*f:** 0.40 (CH_2Cl_2/ac = 7/3) [UV, $KMnO_4$].

^{1}H NMR (500 MHz, DMSO-d_6): δ [ppm] = 2.25 (s, 3H, CH_3), 2.38 – 2.44 (m, 2H, H-2'), 3.41 (t, ^3J = 6.8 Hz, 2H, H-3'), 3.78 (s, 3H, OCH_3), 4.34 (s, 2H, H-4''), 6.81 (d, ^3J =8.3 Hz, 1H, H-8''), 6.97 – 7.02 (m, 2H, H-3'''), 7.25 – 7.30 (m, 4H, H-3, H-1', H-5''), 7.39 (dd, ^3J = 8.3 Hz, ^4J = 2.2 Hz, 1H, H-7''), 7.49 – 7.54 (m, 2H, H-2'''), 7.62 – 7.67 (m, 2H, H-2), 9.21 (bs, 1H, NH), 11.05 (bs, 1H, NNH).

^{13}C NMR (126 MHz, DMSO-d_6): δ [ppm] = 158.9 (C-4'''), 153.8 (C-2''), 149.9 (C-1'), 143.6 (C-4), 137.0 (C-8a''), 136.7 (C-1), 133.2 (C-6''), 132.7 (C-1'''), 130.0 (C-3), 127.5 (C-2'''), 127.5 (C-2), 126.1 (C-7''), 123.7 (C-5''), 118.8 (C-6''), 114.8 (C-3'''), 114.1 (C-8''), 55.6 (OCH_3), 48.3 (C-4''), 43.8 (C-3'), 30.7 (C-2'), 21.4 (CH_3).

HRMS (+ESI): calc. for $C_{25}H_{27}N_4O_4S$ $[M+H]^+$: 479.1748; found: 479.1742.

IR (ATR): $\tilde{\nu}$ [cm^{-1}] = 3345(w, N–H), 2941 (w, C–H), 2906 (w, C–H), 2860 (w, C–H), 2836 (w, C–H), 1651 (s, C=O), 1607 (m, C=C), 1578 (w, C=C), 1504 (m, C=C), 1467 (m), 1334 (m, RSO_2N), 1246 (s, C–O), 1158 (s, RSO_2N), 1046 (m), 814 (vs), 795 (w), 759 (m, C–H), 753 (m, C–H), 737 (w), 720 (w), 706 (m).

m.p. = >194 °C (decomposition).

*3.3.20. N'-(3-(2-Oxo-1,4-dihydropyrido[3,2-d]pyrimidin-3(2H)-yl)propylidene)-4-methylbenzenesulfonohydrazide (**4t**)*

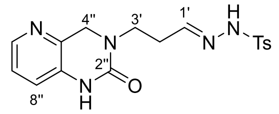

Following GP 3 starting from **SI-8t**, compound **4t** was obtained as a colorless solid (270 mg, 726 µmol, 61%).

***R*f**: 0.10 (CH_2Cl_2/ac = 7/3) [UV, $KMnO_4$].

^{1}H NMR (500 MHz, DMSO-d_6): δ [ppm] = 2.28 (s, 3H, CH_3), 2.38 – 2.43 (m, 2H, H-2'), 3.41 (t, ^3J = 6.8 Hz, 2H, H-3'), 4.37 (s, 2H, H-4''), 7.08 (dd, ^3J = 8.0 Hz, ^4J = 1.5 Hz, 1H, H-8''), 7.16 (dd, ^3J = 8.0 Hz ^3J = 4.7 Hz, 1H, H-7''), 7.24 – 7.29 (m, 3H, H-3, H-1'), 7.61 – 7.65 (m, 2H, H-2), 8.04 (dd, ^3J = 4.7 Hz, ^4J = 1.5 Hz, 1H, H-6''), 9.26 (bs, 1H, NH), 11.04 (bs, 1H, NNH).

^{13}C NMR (126 MHz, DMSO-d_6): δ [ppm] = 152.5 (C-2''), 149.5 (C-1'), 143.2 (C-4), 141.8 (C-6''), 138.7 (C-4a''), 136.3 (C-1), 133.5 (C-8a''), 129.5 (C-3), 127.0 (C-2), 123.1 (C-7''), 119.9 (C-8''), 50.2)C-4''), 43.5 (C-3'), 30.0 (C-2'), 21.1 (CH_3).

HRMS (+ESI): calc. for $C_{17}H_{20}N_5O_3S$ $[M+H]^+$: 374.1281; found: 374.1276.

IR (ATR): $\tilde{\nu}$ [cm^{-1}] = 3199 (w, N–H), 3132 (w, N–H), 2925 (w, C–H), 2857 (w, C–H), 2807 (w, C–H), 1666 (vs, C=O), 1594 (w, C=C), 1510 (w, C=C), 1457 (m), 1323 (s, RSO_2N), 1163 (vs, RSO_2N), 815 (m), 795 (m), 754 (w, C–H), 719 (m, C–H).

m.p. = >230 °C.

3.3.21. N'-(3-(2-Oxo-1,4-dihydropyrido[2,3-d]pyrimidin-3(2H)-yl)propylidene)-4-methylbenzenesulfonohydrazide (4v)

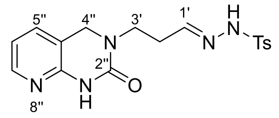

Following GP 3 starting from **SI-8v**, compound **4v** was obtained as a colorless solid (305 mg, 816 µmol, 82%).

***R*f:** 0.19 (CH_2Cl_2/ac = 7/3) [UV, $KMnO_4$].

^{1}H NMR (500 MHz, DMSO-d_6): δ [ppm] = 2.28 (s, 3H, CH_3), 2.36 – 2.42 (m, 2H, H-2'), 3.40 (t, ^3J = 6.8 Hz, 2H, H-3'), 4.29 (s, 2H, H-4''), 6.91 (dd, ^3J = 7.4 Hz, ^3J = 5.0 Hz, 1H, H-6''), 7.24 – 7.29 (m, 3H, H-3, H-1'),7.42 (dd, ^3J = 7.4 Hz, ^4J = 1.7 Hz, 1H, H-5''), 7.61 – 7.65 (m, 2H, H-2), 8.06 (dd, ^3J = 5.0 Hz, ^4J = 1.7 Hz, 1H, H-7''), 9.57 (bs, 1H, NH), 11.05 (bs, 1H, NNH).

^{13}C NMR (126 MHz, DMSO-d_6): δ [ppm] = 153.3 (C-2''), 150.4 (C-8a''), 149.4 (C-1'), 146.9 (C-7''), 143.2 (C-4), 136.2 (C-1), 133.8 (C-5''), 129.5 (C-3), 127.0 (C-2), 117.3 (C-6''), 113.2 (C-4a''), 46.6 (C-4''), 43.2 (C-3'), 30.1 (C-2'), 21.0 (CH_3).

HRMS (+ESI): calc. for $C_{17}H_{20}N_5O_3S$ $[M+H]^+$: 374.1281; found: 374.1283.

IR (ATR): $\tilde{\nu}$ [cm^{-1}] = 3063 (m, N–H), 2901 (w, C–H), 2855 (w, C–H), 1656 (vs, C=O), 1610 (s, C=C), 1498 (s, C=C), 1461 (m), 1320 (s, RSO_2N), 1153 (vs, RSO_2N), 1059 (m), 816 (s), 795 (s, C–H), 762 (m, C–H), 750 (m, C–H), 739 (m, C–H), 705 (m).

m.p. = >173 °C (decomposition).

*3.3.22. N'-(2-(2-Oxo-1,4-dihydroquinazolin-3(2H)-yl)benzylidene)-4-methylbenzenesulfonohydrazide (**4w**)*

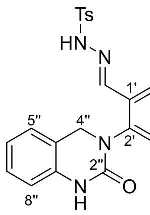

Following GP 3 starting from **SI-13w** (431 mg), compound **8w** was obtained as a colorless solid (572 mg, 3.18 mmol, 80%).

***R*f** (CH_2Cl_2/ac = 7/3): 0.23 [UV, $KMnO_4$].

^{1}H NMR (500 MHz, DMSO-d_6): δ [ppm] = 2.35 (s, 3H, CH_3), 4.45 (d, ^2J = 14.2 Hz, 1H, H^a-4''), 4.90 (d, ^2J = 14.2 Hz, 1H, H^b-4''), 6.78 (dd, ^3J = 7.6 Hz, ^4J = 1.1 Hz, 1H, H-8''), 6.92 (*virt.* td, $^3J \approx {}^3J$ = 7.6 Hz, , 1H, H-6''), 7.10 (d, ^3J = 7.6 Hz, 1H, H-5''), 7.19 (*virt.* td, $^3J \approx {}^3J$ = 7.6 Hz, ^4J = 1.5 Hz, 1H, H-7''), 7.35 (*virt.* td, $^3J \approx {}^3J$ = 7.5 Hz, ^4J = 1.5 Hz, 1H, H-5'), 7.37 – 7.41 (m, 2H, H-3), 7.43 (dd, ^3J = 8.4 Hz, ^4J = 1.5 Hz, 1H, H-3'), 7.48 (ddd, ^3J = 8.4 Hz, ^3J = 7.5 Hz, ^4J = 1.6 Hz, 1H, H-4'), 7.70 – 7.72 (m, 2H, H-2), 7.75 (dd, ^3J = 7.5 Hz, ^4J = 1.6 Hz, 1H, H-6'), 7.92 (s, 1H, CHN), 9.61 (bs, 1H, NH), 11.41 (bs, 1H, NNH).

^{13}C NMR (126 MHz, DMSO-d_6): δ [ppm] = 153.2 (C-2''), 143.5 (CHN), 143.2 (C-4), 141.7 (C-2'), 137.5 (C-8a''), 136.2 (C-1), 130.9 (C-4'), 130.8 (C-1'), 129.7 (C-3), 128.1 (C-7''), 127.7 (C-3'), 127.4 (C-5'), 127.2 (C-2), 125.7 (C-5''), 125.2 (C-6'), 121.4 (C-6''), 118.2 (C-4a''), 113.6 (C-8''), 51.4 (C-4''), 21.1 (CH_3).

HRMS (+ESI): calc. for $C_{22}H_{21}N_4O_3S$ $[M+H]^+$: 421.1329; found: 421.1320.

IR (ATR): $\tilde{\nu}$ [cm^{-1}] = 3366 (w, NH), 2904 (w, C–H), 1658 (vs, C=O), 1600 (m, C=C), 1438 (s), 1323 (s, RSO_2N), 1167 (vs, RSO_2N), 960 (m), 810 (m), 766 (s, C–H), 750 (vs, C–H), 706 (m), 666 (s).

m.p. = >210 °C (decomposition).

*3.3.23. N'-(4-(2-Oxo-1,4-dihydroquinazolin-3(2H)-yl)butylidene)-4-methylbenzenesulfonohydrazide (**4aa**)*

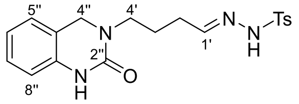

Following GP 3 starting from **SI-8aa**, compound **4aa** was obtained as a colorless solid (289 mg, 747 µmol, 75%).

***R*f:** 0.42 (CH_2Cl_2/ac = 7/3) [UV, $KMnO_4$].

^{1}H NMR (500 MHz, DMSO-d_6): δ [ppm] = 1.60 (*virt.* p, $^3J \approx {}^3J$ = 7.4 Hz, 2H, H-3'), 2.08 (td, ^3J = 7.4 Hz, ^3J = 5.1 Hz, 2H, H-2'), 2.84 (s, 3H, CH_3), 3.17 (t, ^3J = 7.4 Hz, 2H, H-4'), 4.31 (s, 2H, H-4''), 6.75 (dd, ^3J = 7.7 Hz, ^4J = 1.2 Hz, 1H, H-8''), 6.86 (*virt.* td, $^3J \approx {}^3J$ = 7.7 Hz, ^4J = 1.2 Hz, 1H, H-6''), 7.05 (dd, ^3J = 7.7 Hz, ^4J = 1.4 Hz, 1H, H-5''), 7.11 (*virt.* td, $^3J \approx {}^3J$ = 7.7 Hz, ^4J = 1.4 Hz, 1H, H-7''), 7.27 (t, ^3J = 5.1 Hz, 1H, H-1'), 7.36 – 7.40 (m, 2H, H-3), 7.66 – 7.71 (m, 2H, H-2), 9.13 (bs, 1H, NH), 10.93 (bs, 1H, NNH).

^{13}C NMR (126 MHz, DMSO-d_6): δ [ppm] = 153.6 (C-2''), 151.3 (C-1'), 143.3 (C-4), 137.8 (C-8a''), 136.2 (C-1), 129.6 (C-3), 127.8 (C-7''), 127.3 (C-2), 125.5 (C-5''), 121.0 (C-6''), 117.9 (C-4a''), 113.2 (C-8''), 47.8 (C-4''), 45.5 (C-4'), 29.3 (C-2'), 23.1 (C-3'), 21.1 (CH_3).

HRMS (+ESI): calc. for $C_{19}H_{23}N_4O_3S$ $[M+H]^+$: 387.1485; found: 387.1486.

IR (ATR): $\tilde{\nu}$ [cm^{-1}] = 3315 (w, N–H), 3260 (w, N–H), 2959 (w, C–H), 2922 (w, C–H), 2864 (w, C–H), 1652 (s, C=O), 1608 (m, C=C), 1518 (w, C=C), 1448 (m), 1326 (s, RSO_2N), 1166 (s, RSO_2N), 1065 (m), 813 (m), 751 (s, C–H), 705 (m, C–H).

m.p. = >141°C (decomposition).

*3.3.24. N'-(2-((2-Oxo-1,4-dihydroquinazolin-3(2H)-yl)methyl)benzylidene)-4-methylbenzenesulfonohydrazide (**4ab**)*

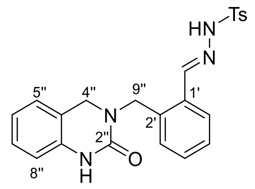

Following GP 3 starting from **SI-8ab**, compound **4ab** was obtained as a colorless solid (858 mg, 1.98 mmol, 91%).

***R*f**: 0.75 (CH_2Cl_2/ac = 7/3) [UV, $KMnO_4$].

^{1}H NMR (500 MHz, DMSO-*d*$_6$): δ [ppm] = 2.31 (s, 3H, CH_3), 4.22 (s, 2H, H-4’’), 4.57 (s, 2H, H-9‘‘), 6.81 (dd, ^3J = 7.6 Hz, ^4J = 1.2 Hz, 1H, H-8’’), 6.85 (*virt.* td, $^3J \approx {}^3J$ = 7.6 Hz, ^4J = 1.2 Hz, 1H, H-6’’), 6.99 (d, ^3J = 7.6 Hz, 1H, H-5’’), 7.13(*virt.* td, $^3J \approx {}^3J$ = 7.6 Hz, ^4J = 1.5 Hz, 1H, H-7’’), 7.26 (dd, ^3J = 7.6 Hz, ^4J = 1.5 Hz, 1H, H-3’), 7.31(*virt.* td, $^3J \approx {}^3J$ = 7.6 Hz, ^4J = 1.5 Hz, 1H, H-5’), 7.34 – 7.40 (m, 3H, H-3, H-4’), 7.58 (dd, ^3J = 7.6 Hz, ^4J = 1.5 Hz, 1H, H-6’), 7.75 – 7.79 (m, 2H, H-2), 8.22 (s, 1H, CHN), 9.35 (bs, 1H, NH), 11.49 (bs, 1H, NNH).

^{13}C NMR (126 MHz, DMSO-*d*$_6$): δ [ppm] = 153.5 (C-2’’), 146.4 (CHN), 143.6 (C-4), 137.6 (C-8a’’), 136.0 (C-1), 135.9 (C-2’), 131.7 (C-1’), 130.0 (C-4’), 129.7 (C-3), 128.0 (C-7’’), 127.9 (C-3’†), 127.9 (C-6’†), 127.5 (C-5’), 127.4 (C-2), 125.7 (C-5’’), 121.2 (C-6’’), 117.4 (C-4a’’), 113.4 (C-8’’), 47.8 (C-4’’), 47.2 (C-9’’), 21.1 (CH_3).

† assignment is interconvertible

HRMS (+ESI): calc. for $C_{23}H_{23}N_4O_3S$ $[M+H]^+$: 423.1485; found: 435.1484.

IR (ATR): $\tilde{\nu}$ [cm^{-1}] = 3316 (w, N–H), 3213 (w, N–H), 2903 (w, C–H), 1656 (s, C=O), 1606 (m, C=C), 1520 (w, C=C), 1511 (m, C=C), 1456 (m), 1322 (s, RSO_2N), 1165 (vs, RSO_2N), 1050 (m), 811 (m), 754 (vs, C–H), 703 (s, C–H).

m.p. = >146 °C (decomposition).

*3.3.25. N'-(3,3-Dimethyl-4-(2-oxo-1,4-dihydroquinazolin-3(2H)-yl)butylidene)-4-methylbenzenesulfonohydrazide (**4ac**)*

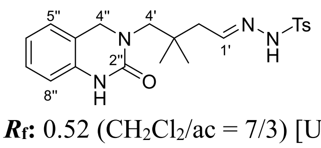

Following GP 3 starting from **SI-8ac**, compound **4ac** was obtained as a colorless solid (681 mg, 1.84 mmol, 75%).

***R*f:** 0.52 (CH_2Cl_2/ac = 7/3) [UV, $KMnO_4$].

^{1}H NMR (500 MHz, DMSO-*d*$_6$): δ [ppm] = 0.71 [s, 6H, $(CH_3)_2$], 1.96 (d, ^3J = 6.2 Hz, 2H, H-2’), 2.34 (s, 3H, CH_3), 3.00 (s, 2H, H-4’), 3.17 (t, ^3J = 7.4 Hz, 2H, H-4’), 4.29 (s, 2H, H-4’’), 6.78 (dd, ^3J = 7.7 Hz, ^4J = 1.2 Hz, 1H, H-8’’), 6.87 (*virt.* td, $^3J \approx {}^3J$ = 7.7 Hz, ^4J = 1.2 Hz, 1H, H-6’’), 7.05 (dd, ^3J = 7.7 Hz, ^4J = 1.4 Hz, 1H, H-5’’), 7.13 (*virt.* td, $^3J \approx {}^3J$ = 7.7 Hz, ^4J = 1.4 Hz, 1H, H-7’’), 7.33 (t, ^3J = 6.2 Hz, 1H, H-1’), 7.35 – 7.39 (m, 2H, H-3), 7.65 – 7.69 (m, 2H, H-2), 9.21 (bs, 1H, NH), 10.94 (bs, 1H, NNH).

^{13}**C NMR** (126 MHz, DMSO-d_6): δ [ppm] = 155.3 (C-2''), 150.5 (C-1'), 143.2 (C-4), 138.3 (C-8a''), 136.2 (C-1), 129.6 (C-3), 127.9 (C-7''), 127.3 (C-2), 125.2 (C-5''), 121.0 (C-6''), 118.8 (C-4a''), 113.2 (C-8''), 56.1 (C-4'), 50.7 (C-4''), 41.9 (C-2'), 36.4 (C-3'), 25.2 [$(CH_3)_2$], 21.0 (CH_3).

HRMS (+ESI): calc. for $C_{21}H_{27}N_4O_3S$ $[M+H]^+$: 415.1798; found: 415.1790.

IR (ATR): $\tilde{\nu}$ [cm^{-1}] = 3335 (w, N–H), 3202 (w, N–H), 2961 (w, C–H), 2912 (w, C–H), 2873 (w, C–H), 1652 (s, C=O), 1604 (m, C=C), 1451 (m), 1319 (s, RSO_2N), 1159 (vs, RSO_2N), 1036 (m), 813 (m), 751 (vs, C–H), 704 (s), 664 (vs).

m.p. = >130 °C (decomposition).

*3.3.26. N'-(2-(2-(2-Oxo-1,4-dihydroquinazolin-3(2H)-yl)ethyl)benzylidene)-4-methylbenzenesulfonohydrazide (**4ba**)*

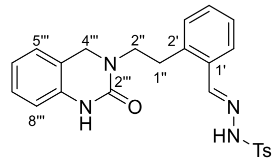

Following GP 3 starting from **SI-8ba**, compound **4ba** was obtained after purification by column chromatography (CH_2Cl_2/MeOH = 49/1) as a colorless solid (277 mg, 618 µmol, 35%).

***R*f**: 0.80 (CH_2Cl_2/ac = 7/3) [UV, $KMnO_4$].

^{1}**H NMR** (500 MHz, DMSO-d_6): δ [ppm] = 2.33 (s, 3H, CH_3), 2.94 – 2.97 (m, 2H, H-1''), 3.35 – 3.38 (m, 2H, H-2''), 4.36 (s, 2H, H-4'''), 6.77 (dd, ^3J = 7.7 Hz, ^4J = 1.2 Hz, 1H, H-8'''), 6.86 (*virt.* td, $^3J \approx {}^3J$ = 7.7 Hz. ^4J = 1.2 Hz, 1H, H-6'''), 7.03 (dd, ^3J = 7.7 Hz, ^4J = 1.5 Hz, 1H, H-5'''), 7.12 (*virt*, td, $^3J \approx {}^3J$ = 7.7 Hz, ^4J = 1.5 Hz, 1H, H-7'''), 2.21 – 2.76 (m, 2H, H-3', H-5'), 7.30 (*virt.* td, $^3J \approx {}^3J$ = 7.6 Hz, ^4J = 1.6 Hz, 1H, H-4'), 7.37 – 7.41 (m, 2H, H-3), 7.57 (dd, ^3J = 7.6 Hz, ^4J = 1.6 Hz, 1H, H-6'), 7.75 – 7.78 (m, 2H, H-2), 8.26 (s, 1H, CHN), 9.17 (s, 1H, NH), 11.46 (s, 1H, NNH).

^{13}**C NMR** (126 MHz, DMSO-d_6): δ [ppm] = 153.5 (C-2'''), 146.2 (CHN), 143.5 (C-4), 138.1 (C-2'), 137.8 (C-8a'''), 136.2 (C-1), 131.6 (C-1'), 130.8 (C-3'), 123.0 (C-4'), 129.8 (C-3), 127.8 (C-7'''), 127.3 (C-2), 126.9 (2C, C-5', C-6'), 125.4 (C-5'''), 121.1 (C-6'''), 117.9 (C-4a'''), 113.3 (C-8'''), 48.1 (C-4'''), 47.8 (C-1''), 23.0 (C-2''), 21.1 (CH_3).

HRMS (+ESI): calc. for $C_{24}H_{25}N_4O_5S$ $[M+H]^+$: 449.1642; found: 449.1635.

IR (ATR): $\tilde{\nu}$ [cm^{-1}] = 3339 (w, N–H), 3199 (w, N–H), 2986 (w, C–H), 2921 (w, C–H), 2879 (w, C–H), 1652 (s, C=O), 1606 (m, C=C), 1450 (m), 1323 (m, RSO_2N), 1160 (vs, RSO_2N), 1044 (m), 813 (m), 751 (vs, C–H), 704 (s), 664 (vs).

m.p. = >114 °C (decomposition).

*3.3.27. N'-((6-(2-(2-Oxo-1,4-dihydroquinazolin-3(2H)-yl)ethyl)benzo[d][1,3]dioxol-5-yl)methylene)-4-methylbenzenesulfonohydrazide (**4bb**)*

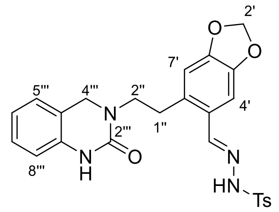

Following GP 3 starting from **SI-11bb**, compound **4bb** was obtained as a colorless solid (289 mg, 455 µmol, 77%).

***R*f:** 0.77 (CH_2Cl_2/ac = 7/3) [UV, $KMnO_4$].

^{1}H NMR (500 MHz, DMSO-d_6): δ [ppm] = 2.35 (s, 3H, CH_3), 2.82 – 2.88 (m, 2H, H-1''), 3.30 – 3.35* (m, 2H, H-2''), 4.37 (s, 2H, H-4'''), 6.01 (s, 2H, H-2'), 6.77 (dd, ^3J = 7.6 Hz, ^4J = 1.2 Hz, 1H, H-8'''), 6.79 (s, 1H, H-7'), 6.87 (*virt.* td, $^3J \approx {}^3J$ = 7.6 Hz, ^4J = 1.2 Hz, 1H, H-6'''), 7.04 (d, ^3J = 7.6 Hz, 1H, H-5'''), 7.06 (s, 1H, H-4'), 7.12 (*virt.* td, $^3J \approx {}^3J$ = 7.6 Hz, ^4J = 1.4 Hz, 1H, H-7'''), 7.37 – 7.42 (m, 2H, H-3), 7.73 – 7.79 (m, 2H, H-2), 8.17 (s, 1H, CHN), 9.16 (bs, 1H, NH), 11.28 (bs, 1H, NNH).

* signal overlaps with residual water.

^{13}C NMR (126 MHz, DMSO-d_6): δ [ppm] = 153.5 (C-2'''), 148.9 (C-7a'), 146.4 (C-3a'), 145.4(CHN), 143.5 (C-1), 137.7 (C-8a'''), 136.1 (C-1), 133.5 (C-6'), 129.7 (C-3), 127.8 (C-7'''), 127.3 (C-2), 125.4 (C-5'''), 125.2 (C-5'), 121.1 (C-6'''), 117.9 (C-4a'''), 113.3 (C-8'''), 110.2 (C-7'), 104.7 (C-4'), 101.5 (C-2'), 48.1 (C-4'''), 48.0 (C-2''), 29.5 (C-1''), 21.0 (CH_3).

HRMS (+ESI): calc. for $C_{25}H_{25}N_4O_5S$ $[M+H]^+$: 493.1540; found: 493.1537.

IR (ATR): $\tilde{\nu}$ [cm^{-1}] = 3347 (w, N–H), 2886 (w, C–H), 2842 (w, C–H), 1666 (m, C=O), 1606 (w, C=C), 1518 (w, C=C), 1458 (m), 1322 (m, RSO_2N), 1244 (m, C–O), 1157 (vs, RSO_2N), 1033 (s), 781 (w), 756 (m, C–H), 724 (w), 707 (m).

m.p. = >169 °C (decomposition).

*3.3.28. N'-(4,5-Dimethoxy-2-(2-(2-oxo-1,4-dihydroquinazolin-3(2H)-yl)ethyl)benzylidene)-4-methylbenzenesulfonohydrazide (**4bc**)*

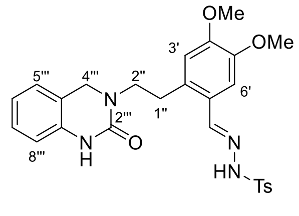

Following GP 3 starting from **SI-11bc**, compound **4bc** was obtained as a colorless solid (289 mg, 455 µmol, 77%).

***R*f:** 0.61 (CH_2Cl_2/ac = 7/3) [UV, $KMnO_4$].

^{1}H NMR (500 MHz, DMSO-d_6): δ [ppm] = 2.34 (s, 3H, CH_3), 2.85 – 2.91 (m, 2H, H-1''), 3.34 – 3.38* (m, 2H, H-2''), 3.63 (s, 3H, C-5'-OCH_3), 3.72 (s, 3H, C-4'-OCH_3), 4.33 (s, 2H, H-4'''), 6.73 (s, 1H, H-3'), 6.77 (dd, ^3J = 7.6 Hz, ^4J = 1.2 Hz, 1H, H-8'''), 6.86 (*virt.* td, $^3J \approx {}^3J$ = 7.6 Hz, ^4J = 1.2 Hz, 1H, H-6'''), 7.03 (dd, ^3J = 7.6 Hz, ^4J = 1.4 Hz, 1H, H-5'''), 7.09 (s, 1H, H-6'), 7.11 (*virt.* td, $^3J \approx {}^3J$ = 7.6 Hz, ^4J = 1.4 Hz, 1H, H-7'''), 7.38 – 7.42 (m, 2H, H-3), 7.75 – 7.80 (m, 2H, H-2), 8.16 (s, 1H, CHN), 9.15 (bs, 1H, NH), 11.22 (bs, 1H, NNH).

* signal overlaps with residual water.

^{13}C NMR (126 MHz, DMSO-d_6): δ [ppm] = 153.5 (C-2'''), 150.3 (C-5'), 147.3 (C-4'), 146.1 (CHN), 143.5 (C-4), 137.8 (C-8a'''), 136.1 (C-1), 131.6 (C-2'),129.6 (C-3), 127.8 (C-7'''), 127.4 (C-2), 125.4 (C-5'''), 123.7 (C-1'), 121.0 (C-6'''), 118.0 (C-4a'''), 113.5 (C-3'), 113.2 (C-8'''), 108.8 (C-6'), 55.4 (C-5'-O*C*H$_3$†), 55.3 (C-4'-O*C*H$_3$†), 48.0 (C-4''), 47.7 (C-2''), 29.2 (C-1''), 21.0 (CH$_3$).

† assignment is interconvertible

HRMS (+ESI): calc. for $C_{26}H_{29}N_4O_5S$ [M+H]$^+$: 509.1853; found: 509.1854.

IR (ATR): $\tilde{\nu}$ [cm^{-1}] = 3264 (w, H–H), 2936 (w, C–H), 2910 (w, C–H), 1653 (s, C=O), 1634 (m, C=N), 1609 (m, C=C), 1598 (m, C=C), 1510 (s, C=C), 1451 (m), 1323 (m, RSO$_2$N), 1266 (s, C–O), 1245 (m, C–O), 1165 (vs, RSO$_2$N), 1047 (m), 816 (m), 781 (w), 745 (s, C–H), 708 (s, C–H).

m.p. = >161 °C (decomposition).

*3.3.29. N'-(2-(2-Oxo-1,4-dihydroquinazolin-3(2H)-yl)ethylidene)-4-methylbenzenesulfonohydrazide (**4ca**)*

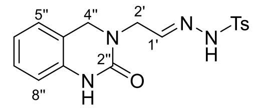

Following GP 3 starting from **SI-8ca**, compound **4ca** was obtained as a colorless solid (312 mg, 870 µmol, 87%).

***R*f**: 0.24 (CH$_2$Cl$_2$/ac = 7/3) [UV, KMnO$_4$].

^{1}H NMR (500 MHz, DMSO-d_6): δ [ppm] = 2.34 (s, 3H, CH$_3$), 3.94 (d, ^3J =4.7 Hz, 2H, H-2'), 4.07 (s, 2H, H-4''), 6.76 (dd, ^3J = 7.6 Hz, ^4J = 1.1 Hz, 1H, H-8''), 6.88 (*virt.* td, $^3J \approx {}^3J$ = 7.6 Hz, ^4J = 1.1 Hz, 1H, H-6''), 6.92 (dd, ^3J = 7.6 Hz, ^4J = 1.6 Hz, 1H, H-5''), 7.12(*virt.* td, $^3J \approx {}^3J$ = 7.6 Hz, ^4J = 1.6 Hz, 1H, H-7''), 7.17 (t, ^3J = 4.7 Hz, 1H, H-1'), 7.33 – 7.37 (m, 2H, H-3), 7.65 – 7.78 (m, 2H, H-2), 9.30 (bs, 1H, NH), 11.13 (bs, 1H, NNH).

^{13}C NMR (126 MHz, DMSO-d_6): δ [ppm] = 153.3 (C-2''), 146.2 (C-1'), 143.4 (C-4), 137.4 (C-8a''), 135.8 (C-1), 129.7 (C-3), 127.9 (C-7''), 127.3 (C-2), 125.5 (C-5''), 121.2 (C-6''), 117.5 (C-4a''), 113.4 (C-8''), 47.6 (C-4''), 47.0 (C-2'), 21.1 (CH$_3$).

HRMS (+ESI): calc. for $C_{17}H_{19}N_4O_3S$ [M+H]$^+$: 359.1172; found: 359.1172.

IR (ATR): $\tilde{\nu}$ [cm^{-1}] = 3200 (w, NH), 3127 (w, N–H), 2987 (w, C–H), 2918 (w, C–H), 2865 (w, C–H), 1664 (s, C=O), 1607 (m, C=C), 1521 (m, C=C), 1446 (m), 1309 (s, RSO$_2$N), 1165 (vs, RSO$_2$N), 1047 (s), 811 (m), 755 (s, C–H), 748 (s, C–H), 740 (s, C–H), 725 (s).

m.p. = >186 °C (decomposition).

3.4. Procedures for individual substrates

*3.4.1. N'-(2,2-Dimethyl-3-(2-oxo-1,4-dihydroquinazolin-3(2H)-yl)propylidene)-4-methylbenzenesulfonohydrazide (**4u**)*

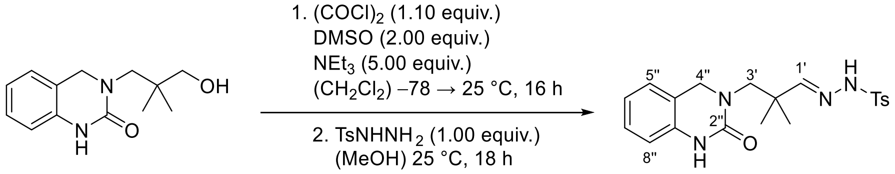

According to a modified procedure by *Omura et al.*,[14] DMSO (2.00 equiv.) was added dropwise to a solution of $(COCl)_2$ (1.10 equiv.) in CH_2Cl_2 (200 mM) at −78 °C. After 15 min, a solution of alcohol **SI-8u** (1.00 equiv.) in CH_2Cl_2 (1.0 M) was added dropwise and the reaction mixture was stirred for 30 min at −78 °C. Then, NEt_3 (5.00 equiv.) was added dropwise, and the mixture was allowed to warm to r.t. overnight (16 h). The reaction was quenched by addition of H_2O (3 mL/mmol) and diluted with CH_2Cl_2 (3 mL/mmol). The aqueous layer was extracted with CH_2Cl_2 (3 × 15 mL). The combined organic layers were washed with brine (20 mL) dried over Na_2SO_4, filtered and the solvent was removed under reduced pressure.

The crude product was redissolved in MeOH (1 M) and *p*-toluenesulfonyl hydrazide (1.00 equiv.) was added. The reaction mixture was stirred for 18 h at 20 °C. The precipitate was collected and washed with little amounts of Et_2O and cold MeOH to obtain the hydrazone **4u** (287 mg, 717 µmol, 56%).as colorless solids.

***R*f**: 0.73 (CH_2Cl_2/ac = 7/3) [UV, $KMnO_4$].

^{1}H NMR (500 MHz, DMSO-*d*6): δ [ppm] = 0.92 [s, 6H, $(CH_3)_2$], 2.18 (s, 3H, CH_3), 3.26 (s, 2H, H-3'), 3.99 (s, 2H, H-4''), 6.76 (d, ^3J = 7.9 Hz, 1H, H-8''), 6.84 – 6.90 (m, 2H, H-5'', H-6''), 7.10 – 7.15 (m, 1H, H-7''), 7.20 – 7.23 (m, 2H, H-3), 7.24 (s, 1H, H-1'), 7.61 – 7.65 (m, 2H, H-2), 9.11 (bs, 1H, NH), 10.85 (bs, 1H, NNH).

^{13}C NMR (126 MHz, DMSO-*d*6): δ [ppm] = 157.0 (C-1'), 154.4 (C-2''), 143.2 (C-4), 137.8 (C-8a''), 136.0 (C-1), 129.4 (C-3), 127.8 (C-7''), 127.1 (C-2), 125.3 (C-5''), 121.0 (C-6''), 118.1 (C-4a''), 113.1 (C-8''), 55.0 (C-3'), 50.1 (C-4''), 48.7 (C-2'), 23.2 [$(CH_3)_2$], 20.9 (CH_3).

HRMS (+ESI): calc. for $C_{20}H_{25}N_4O_4S$ $[M+H]^+$: 401.1642; found: 401.1637.

IR (ATR): $\tilde{\nu}$ [cm^{-1}] = 3338 (w, N–H), 3199 (w, N–H), 2970 (w, C–H), 2920 (w, C–H), 2869 (w, C–H), 1651 (m, C=O), 1605 (m, C=C), 1448 (m), 1319 (m, RSO_2N), 1159 (vs, RSO_2N), 1019 (m), 813 (m), 751 (s, C–H), 705 (s).

m.p. = >121 °C (decomposition).

*3.4.2. 2-(1,3-Dioxolan-2-yl)benzonitrile (**SI-9**)*

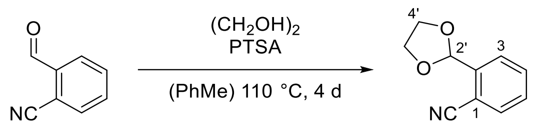

According to a modified procedure by *Lu et al.*,[15] PTSA (39.9 mg, 210 µmol, 1.0 mol%.) was added to a solution of 2-formylbenzonitrile (2.75 g, 21.0 mmol, 1.00 equiv.) and ethylene glycol (5.87 mL, 105 mmol, 5.00 equiv.) in PhMe (47 mL, 450 mM), and the reaction mixture was heated at 110 °C with a *Dean-Stark*-apparatus for 4 d. After cooling to room temperature, the mixture was diluted with Et_2O (50 mL). The organic layer was washed with sat $NaHCO_3$ solution (50 mL), dried over Na_2SO_4, filtered, and the solvent was removed under reduced pressure to obtain the acetal **SI-9** (2.96 g, 16.9 mmol, 80%) as a colorless oil.

^{1}H NMR (500 MHz, $CDCl_3$): δ [ppm] = 4.06 – 4.13 (m, 2H, H^a-4', H^a-5'), 4.22 – 4.28 (m, 2H, H^b-4', H^b-5'), 5.98 (s, 1H, H-2'), 7.47 (ddd, ^3J = 7.6 Hz, ^3J = 6.9 Hz, ^4J = 1.9 Hz, 1H, H-5), 7.58 – 7.64 (m, 2H, H-3, H-4), 7.71 (*virt.* dt, ^3J = 7.6 Hz, $^4J \approx {}^5J$ = 1.0 Hz, 1H, H-6).

^{13}C NMR (126 MHz, MeOD): δ [ppm] = 141.2 (C-, 133.8 (C-6), 132.7 (C-2), 129.9 (C-5), 128.1 (C-, 117.3 (CN), 111.4 (C-1), 102.1 (C-2'), 66.1 (C-4', C-5').

Spectral data matched those reported in the literature.[15]

*3.4.3. (2-(1,3-Dioxolan-2-yl)phenyl)methanamine (**SI-10**)*

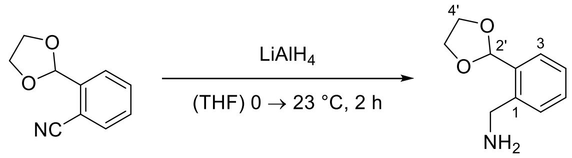

According to a modified procedure by *Iermolenko et al.*,[12] nitrile **SI-9** (1.23 g, 7.00 mmol, 1.00 equiv.) in THF (3.5 mL) was added dropwise to a suspension of $LiAlH_4$ (531 mg, 14.0 mmol, 2.00 equiv.) in THF (35 mL), and the reaction mixture was warmed to room temperature. After 2 h, the reaction was quenched by addition of H_2O (0.15 mL), followed by addition of aq. NaOH (1 M, 0.34 mL) and H_2O again (0.43 mL). The solids were filtered off and washed with EtOAc (3 × 50 mL). The solvents were removed under reduced pressure and the crude amine **SI-10** was directly used for the reductive amination.

*3.4.4. 6-(2-(2-Oxo-1,4-dihydroquinazolin-3(2H)-yl)ethyl)benzo[d][1,3]dioxole-5-carbaldehyde (**SI-11bb**)*

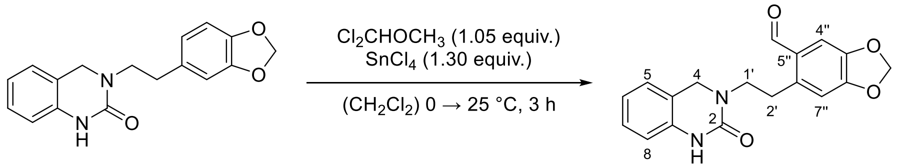

According to a modified procedure by *Cueva et al.*,[16] $SnCl_4$ (0.18 mL, 408 mg, 1.57 mmol, 1.20 equiv.) was added to a solution of quinazolinone **SI-8bb** (387 mg, 1.30 mmol, 1.00 equiv.) and Cl_2CHOCH_3 (0.17 ml, 224 mg, 1.96 mmol, 1.50 equiv.) in CH_2Cl_2 (5.2 ml, 250 mM) at 0 °C. The reaction mixture was kept at this temperature for further 15 min and then stirred at 25 °C for 3 h. The reaction was quenched by pouring onto ice and the aqueous layer was extracted with CH_2Cl_2 (3 × 50 mL). The combined organic layers were dried over Na_2SO_4, filtered and the solvent was removed under reduced pressure. The crude product was directly subjected to hydrazone formation without further purification.

*3.4.5. 4,5-Dimethoxy-2-(2-(2-oxo-1,4-dihydroquinazolin-3(2H)-yl)ethyl)benzaldehyde (**SI-11bc**)*

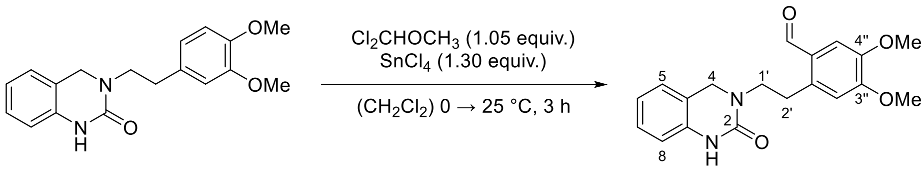

According to a modified procedure by *Cueva et al.*,[16] $SnCl_4$ (0.24 mL, 542 mg, 2.08 mmol, 1.30 equiv.) was added to a solution of quinazolinone **SI-8bc** (500 mg, 1.60 mmol, 1.00 equiv.) and Cl_2CHOCH_3 (0.15 ml, 193 mg, 1.68 mmol, 1.05 equiv.) in CH_2Cl_2 (6.4 ml, 250 mM) at 0 °C. The reaction mixture was kept at this temperature for further 15 min and then stirred at 25 °C for 3 h. The reaction was quenched by pouring onto ice and the aqueous layer was extracted with CH_2Cl_2 (3 × 50 mL). The combined organic layers were dried over Na_2SO_4, filtered and the solvent was removed under reduced pressure. The crude product was directly subjected to hydrazone formation without further purification.

*3.4.6. 6-(2-(2-Oxo-1,4-dihydroquinazolin-3(2H)-yl)ethyl)benzo[d][1,3]dioxole-5-carbaldehyde (**SI-12w**)*

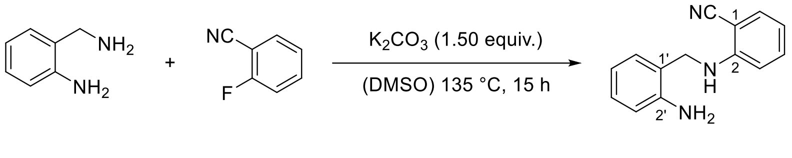

According to a modified procedure by *Gruber et al.*,[17] K_2CO_3 (1.04 g, 7.50 mmol, 1.50 equiv.) was added to a solution of 2-aminobenzylamine (611 mg, 5.00 mmol, 1.00 equiv.) and 2-fluorobenzonitrile (0.54 ml, 606 mg, 5.00 mmol, 1.00 equiv.) in DMSO (1.25 ml, 4 M), and the reaction mixture was stirred at 135 °C for 15 h. After cooling to room temperature, the reaction was quenched by addition of H_2O (25 mL). The aqueous layer was extracted with CH_2Cl_2 (3 × 30 mL). The combined organic layers were dried over Na_2SO_4, filtered and the solvent was removed under reduced pressure. The crude diamine **SI-12w** was directly subjected to the cyclization protocol without purification or analyzation.

*3.4.7. 2-(2-Oxo-1,4-dihydroquinazolin-3(2H)-yl)benzaldehyde (**SI-13w**)*

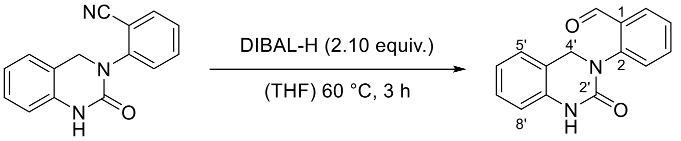

According to a modified procedure by *Zhang et al.*,[18] a solution of DIBAL-H (1 M in THF, 8.40 mL, 8.40 mmol, 2.10 equiv.) was added to a solution of **SI-8w** (997 mg, 4.00 mmol, 1.00 equiv.) in THF (12 ml, 0.3 M), and the reaction mixture was stirred at 60 °C for 3 h. After cooling to room temperature, the reaction was quenched by addition of sat. *Rochelle* salt solution (20 mL). The aqueous layer was extracted with CH_2Cl_2 (3 × 15 mL). The combined organic layers were dried over Na_2SO_4, filtered and the solvent was removed under reduced pressure. The crude aldehyde **SI-13w** was directly subjected to the hydrazone formation without purification or analyzation.

*3.4.8. 2,3-Dihydro-5H-oxazolo[2,3-b]quinazoline (**9ca**)*

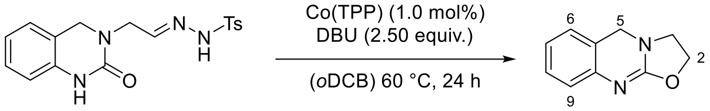

The hydrazone **4ca** (35.8 mg, 100 µmol, 1.00 equiv.) and Co(TPP) (672 µg, 1.00 µmol, 1.0 mol%) were added to a Schlenk tube and the atmosphere was exchanged (3×). The solids were dissolved in *o*DCB (5.0 mL) and DBU (0.04 mL, 38.0 mg, 250 µmol, 2.50 equiv.) was added. The tube was sealed and stirred at 60 °C for 24 h. After cooling the room temperature, the mixture was subjected to flash column chromatography (CH_2Cl_2/ac = 9/1 → 0/1) to yield product **9ca** (11.5 mg, 66.0 µmol, 66%) as a white solid.

***R*f**: 0.12 (CH_2Cl_2/ac = 4/1) [UV, $KMnO_4$].

^{1}H NMR (500 MHz, CD_2Cl_2): δ [ppm] = 3.50 – 3.53 (m, 2H, H-3), 4.40 – 4.43 (m, 2H, H-2), 4.53 (s, 2H, H-5), 6.90 – 6.94 (m, 1H, H-7, H-9), 6.96 (ddt, ^3J = 7.6 Hz, ^4J = 1.6 Hz, ^4J = 0.8 Hz, 1H, H-6), 7.10 – 7.14 (m, 1H, H-8).

^{13}C NMR (126 MHz, CD_2Cl_2): δ [ppm] = 160.8 (C-10a), 144.8 (C-9a), 128.7 (C-8), 125.9 (C-6), 123.9 (C-7), 123.1 (C-9), 119.6 (C-5a), 64.7 (C-2), 49.1 (C-5), 48.7 (C-3).

HRMS (+ESI): calc. for $C_{10}H_{11}N_2O$ $[M+H]^+$: 175.0866; found: 175.0865.

IR (ATR): $\tilde{\nu}$ [cm^{-1}] = 3330 (w), 1639 (vs, C=O), 1601 (s, C=C), 1573 (w, C=C), 1505 (w, C=C), 1482 (s), 1449 (m), 1277 (m), 1233 (w), 1146 (m, C–O), 1018 (m), 825 (w), 745 (vs, C–H), 707 (s), 692 (s), 664 (m).

m.p. = >230 °C.

4. Optimization of the Reaction Conditions with Hydrazone 4a

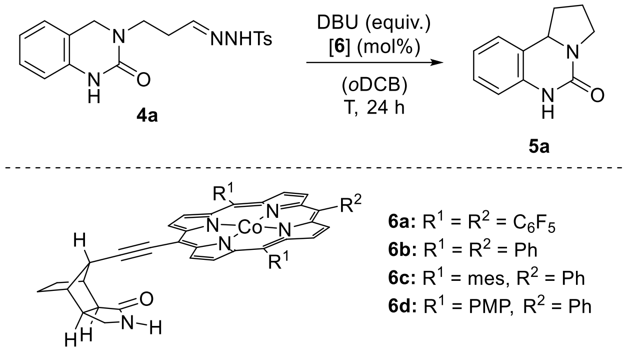

The reaction was performed under the indicated conditions and were initiated by addition of the solvent or the base, when the latter is liquid, to the substrate (100 µmol) and the indicated catalyst. The reaction was terminated after the indicated time by evaporation of the solvent. All yields refer to isolated material.

#	**6**	mol%	equiv.	T [°C]	**5a** [%]	*ee* [%]
1	Co(TPP)	1.0	2.50	60	85	—
2	**a**	1.0	2.50	60	82	81
3	**a**	1.0	2.50	50	36	87
4	**a**	1.0	2.50	40	—	—
5	**a**	2.0	2.50	60	66	81
6	**b**	1.0	2.50	60	72	92
7	**c**	1.0	2.50	60	77	69
8	**d**	1.0	2.50	60	73	86
9	**b**	0.5	2.50	60	99	84
10	**b**	1.0	1.50	60	93	87
11	**b**	1.0	1.50[1]	60	—	—
12	**b**	1.0	1.50	50	51	92
13	**b**	1.0	2.00	60	88	95
14	**b**	1.0	2.00	55	36	97

[1] Cs_2CO_3 was used as base.

5. Enantioselective Catalysis

5.1. General procedure 4 (GP 4): Cobalt-catalyzed Cyclization

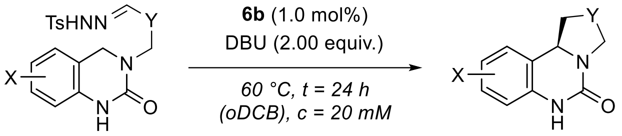

The corresponding hydrazone (100 µmol, 1.00 equiv.) and chiral cobalt catalyst **6b** (769 µg, 1.00 µmol, 1.0 mol%) were added to an oven-dried *Schlenk* tube and the atmosphere was exchanged (3×) to argon. The solids were dissolved in *o*DCB (5.0 mL, 20 mM) to yield a bright red solution. DBU (29.9 µL, 30.5 mg, 200 µmol, 2.00 equiv.) was added dropwise (about five drops), upon which the solution turned from red to copper. The tube was sealed, and the reaction mixture was heated to 60 °C for 24 h. After cooling to room temperature, the crude mixture was subjected to flash column chromatography (CH_2Cl_2/ac = 9/1 → 1/9) to yield cyclization products.

*5.1.1. 2,3,6,10b-Tetrahydropyrrolo[1,2-c]quinazolin-5(1H)-one (**5a**)*

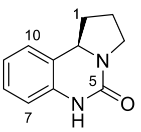

Following GP 4, compound **5a** was obtained as a colorless solid (16.6 mg, 88.2 µmol, 88%).

Scale-up Experiment: A Schlenk flask was equipped with hydrazone **4a** (372 mg, 1.00 mmol, 1.00 equiv.) and chiral catalyst **6b** (7.69 mg, 10.0 µmol, 1.0 mol%), and the atmosphere was exchanged (3×). The solids were dissolved in *o*DCB (50 mL) and DBU (0.30 mL, 304 mg, 2.00 mmol, 2.00 equiv.) was added dropwise to the reaction mixture. The flask was sealed and stirred at 60 °C for 48 h. After cooling to room temperature, the mixture was directly submitted to flash column chromatography (CH_2Cl_2/ac = 9/1 → 1/1) to yield product **4a** (175 mg, 932 µmol, 93%, 95% *ee*) as a colorless solid.

One-pot Experiment: A Schlenk tube was equipped with aldehyde **SI-8a** (20.4 mg, 100 µmol, 1.00 equiv.), $TsNHNH_2$ (22.4 mg, 120 µmol, 1.20 equiv.), and chiral catalyst **6b** (769 µg, 1.00 µmol, 1.0 mol%), and the atmosphere was exchanged (3×). The solids were dissolved in *o*DCB (5 mL) and DBU (0.03 mL, 30.5 mg, 200 µmol, 2.00 equiv.) was added dropwise to the reaction mixture. The tube was sealed and stirred at 60 °C for 24 h. After cooling to room temperature, the mixture was directly submitted to flash column chromatography (CH_2Cl_2/ac = 9/1 → 1/1) to yield product **4a** (15.0 mg, 80 µmol, 80%, 89% *ee*) as a colorless solid.

***R*f**: 0.14 (Hex/EtOAc = 1/4) [UV, $KMnO_4$].

^{1}H NMR (500 MHz, $CDCl_3$): δ [ppm] = 1.19 – 2.14 (m, 3H, H^a-1, H^a-2, H^b-2), 2.54 – 2.60 (m, 1H, H^b-1), 3.60 (ddd, ^2J = 11.1 Hz, ^3J = 9.1 Hz, ^3J = 3.1 Hz, 1H, H^a-3), 3.65 (ddd, ^2J = 11.1 Hz, ^3J = 8.8 Hz, ^3J = 7.6 Hz, 1H, H^b-3), 4.64 (dd, ^3J = 10.0 Hz, ^3J = 5.5 Hz, 1H, H-10b), 6.70 (dd, ^3J = 7.9 Hz, ^4J = 1.2 Hz, 1H, H-7), 6.91 (bs, 1H, NH), 6.97 (*virt.* td, $^3J \approx {}^3J$ = 7.6 Hz, ^4J = 1.2 Hz, 1H, H-9), 7.05 (*virt.* dt, ^3J = 7.6 Hz, $^4J \approx {}^4J$ = 1.2 Hz, 1H, H-10), 7.15 – 7.20 (m, 1H, H-8).

^{13}C NMR (126 MHz, $CDCl_3$): δ [ppm] = 153.3 (C-5), 137.2 (C-6a), 128.3 (C-8), 124.9 (C-10), 122.2 (C-9), 122.0 (C-10a), 113.6 (C-7), 58.1 (C-10b), 44.6 (C-3), 31.8 (C-1), 22.9 (C-2).

HRMS (+ESI): calc. for $C_{11}H_{13}N_2O$ $[M+H]^+$: 189.1022; found: 189.1020.

m.p. = 144-146 °C.

Specific rotation: α_D^{25}: 34.1 (c = 1.0 $CHCl_3$) [95% *ee*].

Chiral HPLC: 95% ee [©CHIRALPAK IC, 20 °C, 50% ^{i}PrOH/*n*-heptane, 1mL/min, 210 nm, t_R = 13.7 min (major), 15.2 min (minor)].

*5.1.2. 10-Bromo-2,3,6,10b-tetrahydropyrrolo[1,2-c]quinazolin-5(1H)-one (**5b**)*

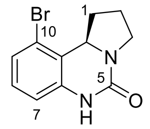

Following GP 4, compound **5b** was obtained as a colorless solid (19.3 mg, 72.3 µmol, 72%).

***R*f**: 0.18 (CH_2Cl_2/ac = 4/1) [UV, $KMnO_4$].

^{1}H NMR (500 MHz, $CDCl_3$): δ [ppm] = 1.66 (*virt.* ddt, ^2J = 12.3 Hz, ^3J = 10.9 Hz, $^3J \approx {}^3J$ = 9.8 Hz, 1H, H^a-1), 1.81 – 1.97 (m, 2H, H^a-2, H^b-2), 2.95 (dddd, ^2J = 12.3 Hz, ^3J = 7.7 Hz, ^3J = 5.3 Hz, ^3J = 2.4 Hz, 1H, H^b-1), 3.23 (ddd, ^2J = 11.7 Hz, ^3J = 9.6 Hz, ^3J = 5.3 Hz, 1H, H^a-3), 4.16 (ddd, ^2J = 11.7 Hz, ^3J = 8.9 Hz, ^3J = 6.6 Hz, 1H, H^b-3), 4.59 (dd, ^3J = 10.9 Hz, ^3J = 5.3 Hz, 1H, H-10b), 6.62 (dd, ^3J = 8.0 Hz, ^4J = 1.1 Hz, 1H, H-7), 7.01 (*virt.* t, $^3J \approx {}^3J$ = 8.0 Hz, 1H, H-8), 7.14 (dd, ^3J = 8.0 Hz, ^4J = 1.1 Hz, 1H, H-9), 7.83 (bs, 1H, NH).

^{13}C NMR (126 MHz, $CDCl_3$): δ [ppm] = 152.3 (C-5), 137.5 (C-6a), 129.6 (C-8), 126.6 (C-9), 122.0 (C-10), 120.1 (C-10a), 113.3 (C-7), 60.9 (C-10b), 44.7 (C-3), 33.1 (C-1), 21.3 (C-2).

HRMS (+ESI): calc. for $C_{11}H_{12}BrN_2O$ $[M+H]^+$: 267.0128; found: 267.0126.

IR (ATR): $\tilde{\nu}$ [cm^{-1}] = 3189 (w, N–H), 2963 (w, C–H), 2896 (w, C–H), 1661 (vs, C=O), 1603 (w, C=C), 1584 (m, C=C), 1445 (m), 1423 (s), 1074 (w, C–Br), 801 (m), 771 (m, C–H), 742 (m, C–H).

m.p. = 228-230 °C.

Specific rotation: α_D^{25}: 145.8 (c = 1.0 $CHCl_3$) [>99% *ee*].

Chiral HPLC: 91% ee [©CHIRALPAK IC, 20 °C, 30% ^{i}PrOH/*n*-heptane, 1mL/min, 210 nm, t_R = 11.8 min (major), 17.8 min (minor)].

*5.1.3. 9-Bromo-2,3,6,10b-tetrahydropyrrolo[1,2-c]quinazolin-5(1H)-one (**5c**)*

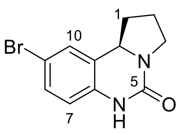

Following GP 4, compound **5c** was obtained as a colorless solid (22.2 mg, 83.5 µmol, 83%).

***R*f**: 0.35 (CH_2Cl_2/ac = 4/1) [UV, $KMnO_4$].

^{1}H NMR (500 MHz, $CDCl_3$): δ [ppm] = 1.92 – 2.10 (m, 2H, H^a-1, H^a-2), 2.11 – 2.18 (m, 1H, H^b-2), 2.54 – 2.60 (m, 1H, H^b-1), 3.59 – 3.70 (m, H^a-3, H^b-3), 4.64 (dd, ^3J = 10.1 Hz, ^3J = 5.5 Hz, 1H, H-10b), 6.62 (d, ^3J = 8.5 Hz, 1H, H-7), 6.99 (bs, 1H, NH), 7.19 (dd, ^4J = 2.0 Hz, ^4J = 0.8 Hz, 1H, H-10), 7.31 (ddd, ^3J = 8.5 Hz, ^4J= 2.0 Hz, ^6J = 0.8 Hz, 1H, H-8).

^{13}C NMR (126 MHz, $CDCl_3$): δ [ppm] = 153.2 (C-5), 136.5 (C-6a), 131.3 (C-8), 127.9 (C-10), 124.0 (C-9), 115.6 (C-7), 114.4 (C-10a), 57.8 (C-10b), 44.7 (C-3), 31.8 (C-1), 22.9 (C-2).

HRMS (+ESI): calc. for $C_{11}H_{12}BrN_2O$ $[M+H]^+$: 267.0128; found: 267.0127.

IR (ATR): $\tilde{\nu}$ [cm^{-1}] = 3189 (w, N–H), 2926 (w, C–H), 2886 (w, C–H), 1662 (vs, C=O), 1591 (m, C=C), 1439 (vs), 1399 (s), 1264 (s), 1076 (m, C–Br), 813 (vs), 790 (m, C–H), 747 (s, C–H).

m.p. = 208-210 °C.

Specific rotation: α_D^{25}: 41.7 (c = 1.0 $CHCl_3$) [94% *ee*].

Chiral HPLC: 94% ee [©CHIRALPAK AD-H, 20 °C, 10% ^{i}PrOH/*n*-heptane, 1mL/min, 210 nm, t_R = 15.9 min (major), 21.1 min (minor)].

*5.1.4. 8-Bromo-2,3,6,10b-tetrahydropyrrolo[1,2-c]quinazolin-5(1H)-one (**5d**)*

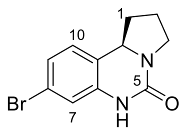

Following GP 4, compound **5d** was obtained as a colorless solid (22.7 mg, 85.0 µmol, 85%).

***R*f**: 0.31 (CH_2Cl_2/ac = 4/1) [UV, $KMnO_4$].

^{1}H NMR (500 MHz, $CDCl_3$): δ [ppm] = 1.92 (*virt.* tdd, $^2J \approx {}^3J$ = 11.8 Hz, ^3J = 10.2 Hz, ^3J = 7.6 Hz, 1H, H^a-1), 2.01 (*virt.* ttd, $^2J \approx {}^3J$ = 11.8 Hz, $^3J \approx {}^3J$ = 9.0 Hz, ^3J = 5.9 Hz, 1H, H^a-2), 2.11 (*virt.* dtdd, ^2J = 11.8 Hz, $^3J \approx {}^3J$ = 7.6 Hz, ^3J = 2.9 Hz, ^3J = 1.4 Hz, 1H, H^b-2), 2.55 (*virt.* dtd, ^2J = 11.8 Hz, $^3J \approx {}^3J$ = 5.9 Hz, ^3J = 1.4 Hz, 1H, H^b-1), 3.59 (ddd, ^2J = 11.1 Hz, ^3J = 9.0 Hz, ^3J = 2.9 Hz, 1H, H^a-3), 3.65 (ddd, ^2J = 11.1 Hz, ^3J = 9.0 Hz, ^3J = 7.6 Hz, 1H, H^b-3), 4.58 (dd, ^3J = 10.2 Hz, ^3J = 5.9 Hz, 1H, H-10b), 6.88 (d, ^4J = 1.9 Hz, 1H, H-7), 6.90 (dd, ^3J = 8.1 Hz, ^4J = 1.2 Hz, 1H, H-10), 7.09 (dd, ^3J = 8.1 Hz, ^4J= 1.9 Hz, 1H, H-9), 7.11 (bs, 1H, NH).

^{13}C NMR (126 MHz, $CDCl_3$): δ [ppm] = 152.8 (C-5), 138.7 (C-6a), 126.4 (C-10), 125.2 (C-9), 121.8 (C-8), 121.1 (C-10a), 116.7 (C-7), 57.9 (C-10b), 44.7 (C-3), 31.9 (C-1), 22.9 (C-2).

HRMS (+ESI): calc. for $C_{11}H_{12}BrN_2O$ $[M+H]^+$: 267.0128; found: 267.0128.

IR (ATR): $\tilde{\nu}$ [cm^{-1}] = 3330 (w, N–H), 2974 (w, C–H), 2946 (w, C–H), 2885 (w, C–H), 1666 (vs, C=O), 1593 (s, C=C), 1503 (w, C=C), 1437 (vs), 1383 (s), 1276 (s), 1071 (m, C–Br), 860 (vs), 761 (vs, C–H), 751 (s, C–H), 715 (s).

m.p. = >230 °C.

Specific rotation: α_D^{25}: 17.9 (c = 1.0 $CHCl_3$) [89% *ee*].

Chiral HPLC: 89% ee [©CHIRALPAK AD-H, 20 °C, 10% ^{i}PrOH/*n*-heptane, 1mL/min, 210 nm, t_R = 20.4 min (minor), 23.0 min (major)].

*5.1.5. 7-Bromo-2,3,6,10b-tetrahydropyrrolo[1,2-c]quinazolin-5(1H)-one (**5e**)*

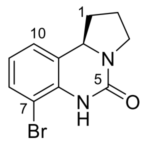

Following GP 4, compound **5e** was obtained as a colorless solid (22.4 mg, 83.9 µmol, 83%).

***R*f**: 0.15 (CH_2Cl_2/ac = 4/1) [UV, $KMnO_4$].

^{1}H NMR (500 MHz, $CDCl_3$): δ [ppm] = 1.91 – 2.07 (m, 2H, H^a-1, H^a-2), 2.09 – 2.17 (m, 1H, H^b-2), 2.54 – 2.60 (m, 1H, H^b-1), 3.58 – 3.69 (m, 2H, H^a-3, H^b-3), 4.67 (dd, ^3J = 10.1 Hz, ^3J = 5.5 Hz, 1H, H-11a), 6.85 (*virt.* t, $^3J \approx {}^3J$ = 7.8 Hz, 1H, H-9), 6.94 (bs, 1H, NH), 7.00 (*virt.* dq, ^3J = 7.8 Hz, $^4J \approx {}^4J \approx {}^5J$ = 1.1 Hz, 1H, H-10), 7.40 (*virt.* dt, ^3J = 7.7 Hz, $^4J \approx {}^6J$ = 1.1 Hz, 1H, H-8).

^{13}C NMR (126 MHz, $CDCl_3$): δ [ppm] = 152.3 (C-5), 135.5 (C-6a), 131.7 (C-8), 124.2 (C-10), 123.7 (C-10a), 123.2 (C-9), 108.3 (C-7), 58.4 (C-10b), 44.7 (C-3), 31.9 (C-1), 23.1 (C-2).

HRMS (+ESI): calc. for $C_{11}H_{12}BrN_2O$ $[M+H]^+$: 267.0128; found: 267.0127.

IR (ATR): $\tilde{\nu}$ [cm^{-1}] = 3214 (w, N–H), 2969 (w, C–H), 2883 (w, C–H), 1660 (vs, C=O), 1604 (m, C=C), 1432 (s), 1341 (m), 1016 (w, C–Br), 753 (vs, C–H), 725 (s, C–H), 708 (m).

m.p. = 131-133 °C.

Specific rotation: α_D^{25}: 4.0 (c = 1.0 $CHCl_3$) [47% *ee*].

Chiral HPLC: 47% ee [©CHIRALPAK AD-H, 20 °C, 10% ^{i}PrOH/*n*-heptane, 1mL/min, 210 nm, t_R = 17.7 min (major), 15.8 min (minor)].

*5.1.6. 9-Chloro-2,3,6,10b-tetrahydropyrrolo[1,2-c]quinazolin-5(1H)-one (**5f**)*

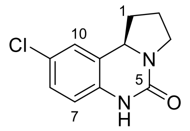

Following GP 4, compound **5f** was obtained as a colorless solid (16.2 mg, 72.8 µmol, 73%).

***R*f**: 0.32 (CH_2Cl_2/ac = 4/1) [UV, $KMnO_4$].

^{1}H NMR (500 MHz, $CDCl_3$): δ [ppm] = 1.85 – 2.18 (m, 3H, H^a-1, H^a-2, H^b-2), 2.54 (ddd, ^2J = 10.9 Hz, ^3J = 5.5 Hz, ^3J = 3.8 Hz, 1H, H^b-1), 3.54 – 3.71 (m, H^a-3, H^b-3), 4.62 (dd, ^3J = 9.9 Hz, ^3J = 5.5 Hz, 1H, H-10b), 6.68 (d, ^3J = 8.5 Hz, 1H, H-7), 7.01 (dd, ^4J = 2.3 Hz, ^4J = 1.1 Hz, 1H, H-10), 7.13 (ddd, ^3J = 8.5 Hz, ^4J= 2.3 Hz, ^6J = 0.8 Hz, 1H, H-9), 7.57 (bs, 1H, NH).

^{13}C NMR (126 MHz, $CDCl_3$): δ [ppm] = 153.0 (C-5), 136.1 (C-6a), 128.4 (C-8), 127.1 (C-9), 125.1 (C-10), 123.7 (C-10a), 115.0 (C-7), 57.9 (C-10b), 44.7 (C-3), 31.8 (C-1), 22.9 (C-2).

HRMS (+ESI): calc. for $C_{11}H_{12}BrN_2O$ $[M+H]^+$: 267.0128; found: 267.0127.

IR (ATR): $\tilde{\nu}$ [cm^{-1}] = 3190 (w, N–H), 2926 (w, C–H), 2884 (w, C–H), 1674 (vs), 1664 (vs, C=O), 1595 (m, C=C), 1441 (vs), 1281 (s), 1085 (m, C–Cl), 816 (s), 780 (m), 747 (s, C–H), 701 (m).

m.p. = 183-185 °C.

Specific rotation: α_D^{25}: 72.2 (c = 1.0 $CHCl_3$) [95% *ee*].

Chiral HPLC: 95% ee [©CHIRALPAK AD-H, 20 °C, 10% ^{i}PrOH/*n*-heptane, 1mL/min, 210 nm, t_R = 15.6 min (major), 21.1 min (minor)].

*5.1.7. 8-Fluoro-2,3,6,10b-tetrahydropyrrolo[1,2-c]quinazolin-5(1H)-one (**5g**)*

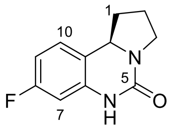

Following GP 4, compound **5g** was obtained as a colorless solid (18.9 mg, 91.7 µmol, 92%).

***R*f**: 0.37 (CH_2Cl_2/ac = 4/1) [UV, $KMnO_4$].

^{1}H NMR (500 MHz, $CDCl_3$): δ [ppm] = 1.92 (*virt.* tdd, $^2J \approx {}^3J$ = 11.5 Hz, ^3J = 10.2 Hz, ^3J = 7.4 Hz, 1H, H^a-1), 1.97 – 2.07 (m, 1H, H^a-2), 2.11 (*virt.* dtdd, ^2J = 11.8 Hz, $^3J \approx {}^3J$ = 7.4 Hz, ^3J = 2.9 Hz, ^3J = 1.4 Hz, 1H, H^b-2), 2.52 – 2.58 (m, 1H, H^b-1), 3.56 – 3.69 (m, 2H, H^a-3, H^b-3), 4.59 (*virt.* ddt, ^3J = 10.2 Hz, ^3J = 5.3 Hz, $^4J \approx {}^5J$ = 1.2 Hz, 1H, H-10b), 6.46 (dd, $^3J_{H–F}$ = 9.0 Hz, $^4J_{H–H}$ = 2.5 Hz, 1H, H-7), 6.66 (*virt.* td, $^3J_{H–F} \approx {}^3J_{H–H}$ = 9.0 Hz, $^4J_{H–H}$ = 2.5 Hz, 1H, H-9), 6.98 (ddd, $^3J_{H–H}$ = 9.0 Hz, $^4J_{H–F}$ = 5.8 Hz, $^4J_{H–H}$ = 1.2 Hz, 1H, H-10), 7.25 (s, 1H, NH).

^{13}C NMR (126 MHz, $CDCl_3$): δ [ppm] = 162.7 (d, $^1J_{C–F}$ = 245.0 Hz, C-8), 152.9 (C-5), 138.9 (d, $^3J_{C–F}$ = 10.9 Hz, C-6a), 126.3 (d, $^3J_{C–F}$ = 9.9 Hz, C-10), 118.0 (d, $^4J_{C–F}$ = 3.0 Hz, C-10a), 108.9 (d, $^2J_{C–F}$ = 22.0 Hz, C-9), 101.2 (d, $^2J_{C–F}$ = 25.6 Hz, C-7), 57.8 (C-10b), 44.7 (C-3), 32.1 (C-1), 22.9 (C-2).

^{19}F NMR (471 MHz, $CDCl_3$): δ [ppm] = −113.6 (*virt.* td, $^3J_{H–F} \approx {}^3J_{H–F}$ = 9.0 Hz,, $^4J_{H–F}$ = 5.8 Hz, 1F).

HRMS (+ESI): calc. for $C_{11}H_{12}FN_2O$ $[M+H]^+$: 207.0928; found: 207.0927.

IR (ATR): $\tilde{\nu}$ [cm^{-1}] = 3173 (w, N–H), 2954 (w, C–H), 2888 (w, C–H), 1668 (vs, C=O), 1609 (s, C=C), 1518 (m, C=C), 1444 (vs), 1170 (s, C–F), 1157 (s), 851 (vs), 777 (s, C–H), 745 (s, C–H), 706 (m).

m.p. = 203-205 °C.

Specific rotation: α_D^{25}: 45.7 (c = 1.0 $CHCl_3$) [99% *ee*].

Chiral HPLC: 99% ee [©CHIRALPAK AS-H, 20 °C, 30% ^{i}PrOH/*n*-heptane, 1mL/min, 210 nm, t_R = 10.6 min (major), 33.2 min (minor)].

*5.1.8. 8-(Trifluoromethyl)-2,3,6,10b-tetrahydropyrrolo[1,2-c]quinazolin-5(1H)-one (**5h**)*

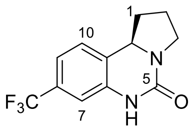

Following GP 4, compound **5h** was obtained as a colorless solid (25.4 mg, 99.1 µmol, 99%).

***R*f**: 0.24 (CH_2Cl_2/ac = 4/1) [UV, $KMnO_4$].

^{1}H NMR (500 MHz, $CDCl_3$): δ [ppm] = 1.93 – 2.17 (m, 3H, H^a-1, H^a-2, H^b-2), 2.60 (*virt.* dtd, ^2J = 11.7 Hz, $^3J \approx {}^3J$ = 5.6 Hz, ^3J = 1.5Hz, 1H, H^b-1), 3.60 – 3.70 (m, 2H, H^a-3, H^b-3), 4.67 (*virt.* ddt, ^3J = 9.9 Hz, ^3J = 5.6 Hz, $^4J \approx {}^5J$ = 1.2 Hz, 1H, H-10b), 6.98 (d, ^4J = 1.6 Hz, 1H, H-7), 7.15 (d, ^3J = 7.9 Hz, 1H, H-10), 7.22 (dd, ^3J = 8.6 Hz, ^4J = 1.6 Hz, 1H, H-9), 7.67 (bs, 1H, NH).

^{13}C NMR (126 MHz, $CDCl_3$): δ [ppm] = 153.1 (C-5), 138.0 (C-10a), 130.9 (q, $^{2}J_{C-F}$ = 32.5 Hz, C-8), 125.6 (q, $^{4}J_{C-F}$ = 1.0 Hz, C-6a), 125.5 (C-10), 123.9 (q, $^{1}J_{C-F}$ = 272.4 Hz, CF_3), 119.0 (q, $^{3}J_{C-F}$ = 3.9 Hz, C-9), 110.8 (q, $^{3}J_{C-F}$ = 3.9 Hz, C-7), 58.0 (C-10b), 44.7 (C-3), 31.8 (C-1), 22.9 (C-2).

^{19}F NMR (471 MHz, $CDCl_3$): δ [ppm] = −62.68 (s, 3F).

HRMS (+ESI): calc. for $C_{12}H_{12}F_3N_2O$ $[M+H]^+$: 257.0896; found: 257.0894.

IR (ATR): $\tilde{\nu}$ [cm^{-1}] = 3182 (w, N–H), 2995 (w, C–H), 2883 (w, C–H), 1669 (s, C=O), 1599 (m, C=C), 1532 (w, C=C), 1445 (m), 1329 (s), 1118 (vs, C–F), 1069 (s), 814 (m), 774 (m, C–H), 759 (m, C–H), 714 (m).

m.p. = 224-226 °C.

Specific rotation: α_D^{25}: 63.9 (c = 1.0 $CHCl_3$) [95% *ee*].

Chiral HPLC: 95% ee [©CHIRALPAK AD-H, 20 °C, 10% ^{i}PrOH/*n*-heptane, 1mL/min, 210 nm, t_R = 15.1 min (minor), 16.6 min (major)].

*5.1.9. 9-Vinyl-2,3,6,10b-tetrahydropyrrolo[1,2-c]quinazolin-5(1H)-one (**5i**)*

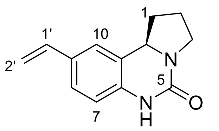

Following GP 4, compound **5i** was obtained as a colorless solid (17.2 mg, 80.3 µmol, 80%).

***R*f**: 0.49 (CH_2Cl_2/ac = 4/1) [UV, $KMnO_4$].

^{1}H NMR (500 MHz, $CDCl_3$): δ [ppm] =

1.92 – 2.18 (m, 3H, H^a-1, H^a-2, H^b-2), 2.56 – 2.61 (m, 1H, H^b-1), 3.60 (ddd, ^{2}J = 11.1 Hz, ^{3}J = 9.1 Hz, ^{3}J = 3.1 Hz, 1H, H^a-3), 3.66 (*virt.* dt, ^{2}J = 11.1 Hz, $^{3}J \approx {}^{3}J$ = 8.5 Hz, 1H, H^b-3), 4.64 (dd, ^{3}J = 10.0 Hz, ^{3}J = 5.5 Hz, 1H, H-10b), 5.15 (dd, ^{3}J = 10.9 Hz, ^{2}J = 0.8 Hz, 1H, H-2'$_{trans}$), 5.62 (dd, ^{3}J = 17.6 Hz, ^{2}J = 0.8 Hz, 1H, H-2'$_{cis}$), 6.61 – 6.68 (m, 2H, H-7, H-1'), 7.08 (*virt.* t, $^{3}J \approx {}^{4}J$ = 1.7 Hz, 1H, H-10), 7.12 (bs, 1H, NH), 7.23 (dd, ^{3}J = 8.2 Hz, ^{4}J = 1.6 Hz, 1H, H-8).

^{13}C NMR (126 MHz, $CDCl_3$): δ [ppm] = 153.2 (C-5), 137.0 (C-6a), 136.2 (C-1'), 132.1 (C-9), 126.5 (C-8), 122.9 (C-10), 122.2 (C-10a), 114.0 (C-7), 112.3 (C-2'), 58.2 (C-10b), 44.7 (C-3), 32.0 (C-1), 22.9 (C-1).

HRMS (+ESI): calc. for $C_{13}H_{15}N_2O$ $[M+H]^+$: 215.1179; found: 215.1178.

IR (ATR): $\tilde{\nu}$ [cm^{-1}] = 3189 (w, N–H), 2935 (w, C–H), 2886 (w, C–H), 1668 (vs, C=O), 1614 (m, C=C), 1595 (m, C=C), 1507 (m, C=C), 1442 (s), 1316 (m), 1268 (s), 833 (vs), 750 (s, C–H), 695 (m).

m.p. = 172-174 °C.

Specific rotation: α_D^{25}: 21.9 (c = 1.0 $CHCl_3$) [95% *ee*].

Chiral HPLC: 95% ee [©CHIRALPAK AD-H, 20 °C, 30% ^{i}PrOH/*n*-heptane, 1mL/min, 210 nm, t_R = 8.1 min (major), 10.7 min (minor)].

*5.1.10. 9-Allyl-2,3,6,10b-tetrahydropyrrolo[1,2-c]quinazolin-5(1H)-one (**5j**)*

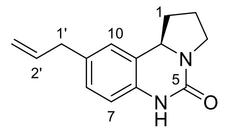

Following GP 4, compound **5j** was obtained as a colorless solid (22.4 mg, 99.4 µmol, 99%).

***R*f**: 0.22 (CH_2Cl_2/ac = 4/1) [UV, $KMnO_4$].

^{1}H NMR (500 MHz, $CDCl_3$): δ [ppm] = 1.90 – 2.13 (m, 3H, H^a-1, H^a-2, H^b-2), 2.53 – 2.58 (m, 1H, H^b-1), 3.32 (dt, ^3J = 6.6 Hz, $^4J \approx {}^4J$ = 1.6 Hz, 2H, H-1'), 3.56 – 3.67 (m, 2H, H^a-3, H^b-3), 4.62 (dd, ^3J = 10.1 Hz, ^3J = 5.5 Hz, 1H, H-10b), 5.04 – 5.09 (m, 2H, H-3'$_{trans}$, H-3'$_{cis}$), 5.93 (ddt, ^3J = 18.0 Hz, ^3J = 9.4 Hz, ^3J = 6.6 Hz, 1H, H-2'), 6.64 (d, ^3J = 8.0 Hz, 1H, H-7), 6.86 (dd, ^4J = 2.0 Hz, ^4J = 0.7 Hz, 1H, H-10), 6.92 (bs, 1H, NH), 6.99 (dd, ^3J = 8.0 Hz, ^4J = 2.0 Hz, 1H, H-8).

^{13}C NMR (126 MHz, $CDCl_3$): δ [ppm] = 153.4 (C-5), 137.6 (C-2'), 135.4 (C-6a), 134.1 (C-9), 128.6 (C-8), 125.1 (C-10), 122.1 (C-10a), 116.0 (C-3'), 113.8 (C-7), 58.2 (C-10b), 44.7 (C-3), 39.7 (C-1'), 31.9 (C-1), 23.0 (C-2).

HRMS (+ESI): calc. for $C_{14}H_{17}N_2O$ $[M+H]^+$: 229.1335; found: 229.1334.

IR (ATR): $\tilde{\nu}$ [cm^{-1}] = 3183 (w, N–H), 2971 (w, C–H), 2928 (w, C–H), 2885 (w, C–H), 1666 (vs, C=O), 1639 (m, C=C), 1598 (m, C=C), 1509 (m, C=C), 1446 (s), 1290 (m), 833 (s), 789 (m), 751 (s, C–H), 720 (m, C–H), 703 (m).

m.p. = 161-163 °C.

Specific rotation: α_D^{25}: 36.0 (c = 1.0 $CHCl_3$) [93% *ee*].

Chiral HPLC: 93% ee [©CHIRALPAK AD-H, 20 °C, 30% ^{i}PrOH/*n*-heptane, 1mL/min, 210 nm, t_R = 8.7 min (major), 10.5 min (minor)].

*5.1.11. 7-Methyl-2,3,6,10b-tetrahydropyrrolo[1,2-c]quinazolin-5(1H)-one (**5k**)*

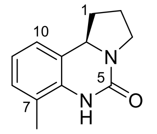

Following GP 4, compound **5k** was obtained as a colorless solid (18.0 mg, 89.0 µmol, 89%).

***R*f**: 0.31 (CH_2Cl_2/ac = 4/1) [UV, $KMnO_4$].

^{1}H NMR (500 MHz, $CDCl_3$): δ [ppm] = 1.91 – 2.14 (m, 3H, H^a-1, H^a-2, H^b-2), 2.21 (s, 3H, CH_3), 2.53 – 2.60 (m, 1H, H^b-1), 3.57 – 3.69 (m, 2H, H^a-3, H^b-3), 4.65 (dd, ^3J = 10.1 Hz, ^3J = 5.5 Hz, 1H, H-11a), 6.51 (bs, 1H, NH), 6.87 – 6.94 (m, 2H, H-9, H-10), 7.02 – 7.06 (m, 1H, H-8).

^{13}C NMR (126 MHz, $CDCl_3$): δ [ppm] = 153.2 (C-5), 135.3 (C-6a), 129.9 (C-8), 122.8 (C-10), 122.3 (C-9), 121.6 (C-10a†), 121.5 (C-7†), 58.3 (C-10b), 44.8 (C-3), 32.1 (C-1), 23.0, 17.0 (CH_3).

† assignment is interconvertible

HRMS (+ESI): calc. for $C_{12}H_{15}N_2O$ $[M+H]^+$: 211.1179; found: 211.1178.

IR (ATR): $\tilde{\nu}$ [cm^{-1}] = 3208 (w, N–H), 2970 (w, C–H), 2952 (w, C–H), 2899 (w, C–H), 1658 (vs, C=O), 1603 (w, C=C), 1436 (s), 1267 (m), 1164 (m), 813 (w), 787 (m, C–H), 740 (m, C–H), 710 (m), 675 (m).

m.p. = 188-190 °C.

Specific rotation: α_D^{25}: 52.1 (c = 1.0 $CHCl_3$) [92% *ee*].

Chiral HPLC: 92% ee [©CHIRALPAK IA, 20 °C, 10% ^{i}PrOH/*n*-heptane, 1mL/min, 210 nm, t_R = 10.1 min (major), 12.4 min (minor)].

*5.1.12. 9-Methoxy-2,3,6,10b-tetrahydropyrrolo[1,2-c]quinazolin-5(1H)-one (**5l**)*

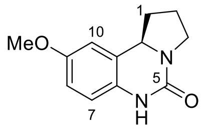

Following GP 4, compound **5l** was obtained as a colorless solid (21.7 mg, 99.4 µmol, 99%).

***R*f**: 0.20 (CH_2Cl_2/ac = 4/1) [UV, $KMnO_4$].

^{1}H NMR (500 MHz, $CDCl_3$): δ [ppm] = 1.90 – 2.05 (m, 2H, H^a-1, H^a-2), 2.06 – 2.12 (m, 1H, H^b-2), 2.50 – 2.55 (m, 1H, H^b-1), 3.59 (ddd, ^2J = 11.0 Hz, ^3J = 9.1 Hz, ^3J = 3.1 Hz, 1H, H^a-3), 3.65 (ddd, ^2J = 11.0 Hz, ^3J = 8.6 Hz, ^3J = 7.6 Hz, 1H, H^b-3), 3.77 (s, 3H, OCH_3), 4.61 (dd, ^3J = 10.1 Hz, ^3J = 5.5 Hz, 1H, H-10b), 6.61 (dd, ^4J = 2.8 Hz, ^4J = 1.0 Hz, 1H, H-10), 6.64 (d, ^3J = 8.6 Hz, 1H, H-7), 6.73 (ddd, ^3J = 8.6 Hz, ^4J = 2.8 Hz, ^6J = 0.8 Hz, 1H, H-8), 6.99 (bs, 1H, NH).

^{13}C NMR (126 MHz, $CDCl_3$): δ [ppm] = 155.2 (C-7), 153.5 (C-5), 130.9 (C-6a), 123.3 (C-10a), 114.5 (C-7), 113.3 (C-8), 111.0 (C-10), 58.3 (C-10b), 55.8 (OCH_3), 44.7 (C-3), 31.9 (C-1), 22.9 (C-2).

HRMS (+ESI): calc. for $C_{12}H_{15}N_2O_2$ $[M+H]^+$: 219.1126; found: 219.1128.

IR (ATR): $\tilde{\nu}$ [cm^{-1}] = 3189 (w, N–H), 2933 (w, C–H), 2897 (w, C–H), 1669 (vs, C=O), 1510 (s, C=C), 1446 (vs), 1264 (s), 1243 (s, C–O), 1154 (s), 817 (s), 799 (s), 770 (s, C–H), 750 (s, C–H), 717 (s), 683 (s).

m.p. = 141-143 °C.

Specific rotation: α_D^{25}: 62.1 (c = 1.0 $CHCl_3$) [94% *ee*].

Chiral HPLC: 94% ee [©CHIRALPAK AD-H, 20 °C, 30% ^{i}PrOH/*n*-heptane, 1mL/min, 210 nm, t_R = 11.7 min (major), 13.5 min (minor)].

*5.1.13. 7-Methoxy-2,3,6,10b-tetrahydropyrrolo[1,2-c]quinazolin-5(1H)-one (**5m**)*

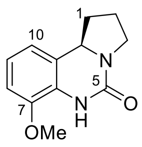

Following GP 4, compound **5m** was obtained as a colorless solid (20.6 mg, 94.4 µmol, 94%).

***R*f**: 0.40 (CH_2Cl_2/ac = 4/1) [UV, $KMnO_4$].

^{1}H NMR (500 MHz, $CDCl_3$): δ [ppm] = 1.89 – 2.13 (m, 3H, H^a-1, H^a-2, H^b-2), 2.51 – 2.57 (m, 1H, H^b-1), 3.58 (ddd, ^2J = 11.0 Hz, ^3J = 9.1 Hz, ^3J = 3.1 Hz, 1H, H^a-3), 3.66 (*virt.* dt, ^2J = 11.0 Hz, $^3J \approx {}^3J$ = 8.4 Hz, 1H, H^b-3), 3.85 (s, 3H, OCH_3), 4.66 (dd, ^3J = 10.1 Hz, ^3J = 5.5 Hz, 1H, H-11a), 6.67 (*virt.* dt, ^3J = 7.9 Hz, $^4J \approx {}^4J$ = 0.9 Hz, 1H, H-10), 6.6 (*virt.* dt, ^3J = 7.9 Hz, $^4J \approx {}^6J$ = 0.9 Hz, 1H, H-8), 6.92 (*virt.* t, $^3J \approx {}^3J$ = 7.9 Hz, 1H, H-9), 6.95 (bs, 1H, NH).

^{13}C NMR (126 MHz, $CDCl_3$): δ [ppm] = 152.9 (C-5), 145.3 (C-7), 126.8 (C-6a), 122.3 (C-10a), 122.2 (C-9), 116.8 (C-10), 109.6 (C-8), 58.4 (C-10b), 55.9 (OCH_3), 44.7 (C-3), 32.0 (C-1), 23.0 (C-2).

HRMS (+ESI): calc. for $C_{12}H_{15}N_2O_2$ $[M+H]^+$: 219.1128; found: 219.1126.

IR (ATR): $\tilde{\nu}$ [cm^{-1}] = 3226 (w, N–H), 2966 (w, C–H), 2935 (w, C–H), 2901 (w, C–H), 2837 (w, C–H), 1652 (vs, C=O), 1492 (m, C=C), 1431 (s), 1263 (s, C–O), 1075 (s), 812 (w), 781 (m, C–H), 758 (m, C–H), 732 (vs, C–H).

m.p. = 127-129 °C.

Specific rotation: α_D^{25}: 25.9 (c = 1.0 $CHCl_3$) [58% *ee*].

Chiral HPLC: 58% ee [©CHIRALPAK AD-H, 20 °C, 30% ^{i}PrOH/*n*-heptane, 1mL/min, 210 nm, t_R = 8.1 min (major), 9.1 min (minor)].

*5.1.14. 8-(Furan-3-yl)-2,3,6,10b-tetrahydropyrrolo[1,2-c]quinazolin-5(1H)-one (**5n**)*

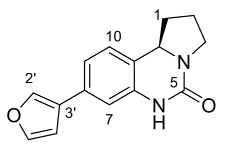

Following GP 4, compound **5n** was obtained as a colorless solid (22.7 mg, 89.2 µmol, 89%).

***R*f**: 0.40 (CH_2Cl_2/ac = 4/1) [UV, $KMnO_4$].

^{1}H NMR (500 MHz, $CDCl_3$): δ [ppm] = 1.91 – 2.07 (m, 2H, H^a-1, H^a-2), 2.08 – 2.15 (m, 1H, H^b-2), 2.55 – 2.60 (m, 1H, H^b-1), 3.60 (ddd, ^2J = 11.1 Hz, ^3J = 9.2 Hz, ^3J = 3.0 Hz, 1H, H^a-3), 3.67 (ddd, ^2J = 11.1 Hz, ^3J = 8.9 Hz, ^3J = 7.8 Hz, 1H, H^b-3), 4.65 (ddd, ^3J = 10.0 Hz, ^3J = 5.31 Hz, ^4J = 0.9 Hz, 1H, H-10b), 6.65 (dd, ^3J = 1.7 Hz, ^4J = 0.9 Hz, 1H, H-4'), 6.81 (dd, ^4J = 1.6 Hz, 1H, H-7), 7.00 (bs, 1H, NH), 7.05 (dd, ^3J = 7.8 Hz, ^4J = 0.9 Hz, 1H, H-10), 7.10 (dd, ^3J = 7.8 Hz, ^4J = 1.6 Hz, 1H, H-9), 7.47 (*virt.* t, $^3J \approx {}^4J$ = 1.7 Hz, 1H, H-5'), 7.70 (dd, ^4J = 1.7 Hz, ^4J = 0.9 Hz, 1H, H-2').

^{13}C NMR (126 MHz, $CDCl_3$): δ [ppm] = 153.2 (C-5), 143.9 (C-5'), 138.8 (C-2'), 137.8 (C-6a), 132.9 (C-8), 125.9 (C-3'), 125.5 (C-10), 120.9 (C-4a),, 120.0 (C-9), 111.0 (C-7), 108.9 (C-4'), 58.1 (C-10b), 44.7 (C-3), 32.0 (C-1), 23.0 (C-2).

HRMS (+ESI): calc. for $C_{15}H_{15}N_2O_2$ $[M+H]^+$: 255.1128; found: 255.1125.

IR (ATR): $\tilde{\nu}$ [cm^{-1}] = 3207 (w, N–H), 2981 (w, C–H), 2953 (w, C–H), 2870 (w, C–H), 2822 (w, C–H), 1667 (vs, C=O), 1623 (m, C=C), 1592 (m, C=C), 1579 (m, C=C), 1524 (w, C=C), 1500 (w, C=C), 1439 (vs), 1056 (m), 873 (vs), 811 (m), 790 (s), 766 (vs, C–H), 757 (m, C–H), 727 (m, C–H).

m.p. = >230 °C.

Specific rotation: α_D^{25}: −28.2 (c = 1.0 $CHCl_3$) [98% *ee*].

Chiral HPLC: 98% ee [©CHIRALPAK IA, 20 °C, 30% ^{i}PrOH/*n*-heptane, 1mL/min, 210 nm, t_R = 12.2 min (minor), 14.3 min (major)].

*5.1.15. 8-(Thiophen-3-yl)-2,3,6,10b-tetrahydropyrrolo[1,2-c]quinazolin-5(1H)-one (**5o**)*

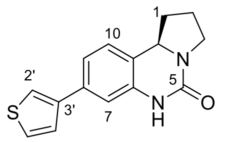

Following GP 4, compound **5o** was obtained as a colorless solid (24.2 mg, 89.5 µmol, 89%).

***R*f**: 0.28 (CH_2Cl_2/ac = 4/1) [UV, $KMnO_4$].

^{1}H NMR (500 MHz, $CDCl_3$): δ [ppm] = 1.92 – 2.18 (m, 3H, H^a-1, H^a-2, H^b-2), 2.56 – 2.60 (m, 1H, H^b-1), 3.61 (ddd, ^2J = 11.1 Hz, ^3J = 9.1 Hz, ^3J = 3.1 Hz, 1H, H^a-3), 3.67 (*virt.* dt, ^2J = 11.1 Hz, $^3J \approx {}^3J$ = 8.4 Hz, 1H, H^b-3), 4.66 (ddd, ^3J = 10.0 Hz, ^3J = 5.3 Hz, ^4J = 1.0 Hz, 1H, H-10b), 6.91 (d, ^4J = 1.7 Hz, 1H, H-7), 7.05 (bs, 1H, NH), 7.07 (dd, ^3J = 7.9 Hz, ^4J = 1.0 Hz, 1H, H-10), 7.20 (dd, ^3J = 7.9 Hz, ^4J = 1.7 Hz, 1H, H-9), 7.34 (dd, ^3J = 5.0 Hz, ^4J = 1.4 Hz, 1H, H-4'), 7.38 (dd, ^3J = 5.0 Hz, ^4J = 2.9 Hz, 1H, H-5'), 7.43 (dd, ^4J = 2.9 Hz, ^4J = 1.4 Hz, 1H, H-2').

^{13}C NMR (126 MHz, $CDCl_3$): δ [ppm] = 153.4 (C-5), 141.44 (C-3'), 137.1 (C-6a), 136.5 (C-8), 126.6 (C-4'†), 126.4 (C-5'†), 125.5 (C-10), 121.0 (C-8‡), 121.1 (C-2'‡), 120.7 (C-10a), 111.9 (C-7), 58.2 (C-10b), 44.9 (C-3), 32.0 (C-1), 23.0 (C-2).

†,‡ assignment is interconvertible

HRMS (+ESI): calc. for $C_{15}H_{15}N_2OS$ $[M+H]^+$: 271.0900; found: 271.0897.

IR (ATR): $\tilde{\nu}$ [cm^{-1}] = 3191 (w, N–H), 2973 (w, C–H), 2948 (w, C–H), 2869 (w, C–H), 1663 (vs, C=O), 1621 (m, C=C), 1586 (m, C=C), 1543 (w, C=C), 1512 (w, C=C), 1440 (vs), 1228 (m), 1088 (m), 823 (m), 785 (vs, C–H), 755 (s, C–H), 736 (s, C–H), 718 (m).

m.p. = 228-230 °C.

Specific rotation: α_D^{25}: −57.9 (c = 1.0 $CHCl_3$) [97% *ee*].

Chiral HPLC: 97% ee [©CHIRALPAK IC, 20 °C, 30% ^{i}PrOH/*n*-heptane, 1mL/min, 210 nm, t_R = 13.5 min (minor), 15.6 min (major)].

*5.1.16. tert-Butyl 2-(5-oxo-1,2,3,5,6,10b-hexahydropyrrolo[1,2-c]quinazolin-8-yl)-1H-pyrrole-1-carboxylate (**5p**)*

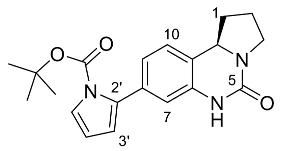

Following GP 4, compound **5p** was obtained as a colorless solid (32.3 mg, 91.4 µmol, 91%).

***R*f**: 0.41 (CH_2Cl_2/ac = 4/1) [UV, $KMnO_4$].

^{1}H NMR (500 MHz, $CDCl_3$): δ [ppm] = 1.41 [s, 9H, $(CH_3)_3$], 1.91 – 2.14 (m, 3H, H^a-1, H^a-2, H^b-2), 2.54 – 2.59 (m, 1H, H^b-1), 3.59 (ddd, ^2J = 11.1 Hz, ^3J = 9.1 Hz, ^3J = 3.1 Hz, 1H, H^a-3), 3.66 (*virt.* dt, ^2J = 11.1 Hz, $^3J \approx {}^3J$ = 8.4 Hz, 1H, H^b-3), 4.66 (ddd, ^3J = 9.9 Hz, ^3J = 5.4 Hz, ^4J = 0.9 Hz, 1H, H-10b), 6.18 (dd, ^3J = 3.3 Hz, ^4J = 1.8 Hz, 1H, H-3'), 6.21 (*virt.* t, $^3J \approx {}^3J$ = 3.3 Hz, 1H, H-4'), 6.68 (d, ^4J = 1.6 Hz, 1H, H-7), 6.84 (bs, 1H, NH), 6.96 (dd, ^3J = 7.8 Hz, ^4J = 1.6 Hz, 1H, H-9), 7.03 (d, ^3J = 7.8 Hz, 1H, H-10), 7.32 (dd, ^3J = 3.3 Hz, ^4J = 1.8 Hz, 1H, H-5').

^{13}C NMR (126 MHz, $CDCl_3$): δ [ppm] = 153.3 (C-5), 149.3 (COO), 136.6 (C-6a), 134.6 (C-8), 134.3 (C-2'), 124.2 (C-10), 123.4 (C-9), 122.9 (C-5'), 120.9 (C-10a), 114.9 (C-3'), 114.4 (C-7), 110.8 (C-4'), 83.9 [$C(CH_3)_3$], 58.1 (C-10b), 44.7 (C-3), 32.0 (C-1), 27.8 [$C(CH_3)_3$], 23.0 (C-2).

HRMS (+ESI): calc. for $C_{10}H_{24}N_3O_3$ $[M+H]^+$: 354.1812; found: 354.1809.

IR (ATR): $\tilde{\nu}$ [cm^{-1}] = 3172 (w, N–H), 2971 (w, C–H), 1729 (m, C=O), 1668 (vs, C=O), 1624 (w, C=C), 1586 (w, C=C), 1524 (w, C=C), 1454 (s), 1340 (s), 1315 (vs), 1287 (m, C–O), 1142 (vs), 810 (m), 773 (m, C–H), 763 (m, C–H), 738 (vs, C–H), 712 (m).

m.p. = >192 °C (decomposition).

Specific rotation: α_D^{25}: 14.7 (c = 1.0 $CHCl_3$) [97% *ee*].

Chiral HPLC: 97% ee [©CHIRALPAK AD-H, 20 °C, 30% ^{i}PrOH/*n*-heptane, 1mL/min, 210 nm, t_R = 8.9 min (minor), 17.7 min (major)].

*5.1.17. 8-(Pyridin-3-yl)-2,3,6,10b-tetrahydropyrrolo[1,2-c]quinazolin-5(1H)-one (**5q**)*

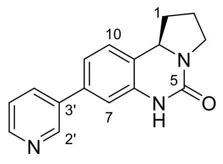

Following GP 4, compound **5q** was obtained as a colorless solid (24.6 mg, 92.9 µmol, 93%) as an inseparable mixture with remaining substrate **4q** (3.2 mg, 7.10 µmol, 7%).

***R*f**: 0.41 (CH_2Cl_2/MeOH = 19/1) [UV, $KMnO_4$].

^{1}H NMR (500 MHz, $CDCl_3$): δ [ppm] = 1.94 – 2.17 (m, 3H, H^a-1, H^a-2, H^b-2), 2.58 – 2.63 (m, 1H, H^b-1), 3.62 (ddd, ^2J = 11.1 Hz, ^3J = 9.1 Hz, ^3J = 3.1 Hz, 1H, H^a-3), 3.68 (*virt.* dt, ^2J = 11.1 Hz, $^3J \approx {}^3J$ = 8.4 Hz, 1H, H^b-3), 4.66 (dd, ^3J = 9.9 Hz, ^3J = 5.5 Hz, 1H, H-10b), 6.91 (d, ^4J = 1.6 Hz, 1H, H-7), 7.15 (d, ^3J = 7.9 Hz, 1H, H-10), 7.03 (dd, ^3J = 7.9 Hz, ^4J = 1.6 Hz, 1H, H-9), 7.27 (bs, 1H, NH), 7.37 (ddd, ^3J = 7.8 Hz, ^3J = 4.8 Hz, ^5J = 0.9 Hz, 1H, H-5'), 7.84 (ddd, ^3J = 3.8 Hz, ^4J = 2.2 Hz, ^4J = 1.7 Hz, 1H, H-4'), 8.60 (dd, ^3J = 4.8 Hz, ^4J = 1.7 Hz, 1H, H-6'), 8.80 (dd, ^4J = 2.2 Hz, ^5J = 0.9 Hz, 1H, H-2').

^{13}C NMR (126 MHz, $CDCl_3$): δ [ppm] = 153.2 (C-5), 148.7 (C-6'), 148.1 (C-2'), 138.3 (C-6a†), 138.2 (C-8†), 136.1 (C-3'), 134.6 (C-4'), 125.8 (C-10), 123.8 (C-5'), 122.1 (C-10a), 121.2 (C-9), 112.3 (C-7), 58.0 (C-10b), 44.7 (C-3), 32.0 (C-1), 23.0 (C-2).

† assignment is interconvertible

HRMS (+ESI): calc. for $C_{16}H_{16}N_3O$ $[M+H]^+$: 266.1288; found: 266.1286.

IR (ATR): $\tilde{\nu}$ [cm^{-1}] = 3199 (w, N–H), 1652 (s, C=O), 1626 (s, C=N), 1594 (s, C=C), 1530 (w, C=C), 1444 (s), 1416 (s), 1374 (s), 1057 (m), 803 (s), 775 (s, C–H), 752 (s, C–H), 706 (vs), 658 (s).

m.p. = 159-161 °C.

Specific rotation: α_D^{25}: −50.2 (c = 1.0 $CHCl_3$) [97% *ee*].

Chiral HPLC: 97% ee [©CHIRALPAK AD-H, 20 °C, 30% ^{i}PrOH/*n*-heptane, 1mL/min, 210 nm, t_R = 23.8 min (minor), 26.4 min (major)].

*5.1.18. Ethyl (E)-3-(5-oxo-1,2,3,5,6,10b-hexahydropyrrolo[1,2-c]quinazolin-8-yl)acrylate (**5r**)*

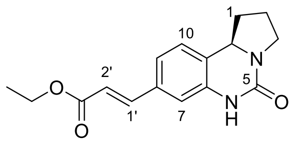

Following GP 4, compound **5r** was obtained as a colorless solid (22.0 mg, 96.8 µmol, 97%).

***R*f**: 0.23 (CH_2Cl_2/ac = 4/1) [UV, $KMnO_4$].

^{1}H NMR (500 MHz, $CDCl_3$): δ [ppm] = 1.27 (t, ^3J = 7.1 Hz, 3H, CH_3), 1.84 – 2.08 (m, 3H, H^a-1, H^a-2, H^b-2), 2.50 (*virt.* dtd, ^2J = 11.6 Hz, $^3J \approx {}^3J$ = 5.8 Hz, ^3J = 1.7 Hz, 1H, H^b-1), 3.54 (ddd, ^2J = 11.1 Hz, ^3J = 9.1 Hz, ^3J = 3.1 Hz, 1H, H^a-3), 3.61 (ddd, ^2J = 11.1 Hz, ^3J = 8.9 Hz, ^3J = 7.8 Hz, 1H, H^b-3), 4.19 (q, ^3J = 7.1 Hz, 2H, OCH_2), 4.58 (dd, ^3J = 10.1 Hz, ^3J = 5.8 Hz, 1H, H-10b), 6.32 (d, ^3J = 16.0 Hz, 1H, H-2'), 6.80 (d, ^4J = 1.6 Hz, 1H, H-7), 6.99 (d, ^3J = 7.9 Hz, 1H, H-10), 7.06 (dd, ^3J = 7.9 Hz, ^4J = 1.6 Hz, 1H, H-9), 7.38 (bs, 1H, NH), 7.53 (d, ^3J = 16.0 Hz, 1H, H-1').

^{13}C NMR (126 MHz, $CDCl_3$): δ [ppm] = 167.0 (COO), 153.2 (C-5), 143.9 (C-1'), 138.0 (C-6a), 134.8 (C-8), 125.5 (C-10), 124.2 (C-10a), 122.2 (C-9), 118.8 (C-2'), 112.9 (C-7), 60.8 (OCH_2), 58.1 (C-10b), 44.7 (C-3), 31.9 (C-1), 22.9 (C-2), 14.5 (CH_3).

HRMS (+ESI): calc. for $C_{16}H_{19}N_2O_3$ $[M+H]^+$: 287.1390; found: 287.1387.

IR (ATR): $\tilde{\nu}$ [cm^{-1}] = 3208 (w, N–H), 2971 (w, C–H), 2880 (w, C–H), 1705 (s, C=O), 1666 (vs, C=O), 1635 (s), 1586 (m, C=C), 1526 (w, C=C), 1443 (s), 1318 (s), 1279 (s, C–O), 1031 (m), 804 (m), 779 (m, C–H), 756 (s, C–H), 729 (m), 704 (m).

m.p. = 154-156 °C.

Specific rotation: α_D^{25}: 87.3 (c = 0.25 $CHCl_3$) [96% *ee*].

Chiral HPLC: 96% ee [©CHIRALCEL OD-RH, 20 °C, 20 → 100% (30 min) ^{i}PrOH/*n*-heptane, 1mL/min, 210 nm, t_R = 14.3 min (major), 17.3 min (minor)].

*5.1.19. 9-(4-Methoxyphenyl)-2,3,6,10b-tetrahydropyrrolo[1,2-c]quinazolin-5(1H)-one (**5s**)*

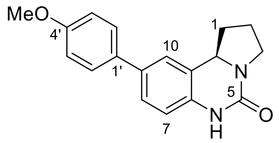

Following GP 4, compound **5s** was obtained as a colorless solid (28.5 mg, 96.8 µmol, 97%).

***R*f**: 0.30 (CH_2Cl_2/ac = 4/1) [UV, $KMnO_4$].

^{1}H NMR (500 MHz, $CDCl_3$): δ [ppm] = 1.96 – 2.16 (m, 3H, H^a-1, H^a-2, H^b-2), 2.58 – 2.66 (m, 1H, H^b-1), 3.62 (ddd, ^2J = 10.8 Hz, ^3J = 8.9 Hz, ^3J = 3.0 Hz, 1H, H^a-3), 3.68 (*virt.* dt, ^2J = 10.8 Hz, $^3J \approx {}^3J$ = 8.1 Hz, 1H, H^b-3), 3.85 (s, 3H, OCH_3), 4.69 (dd, ^3J = 9.7 Hz, ^3J = 5.5 Hz, 1H, H-10b), 6.77 (d, ^3J = 8.2 Hz, 1H, H-7), 6.94 – 6.99 (m, 2H, H-3'), 7.19 (bs, 1H, NH), 7.21 (*virt.* t, $^4J \approx {}^4J$ = 1.8 Hz, 1H, H-10), 7.35 (ddd, ^3J = 8.2 Hz, ^4J = 1.8 Hz, ^6J = 0.8 Hz, 1H, H-8), 7.43 – 7.47 (m, 2H, H-2').

^{13}C NMR (126 MHz, $CDCl_3$): δ [ppm] = 159.0 (C-4'), 153.3 (C-5), 136.1 (C-6a), 135.3 (C-9), 133.3 (C-1'), 127.9 (C-2'), 126.8 (C-8), 123.3 (C-10), 122.5 (C-10a), 114.4 (C-3'), 114.2 (C-7), 58.3 (C-10b), 55.5 (OCH_3), 44.7 (C-3), 32.0 (C-1), 23.0 (C-2).

HRMS (+ESI): calc. for $C_{11}H_{12}FN_2O$ [M+H]$^+$: 295.1441; found: 295.1439.

IR (ATR): $\tilde{\nu}$ [cm^{-1}] = 3192 (w, N–H), 2938 (w, C–H), 2889 (w, C–H), 1667 (s, C=O), 1619 (m, C=C), 1601 (m, C=C), 1576 (w, C=C), 1501 (m, C=C), 1439 (vs), 1245 (s, C–O), 1180 (s), 1023 (s), 820 (vs), 811 (vs), 749 (m, C–H), 706 (m).

m.p. = >230 °C.

Specific rotation: α_D^{25}: 48.1 (c = 1.0 $CHCl_3$) [91% *ee*].

Chiral HPLC: 91% ee [©CHIRALPAK AD-H, 20 °C, 30% ^{i}PrOH/*n*-heptane, 1mL/min, 210 nm, t_R = 13.8 min (major), 18.6 min (minor)].

*5.1.20. 8,9,10,10a-Tetrahydropyrido[2,3-e]pyrrolo[1,2-c]pyrimidin-6(5H)-one (**5t**)*

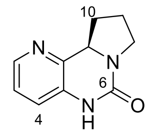

Following GP 4, compound **5t** was obtained as a colorless solid (19.8 mg; corrected without residual CH_2Cl_2: 18.5 mg, 97.7 µmol, 98%).

***R*f**: 0.08 (CH_2Cl_2/ac = 4/1) [UV, $KMnO_4$].

^{1}H NMR (500 MHz, $CDCl_3$): δ [ppm] = 1.98 – 2.19 (m, 3H, H^a-9, H^b-9, H^a-10), 2.69 (*virt.* dtd, ^2J = 12.1 Hz, $^3J \approx {}^3J$ = 6.1 Hz, ^3J = 1.9 Hz, 1H, H^b-10), 3.59 (ddd, ^2J = 11.1 Hz, ^3J = 9.1 Hz, ^3J = 3.5 Hz, 1H, H^a-8), 3.73 (*virt.* dt, ^2J = 11.1 Hz, $^3J \approx {}^3J$ = 8.1 Hz, 1H, H^b-8), 5.71 (dd, ^3J = 9.8 Hz, ^3J = 6.1 Hz, 1H, H-10a), 7.08 (dd, ^3J = 8.1 Hz, ^4J = 1.6 Hz, 1H, H-4), 7.12 (ddd, ^3J = 8.1 Hz, ^3J = 4.7 Hz, ^6J = 0.7 Hz, 1H, H-3), 8.00 (bs, 1H, NH), 8.18 (dd, ^3J = 4.7 Hz, ^4J = 1.6 Hz, 1H, H-2).

^{13}C NMR (126 MHz, $CDCl_3$): δ [ppm] = 153.0 (C-6), 142.8 (C-2), 142.0 (C-10b), 133.6 (C-4a), 123.4 (C-3), 121.0 (C-4), 59.9 (C-10a), 45.3 (C-8), 31.1 (C-10), 22.8 (C-9).

HRMS (+ESI): calc. for $C_{10}H_{12}N_3O$ [M+H]$^+$: 190.0975; found: 190.0973.

IR (ATR): $\tilde{\nu}$ [cm^{-1}] = 3206 (w, N–H), 2970 (w, C–H), 2885 (w, C–H), 1660 (vs, C=O), 1590 (m, C=C), 1496 (w), 1439 (s), 1421 (vs), 1057 (w), 812 (m), 777 (w, C–H), 750 (m, C–H), 731 (m, C–H).

m.p. = >230 °C.

Specific rotation: α_D^{25}: 38.0 (c = 1.0 $CHCl_3$) [81% *ee*].

Chiral HPLC: 81% ee [©CHIRALPAK AD-H, 20 °C, 10% ^{i}PrOH/*n*-heptane, 1mL/min, 210 nm, t_R = 12.2 min (major), 13.4 min (minor)].

*5.1.21. 2,2-Dimethyl-2,3,6,10b-tetrahydropyrrolo[1,2-c]quinazolin-5(1H)-one (**5u**)*

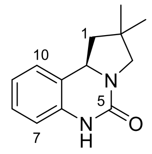

Following GP 4, compound **5u** was obtained as a colorless solid (16.6 mg, 88.2 µmol, 88%).

***R*f**: 0.50 (CH_2Cl_2/ac = 4/1) [UV, $KMnO_4$].

^{1}H NMR (500 MHz, $CDCl_3$): δ [ppm] = 1.19 (s, 3H, CH_3^a), 1.22 (s, 3H, CH_3^b), 1.92 (*virt.* t, $^2J \approx {}^3J$ = 11.2 Hz, 1H, H^a-1), 2.28 (dd, ^2J = 11.2 Hz, ^3J = 5.9 Hz, 1H, H^b-1), 3.36 (d, ^2J = 11.0 Hz, 1H, H^a-3), 3.39 (d, ^2J = 11.0 Hz, 1H, H^b-3), 4.84 (dd, ^3J = 11.2 Hz, ^3J = 5.9 Hz, 1H, H-10b), 6.71 (d, ^3J = 7.6 Hz, 1H, H-7), 6.95 – 7.03 (m, 2H, H-9, H-10), 7.03 (bs, 1H, NH), 7.17 (*virt.* td, $^3J \approx {}^3J$ = 7.6 Hz, ^4J = 2.2 Hz, 1H, H-8).

^{13}C NMR (126 MHz, $CDCl_3$): δ [ppm] = 153.4 (C-6), 137.4 (C-4a), 128.4 (C-8), 124.6 (C-10), 122.7 (C-10a), 122.4 (C-9), 113.8 (C-7), 58.1 (C-3), 57.2 (C-10b), 45.9 (C-1), 37.1 (C-2), 28.0 (CH_3^a), 27.9 (CH_3^b).

HRMS (+ESI): calc. for $C_{13}H_{17}N_2O$ $[M+H]^+$: 217.1335; found: 217.1335.

IR (ATR): $\tilde{\nu}$ [cm^{-1}] = 3206 (w, N–H), 2958 (w, C–H), 2928 (w, C–H), 2871 (w, C–H), 1661 (s, C=O), 1599 (s, C=C), 1509 (m, C=C), 1441 (m), 1034 (m), 811 (m), 751 (vs, C–H), 681 (s).

m.p. = 98-100 °C.

Specific rotation: α_D^{25}: 41.6 (c = 1.0 $CHCl_3$) [85% *ee*].

Chiral HPLC: 85% ee [©CHIRALPAK AS-H, 20 °C, 50% ^{i}PrOH/*n*-heptane, 1mL/min, 210 nm, t_R = 10.3 min (major), 16.8 min (minor)].

*5.1.22. 8,9,10,10a-Tetrahydropyrido[3,2-e]pyrrolo[1,2-c]pyrimidin-6(5H)-one (**5v**)*

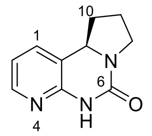

Following GP 4, compound **5v** was obtained as a colorless solid (13.5 mg, 71.4 µmol, 71%).

***R*f**: 0.15 (CH_2Cl_2/ac = 3/2) [UV, $KMnO_4$].

^{1}H NMR (500 MHz, $CDCl_3$): δ [ppm] = 1.90 – 2.00 (m, 1H, H^a-10), 2.00 – 2.10 (m, 1H, H^a-9), 2.10 – 2.18 (m, 1H, H^b-9), 2.54 – 2.61 (m, 1H, H^b-10), 3.60 (ddd, ^2J = 11.1 Hz, ^3J = 9.3 Hz, ^3J = 3.0 Hz, 1H, H^a-8), 3.69 (*virt.* dt, ^2J = 11.1 Hz, $^3J \approx {}^3J$ = 8.3 Hz, 1H, H^b-8), 4.66 (dd, ^3J = 10.3 Hz, ^3J = 5.5 Hz, 1H, H-11a), 6.95 (dd, ^3J = 7.5 Hz, ^3J = 5.2 Hz, 1H, H-2), 7.40 (*virt.* dt, ^3J = 7.5 Hz, $^4J \approx {}^6J$ = 1.5 Hz, 1H, H-1), 7.66 (bs, 1H, NH), 8.14 (d, ^3J = 5.2 Hz, 1H, H-3).

^{13}C NMR (126 MHz, $CDCl_3$): δ [ppm] = 151.7 (C-6), 150.3 (C-4a), 146.2 (C-3), 134.1 (C-1), 118.1 (C-2), 57.4 (C-10a), 44.7 (C-8), 31.7 (C-10), 22.9 (C-9).

HRMS (+ESI): calc. for $C_{10}H_{12}N_3O$ $[M+H]^+$: 190.0975; found: 190.0974.

IR (ATR): $\tilde{\nu}$ [cm^{-1}] = 3188 (w, N–H), 2968 (w, C–H), 2890 (w, C–H), 1675 (s, C=O), 1600 (m, C=C), 1415 (vs), 1160 (m), 820 (w), 796 (m), 771 (vs, C–H), 752 (m, C–H), 729 (m).

m.p. = 228-230 °C.

Specific rotation: α_D^{25}: 45.8 (c = 1.0 $CHCl_3$) [49% *ee*].

Chiral HPLC: 49% ee [©CHIRALPAK AD-H, 20 °C, 50% ^{i}PrOH/*n*-heptane, 1mL/min, 210 nm, t_R = 13.3 min (minor), 19.6 min (major)].

*5.1.23. 5,8,9,10,11,11a-Hexahydro-6H-pyrido[1,2-c]quinazolin-6-one (**5aa**)*

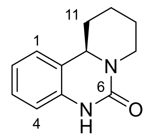

Following GP 4, compound **5aa** was obtained as a colorless solid (16.6 mg, 88.2 µmol, 88%).

***R*f**: 0.27 (CH_2Cl_2/ac = 4/1) [UV, $KMnO_4$].

^{1}H NMR (500 MHz, $CDCl_3$): δ [ppm] = 1.55 – 1.63 (m, 1H, H^a-9), 1.65 – 1.75 (m, 3H, H^b-9, H^a-10, H^a-11), 1.87 – 1.92 (m, 1H, H^b-11), 1.97 – 2.03 (m, 1H, H^b-10), 2.67 (*virt.* td, $^3J \approx {}^3J$ = 13.1 Hz, ^3J = 2.8 Hz, 1H, H^a-8), 4.47 (dd, ^3J = 10.6 Hz, ^3J = 3.4 Hz, 1H, H-11a), 4.58 (*virt.* ddt, ^3J =13.1 Hz, ^3J = 4.1 Hz, ^3J =1.9 Hz, 1H, H^b-8), 6.61 (dd, ^3J = 7.7 Hz, ^4J = 1.2 Hz, 1H, H-4), 6.90 – 6.95 (m, 2H, NH, H-2), 7.02 (dd, ^3J = 7.7 Hz, ^4J = 1.4 Hz, 1H, H-1), 7.14 (*virt.* td, $^3J \approx {}^3J$ = 7.7 Hz, ^4J = 1.4 Hz, 1H, H-3).

^{13}C NMR (126 MHz, $CDCl_3$): δ [ppm] = 152.6 (C-6), 135.5 (C-4a), 128.3 (C-3), 125.7 (C-1), 122.1 (C-2), 121.3 (C-11b), 113.5 (C-4), 59.3 (C-11a), 44.5 (C-8), 34.7 (C-11), 25.3 (C-10), 24.9 (C-9).

HRMS (+ESI): calc. for $C_{12}H_{15}N_2O$ $[M+H]^+$: 203.1179; found: 203.1177.

IR (ATR): $\tilde{\nu}$ [cm^{-1}] = 3192 (w, N–H), 2994 (w, C–H), 2942 (w, C–H), 2909 (w, C–H), 2860 (w, C–H), 1655 (s, C=O), 1602 (m, C=C), 1467 (m), 1259 (m), 1037 (w), 841 (m), 789 (m, C–H), 749 (vs, C–H), 716 (m), 705 (m).

m.p. = 129-131 °C.

Specific rotation: α_D^{25}: 91.0 (c = 1.0 $CHCl_3$) [98% *ee*].

Chiral HPLC: 98% ee [©CHIRALPAK AD-H, 20 °C, 30% ^{i}PrOH/*n*-heptane, 1mL/min, 210 nm, t_R = 9.3 min (major), 20.0 min (minor)].

*5.1.24. 5,8,13,13a-Tetrahydro-6H-isoquinolino[2,3-c]quinazolin-6-one (**5ab**)*

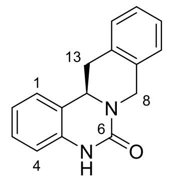

Following GP 4, compound **5ab** was obtained as a colorless solid (24.7 mg, 98.6 µmol, 99%).

***R*f**: 0.51 (CH_2Cl_2/ac = 4/1) [UV, $KMnO_4$].

^{1}H NMR (500 MHz, $CDCl_3$): δ [ppm] = 2.99 (dd, ^2J = 16.1 Hz, ^3J = 3.5 Hz, 1H, H^a-13), 3.19 (dd, ^3J = 16.1 Hz, ^3J = 11.8 Hz, 1H, H^b-13), 4.31 (d, ^2J = 17.0 Hz, 1H, H^a-8), 4.86 (dd, ^3J = 11.8 Hz, ^3J = 3.5 Hz, 1H, H-13a), 5.52 (d, ^2J = 17.0 Hz, 1H, H^b-8), 6.73 (dd, ^3J = 7.9 Hz, ^4J = 1.1 Hz, 1H, H-4), 7.00 (*virt.* td, $^3J \approx {}^3J$ = 7.5 Hz, ^4J = 1.1 Hz, 1H, H-2), 7.10 – 7.24 (m, 6H, H-1, H-3, H-9, H-10, H-11, H-12), 7.48 (bs, 1H, NH).

^{13}C NMR (126 MHz, $CDCl_3$): δ [ppm] = 152.7 (C-6), 135.5 (C-4a), 133.5 (C-8a†), 133.4 (C-12a†), 129.2 (C-12), 128.8 (C-3), 126.8 (C-10‡), 126.7 (C-11‡), 126.4 (C-9), 125.8 (C-1), 122.4 (C-2), 120.4 (C-4a), 114.0 (C-4), 55.70, 45.14, 37.35.

†,‡ assignment is interconvertible

HRMS (+ESI): calc. for $C_{16}H_{15}N_2O$ $[M+H]^+$: 251.1179; found: 251.1176.

IR (ATR): $\tilde{\nu}$ [cm^{-1}] = 3321 (w, N–H), 2914 (w, C–H), 2898 (w, C–H), 1656 (s, C=O), 1606 (m, C=C), 1582 (m, C=C), 1469 (m), 1281 (m), 1110 (m), 842 (w), 765 (s, C–H), 737 (vs, C–H), 722 (s, C–H), 713 (s), 704 (m).

m.p. = 228-230 °C.

Specific rotation: α_D^{25}: 57.5 (c = 1.0 $CHCl_3$) [96% *ee*].

Chiral HPLC: 96% ee [©CHIRALPAK IC, 20 °C, 50% ^{i}PrOH/*n*-heptane, 1mL/min, 210 nm, t_R = 9.1 min (minor), 10.5 min (major)].

*5.1.25. 9,9-Dimethyl-5,8,9,10,11,11a-hexahydro-6H-pyrido[1,2-c]quinazolin-6-one (**5ac**)*

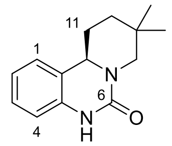

Following GP 4, with dilute concentration (10 mL, 10 mM), compound **5ac** was obtained as a colorless solid (18.9 mg, 82.1 µmol, 82%).

***R*f**: 0.24 (CH_2Cl_2/ac = 4/1) [UV, $KMnO_4$].

^{1}H NMR (500 MHz, $CDCl_3$): δ [ppm] = 0.98 (s, 3H, CH_3^a), 1.02 (s, 3H, CH_3^b), 1.53 (*virt.* td, $^2J \approx {}^3J$ = 13.4 Hz, ^3J = 4.5 Hz, 1H, H^a-10), 1.63 (*virt.* ddt, ^2J = 13.4 Hz, ^3J =4.1 Hz, $^3J \approx {}^4J$ = 2.9 Hz, 1H, H^b-10), 1.83 (*virt.* ddt, ^2J = 13.4 Hz, ^3J = 4.5 Hz, $^3J \approx {}^3J$ = 2.9 Hz, 1H, H^a-11), 1.91 (*virt.* tdd, $^2J \approx {}^3J$ = 13.4 Hz, ^3J = 11.6 Hz, ^3J = 4.1 Hz, 1H, H^b-11), 2.45 (d, ^2J = 13.1 Hz, 1H, H^a-8), 4.28 (dd, ^2J = 13.1 Hz, ^4J = 2.9 Hz, 1H, H^b-8), 4.43 (dd, ^3J = 11.6 Hz, ^3J = 2.9 Hz, 1H, H-11a), 6.61 (dd, ^3J = 7.7 Hz, ^4J = 1.2 Hz, 1H, H-4), 6.93 (*virt.* td, $^3J \approx {}^3J$ = 7.7 Hz, ^4J = 1.2 Hz, 1H, H-2), 7.01 (bs, 1H, NH), 7.03 (d, ^3J = 7.7 Hz, 1H, H-1), 7.14 (*virt.* td. $^3J \approx {}^3J$ = 7.7 Hz, ^4J = 1.4 Hz, 1H, H-3).

^{13}C NMR (126 MHz, $CDCl_3$): δ [ppm] = 152.7 (C-6), 135.2 (C-4a), 128.4 (C-3), 125.7 (C-1), 122.2 (C-2), 120.7 (C-11b), 113.8 (C-4), 58.8 (C-11a), 54.4 (C-8), 38.4 (C-10), 31.5 (C-11), 30.9 (C-9), 29.0 (CH_3^a), 23.2 (CH_3^b).

HRMS (+ESI): calc. for $C_{14}H_{19}N_2O$ $[M+H]^+$: 231.1492; found: 231.1491.

IR (ATR): $\tilde{\nu}$ [cm^{-1}] = 3315 (w, N–H), 2952 (m, C–H), 2914 (m, C–H), 2865 (w, C–H), 1657 (s, C=O), 1600 (m, C=C), 1466 (s), 1298 (s), 1105 (m), 801 (m), 748 (vs, C–H), 723 (m, C–H), 694 (m).

m.p. = >230 °C.

Specific rotation: α_D^{25}: 68.1 (c = 1.0 $CHCl_3$) [94% *ee*].

Chiral HPLC: 94% ee [©CHIRALPAK AD-H, 20 °C, 30% ^{i}PrOH/*n*-heptane, 1mL/min, 210 nm, t_R = 8.7 min (major), 12.5 min (minor)].

*5.1.26. 8,9,14,14a-Tetrahydrobenzo[4,5]azepino[1,2-c]quinazolin-6(5H)-one (**5ba**)*

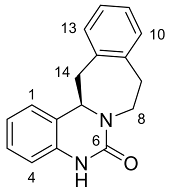

Following GP 4, compound **5ba** was obtained as a colorless solid (23.5 mg, 88.9 μmol, 89%).

***R*f**: 0.44 (CH_2Cl_2/ac = 4/1) [UV, $KMnO_4$].

^{1}H NMR (500 MHz, $CDCl_3$): δ [ppm] = 2.75 – 2.80 (m, 2H, H^a-8, H^a-14), 2.90 (ddd, ^2J = 15.4 Hz, ^3J = 5.4 Hz, ^3J = 0.9 Hz, 1H, H^a-9), 3.27 (ddd, ^2J = 15.4 Hz, ^3J = 11.3 Hz, ^3J = 2.0 Hz, 1H, H^b-9), 3.54 (dd, ^2J = 15.1 Hz, ^3J = 10.2 Hz, 1H, H^b-14), 4.56 (d, ^3J = 10.2 Hz, 1H, H-14a), 4.72 (ddd, ^2J = 13.9 Hz, ^3J = 5.4 Hz, ^3J = 2.0 Hz, 1H, H^b-8), 6.72 (dd, ^3J = 8.1 Hz, ^4J = 1.1 Hz, 1H, H-4), 7.01 (*virt.* td, $^3J \approx {}^3J$ = 7.5 Hz, ^4J = 1.2 Hz, 1H, H-2), 7.12 (dd, ^3J = 7.5 Hz, ^4J = 1.4 Hz, 1H, H-1), 7.15 – 7.18 (m, 5H, H-3, H-10, H-11. H-12, H-13), 7.29 (bs, 1H, NH).

^{13}C NMR (126 MHz, $CDCl_3$): δ [ppm] = 153.5 (C-6), 141.2 (C-9a), 139.5 (C-13a), 135.6 (C-4a), 130.3 (C-10†), 130.3 (C-13†), 128.5 (C-3), 127.2 (C-11‡), 126.7 (C-12‡), 126.0 (C-1), 122.6 (C-2), 122.5 (C-4a), 113.7 (C-4), 61.5 (C-14a), 47.7 (C-14), 46.6 (C-8), 38.0 (C-9).

†,‡ assignment is interconvertible

HRMS (+ESI): calc. for $C_{17}H_{17}N_2O$ $[M+H]^+$: 265.1335; found: 265.1334.

IR (ATR): $\tilde{\nu}$ [cm^{-1}] = 3199 (w, N–H), 2914 (w, C–H), 1659 (s, C=O), 1603 (m, C=C), 1508 (w, C=C), 1460 (m), 1300 (m), 1103 (w), 813 (w), 750 (vs, C–H), 693 (m).

m.p. = 108-110 °C.

Specific rotation: α_D^{25}: 87.3 (c = 1.0 $CHCl_3$) [75% *ee*].

Chiral HPLC: 75% ee [©CHIRALPAK AD-H, 20 °C, 30% ^{i}PrOH/*n*-heptane, 1mL/min, 210 nm, t_R = 7.8 min (major), 22.0 min (minor)].

*5.1.27. 8,9,15,15a-Tetrahydro-[1,3]dioxolo[4,5:4,5]benzo[1,2:4,5]azepino[1,2-c]quinazolin-6(5H)-one (**5bb**)*

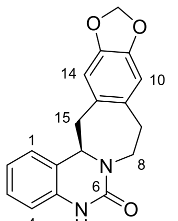

Following GP 4, compound **5bb** was obtained as a colorless solid (31.5 mg; corrected without residual CH_2Cl_2: 30.1 mg, 97.6 μmol, 98%).

***R*f**: 0.51 (CH_2Cl_2/ac = 4/1) [UV, $KMnO_4$].

^{1}H NMR (500 MHz, $CDCl_3$): δ [ppm] = 2.65 (dd, ^2J = 15.2 Hz, ^3J = 1.2 Hz, 1H, H^a-15), 2.74 (dd, ^2J = 13.9 Hz, ^3J = 11.0 Hz, 1H, H^a-8), 2.78 (dd, ^2J = 15.7 Hz, ^3J = 5.7 Hz, 1H, H^a-9), 3.17 (ddd, ^2J = 15.7 Hz, ^3J = 11.0 Hz, ^3J = 2.0 Hz, 1H, H^b-9), 3.44 (dd, ^2J = 15.2 Hz, ^3J = 10.0 Hz, 1H, H^b-15), 4.52 (d, ^3J = 10.0 Hz, 1H, H-15a), 4.68 (ddd, ^2J = 13.9 Hz, ^3J = 5.7 Hz, ^3J = 2.0 Hz, 1H, H^b-8), 5.93 (d, ^2J = 1.5 Hz, 1H, H^a-12), 5.95 (d, ^2J = 1.50 Hz, 1H, H^b-12), 6.64 (s, 1H, H-14), 6.68 – 6.69 (m, 2H, H-4, H-10), 6.92 (bs, 1H, NH), 7.00 (*virt.* td, $^3J \approx {}^3J$ = 7.6 Hz, ^4J = 1.1 Hz, 1H, H-2), 7.11 (dd, ^3J = 7.6 Hz, ^4J = 1.4 Hz, 1H, H-1), 7.19 (*virt.* td, $^3J \approx {}^3J$ = 7.6 Hz, ^4J = 1.4 Hz, 1H, H-3).

^{13}**C NMR** (126 MHz, $CDCl_3$): δ [ppm] = 153.1 (C-6), 146.3 (C-10a), 145.9 (C-13a), 135.5 (C-4a), 134.6 (C-9a), 132.7 (C-14a), 128.5 (C-3), 126.1 (C-1), 122.7 (C-2), 122.5 (C-15b), 113.6 (C-4), 110.8 (C-10†), 110.8 (C-14†), 101.2 (C-12), 61.8 (C-15a), 47.4 (C-15), 46.8 (C-8), 37.7 (C-9).

† assignment is interconvertible

HRMS (+ESI): calc. for $C_{18}H_{17}N_2O_3$ $[M+H]^+$: 309.1234; found: 309.1233.

IR (ATR): $\tilde{\nu}$ [cm^{-1}] = 3191 (w, N–H), 2991 (w, C–H), 2899 (w, C–H), 1661 (s, C=O), 1606 (m, C=C), 1502 (m, C=C), 1471 (s), 1268 (s, C–O), 1165 (m), 1041 (m), 856 (m), 796 (m), 744 (vs, C–H), 697 (m).

m.p. = >230 °C.

Specific rotation: α_D^{25}: 40.1 (c = 1.0 $CHCl_3$) [78% *ee*].

Chiral HPLC: 78% ee [©CHIRALPAK IA, 20 °C, 10% ^{i}PrOH/*n*-heptane, 1mL/min, 210 nm, t_R = 23.0 min (major), 40.6 min (minor)].

*5.1.28. 11,12-Dimethoxy-8,9,14,14a-tetrahydrobenzo[4,5]azepino[1,2-c]quinazolin-6(5H)-one (**5bc**)*

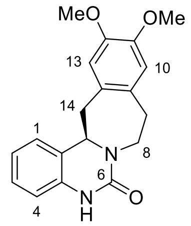

Following GP 4, compound **5bc** was obtained as a colorless solid (31.7 mg, 97.7 µmol, 98%).

***R*f**: 0.38 (CH_2Cl_2/ac = 4/1) [UV, $KMnO_4$].

^{1}**H NMR** (500 MHz, $CDCl_3$): δ [ppm] = 2.66 (dd, ^2J = 15.2 Hz, ^3J = 1.7 Hz, 1H, H^a-14), 2.78 (ddd, ^2J = 13.6 Hz, ^3J = 11.0 Hz, ^3J = 0.9 Hz, 1H, H^a-8), 2.81 (ddd, ^2J = 15.0 Hz, ^3J = 5.7 Hz, ^3J = 0.9 Hz, 1H, H^a-9), 3.23 (ddd, ^2J = 15.0 Hz, ^3J = 11.0 Hz, ^3J = 2.0 Hz, 1H, H^b-9), 3.48 (dd, ^2J = 15.2 Hz, ^3J = 10.3 Hz, 1H, H^b-14), 3.87 (s, 3H, C-11-OCH_3†), 3.89 (s, 3H, C-12-OCH_3†), 4.56 (d, ^3J = 10.3 Hz, 1H, H-14a), 4.69 (ddd, ^2J = 13.6 Hz, ^3J = 5.7 Hz, ^3J = 2.0 Hz, 1H, H^b-8), 6.66 (s, 1H, H-13), 6.71 – 6.73 (m, 2H, H-4, H-10), 7.01 (*virt.* td, $^3J \approx {}^3J$ = 7.6 Hz, ^4J = 1.1 Hz, 1H, H-2), 7.15 (dd, ^3J = 7.6 Hz, ^4J = 1.4 Hz, 1H, H-1), 7.18 – 7.22 (m, 2H, H-3, NH).

† assignment is interconvertible

^{13}**C NMR** (126 MHz, $CDCl_3$): δ [ppm] = 153.4 (C-6), 147.3 (C-11), 146.9 (C-12), 135.5 (C-8a), 133.4 (C-9a), 131.4 (C-13a), 128.5 (C-7), 126.1 (C-5), 122.6 (C-6), 122.5 (C-14b), 114.1 (C-13), 113.9 (C-4†), 113.7 (C-10†), 61.8 (C-14a), 56.2 (C-11-OCH_3‡), 56.1 (C-12-OCH_3‡), 47.3 (C-14), 47.0 (C-8), 37.6 (C-9).

†,‡ assignment is interconvertible

HRMS (+ESI): calc. for $C_{19}H_{21}N_2O_3$ $[M+H]^+$: 325.1547; found: 325.1546.

IR (ATR): $\tilde{\nu}$ [cm^{-1}] = 3314 (w, N–H), 2937 (w, C–H), 2907 (w, C–H), 2836 (w, C–H), 1653 (vs, C=O), 1603 (m, C=C), 1519 (m, C=C), 1464 (vs), 1304 (s, C–O), 1232 (s, C–O), 1164 (m), 1100 (s), 808 (m), 789 (m), 757 (vs, C–H), 736 (m), 726 (m, C–H), 705 (m).

m.p. = >230 °C.

Specific rotation: α_D^{25}: 71.8 (c = 1.0 $CHCl_3$) [74% *ee*].

Chiral HPLC: 74% ee [©CHIRALPAK IA, 20 °C, 10% ^{i}PrOH/*n*-heptane, 1mL/min, 210 nm, t_R = 20.6 min (major), 40.8 min (minor)].

6. Deuteration Experiments

6.1. Substrate Synthesis

*6.1.1. (2-Aminophenyl)methan-d_2-ol (**SI-14-d_2**)*

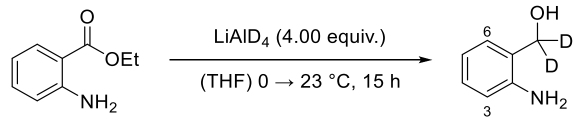

Ethyl anthranilate (1.77 mL, 1.98 g, 12.0 mmol, 1.00 equiv.) was added dropwise to a suspension of $LiAlD_4$ (2.01 g, 48.0 mmol, 4.00 equiv.) in THF (48 mL, 250 mM) at 0 °C. The reaction mixture was warmed to room temperature and stirred for 15 h. The mixture was quenched by sequential addition of H_2O (0.26 mL), aq. NaOH (1 M, 0.58 mL), and H_2O (0.78 mL) again, and stirred vigorously for 1 h. The solids were filtered and washed with EtOAc (3 × 20 mL). After removal of all volatiles, the deuterated alcohol **SI-14**-d_2 (1.50 g, 12.0 mmol, >99%., >99% D) was obtained as a colorless liquid, which was used without further purification.

^{1}H NMR (500 MHz, $CDCl_3$): δ [ppm] = 6.72 – 6.75 (m, 2H), 7.07 – 7.09 (m, 1H), 7.14 (*virt.* td, $^3J \approx {}^3J$ = 7.6 Hz, ^4J = 1.6 Hz, 1H).

^{13}C NMR (126 MHz, $CDCl_3$): δ [ppm] = 168.5 (C-1, C-3), 133.8 (C-5, C-6), 132.3 (C-3a, C-7a), 123.3 (C-4, C-7), 102.7 (C-1''), 65.1 (C-4'', C-5''), 33.3 (C-1'), 32.3 (C-2').

Spectral data matched those reported in the literature.[19]

*6.1.2. 2-Aminobenzaldehyde-d_1 (**SI-15-d_1**)*

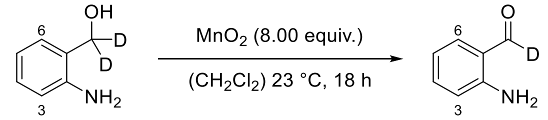

MnO_2 (8.35 g, 96.0 mmol, 8.00 equiv.) was added in one portion to a solution of **Si-14**-d_2, (1.50 g, 12.0 mmol, 1.00 equiv.) and the resulting suspension was stirred at room temperature for 18 h. The solids were filtered off, and the solvents were removed under reduced pressure to obtain the deuterated aldehyde **SI-15**-d_1 (931 mg, 7.62 mmol, 64%, >99%D) as a pale-yellow solid, which was directly used in the subsequent steps without analyzation.

*6.1.3. 3-(3,3-Diethoxypropyl)-3,4-dihydroquinazolin-2(1H)-one-4,4-d_2 (**SI-8a-d_2**)*

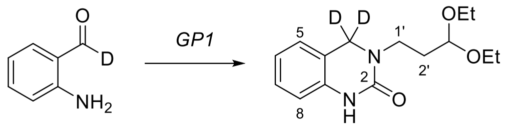

Following GP1 starting from **SI-15**-d_1 (900 mg, 7.37 mmol, 1.00 equiv.) and using $NaBD_4$ as reductant, deuterated quinazolinone **SI-8a**-d_2 (1.32 g, 4.71 mmol, 76%, >99% D) was obtained as a pale-yellow solid.

***R*f**: 0.35 (CH_2Cl_2/ac = 4/1) [UV, $KMnO_4$].

^{1}H NMR (500 MHz, $CDCl_3$): δ [ppm] = 1.20 (d, ^3J = 7.1 Hz, 6H, CH_3), 1.95 – 1.99 (m, 2H, H-2'), 3.49 – 3.55 (m, 4H, OCH_2^a, C-1'), 3.67 (dq, ^2J = 9.4 Hz, ^3J = 7.1 Hz, 2H, OCH_2^b), 4.59 (t, ^3J = 5.6 Hz, 1H, H-3'), 6.68 (dd, 7.7 Hz, ^4J = 1.1 Hz, 1H, H-8), 6.92 (*virt.* td, $^3J \approx {}^3J$ = 7.7 Hz, ^4J = 1.1 Hz, 1H, H-6), 7.03 (dd, ^3J = 7.7 Hz, ^4J = 1.4 Hz, 1H, H-5), 7.15 (*virt.* td, $^3J \approx {}^3J$ = 7.7 Hz, ^4J = 1.4 Hz, 1H, H-7), 7.33 (bs, 1H, NH).

^{13}C NMR (126 MHz, $CDCl_3$): δ [ppm] = 154.3 (C-2), 137.1 (C-8a), 128.3 (C-7), 125.6 (C-5), 122.0 (C-6), 117.7 (C-4a), 113.5 (C-8), 101.4 (C-3'), 61.7 (OCH_2), 48.4 (C-4), 43.6 (C-1'), 31.4 (C-2'), 15.5 (CH_3).

HRMS (+ESI): calc. for $C_{15}H_{20}D_2N_2NaO_3$ $[M+Na]^+$: 303.1648; found: 303.1645.

IR (ATR): $\tilde{\nu}$ [cm^{-1}] = 3425 (w, N–H), 2974 (w, C–H), 2930 (w, C–H), 2878 (w, C–H), 1668 (m, C=O), 1603 (m, C=C), 1529 (w, C=C), 1511 (w, C=C), 1457 (m), 1122 (s, C–O), 1114 (s), 1056 (vs), 1021 (m), 839 (w), 750 (vs, C–H), 722 (w. C–H).

m.p. = 57-59 °C.

*6.1.4. N'-(3-(2-Oxo-1,4-dihydroquinazolin-3(2H)-yl-4,4-d_2)propylidene)-4-methylbenzenesulfonohydrazide (**4a-**d_2)*

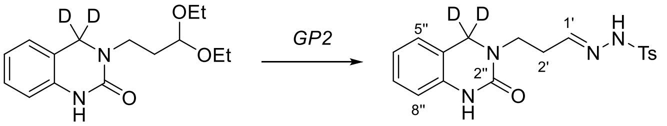

Following GP2 starting from **SI-8a**-d_2 (1.17 g, 4.19 mmol, 1.00 equiv.), deuterated hydrazone **4a**-d_2 (292 mg, 780 µmol, 19%, >99% D) was obtained as a colorless solid.

^{1}H NMR (500 MHz, DMSO-d_6): δ [ppm] = 2.28 (s, 3H, CH_3), 2.38 (td, ^3J = 6.9 Hz, ^3J = 5.3 Hz, 2H, H-2'), 3.39 (t, ^3J = 6.9 Hz, 2H, H-3'), 6.75 (dd, ^3J = 7.7 Hz, ^4J = 1.2 Hz, 1H, H-8''), 6.86 (*virt.* td, $^3J \approx {}^3J$ = 7.7 Hz, ^4J = 1.2 Hz, 1H, H-6''), 7.00 (dd, ^3J = 7.7 Hz, ^4J = 1.5 Hz, 1H, H-5''), 7.12 (*virt.* td, $^3J \approx {}^3J$ = 7.7 Hz, ^4J = 1.5 Hz, 1H, H-7''), 7.27 – 7.29 (m, 3H, H-3, H-1'), 7.62 – 7.65 (m, 2H, H-2), 9.12 (bs, 1H, NH), 11.04 (bs, 1H, NNH).

^{13}C NMR (126 MHz, DMSO-d_6): δ [ppm] = 153.5 (C-2''), 149.5 (C-1'), 143.2 (C-4), 137.7 (C-8a''), 136.3 (C-1), 129.6 (C-3), 127.8 (C-7''), 127.1 (C-2), 125.5 (C-5''), 121.0 (C-6''), 117.7 (C-4a''), 113.2 (C-8''), 47.2 (C-4''), 43.3 (C-3'), 30.2 (C-2'), 21.0 (CH_3).

HRMS (+ESI): calc. for $C_{18}H_{19}D_2N_4O_3S$ $[M+H]^+$: 375.1454; found: 375.1454.

IR (ATR): $\tilde{\nu}$ [cm^{-1}] = 3351 (w, N–H), 2866 (w, C–H), 2842 (w, C–H), 1648 (s, C=O), 1602 (m, C=C), 1511 (w, C=C), 1444 (s), 1333 (s, RSO_2N), 1163 (vs, RSO_2N), 1096 (m), 1058 (s), 817 (m), 770 (m, C–H), 754 (vs, C–H), 748 (s, C–H), 734 (m, C–H), 706 (m).

m.p. = >226 °C (decomposition).

6.2. KIE Measurements

6.2.1. Theoretical Background

The KIE of a given reaction is defined as

$$KIE = \frac{k_H}{k_D} = \frac{\frac{d\,[\mathbf{5a}]}{dt}}{\frac{d\,[\mathbf{5a}\text{-}d_2]}{dt}}$$

wherein k_H is the relative rate constant of the substrate **5a** and k_D the relative rate constant of the substrate **5a**-d_2 at low conversions and under identical reaction conditions. For this experiment, conversions lower than 20% were chosen to be suitable for the determination and a linear relationship between concentration and time was observed. Data triplets of each rate constant were measured and the mean and standard deviation were calculated according to:

$$\bar{k} = \frac{1}{n}\sum_{i=1}^{n} k_i$$

$$\sigma_{\bar{k}} = \sqrt{\frac{1}{n(n-1)}\sum_{i=1}^{n}\left(k_i - \bar{k}\right)^2}$$

The standard error of the obtained KIE can be determined according to Gaussian law of error propagation using the following formula:

$$\sigma_{\overline{KIE}} = \sqrt{\left(\frac{\sigma_{\overline{k_H}}}{\overline{k_D}}\right)^2 + \left(\frac{-\overline{k_H}}{\overline{k_D}^2}\sigma_{\overline{k_D}}\right)^2}$$

6.2.2. Experimental Procedure for the Determination of the KIE

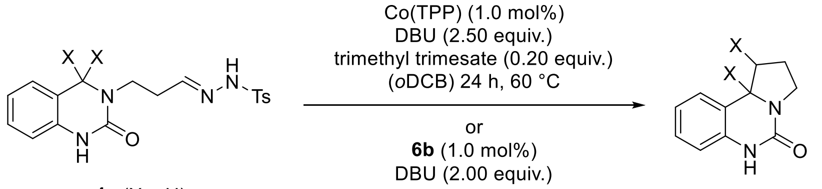

4a (X = H)
4a-d_2 (X = D)

5a (X = H)
5a-d_2 (X = D)

A Schlenk tube was equipped with the corresponding hydrazone (100 µmol, 1.00 equiv.), catalyst (1.00 µmol, 1.0 mol%), and trimethyl trimesate (5.04 mg, 20.0 µmol, 0.20 equiv.) as internal standard, and the atmosphere was exchanged (3×). The solids were dissolved in *o*DCB (5.0 mL) and DBU was added dropwise to the solution. The tube was sealed, and the reaction mixture was stirred at 60 °C. At intervals of 30 min, aliquots (100 µL) of the reaction mixture were removed and immediately diluted with $CDCl_3$ (300 µL) to stop the reaction. Quantification of the product was performed using NMR spectroscopy (500 MHz, relaxation delay = 10 s), referencing the integrals to trimethyl trimesate (δ = 8.92 ppm) as internal standard.

6.2.3. KIE Experiments with Co(TPP)

Table 1 *Product formation of* ***5a*** *with Co(TPP) at different reaction times.*

Entry	time [h]	Y(**5a**) 1st run	Y(**5a**) 2nd run	Y(**5a**) 3rd run
1	0.5	0.045	0.038	0.036
2	1.0	0.071	0.078	0.078
3	1.5	0.111	0.111	0.118
4	2.0	0.137	0.143	0.141
5	2.5	0.174	0.158	0.169

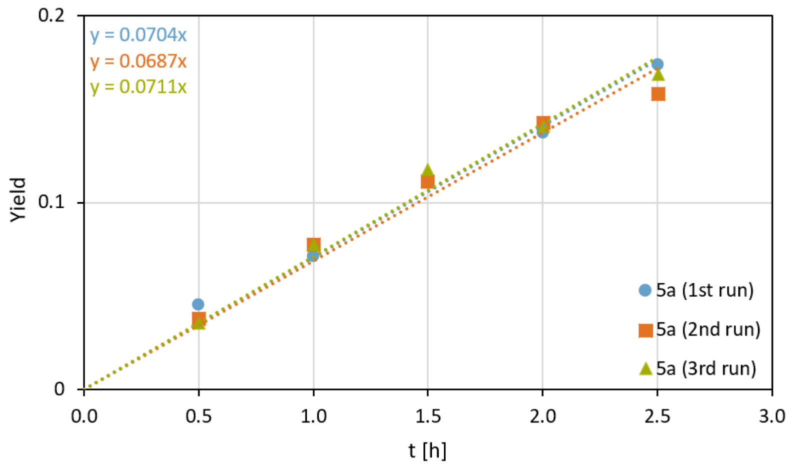

Table 2 *Product formation of* ***5a**-d_2 *with Co(TPP) at different reaction times.*

Entry	time [h]	Y(**5a**-d_2) 1st run	Y(**5a**-d_2) 2nd run	Y(**5a**-d_2) 3rd run
1	0.5	0.033	0.026	0.031
2	1.0	0.065	0.058	0.059
3	1.5	0.095	0.083	0.080
4	2.0	0.132	0.119	0.119
5	2.5	0.147	0.152	0.149
6	3.0	0.177	0.181	0.180

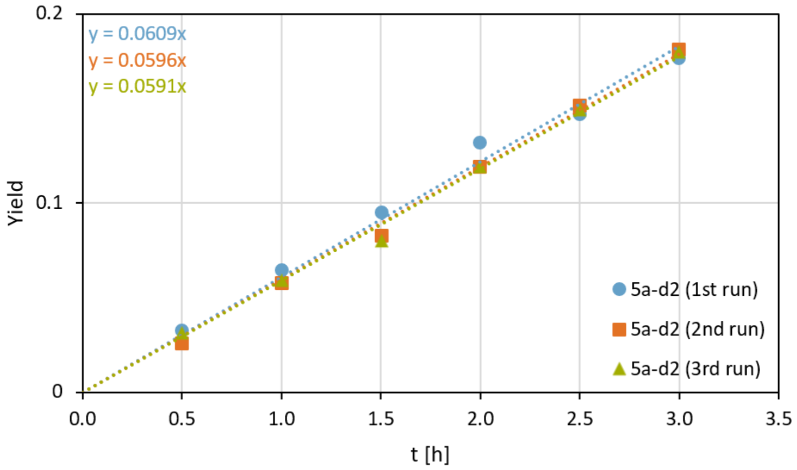

According to the stated equations, the KIE of the reaction with Co(TPP) was calculated to be

$$KIE = \frac{\overline{k_H}}{\overline{k_D}} = \frac{0.0701}{0.0599} = 1.17 \pm\ 0.03$$

with the standard error of the mean σ = 0.016, and a 95% confidence interval of ±0.03.

*6.2.4. KIE Experiments with Chiral Catalyst **6b**:*

Table 3 *Product formation of **5a** with chiral catalyst **6b** at different reaction times.*

Entry	time [min]	Y(**5a**) 1st run	Y(**5a**) 2nd run	Y(**5a**) 3rd run
1	0.5	0.03	0.022	0.025
2	1.0	0.059	0.059	0.051
3	1.5	0.114	0.084	0.091
4	2.0	0.139	0.136	0.129
5	2.5	0.176	0.152	0.165

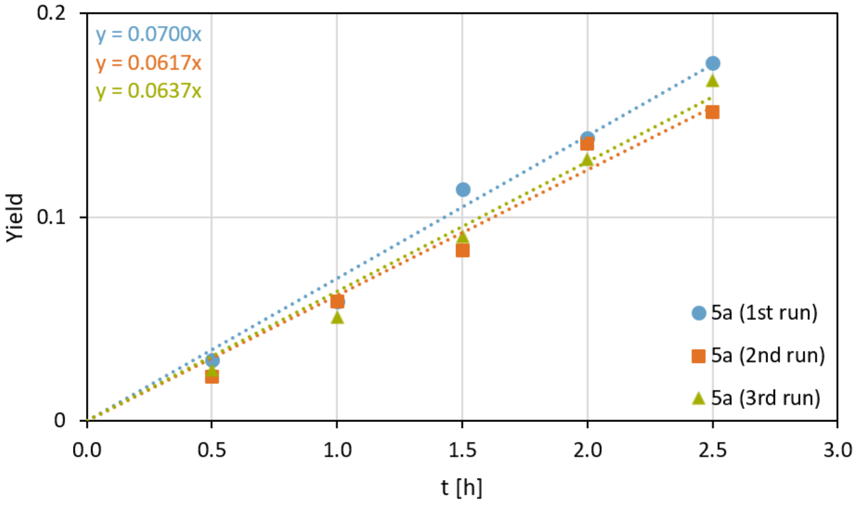

Table 4 *Product formation of **5a**-d_2 with chiral catalyst **6b** at different reaction times.*

Entry	time [min]	Y(**5a**-d_2) 1st run	Y(**5a**-d_2) 2nd run	Y(**5a**-d_2) 3rd run
1	0.5	0.023	0.015	0.018
2	1.0	0.043	0.043	0.044
3	1.5	0.071	0.071	0.073
4	2.0	0.096	0.093	0.095
5	2.5	0.131	0.118	0.125
6	3.0	0.154	0.154	0.047
7	3.5	0.180	0.174	0.180

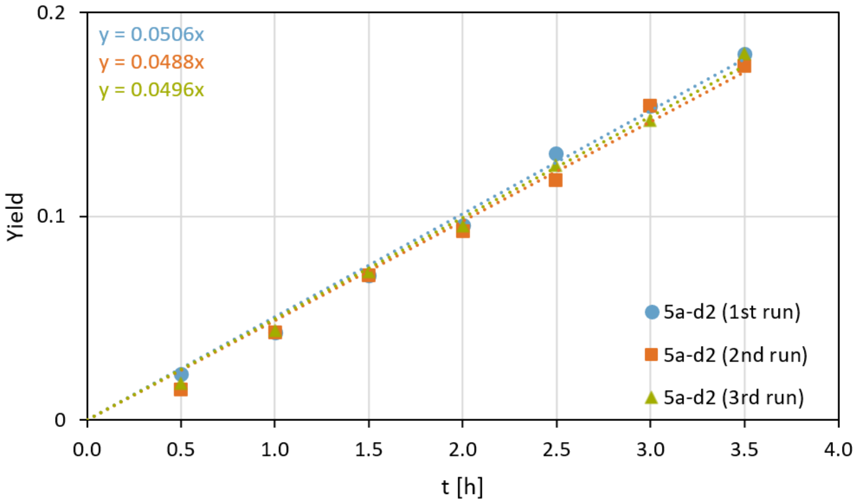

According to the stated equations, the KIE of the reaction with chiral catalyst **6b** was calculated to be

$$KIE = \frac{\overline{k_H}}{\overline{k_D}} = \frac{0.0651}{0.0497} = 1.31 \pm 0.11$$

with the standard error of the mean σ = 0.053, and a 95% confidence interval of ±0.11.

7. Crystallographic Data

SC-XRD structure report for compound 5b

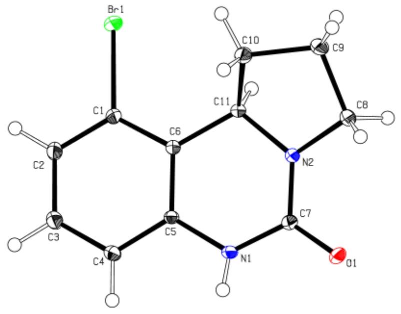

Figure 1: ORTEP representation of the solid-state structure of compound **5b** (C = black, N = blue, O = red and Br = green) shown with 50 % probability displacement ellipsoids.

A colourless, fragment-shaped crystal of $C_{11}H_{11}BrN_2O$ coated with perfluorinated ether and fixed on top of a Kapton micro sampler was used for X-ray crystallographic analysis. The X-ray intensity data were collected at 100(2) K on a Bruker D8 VENTURE three-angle diffractometer with a TXS rotating anode with MoK_α radiation (λ=0.71073 Å) using APEX4.[1] The diffractometer was equipped with a Helios optic monochromator, a Bruker PHOTON III detector, and an Oxford Cryostreamlow temperature device.

A matrix scan was used to determine the initial lattice parameters. All data were integrated with the Bruker SAINT V8.40B software package using a narrow-frame algorithm and the reflections were corrected for Lorentz and polarisation effects, scan speed, and background.[2] The integration of the data using a orthorhombic unit cell yielded a total of 66069 reflections within a 2θ range [°] of 6.07 to 66.27 (0.65 Å), of which 3851 were independent. Data were corrected for absorption effects including odd and even ordered spherical harmonics by the multi-scan method (SADABS 2016/2).[3] Space group assignment was based upon systematic absences, E statistics, and successful refinement of the structure.

The structure was solved by direct methods using SHELXT and refined by full-matrix least-squares methods against F^2 by minimizing $\Sigma w(F_o^2 - F_c^2)^2$ using SHELXL in conjunction with SHELXLE.[4-6] All non-hydrogen atoms were refined with anisotropic displacement parameters. Hydrogen atoms were refined isotropically on calculated positions using a riding model with their U_{iso} values constrained to 1.5 times the U_{eq} of their pivot atoms for terminal sp^3 carbon atoms and a C–H distance of 0.98 Å. Non-methyl hydrogen atoms were refined using a riding model with methylene, aromatic, and other C–H distances of 0.99 Å, 0.95 Å, and 1.00 Å, respectively, and U_{iso} values constrained to 1.2 times the U_{eq} of their pivot atoms.

Neutral atom scattering factors for all atoms and anomalous dispersion corrections for the non-hydrogen atoms were taken from International Tables for Crystallography.[7] Supplementary crystallographic data reported in this paper have been deposited with the Cambridge Crystallographic Data Centre (CCDC 2497766) and can be obtained free of charge from The Cambridge Crystallographic Data Centre via www.ccdc.cam.ac.uk/structures.[8] This report and the CIF file were generated using FinalCif.[9]

Table 1. Crystal data and structure refinement for compound 5b.

CCDC number	2497766
Empirical formula	$C_{11}H_{11}BrN_2O$
Formula weight	267.13
Temperature [K]	100(2)
Crystal system	orthorhombic
Space group (number)	$P2_12_12_1$ (19)
a [Å]	6.4977(5)
b [Å]	7.0484(6)
c [Å]	22.1436(18)
α [°]	90
β [°]	90
γ [°]	90
Volume [Å^3]	1014.14(14)
Z	4
ρ_{calc} [gcm^{-3}]	1.750
μ [mm^{-1}]	4.025
F(000)	536
Crystal size [mm^3]	0.057×0.082×0.093
Crystal colour	colourless
Crystal shape	fragment
Radiation	MoK_α (λ=0.71073 Å)
2θ range [°]	6.07 to 66.27 (0.65 Å)
Index ranges	$-9 \le h \le 9$<br>$-10 \le k \le 10$<br>$-34 \le l \le 34$
Reflections collected	66069
Independent reflections	3851<br>R_{int} = 0.0527<br>R_{sigma} = 0.0238
Completeness to θ = 25.242°	99.9
Data / Restraints / Parameters	3851 / 0 / 142
Goodness-of-fit on F^2	1.054
Final R indexes [I≥2σ(I)]	R_1 = 0.0249<br>wR_2 = 0.0663
Final R indexes [all data]	R_1 = 0.0269<br>wR_2 = 0.0676
Largest peak/hole [eÅ^{-3}]	1.46/−0.43

Flack X parameter	0.034(3)

Atom	*x*	*y*	*z*	U_{eq}
Br1	0.37779(4)	0.15232(3)	0.74079(2)	0.02136(7)
O1	0.8288(3)	0.5770(2)	0.50196(8)	0.0174(3)
N1	0.5489(3)	0.6220(3)	0.56193(9)	0.0142(3)
H5	0.501(6)	0.700(5)	0.5401(17)	0.021
N2	0.7712(3)	0.3718(3)	0.57906(8)	0.0136(3)
C2	0.1371(4)	0.4541(3)	0.69684(10)	0.0168(3)
H2	0.045500	0.418206	0.728263	0.020
C1	0.3154(3)	0.3508(3)	0.68597(9)	0.0139(3)
C3	0.0955(3)	0.6114(3)	0.66070(10)	0.0181(4)
H3	−0.026680	0.682611	0.667120	0.022
C4	0.2311(3)	0.6645(3)	0.61549(10)	0.0163(4)
H4	0.202074	0.772183	0.591131	0.020
C5	0.4106(3)	0.5598(3)	0.60570(9)	0.0111(3)
C6	0.4545(3)	0.3968(3)	0.63985(9)	0.0116(3)
C7	0.7238(3)	0.5252(3)	0.54562(10)	0.0126(3)
C8	0.9227(3)	0.2341(3)	0.55615(11)	0.0179(4)
H8A	1.064842	0.274683	0.565750	0.021
H8B	0.909602	0.217997	0.511900	0.021
C9	0.8674(4)	0.0499(3)	0.58952(11)	0.0193(4)
H9A	0.963264	0.027271	0.623522	0.023
H9B	0.873070	−0.060141	0.561761	0.023
C10	0.6469(4)	0.0812(3)	0.61274(11)	0.0174(4)
H10A	0.543679	0.056432	0.580831	0.021
H10B	0.616808	0.000539	0.648253	0.021
C11	0.6542(3)	0.2924(3)	0.62980(9)	0.0122(3)
H1	0.743(6)	0.300(5)	0.6674(15)	0.018

U_{eq} is defined as 1/3 of the trace of the orthogonalized U_{ij} tensor.

Table 1. Anisotropic displacement parameters (Å^2) for compound 5b. The anisotropic displacement factor exponent takes the form: $-2\pi^2[h^2(a^*)^2U_{11} + k^2(b^*)^2U_{22} + \ldots + 2hka^*b^*U_{12}]$

Atom	U_{11}	U_{22}	U_{33}	U_{23}	U_{13}	U_{12}
Br1	0.02190(10)	0.02237(11)	0.01980(10)	0.01081(8)	0.00613(8)	0.00454(9)
O1	0.0189(7)	0.0165(7)	0.0169(7)	0.0044(6)	0.0064(5)	0.0005(5)
N1	0.0161(7)	0.0124(8)	0.0142(7)	0.0047(6)	0.0029(6)	0.0034(6)
N2	0.0130(7)	0.0125(8)	0.0152(7)	0.0031(6)	0.0035(5)	0.0024(6)
C2	0.0134(8)	0.0206(9)	0.0164(8)	0.0022(7)	0.0023(7)	0.0011(8)
C1	0.0134(7)	0.0152(8)	0.0131(8)	0.0026(7)	0.0008(6)	0.0007(7)
C3	0.0156(9)	0.0209(10)	0.0178(9)	0.0023(7)	0.0023(7)	0.0039(7)
C4	0.0166(8)	0.0155(9)	0.0169(9)	0.0034(8)	0.0015(7)	0.0042(7)
C5	0.0124(8)	0.0103(7)	0.0106(7)	0.0013(6)	0.0002(6)	0.0011(6)
C6	0.0116(7)	0.0115(8)	0.0115(8)	0.0008(6)	0.0000(6)	0.0003(6)
C7	0.0134(8)	0.0117(8)	0.0127(8)	0.0005(7)	−0.0003(6)	−0.0008(7)
C8	0.0169(9)	0.0150(9)	0.0218(10)	0.0020(7)	0.0058(7)	0.0046(7)
C9	0.0212(9)	0.0137(8)	0.0230(10)	0.0012(7)	0.0049(9)	0.0051(8)
C10	0.0186(10)	0.0122(8)	0.0216(9)	0.0008(7)	0.0043(8)	0.0007(7)
C11	0.0126(8)	0.0112(7)	0.0129(8)	0.0025(6)	0.0004(6)	0.0010(6)

Table 2. Bond lengths and angles for compound 5b.

Atom–Atom	Length [Å]
Br1–C1	1.896(2)
O1–C7	1.238(3)
N1–C7	1.374(3)
N1–C5	1.392(3)
N1–H5	0.80(4)
N2–C7	1.346(3)
N2–C11	1.468(3)
N2–C8	1.473(3)
C2–C1	1.389(3)
C2–C3	1.394(3)
C2–H2	0.9500
C1–C6	1.402(3)
C3–C4	1.385(3)
C3–H3	0.9500
C4–C5	1.397(3)
C4–H4	0.9500
C5–C6	1.405(3)
C6–C11	1.508(3)
C8–C9	1.537(3)
C8–H8A	0.9900
C8–H8B	0.9900
C9–C10	1.538(3)
C9–H9A	0.9900
C9–H9B	0.9900
C10–C11	1.537(3)
C10–H10A	0.9900
C10–H10B	0.9900
C11–H1	1.01(3)
**Atom–Atom–Atom**	**Angle [°]**
C7–N1–C5	124.06(18)
C7–N1–H5	121(3)
C5–N1–H5	113(3)
C7–N2–C11	127.50(18)
C7–N2–C8	119.53(18)
C11–N2–C8	111.02(17)
C1–C2–C3	118.6(2)

C1–C2–H2	120.7
C3–C2–H2	120.7
C2–C1–C6	122.9(2)
C2–C1–Br1	117.00(15)
C6–C1–Br1	119.93(16)
C4–C3–C2	120.4(2)
C4–C3–H3	119.8
C2–C3–H3	119.8
C3–C4–C5	120.0(2)
C3–C4–H4	120.0
C5–C4–H4	120.0
N1–C5–C4	118.70(18)
N1–C5–C6	120.08(18)
C4–C5–C6	121.21(18)
C1–C6–C5	116.76(19)
C1–C6–C11	123.32(18)
C5–C6–C11	119.64(18)
O1–C7–N2	122.7(2)
O1–C7–N1	121.0(2)
N2–C7–N1	116.35(18)
N2–C8–C9	103.60(18)
N2–C8–H8A	111.0
C9–C8–H8A	111.0
N2–C8–H8B	111.0
C9–C8–H8B	111.0
H8A–C8–H8B	109.0
C8–C9–C10	104.89(18)
C8–C9–H9A	110.8
C10–C9–H9A	110.8
C8–C9–H9B	110.8
C10–C9–H9B	110.8
H9A–C9–H9B	108.8
C11–C10–C9	101.12(17)
C11–C10–H10A	111.5
C9–C10–H10A	111.5
C11–C10–H10B	111.5
C9–C10–H10B	111.5
H10A–C10–H10B	109.4
N2–C11–C6	111.87(16)

N2–C11–C10	101.37(16)
C6–C11–C10	118.86(17)
N2–C11–H1	108(2)
C6–C11–H1	110(2)
C10–C11–H1	106(2)

Table 3. Torsion angles for compound 5b.

**Atom–Atom–Atom–Atom**	**Torsion Angle [°]**
C3–C2–C1–C6	−0.6(3)
C3–C2–C1–Br1	174.48(18)
C1–C2–C3–C4	−0.9(3)
C2–C3–C4–C5	0.3(4)
C7–N1–C5–C4	−175.8(2)
C7–N1–C5–C6	5.4(3)
C3–C4–C5–N1	−176.9(2)
C3–C4–C5–C6	1.9(3)
C2–C1–C6–C5	2.6(3)
Br1–C1–C6–C5	−172.34(15)
C2–C1–C6–C11	176.5(2)
Br1–C1–C6–C11	1.6(3)
N1–C5–C6–C1	175.53(19)
C4–C5–C6–C1	−3.2(3)
N1–C5–C6–C11	1.4(3)
C4–C5–C6–C11	−177.4(2)
C11–N2–C7–O1	−177.1(2)
C8–N2–C7–O1	−14.5(3)
C11–N2–C7–N1	2.1(3)
C8–N2–C7–N1	164.72(19)
C5–N1–C7–O1	172.1(2)
C5–N1–C7–N2	−7.1(3)
C7–N2–C8–C9	−156.2(2)
C11–N2–C8–C9	9.1(2)
N2–C8–C9–C10	18.1(2)
C8–C9–C10–C11	−37.0(2)
C7–N2–C11–C6	3.9(3)
C8–N2–C11–C6	−159.97(18)
C7–N2–C11–C10	131.5(2)
C8–N2–C11–C10	−32.3(2)
C1–C6–C11–N2	−179.17(19)
C5–C6–C11–N2	−5.4(3)
C1–C6–C11–C10	63.2(3)
C5–C6–C11–C10	−123.0(2)
C9–C10–C11–N2	41.4(2)
C9–C10–C11–C6	164.39(18)

For crystallization, the major enantiomer E1 of **5b** was used (t_R = 11.8 min). For comparison the HPLC trace of the racemate (*rac*-**5b**, top left), the trace of the enantioselective cyclization reaction product (**5b**, top right) as well as the trace of the enantiomerically pure material of E1 (**5b**, bottom) used for crystallization are shown.

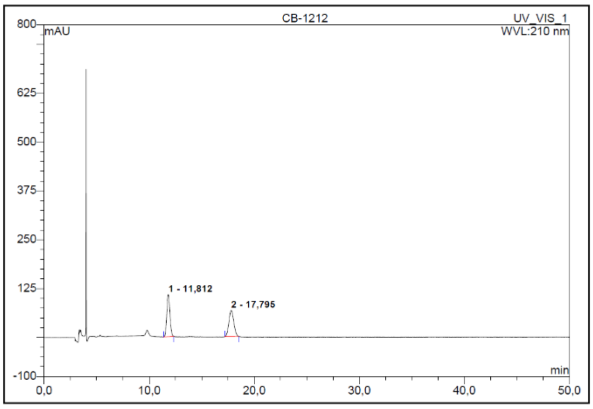

No.	Ret.Time min	Peak Name	Height mAU	Area mAU*min	Rel.Area %	Amount	Type
1	11,81	n.a.	107,798	37,279	50,69	n.a.	BMB
2	17,79	n.a.	67,175	36,264	49,31	n.a.	BMB
Total:			174,972	73,543	100,00	0,000	

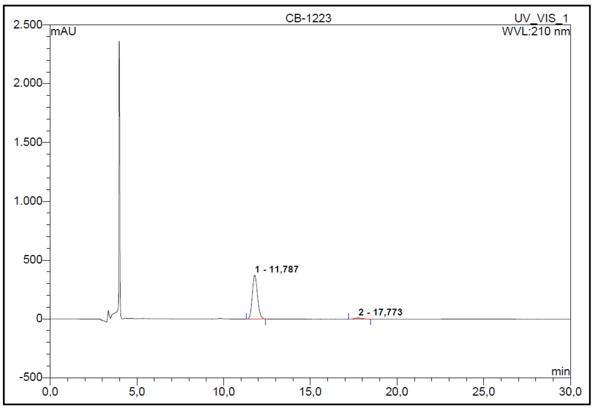

No.	Ret.Time min	Peak Name	Height mAU	Area mAU*min	Rel.Area %	Amount	Type
1	11,79	n.a.	373,687	130,206	95,53	n.a.	BMB
2	17,77	n.a.	11,708	6,092	4,47	n.a.	BMB*
Total:			385,395	136,298	100,00	0,000	

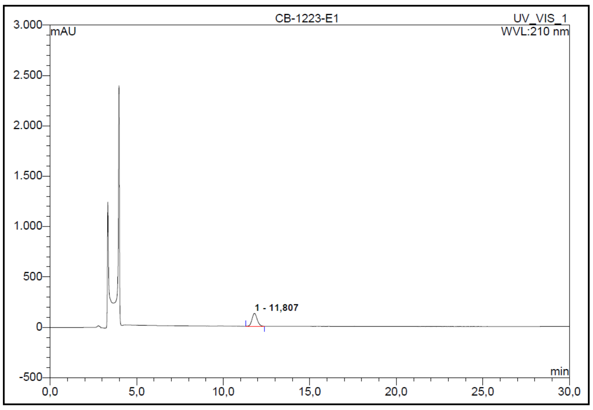

No.	Ret.Time min	Peak Name	Height mAU	Area mAU*min	Rel.Area %	Amount	Type
1	11,81	n.a.	127,387	43,873	100,00	n.a.	BMB
Total:			127,387	43,873	100,00	0,000	

References for this Section

[1] *APEX4 Suite of Crystallographic Software, Version 2021-10.0,* Bruker AXS Inc., Madison, Wisconsin, USA, **2021**.

[2] Bruker, *SAINT*, *V8.40B*, Bruker AXS Inc., Madison, Wisconsin, USA.

[3] L. Krause, R. Herbst-Irmer, G. M. Sheldrick, D. Stalke, *J. Appl. Cryst.* **2015**, *48*, 3–10, doi:10.1107/S1600576714022985.

[4] G. M. Sheldrick, *Acta Cryst.* **2015**, *A71*, 3–8, doi:10.1107/S2053273314026370.

[5] G. M. Sheldrick, *Acta Cryst.* **2015**, *C71*, 3–8, doi:10.1107/S2053229614024218.

[6] C. B. Huebschle, G. M. Sheldrick, B. Dittrich, *J. Appl. Cryst.* **2011**, *44*, 1281–1284, doi:10.1107/S0021889811043202.

[7] Ed. E. Prince, *International Tables for Crystallography Volume C, Mathematical, Physical and Chemical Tables*, International Union of Crystallography, Chester, England, **2006**, 500–502; 219–222; 193–199.

[8] C. R. Groom, I. J. Bruno, M. P. Lightfoot, S. C. Ward, *Acta Cryst.* **2016**, *B72*, 171–179, doi:10.1107/S2052520616003954.

[9] D. Kratzert, *FinalCif*, *V144*, https://dkratzert.de/finalcif.html.

8. Computational Studies

8.1. Methods

Density Functional Theory (DFT) calculations were performed with the Gaussian16 suite of programs (Revision C.01).[20] The M06L functional[21] (with density fitting) with additional D3 dispersion corrections by Grimme[22] was used on an ultrafine grid. Cobalt was described with the def2-TZVPD[23] basis set (including the corresponding pseudopotential), while def2-TZVP[24] was used for all other atoms. The nature of minima and transition states was confirmed by frequency calculations, i.e. the absence of imaginary frequencies or the presence of one negative frequency which corresponded to the respective process. These negative frequencies were followed in both directions to obtain starting material and product of the transition state. Finally, low-frequency entropy corrections by Grimme[25] were applied to all structures. All energies correspond to the gas-phase.

KIE predictions were obtained with the *Kinisot* software by the Paton group,[26] which is based on an earlier program by Rzepa.[27] The software was run with the Gaussian output files for complex **9** and transition state **TS1**, with a temperature of 298.15 and a vibrational scaling factor of 0.97.

Below, we provide energies (in Hartree) and coordinates of all relevant structures. The latter are also included in an accompanying xyz file.

8.2. Molecule Data

Complex 9 (doublet)

Charge: 0, Multiplicity: 2, NImag: 0

E = -4231.88938636 ht

G = -4231.06578006 ht

G (with entropy correction) = -4231.05339836 ht

C -6.82189100 0.99887000 -0.26484500

C -6.56142100 2.51908100 -0.20109200

C -5.71787500 0.37580000 0.56177700

N -5.03216300 1.35155600 1.17628200

H -4.17635100 1.13345000 1.69745700

C -5.35072300 2.68798800 0.73584000

C -0.48987300 -2.34110400 2.67333300

C -2.25437800 -0.60640100 2.39545200

C -1.52268400 -3.35116000 2.29117000

N -3.08138600 -1.57450400 1.90411800

O	*-2.60567200*	*0.57640600*	*2.43713500*
C	*-2.78356400*	*-2.92316000*	*1.88026200*
C	*-1.23864800*	*-4.70828800*	*2.27920300*
H	*-3.98034800*	*-1.26429400*	*1.52276100*
C	*-3.72978900*	*-3.84596400*	*1.43576500*
H	*-0.26349200*	*-5.04263500*	*2.61378400*
C	*-2.17101200*	*-5.63361200*	*1.83592000*
C	*-3.41642200*	*-5.19293700*	*1.40830200*
H	*-4.69879600*	*-3.48773100*	*1.11117400*
H	*-1.92609500*	*-6.68708900*	*1.82418100*
H	*-4.15310200*	*-5.90433300*	*1.05749400*
C	*-6.35390600*	*2.88454400*	*-1.67677000*
H	*-5.82199200*	*3.82315900*	*-1.83260000*
C	*-6.76945400*	*0.67117600*	*-1.75897100*
H	*-6.60941000*	*-0.38602400*	*-1.96325800*
C	*-7.71810200*	*2.80579100*	*-2.36596600*
C	*-8.00810300*	*1.28457800*	*-2.41338400*
C	*-5.67277000*	*1.62993200*	*-2.27724800*
H	*-7.67058400*	*3.22939300*	*-3.36906700*
H	*-8.47820200*	*3.36525500*	*-1.82079700*
H	*-8.11901800*	*0.93534300*	*-3.43990500*
H	*-8.92082400*	*1.01079500*	*-1.88475000*
H	*-7.77754500*	*0.68884900*	*0.16123500*
H	*-7.41386100*	*3.07095400*	*0.19394000*
H	*-5.71711000*	*1.68087200*	*-3.37076900*
C	*-4.31087500*	*1.35298900*	*-1.88941600*
C	*-3.16020200*	*1.12193600*	*-1.60300600*
C	*-1.80562200*	*0.88467800*	*-1.27169900*

C	*-0.99651800*	*1.98464200*	*-0.97885500*
C	*-1.35359900*	*-0.43352600*	*-1.19641900*
C	*-1.50920400*	*3.30784600*	*-0.84058800*
N	*0.33433200*	*1.94275400*	*-0.66452300*
C	*-2.21924400*	*-1.56633300*	*-1.25266300*
N	*-0.05570300*	*-0.81601700*	*-1.00416900*
H	*-2.52727700*	*3.58937400*	*-1.05386100*
C	*-0.49701900*	*4.07274800*	*-0.37515700*
C	*0.65321400*	*3.22846700*	*-0.28739800*
C	*-1.44290600*	*-2.65624700*	*-1.06230200*
H	*-3.29065800*	*-1.51147000*	*-1.36689400*
C	*-0.09305400*	*-2.19177600*	*-0.94619000*
H	*-0.51750000*	*5.11776400*	*-0.11714400*
C	*1.91109900*	*3.65931700*	*0.10797200*
H	*-1.75345400*	*-3.68466000*	*-0.99054300*
C	*1.02074000*	*-3.01731600*	*-0.85233800*
C	*3.03859200*	*2.84623500*	*0.04518900*
C	*2.32617700*	*-2.52431100*	*-0.85601700*
C	*4.37095900*	*3.33653800*	*0.20247600*
N	*3.05627900*	*1.51912200*	*-0.30730400*
N	*2.69193200*	*-1.20372500*	*-0.79122000*
C	*3.48436500*	*-3.35191100*	*-0.99226100*
C	*5.20411100*	*2.30904000*	*-0.07445300*
H	*4.62677100*	*4.35458200*	*0.44392200*
C	*4.38565400*	*1.17321100*	*-0.36040600*
C	*4.06551400*	*-1.20089000*	*-0.83544700*
C	*4.55910400*	*-2.53312500*	*-0.98654400*
H	*3.46865800*	*-4.42285400*	*-1.10039900*

H	*6.28042800*	*2.31604600*	*-0.10089600*
C	*4.88261200*	*-0.09166900*	*-0.65111300*
H	*5.59929900*	*-2.80138500*	*-1.06188300*
C	*6.35143500*	*-0.28374900*	*-0.71750900*
C	*7.15358100*	*-0.09909000*	*0.40851200*
C	*6.96194700*	*-0.66584200*	*-1.91203700*
C	*8.52474300*	*-0.28718500*	*0.34154100*
C	*8.33347600*	*-0.85113300*	*-1.98033400*
C	*9.11977700*	*-0.66224700*	*-0.85371600*
C	*0.83935700*	*-4.48542000*	*-0.79145100*
C	*1.38810800*	*-5.21198600*	*0.26722900*
C	*0.14707500*	*-5.18540100*	*-1.78062000*
C	*1.23925400*	*-6.58657400*	*0.34340200*
C	*-0.00381700*	*-6.56029700*	*-1.70510500*
C	*0.53940900*	*-7.26626600*	*-0.64201700*
C	*2.08739100*	*5.05727500*	*0.56696400*
C	*1.86724300*	*6.13883600*	*-0.28549800*
C	*2.49925000*	*5.31464200*	*1.87476700*
C	*2.04760700*	*7.43903500*	*0.15863400*
C	*2.67620700*	*6.61452100*	*2.32033000*
C	*2.45046300*	*7.68140400*	*1.46344200*
H	*-5.58063400*	*3.33292700*	*1.58706600*
H	*-4.49708500*	*3.13516900*	*0.21698400*
O	*-5.51268700*	*-0.83142600*	*0.65963800*
H	*6.68847400*	*0.18771500*	*1.34403400*
H	*9.12952600*	*-0.14515100*	*1.22815000*
H	*10.19092100*	*-0.80803800*	*-0.90648700*
H	*8.78937600*	*-1.14083000*	*-2.91851700*

H	*6.34805300*	*-0.81244700*	*-2.79263300*
H	*2.67428300*	*4.47964900*	*2.54316700*
H	*2.98864500*	*6.79430400*	*3.34111200*
H	*2.59010100*	*8.69715300*	*1.81008000*
H	*1.87881600*	*8.26569600*	*-0.51971800*
H	*1.56229200*	*5.94997200*	*-1.30774600*
H	*-0.26243300*	*-4.64094000*	*-2.62235100*
H	*-0.54031500*	*-7.08341200*	*-2.48631900*
H	*0.42123900*	*-8.34062800*	*-0.58433600*
H	*1.66568700*	*-7.12724000*	*1.17919200*
H	*1.92881100*	*-4.67908900*	*1.04128500*
Co	*1.49771200*	*0.34406100*	*-0.55102600*
N	*-1.04006700*	*-1.00760000*	*2.86585300*
H	*0.01168600*	*-2.64028900*	*3.60121800*
H	*0.30169300*	*-2.30003400*	*1.90566600*
C	*-0.13260500*	*0.02622000*	*3.33416600*
H	*-0.68192800*	*0.68827700*	*4.00398000*
H	*0.64735000*	*-0.47050900*	*3.91477000*
C	*0.47429100*	*0.86469600*	*2.20265500*
H	*-0.35981200*	*1.31514900*	*1.66596400*
H	*1.02539000*	*1.69930200*	*2.66323000*
C	*1.34483000*	*0.06461600*	*1.31147500*
H	*2.26256700*	*-0.34106500*	*1.74762400*

Intermediate 10 (doublet)

Charge: 0, Multiplicity: 2, NImag: 0

E = -4231.92321672 ht

G = -4231.09817430 ht

G (with entropy correction) = -4231.08603886 ht

C	*-6.81513900*	*1.06347800*	*-0.37031700*
C	*-6.57101100*	*2.58674100*	*-0.30362200*
C	*-5.73644000*	*0.45366000*	*0.49726400*
N	*-5.07564200*	*1.43849000*	*1.12403300*
H	*-4.23778400*	*1.22535600*	*1.67033100*
C	*-5.38806600*	*2.77049200*	*0.66523700*
C	*-0.77665000*	*-2.34438800*	*2.90076700*
C	*-2.38070600*	*-0.58897600*	*2.44857200*
C	*-1.63921900*	*-3.32454000*	*2.40944400*
N	*-3.20985600*	*-1.56350100*	*1.98547200*
O	*-2.69346900*	*0.60006000*	*2.45493200*
C	*-2.90378600*	*-2.91256600*	*1.91694100*
C	*-1.33189300*	*-4.69993100*	*2.35399700*
H	*-4.08689200*	*-1.23916600*	*1.55660400*
C	*-3.79505200*	*-3.82333200*	*1.37866400*
H	*-0.37604200*	*-5.03578700*	*2.73583300*
C	*-2.22438300*	*-5.60201200*	*1.81504400*
C	*-3.45499100*	*-5.17133300*	*1.32252600*
H	*-4.74629700*	*-3.46821800*	*1.00210300*
H	*-1.96264600*	*-6.65135300*	*1.77306800*
H	*-4.15125000*	*-5.88273100*	*0.89921400*
C	*-6.32406900*	*2.95052900*	*-1.77325400*
H	*-5.80005600*	*3.89557500*	*-1.91563600*

C	*-6.70903500*	*0.73201300*	*-1.86127200*
H	*-6.52901200*	*-0.32361600*	*-2.05708000*
C	*-7.66563600*	*2.85240400*	*-2.50328000*
C	*-7.93400000*	*1.32742400*	*-2.55668500*
C	*-5.60865600*	*1.70336400*	*-2.34759300*
H	*-7.59334700*	*3.27519700*	*-3.50518400*
H	*-8.44933900*	*3.40251600*	*-1.98253500*
H	*-8.00639300*	*0.97473700*	*-3.58540800*
H	*-8.85981900*	*1.04239800*	*-2.05761900*
H	*-7.78228400*	*0.74629700*	*0.02326500*
H	*-7.43946400*	*3.13088800*	*0.06637500*
H	*-5.62046900*	*1.75082600*	*-3.44213000*
C	*-4.25611700*	*1.43904300*	*-1.91931300*
C	*-3.11158200*	*1.20822200*	*-1.60861200*
C	*-1.76516600*	*0.94972500*	*-1.25962100*
C	*-0.93628400*	*2.03261700*	*-0.95867200*
C	*-1.34199300*	*-0.37854200*	*-1.17441800*
C	*-1.42383000*	*3.36381700*	*-0.80695400*
N	*0.39073300*	*1.95883700*	*-0.63161400*
C	*-2.23096400*	*-1.49335400*	*-1.21833700*
N	*-0.05133500*	*-0.78695100*	*-0.98088500*
H	*-2.43427900*	*3.66920100*	*-1.02414000*
C	*-0.40110100*	*4.10060700*	*-0.31861900*
C	*0.73212900*	*3.23222500*	*-0.23459800*
C	*-1.47783200*	*-2.59836000*	*-1.01646700*
H	*-3.30089800*	*-1.41832500*	*-1.33557900*
C	*-0.11860800*	*-2.16206200*	*-0.90982200*
H	*-0.40432800*	*5.14157300*	*-0.04387100*

C	*1.99820300*	*3.63140300*	*0.17054400*
H	*-1.80934400*	*-3.61965400*	*-0.93646800*
C	*0.97873600*	*-3.00948700*	*-0.81692700*
C	*3.11139600*	*2.79863100*	*0.08555500*
C	*2.29409400*	*-2.54362500*	*-0.84833000*
C	*4.45443300*	*3.26721700*	*0.22616600*
N	*3.10173500*	*1.47731800*	*-0.28505200*
N	*2.68385300*	*-1.22920900*	*-0.79874100*
C	*3.43444300*	*-3.39275900*	*-0.99727900*
C	*5.26668100*	*2.23319200*	*-0.08634000*
H	*4.73076300*	*4.27735000*	*0.47824800*
C	*4.42519400*	*1.11399800*	*-0.37291000*
C	*4.05636000*	*-1.25033400*	*-0.86624200*
C	*4.52317500*	*-2.59209600*	*-1.01927000*
H	*3.39772800*	*-4.46439600*	*-1.09394000*
H	*6.34220200*	*2.22657200*	*-0.13585400*
C	*4.89524200*	*-0.15495900*	*-0.69119400*
H	*5.55707300*	*-2.87926700*	*-1.11040400*
C	*6.35800100*	*-0.37089700*	*-0.79831400*
C	*7.19709800*	*-0.20063000*	*0.30290300*
C	*6.92583500*	*-0.76416900*	*-2.01043200*
C	*8.56218600*	*-0.41238000*	*0.19449900*
C	*8.29110600*	*-0.97311200*	*-2.12019100*
C	*9.11433500*	*-0.79756900*	*-1.01799900*
C	*0.76973000*	*-4.47264900*	*-0.73513000*
C	*1.30604700*	*-5.19171100*	*0.33466200*
C	*0.06581600*	*-5.17459800*	*-1.71427100*
C	*1.13665200*	*-6.56267600*	*0.42955000*

C	*-0.10613100*	*-6.54596700*	*-1.61970000*
C	*0.42695500*	*-7.24513300*	*-0.54696700*
C	*2.20374000*	*5.01590500*	*0.65639700*
C	*1.99211300*	*6.12104200*	*-0.16786100*
C	*2.64009300*	*5.23684900*	*1.96318700*
C	*2.20299700*	*7.40728100*	*0.30282900*
C	*2.84783600*	*6.52258800*	*2.43543900*
C	*2.62913900*	*7.61278600*	*1.60657100*
H	*-5.64537200*	*3.41954100*	*1.50530300*
H	*-4.52274400*	*3.21792800*	*0.16670700*
O	*-5.52983800*	*-0.75185500*	*0.61661800*
H	*6.76562100*	*0.09295100*	*1.25230100*
H	*9.19587000*	*-0.28130000*	*1.06243100*
H	*10.18073200*	*-0.96181700*	*-1.10305400*
H	*8.71329100*	*-1.27088300*	*-3.07154300*
H	*6.28303000*	*-0.90046700*	*-2.87183000*
H	*2.81144100*	*4.38412100*	*2.60980000*
H	*3.17931500*	*6.67308300*	*3.45497700*
H	*2.79299200*	*8.61765200*	*1.97373100*
H	*2.04061000*	*8.25201000*	*-0.35453600*
H	*1.67118600*	*5.96152900*	*-1.19018500*
H	*-0.33598600*	*-4.63520700*	*-2.56302000*
H	*-0.65111800*	*-7.07174000*	*-2.39324100*
H	*0.29261500*	*-8.31675200*	*-0.47449400*
H	*1.55374300*	*-7.09825200*	*1.27332900*
H	*1.85250300*	*-4.65489000*	*1.10203500*
Co	*1.52249100*	*0.33252800*	*-0.54918900*
N	*-1.15610600*	*-1.01112700*	*2.92877900*

H	*0.20115900*	*-2.56479500*	*3.30243000*
H	*1.00251500*	*-1.15262100*	*1.31686100*
C	*-0.21780100*	*0.02609500*	*3.34460900*
H	*-0.75941000*	*0.73416000*	*3.97081500*
H	*0.54558200*	*-0.45777900*	*3.95482500*
C	*0.38685700*	*0.75312900*	*2.14342100*
H	*-0.45732800*	*1.08328300*	*1.53937700*
H	*0.88088300*	*1.66500400*	*2.48804400*
C	*1.33824000*	*-0.11409000*	*1.36135400*
H	*2.35424500*	*-0.09105700*	*1.75952300*

Tranition State TS1 (doublet)

Charge: 0, Multiplicity: 2, NImag: 1

E = -4231.87796777 ht

G = -4231.05564744 ht

G (with entropy correction) = -4231.04397093 ht

C	*-6.94390100*	*0.42757700*	*0.28357800*
C	*-6.79729700*	*1.93666800*	*-0.00695700*
C	*-5.75840600*	*0.07665000*	*1.15427000*
N	*-5.11446400*	*1.20644600*	*1.48351900*
H	*-4.25607200*	*1.17634400*	*2.03867000*
C	*-5.55159200*	*2.38690300*	*0.77883200*
C	*-0.07032600*	*-1.52371100*	*2.71527700*
C	*-2.14537400*	*-0.22511000*	*2.84961000*
C	*-0.84970800*	*-2.69664100*	*2.31455400*
N	*-2.79914600*	*-1.30859300*	*2.34703200*
O	*-2.69508100*	*0.86488300*	*2.98638700*
C	*-2.22848600*	*-2.55109300*	*2.13658500*
C	*-0.26979400*	*-3.93990100*	*2.08375500*
H	*-3.79514600*	*-1.18473000*	*2.12236900*
C	*-3.01015000*	*-3.63850000*	*1.75545500*
H	*0.80038000*	*-4.04963900*	*2.21800700*
C	*-1.04000100*	*-5.02271100*	*1.69563900*
C	*-2.41287600*	*-4.86720000*	*1.53862000*
H	*-4.07662700*	*-3.50095700*	*1.62943700*
H	*-0.57371400*	*-5.98237700*	*1.51712200*
H	*-3.02236700*	*-5.71100700*	*1.24191800*
C	*-6.70418800*	*1.97456800*	*-1.53753900*
H	*-6.25775100*	*2.88648500*	*-1.93405900*

C	*-6.94806200*	*-0.22268100*	*-1.10173400*
H	*-6.71777000*	*-1.28639100*	*-1.08034100*
C	*-8.09600500*	*1.65672400*	*-2.08958900*
C	*-8.26668000*	*0.14760500*	*-1.78255900*
C	*-5.96296400*	*0.66398200*	*-1.89770400*
H	*-8.14077000*	*1.84843900*	*-3.16151100*
H	*-8.86509700*	*2.27319500*	*-1.62435100*
H	*-8.40681900*	*-0.42817800*	*-2.69732100*
H	*-9.12376300*	*-0.06026400*	*-1.14249400*
H	*-7.84954100*	*0.16300400*	*0.83206400*
H	*-7.66520500*	*2.50869700*	*0.31972300*
H	*-6.08109300*	*0.46538000*	*-2.96884400*
C	*-4.55827700*	*0.56967400*	*-1.57845000*
C	*-3.36722000*	*0.48385900*	*-1.39285500*
C	*-1.96610900*	*0.41991300*	*-1.19799800*
C	*-1.26259700*	*1.62240100*	*-1.09954300*
C	*-1.35446200*	*-0.83391000*	*-1.15189400*
C	*-1.89573300*	*2.89959100*	*-1.14671300*
N	*0.08361700*	*1.75097800*	*-0.89575400*
C	*-2.07784600*	*-2.06063000*	*-1.23166000*
N	*-0.00889700*	*-1.06067500*	*-1.06313100*
H	*-2.94854300*	*3.04969900*	*-1.32071200*
C	*-0.93069100*	*3.82114600*	*-0.93625500*
C	*0.29793000*	*3.10512300*	*-0.77873100*
C	*-1.16169700*	*-3.05189100*	*-1.20224000*
H	*-3.15280100*	*-2.13241400*	*-1.28031000*
C	*0.12551400*	*-2.42807100*	*-1.11257300*
H	*-1.03235800*	*4.89173700*	*-0.88214000*

C	*1.51140700*	*3.71055400*	*-0.48179500*
H	*-1.33596700*	*-4.11383400*	*-1.20849300*
C	*1.32825500*	*-3.12656000*	*-1.06866300*
C	*2.69488300*	*3.00064700*	*-0.30625800*
C	*2.56387800*	*-2.50313700*	*-0.89685800*
C	*3.96184000*	*3.62084600*	*-0.07493200*
N	*2.83790200*	*1.63736600*	*-0.39091100*
N	*2.77281700*	*-1.15997200*	*-0.69893800*
C	*3.81180900*	*-3.20450200*	*-0.89480300*
C	*4.87871200*	*2.63101000*	*-0.00799600*
H	*4.12454200*	*4.68330400*	*-0.00868100*
C	*4.17764700*	*1.39994200*	*-0.19844600*
C	*4.12823500*	*-1.02570400*	*-0.52284600*
C	*4.77585300*	*-2.29492800*	*-0.63912200*
H	*3.93115300*	*-4.25881200*	*-1.07428000*
H	*5.94372900*	*2.71936400*	*0.12485200*
C	*4.80247900*	*0.16006900*	*-0.25363400*
H	*5.83562900*	*-2.46050400*	*-0.54606500*
C	*6.26920300*	*0.10871000*	*-0.03368600*
C	*6.81135000*	*0.42534900*	*1.21163700*
C	*7.13848200*	*-0.24532100*	*-1.06518200*
C	*8.18061400*	*0.38456000*	*1.42134800*
C	*8.50804000*	*-0.28364800*	*-0.85720500*
C	*9.03387000*	*0.02986300*	*0.38728300*
C	*1.29322400*	*-4.59998300*	*-1.22277400*
C	*1.83817200*	*-5.44520500*	*-0.25353700*
C	*0.70972500*	*-5.18549600*	*-2.34808700*
C	*1.79331200*	*-6.82188400*	*-0.39770000*

C	*0.66269900*	*-6.56231800*	*-2.49377800*
C	*1.20152300*	*-7.38695000*	*-1.51758700*
C	*1.55073700*	*5.18609100*	*-0.33943600*
C	*1.28539400*	*6.02093400*	*-1.42454200*
C	*1.85520500*	*5.77129500*	*0.88959600*
C	*1.32345300*	*7.39918300*	*-1.28497400*
C	*1.89056900*	*7.14917100*	*1.03082600*
C	*1.62519600*	*7.96809000*	*-0.05666400*
H	*-5.77885800*	*3.19435700*	*1.47835900*
H	*-4.76351400*	*2.75068000*	*0.11234100*
O	*-5.46287100*	*-1.06067200*	*1.51354800*
H	*6.14464800*	*0.70294100*	*2.01946100*
H	*8.58161700*	*0.62816200*	*2.39701500*
H	*10.10346200*	*-0.00074600*	*0.54993400*
H	*9.16681000*	*-0.55466700*	*-1.67244400*
H	*6.72879800*	*-0.48548300*	*-2.03897400*
H	*2.05935700*	*5.13051500*	*1.73962200*
H	*2.12198200*	*7.58426400*	*1.99475200*
H	*1.65380200*	*9.04451200*	*0.05251000*
H	*1.12114800*	*8.03072900*	*-2.14071000*
H	*1.05361200*	*5.57680400*	*-2.38517700*
H	*0.29539100*	*-4.54449100*	*-3.11656500*
H	*0.20888000*	*-6.99198800*	*-3.37772200*
H	*1.16465700*	*-8.46269300*	*-1.63080200*
H	*2.21656900*	*-7.45533900*	*0.37172000*
H	*2.29535000*	*-5.00902900*	*0.62612400*
Co	*1.40455400*	*0.28572800*	*-0.61438500*
N	*-0.83412500*	*-0.43294800*	*3.23790700*

H	*0.75509800*	*-1.76298900*	*3.38868900*
H	*0.52373200*	*-1.01152200*	*1.75265400*
C	*-0.02073600*	*0.74513400*	*3.45512600*
H	*-0.62216700*	*1.50228700*	*3.94949000*
H	*0.80696700*	*0.46388500*	*4.10968600*
C	*0.49704900*	*1.26650400*	*2.10022500*
H	*-0.37105300*	*1.63757600*	*1.55274200*
H	*1.14263400*	*2.13233800*	*2.29307400*
C	*1.23116300*	*0.21673000*	*1.33282400*
H	*2.19631600*	*-0.00950000*	*1.81326300*

Final Product Complex (doublet)

Charge: 0, Multiplicity: 2, NImag: 0

E = -4231.98317165 ht

G = -4231.15346430 ht

G (with entropy correction) = -4231.14195753 ht

C	*-6.57005000*	*-0.19011700*	*1.20080800*
C	*-6.91986000*	*1.27079000*	*0.84138900*
C	*-5.10531000*	*-0.15361400*	*1.59925400*
N	*-4.71914900*	*1.13037200*	*1.69330100*
H	*-3.72890900*	*1.35857200*	*1.82620000*
C	*-5.64885600*	*2.08395300*	*1.14110700*
C	*0.98455900*	*-0.10744900*	*3.08646400*
C	*-1.18375800*	*0.67685100*	*2.19704100*
C	*0.59216500*	*-1.49742300*	*2.70902400*
N	*-1.61470300*	*-0.62078700*	*2.31265000*
O	*-1.94055700*	*1.61220700*	*1.93969600*
C	*-0.75058500*	*-1.70785700*	*2.37508000*
C	*1.45790500*	*-2.57709100*	*2.76346000*
H	*-2.60699600*	*-0.79245400*	*2.11861900*
C	*-1.21180800*	*-2.99746000*	*2.12239000*
H	*2.49821300*	*-2.41036000*	*3.01855600*
C	*1.00038100*	*-3.86282900*	*2.51671700*
C	*-0.33601600*	*-4.06615200*	*2.20019800*
H	*-2.25204200*	*-3.14477900*	*1.86081200*
H	*1.68265000*	*-4.70020800*	*2.58355200*
H	*-0.69946900*	*-5.06672000*	*2.00023500*
C	*-7.33659400*	*1.16609900*	*-0.63163000*
H	*-7.27726500*	*2.10732200*	*-1.17759400*

C	*-6.89082600*	*-0.97168500*	*-0.07596300*
H	*-6.41897900*	*-1.95233400*	*-0.11231100*
C	*-8.71176800*	*0.49622400*	*-0.67231200*
C	*-8.40868000*	*-0.96661900*	*-0.26270400*
C	*-6.45960500*	*0.01830800*	*-1.18636800*
H	*-9.13683800*	*0.54413200*	*-1.67493000*
H	*-9.41921600*	*0.98630600*	*-0.00362400*
H	*-8.70691300*	*-1.66594600*	*-1.04368200*
H	*-8.92797700*	*-1.26738800*	*0.64707400*
H	*-7.13546900*	*-0.58952200*	*2.04464500*
H	*-7.75318900*	*1.65505700*	*1.42901600*
H	*-6.85409200*	*-0.30769600*	*-2.15464700*
C	*-5.03334900*	*0.20815100*	*-1.28914100*
C	*-3.82634200*	*0.25072900*	*-1.29280300*
C	*-2.41454000*	*0.22920400*	*-1.21638300*
C	*-1.71521800*	*1.43482500*	*-1.22492900*
C	*-1.78761200*	*-1.01688500*	*-1.15994000*
C	*-2.34975500*	*2.70377300*	*-1.35638500*
N	*-0.35898000*	*1.57264100*	*-1.11351800*
C	*-2.49376700*	*-2.25324500*	*-1.24243800*
N	*-0.43588800*	*-1.22759400*	*-1.10305100*
H	*-3.41370900*	*2.84088200*	*-1.45837600*
C	*-1.37164900*	*3.63377100*	*-1.32540100*
C	*-0.13417600*	*2.93043800*	*-1.17734100*
C	*-1.56189800*	*-3.22927700*	*-1.28874600*
H	*-3.56752300*	*-2.33882400*	*-1.25963600*
C	*-0.28351000*	*-2.59155100*	*-1.20441900*
H	*-1.46752400*	*4.70485600*	*-1.37933300*

C	*1.10081100*	*3.55255900*	*-1.05734700*
H	*-1.71395100*	*-4.29440400*	*-1.33870900*
C	*0.92013800*	*-3.28379800*	*-1.17730700*
C	*2.28192000*	*2.85664000*	*-0.81467400*
C	*2.13693300*	*-2.66465400*	*-0.90424400*
C	*3.55800500*	*3.48346500*	*-0.67743600*
N	*2.39987900*	*1.49330600*	*-0.66953300*
N	*2.32901000*	*-1.31474000*	*-0.72115400*
C	*3.37059700*	*-3.36726400*	*-0.74773800*
C	*4.45403000*	*2.50415900*	*-0.41991900*
H	*3.74101300*	*4.53901700*	*-0.78561100*
C	*3.73424900*	*1.27033800*	*-0.42073000*
C	*3.66971300*	*-1.17493400*	*-0.44260800*
C	*4.30872500*	*-2.45220500*	*-0.41909000*
H	*3.49770800*	*-4.42906700*	*-0.86786500*
H	*5.51759000*	*2.59643100*	*-0.27759900*
C	*4.34096200*	*0.02895300*	*-0.26241200*
H	*5.34964000*	*-2.61972700*	*-0.20164200*
C	*5.78021700*	*0.00357800*	*0.09288900*
C	*6.20354000*	*0.51373000*	*1.32068800*
C	*6.74029200*	*-0.50786600*	*-0.77961600*
C	*7.54433600*	*0.50443800*	*1.66991000*
C	*8.08193600*	*-0.51436400*	*-0.43258700*
C	*8.48834300*	*-0.01064100*	*0.79407000*
C	*0.88841100*	*-4.73750500*	*-1.47029900*
C	*1.31694600*	*-5.69519900*	*-0.55083300*
C	*0.40661200*	*-5.17942400*	*-2.70447000*
C	*1.26589900*	*-7.04635900*	*-0.85442500*

C	*0.35891500*	*-6.52949500*	*-3.01039900*
C	*0.78802500*	*-7.46944200*	*-2.08547200*
C	*1.15502800*	*5.03212700*	*-1.12833400*
C	*0.81401600*	*5.70964200*	*-2.29840400*
C	*1.53773500*	*5.78050300*	*-0.01462500*
C	*0.85873800*	*7.09363000*	*-2.35495200*
C	*1.57937600*	*7.16415700*	*-0.06925600*
C	*1.24102000*	*7.82543800*	*-1.24068000*
H	*-5.83888200*	*2.89677300*	*1.84569500*
H	*-5.24099900*	*2.53763800*	*0.23154900*
O	*-4.40719500*	*-1.13737600*	*1.82615300*
H	*5.46411300*	*0.91818200*	*2.00269700*
H	*7.85211000*	*0.89892400*	*2.62995900*
H	*9.53619200*	*-0.01691600*	*1.06491500*
H	*8.81325800*	*-0.90925900*	*-1.12629500*
H	*6.42488500*	*-0.89418500*	*-1.74130100*
H	*1.79650500*	*5.26236100*	*0.90218100*
H	*1.87159700*	*7.72766700*	*0.80769900*
H	*1.27438200*	*8.90636700*	*-1.28452600*
H	*0.59720100*	*7.60171300*	*-3.27443600*
H	*0.51655400*	*5.13779200*	*-3.16916800*
H	*0.07107500*	*-4.44639200*	*-3.42813700*
H	*-0.01269700*	*-6.84737800*	*-3.97624400*
H	*0.74936200*	*-8.52490200*	*-2.32212600*
H	*1.59599000*	*-7.77203600*	*-0.12176900*
H	*1.67814100*	*-5.36831600*	*0.41515000*
Co	*0.97774300*	*0.13099000*	*-0.86674600*
N	*0.15442000*	*0.84976100*	*2.36306100*

H	*0.82384500*	*0.02560800*	*4.17304700*
H	*2.63284700*	*0.01874800*	*1.74249400*
C	*0.76096600*	*2.17188900*	*2.35761500*
H	*0.64458800*	*2.62154600*	*1.37273600*
H	*0.25746400*	*2.83346400*	*3.06854500*
C	*2.22371000*	*1.90619200*	*2.73943600*
H	*2.91748000*	*2.38839400*	*2.05080200*
H	*2.43127300*	*2.30602000*	*3.73245800*
C	*2.37663100*	*0.38428700*	*2.73799400*
H	*3.14029900*	*0.03036900*	*3.42933100*

Transition State TS2 (doublet)

Charge: 0, Multiplicity: 2, NImag: 1

E = -4231.90621171 ht

G = -4231.08241330 ht

G (with entropy correction) = -4231.07011332 ht

C	*-6.84324200*	*0.25410700*	*0.24094300*
C	*-6.86154100*	*1.77975400*	*0.00120000*
C	*-5.59260600*	*-0.00193100*	*1.05393900*
N	*-5.05248700*	*1.17742700*	*1.39360000*
H	*-4.19735200*	*1.21816900*	*1.94957700*
C	*-5.62533500*	*2.32720300*	*0.73766700*
C	*-0.01927200*	*-1.28577900*	*3.29123200*
C	*-2.09188600*	*-0.07292200*	*3.02897300*
C	*-0.62497400*	*-2.49068800*	*2.85348300*
N	*-2.63508000*	*-1.22670500*	*2.55922900*
O	*-2.68591200*	*0.99784900*	*3.01082300*
C	*-1.97636200*	*-2.43971700*	*2.46482600*
C	*0.05511800*	*-3.71080600*	*2.75486700*
H	*-3.59430600*	*-1.15911200*	*2.18467600*
C	*-2.62368700*	*-3.57434000*	*1.99329900*
H	*1.09781200*	*-3.75291400*	*3.04784400*
C	*-0.59078200*	*-4.83828600*	*2.29011800*
C	*-1.92813900*	*-4.76866800*	*1.90763100*
H	*-3.66079200*	*-3.50324500*	*1.69101700*
H	*-0.05792100*	*-5.77667100*	*2.21934200*
H	*-2.43031800*	*-5.65337500*	*1.53843200*
C	*-6.85873800*	*1.87926100*	*-1.52901300*
H	*-6.52656900*	*2.84404400*	*-1.91167800*

C	*-6.86583900*	*-0.34493700*	*-1.16761800*
H	*-6.53302800*	*-1.38096600*	*-1.20383500*
C	*-8.24276100*	*1.44540600*	*-2.01836900*
C	*-8.25177500*	*-0.08209900*	*-1.75883000*
C	*-6.01708900*	*0.66146800*	*-1.97894300*
H	*-8.36530100*	*1.66552700*	*-3.07870000*
H	*-9.03992300*	*1.96939800*	*-1.49117900*
H	*-8.39052900*	*-0.63938800*	*-2.68510500*
H	*-9.04629500*	*-0.39176800*	*-1.08027800*
H	*-7.68987400*	*-0.11492900*	*0.82274100*
H	*-7.76190800*	*2.24948600*	*0.39617500*
H	*-6.17291000*	*0.49103100*	*-3.05004400*
C	*-4.59769500*	*0.66505400*	*-1.71977500*
C	*-3.40226900*	*0.61075800*	*-1.55318300*
C	*-2.00346600*	*0.52447400*	*-1.35467800*
C	*-1.27500800*	*1.70632600*	*-1.20949400*
C	*-1.42502500*	*-0.74614800*	*-1.31537400*
C	*-1.88065200*	*2.99789900*	*-1.21148000*
N	*0.07121200*	*1.79565100*	*-0.98208400*
C	*-2.18241000*	*-1.95350800*	*-1.37844400*
N	*-0.08605900*	*-1.00863200*	*-1.21685500*
H	*-2.92920200*	*3.17690600*	*-1.38437600*
C	*-0.89785200*	*3.88887900*	*-0.95579700*
C	*0.31430800*	*3.14022300*	*-0.81665900*
C	*-1.29396900*	*-2.96986700*	*-1.32546800*
H	*-3.25836000*	*-1.99602300*	*-1.43319400*
C	*0.00922300*	*-2.38152800*	*-1.24187200*
H	*-0.97735800*	*4.95825000*	*-0.85766400*

C	*1.54068300*	*3.70694700*	*-0.49711200*
H	*-1.49570900*	*-4.02745900*	*-1.31959600*
C	*1.19070300*	*-3.11213700*	*-1.17841600*
C	*2.70771200*	*2.96483900*	*-0.34037900*
C	*2.44259600*	*-2.52326300*	*-1.00892300*
C	*3.98814900*	*3.55177300*	*-0.09120700*
N	*2.81881100*	*1.60131000*	*-0.45763100*
N	*2.68815100*	*-1.18447000*	*-0.82282100*
C	*3.66836300*	*-3.26160200*	*-0.99003200*
C	*4.88292200*	*2.54101000*	*-0.05270600*
H	*4.17430200*	*4.60800100*	*0.00611600*
C	*4.15331900*	*1.33091300*	*-0.27588400*
C	*4.04700000*	*-1.08762600*	*-0.64387700*
C	*4.65820500*	*-2.37644100*	*-0.74620700*
H	*3.75490700*	*-4.32270500*	*-1.14862000*
H	*5.94946600*	*2.60302100*	*0.08259100*
C	*4.75027500*	*0.07819800*	*-0.36151900*
H	*5.71206700*	*-2.57196800*	*-0.64409300*
C	*6.21631700*	*-0.01409700*	*-0.15205900*
C	*6.77848900*	*0.27948400*	*1.09016900*
C	*7.06663700*	*-0.38982900*	*-1.19190200*
C	*8.14753600*	*0.19646300*	*1.28834100*
C	*8.43601000*	*-0.47072700*	*-0.99559700*
C	*8.98139600*	*-0.17900700*	*0.24577000*
C	*1.11216000*	*-4.58642200*	*-1.30515200*
C	*1.58945200*	*-5.42443300*	*-0.29618700*
C	*0.55227400*	*-5.17629000*	*-2.43986200*
C	*1.50267000*	*-6.80210700*	*-0.41287300*

C	*0.46723000*	*-6.55386500*	*-2.55938600*
C	*0.93967900*	*-7.37283500*	*-1.54459100*
C	*1.61302400*	*5.17598400*	*-0.30832200*
C	*1.36544700*	*6.05119500*	*-1.36555400*
C	*1.93132800*	*5.71528900*	*0.93821800*
C	*1.43418500*	*7.42310700*	*-1.18212800*
C	*1.99734200*	*7.08682700*	*1.12354700*
C	*1.74930000*	*7.94585100*	*0.06324900*
H	*-5.88949400*	*3.09700400*	*1.46582600*
H	*-4.90647700*	*2.77273700*	*0.04279800*
O	*-5.17649500*	*-1.11215800*	*1.37973200*
H	*6.12722600*	*0.57256500*	*1.90504000*
H	*8.56360400*	*0.42281900*	*2.26189600*
H	*10.05085100*	*-0.24291800*	*0.39950600*
H	*9.07951400*	*-0.75815300*	*-1.81741300*
H	*6.64176400*	*-0.61379100*	*-2.16303100*
H	*2.12282900*	*5.04344200*	*1.76696900*
H	*2.23943900*	*7.48565500*	*2.10049100*
H	*1.80209000*	*9.01735300*	*0.20662300*
H	*1.24551000*	*8.08595900*	*-2.01707900*
H	*1.12323900*	*5.64331800*	*-2.33958100*
H	*0.18659000*	*-4.53844800*	*-3.23537900*
H	*0.03448900*	*-6.98888600*	*-3.45128800*
H	*0.87212600*	*-8.44918100*	*-1.63637100*
H	*1.87154400*	*-7.43211900*	*0.38711300*
H	*2.02163400*	*-4.98072700*	*0.59243400*
Co	*1.36674500*	*0.29797200*	*-0.78409900*
N	*-0.80087100*	*-0.17243300*	*3.55139800*

H	*0.92654200*	*-1.30906400*	*3.81274200*
H	*0.34533400*	*-0.71958500*	*1.05132000*
C	*-0.02541000*	*1.04948600*	*3.55368000*
H	*-0.64621800*	*1.87966600*	*3.87673900*
H	*0.79845700*	*0.92109200*	*4.25588100*
C	*0.50220300*	*1.24292000*	*2.10631500*
H	*-0.33290000*	*1.63353700*	*1.52340000*
H	*1.26873000*	*2.02122500*	*2.11648400*
C	*1.01515100*	*-0.01256500*	*1.51016500*
H	*2.01237500*	*-0.36239000*	*1.74056700*

Complex 9 (quartet)

Charge: 0, Multiplicity: 4, NImag: 0

E = -4231.86333464 ht

G = -4231.04254371 ht

G (with entropy correction) = -4231.03078940 ht

C	*-6.96652500*	*0.93880700*	*-0.16386900*
C	*-6.68405900*	*2.45642600*	*-0.19417500*
C	*-5.92997300*	*0.36364800*	*0.77436300*
N	*-5.26133800*	*1.37275900*	*1.35266700*
H	*-4.46000200*	*1.18384100*	*1.96009100*
C	*-5.52454200*	*2.67714700*	*0.79494100*
C	*-0.58115800*	*-2.04614100*	*2.62481900*
C	*-2.51574200*	*-0.50542600*	*2.67671300*
C	*-1.50627600*	*-3.11475900*	*2.15657800*
N	*-3.23704800*	*-1.46472500*	*2.03470700*
O	*-2.95791500*	*0.62682700*	*2.86851400*
C	*-2.82025800*	*-2.77144700*	*1.84670900*
C	*-1.07916600*	*-4.41587500*	*1.94400700*
H	*-4.18274600*	*-1.20493600*	*1.72820400*
C	*-3.69234900*	*-3.72614700*	*1.32771500*
H	*-0.05685600*	*-4.67998700*	*2.18758600*
C	*-1.93396600*	*-5.36903200*	*1.41301200*
C	*-3.24232200*	*-5.01541400*	*1.10725300*
H	*-4.70809300*	*-3.43658400*	*1.08953300*
H	*-1.57998700*	*-6.37606900*	*1.23754700*
H	*-3.91991800*	*-5.75206400*	*0.69497200*
C	*-6.38481500*	*2.71073600*	*-1.67676300*
H	*-5.83243500*	*3.63078700*	*-1.86787300*

C	*-6.82309800*	*0.50139900*	*-1.62345700*
H	*-6.66500500*	*-0.56936800*	*-1.73945100*
C	*-7.70674500*	*2.59363700*	*-2.43920400*
C	*-8.01088100*	*1.07496900*	*-2.39746900*
C	*-5.68511000*	*1.41038100*	*-2.14131000*
H	*-7.59522000*	*2.94547100*	*-3.46465500*
H	*-8.49144700*	*3.19538800*	*-1.98072100*
H	*-8.06002300*	*0.65419600*	*-3.40170200*
H	*-8.95857900*	*0.84596600*	*-1.91074000*
H	*-7.95281300*	*0.67318000*	*0.22028200*
H	*-7.54757500*	*3.04655100*	*0.11133300*
H	*-5.66980500*	*1.38177900*	*-3.23676900*
C	*-4.34246000*	*1.15613100*	*-1.67418100*
C	*-3.18768800*	*0.94918900*	*-1.38149300*
C	*-1.81738500*	*0.74118900*	*-1.09030700*
C	*-1.03359900*	*1.88146500*	*-0.83233500*
C	*-1.33470700*	*-0.57794800*	*-1.11025400*
C	*-1.57071100*	*3.19872100*	*-0.71230600*
N	*0.31625500*	*1.89829000*	*-0.63437000*
C	*-2.16402100*	*-1.73013000*	*-1.29359100*
N	*-0.03475800*	*-0.94578300*	*-0.94180100*
H	*-2.61168000*	*3.45156800*	*-0.83314800*
C	*-0.53397200*	*4.02121600*	*-0.41868000*
C	*0.64727800*	*3.21115700*	*-0.39379500*
C	*-1.35087200*	*-2.80926500*	*-1.22362000*
H	*-3.23592500*	*-1.70546000*	*-1.41597700*
C	*-0.01436100*	*-2.31346300*	*-1.02982200*
H	*-0.56758600*	*5.08160700*	*-0.23296400*

C	*1.93883200*	*3.70024800*	*-0.17279900*
H	*-1.63767300*	*-3.84676500*	*-1.26451200*
C	*1.13727800*	*-3.10460400*	*-0.94481400*
C	*3.09837200*	*2.91303300*	*-0.17328900*
C	*2.44051200*	*-2.59879500*	*-0.79129900*
C	*4.43359000*	*3.43781200*	*-0.15060600*
N	*3.13523400*	*1.55161500*	*-0.26544400*
N	*2.78662500*	*-1.28172600*	*-0.62906400*
C	*3.62249700*	*-3.40598000*	*-0.83358200*
C	*5.27329200*	*2.37897000*	*-0.22761100*
H	*4.69245800*	*4.48335500*	*-0.12501400*
C	*4.45483600*	*1.20143100*	*-0.28611400*
C	*4.15675600*	*-1.24795500*	*-0.55993000*
C	*4.68010200*	*-2.57424000*	*-0.67667800*
H	*3.64796300*	*-4.47168800*	*-0.98432600*
H	*6.34946800*	*2.39435300*	*-0.27474200*
C	*4.94788800*	*-0.10356300*	*-0.39697000*
H	*5.72330500*	*-2.84016400*	*-0.64692200*
C	*6.42045000*	*-0.27861400*	*-0.35933900*
C	*7.15123800*	*0.08097000*	*0.77374700*
C	*7.11266900*	*-0.79334700*	*-1.45634100*
C	*8.52749900*	*-0.07462100*	*0.81196200*
C	*8.48927400*	*-0.94553400*	*-1.42066300*
C	*9.20183600*	*-0.58845500*	*-0.28555500*
C	*0.98421800*	*-4.57448500*	*-1.06023100*
C	*1.46281400*	*-5.42336900*	*-0.05896200*
C	*0.36337900*	*-5.15574600*	*-2.16732600*
C	*1.31081300*	*-6.79654100*	*-0.14953600*

C	*0.20922100*	*-6.52958400*	*-2.26012900*
C	*0.67828300*	*-7.35603400*	*-1.25014400*
C	*2.10879900*	*5.15791400*	*0.03582400*
C	*1.72733500*	*6.07943400*	*-0.94044100*
C	*2.67374300*	*5.64267800*	*1.21663700*
C	*1.90092400*	*7.43943500*	*-0.74094400*
C	*2.84325800*	*7.00263200*	*1.41918100*
C	*2.45725900*	*7.90649900*	*0.44061000*
H	*-5.78678700*	*3.38939100*	*1.58051700*
H	*-4.63397300*	*3.06661400*	*0.29192400*
O	*-5.75655100*	*-0.83564000*	*0.97812000*
H	*6.62495200*	*0.48157500*	*1.63190900*
H	*9.07437200*	*0.20362300*	*1.70394700*
H	*10.27712700*	*-0.70846200*	*-0.25719900*
H	*9.00768200*	*-1.33929500*	*-2.28568900*
H	*6.55800200*	*-1.06602900*	*-2.34606600*
H	*2.97571200*	*4.93665600*	*1.98126900*
H	*3.27595200*	*7.35743500*	*2.34598600*
H	*2.59173700*	*8.96903100*	*0.59680100*
H	*1.60632300*	*8.13665500*	*-1.51501500*
H	*1.30190200*	*5.71569100*	*-1.86789700*
H	*0.00791400*	*-4.51586600*	*-2.96542900*
H	*-0.27192200*	*-6.95622800*	*-3.13100700*
H	*0.55786300*	*-8.42918000*	*-1.32312200*
H	*1.68379800*	*-7.43135800*	*0.64448000*
H	*1.95433100*	*-4.98857600*	*0.80336700*
Co	*1.49580100*	*0.25696800*	*-0.12751500*
N	*-1.27527700*	*-0.88217400*	*3.13863400*

H	*0.08855500*	*-2.41938900*	*3.40621900*
H	*0.08772600*	*-1.73108500*	*1.79128800*
C	*-0.42690400*	*0.15473300*	*3.67303800*
H	*-1.04695600*	*0.83963000*	*4.24565600*
H	*0.29448000*	*-0.31540800*	*4.34547300*
C	*0.31204600*	*0.94872300*	*2.57554500*
H	*-0.42317400*	*1.47542600*	*1.96270600*
H	*0.89663400*	*1.72279200*	*3.09487000*
C	*1.25970200*	*0.16870300*	*1.76546100*
H	*1.96518200*	*-0.40152800*	*2.38840500*

Complex 10 (quartet)

Charge: 0, Multiplicity: 4, NImag: 0

E = -4231.88953899 ht

G = -4231.06890337 ht

G (with entropy correction) = -4231.05668561 ht

C	*-6.88815800*	*0.96571600*	*-0.12345200*
C	*-6.65311200*	*2.49218300*	*-0.14281800*
C	*-5.79672300*	*0.41081000*	*0.76206900*
N	*-5.13169000*	*1.43108900*	*1.32181200*
H	*-4.30155900*	*1.25362200*	*1.88789500*
C	*-5.45354800*	*2.73485500*	*0.79182800*
C	*-0.71404200*	*-2.22996200*	*2.97296200*
C	*-2.42392300*	*-0.54626400*	*2.68512200*
C	*-1.51815800*	*-3.21343200*	*2.39274800*
N	*-3.20908400*	*-1.53215700*	*2.17710000*
O	*-2.77817600*	*0.62356100*	*2.75883700*
C	*-2.82138900*	*-2.84233000*	*1.98116100*
C	*-1.10486100*	*-4.54238400*	*2.17385400*
H	*-4.12211600*	*-1.22922000*	*1.79825000*
C	*-3.67296900*	*-3.76452900*	*1.39225800*
H	*-0.10727000*	*-4.83560500*	*2.47547700*
C	*-1.95088800*	*-5.44922000*	*1.57616000*
C	*-3.23687600*	*-5.06585000*	*1.19176400*
H	*-4.66179300*	*-3.44791400*	*1.08573000*
H	*-1.61278900*	*-6.46169600*	*1.39993200*
H	*-3.89828200*	*-5.78455900*	*0.72659000*
C	*-6.43568200*	*2.77794800*	*-1.63380500*
H	*-5.92244800*	*3.71795700*	*-1.83586800*

C	-6.80204400	0.55542800	-1.59607300
H	-6.61654500	-0.50778700	-1.73854900
C	-7.78952700	2.63048800	-2.33236000
C	-8.04419600	1.10246500	-2.30085900
C	-5.71908700	1.50785000	-2.15231000
H	-7.73949800	3.00077200	-3.35608400
H	-8.56902700	3.20024100	-1.82656000
H	-8.12925900	0.69523600	-3.30819000
H	-8.95939600	0.83613500	-1.77264000
H	-7.84771300	0.66520700	0.30084100
H	-7.51824200	3.04953600	0.21507000
H	-5.75358400	1.49633200	-3.24756000
C	-4.35369000	1.28113100	-1.74193400
C	-3.19089700	1.08442300	-1.47574600
C	-1.81870300	0.86785100	-1.20232300
C	-1.00918500	2.00264500	-1.00119700
C	-1.36960800	-0.46209700	-1.13805400
C	-1.51826600	3.33123000	-0.88031900
N	0.33486700	1.98711400	-0.77588300
C	-2.23190700	-1.60507500	-1.18158400
N	-0.06774000	-0.85148800	-1.02840400
H	-2.54941600	3.61198700	-1.02382400
C	-0.47292600	4.12082700	-0.52844600
C	0.68472600	3.27797700	-0.46891900
C	-1.43705700	-2.69704000	-1.09305700
H	-3.30882100	-1.56441700	-1.24265600
C	-0.07920300	-2.22570200	-1.03737900
H	-0.49174500	5.17471300	-0.30644700

C	*1.96698300*	*3.70145700*	*-0.10071400*
H	*-1.74443700*	*-3.72886800*	*-1.05749400*
C	*1.05630300*	*-3.04222600*	*-1.03860600*
C	*3.09492600*	*2.87121900*	*-0.03059300*
C	*2.38249300*	*-2.57311400*	*-1.01109200*
C	*4.43833500*	*3.35643700*	*0.11369300*
N	*3.10840400*	*1.51563300*	*-0.21624100*
N	*2.76225400*	*-1.26763600*	*-0.87118300*
C	*3.54452800*	*-3.40799500*	*-1.08470300*
C	*5.25964400*	*2.28951900*	*-0.01020500*
H	*4.71698600*	*4.39017700*	*0.23353300*
C	*4.42704700*	*1.13780800*	*-0.21608900*
C	*4.12624000*	*-1.26626000*	*-0.77955100*
C	*4.62368900*	*-2.60022600*	*-0.93469800*
H	*3.54292900*	*-4.47497000*	*-1.23211000*
H	*6.33673800*	*2.28709800*	*-0.00890800*
C	*4.91632300*	*-0.14839900*	*-0.47363900*
H	*5.66049900*	*-2.89141900*	*-0.90737100*
C	*6.38433600*	*-0.34747800*	*-0.41489700*
C	*7.09438100*	*-0.11040100*	*0.76300500*
C	*7.09250300*	*-0.77813400*	*-1.53781700*
C	*8.46582900*	*-0.29884100*	*0.81733400*
C	*8.46450300*	*-0.96282300*	*-1.48569200*
C	*9.15652400*	*-0.72488400*	*-0.30743300*
C	*0.85707900*	*-4.51144900*	*-1.06442700*
C	*1.38611600*	*-5.31445400*	*-0.05113400*
C	*0.14204500*	*-5.13499800*	*-2.08805400*
C	*1.20018100*	*-6.68672600*	*-0.05393500*

C	*-0.04680600*	*-6.50785900*	*-2.09225100*
C	*0.47954400*	*-7.28958600*	*-1.07464200*
C	*2.16199400*	*5.13722400*	*0.20930500*
C	*1.89282500*	*6.12772400*	*-0.73641600*
C	*2.63223600*	*5.53190600*	*1.46308100*
C	*2.08346500*	*7.46703900*	*-0.43717700*
C	*2.81912300*	*6.87087800*	*1.76474200*
C	*2.54512300*	*7.84412300*	*0.81522300*
H	*-5.69672300*	*3.43041100*	*1.59795600*
H	*-4.59756900*	*3.15117600*	*0.25251200*
O	*-5.58589700*	*-0.78648700*	*0.95130100*
H	*6.55492400*	*0.21927200*	*1.64288600*
H	*8.99626200*	*-0.11604300*	*1.74330400*
H	*10.22828100*	*-0.87027200*	*-0.26614500*
H	*8.99565500*	*-1.28888800*	*-2.37095200*
H	*6.55351700*	*-0.95937100*	*-2.45993400*
H	*2.84502000*	*4.77148800*	*2.20531600*
H	*3.17683800*	*7.15514600*	*2.74626600*
H	*2.69286500*	*8.89053400*	*1.04913300*
H	*1.87690300*	*8.21885200*	*-1.18836300*
H	*1.54142600*	*5.83426900*	*-1.71820000*
H	*-0.25977900*	*-4.52991800*	*-2.89142100*
H	*-0.59988400*	*-6.96958900*	*-2.90031300*
H	*0.33391100*	*-8.36217700*	*-1.08002300*
H	*1.61551300*	*-7.28663400*	*0.74643100*
H	*1.94283000*	*-4.84303600*	*0.75056800*
Co	*1.50244800*	*0.31510600*	*-0.44847700*
N	*-1.16915000*	*-0.94634600*	*3.13971500*

H	*0.27444900*	*-2.43421400*	*3.35667200*
H	*0.86482500*	*-1.23220600*	*1.35374400*
C	*-0.23734700*	*0.11070800*	*3.48978500*
H	*-0.78820100*	*0.87326800*	*4.03532800*
H	*0.51964400*	*-0.31843700*	*4.14579200*
C	*0.39656500*	*0.71647300*	*2.22195400*
H	*-0.43565600*	*1.04952700*	*1.59630300*
H	*0.93885600*	*1.62144500*	*2.50540900*
C	*1.29512200*	*-0.24010000*	*1.50219100*
H	*2.29464500*	*-0.31407500*	*1.93300100*

Transition State TS1 (quartet)

Charge: 0, Multiplicity: 4, NImag: 1

E = -4231.86085210 ht

G = -4231.04030471 ht

G (with entropy correction) = -4231.02885611 ht

C	*-7.01312700*	*0.56018100*	*0.02773500*
C	*-6.82706500*	*2.06973100*	*-0.23771600*
C	*-5.90596900*	*0.18985400*	*0.98672500*
N	*-5.27771500*	*1.30881700*	*1.37708900*
H	*-4.46091400*	*1.26237200*	*1.98884100*
C	*-5.64501600*	*2.50016400*	*0.64989300*
C	*-0.34038500*	*-1.54697800*	*2.78935800*
C	*-2.39718600*	*-0.20399300*	*2.84068700*
C	*-1.12284800*	*-2.68315700*	*2.27757500*
N	*-3.04133000*	*-1.25178000*	*2.26177900*
O	*-2.92440300*	*0.89260500*	*2.99403900*
C	*-2.48467800*	*-2.49561500*	*2.03434800*
C	*-0.55551000*	*-3.92816900*	*2.03180100*
H	*-4.02708700*	*-1.10388800*	*1.99912200*
C	*-3.26929000*	*-3.55208100*	*1.57441500*
H	*0.50423900*	*-4.06685600*	*2.21230200*
C	*-1.32673900*	*-4.97762700*	*1.56586500*
C	*-2.68687200*	*-4.78333100*	*1.34585900*
H	*-4.32395000*	*-3.38486300*	*1.39679600*
H	*-0.87182000*	*-5.93976200*	*1.37243100*
H	*-3.29723100*	*-5.60192400*	*0.98671300*
C	*-6.60606000*	*2.11675500*	*-1.75479000*
H	*-6.11924900*	*3.02660400*	*-2.10552100*

C	*-6.90797900*	*-0.08016900*	*-1.35837200*
H	*-6.69095900*	*-1.14640700*	*-1.32604900*
C	*-7.95045300*	*1.81801400*	*-2.42285500*
C	*-8.16163200*	*0.30873100*	*-2.14323000*
C	*-5.85053700*	*0.80112600*	*-2.06177000*
H	*-7.90392000*	*2.01800800*	*-3.49313300*
H	*-8.74930400*	*2.43946100*	*-2.01824900*
H	*-8.23058400*	*-0.25911900*	*-3.07093100*
H	*-9.07135700*	*0.10580100*	*-1.57864100*
H	*-7.96324400*	*0.30115400*	*0.49812400*
H	*-7.71259800*	*2.64930900*	*0.02127700*
H	*-5.88444800*	*0.61212200*	*-3.14077900*
C	*-4.47509100*	*0.68411000*	*-1.63633400*
C	*-3.29664500*	*0.57047000*	*-1.38934900*
C	*-1.90484100*	*0.46317900*	*-1.14503700*
C	*-1.19136400*	*1.66425900*	*-0.95631000*
C	*-1.33793700*	*-0.82357500*	*-1.15357100*
C	*-1.81037600*	*2.94938300*	*-0.89305400*
N	*0.15728000*	*1.78016300*	*-0.78443700*
C	*-2.08515000*	*-2.03370500*	*-1.33074000*
N	*-0.01566700*	*-1.09950800*	*-0.99703600*
H	*-2.86652900*	*3.13005900*	*-1.01137500*
C	*-0.82490600*	*3.85124800*	*-0.66046000*
C	*0.40610000*	*3.11970200*	*-0.60487200*
C	*-1.19357000*	*-3.04997800*	*-1.28037300*
H	*-3.15584700*	*-2.08704500*	*-1.45277700*
C	*0.10546900*	*-2.45838800*	*-1.08795600*
H	*-0.92662900*	*4.91533100*	*-0.52803900*

C	*1.66175600*	*3.69748500*	*-0.37819500*
H	*-1.40144500*	*-4.10452100*	*-1.34219000*
C	*1.31081000*	*-3.16504300*	*-0.98455500*
C	*2.86727800*	*2.98670600*	*-0.28835700*
C	*2.57494100*	*-2.57527100*	*-0.79685400*
C	*4.16846600*	*3.59323200*	*-0.23657800*
N	*2.99008500*	*1.62795100*	*-0.30761400*
N	*2.83243700*	*-1.23790400*	*-0.64056800*
C	*3.80705200*	*-3.30634800*	*-0.77689700*
C	*5.07176200*	*2.58532500*	*-0.22466200*
H	*4.36524000*	*4.65243700*	*-0.25466500*
C	*4.32712800*	*1.35734500*	*-0.26431200*
C	*4.19181600*	*-1.11531900*	*-0.50643300*
C	*4.80211100*	*-2.40780800*	*-0.58044900*
H	*3.90865200*	*-4.36961800*	*-0.91230500*
H	*6.14623800*	*2.66532300*	*-0.22887300*
C	*4.90055000*	*0.08096000*	*-0.32278300*
H	*5.85692200*	*-2.60883100*	*-0.49707400*
C	*6.37770600*	*-0.00037700*	*-0.21249900*
C	*7.02890700*	*0.43036900*	*0.94421300*
C	*7.15408400*	*-0.49117800*	*-1.26322000*
C	*8.40887800*	*0.36593800*	*1.05064600*
C	*8.53430700*	*-0.55223000*	*-1.15964000*
C	*9.16709600*	*-0.12578800*	*-0.00133600*
C	*1.25558300*	*-4.64243900*	*-1.09762800*
C	*1.75619400*	*-5.46218000*	*-0.08250300*
C	*0.69960200*	*-5.26062800*	*-2.21958100*
C	*1.69055000*	*-6.84239900*	*-0.17660000*

C	*0.62996300*	*-6.64113100*	*-2.31483100*
C	*1.12107300*	*-7.43872000*	*-1.29203600*
C	*1.73292600*	*5.17165000*	*-0.24318600*
C	*1.31305000*	*6.01486600*	*-1.27327300*
C	*2.23520000*	*5.75364600*	*0.92212100*
C	*1.39022400*	*7.39185600*	*-1.14150700*
C	*2.30888100*	*7.13040300*	*1.05678800*
C	*1.88657500*	*7.95516200*	*0.02472000*
H	*-5.91885900*	*3.30420600*	*1.33644800*
H	*-4.80253100*	*2.85975100*	*0.05160800*
O	*-5.65098800*	*-0.95416000*	*1.35790600*
H	*6.43710500*	*0.81495700*	*1.76630500*
H	*8.89313900*	*0.69861900*	*1.96004500*
H	*10.24530000*	*-0.17441200*	*0.08003000*
H	*9.11810900*	*-0.92881000*	*-1.99000200*
H	*6.66174900*	*-0.81729800*	*-2.17143800*
H	*2.56425100*	*5.10946800*	*1.72918400*
H	*2.69427300*	*7.56044100*	*1.97258700*
H	*1.94586600*	*9.03095400*	*0.12792300*
H	*1.06741900*	*8.02731200*	*-1.95645700*
H	*0.93361200*	*5.57625300*	*-2.18823600*
H	*0.32712000*	*-4.64264300*	*-3.02719100*
H	*0.19794400*	*-7.09546600*	*-3.19745400*
H	*1.06744300*	*-8.51711600*	*-1.36691200*
H	*2.08092000*	*-7.45387300*	*0.62729200*
H	*2.19891100*	*-5.00129300*	*0.79234800*
Co	*1.43658300*	*0.23223800*	*-0.25676100*
N	*-1.11489000*	*-0.46545100*	*3.30279800*

H	*0.41196000*	*-1.84939200*	*3.52209700*
H	*0.32945100*	*-1.06420000*	*1.92279700*
C	*-0.29848700*	*0.66413800*	*3.67778600*
H	*-0.93215900*	*1.42476800*	*4.12360600*
H	*0.43200100*	*0.32420000*	*4.41499800*
C	*0.41511200*	*1.21864900*	*2.42624300*
H	*-0.33892600*	*1.68992200*	*1.79233600*
H	*1.09286500*	*2.01167400*	*2.76295900*
C	*1.20734000*	*0.20660500*	*1.68425500*
H	*2.04020400*	*-0.17289500*	*2.29506500*

Transition State TS2 (quartet)

Charge: 0, Multiplicity: 4, NImag: 1

E = -4231.88315619 ht

G = -4231.06232764 ht

G (with entropy correction) = -4231.05022164 ht

C	*-6.89302200*	*0.32817100*	*0.16306400*
C	*-6.87866600*	*1.85228300*	*-0.08754000*
C	*-5.67756000*	*0.05957600*	*1.02157300*
N	*-5.13319000*	*1.23211300*	*1.37618400*
H	*-4.29671000*	*1.26426800*	*1.95840700*
C	*-5.66317500*	*2.38667500*	*0.69151800*
C	*-0.15258900*	*-1.31658200*	*3.24603900*
C	*-2.21264500*	*-0.07270900*	*3.02880100*
C	*-0.77311400*	*-2.49794500*	*2.75846600*
N	*-2.77147800*	*-1.20450500*	*2.52805700*
O	*-2.78808600*	*1.00551300*	*3.05118100*
C	*-2.12707100*	*-2.41751200*	*2.38988300*
C	*-0.10234100*	*-3.71709300*	*2.60692800*
H	*-3.73544900*	*-1.11723800*	*2.16154400*
C	*-2.79446500*	*-3.53305400*	*1.89607800*
H	*0.94529800*	*-3.77541300*	*2.87847600*
C	*-0.76562800*	*-4.82262200*	*2.11830500*
C	*-2.11103600*	*-4.72748900*	*1.76582900*
H	*-3.83493500*	*-3.44345200*	*1.61162300*
H	*-0.24330200*	*-5.76216900*	*2.00463900*
H	*-2.62736400*	*-5.59663100*	*1.37933900*
C	*-6.81555500*	*1.93983600*	*-1.61717500*
H	*-6.45364300*	*2.89642600*	*-1.99326800*

C	*-6.87127200*	*-0.28088800*	*-1.24115600*
H	*-6.55393400*	*-1.32223200*	*-1.25760400*
C	*-8.18668300*	*1.52408300*	*-2.15598900*
C	*-8.22969100*	*-0.00116400*	*-1.88626800*
C	*-5.97649800*	*0.70600600*	*-2.02597600*
H	*-8.26484900*	*1.73796600*	*-3.22172900*
H	*-8.99527200*	*2.06427400*	*-1.66363400*
H	*-8.34161600*	*-0.56334300*	*-2.81316700*
H	*-9.05444300*	*-0.29333400*	*-1.23656600*
H	*-7.76589100*	*-0.02399200*	*0.71590600*
H	*-7.78605000*	*2.33830900*	*0.26981900*
H	*-6.09669200*	*0.52958800*	*-3.10083600*
C	*-4.56645500*	*0.69244500*	*-1.71891100*
C	*-3.37371000*	*0.62814000*	*-1.53275000*
C	*-1.97496900*	*0.54002600*	*-1.32901900*
C	*-1.26756200*	*1.74221600*	*-1.14708000*
C	*-1.41002900*	*-0.75354900*	*-1.32363100*
C	*-1.87514800*	*3.04112900*	*-1.11264600*
N	*0.07102200*	*1.84131800*	*-0.92825000*
C	*-2.17465900*	*-1.95894700*	*-1.36140500*
N	*-0.07740000*	*-1.04131300*	*-1.25290100*
H	*-2.92371700*	*3.23676000*	*-1.27092300*
C	*-0.88529500*	*3.92852600*	*-0.85738000*
C	*0.33616700*	*3.17283500*	*-0.75398400*
C	*-1.28925300*	*-2.98652900*	*-1.30368800*
H	*-3.25221700*	*-2.00653300*	*-1.39260800*
C	*0.02265000*	*-2.41115800*	*-1.24087200*
H	*-0.97352600*	*4.99597800*	*-0.74124900*

C	*1.59673900*	*3.71334100*	*-0.47887300*
H	*-1.51193600*	*-4.04006700*	*-1.27092600*
C	*1.21524100*	*-3.14659500*	*-1.13273600*
C	*2.78809100*	*2.97470900*	*-0.35823300*
C	*2.49006200*	*-2.59319600*	*-0.92497300*
C	*4.08636800*	*3.55480200*	*-0.19523000*
N	*2.90165300*	*1.61187000*	*-0.42773900*
N	*2.75540800*	*-1.26908800*	*-0.72604500*
C	*3.71942700*	*-3.34155000*	*-0.90513700*
C	*4.97892100*	*2.53272700*	*-0.15934200*
H	*4.29845500*	*4.61017900*	*-0.14939100*
C	*4.23375000*	*1.31838600*	*-0.29601800*
C	*4.10864400*	*-1.15846400*	*-0.57065200*
C	*4.71634600*	*-2.45815700*	*-0.66918600*
H	*3.81340300*	*-4.40156000*	*-1.07127600*
H	*6.05084800*	*2.60312700*	*-0.07618600*
C	*4.80546000*	*0.03625600*	*-0.34537600*
H	*5.76898100*	*-2.66840900*	*-0.57825300*
C	*6.27721800*	*-0.05098500*	*-0.17117600*
C	*6.87718200*	*0.32541700*	*1.03096300*
C	*7.09780800*	*-0.50227900*	*-1.20549600*
C	*8.25083800*	*0.24837000*	*1.19627000*
C	*8.47177600*	*-0.57722000*	*-1.04260200*
C	*9.05367700*	*-0.20353900*	*0.15962400*
C	*1.11887100*	*-4.61985400*	*-1.25470900*
C	*1.61182500*	*-5.46363200*	*-0.25666800*
C	*0.53182000*	*-5.20891000*	*-2.37718400*
C	*1.51607600*	*-6.84073900*	*-0.37166800*

C	*0.43548400*	*-6.58574000*	*-2.49452200*
C	*0.92522000*	*-7.40841400*	*-1.49083500*
C	*1.69265100*	*5.18312000*	*-0.30620700*
C	*1.35271000*	*6.05599000*	*-1.34047500*
C	*2.12917900*	*5.72974800*	*0.90172000*
C	*1.44599700*	*7.42829700*	*-1.17281600*
C	*2.21968800*	*7.10160000*	*1.07193700*
C	*1.87887900*	*7.95658600*	*0.03432000*
H	*-5.94240100*	*3.16476400*	*1.40492600*
H	*-4.91110500*	*2.81514300*	*0.02208300*
O	*-5.28891500*	*-1.05558000*	*1.36759100*
H	*6.25063700*	*0.67805900*	*1.84161400*
H	*8.69501600*	*0.53958400*	*2.13976800*
H	*10.12686300*	*-0.26256800*	*0.28730200*
H	*9.09059100*	*-0.92345500*	*-1.86075900*
H	*6.64526000*	*-0.78826700*	*-2.14733500*
H	*2.39368900*	*5.06185800*	*1.71340600*
H	*2.55392600*	*7.50419000*	*2.01979900*
H	*1.95130300*	*9.02854000*	*0.16539800*
H	*1.18522100*	*8.08733000*	*-1.99128100*
H	*1.02119800*	*5.64438800*	*-2.28615900*
H	*0.15692800*	*-4.56954000*	*-3.16703200*
H	*-0.01757100*	*-7.01777700*	*-3.37780400*
H	*0.85070100*	*-8.48436200*	*-1.58196000*
H	*1.89984600*	*-7.47299300*	*0.41955600*
H	*2.06464500*	*-5.02332900*	*0.62360200*
Co	*1.38370600*	*0.27099900*	*-0.59667400*
N	*-0.91752700*	*-0.21130600*	*3.54546600*

H	*0.78458600*	*-1.38096500*	*3.77927900*
H	*0.29408100*	*-0.70303500*	*1.07043200*
C	*-0.12625000*	*0.99796500*	*3.62306500*
H	*-0.74946400*	*1.82548700*	*3.94717100*
H	*0.66674700*	*0.83428500*	*4.35273000*
C	*0.46238800*	*1.21971900*	*2.20261600*
H	*-0.33307200*	*1.64956400*	*1.59295600*
H	*1.24905200*	*1.97448700*	*2.26553200*
C	*0.96535200*	*-0.03582500*	*1.59508100*
H	*1.93539500*	*-0.43378400*	*1.86228900*

9. NMR Titration Experiments

In the following, the basic premises and the experimental data are presented. A detailed description of the practical procedures and the theoretical background of NMR titrations, as well as the synthesis and determination of the dimerization constant of the model substrate **8** can be found in the supplementary information to *Org. Biomol. Chem.* **2011**, *9*, 5316-5329.[28]

9.1. Determination of the Dimerization Constant K_{dim} of Catalyst 7

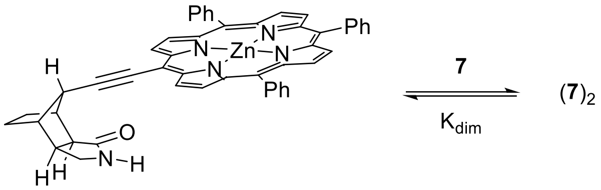

7

Self-association of catalyst **7** at the lactam binding motif should be rather small, since the bulky porphyrin units would severely interact upon binding.[29] To validate this hypothesis, the equilibrium concentrations were planned to be determined by dilution experiments with catalyst **7** and investigated by observing the change in chemical shift of the NH signal of the catalyst. For an initial experiment, a comparatively low concentration range from 7.5 10^{-5} mol L^{-1} and 7.5 10^{-3} mol L^{-1} of **7** in toluene-d_8 was chosen.

Entry	$[7]_0$ [mol/l]	δ_{NH} [ppm]
1	7.500E-05	4.479
2	1.050E-04	4.458
3	1.463E-04	4.435
4	1.969E-04	4.415
5	2.813E-04	4.381
6	3.938E-04	4.345
7	5.438E-04	4.314
8	7.500E-04	4.280
9	1.050E-03	4.244
10	1.463E-03	4.210
11	2.063E-03	4.178
12	2.813E-03	4.148
13	3.938E-03	4.111
14	5.438E-03	4.078
15	7.500E-03	4.048

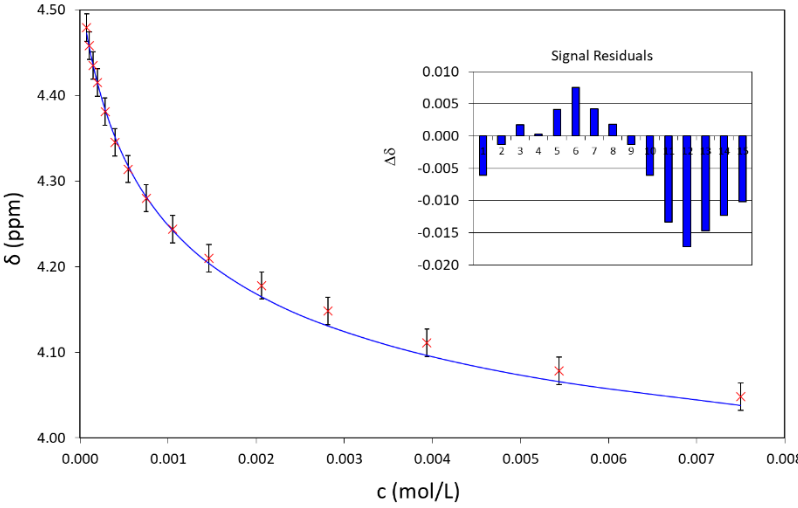

Intriguingly, an inverse shift of the NH signal of the lactam binding motif was observed as compared to previous examples from our group.[30-33] This suggests that the lactam binding site is not involved in the dimerization of catalyst **7**. Therefore, no self-association of catalyst **7** *via* the lactam binding site is assumed for further calculations.

9.2. Determination of the Association Constant K_a of Model Substrate **8**

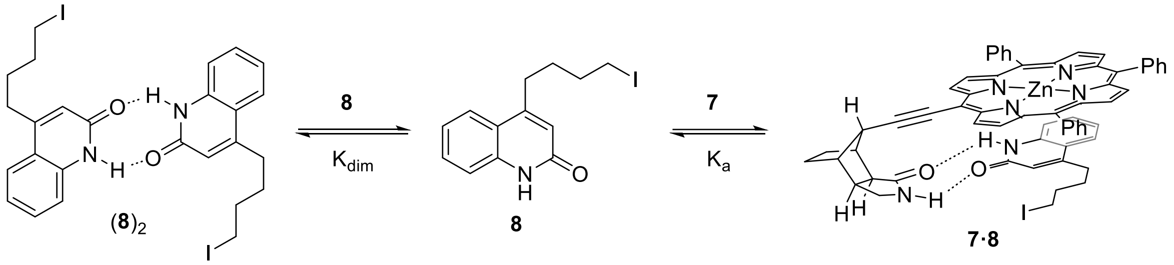

Zinc porphyrin **7** was chosen as a suitable surrogate for the NMR non-active cobalt catalyst of the reaction in the applied concentration range. The chemical shift of the NH signal of the catalyst was observed for the determination of the association constant K_a. The dimerization constant of **8** (K_{dim} = 2001 ± 133 L mol^{-1} at 298 K) was adopted from the literature.[28] From previous investigations,[30] an estimate of 2500 L mol^{-1} for the association constant for **7·8** was reasonable as starting point for the determination of a suitable concentration range. The catalyst concentration was kept constant at 1.5 10^{-4} mol L^{-1} to keep dilution effects negligible.[34] Consequently, ten samples with concentrations ranging from 0.0 mol L^{-1} and 2.5 10^{-3} mol L^{-1} of **8** in toluene-d_8 were analyzed. The chemical shift of the NH proton of sample 10 could not be determined due to overlaps with other signals.

Entry	$[7]_0$ [mol L^{-1}]	δ_{NH} [ppm]
1	0.00E+00	4.448
2	2.50E-05	4.624
3	4.50E-05	4.763
4	7.81E-05	4.944
5	1.41E-04	5.215
6	2.50E-04	5.551
7	4.50E-04	5.919
8	7.81E-04	6.286
9	1.41E-03	6.616
10	2.50E-03	-*

*not determined due to overlaps with other signals

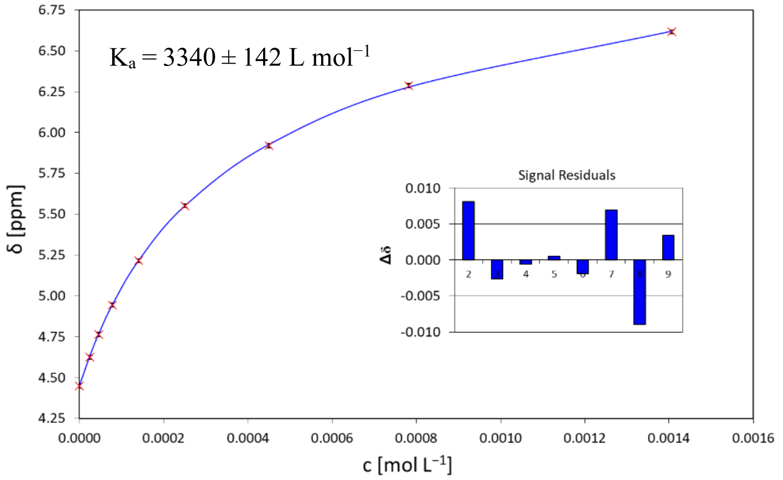

For the obtained data and the boundary conditions described above, the best fit curve was determined using non-linear regression. For the association constant, a value of 3340 L mol^{-1} was obtained.

Monta Carlo analysis was used to check the robustness of the best fit curve.[28, 35] Therefore, 1000 data sets were calculated, in which the individual data points deviate randomly and normally distributed with the standard deviation (σ = 0.006 ppm) of the measured data set around the best fit values. The association constant was calculated for each of the 1000 generated datasets, and, subsequently, from these values the mean (3348 L mol^{-1}), standard deviation (σ = 55 L mol^{-1}) and 95% confidence interval (± 142 L mol^{-1}).

9.3. Determination of the Catalyst Occupancy

The catalyst occupancy can be calculated as follows:

$$Catalyst\ Occupancy = \frac{[\mathbf{7} \cdot \mathbf{8}]}{[\mathbf{7} \cdot \mathbf{8}] + [\mathbf{7}]} = \frac{[\mathbf{7} \cdot \mathbf{8}]}{[\mathbf{7}]_0}$$

While the initial concentration of catalyst **7** is known, the equilibrium concentration of complex **7·8** needs to be determined from the following equations:

The dimerization constant is determined as

$$K_{dim} = \frac{[\mathbf{8_2}]}{[\mathbf{8}]^2}$$

and the association constant as

$$K_a = \frac{[\mathbf{7} \cdot \mathbf{8}]}{[\mathbf{7}][\mathbf{8}]}$$

With the boundaries of the concentrations

$$[\mathbf{7}]_0 = [\mathbf{7}] + [\mathbf{7} \cdot \mathbf{8}]$$

and

$$[\mathbf{8}]_0 = [\mathbf{8}] + 2[\mathbf{8}_2] + [\mathbf{7} \cdot \mathbf{8}]$$

the concentration of substrate **8** can be calculated depending on the initial concentrations of the catalyst ($[\mathbf{7}]_0$) and the substrate ($[\mathbf{8}]_0$), and the dimerization constant K_{dim} and the association constant K_a according to the following equation:

$$0 = K_a^2[\mathbf{7} \cdot \mathbf{8}]^3 + (2K_{dim} - K_a - 2[\mathbf{7}]_0K_a^2 - [\mathbf{8}]_0K_a^2)[\mathbf{7} \cdot \mathbf{8}]^2 + ([\mathbf{7}]_0K_a + [\mathbf{7}]_0^2K_a^2 + 2[\mathbf{7}]_0[\mathbf{8}]_0K_a^2)[\mathbf{7} \cdot \mathbf{8}] - [\mathbf{7}]_0^2[\mathbf{8}]_0K_a^2$$

Solving this cubic equation with the initial concentrations under the previously described reaction conditions ($[\mathbf{7}]_0$ = 0.2 mM, $[\mathbf{8}]_0$ = 20 mM, K_a = 2001 L mol^{-1}, K_{dim} = 3340 L mol^{-1}), the concentration of complex **7·8** was calculated to be 0.1751 mM. Therefore, the catalyst occupancy is calculated to be 88% at room temperature.

10. NMR Spectra of New Compounds

10.1. Catalyst Synthesis

*10.1.1. 5,10,15-Triphenylporphyrin zinc(II) (**SI-4b**)*

^{1}H NMR (500 MHz, $CDCl_3$):

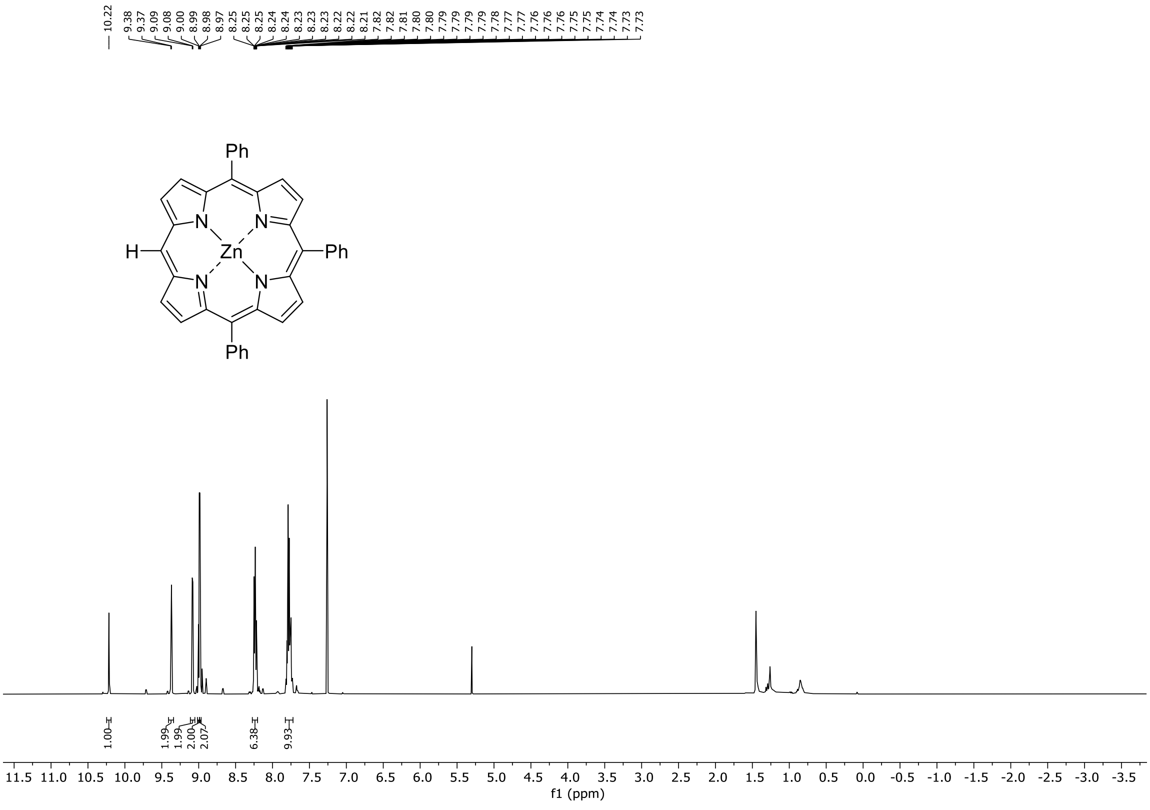

^{13}C NMR (126 MHz, $CDCl_3$):

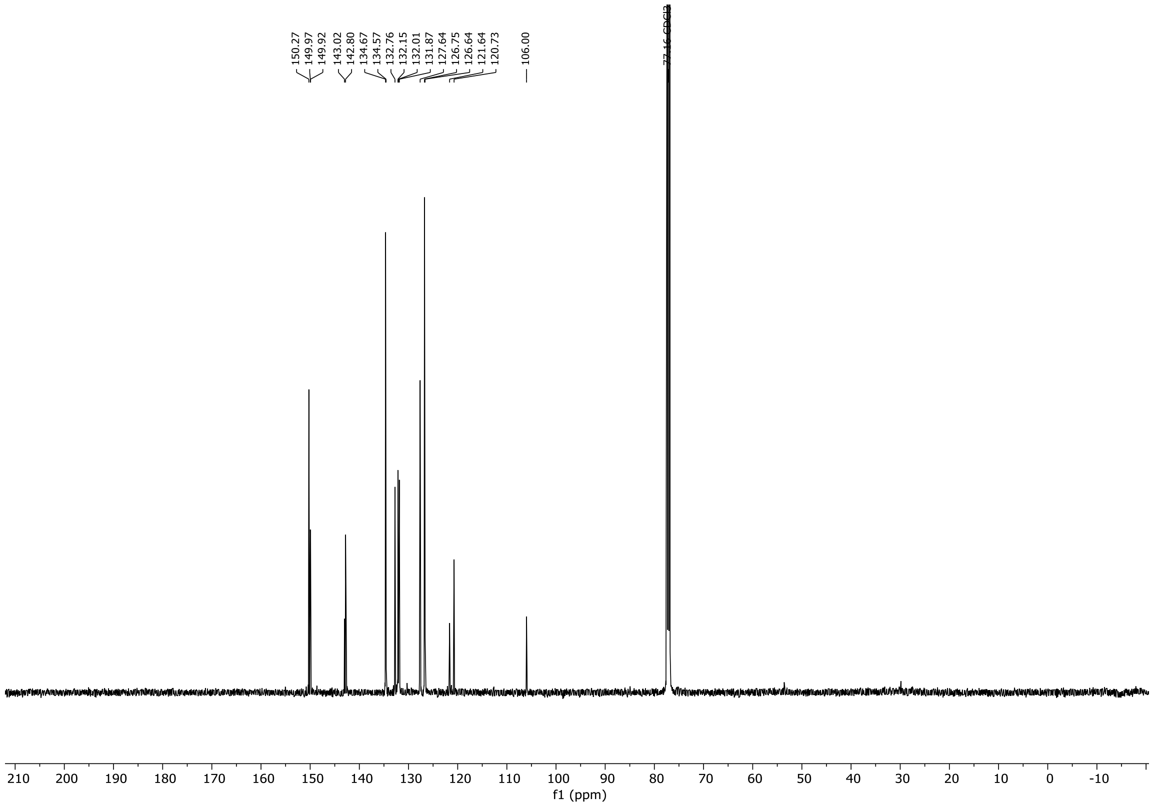

10.1.2. 5,15-Bis(4-methoxyphenyl)-10-phenylporphyrin zinc(II) ***(SI-4d)***

^{1}H NMR (500 MHz, $CDCl_3$):

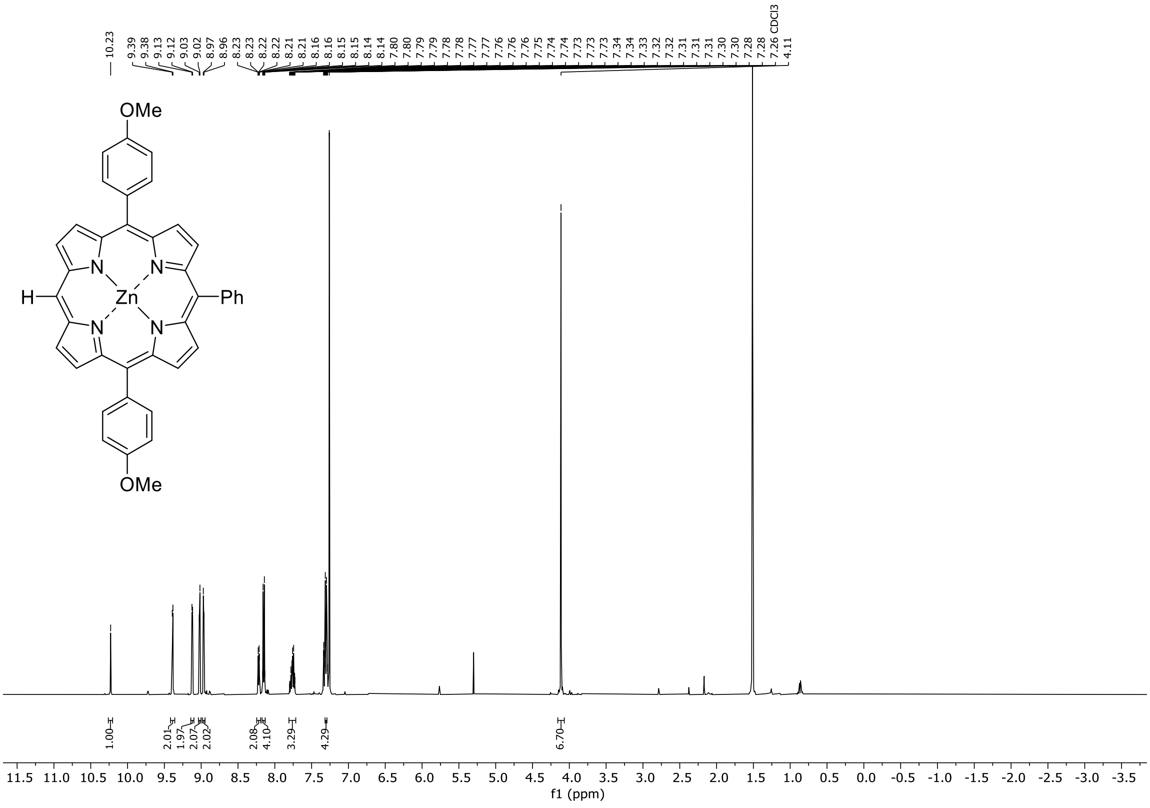

^{13}C NMR (126 MHz, $CDCl_3$):

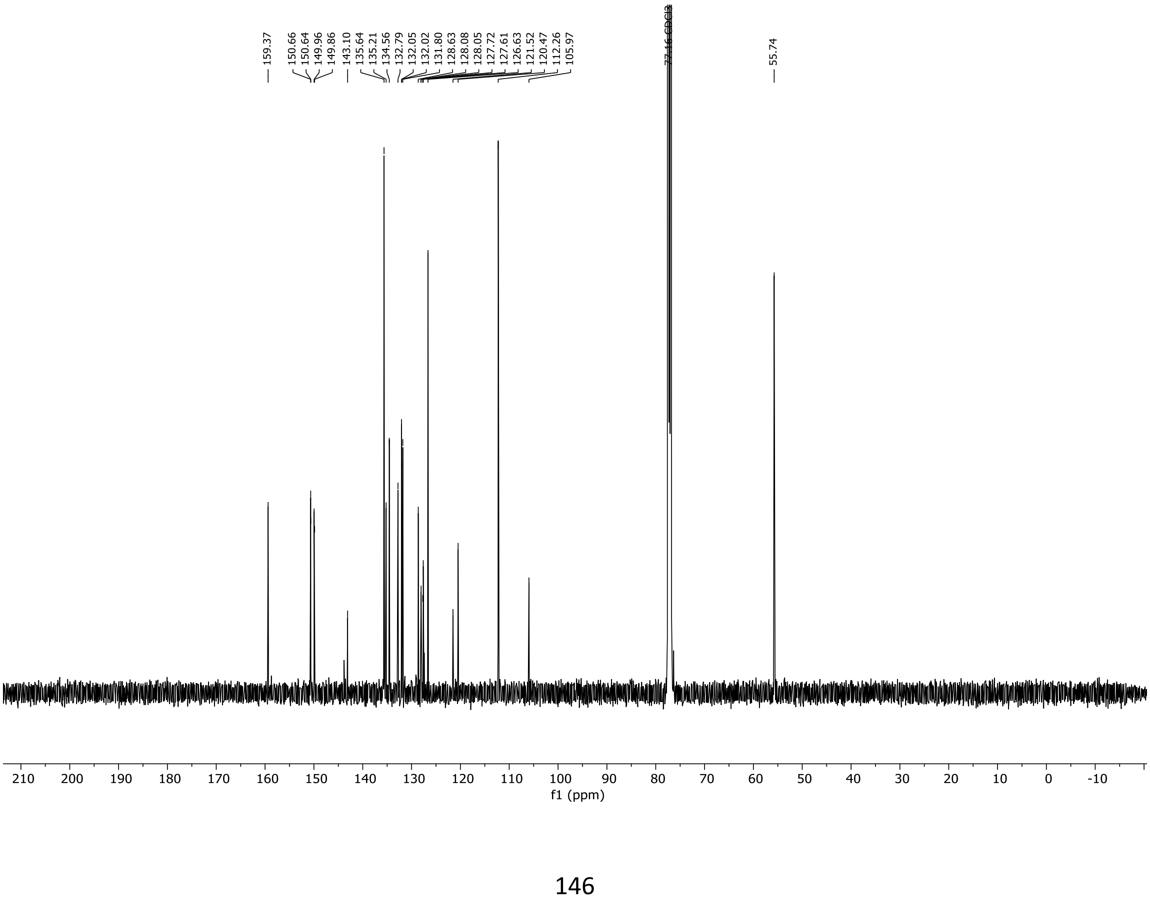

10.1.3. 5-((3aS,7aR,8R)-8-Ethynyl)octahydro-1H-4,7-methanoisoindol-1-on-9-yl)-10,15,20-triphenylporphyrin zinc(II) (7)

^{1}H NMR (500 MHz, $CDCl_3$):

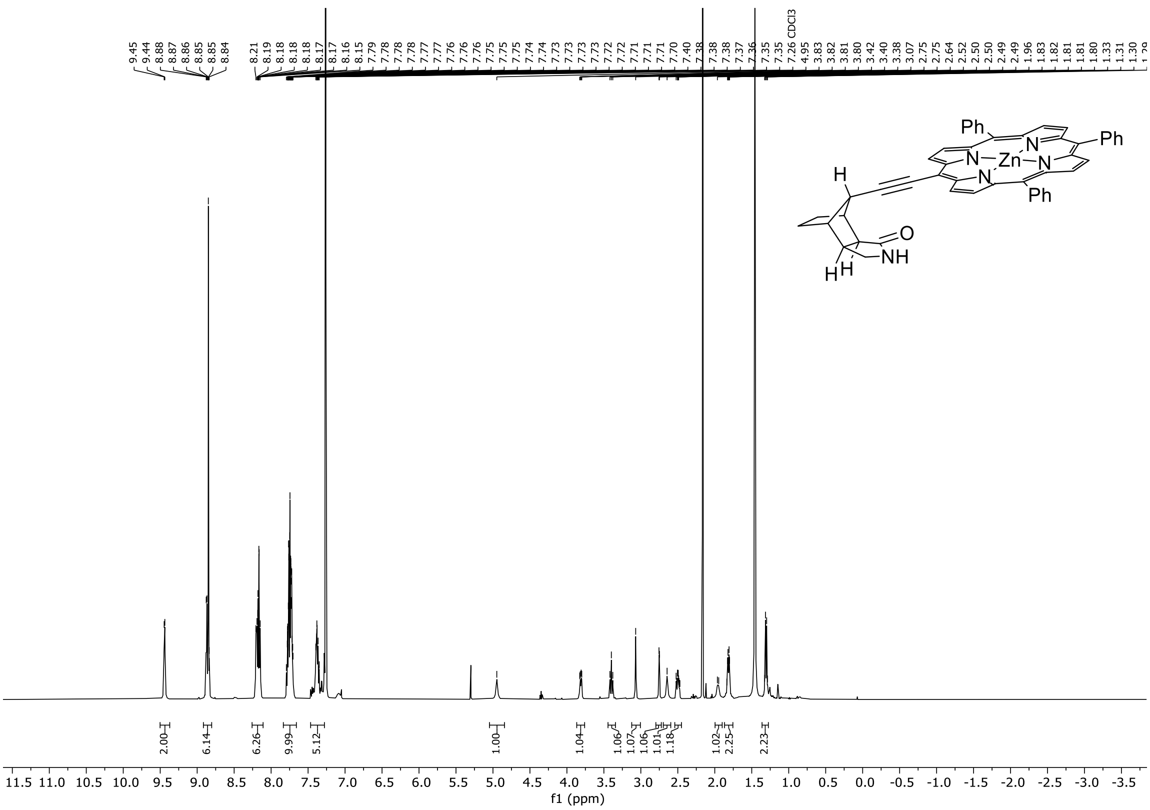

^{13}C NMR (126 MHz, $CDCl_3$):

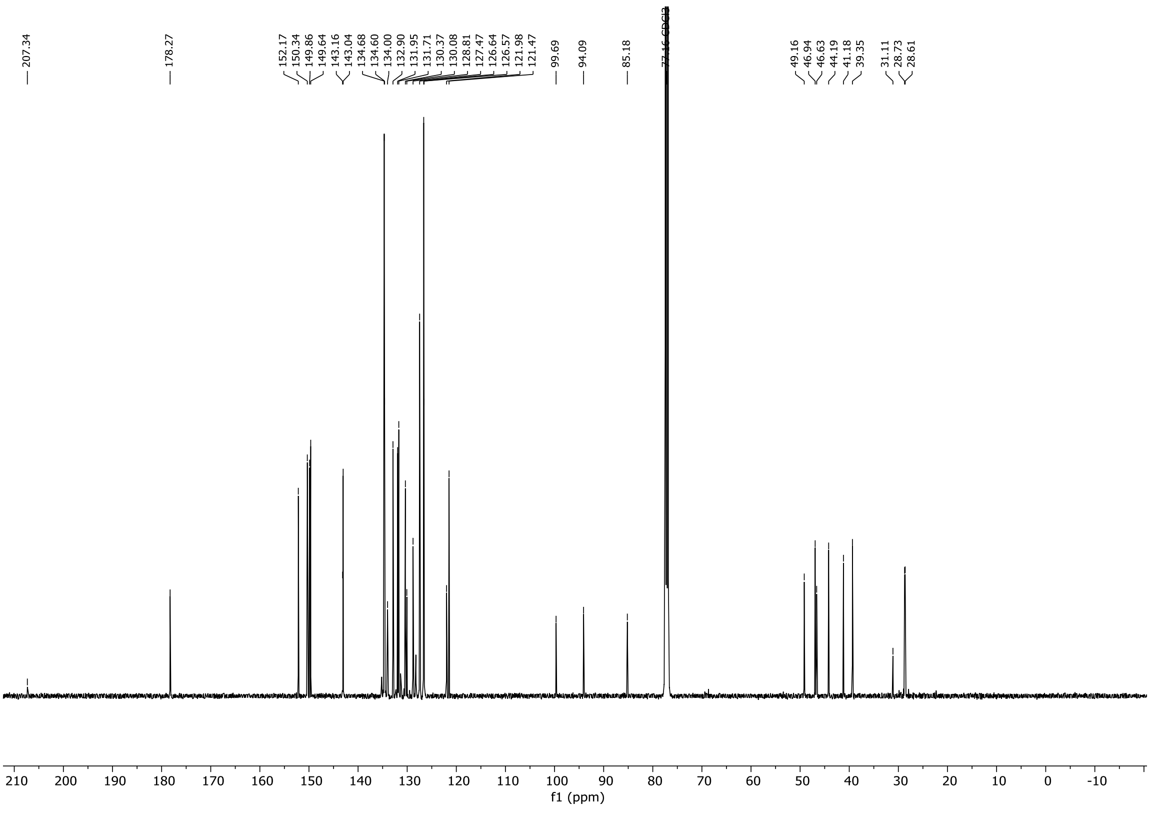

*10.1.4. 5-((3aS,7aR,8R)-8-Ethynyl)octahydro-1H-4,7-methanoisoindol-1-on-9-yl)-10,15,20-triphenylporphyrin (SI-7**b**)*

^{1}H NMR (500 MHz, $CDCl_3$):

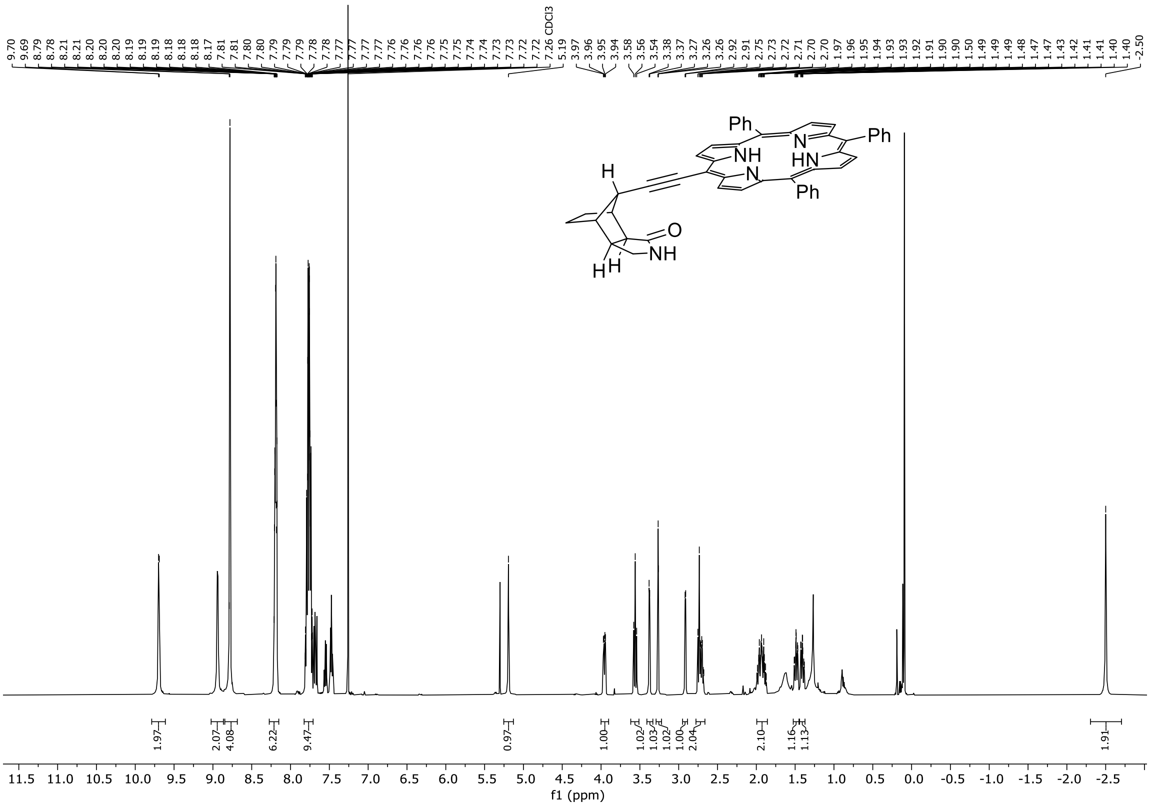

^{13}C NMR (126 MHz, $CDCl_3$):

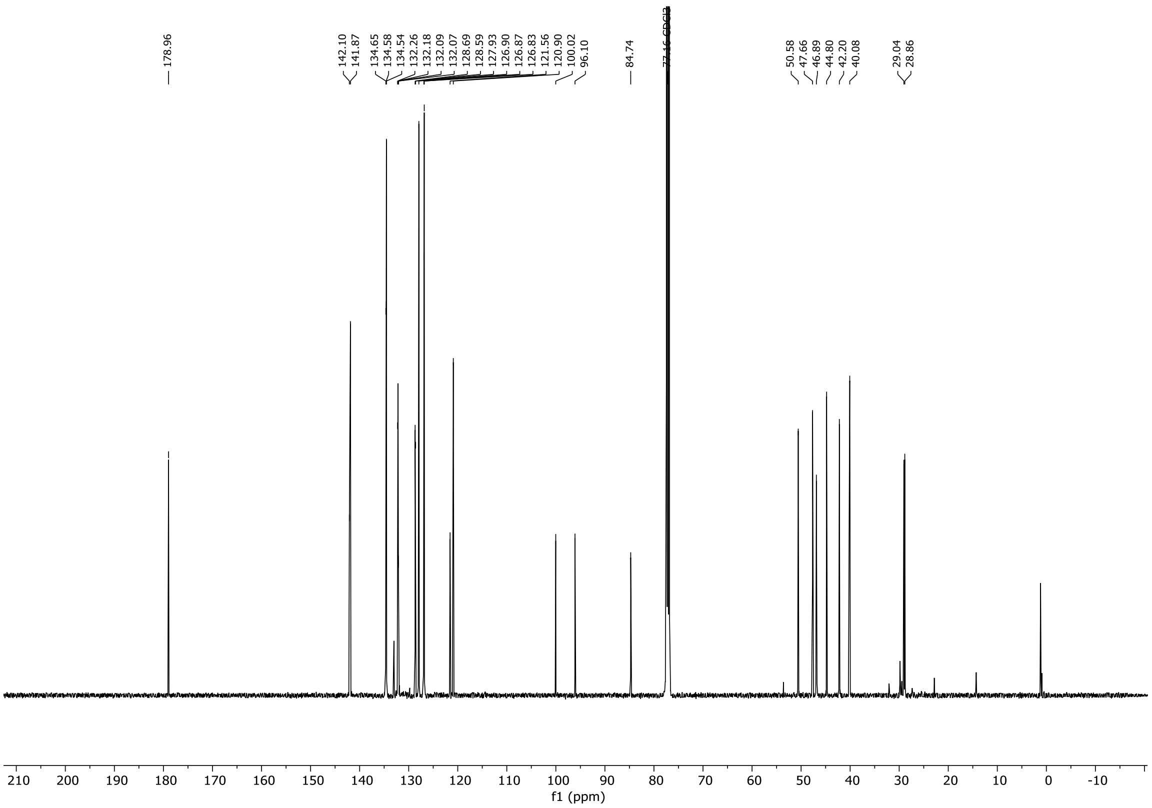

*10.1.5. 5-((3aS,7aR,8R)-8-Ethynyl)octahydro-1H-4,7-methanoisoindol-1-on-9-yl)-10,20-dimesityl-15-phenylporphyrin (SI-7**c**)*

^{1}H NMR (500 MHz, $CDCl_3$):

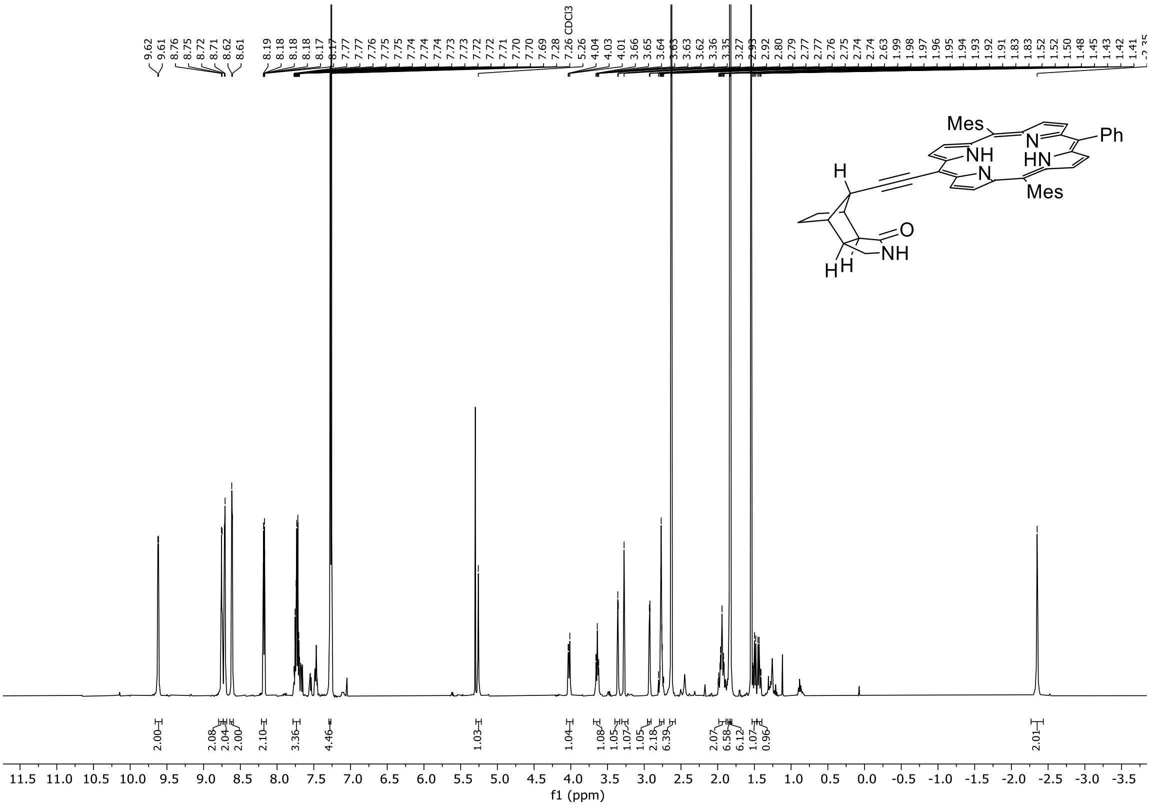

^{13}C NMR (126 MHz, $CDCl_3$):

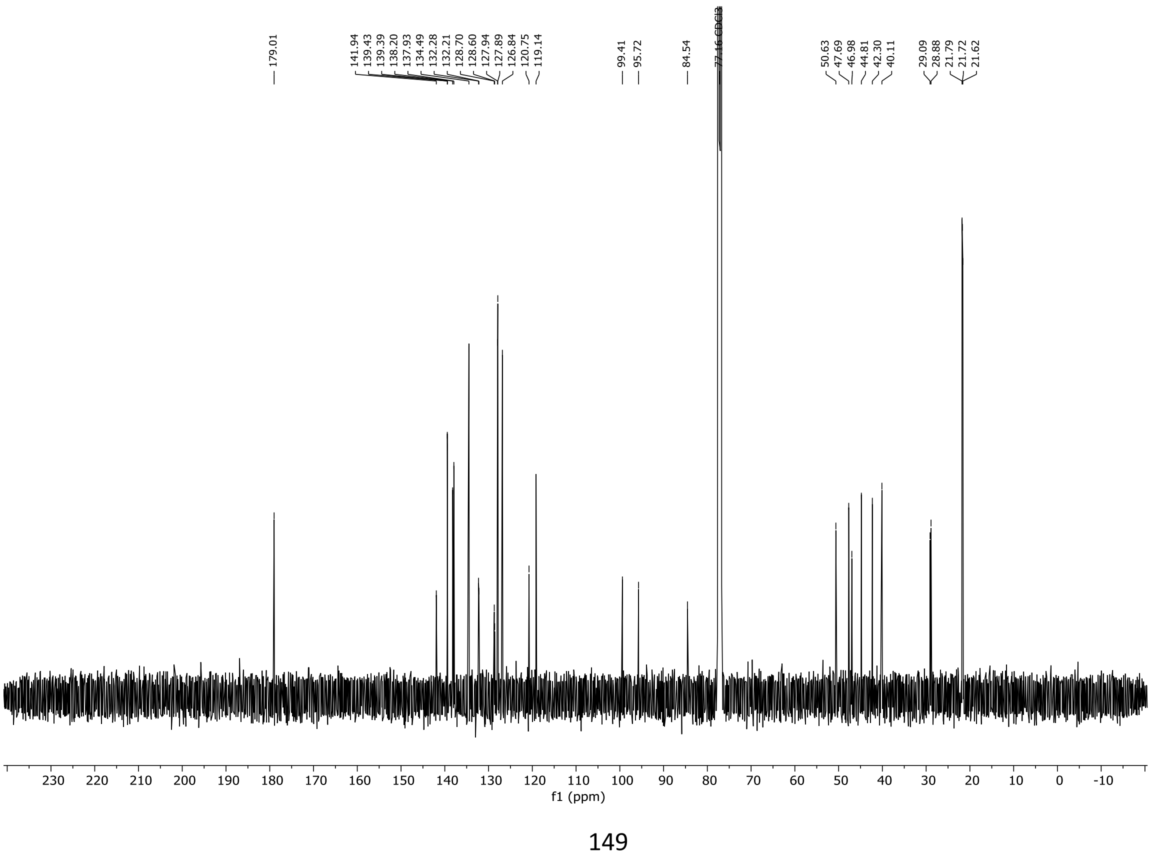

*10.1.6. 5-((3aS,7aR,8R)-8-Ethynyl)octahydro-1H-4,7-methanoisoindol-1-on-9-yl)-10,20-bis(4-methoxyphenyl)-15-phenylporphyrin (SI-7**d**)*

^{1}H NMR (500 MHz, $CDCl_3$):

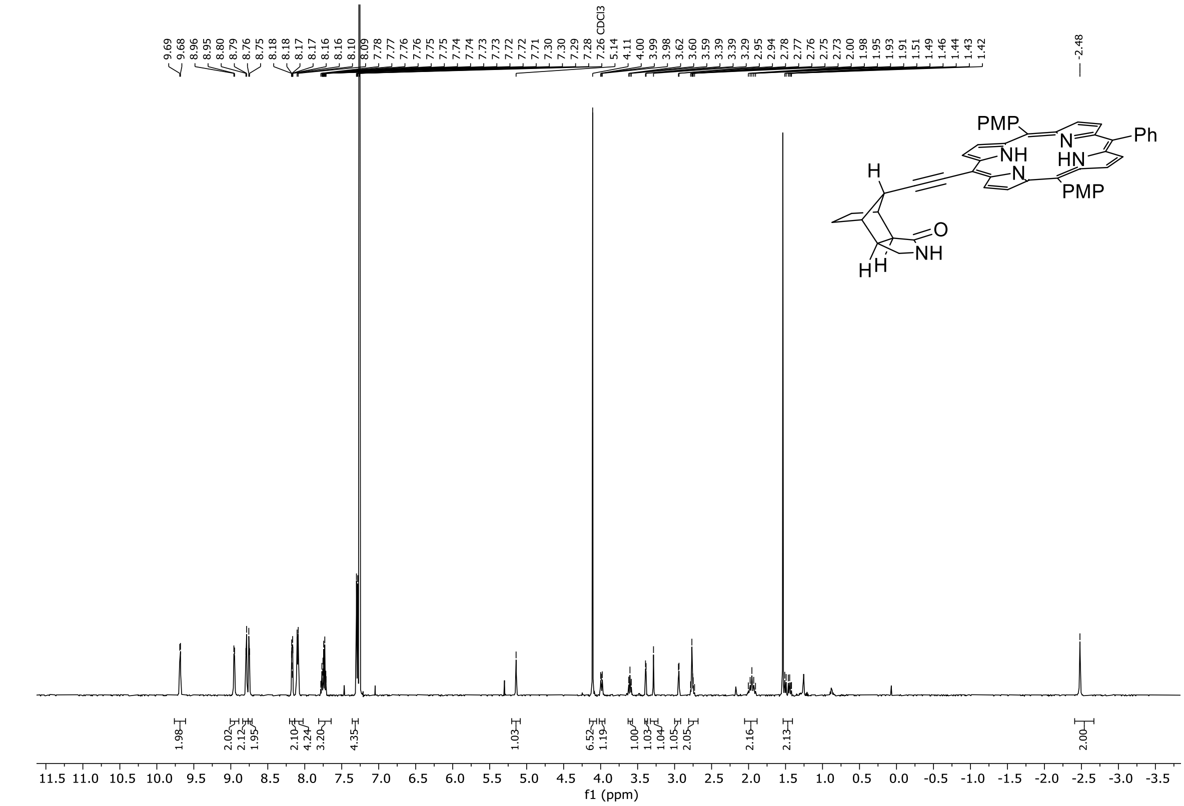

^{13}C NMR (126 MHz, $CDCl_3$):

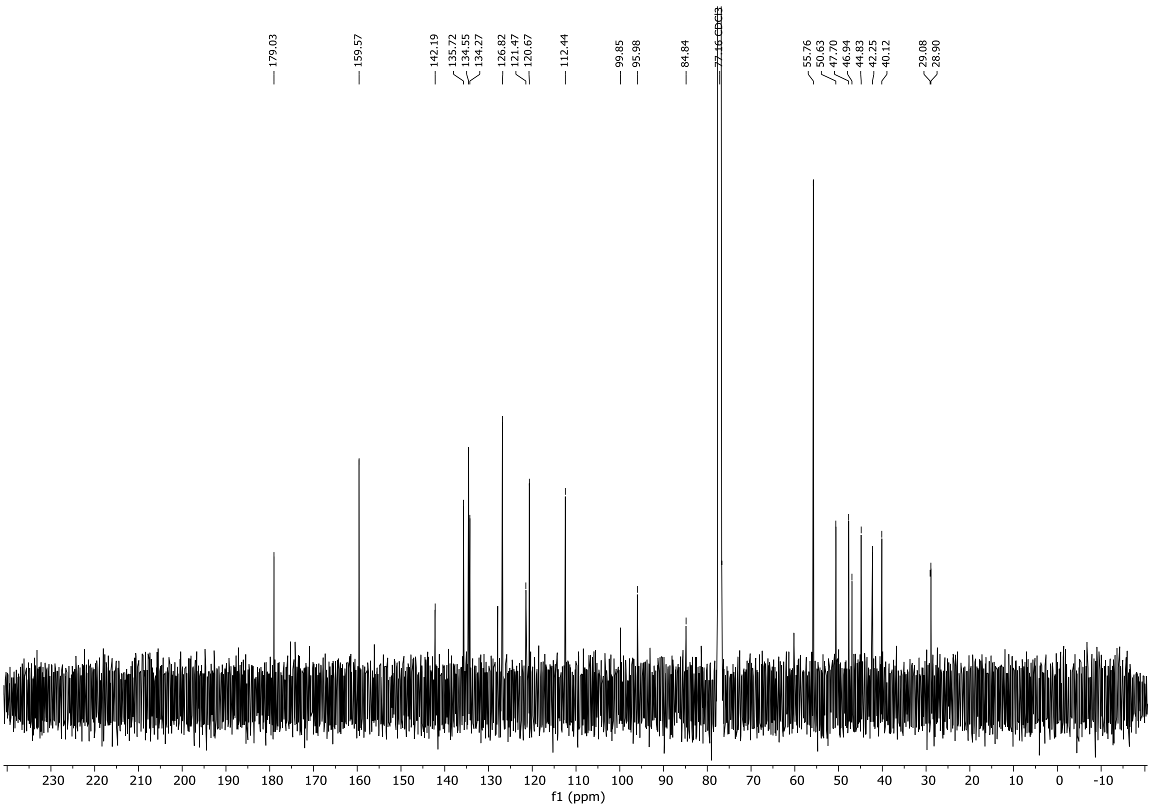

10.2. Alkylated 3,4-Dihydroquinazolin-2(1H)-ones

*10.2.1. 3-(3,3-Diethoxypropyl)-3,4-dihydroquinazolin-2(1H)-one (**SI-8a**)*

^{1}H NMR (500 MHz, $CDCl_3$):

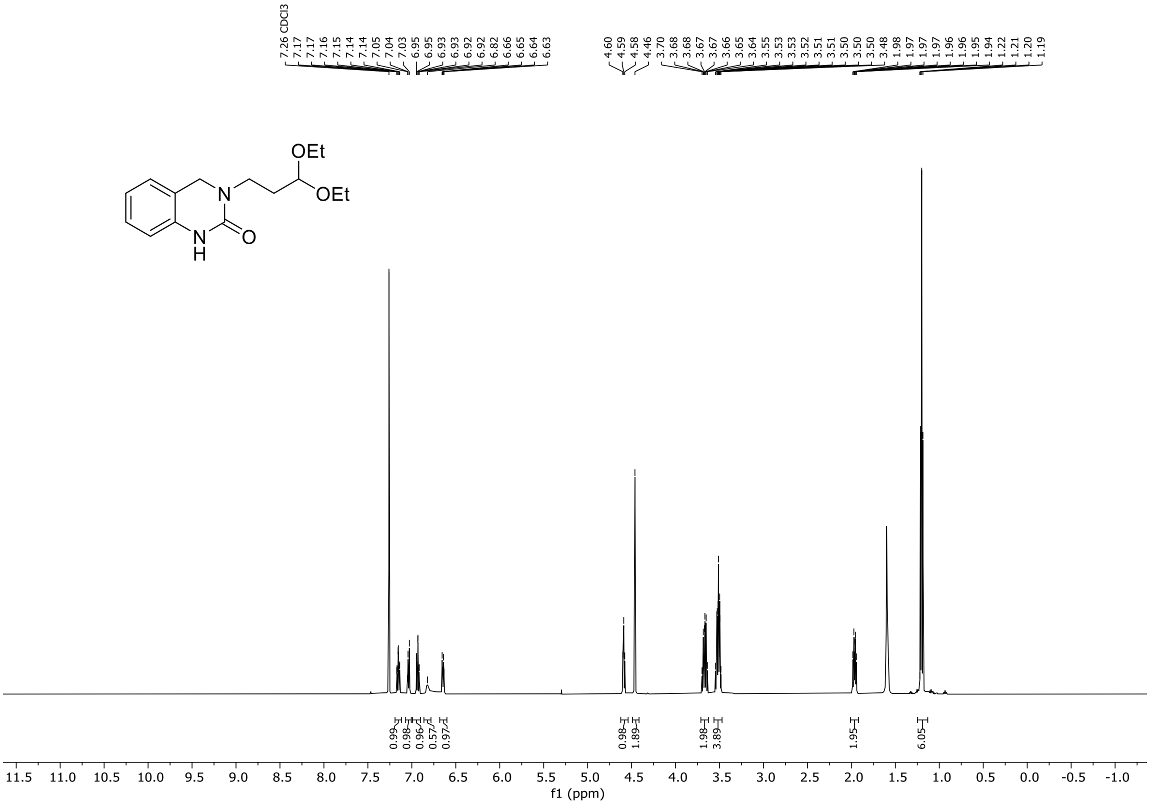

^{13}C NMR (126 MHz, $CDCl_3$):

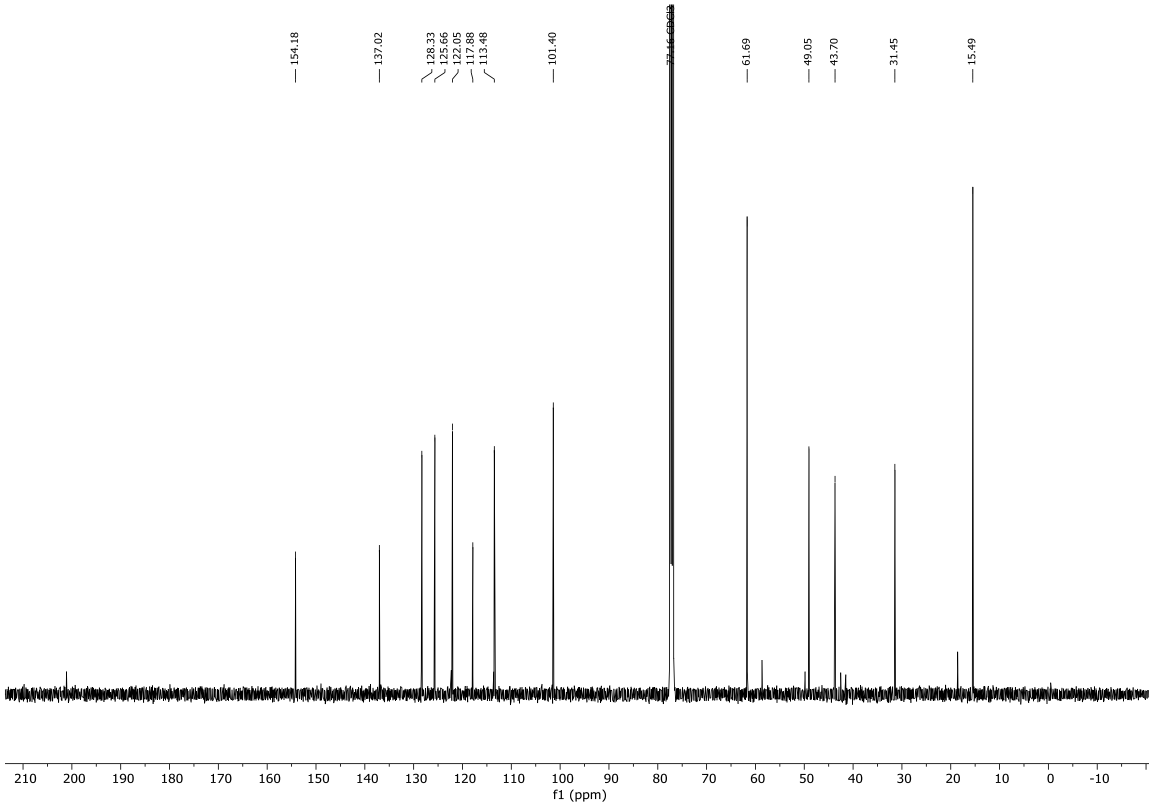

*10.2.2. 5-Bromo-3-(3,3-diethoxypropyl)-3,4-dihydroquinazolin-2(1H)-one (**SI-8b**)*

^{1}H NMR (500 MHz, $CDCl_3$):

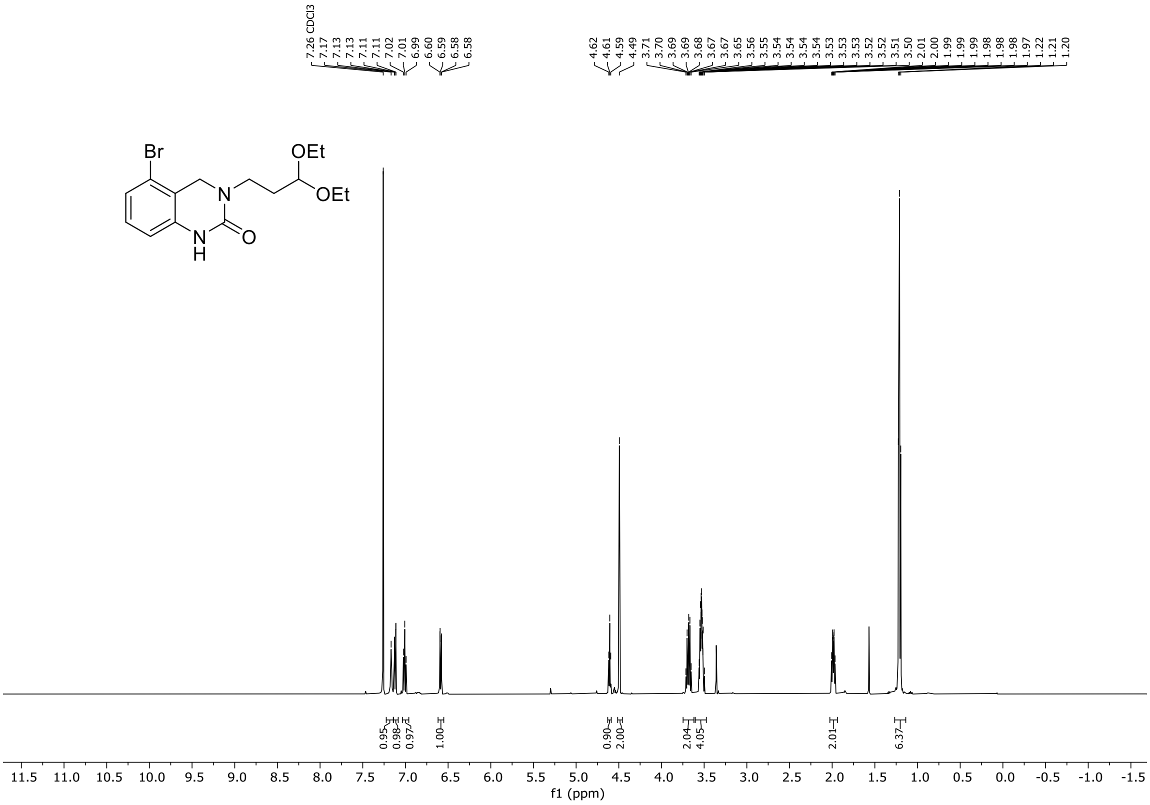

^{13}C NMR (126 MHz, $CDCl_3$):

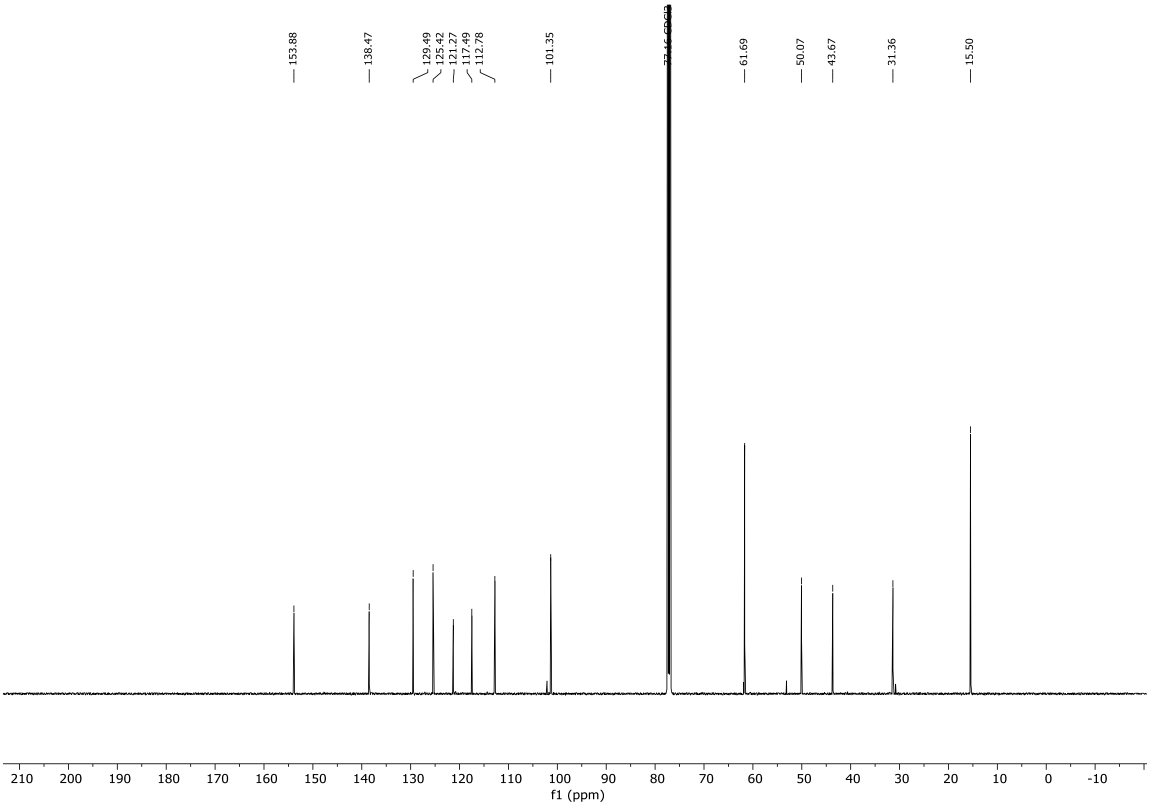

*10.2.3. 6-Bromo-3-(3,3-diethoxypropyl)-3,4-dihydroquinazolin-2(1H)-one (**SI-8c**)*

^{1}H NMR (500 MHz, $CDCl_3$):

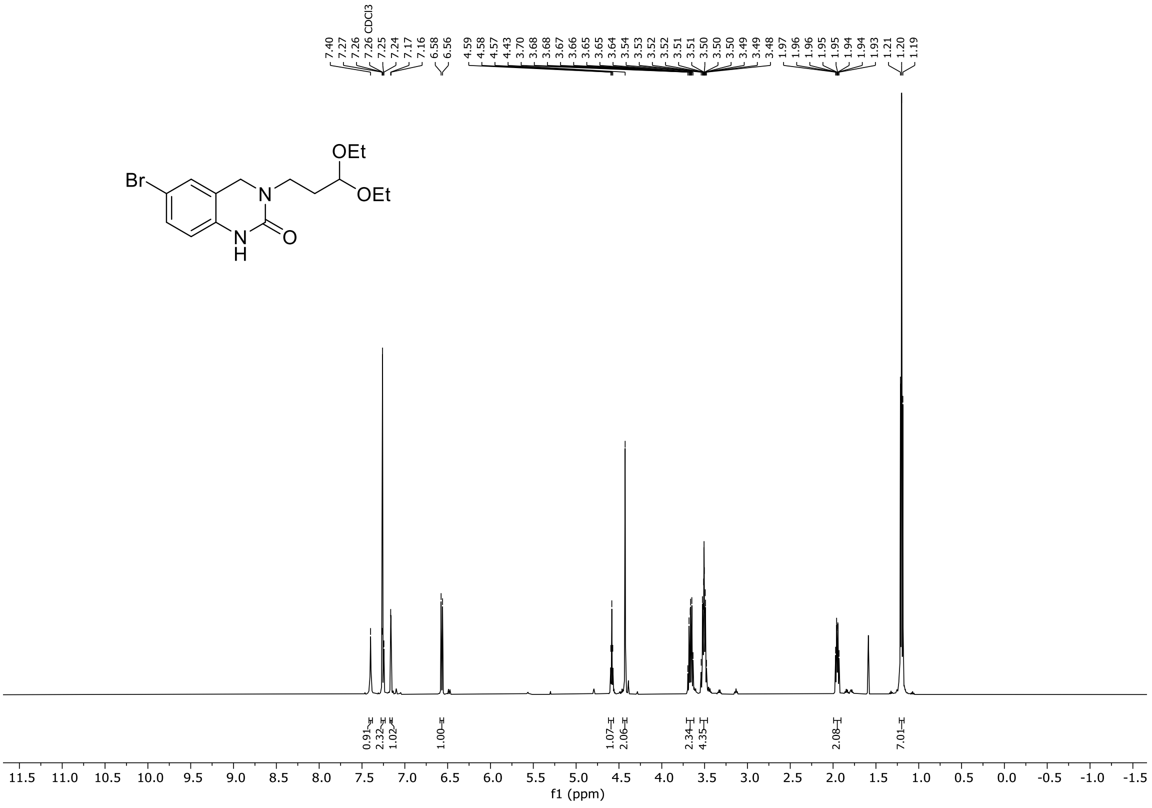

^{13}C NMR (126 MHz, $CDCl_3$):

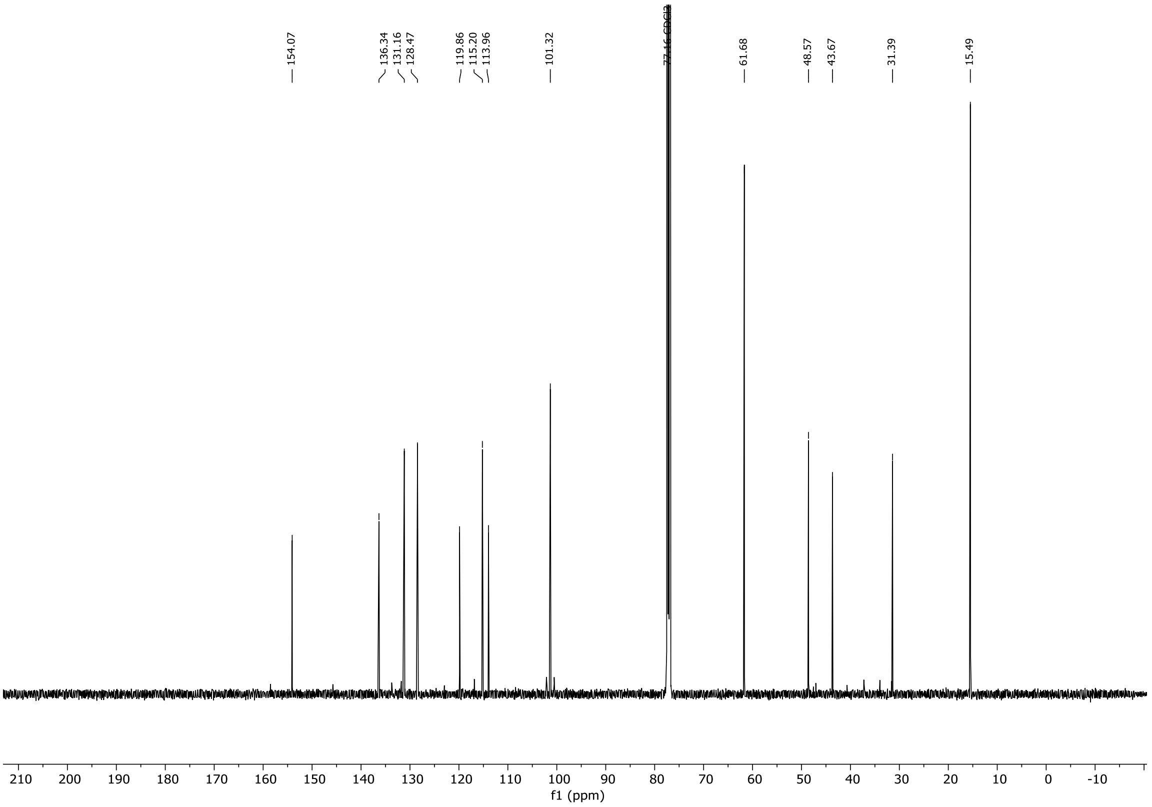

*10.2.4. 7-Bromo-3-(3,3-diethoxypropyl)-3,4-dihydroquinazolin-2(1H)-one (**SI-8d**)*

^{1}H NMR (500 MHz, $CDCl_3$):

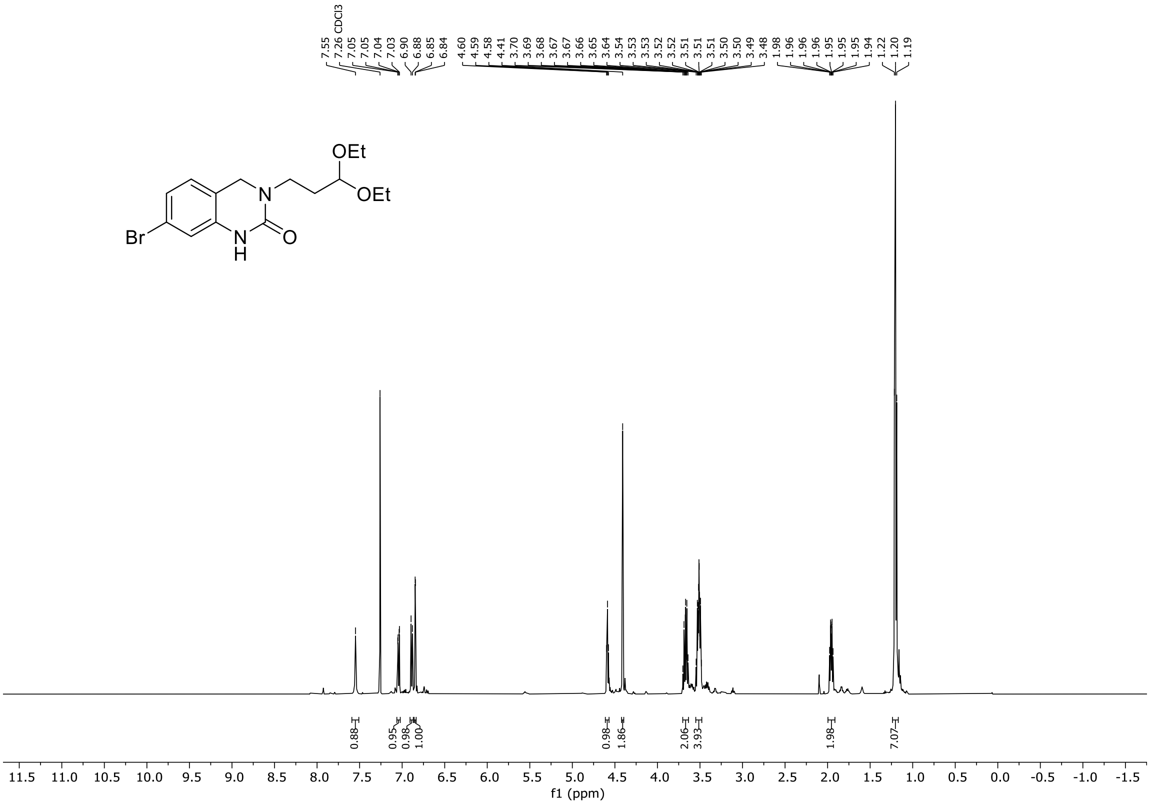

^{13}C NMR (126 MHz, $CDCl_3$):

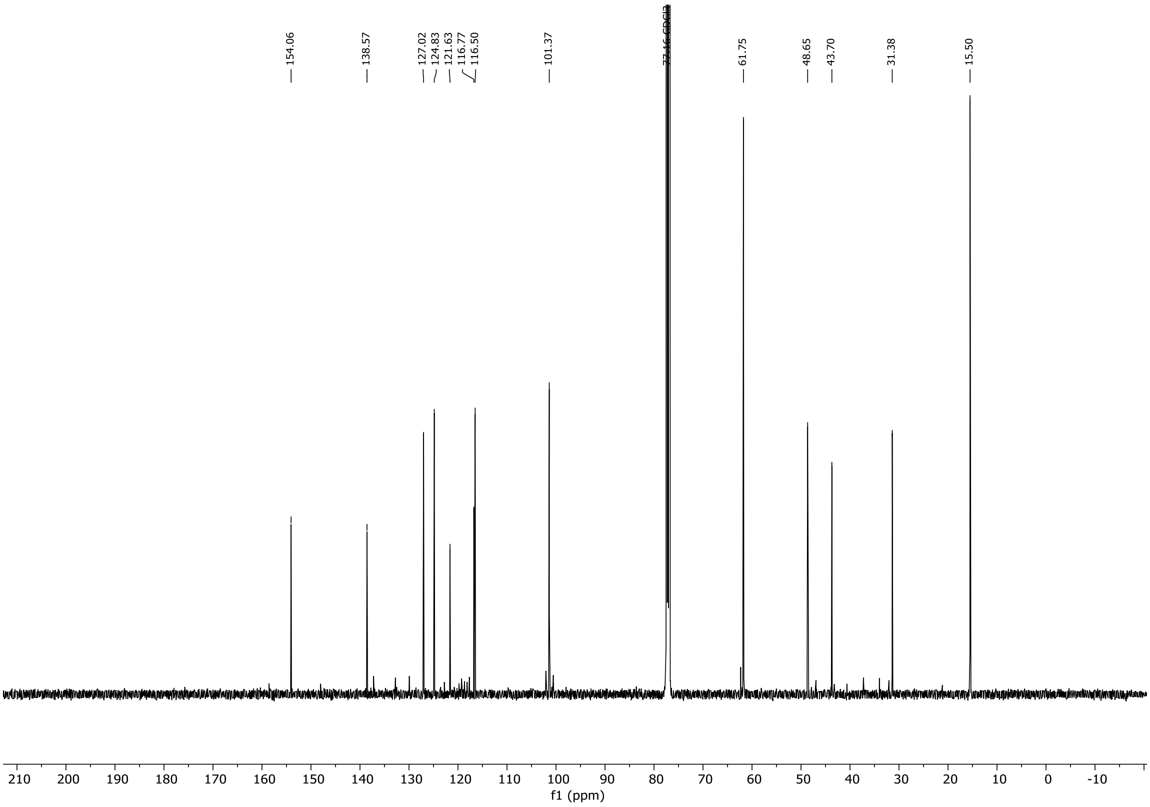

*10.2.5. 8-Bromo-3-(3,3-diethoxypropyl)-3,4-dihydroquinazolin-2(1H)-one (**SI-8e**)*

^{1}H NMR (500 MHz, $CDCl_3$):

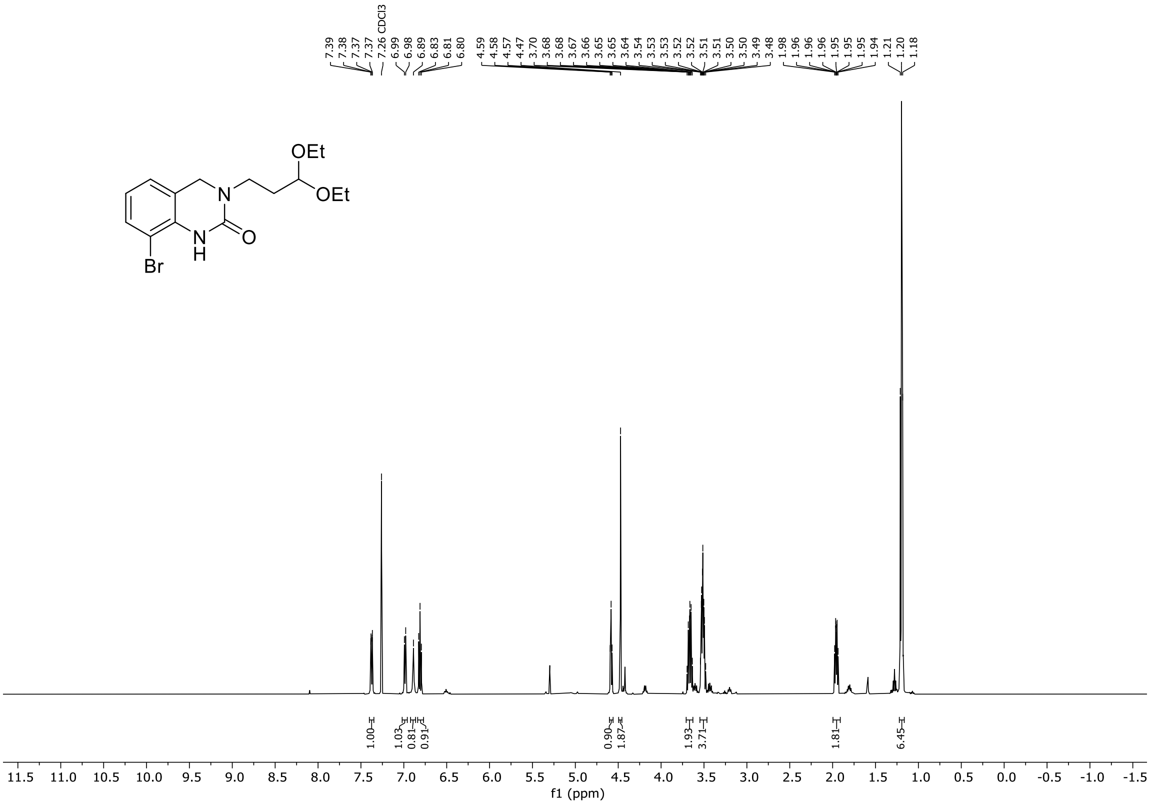

^{13}C NMR (126 MHz, $CDCl_3$):

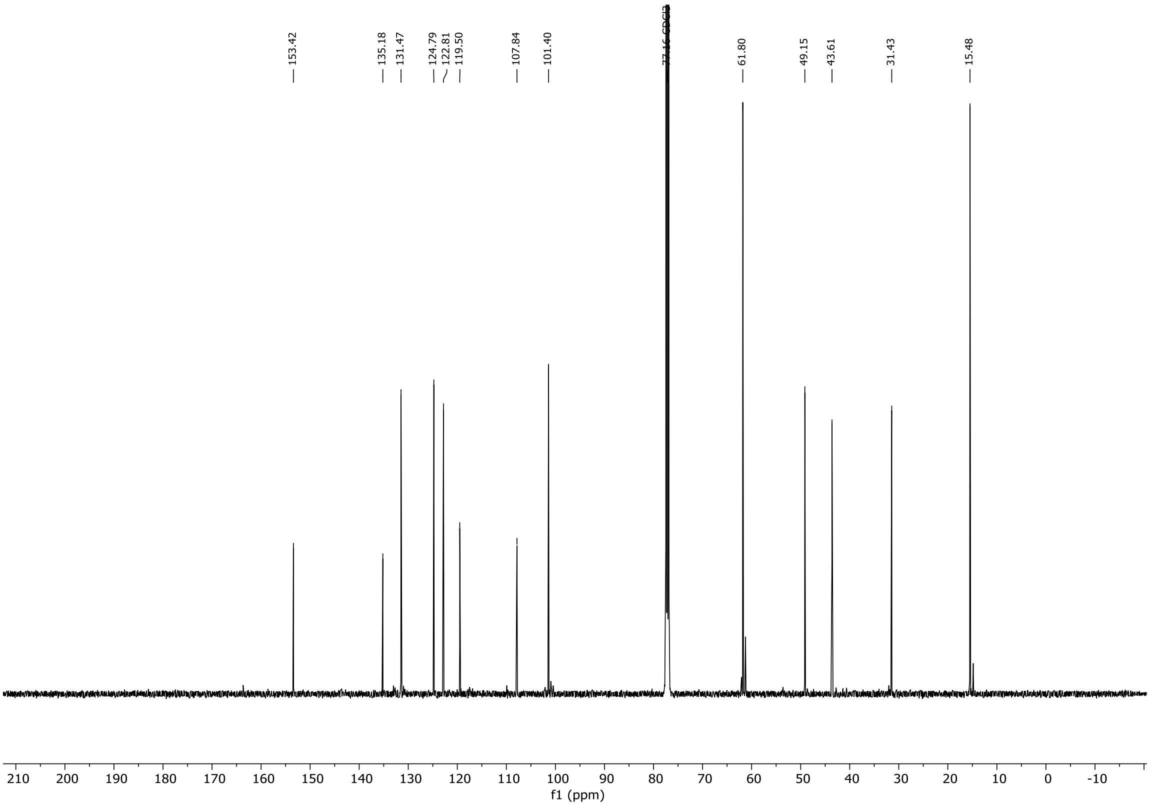

*10.2.6. 6-Chloro-3-(3,3-diethoxypropyl)-3,4-dihydroquinazolin-2(1H)-one (**SI-8f**)*

^{1}H NMR (500 MHz, $CDCl_3$):

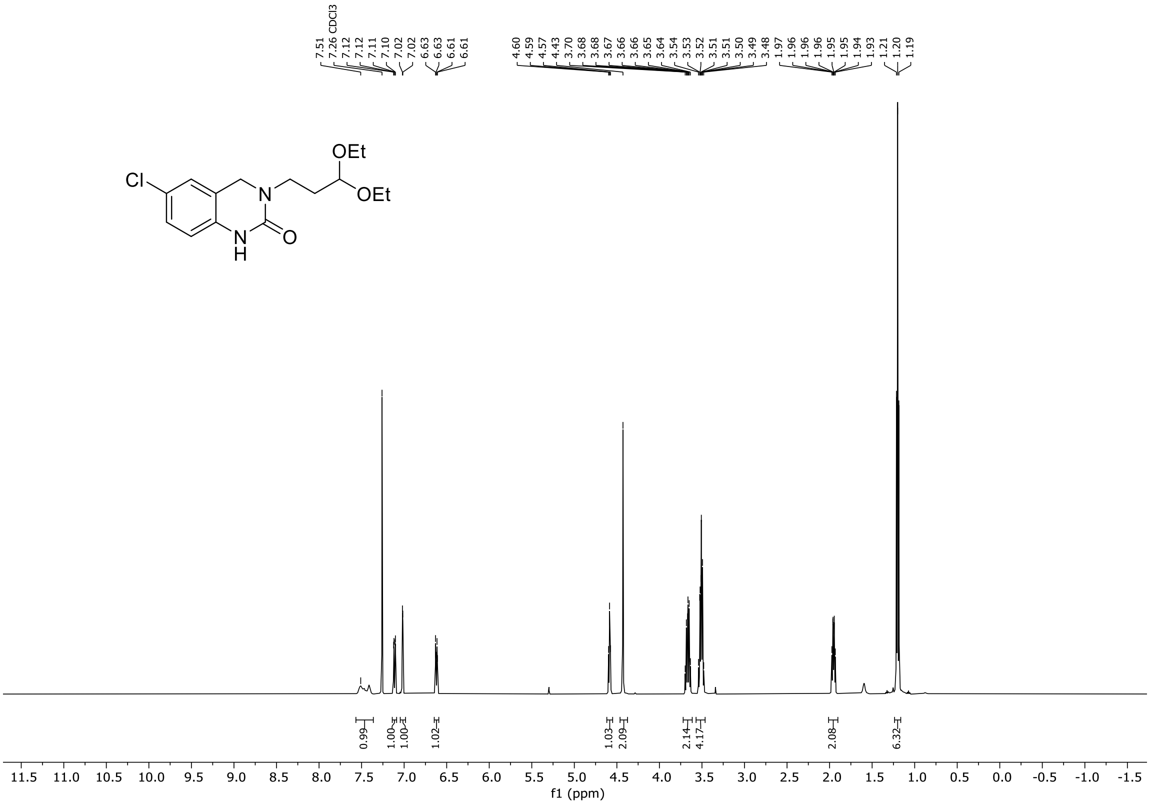

^{13}C NMR (126 MHz, $CDCl_3$):

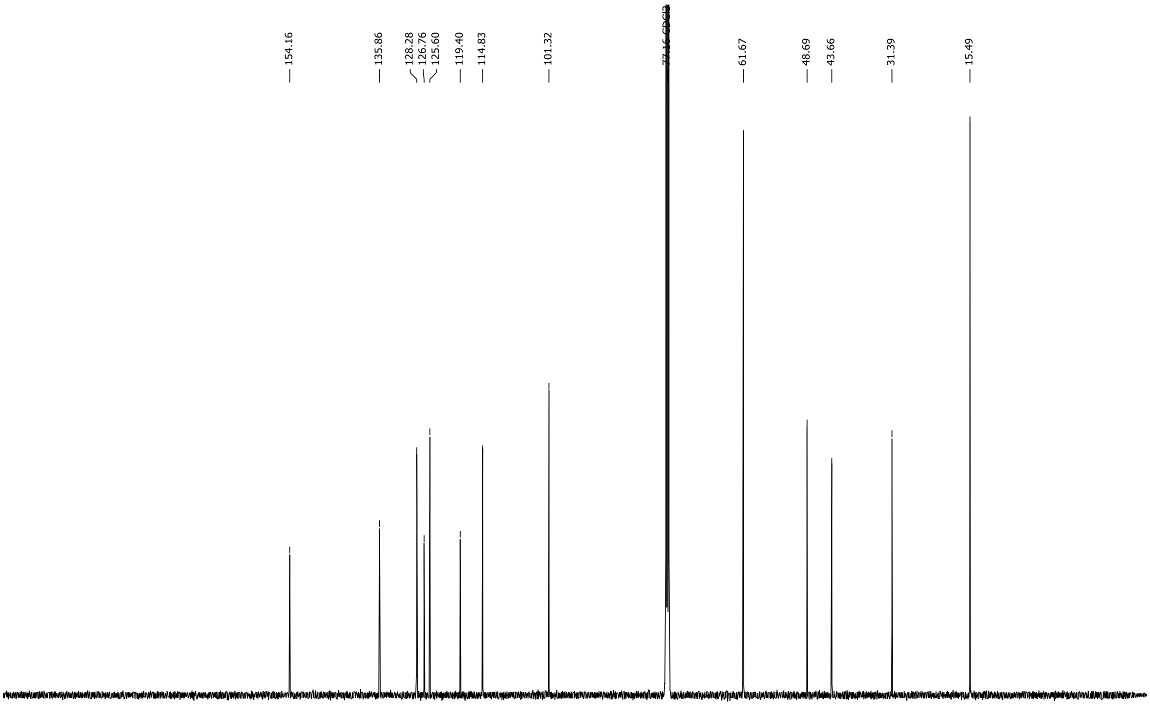

*10.2.7. 3-(3,3-Diethoxypropyl)-7-fluoro-3,4-dihydroquinazolin-2(1H)-one (**SI-8g**)*

^{1}H NMR (500 MHz, $CDCl_3$):

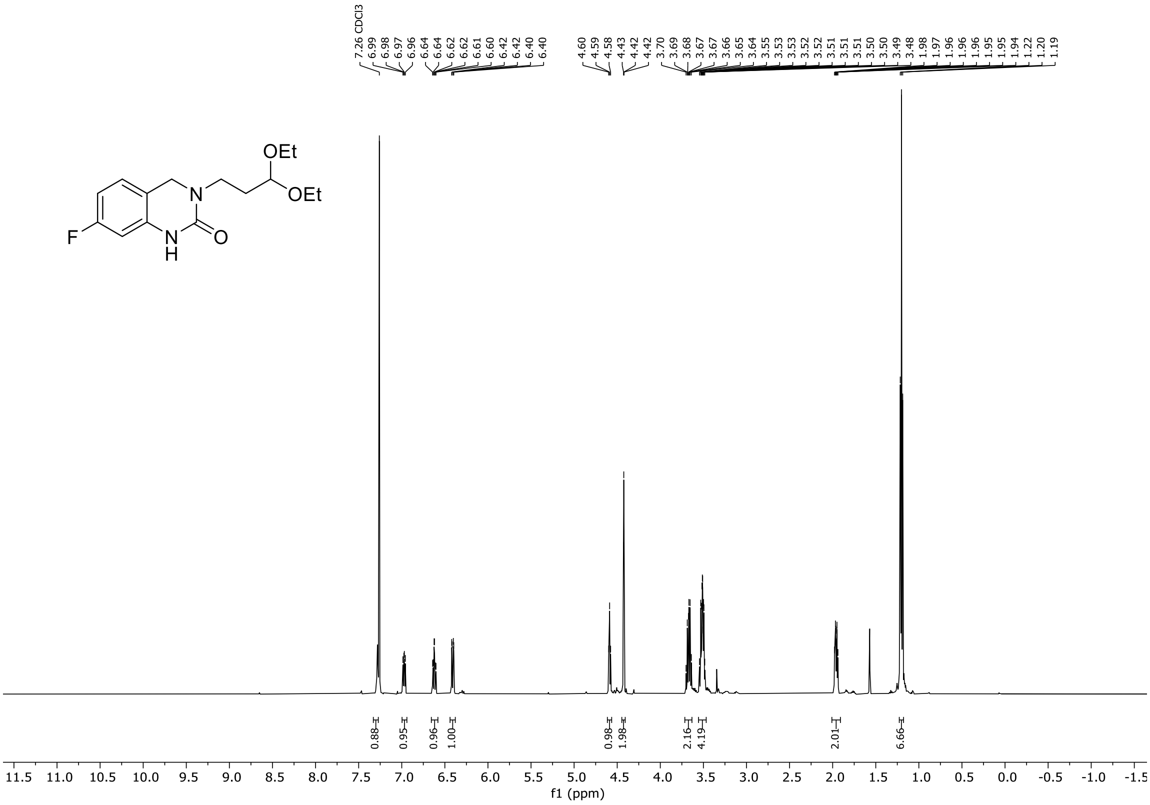

^{13}C NMR (126 MHz, $CDCl_3$):

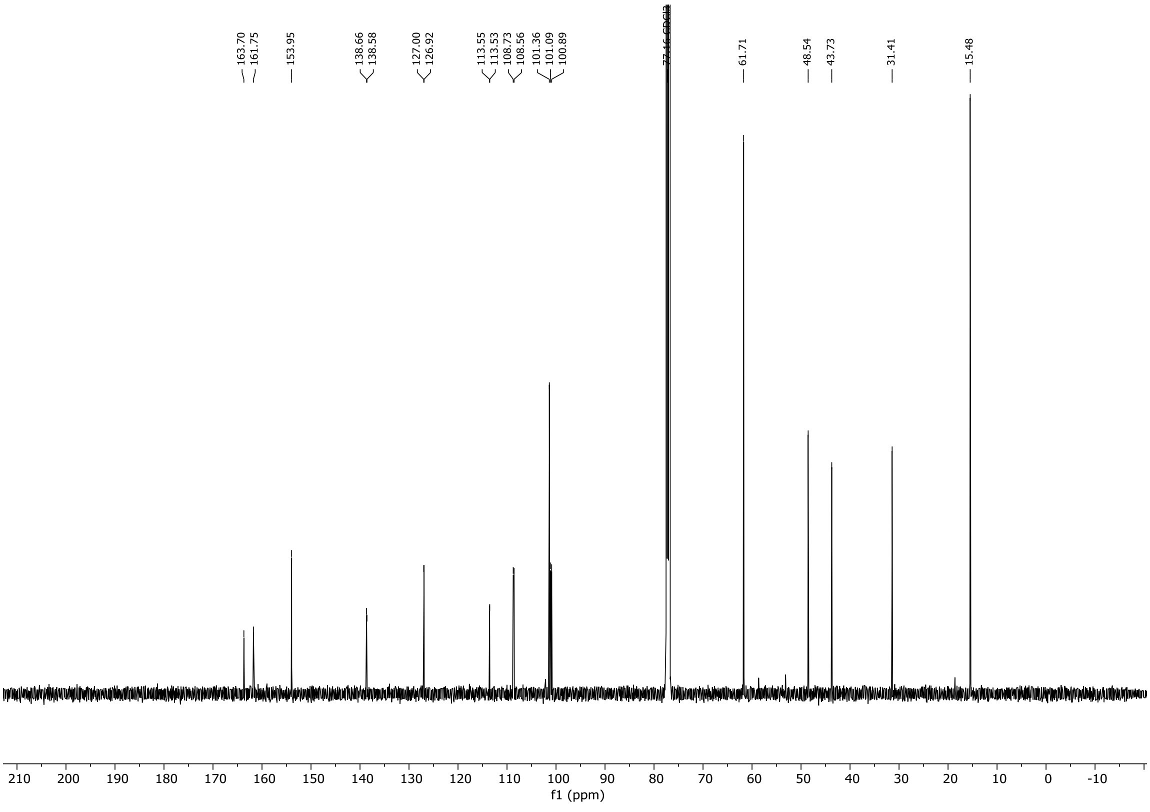

^{19}F NMR (471 MHz, $CDCl_3$):

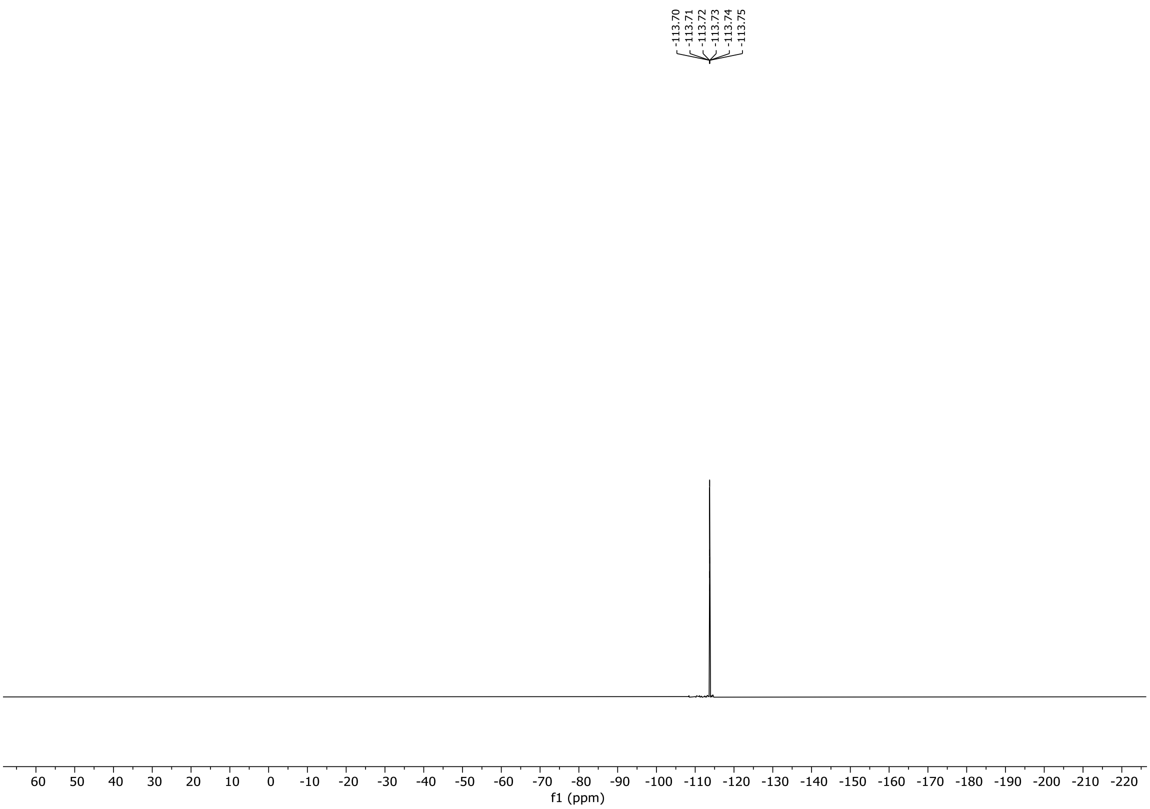

*10.2.8. 3-(3,3-Diethoxypropyl)-7-(trifluoromethyl)-3,4-dihydroquinazolin-2(1H)-one (**SI-8h**)*

^{1}H NMR (500 MHz, $CDCl_3$):

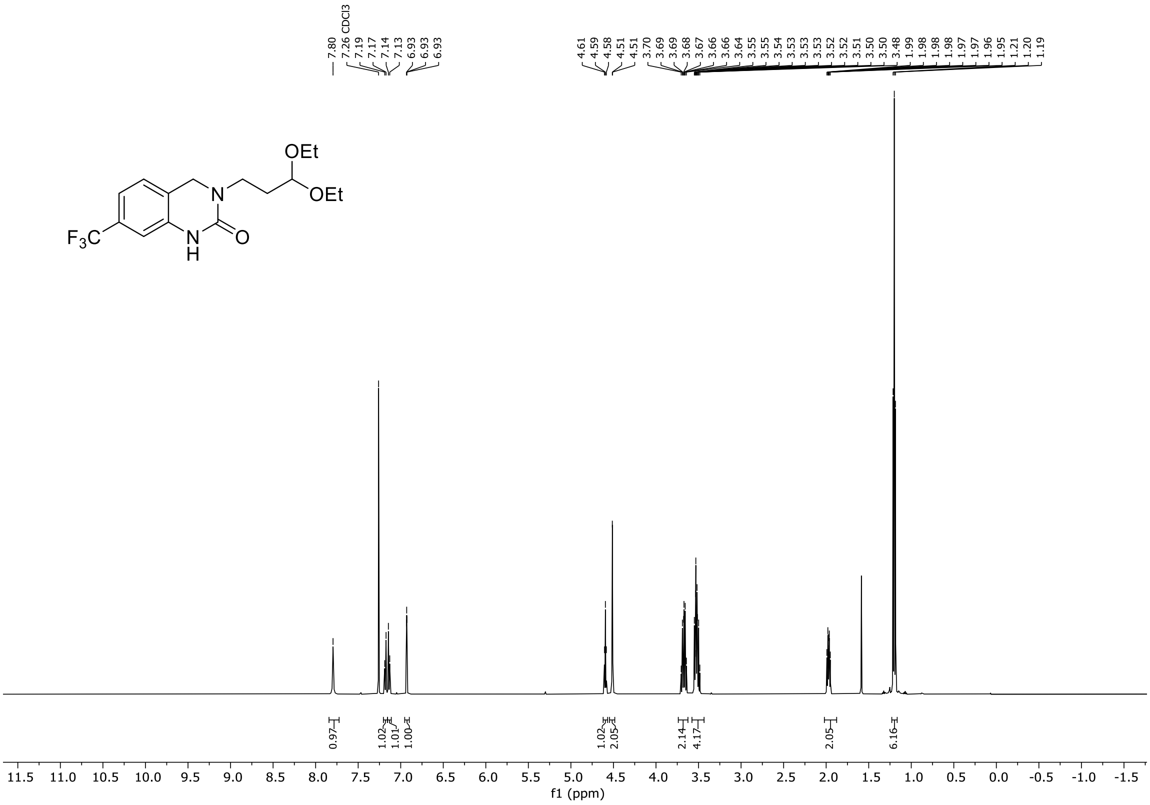

^{13}C NMR (126 MHz, $CDCl_3$):

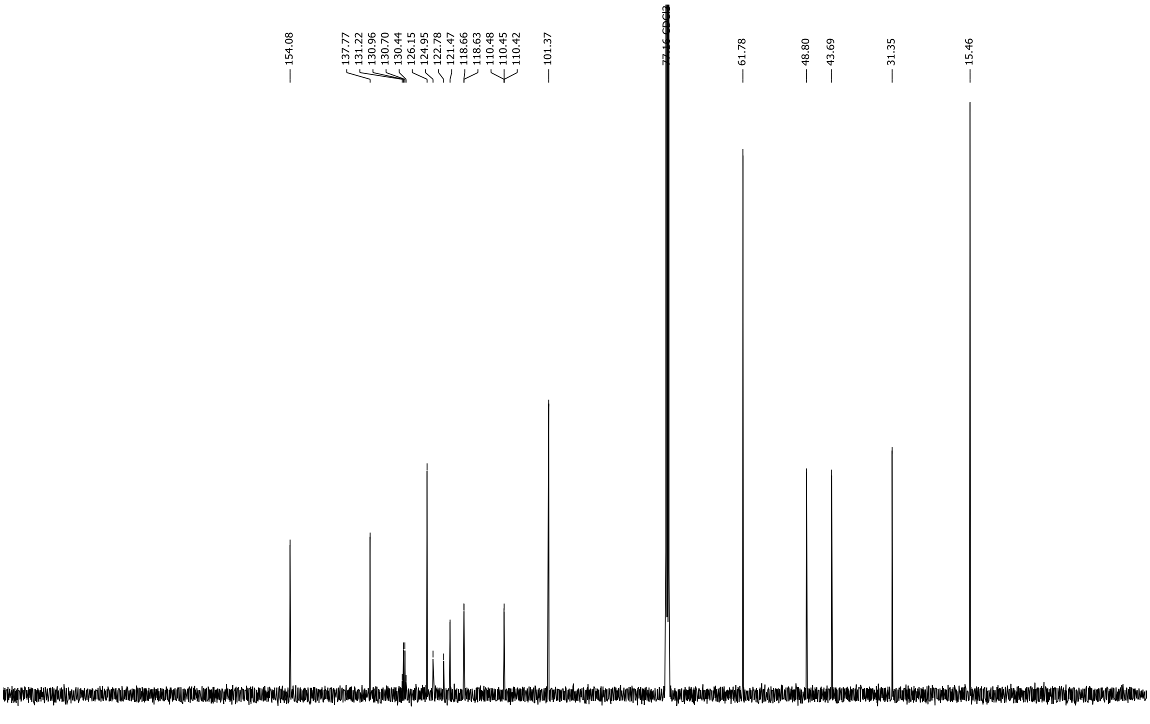

^{19}F NMR (471 MHz, $CDCl_3$):

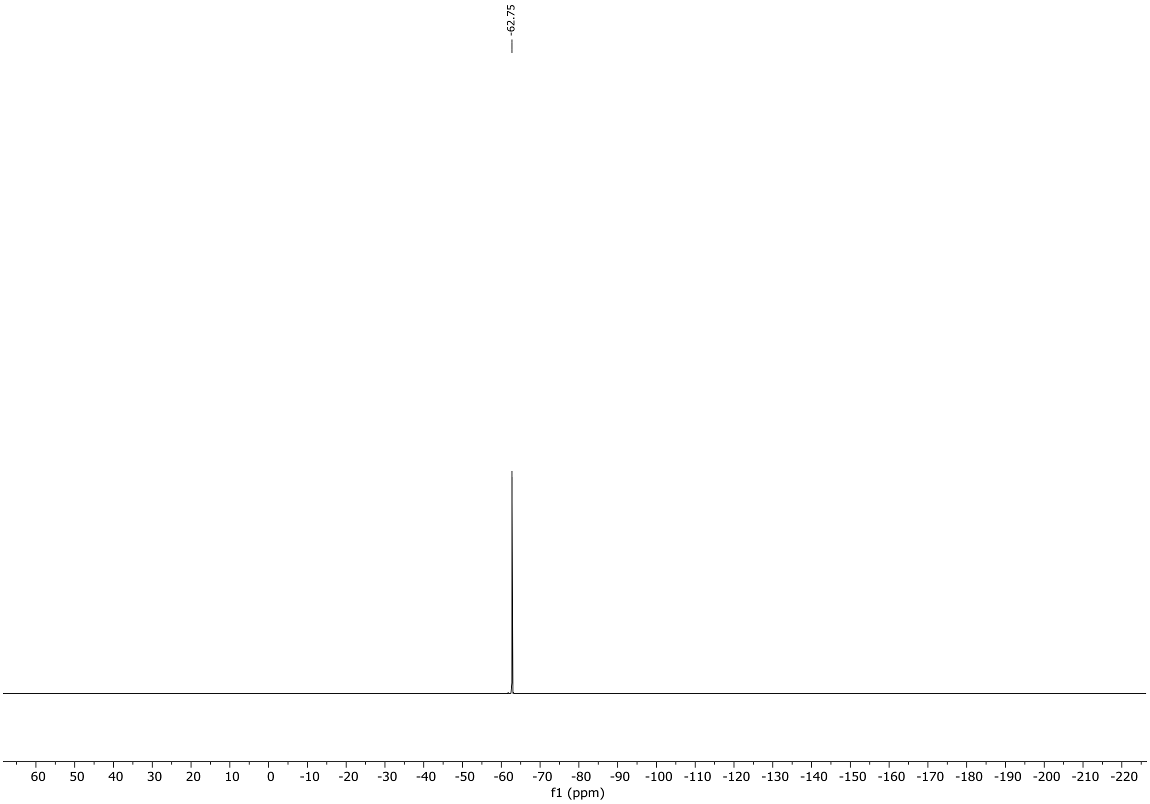

*10.2.9. 3-(3,3-Diethoxypropyl)-6-vinyl-3,4-dihydroquinazolin-2(1H)-one (**SI-8i**)*

^{1}H NMR (500 MHz, CDCl$_3$):

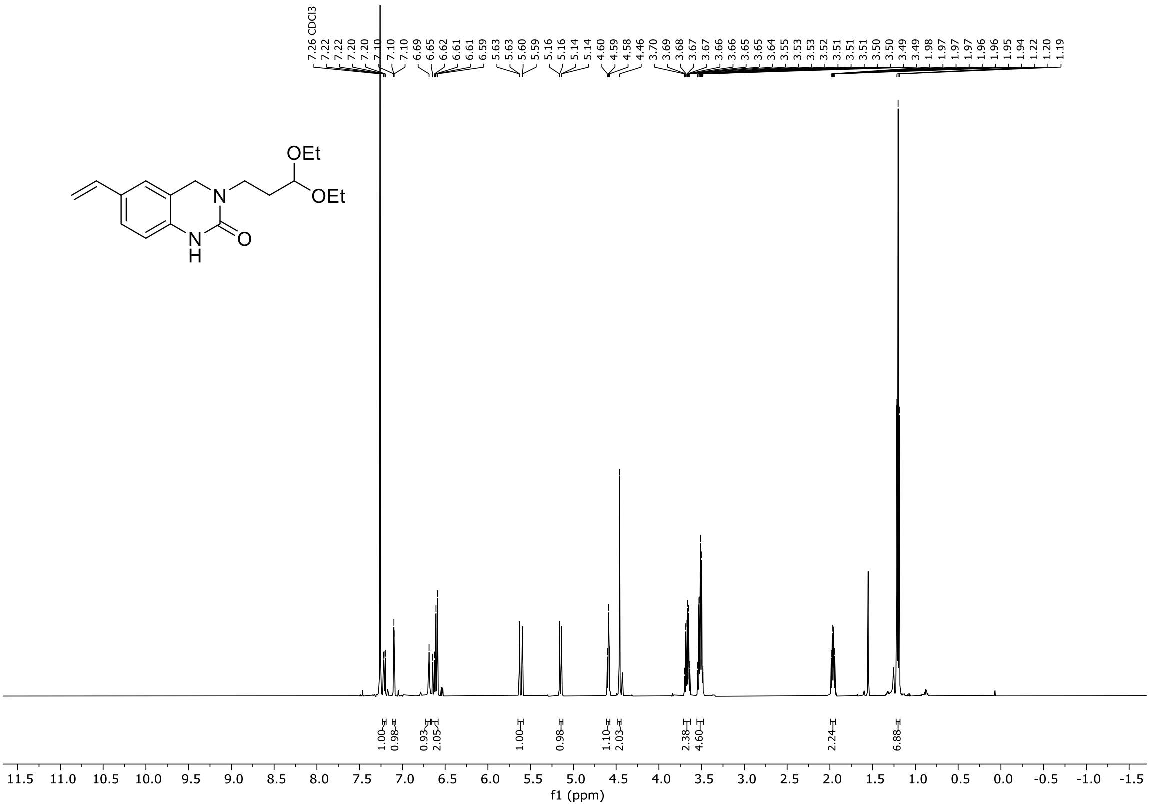

^{13}C NMR (126 MHz, CDCl$_3$):

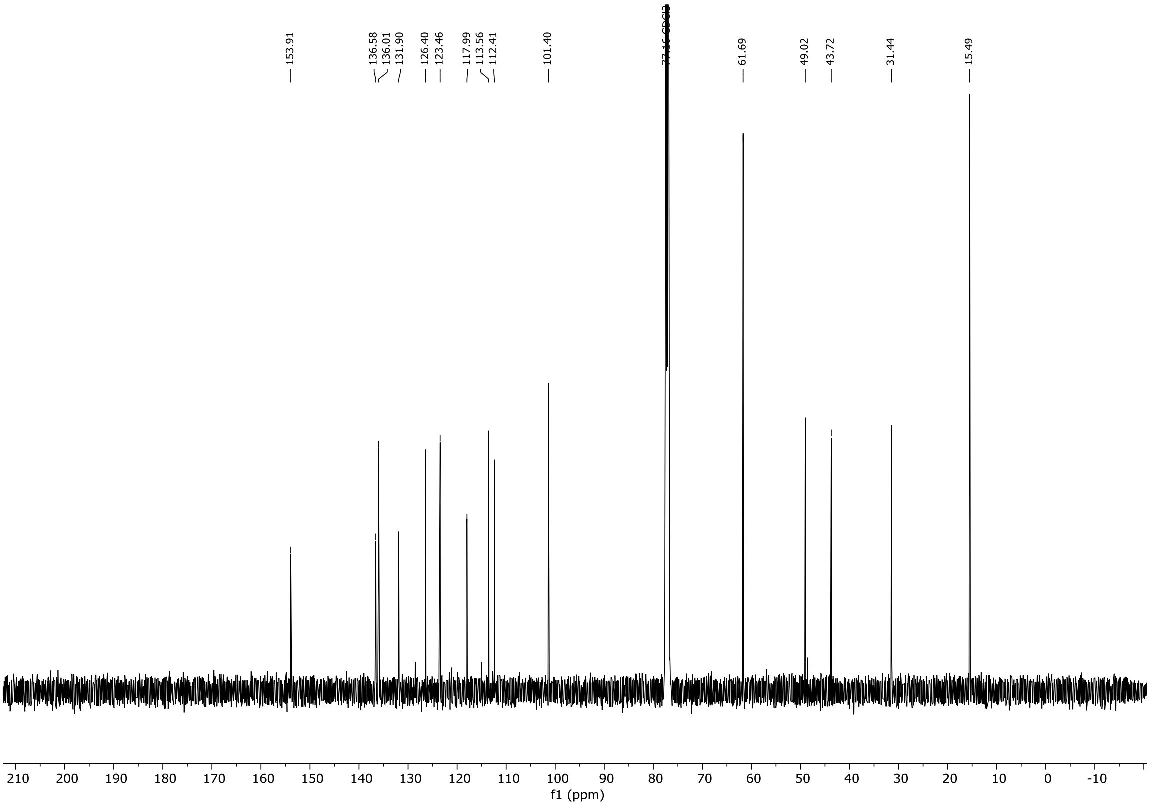

*10.2.10. 6-Allyl-3-(3,3-diethoxypropyl)-3,4-dihydroquinazolin-2(1H)-one (**SI-8j**)*

^{1}H NMR (500 MHz, $CDCl_3$):

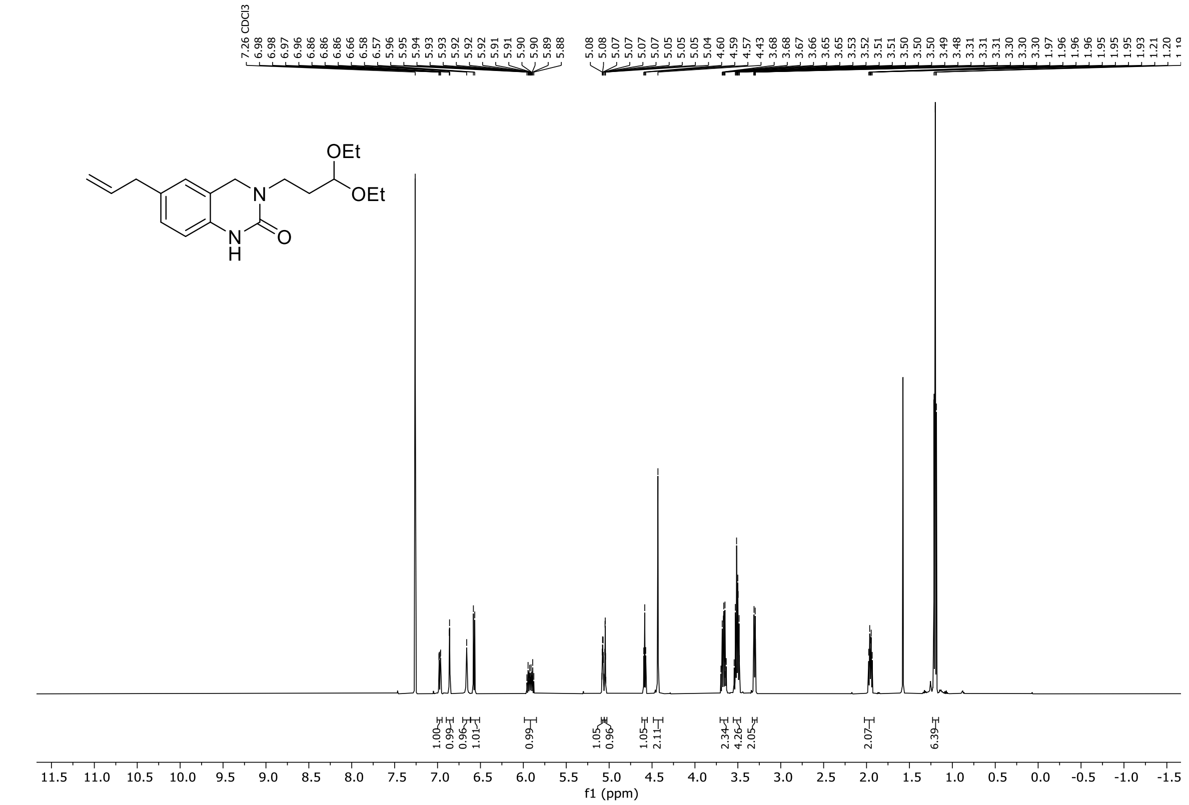

^{13}C NMR (126 MHz, $CDCl_3$):

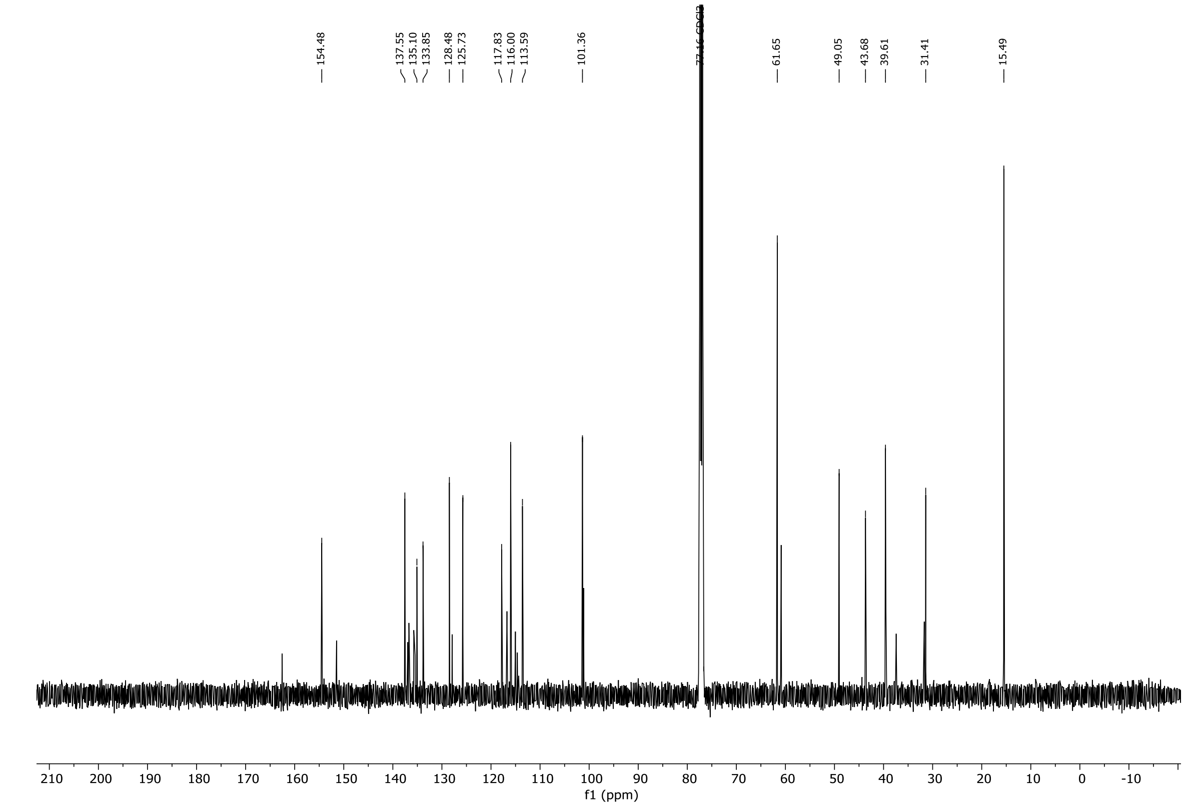

*10.2.11. 3-(3,3-Diethoxypropyl)-8-methyl-3,4-dihydroquinazolin-2(1H)-one (**SI-8k**)*

^{1}H NMR (500 MHz, $CDCl_3$):

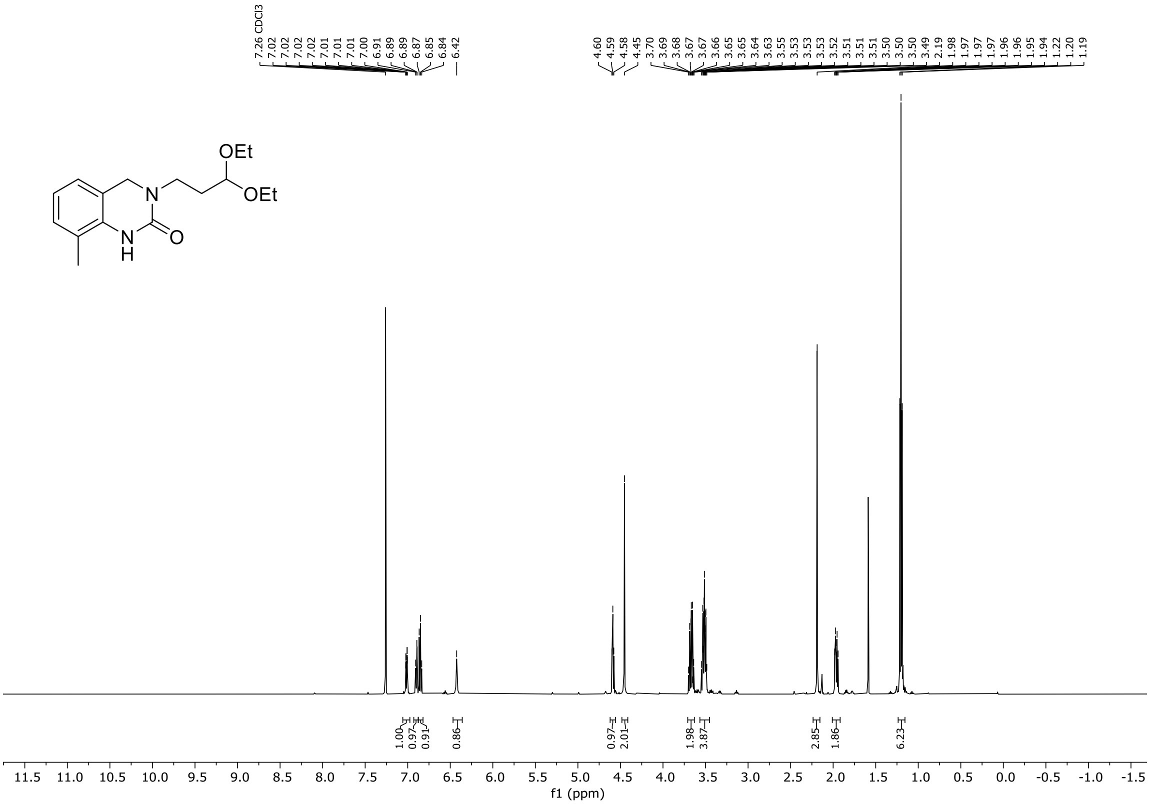

^{13}C NMR (126 MHz, $CDCl_3$):

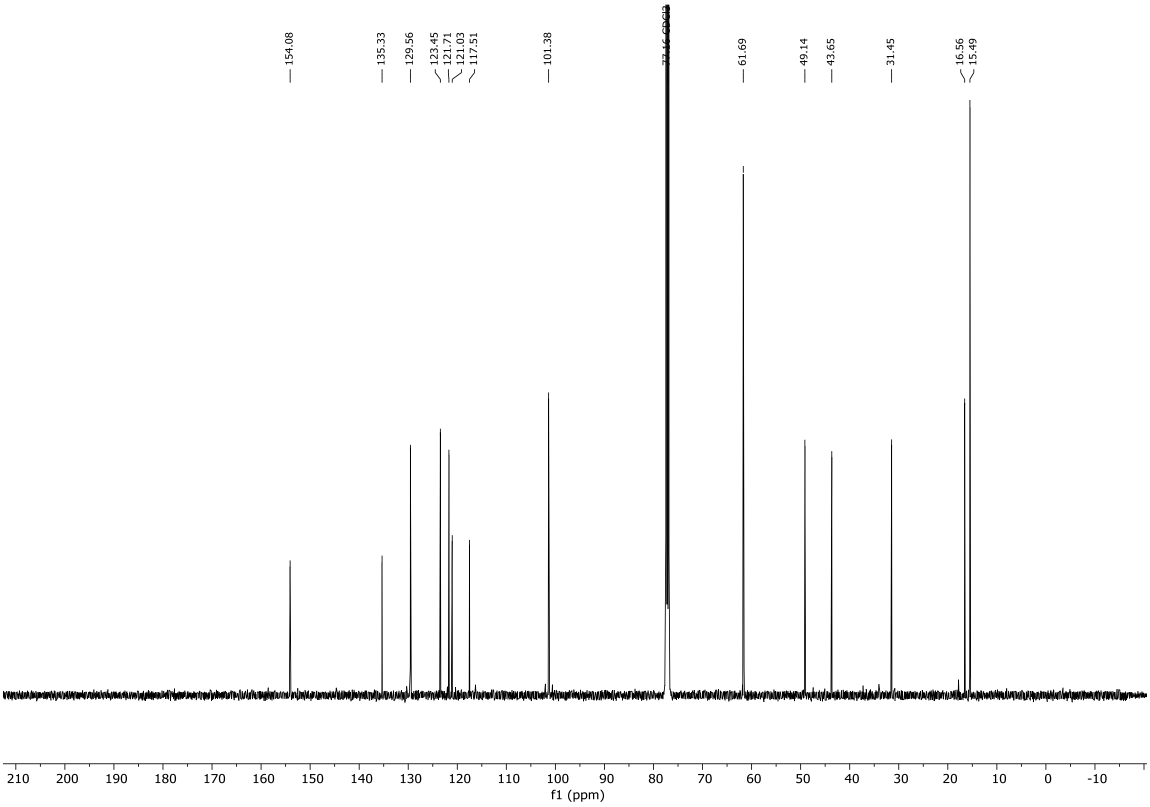

*10.2.12. 3-(3,3-Diethoxypropyl)-6-methoxy-3,4-dihydroquinazolin-2(1H)-one (**SI-8l**)*

^{1}H NMR (500 MHz, $CDCl_3$):

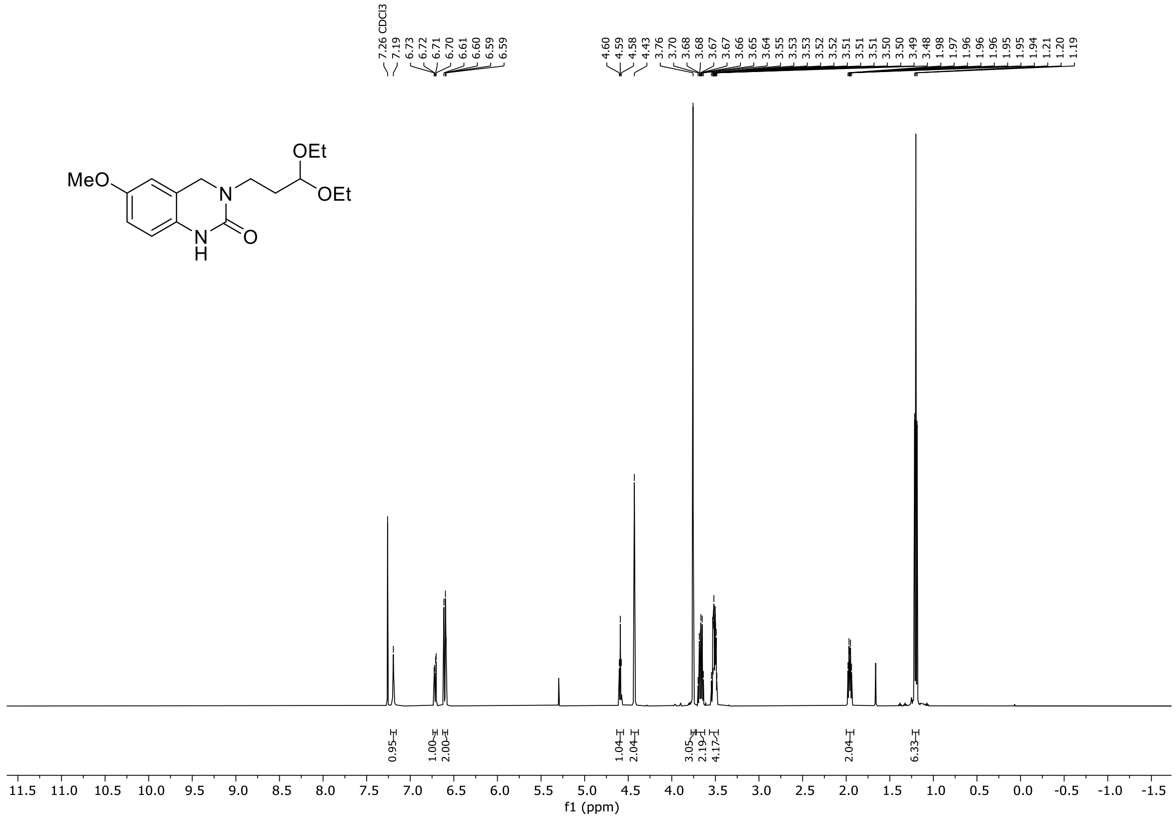

^{13}C NMR (126 MHz, $CDCl_3$):

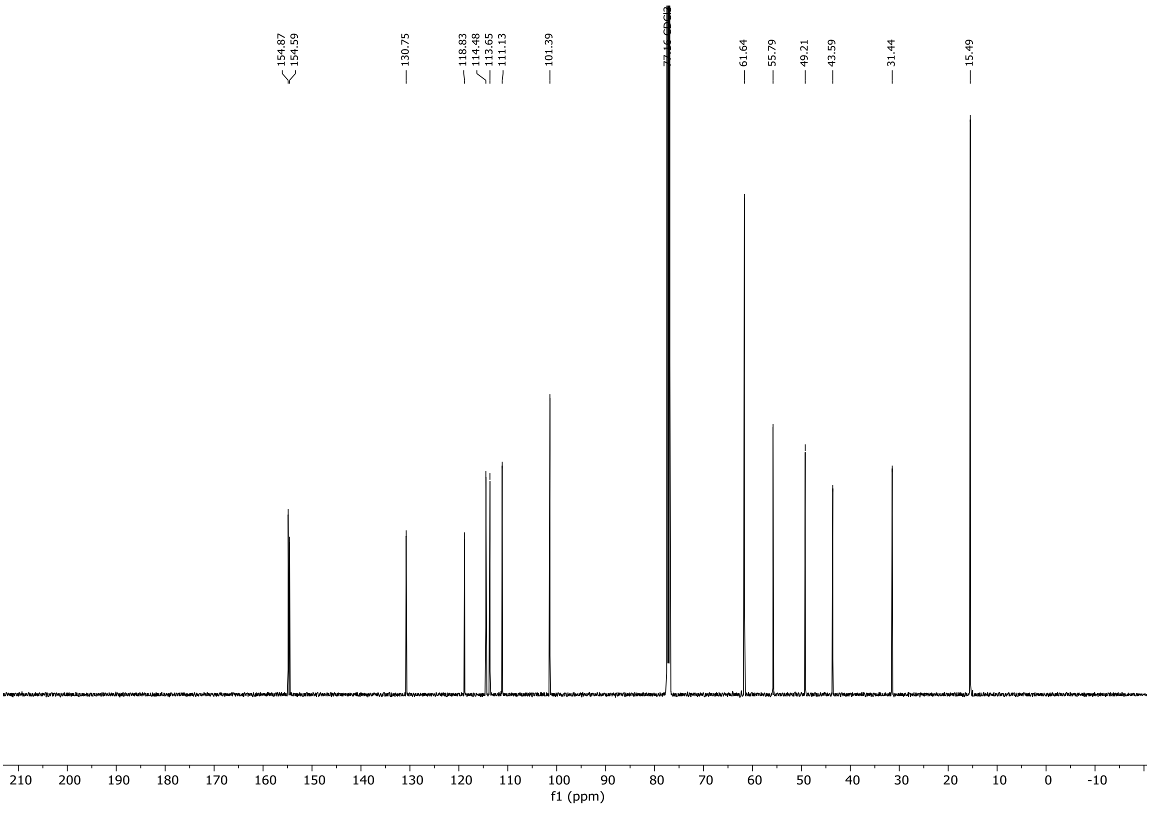

*10.2.13. 3-(3,3-Diethoxypropyl)-8-methoxy-3,4-dihydroquinazolin-2(1H)-one (**SI-8m**)*

^{1}H NMR (500 MHz, $CDCl_3$):

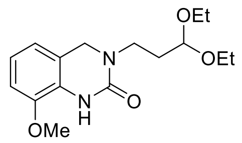

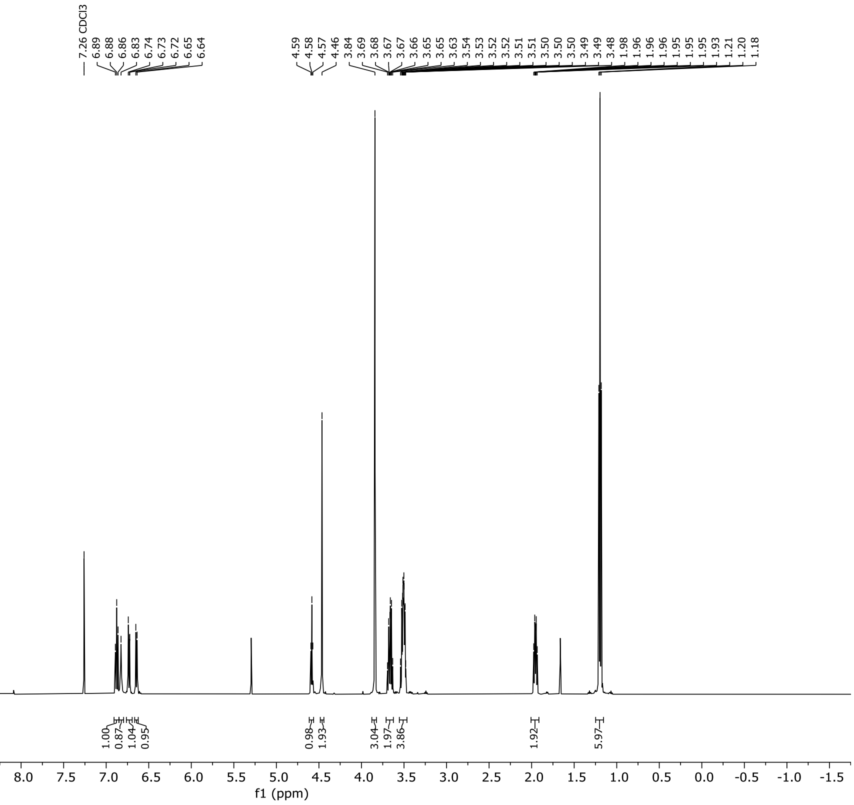

^{13}C NMR (126 MHz, $CDCl_3$):

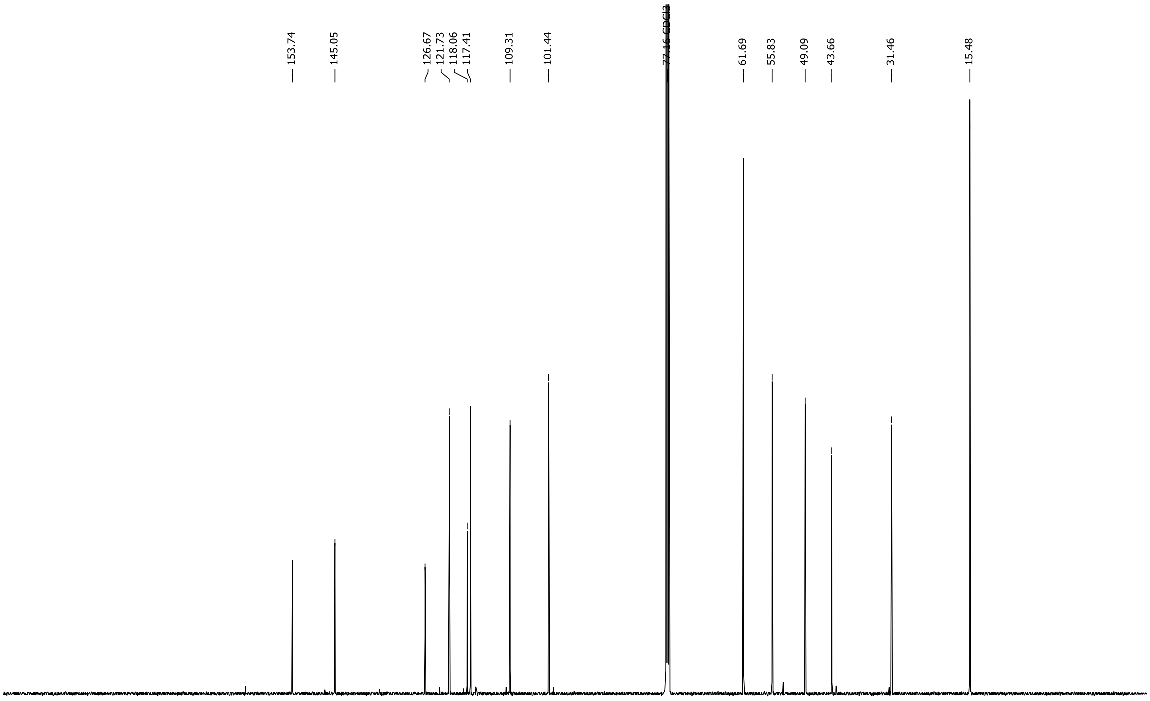

*10.2.14. 3-(3,3-Diethoxypropyl)-7-(furan-3-yl)-3,4-dihydroquinazolin-2(1H)-one (**SI-8n**)*

^{1}H NMR (500 MHz, CD_2Cl_2):

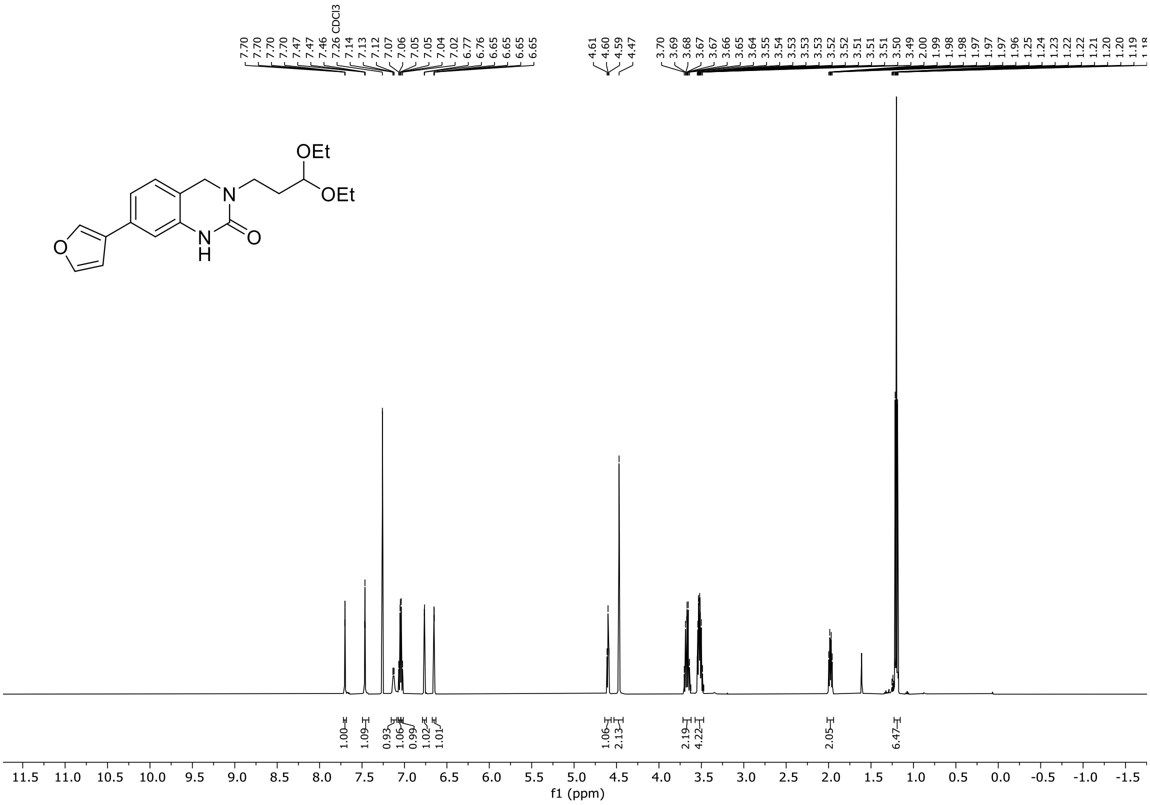

^{13}C NMR (126 MHz, $CDCl_3$):

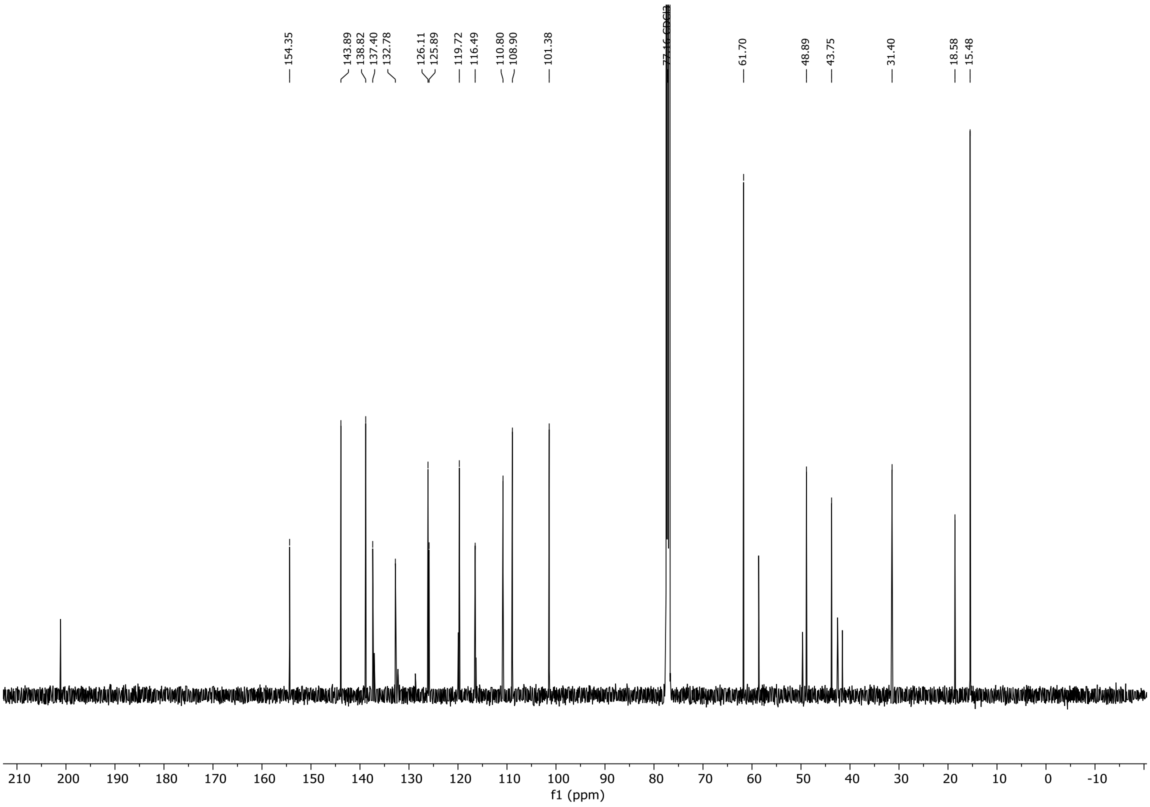

*10.2.15. 3-(3,3-Diethoxypropyl)-7-(thiophen-3-yl)-3,4-dihydroquinazolin-2(1H)-one (**SI-8o**)*

^{1}H NMR (500 MHz, CD_2Cl_2):

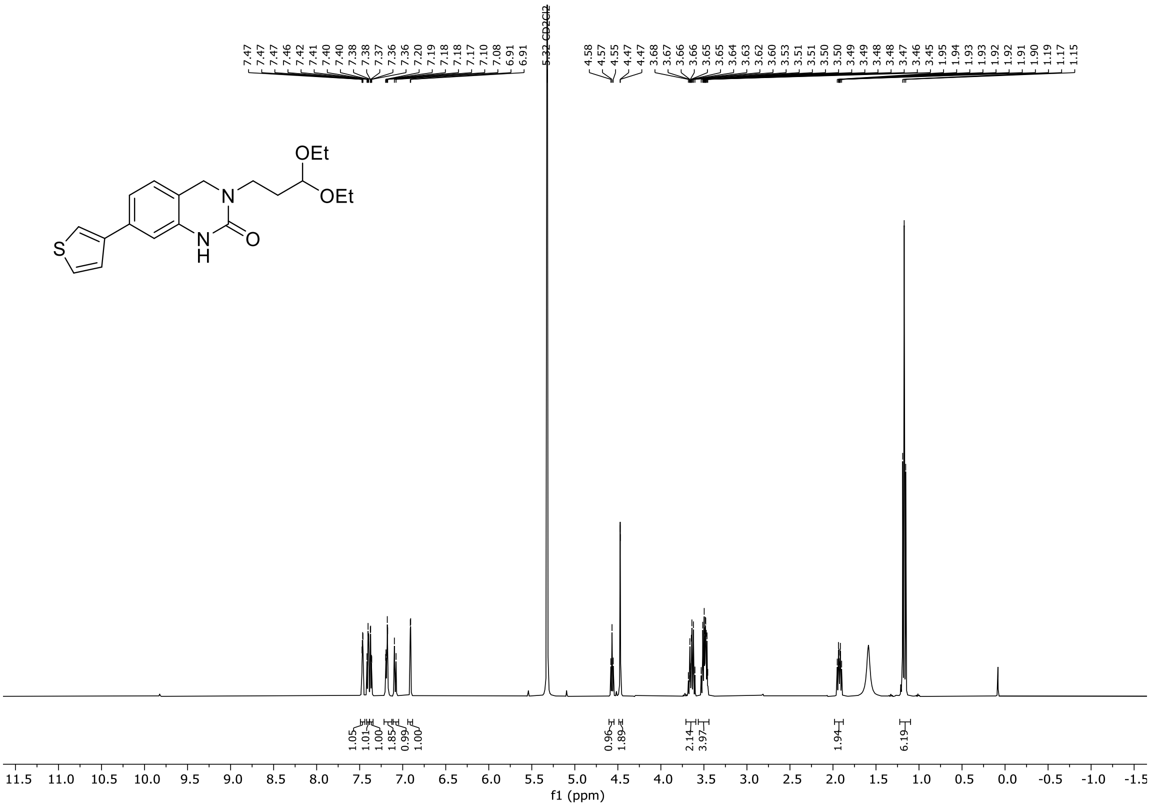

^{13}C NMR (126 MHz, $CDCl_3$):

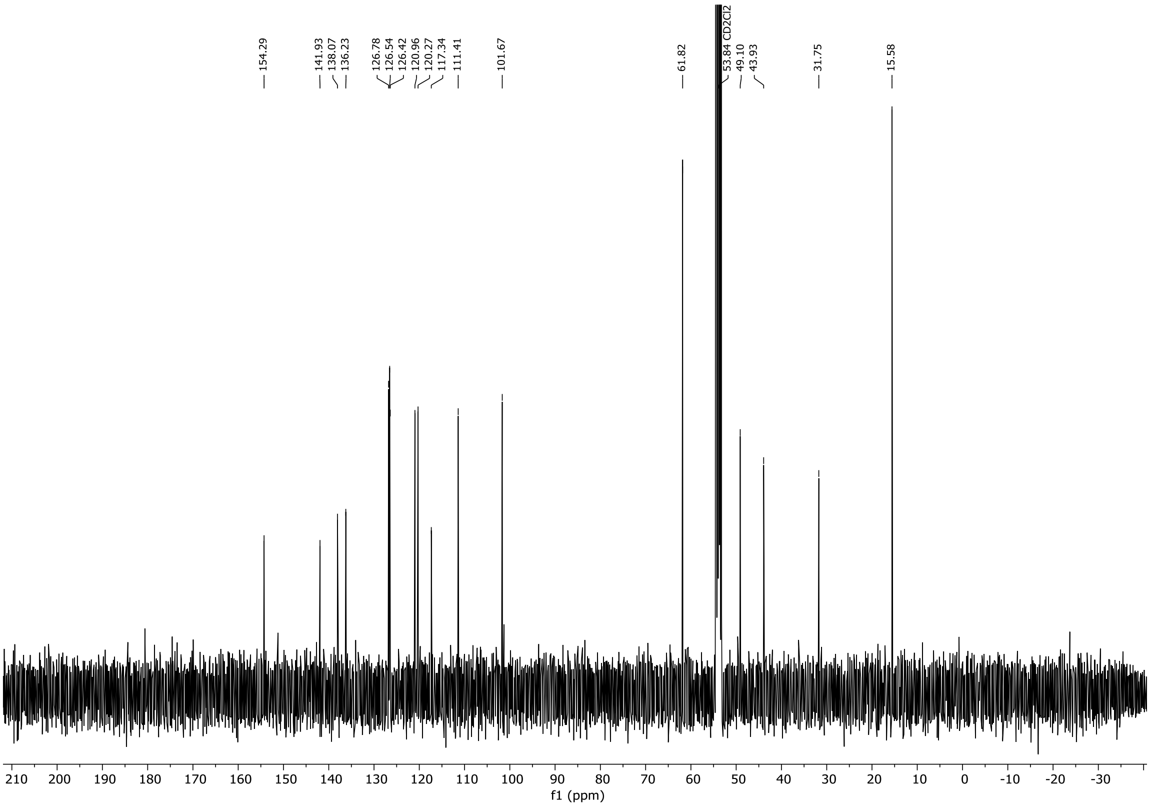

*10.2.16. tert-Butyl 2-(3-(3,3-diethoxypropyl)-2-oxo-1,2,3,4-tetrahydroquinazolin-7-yl)-1H-pyrrole-1-carboxylate **(SI-8p)***

^{1}H NMR (500 MHz, CD_2Cl_2):

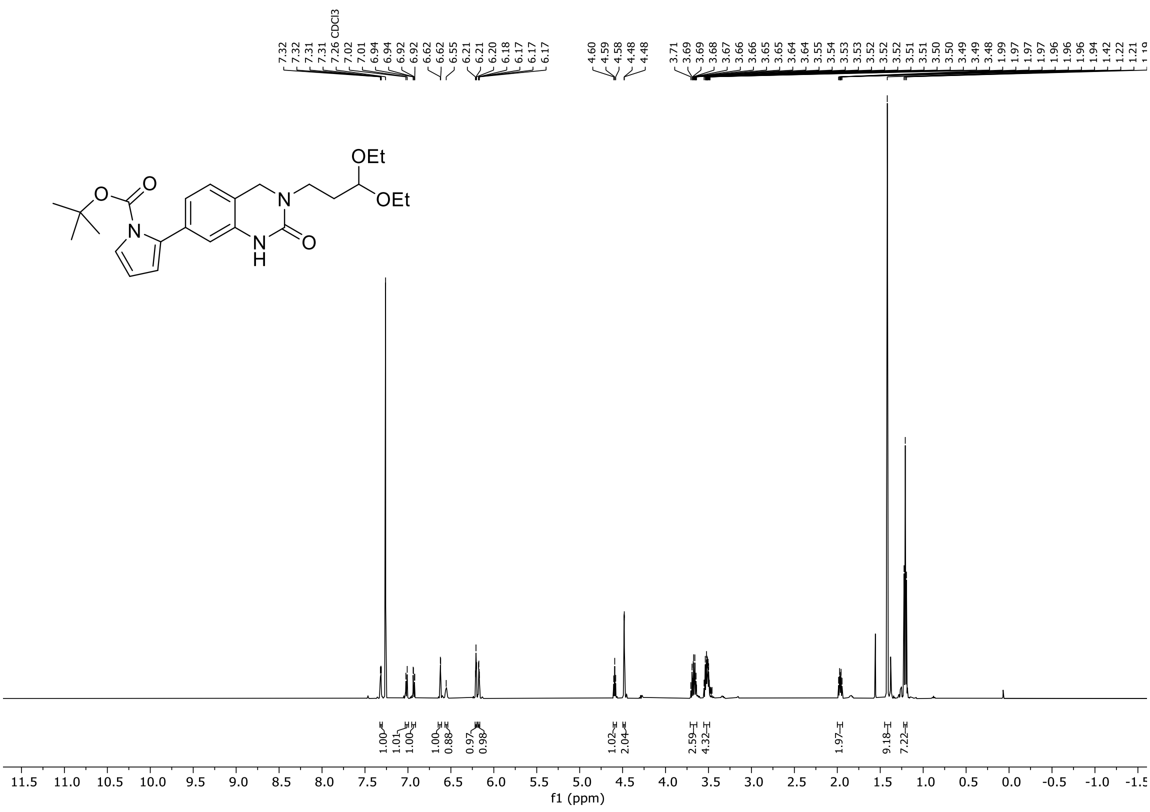

^{13}C NMR (126 MHz, $CDCl_3$):

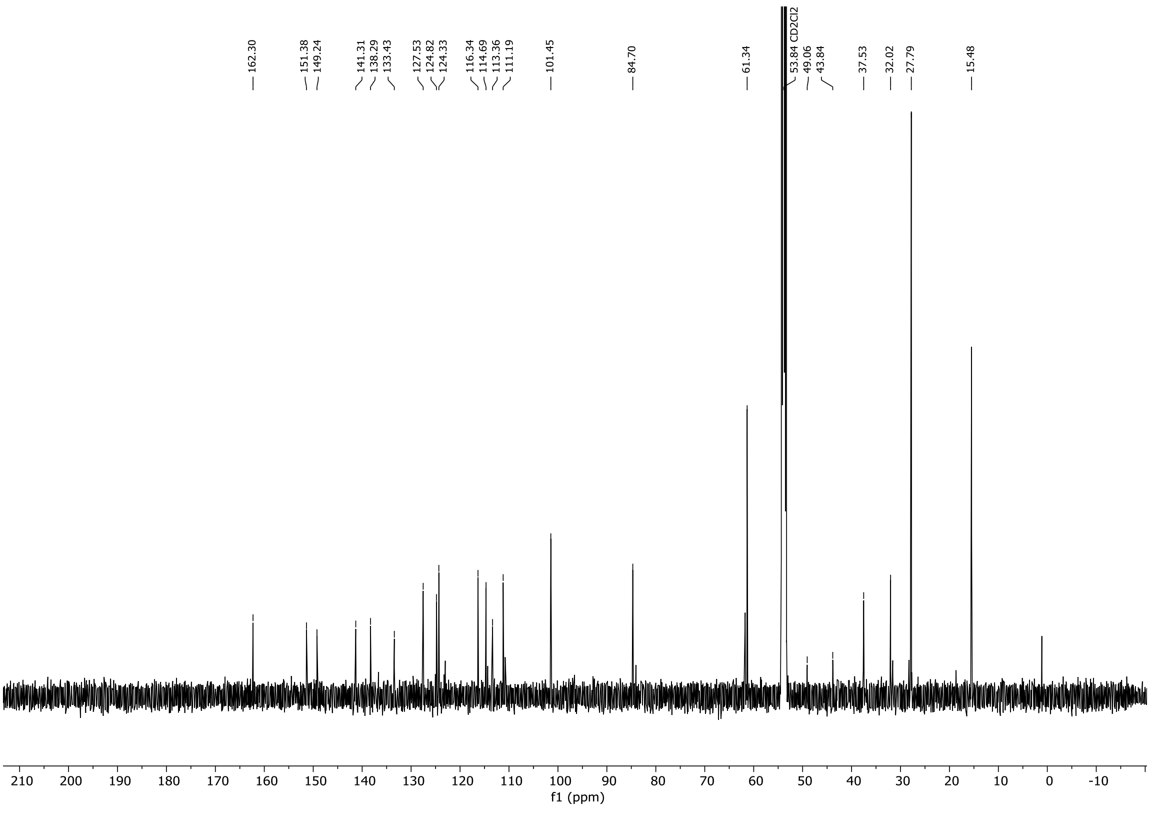

*10.2.17. 3-(3,3-Diethoxypropyl)-7-(pyridin-3-yl)-3,4-dihydroquinazolin-2(1H)-one (**SI-8q**)*

^{1}H NMR (500 MHz, $CDCl_3$):

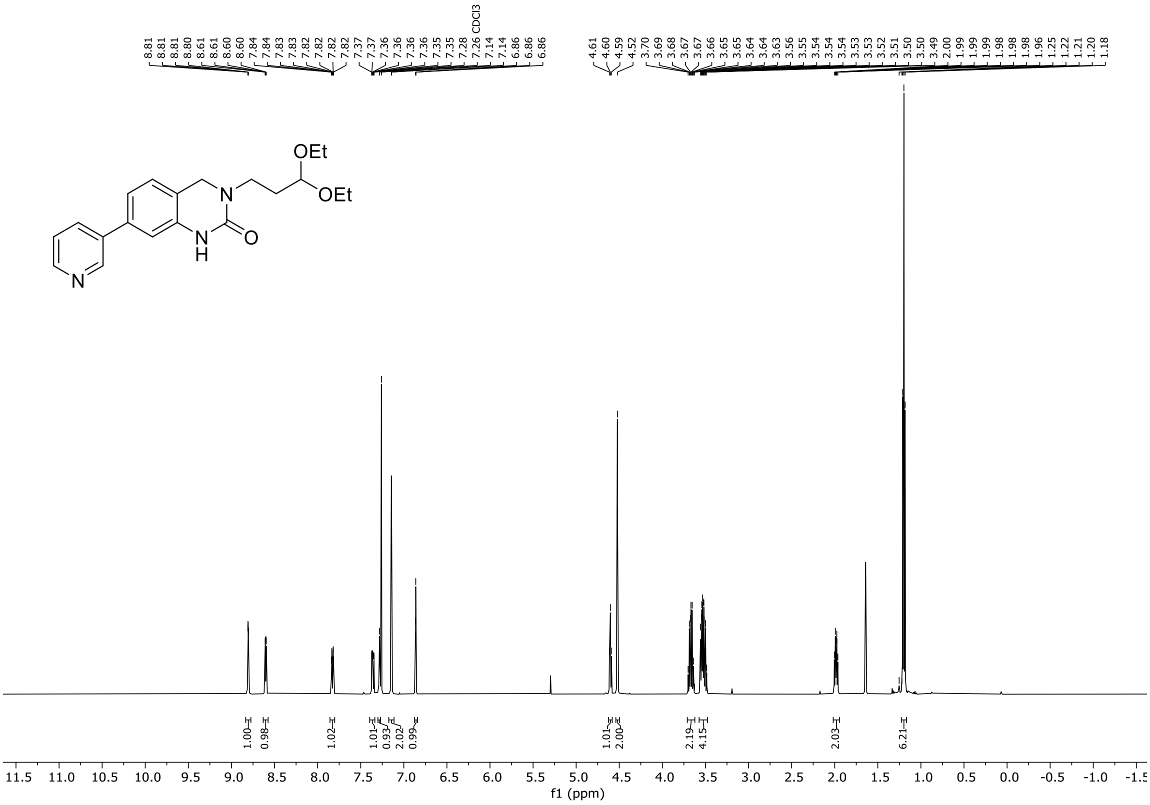

^{13}C NMR (126 MHz, $CDCl_3$):

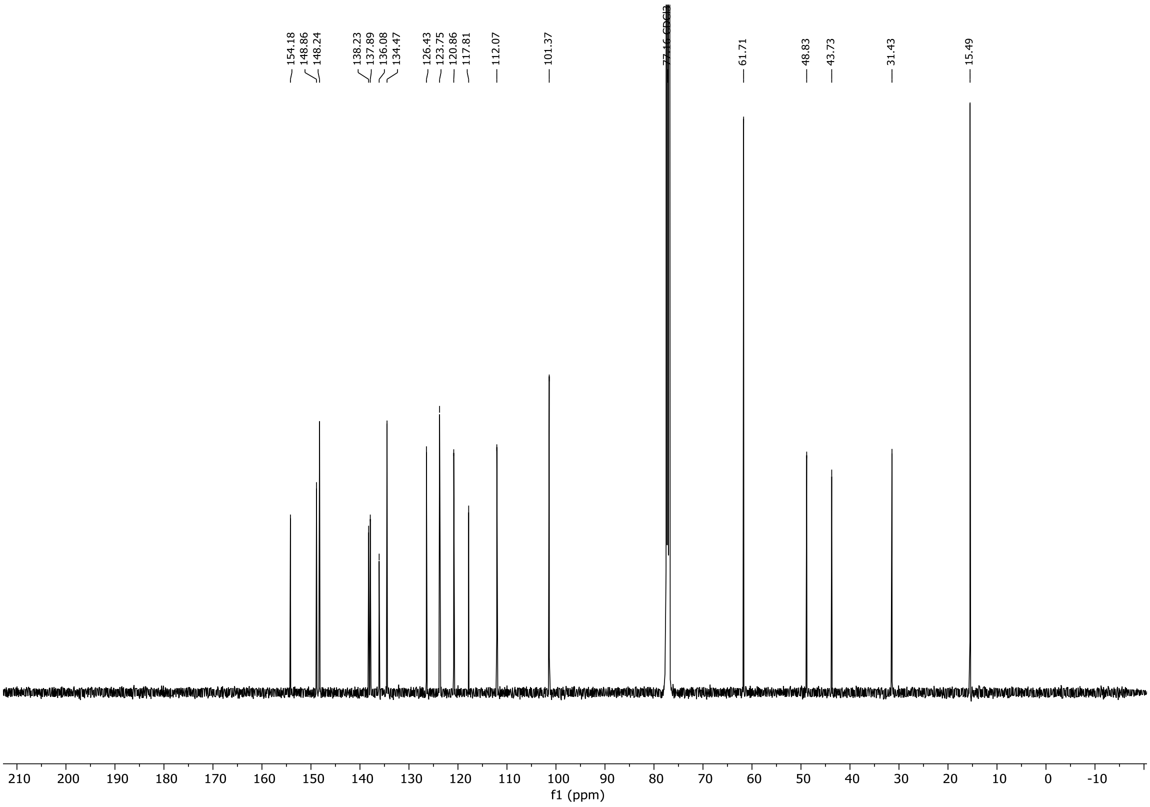

*10.2.18. Ethyl (E)-3-(3-(3,3-diethoxypropyl)-2-oxo-1,2,3,4-tetrahydroquinazolin-7-yl)acrylate (**SI-8r**)*

^{1}H NMR (500 MHz, $CDCl_3$):

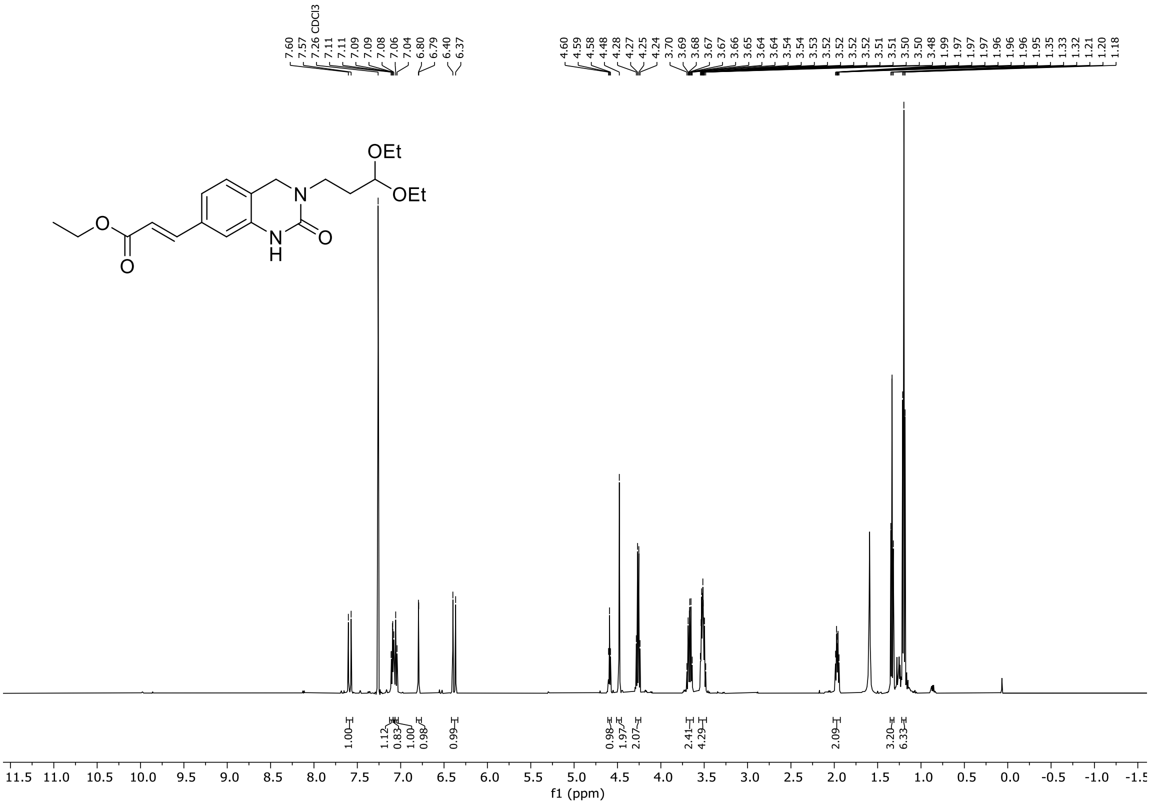

^{13}C NMR (126 MHz, $CDCl_3$):

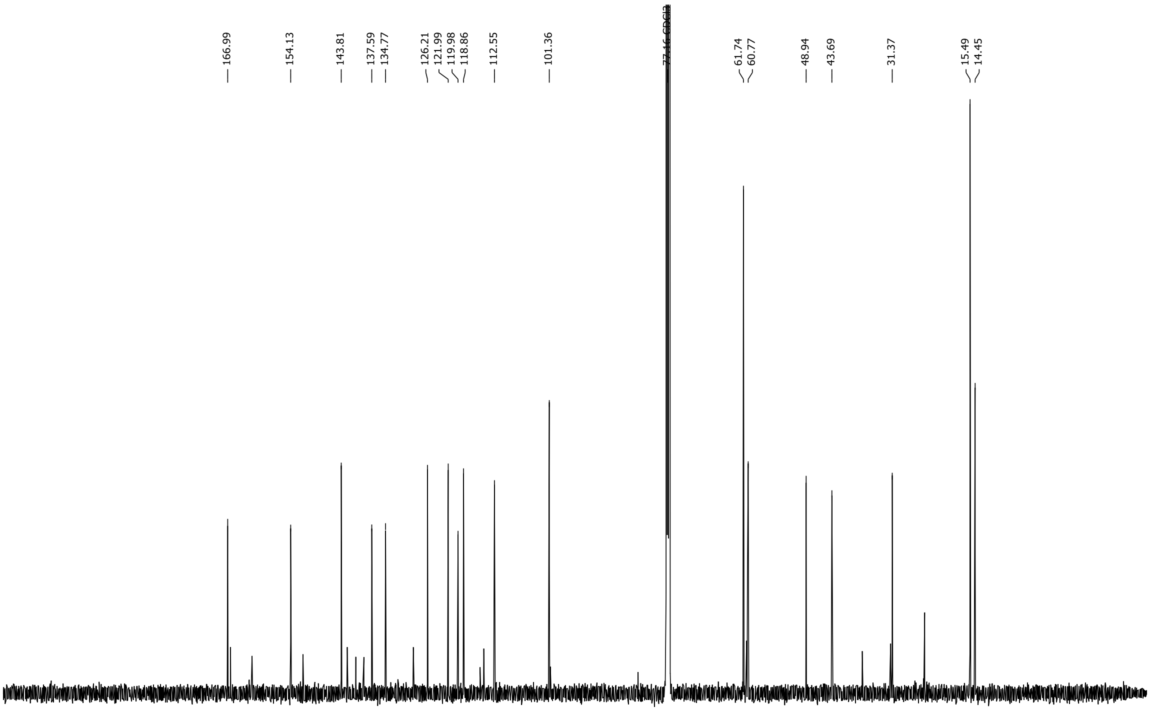

*10.2.19. 3-(3,3-Diethoxypropyl)-6-(4-methoxyphenyl)-3,4-dihydroquinazolin-2(1H)-one (**SI-8s**)*

^{1}H NMR (500 MHz, $CDCl_3$):

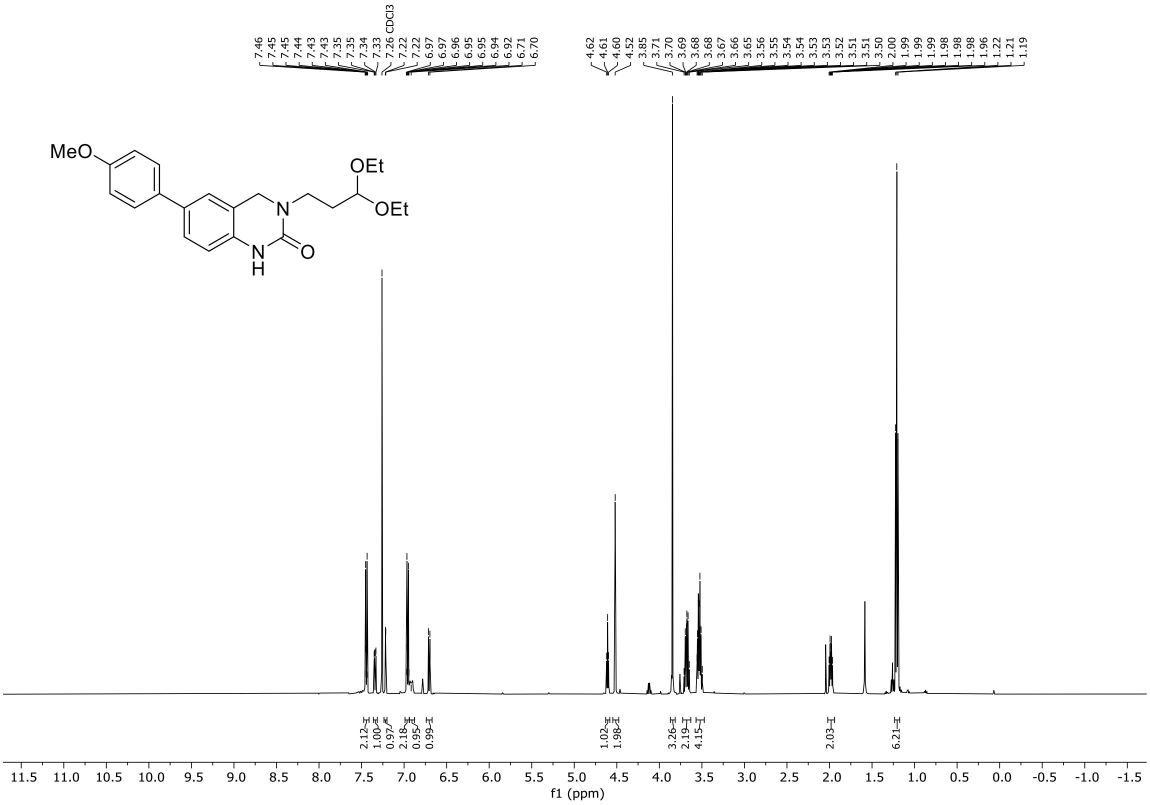

^{13}C NMR (126 MHz, $CDCl_3$):

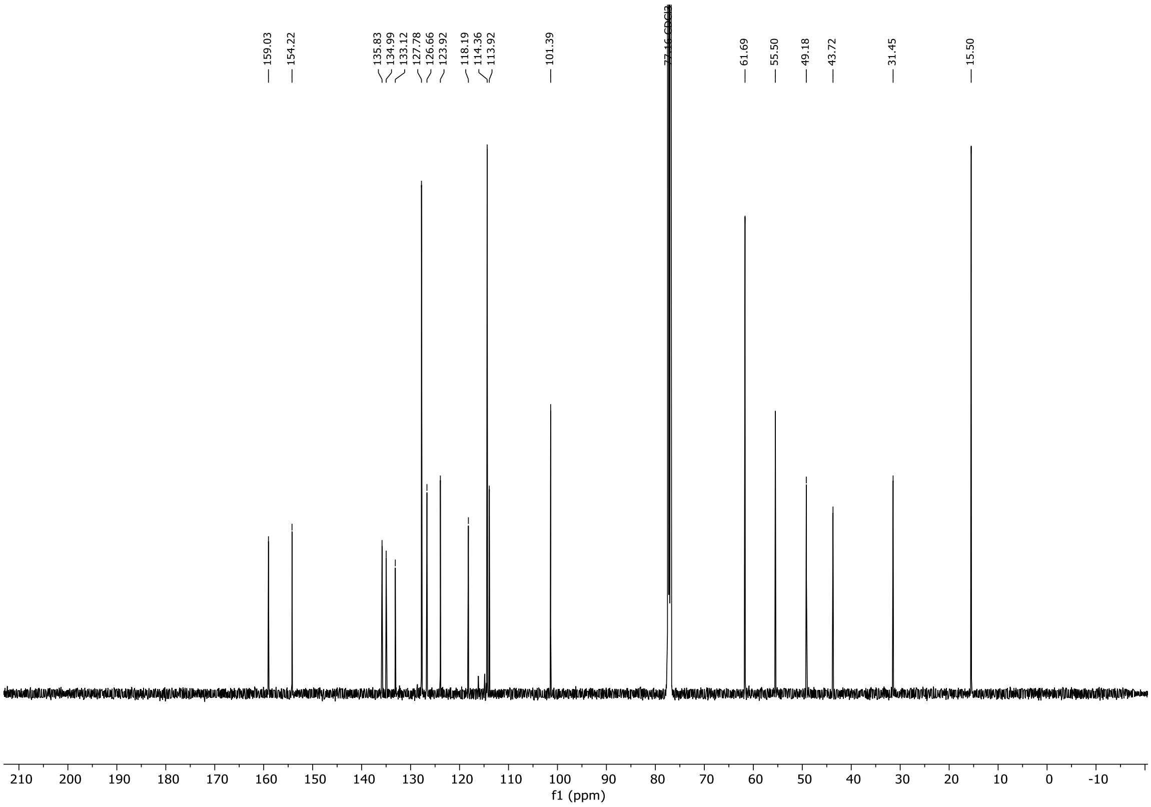

*10.2.20. 3-(3,3-Diethoxypropyl)-3,4-dihydropyrido[2,3-d]pyrimidin-2(1H)-one (**SI-8t**)*

^{1}H NMR (500 MHz, $CDCl_3$):

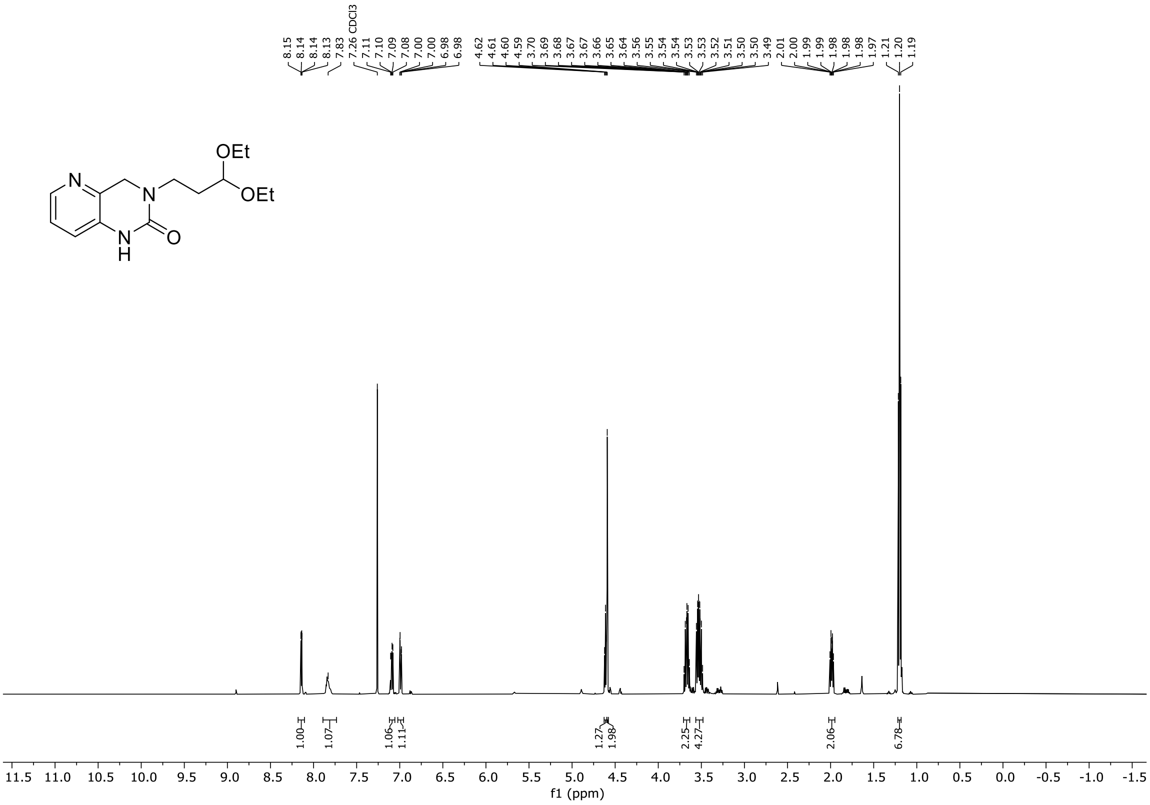

^{13}C NMR (126 MHz, $CDCl_3$):

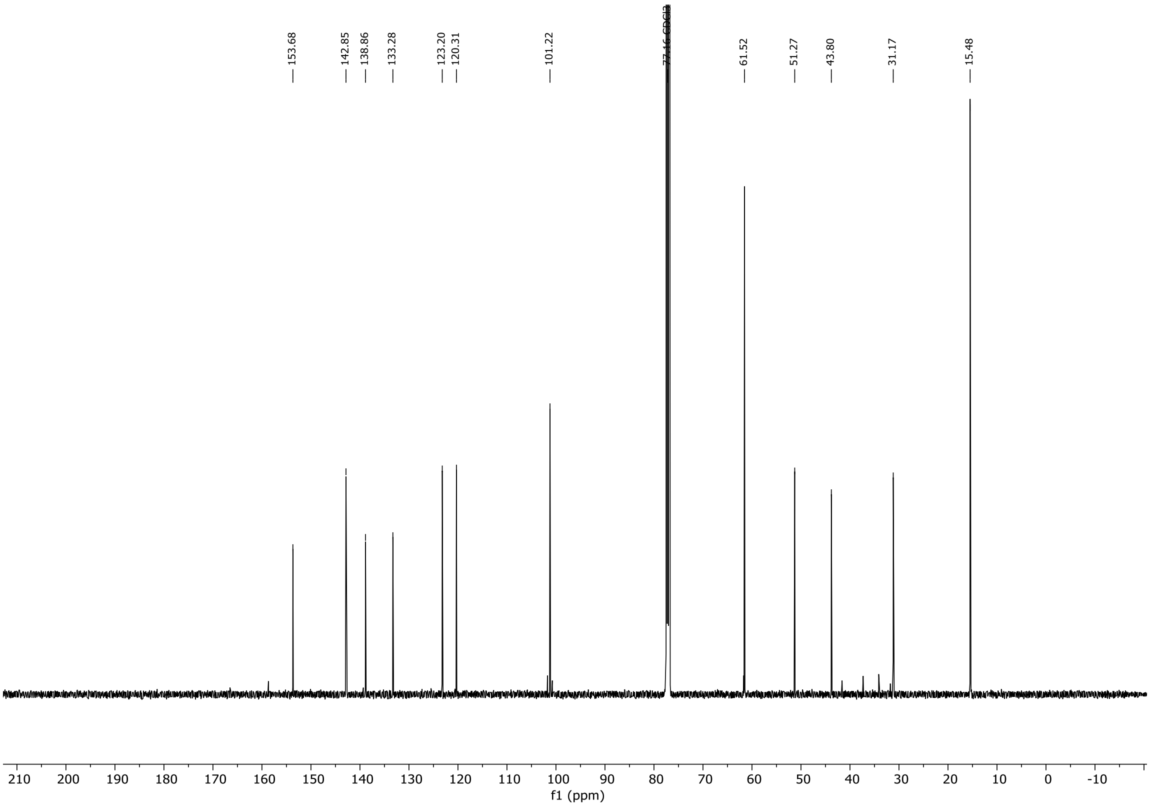

10.2.21. 3-(3-Hydroxy-2,2-dimethylpropyl)-3,4-dihydroquinazolin-2(1H)-one ***(SI-8u)***

^{1}H NMR (500 MHz, $CDCl_3$):

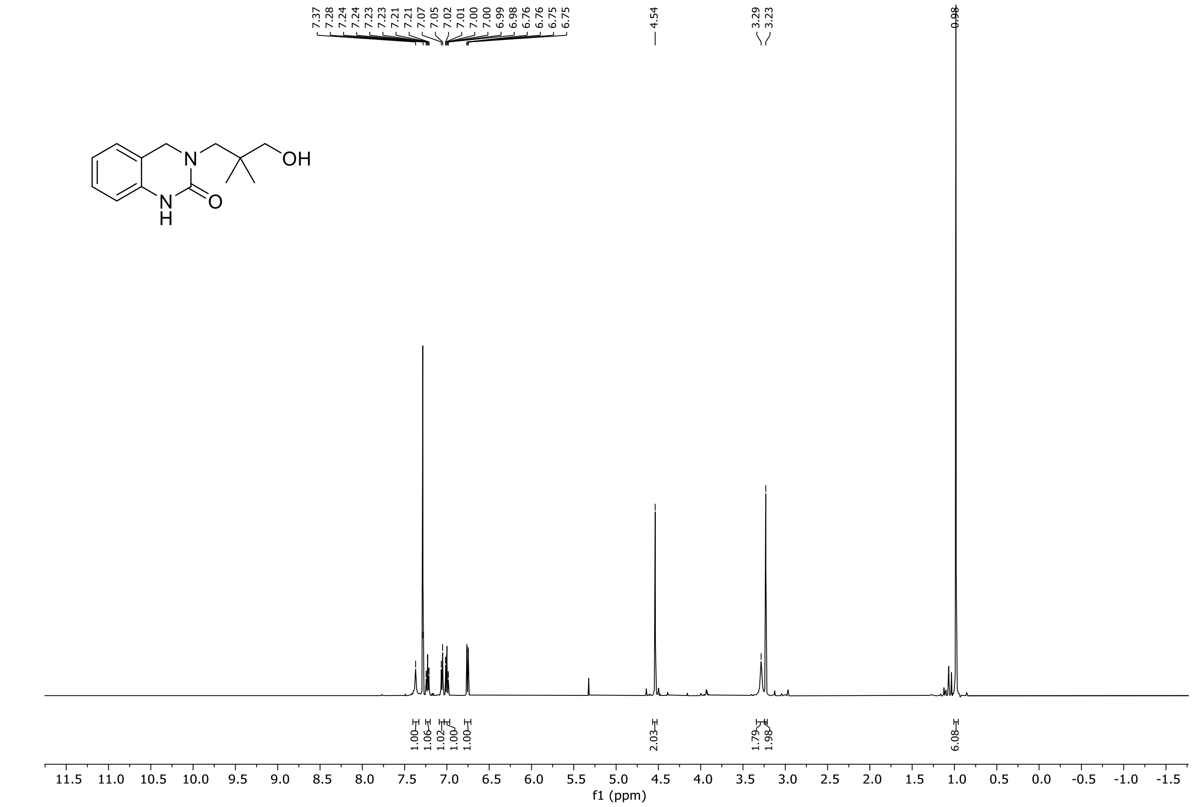

^{13}C NMR (126 MHz, $CDCl_3$):

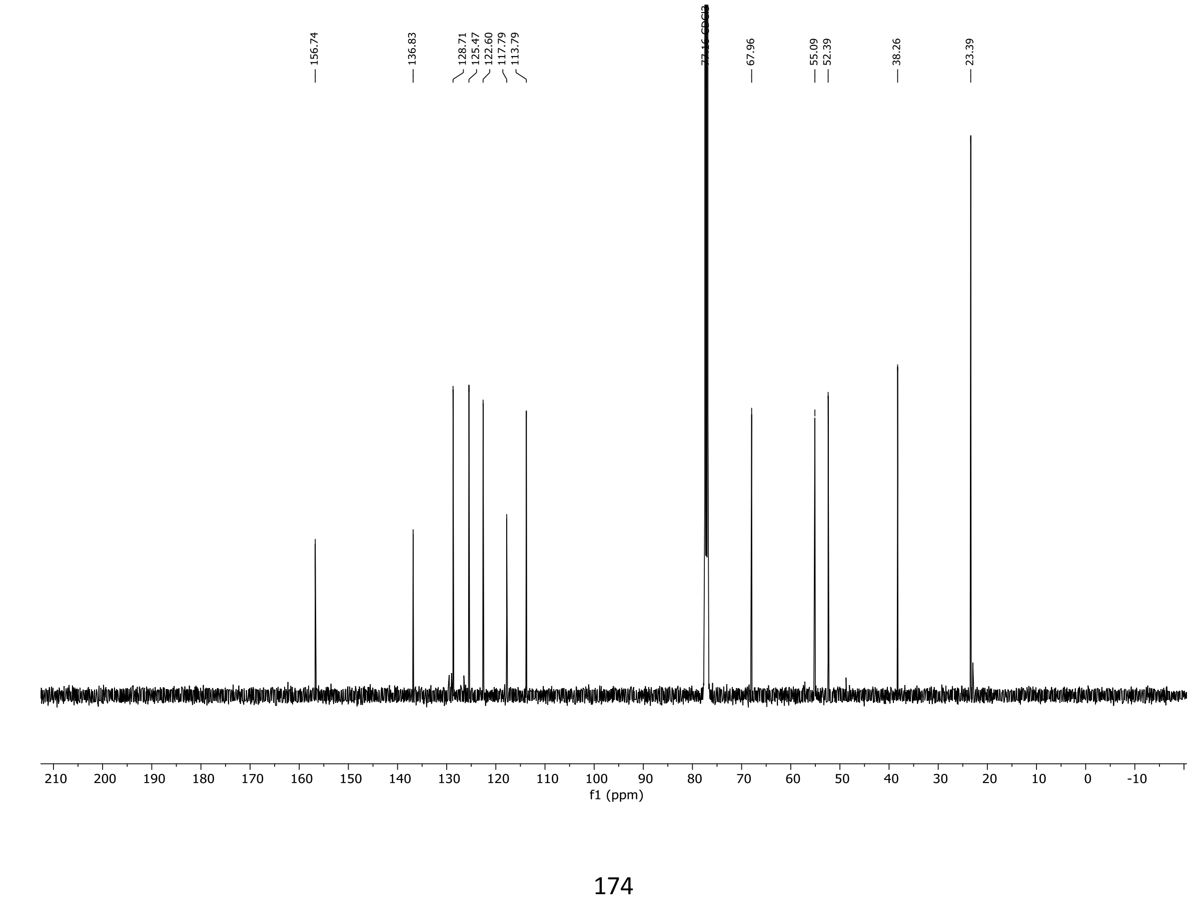

*10.2.22. 3-(3,3-Diethoxypropyl)-3,4-dihydropyrido[2,3-d]pyrimidin-2(1H)-one (**SI-8v**)*

^{1}H NMR (500 MHz, $CDCl_3$):

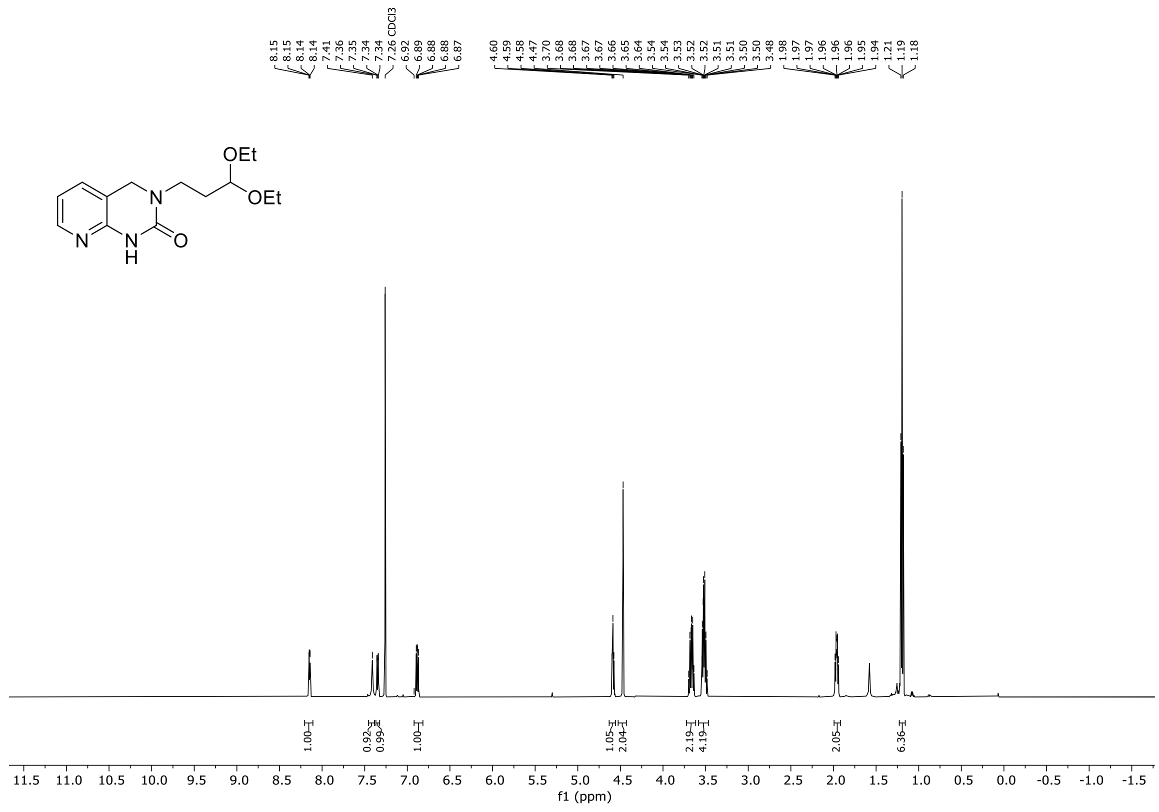

^{13}C NMR (126 MHz, $CDCl_3$):

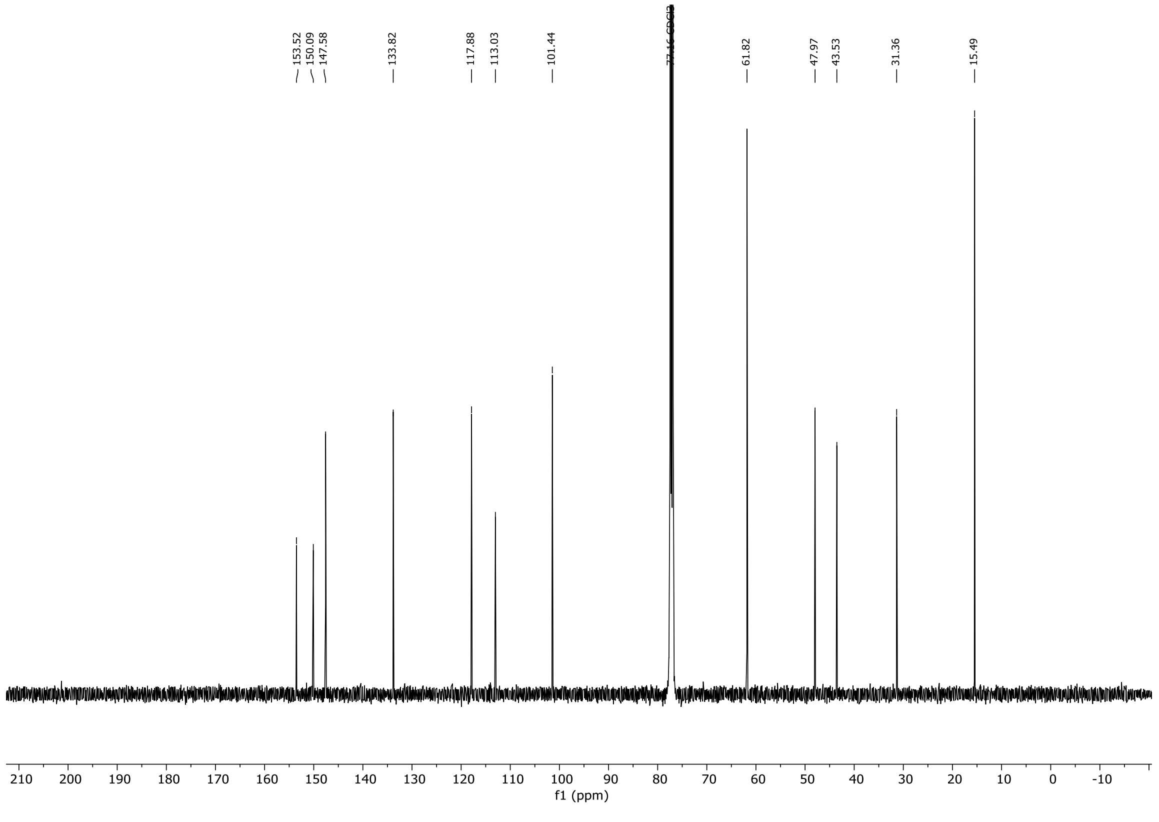

*10.2.23. 2-(2-Oxo-1,4-dihydroquinazolin-3(2H)-yl)benzonitrile (**SI-8w**)*

^{1}H NMR (500 MHz, $CDCl_3$):

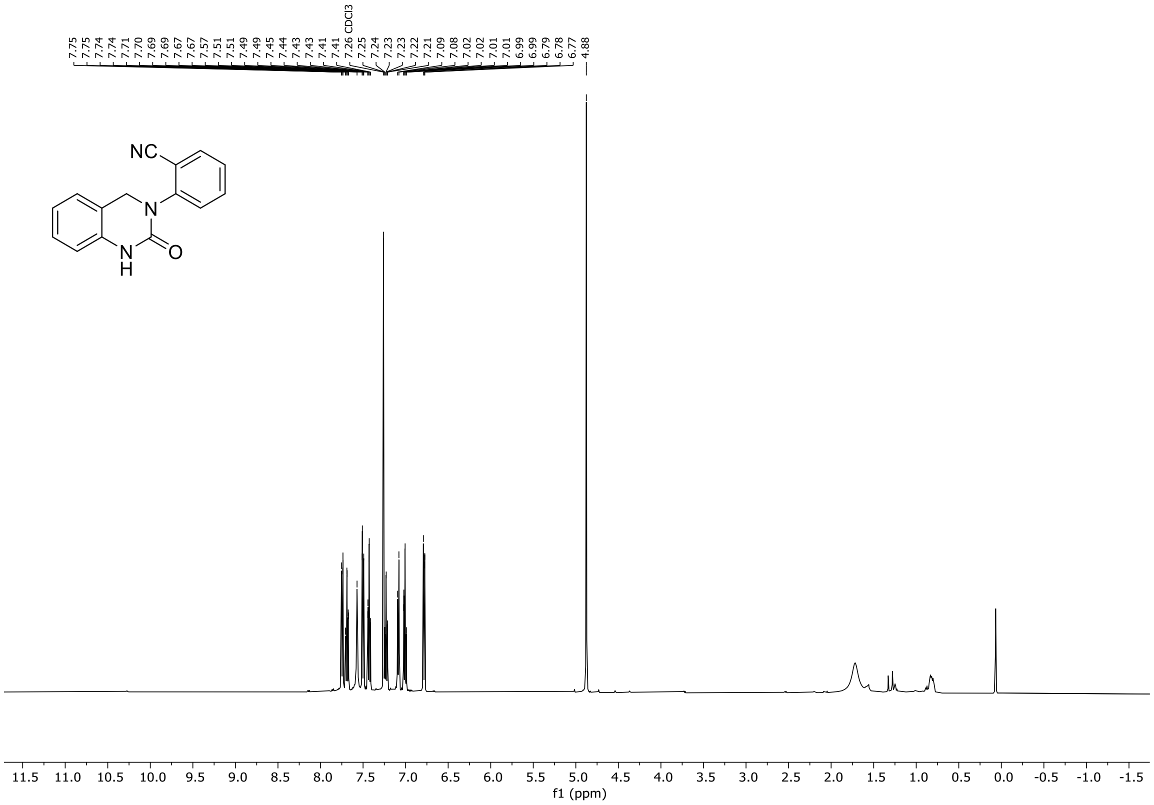

^{13}C NMR (126 MHz, $CDCl_3$):

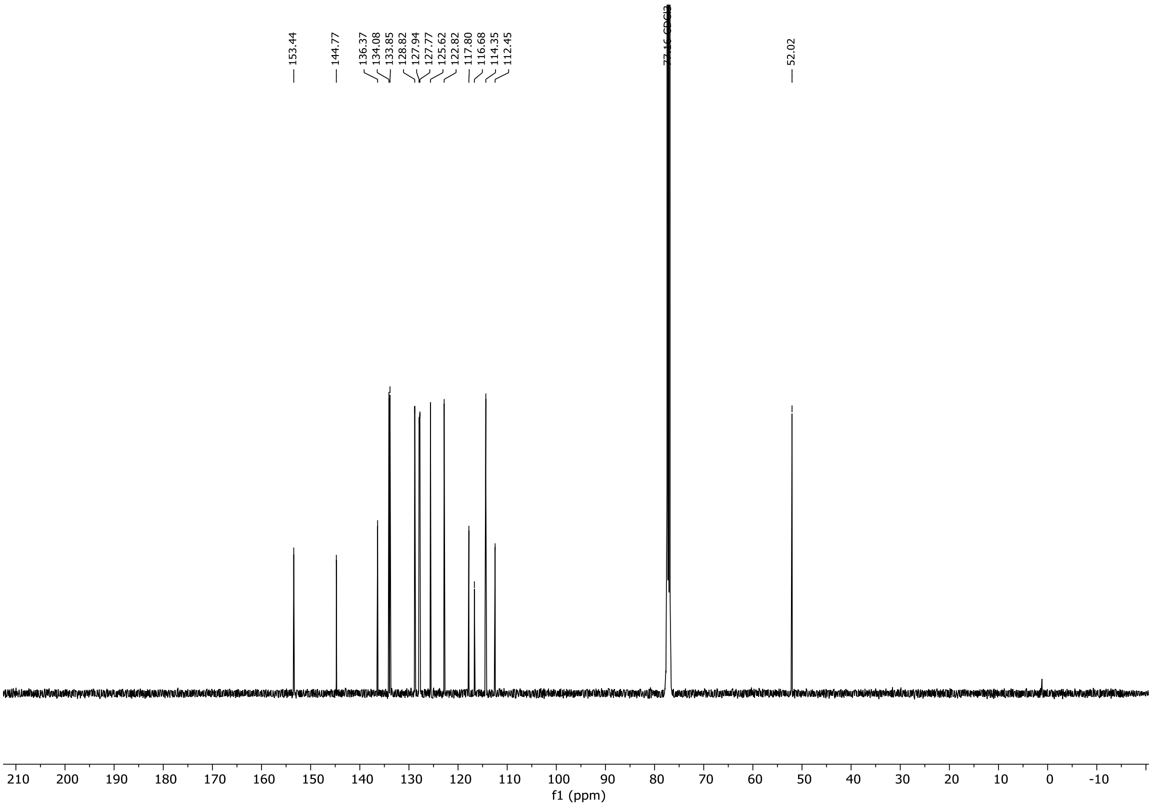

*10.2.24. 3-(4,4-Diethoxybutyl)-3,4-dihydroquinazolin-2(1H)-one (**SI-8aa**)*

^{1}H NMR (500 MHz, $CDCl_3$):

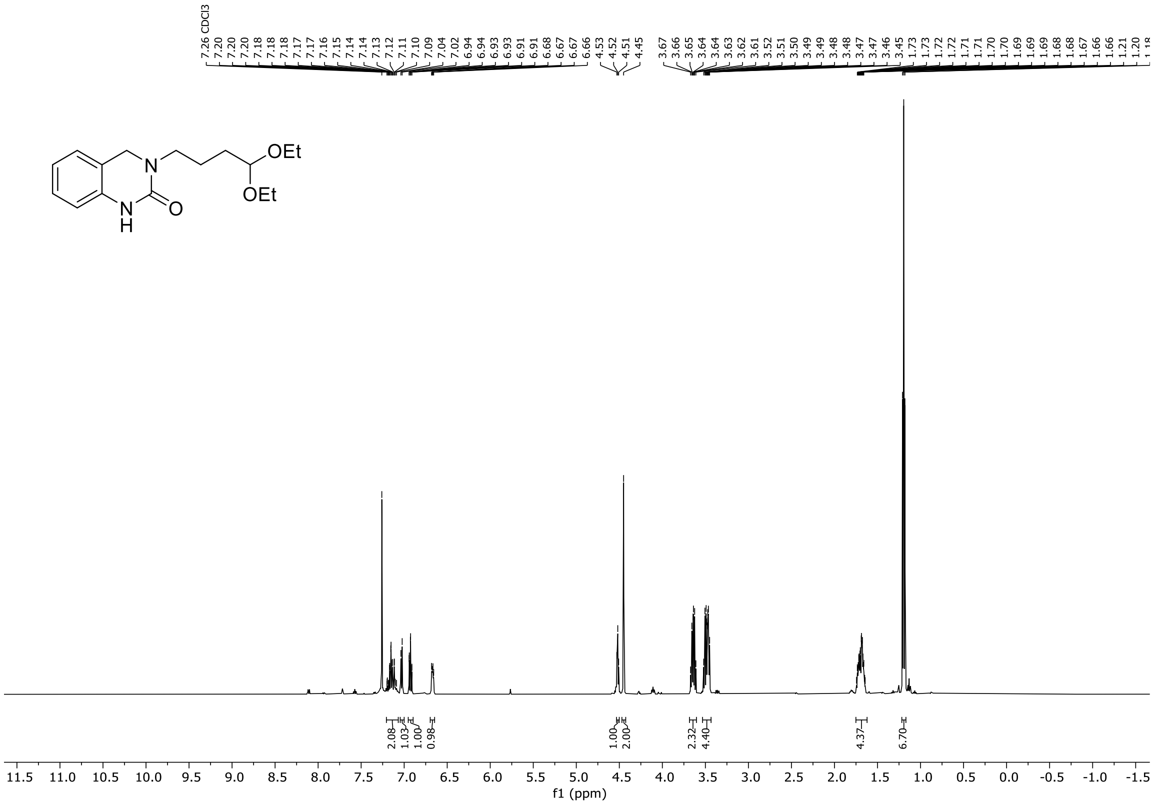

^{13}C NMR (126 MHz, $CDCl_3$):

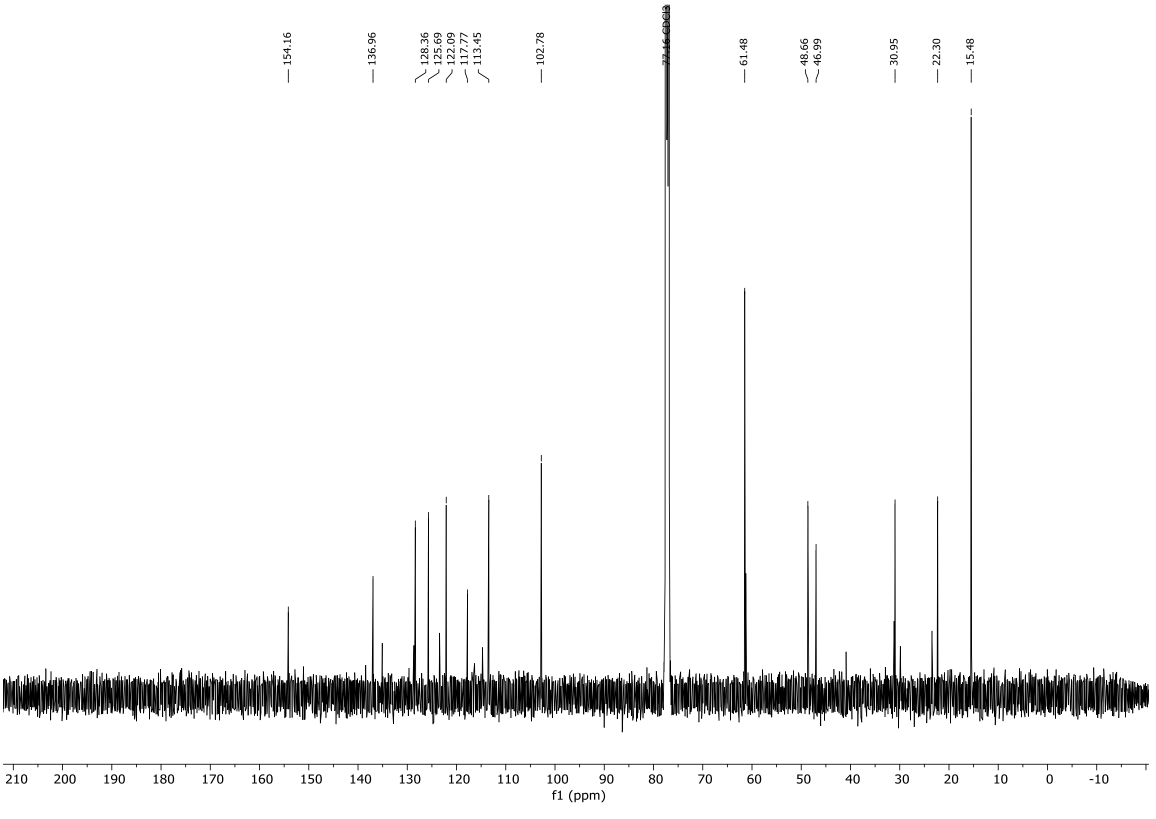

*10.2.25. 3-(4,4-Dimethoxy-2,2-dimethylbutyl)-3,4-dihydroquinazolin-2(1H)-one (**SI-8ac**)*

^{1}H NMR (500 MHz, $CDCl_3$):

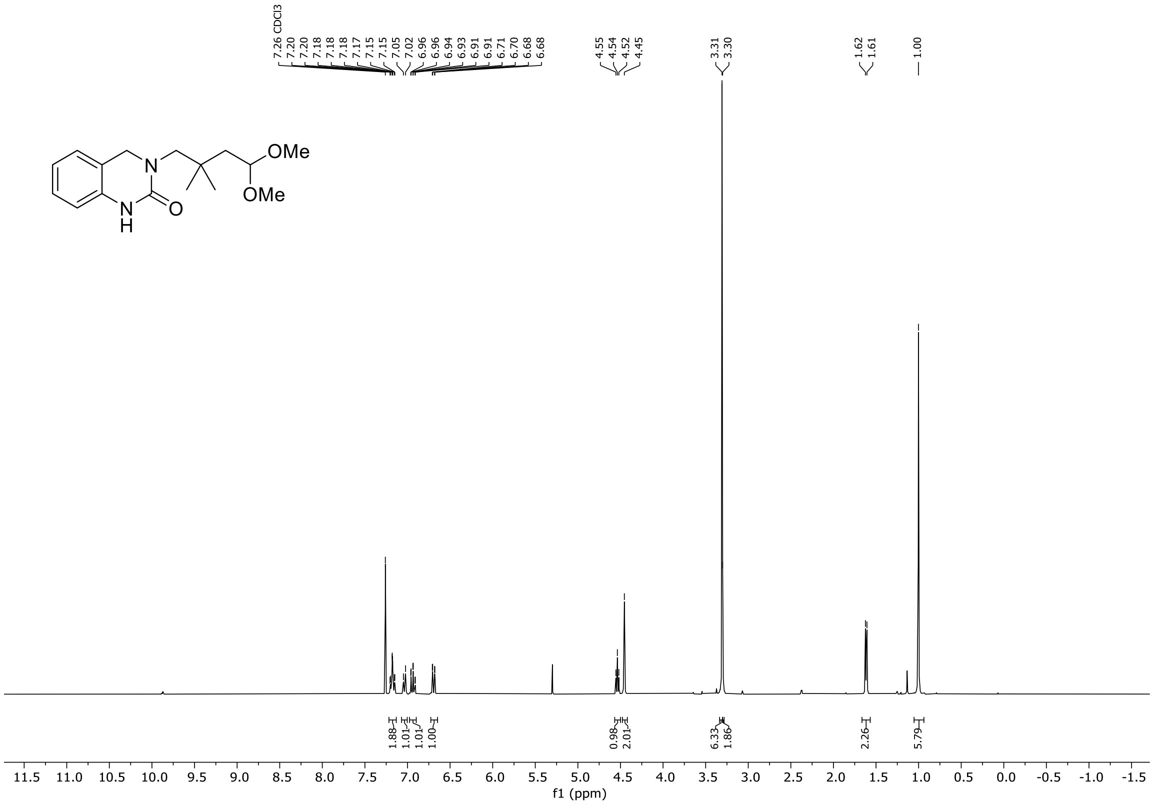

^{13}C NMR (126 MHz, $CDCl_3$):

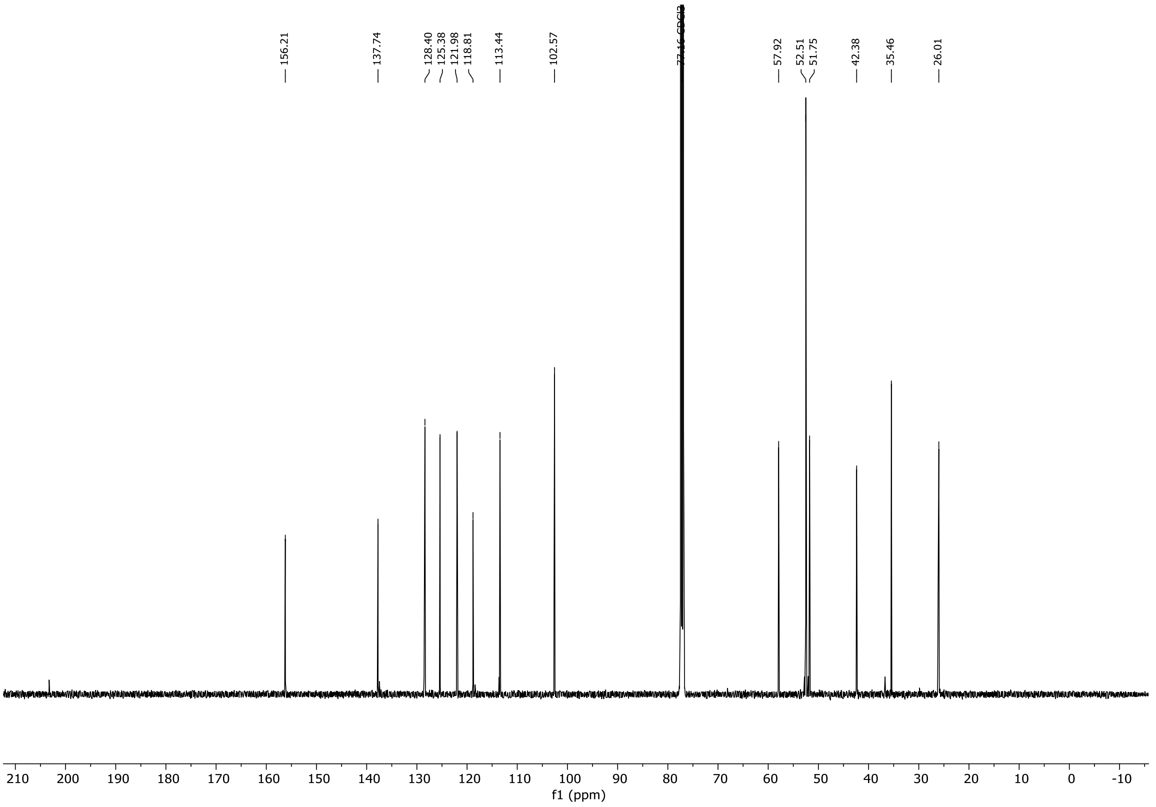

*10.2.26. 3-(2-(1,3-Dioxolan-2-yl)phenethyl)-3,4-dihydroquinazolin-2(1H)-one (**SI-8ba**)*

^{1}H NMR (500 MHz, $CDCl_3$):

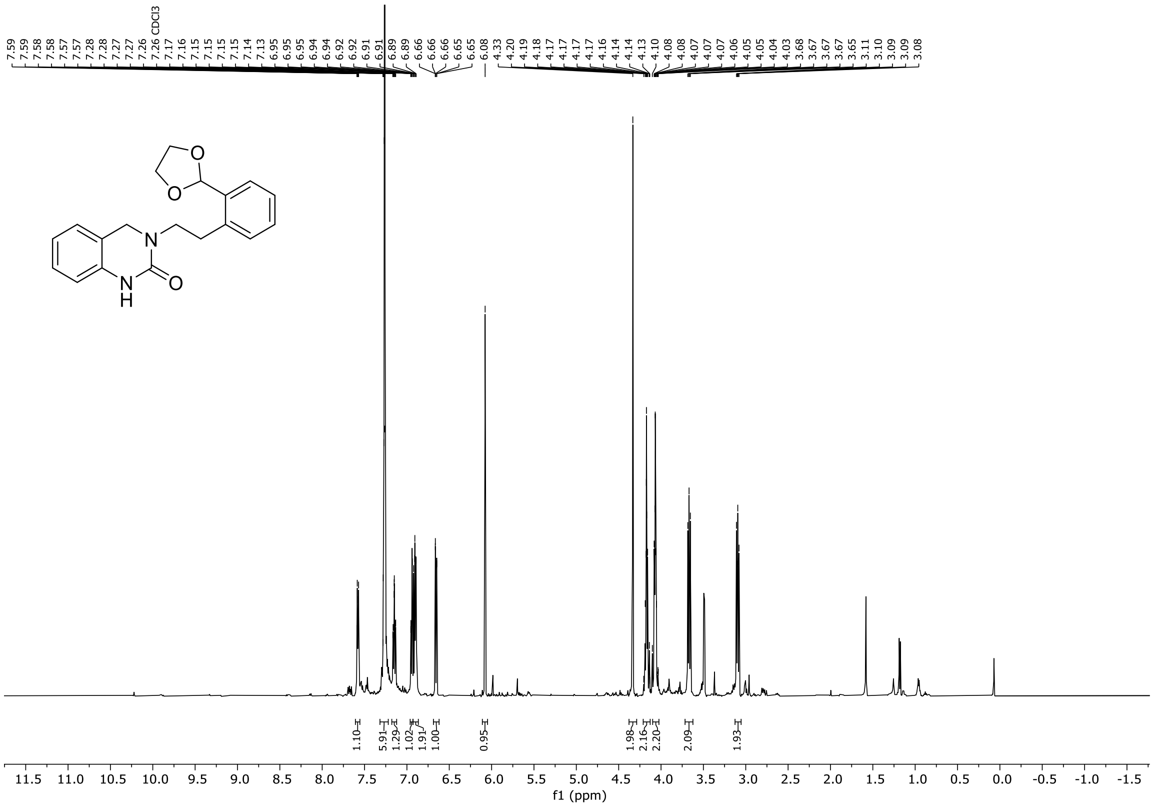

^{13}C NMR (126 MHz, $CDCl_3$):

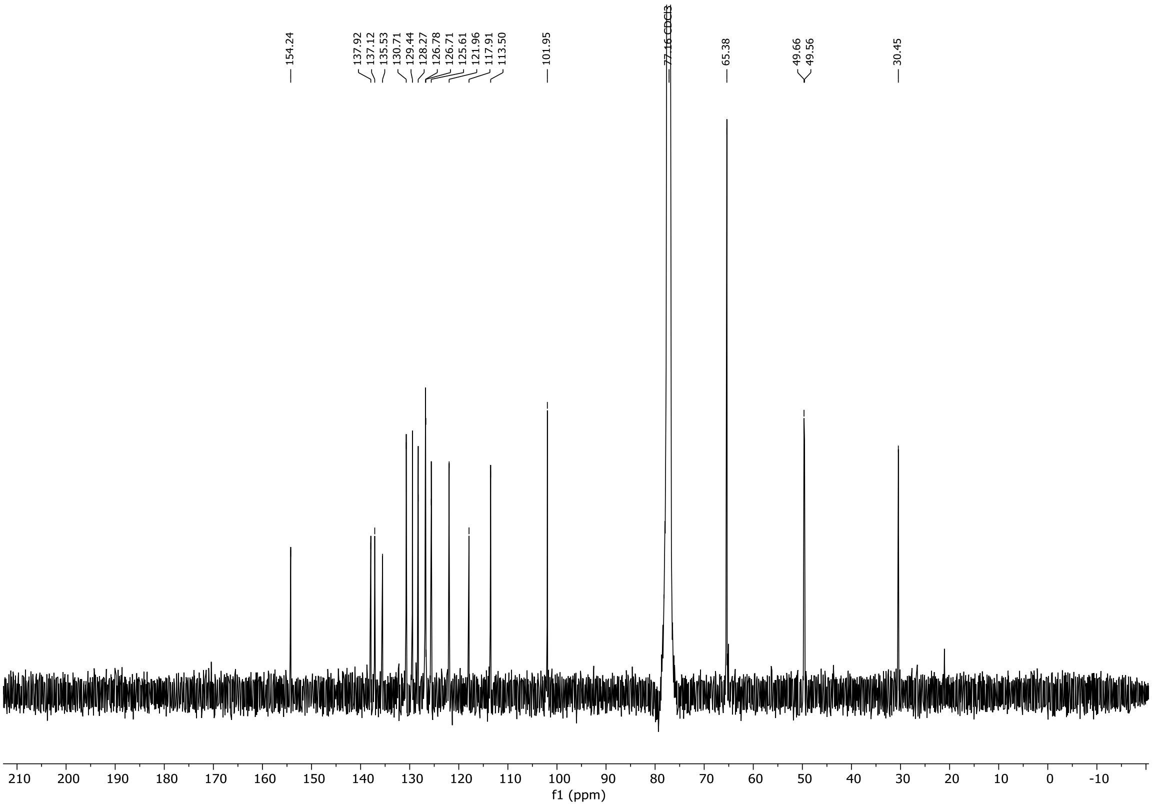

*10.2.27. 3-(2-(Benzo[d][1,3]dioxol-5-yl)ethyl)-3,4-dihydroquinazolin-2(1H)-one (**SI-8bb**)*

^{1}H NMR (500 MHz, $CDCl_3$):

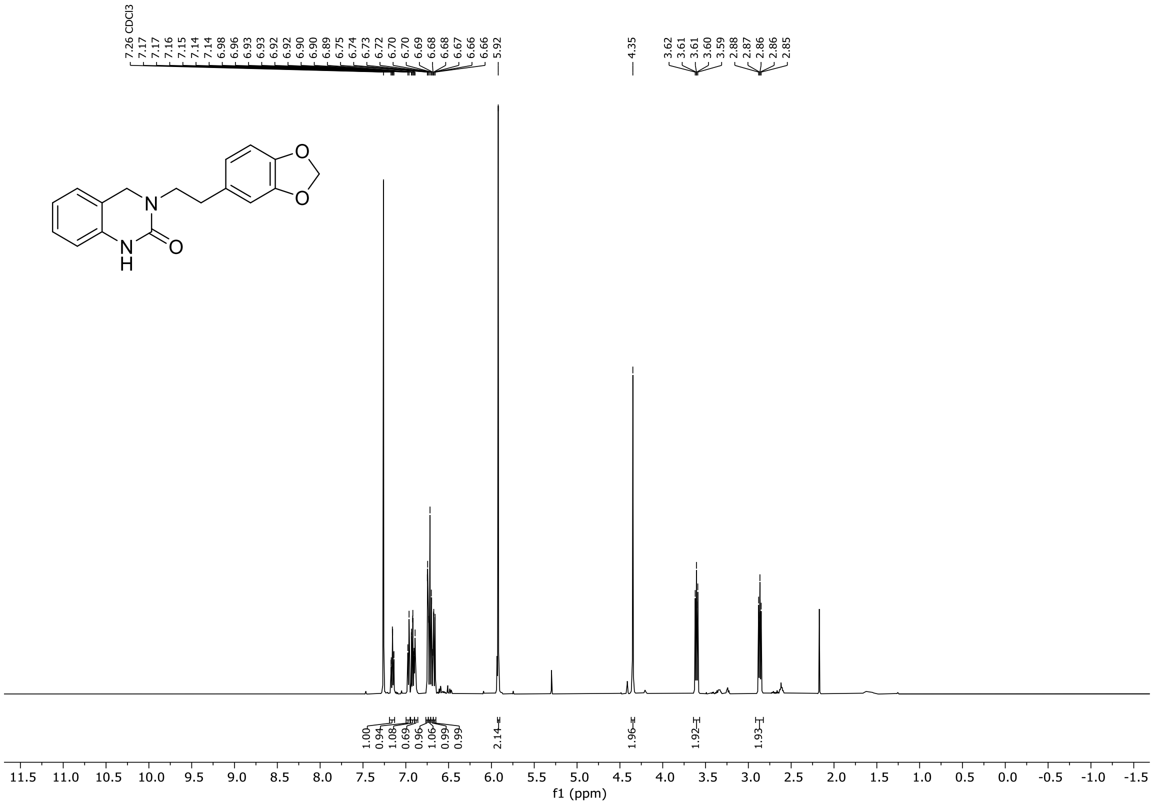

^{13}C NMR (126 MHz, $CDCl_3$):

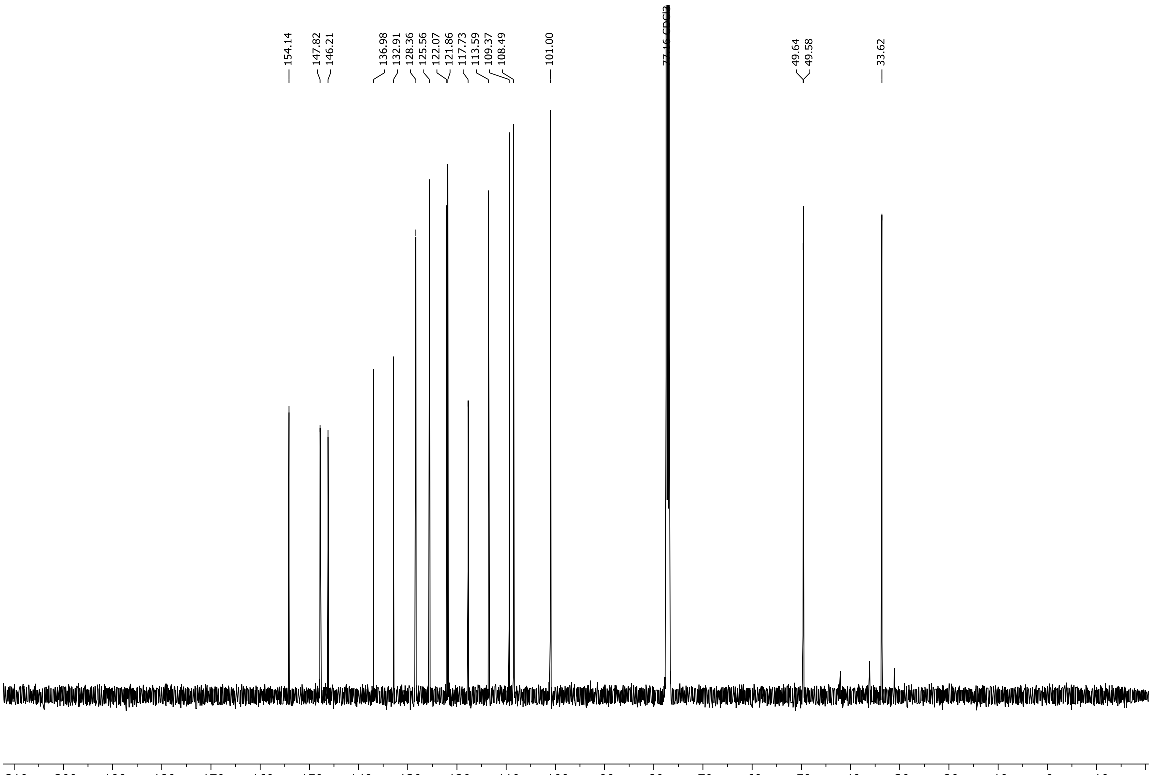

*10.2.28. 3-(3,4-Dimethoxyphenethyl)-3,4-dihydroquinazolin-2(1H)-one (**SI-8bc**)*

^{1}H NMR (500 MHz, $CDCl_3$):

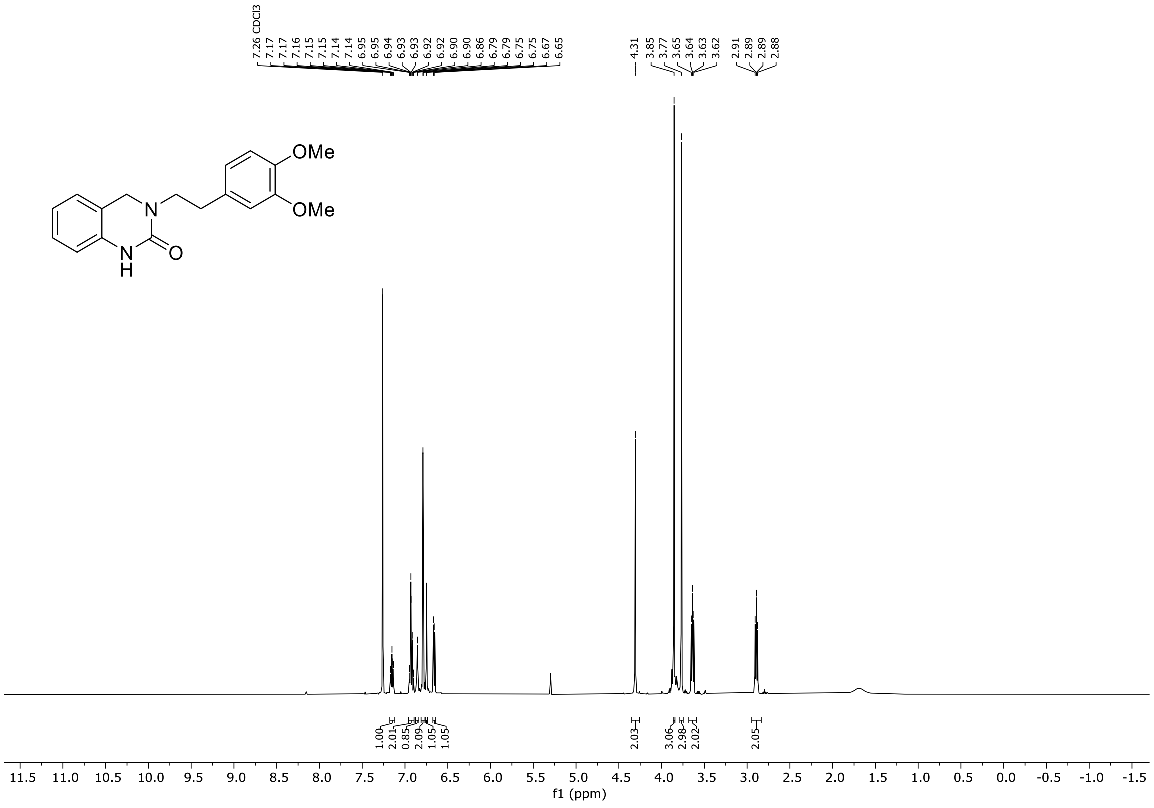

^{13}C NMR (126 MHz, $CDCl_3$):

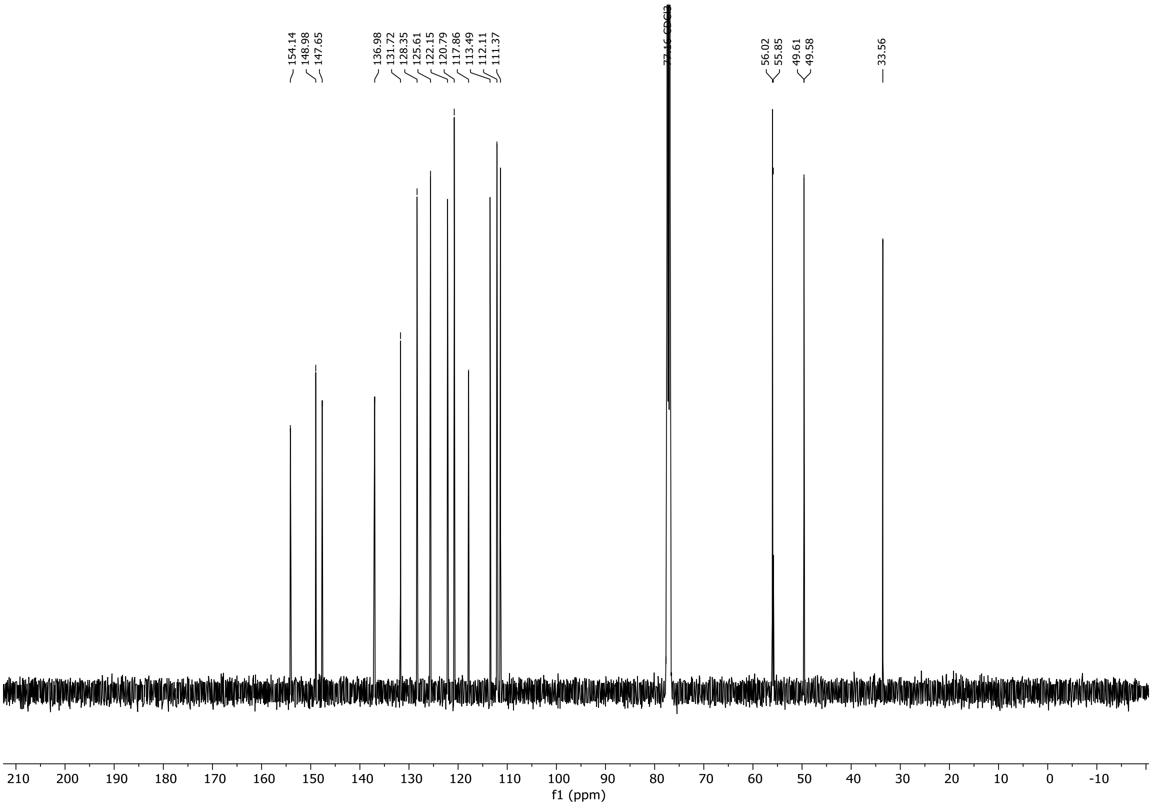

*10.2.29. 3-(3,4-Dimethoxyphenethyl)-3,4-dihydroquinazolin-2(1H)-one (**SI-8bc**)*

^{1}H NMR (500 MHz, $CDCl_3$):

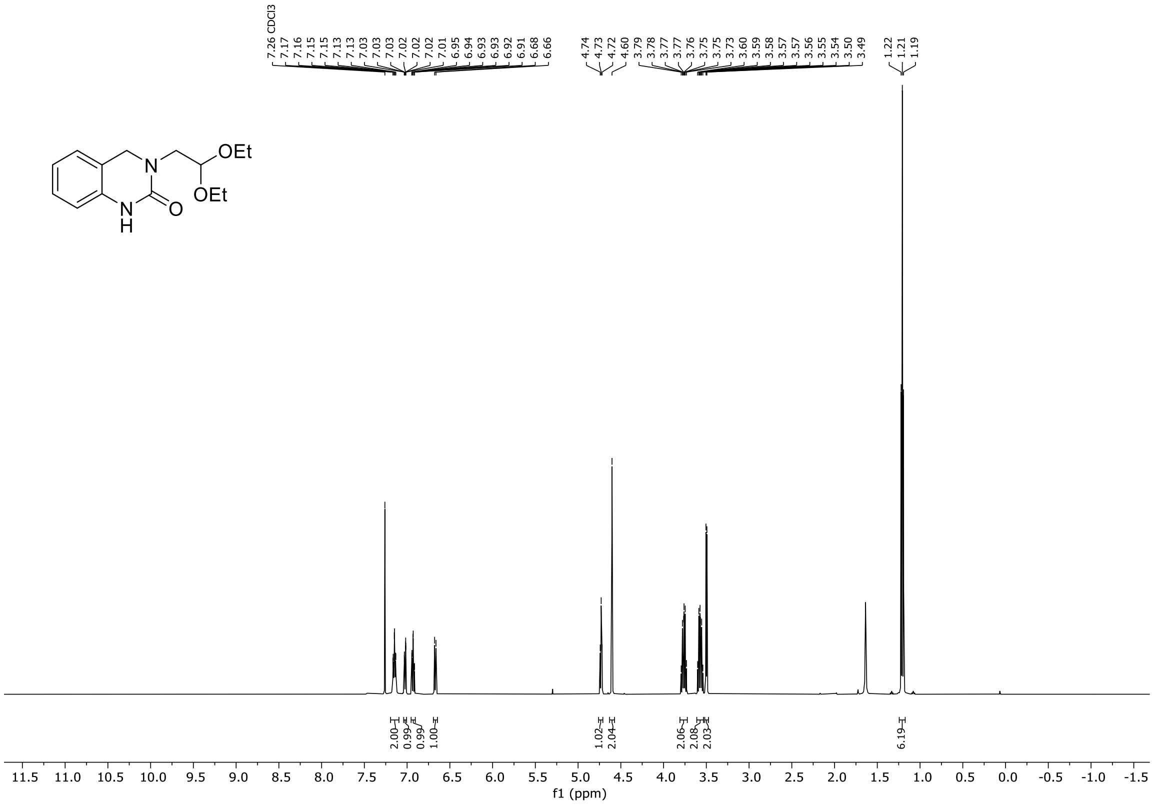

^{13}C NMR (126 MHz, $CDCl_3$):

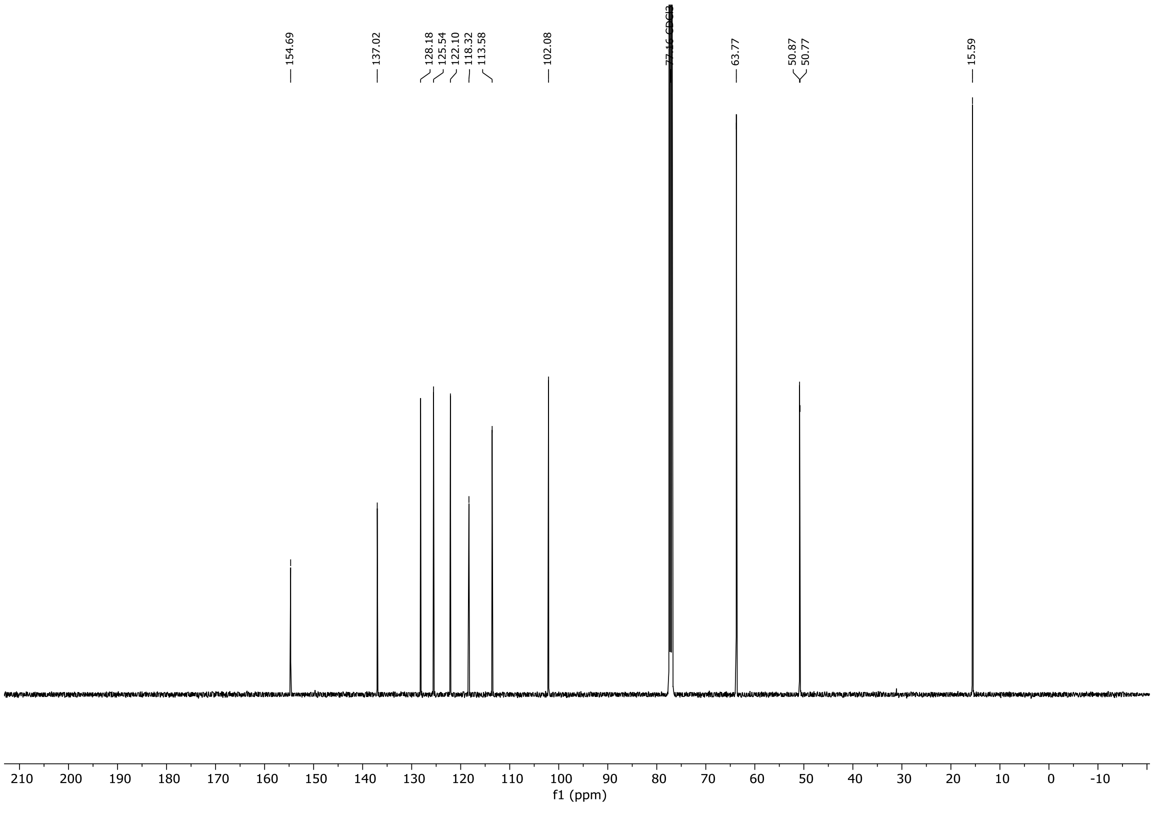

10.3. Hydrazone Substrates

*10.3.1. N'-(3-(2-Oxo-1,4-dihydroquinazolin-3(2H)-yl)propylidene)-4-methylbenzenesulfonohydrazide (**4a**)*

^{1}H NMR (500 MHz, DMSO-d_6):

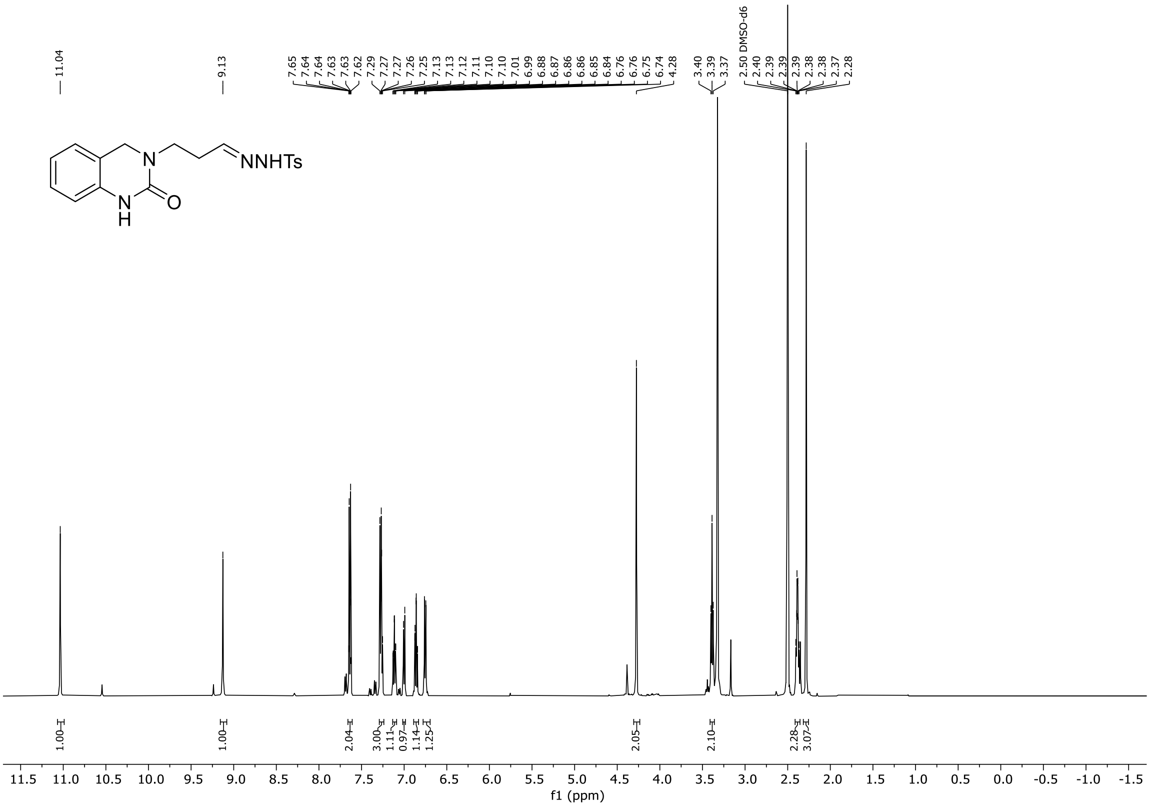

^{13}C NMR (126 MHz, DMSO-d_6):

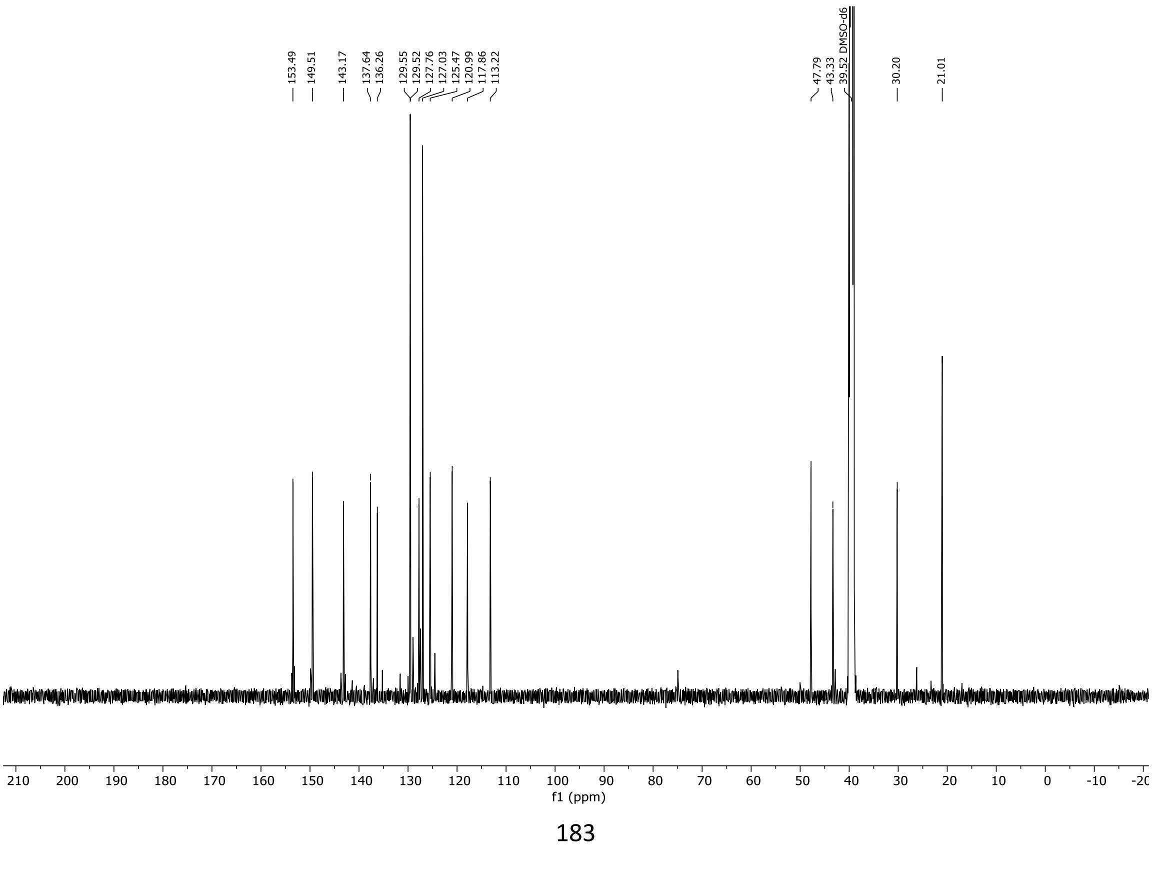

*10.3.2. N'-(3-(5-Bromo-2-oxo-1,4-dihydroquinazolin-3(2H)-yl)propylidene)-4-methylbenzenesulfonohydrazide (**4b**)*

^{1}H NMR (500 MHz, DMSO-d_6):

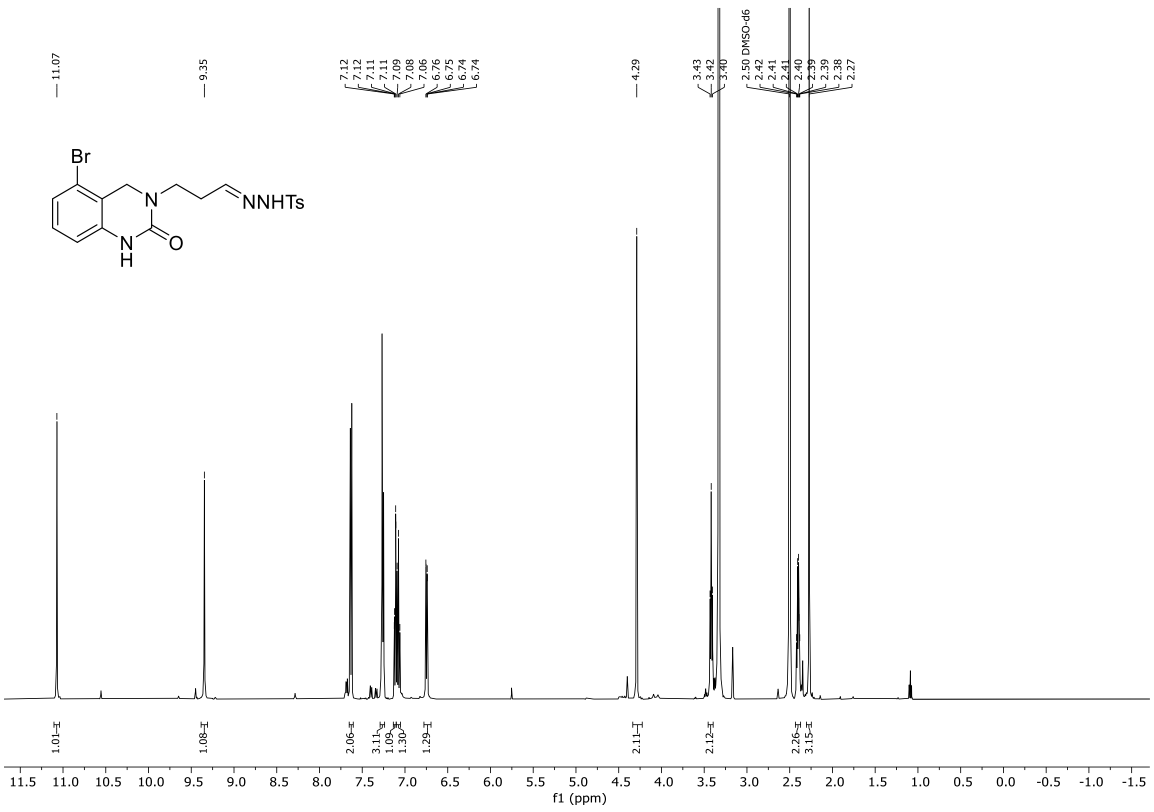

^{13}C NMR (126 MHz, DMSO-d_6):

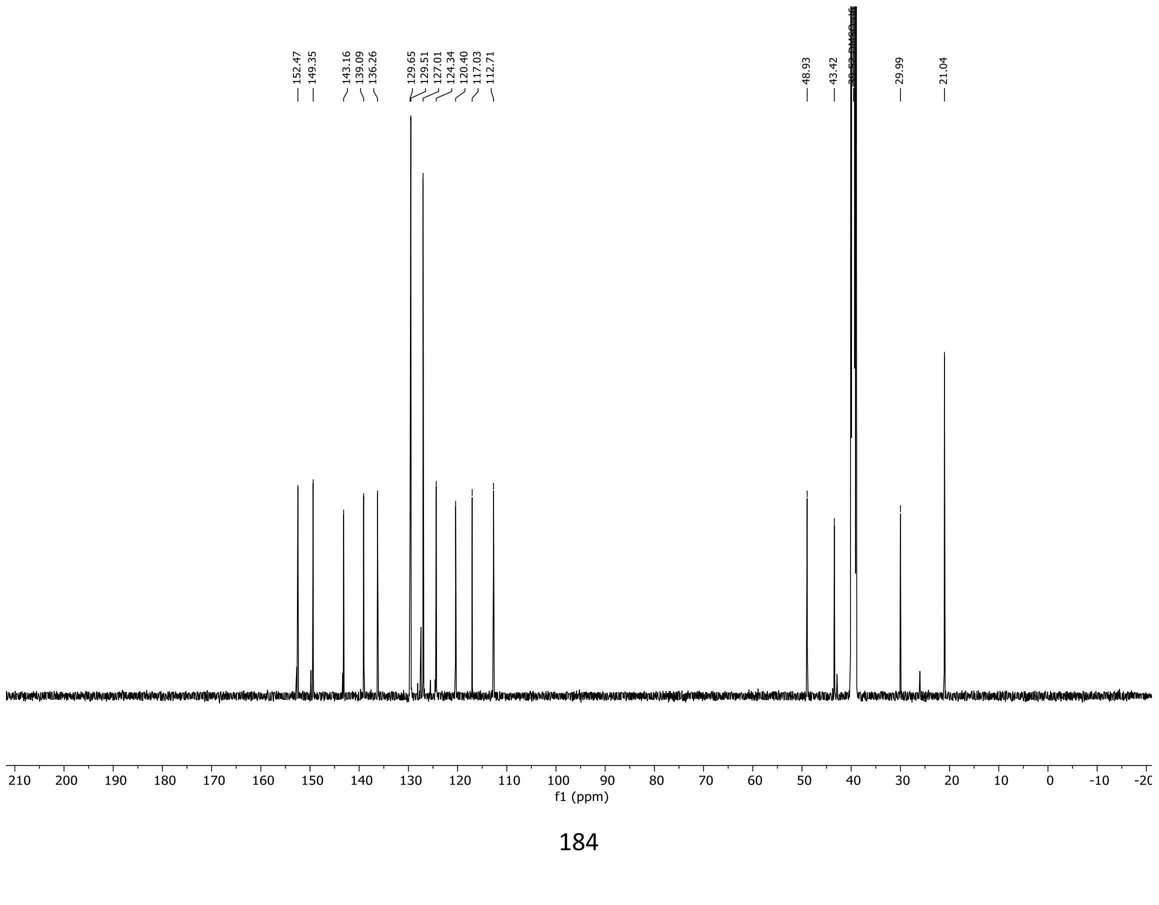

*10.3.3. N'-(3-(6-Bromo-2-oxo-1,4-dihydroquinazolin-3(2H)-yl)propylidene)-4-methylbenzenesulfonohydrazide **(4c)***

^{1}H NMR (500 MHz, DMSO-d_6):

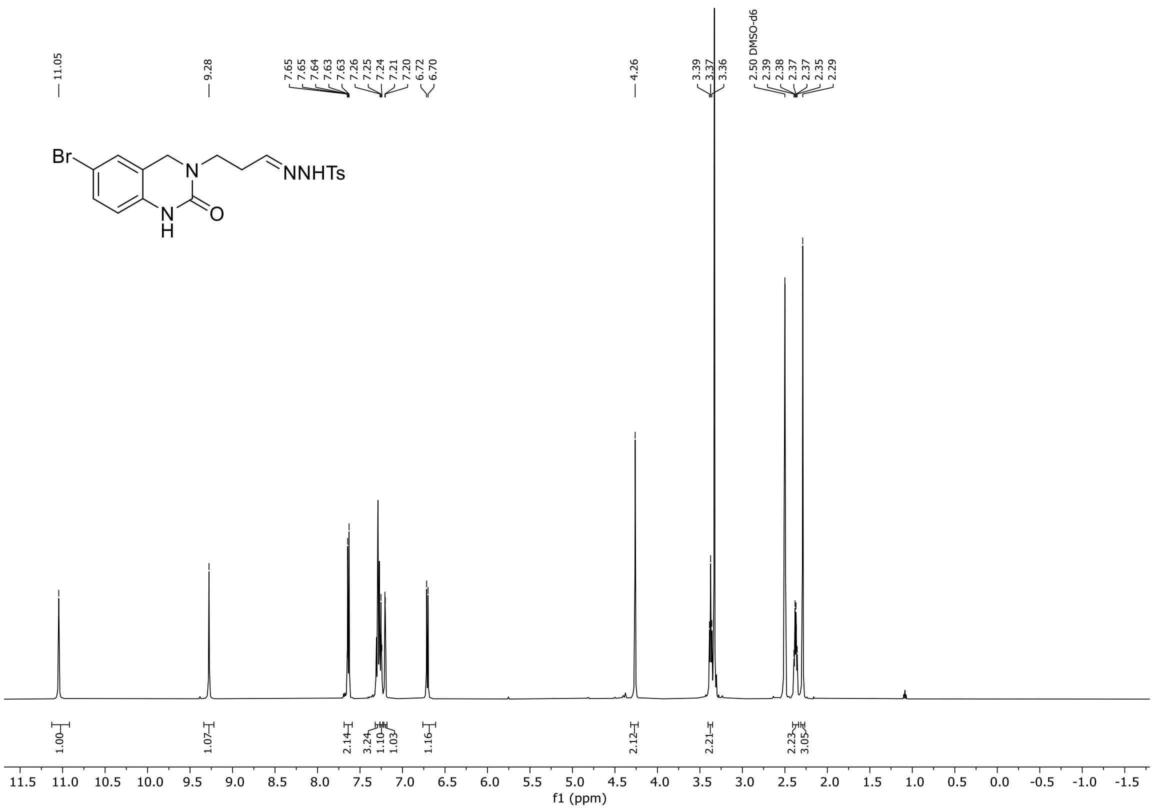

^{13}C NMR (126 MHz, DMSO-d_6):

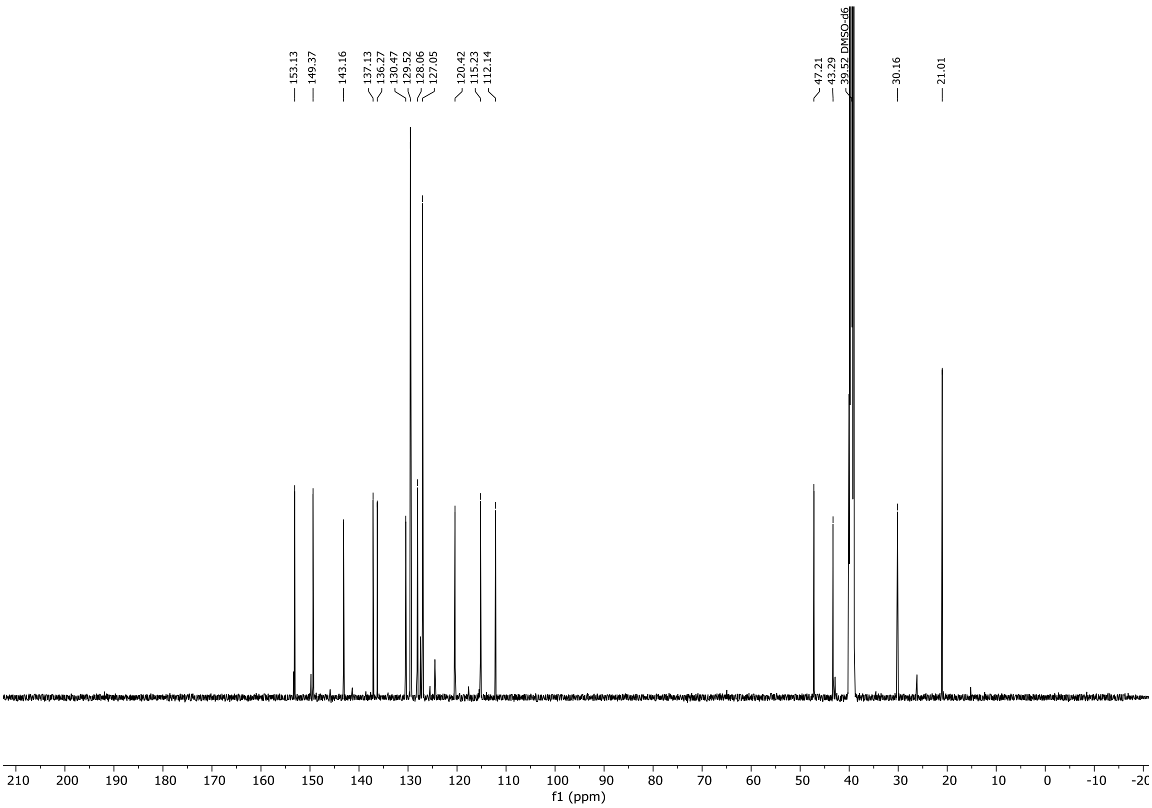

10.3.4. N'-(3-(7-Bromo-2-oxo-1,4-dihydroquinazolin-3(2H)-yl)propylidene)-4-methylbenzenesulfonohydrazide ***(4d)***

^{1}H NMR (500 MHz, DMSO-d_6):

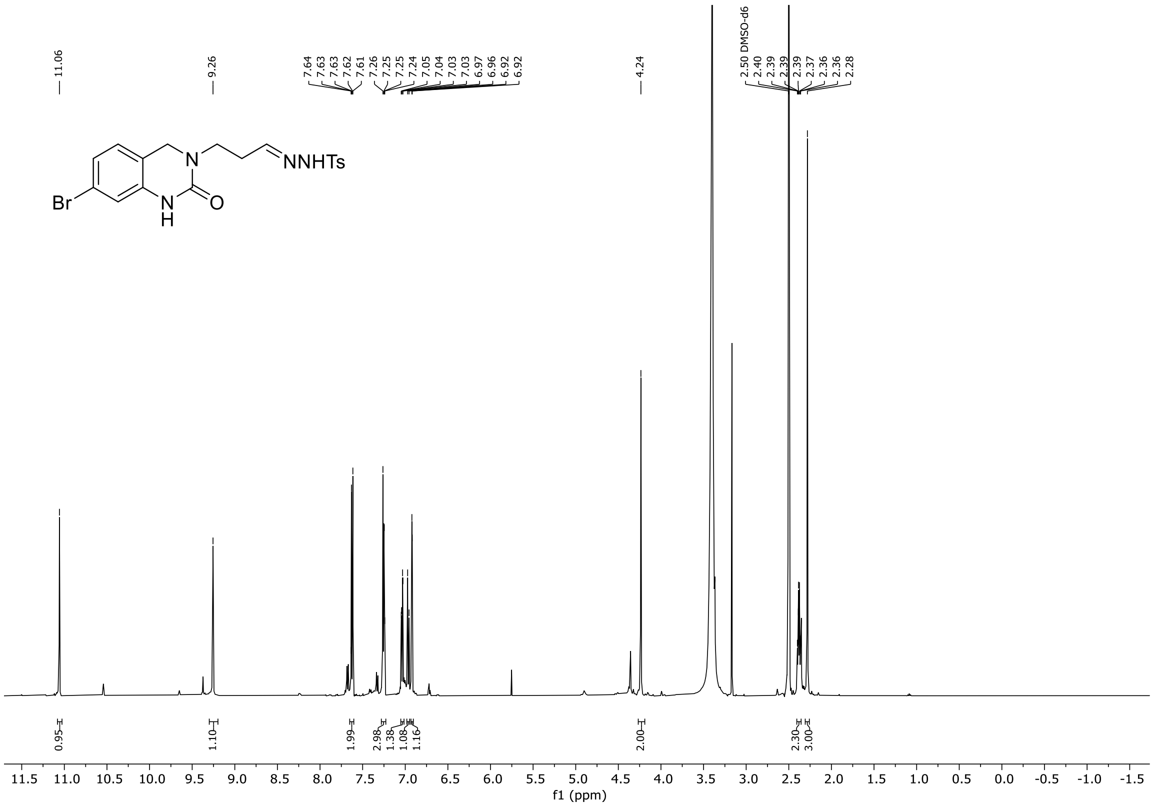

^{13}C NMR (126 MHz, DMSO-d_6):

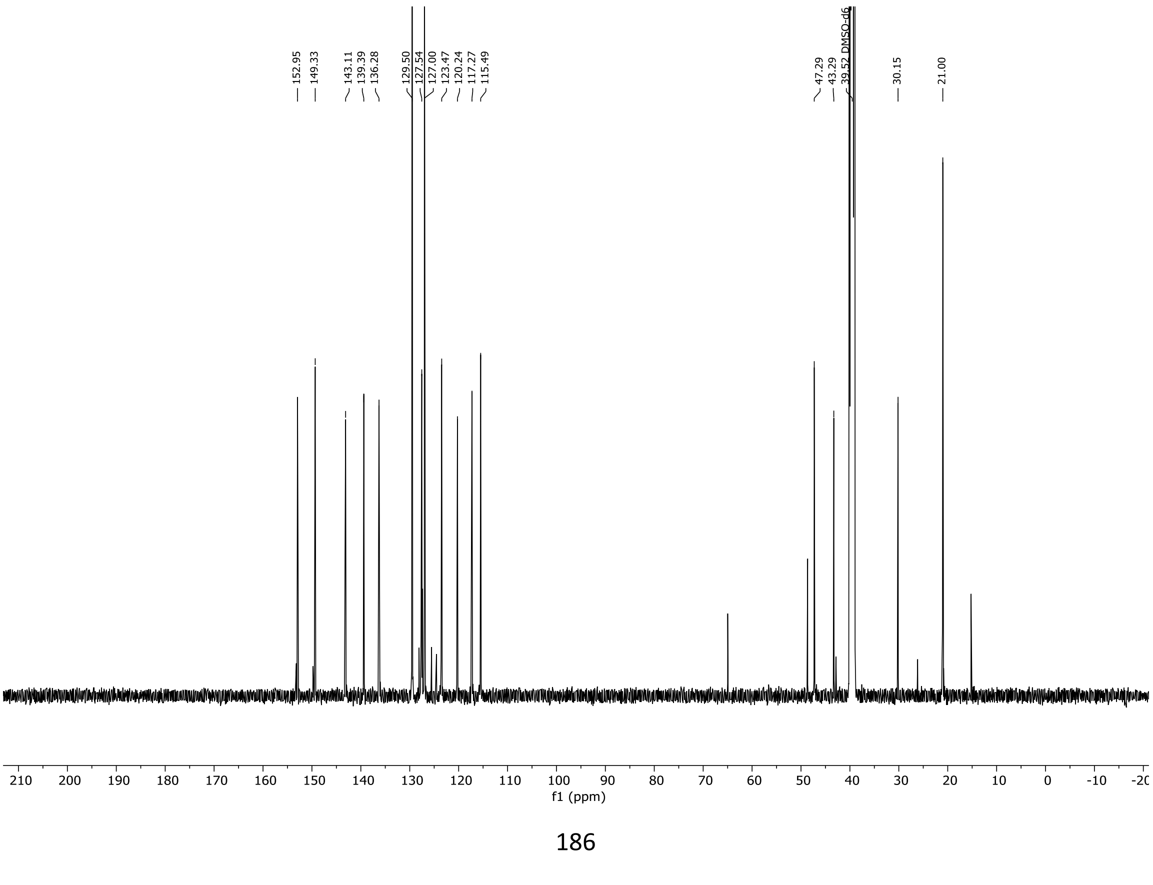

10.3.5. N'-(3-(8-Bromo-2-oxo-1,4-dihydroquinazolin-3(2H)-yl)propylidene)-4-methylbenzenesulfonohydrazide ***(4e)***

^{1}H NMR (500 MHz, DMSO-d_6):

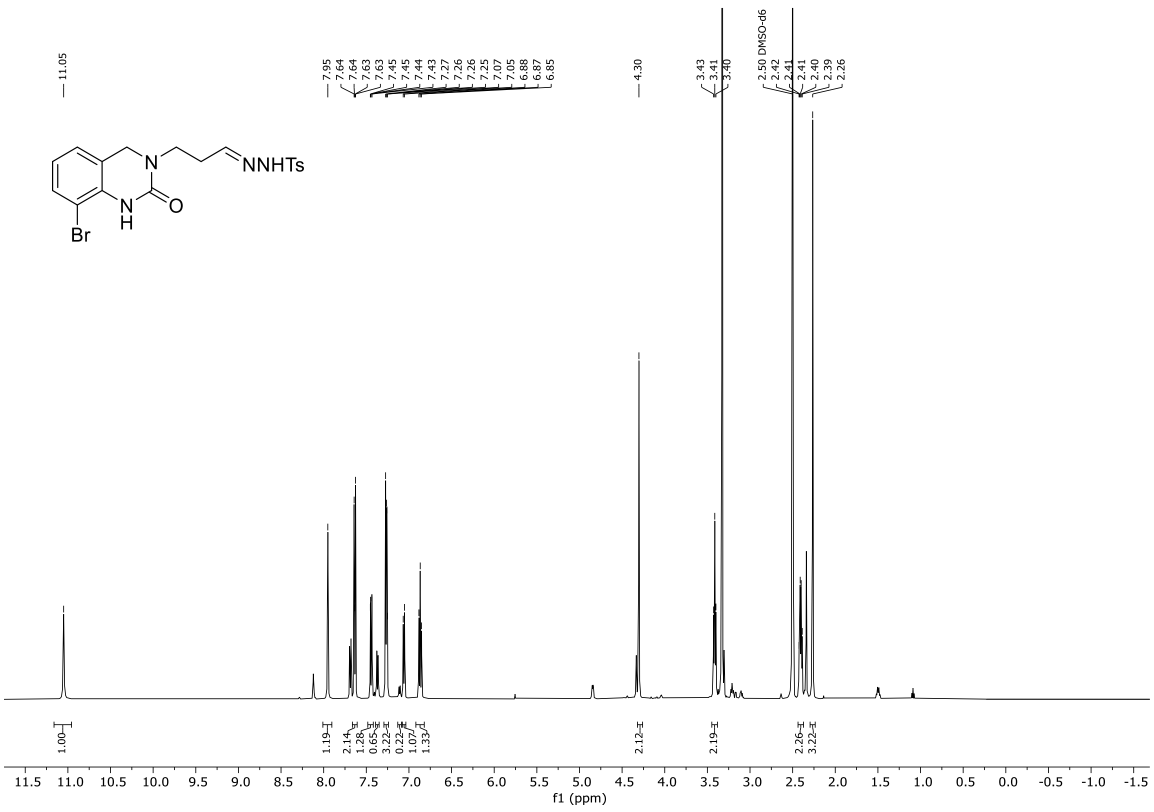

^{13}C NMR (126 MHz, DMSO-d_6):

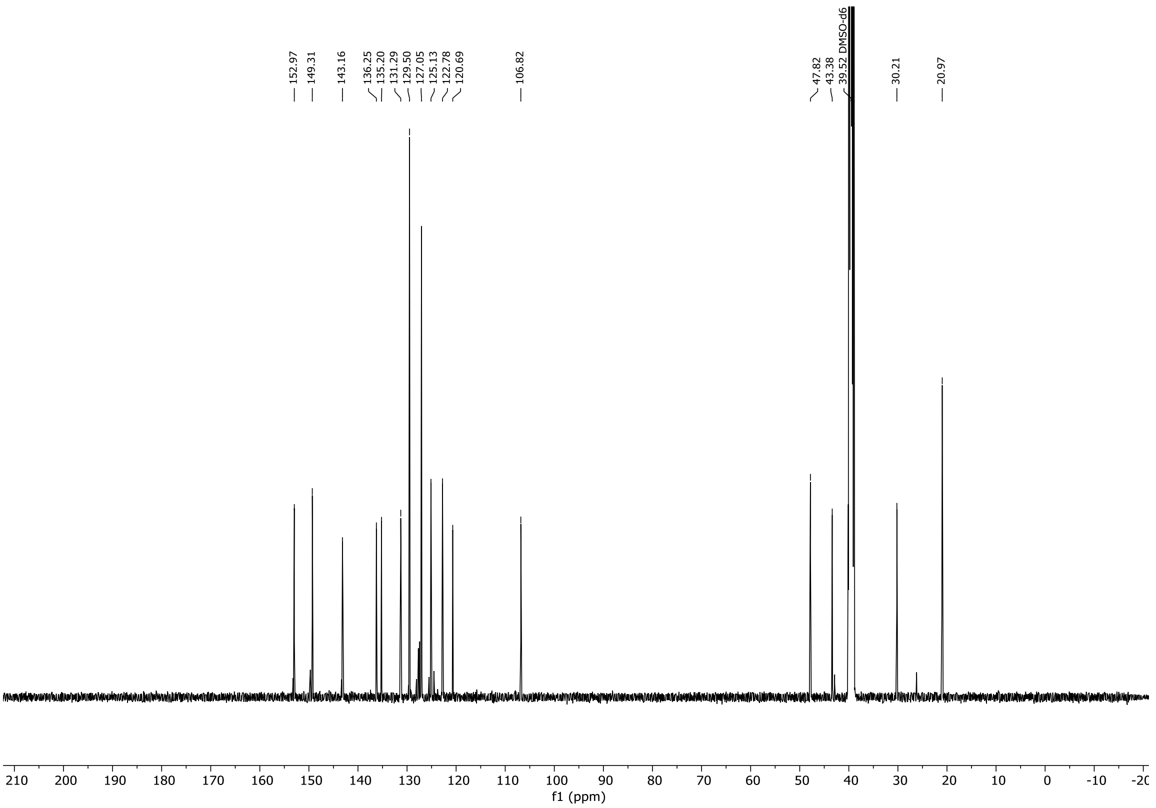

10.3.6. N'-(3-(6-Chloro-2-oxo-1,4-dihydroquinazolin-3(2H)-yl)propylidene)-4-methylbenzenesulfonohydrazide (4f)

^{1}H NMR (500 MHz, DMSO-d_6):

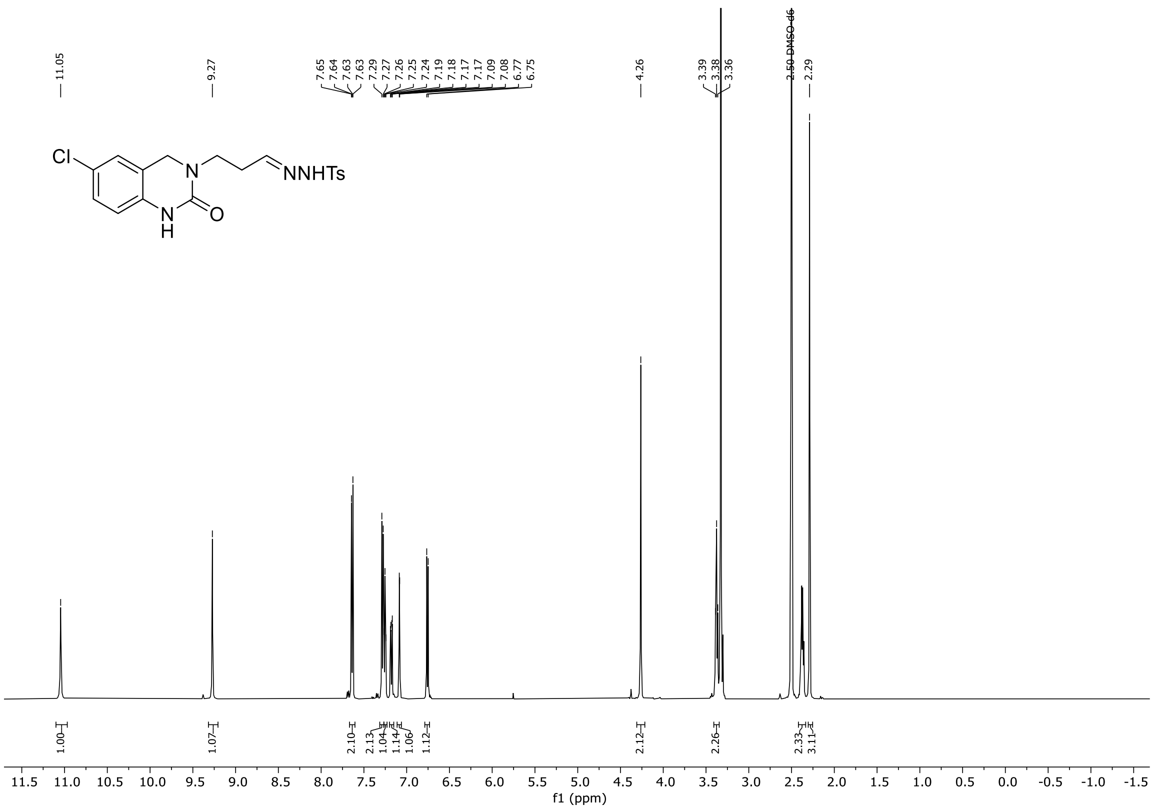

^{13}C NMR (126 MHz, DMSO-d_6):

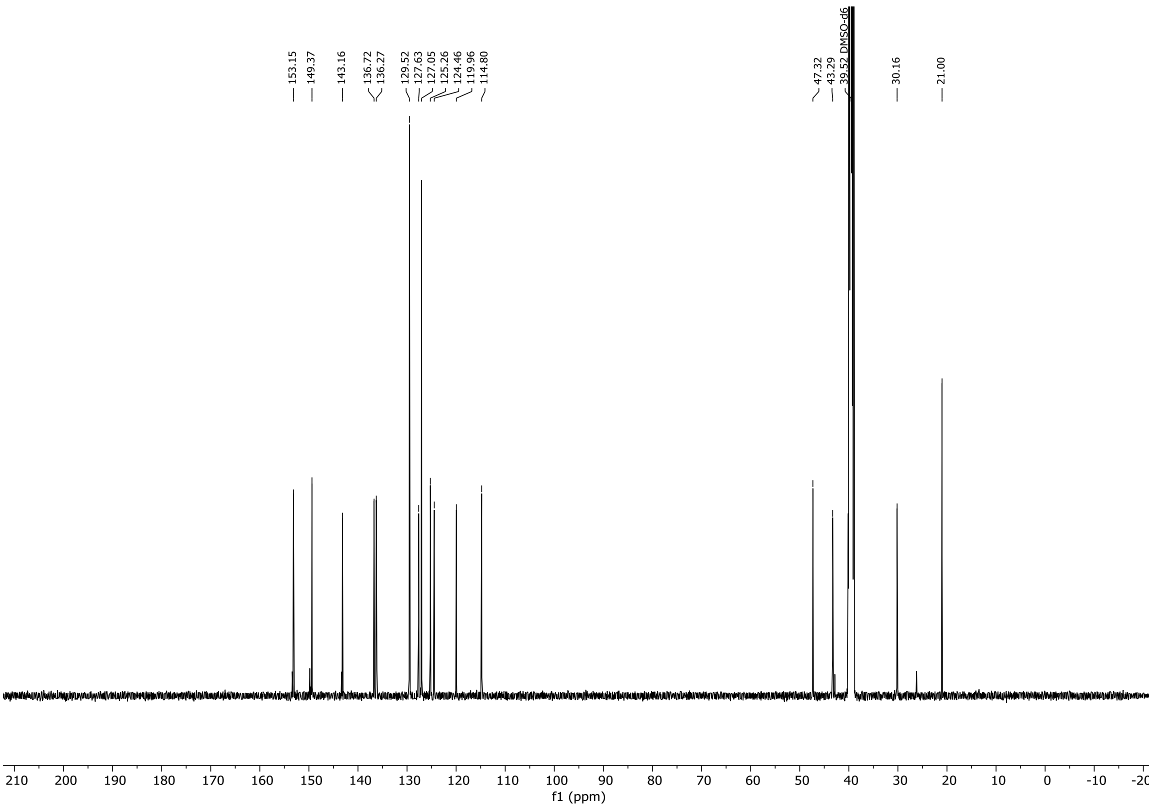

10.3.7. N'-(3-(7-Fluoro-2-oxo-1,4-dihydroquinazolin-3(2H)-yl)propylidene)-4-methylbenzenesulfonohydrazide ***(4g)***

^{1}H NMR (500 MHz, DMSO-d_6):

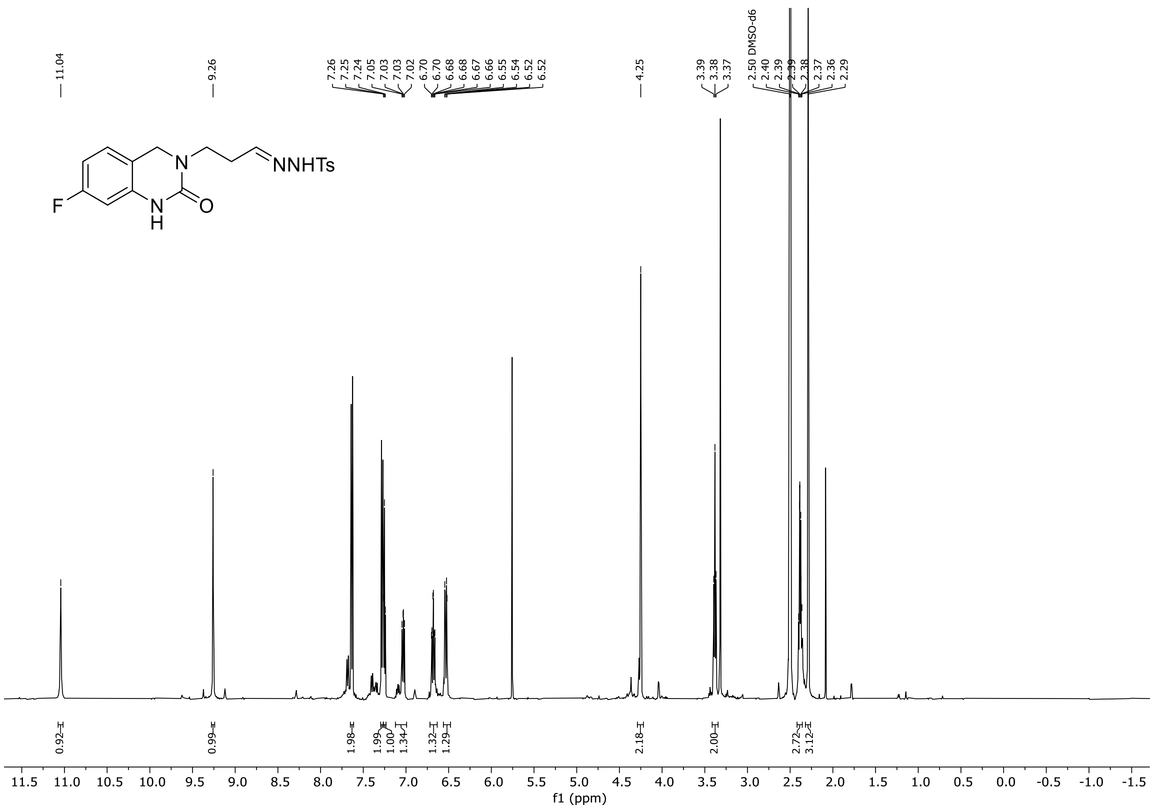

^{13}C NMR (126 MHz, DMSO-d_6):

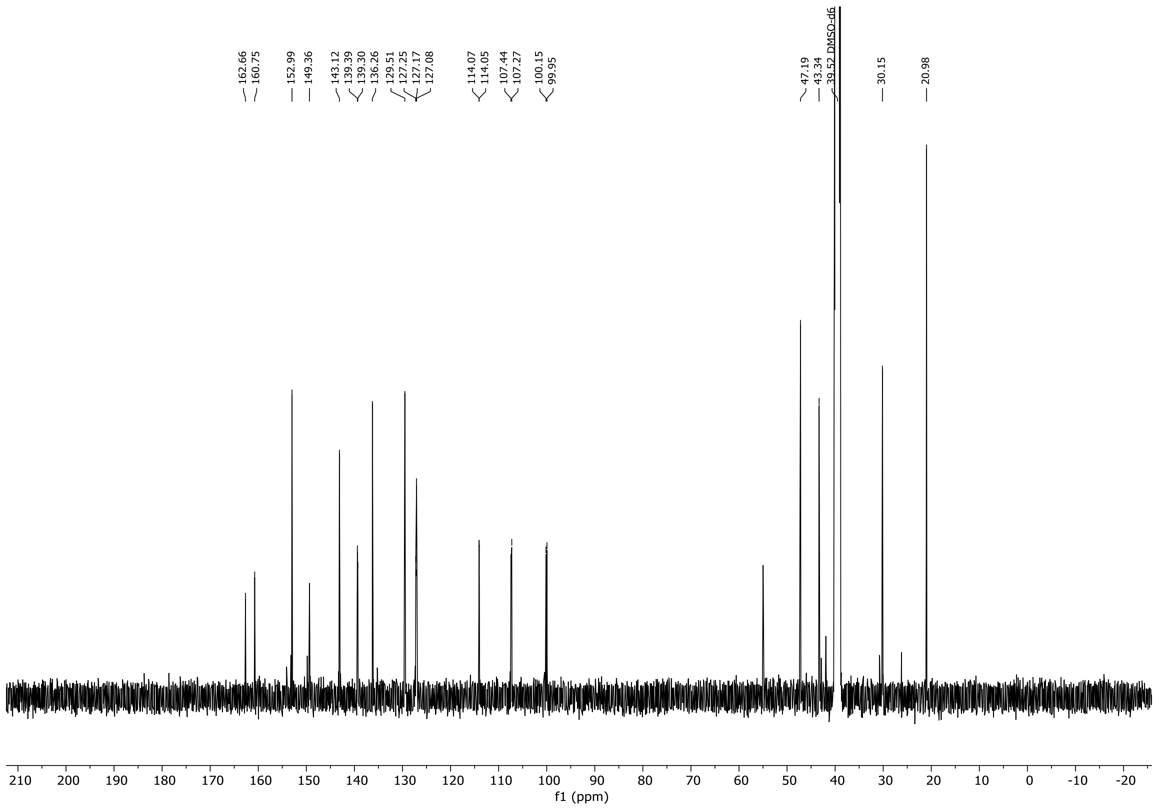

^{19}F NMR (471 MHz, DMSO-d_6):

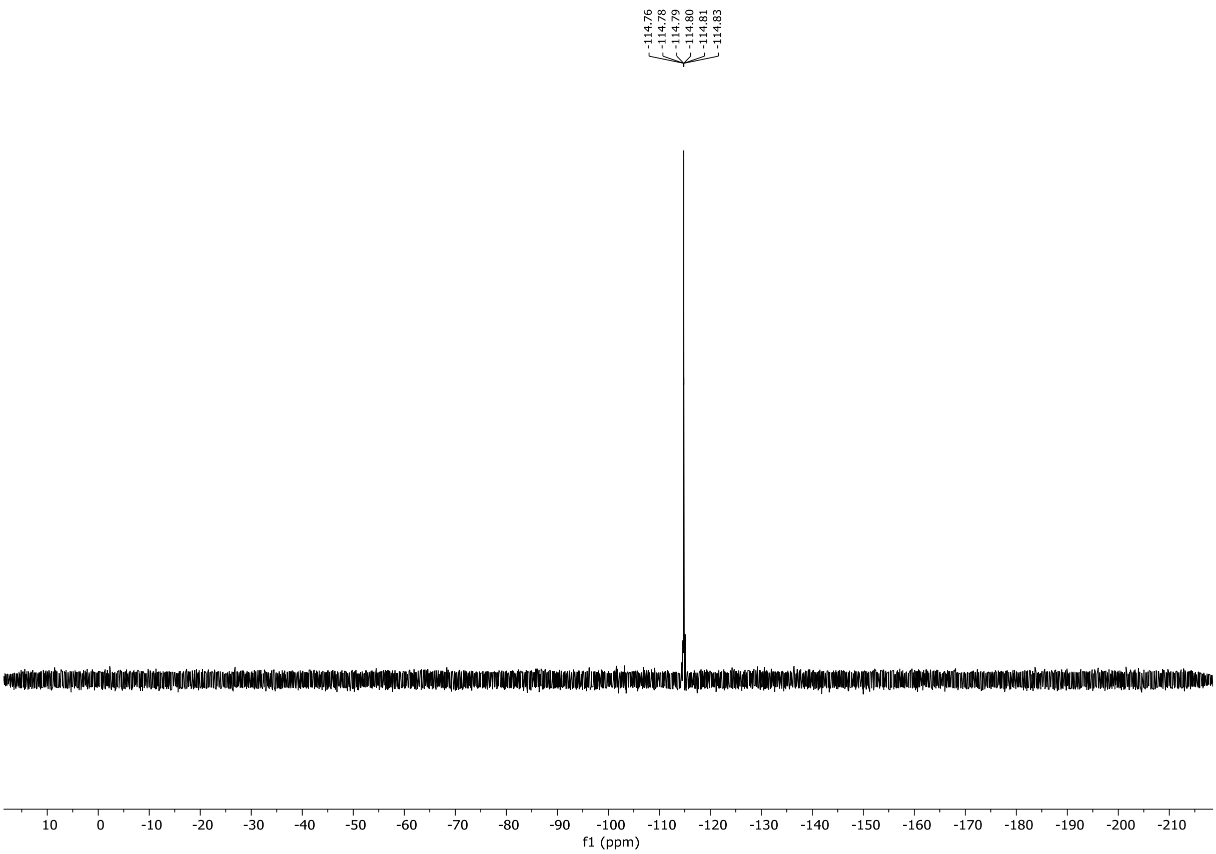

*10.3.8. N'-(3-(2-Oxo-7-(trifluoromethyl)-1,4-dihydroquinazolin-3(2H)-yl)propylidene)-4-methylbenzenesulfonohydrazide (**4h**)*

^{1}H NMR (500 MHz, DMSO-d_6):

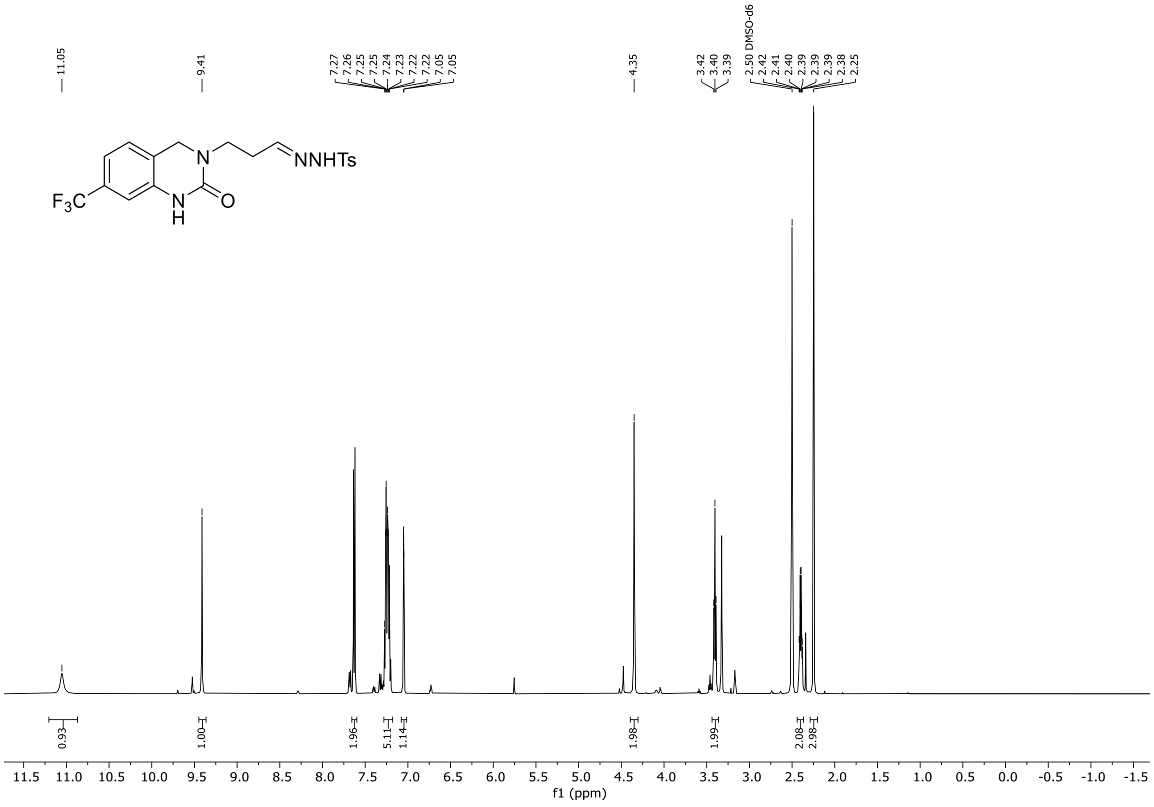

^{13}C NMR (126 MHz, DMSO-d_6):

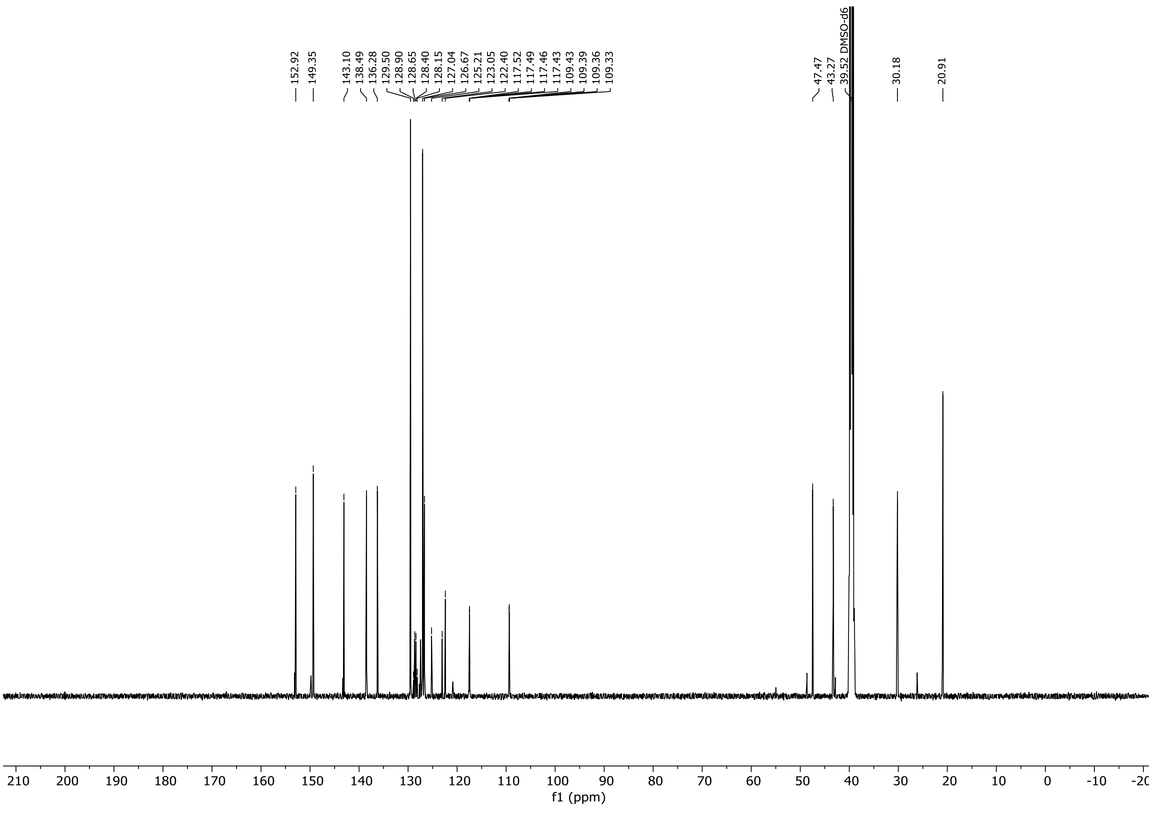

^{19}F NMR (471 MHz, DMSO-d_6):

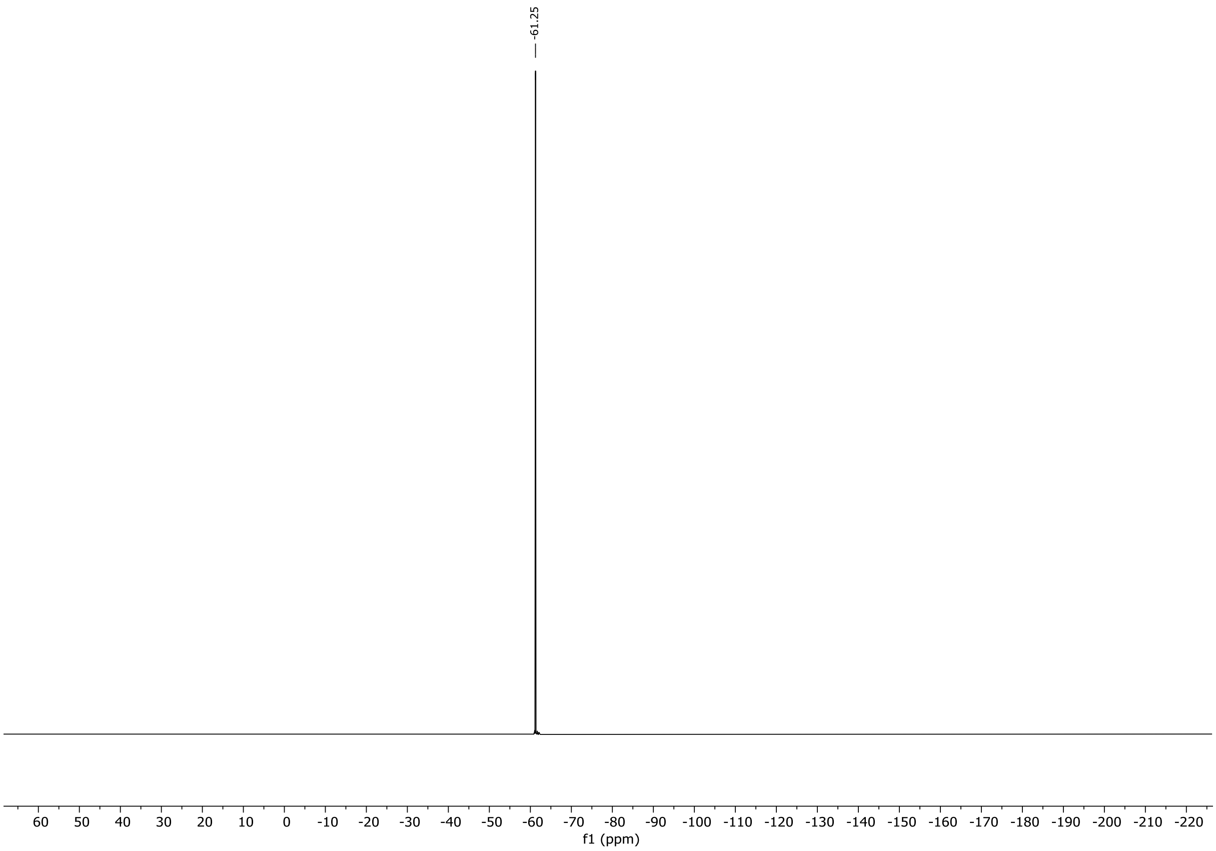

*10.3.9. N'-(3-(2-Oxo-6-vinyl-1,4-dihydroquinazolin-3(2H)-yl)propylidene)-4-methylbenzenesulfonohydrazide **(4i)***

^{1}H NMR (500 MHz, DMSO-d_6):

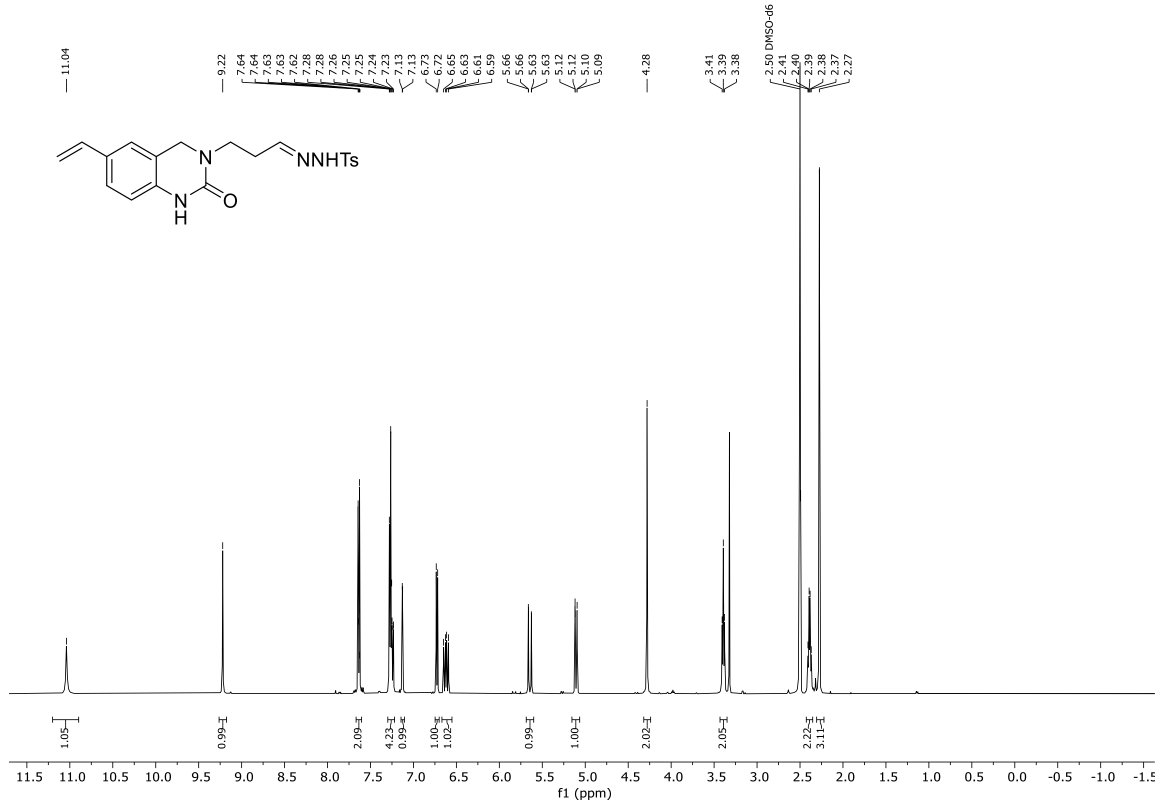

^{13}C NMR (126 MHz, DMSO-d_6):

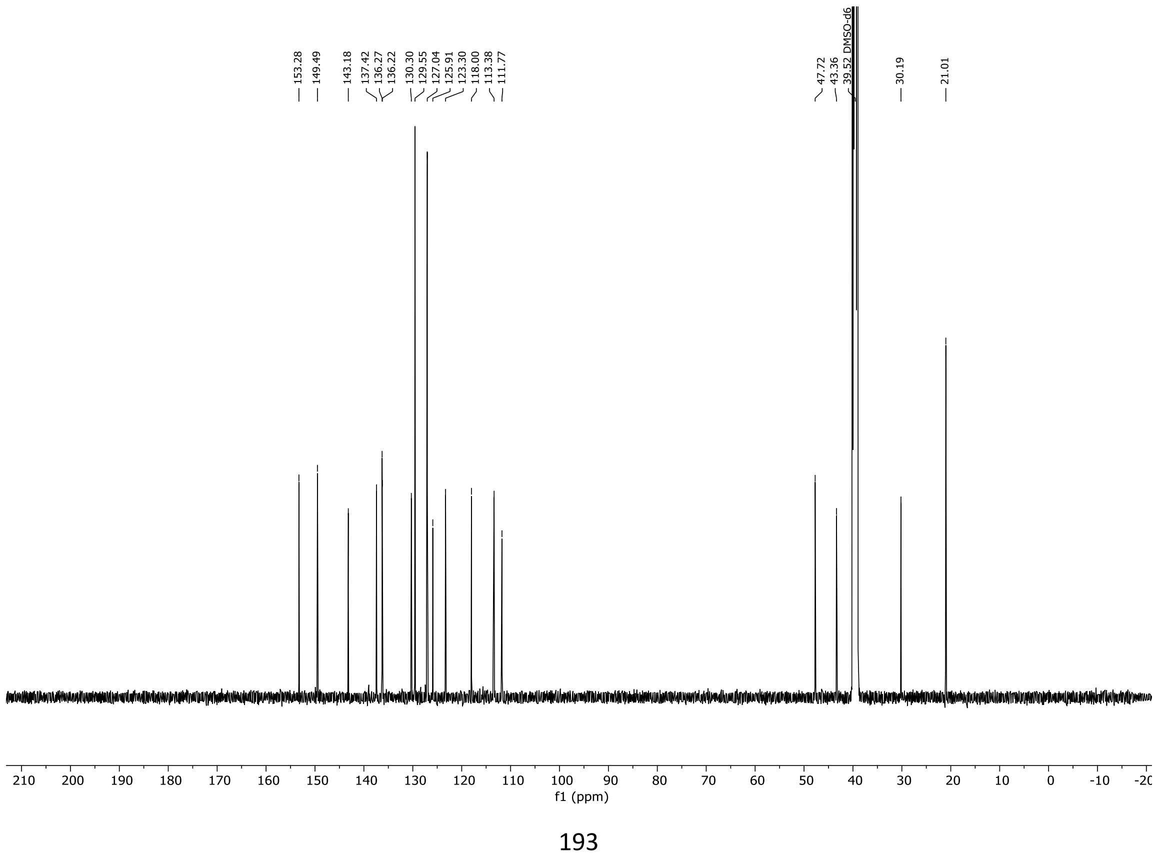

10.3.10. N'-(3-(6-Allyl-2-oxo-1,4-dihydroquinazolin-3(2H)-yl)propylidene)-4-methylbenzenesulfonohydrazide ***(4j)***

^{1}H NMR (500 MHz, DMSO-d_6):

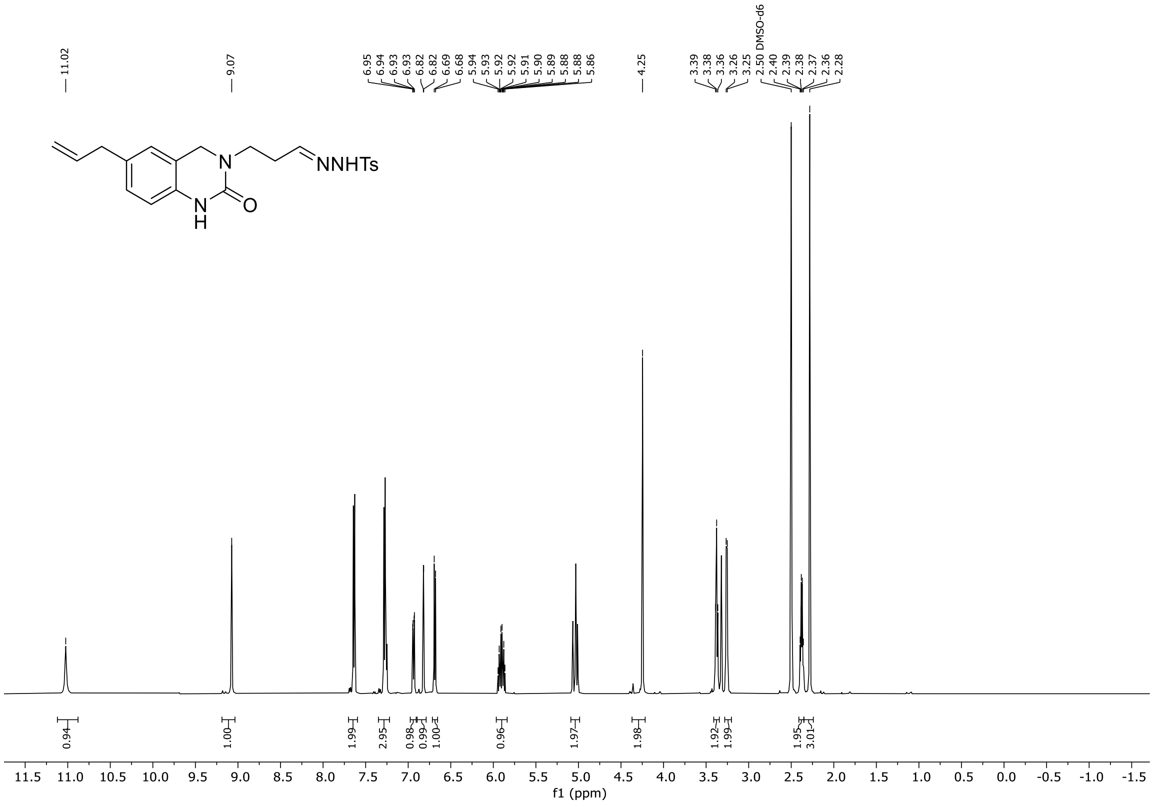

^{13}C NMR (126 MHz, DMSO-d_6):

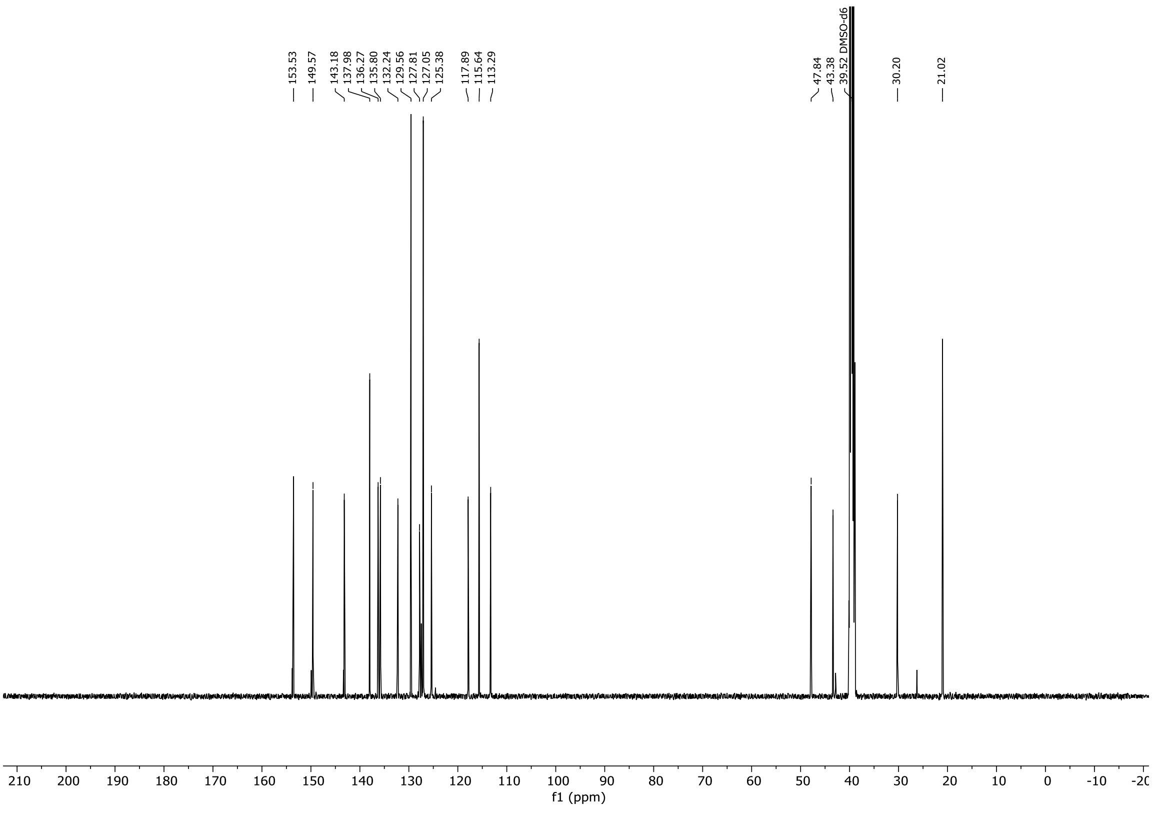

*10.3.11. N'-(3-(8-Methyl-2-oxo-1,4-dihydroquinazolin-3(2H)-yl)propylidene)-4-methylbenzenesulfonohydrazide **(4k)***

^{1}H NMR (500 MHz, DMSO-*d*$_6$):

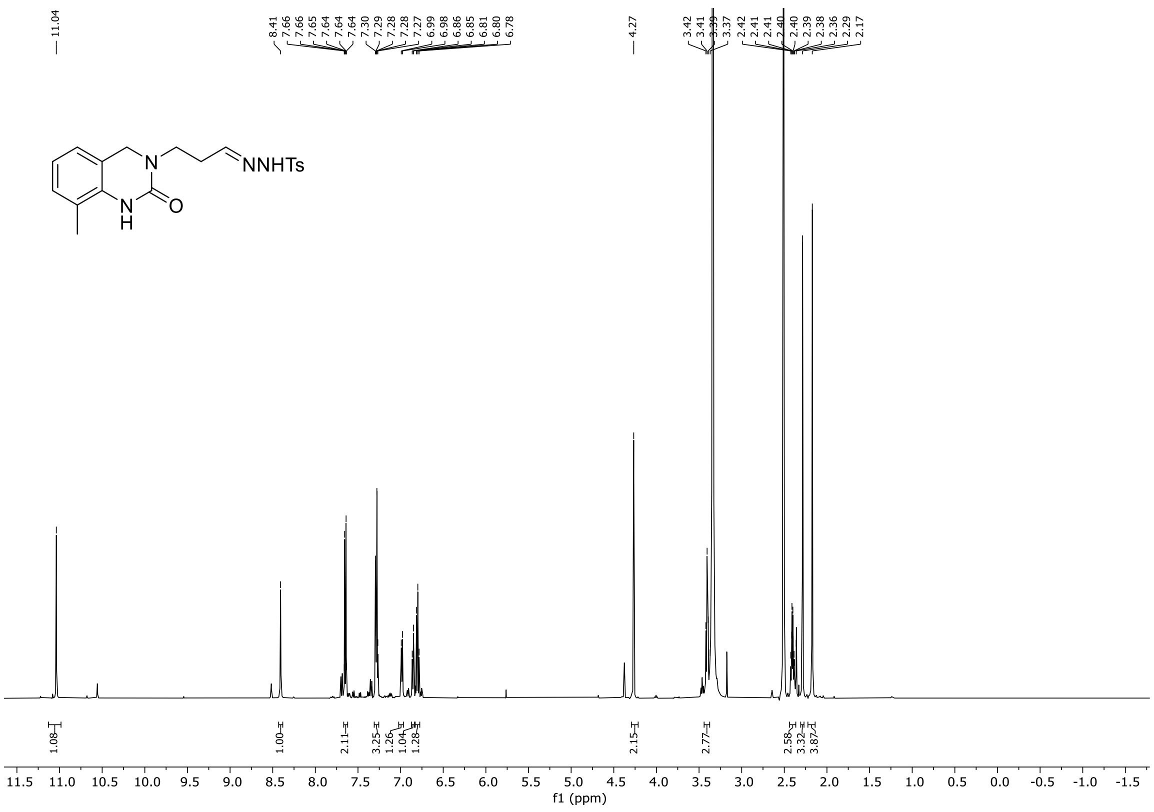

^{13}C NMR (126 MHz, DMSO-*d*$_6$):

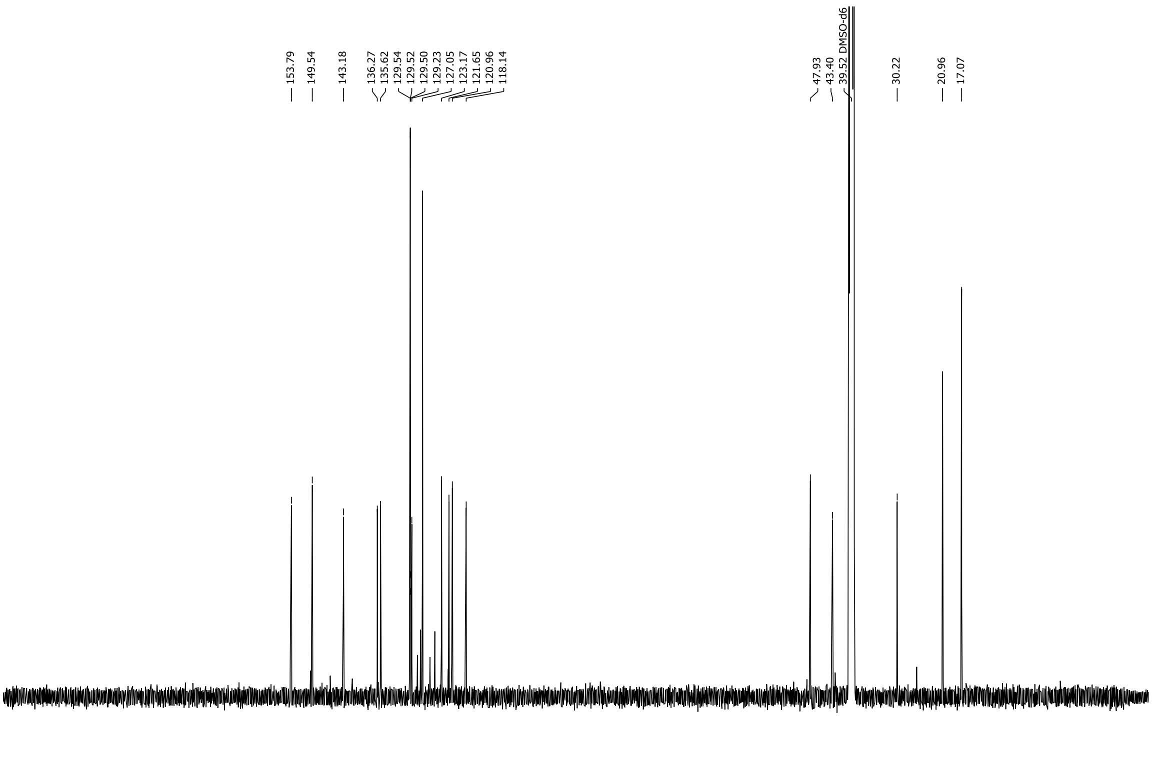

*10.3.12. N'-(3-(6-Methoxy-2-oxo-1,4-dihydroquinazolin-3(2H)-yl)propylidene)-4-methylbenzenesulfonohydrazide (**4l**)*

^{1}H NMR (500 MHz, DMSO-d_6):

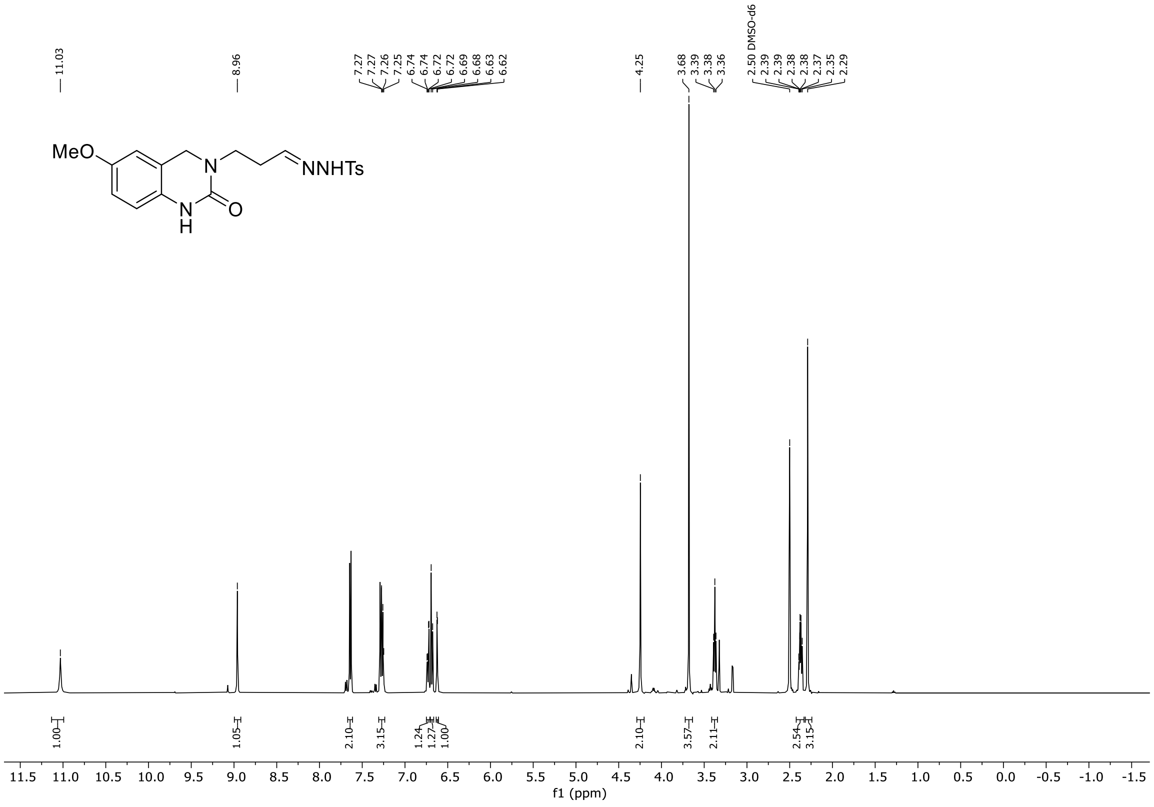

^{13}C NMR (126 MHz, DMSO-d_6):

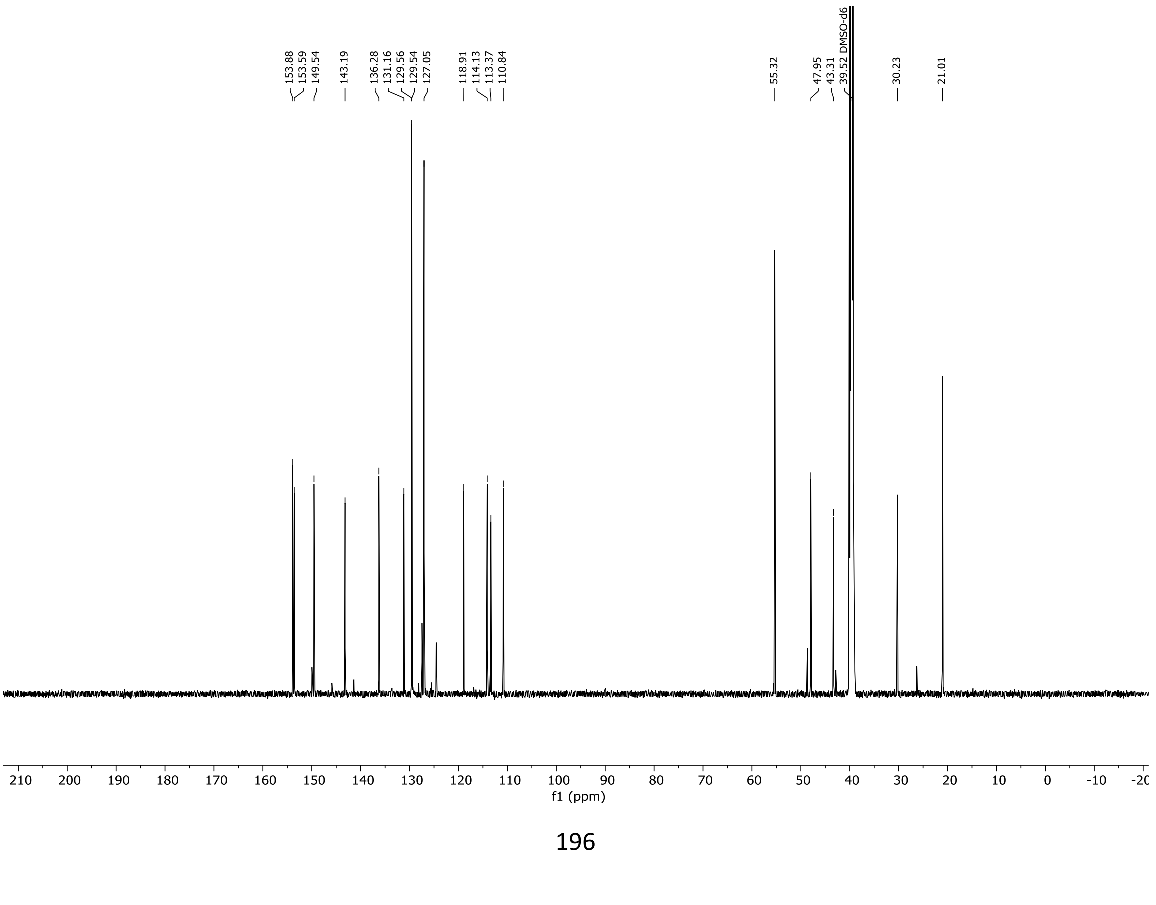

*10.3.13. N'-(3-(8-Methoxy-2-oxo-1,4-dihydroquinazolin-3(2H)-yl)propylidene)-4-methylbenzenesulfonohydrazide (**4m**)*

^{1}H NMR (500 MHz, DMSO-d_6):

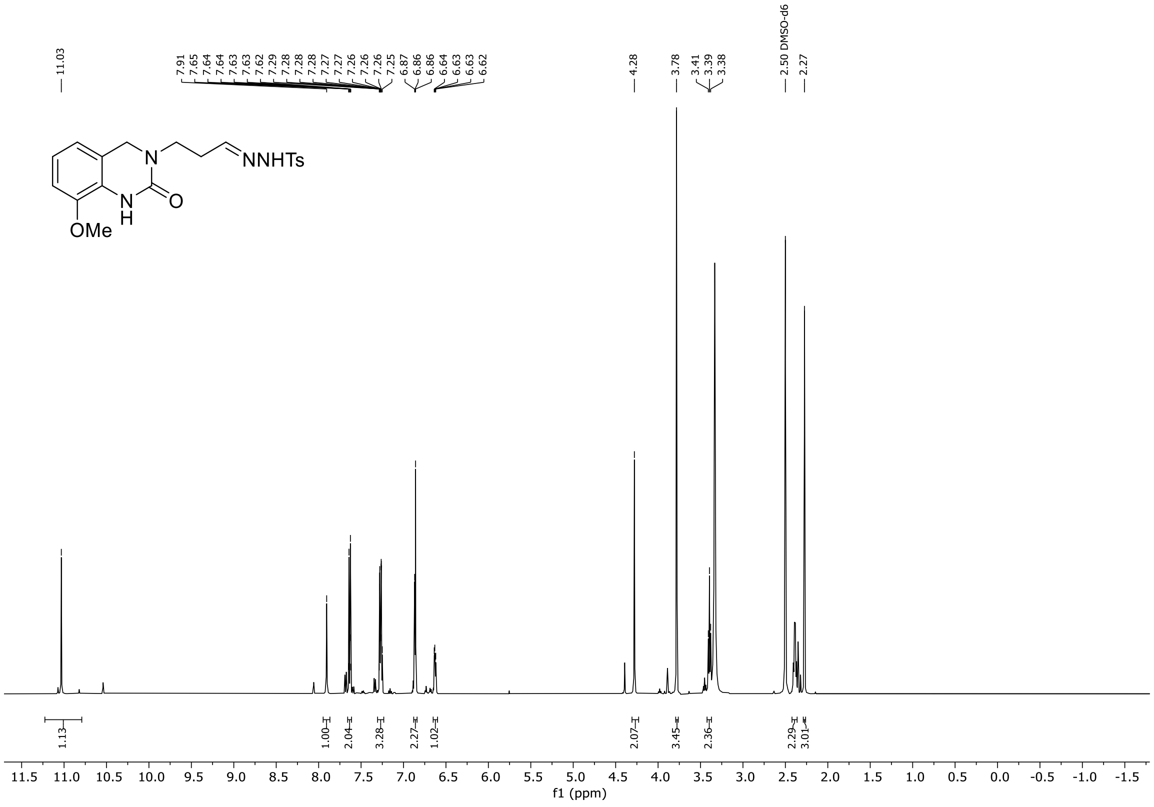

^{13}C NMR (126 MHz, DMSO-d_6):

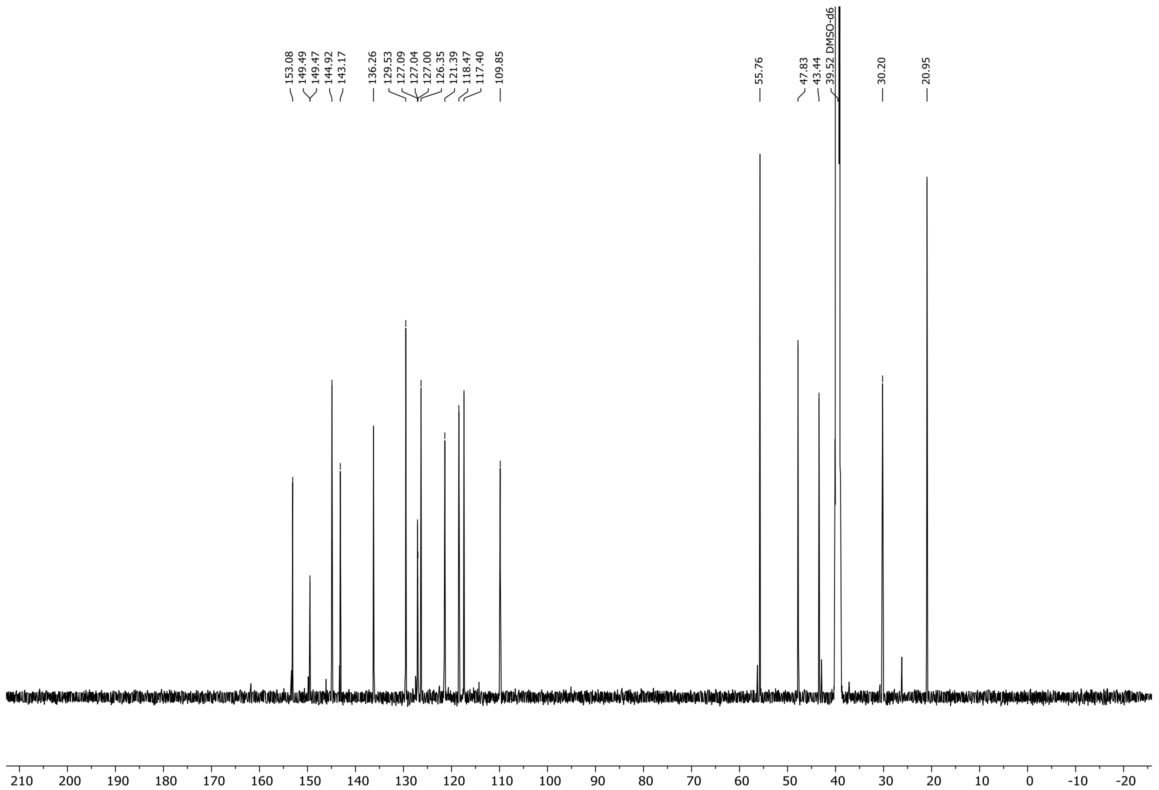

*10.3.14. N'-(3-(7-(Furan-3-yl)-2-oxo-1,4-dihydroquinazolin-3(2H)-yl)propylidene)-4-methylbenzenesulfonohydrazide (**4n**)*

^{1}H NMR (500 MHz, DMSO-d_6):

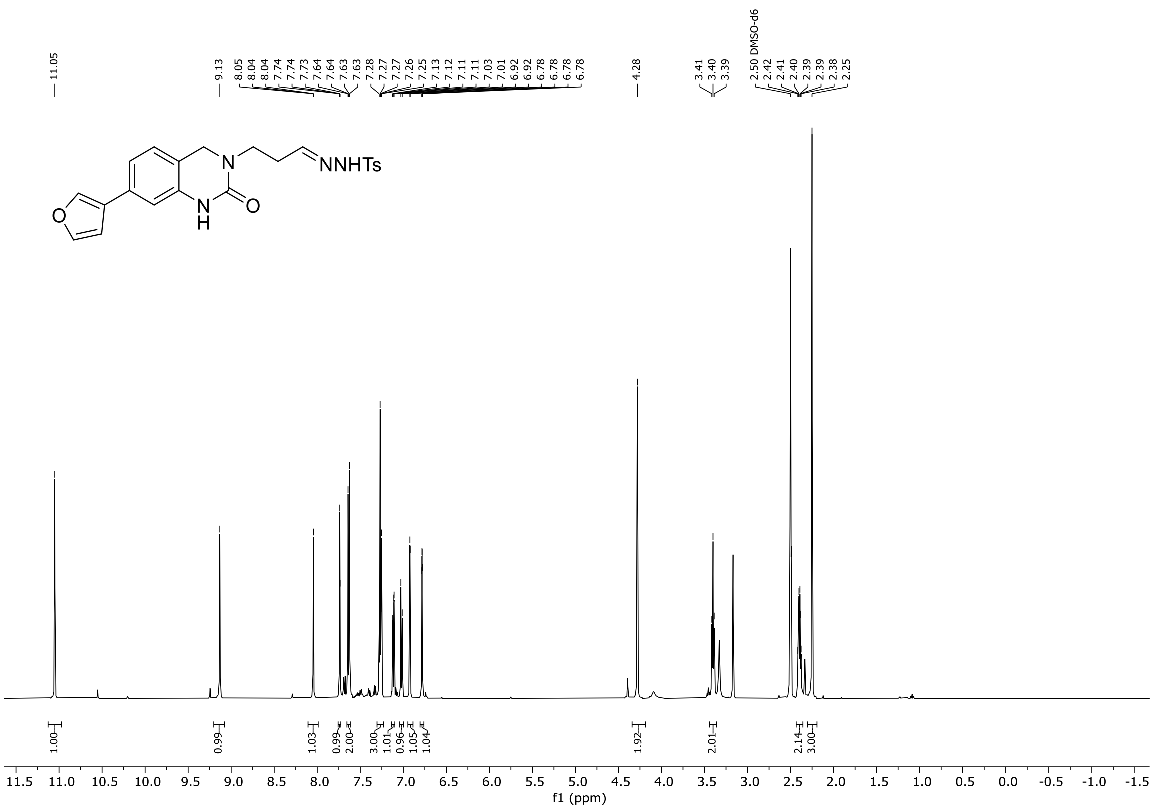

^{13}C NMR (126 MHz, DMSO-d_6):

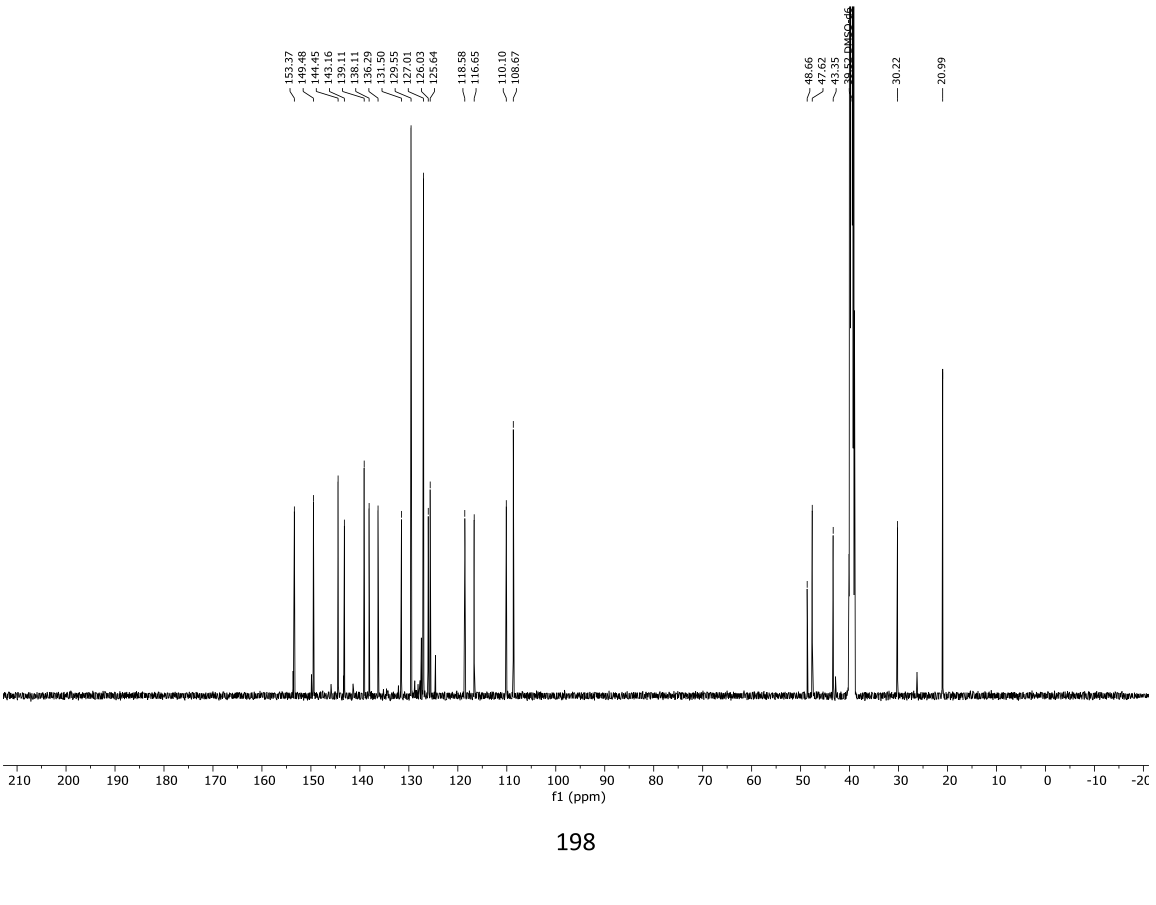

*10.3.15. N'-(3-(2-Oxo-7-(thiophen-3-yl)-1,4-dihydroquinazolin-3(2H)-yl)propylidene)-4-methylbenzenesulfonohydrazide (**4o**)*

^{1}H NMR (500 MHz, DMSO-d_6):

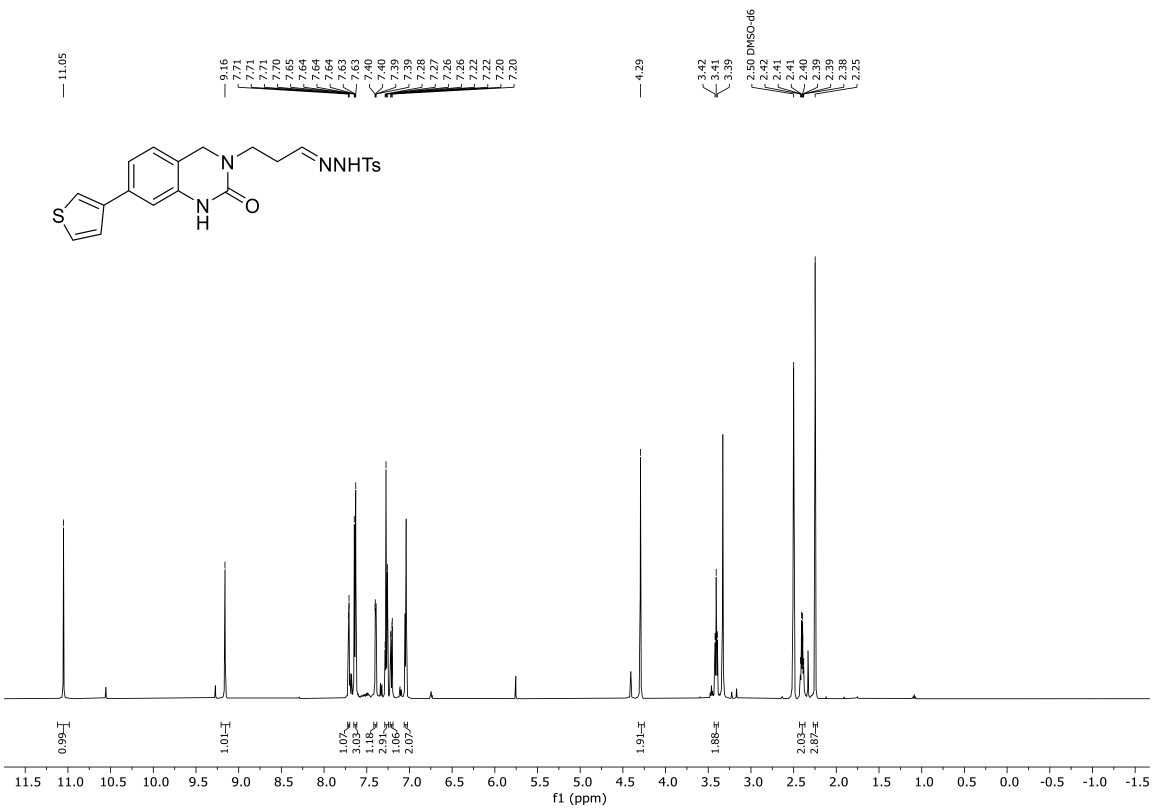

^{13}C NMR (126 MHz, DMSO-d_6):

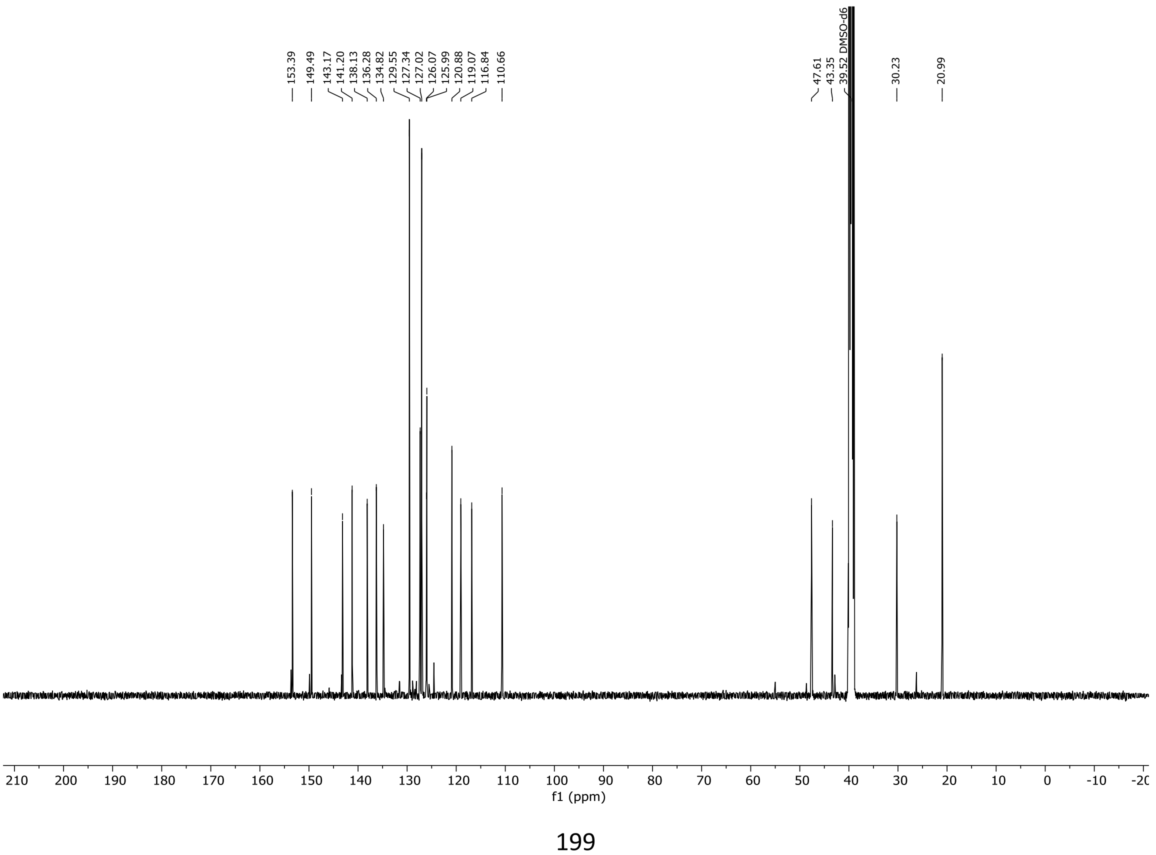

*10.3.16. tert-Butyl 2-(2-oxo-3-(3-(2-tosylhydrazineylidene)propyl)-1,2,3,4-tetrahydroquinazolin-7-yl)-1H-pyrrole-1-carboxylate (**4p**)*

^{1}H NMR (500 MHz, DMSO-d_6):

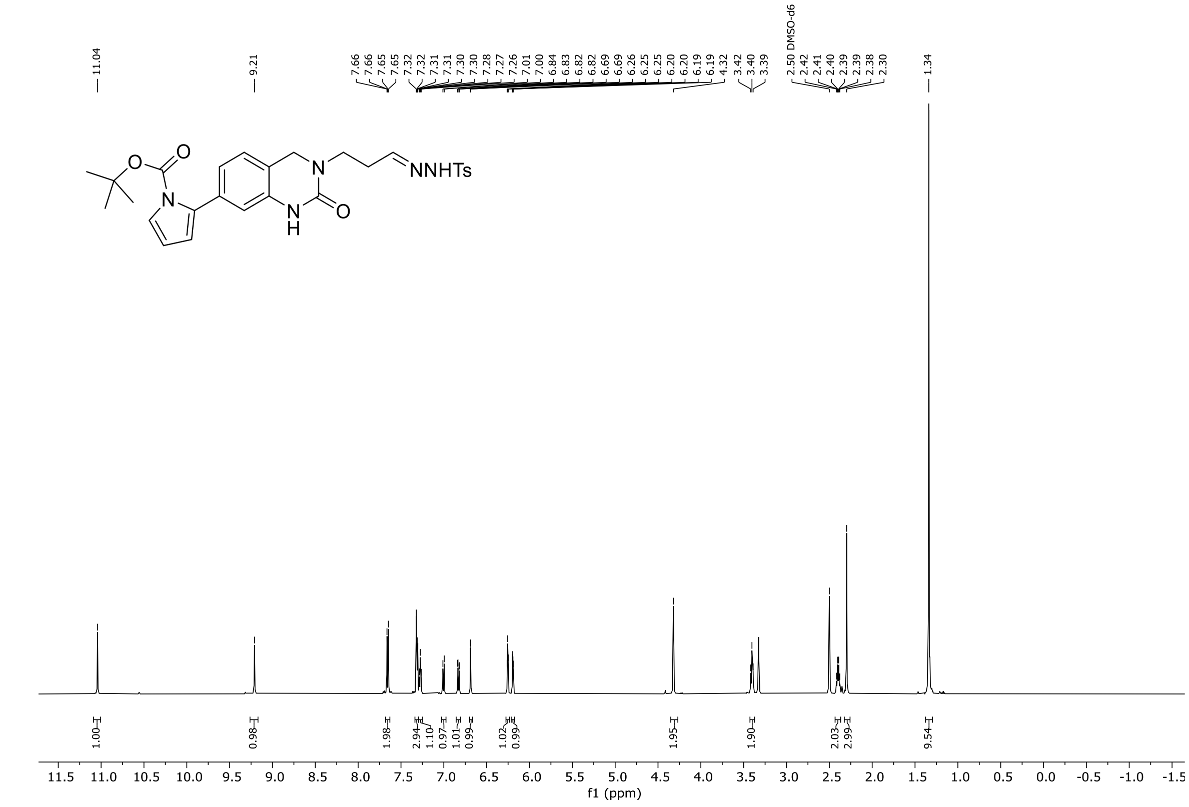

^{13}C NMR (126 MHz, DMSO-d_6):

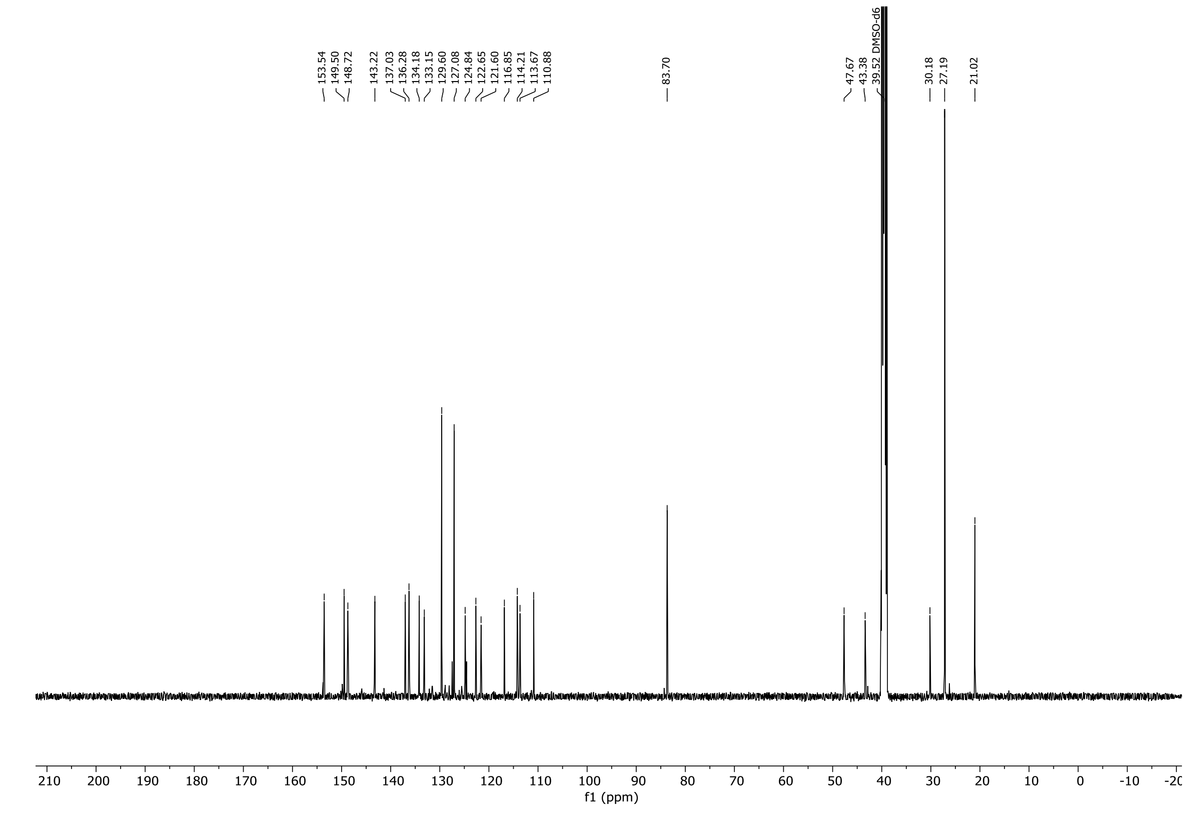

*10.3.17. N'-(3-(2-Oxo-7-(pyridin-3-yl)-1,4-dihydroquinazolin-3(2H)-yl)propylidene)-4-methylbenzenesulfonohydrazide (**4q**)*

^{1}H NMR (500 MHz, DMSO-d_6):

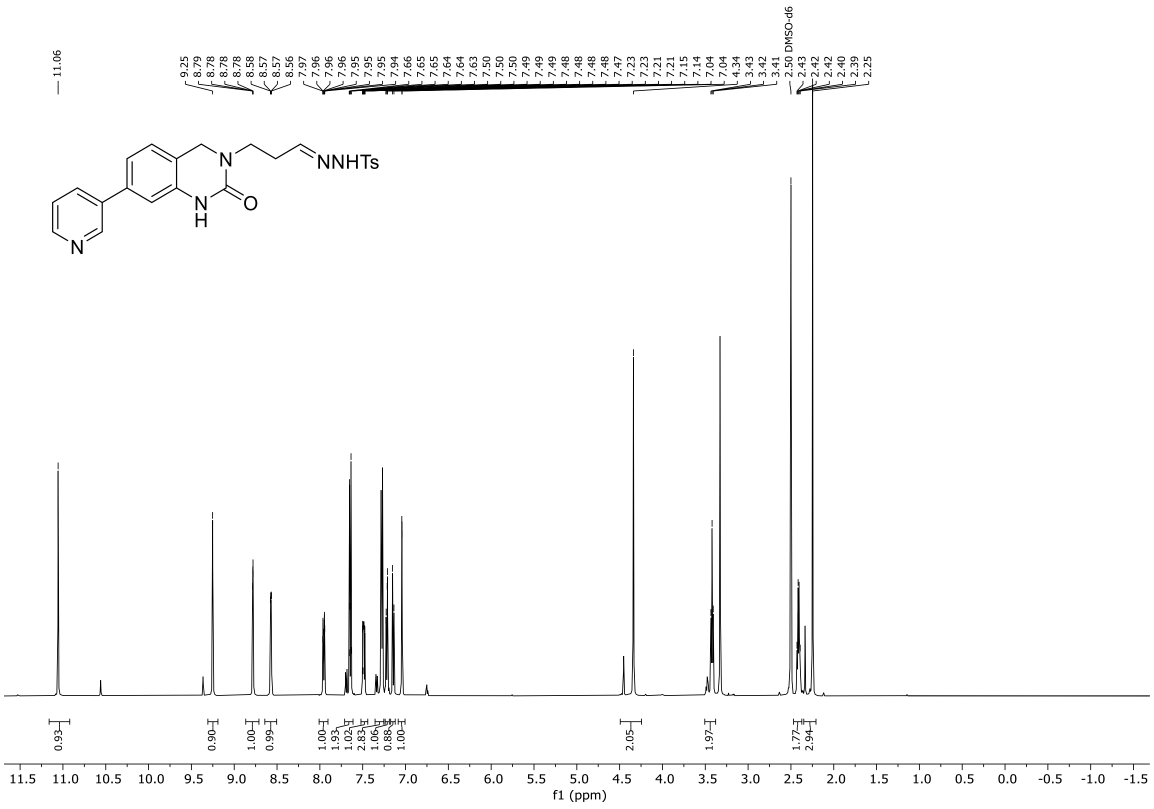

^{13}C NMR (126 MHz, DMSO-d_6):

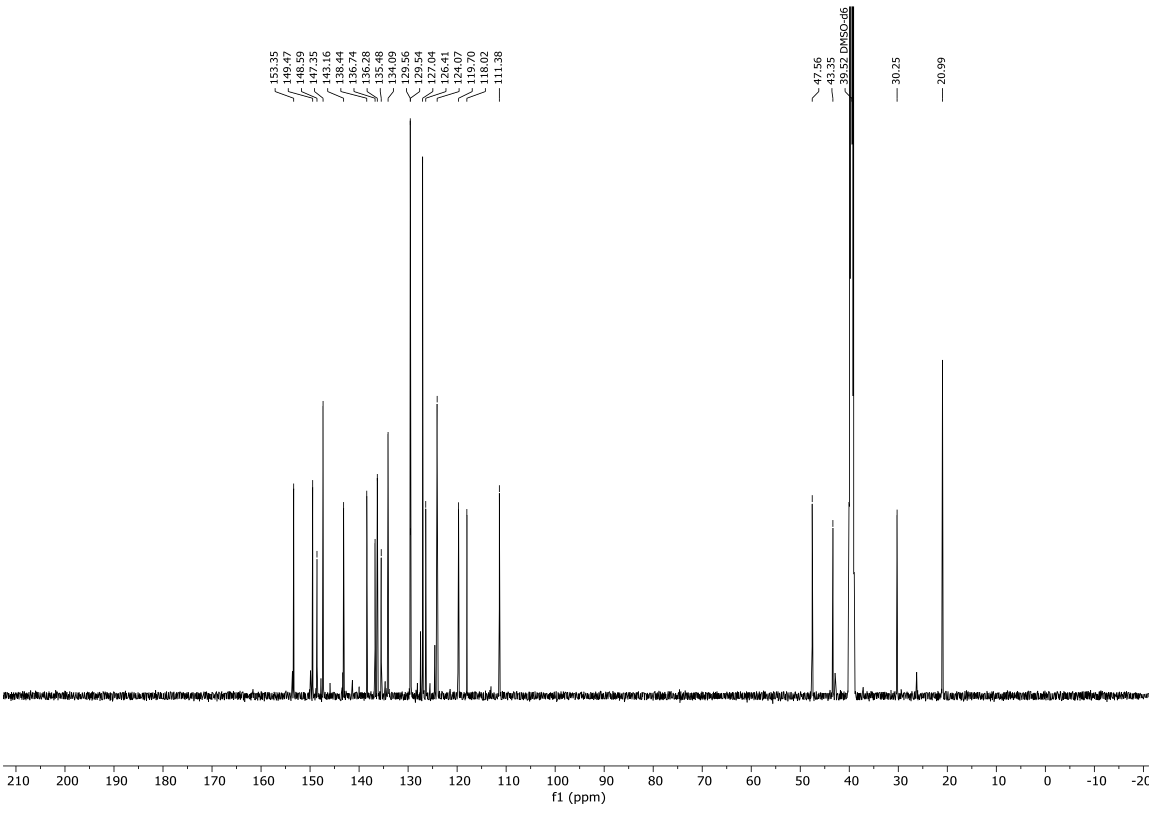

*10.3.18. Ethyl (E)-3-(2-oxo-3-(3-(2-tosylhydrazineylidene)propyl)-1,2,3,4-tetrahydroquinazolin-7-yl)acrylate **(4r)***

^{1}H NMR (500 MHz, DMSO-d_6):

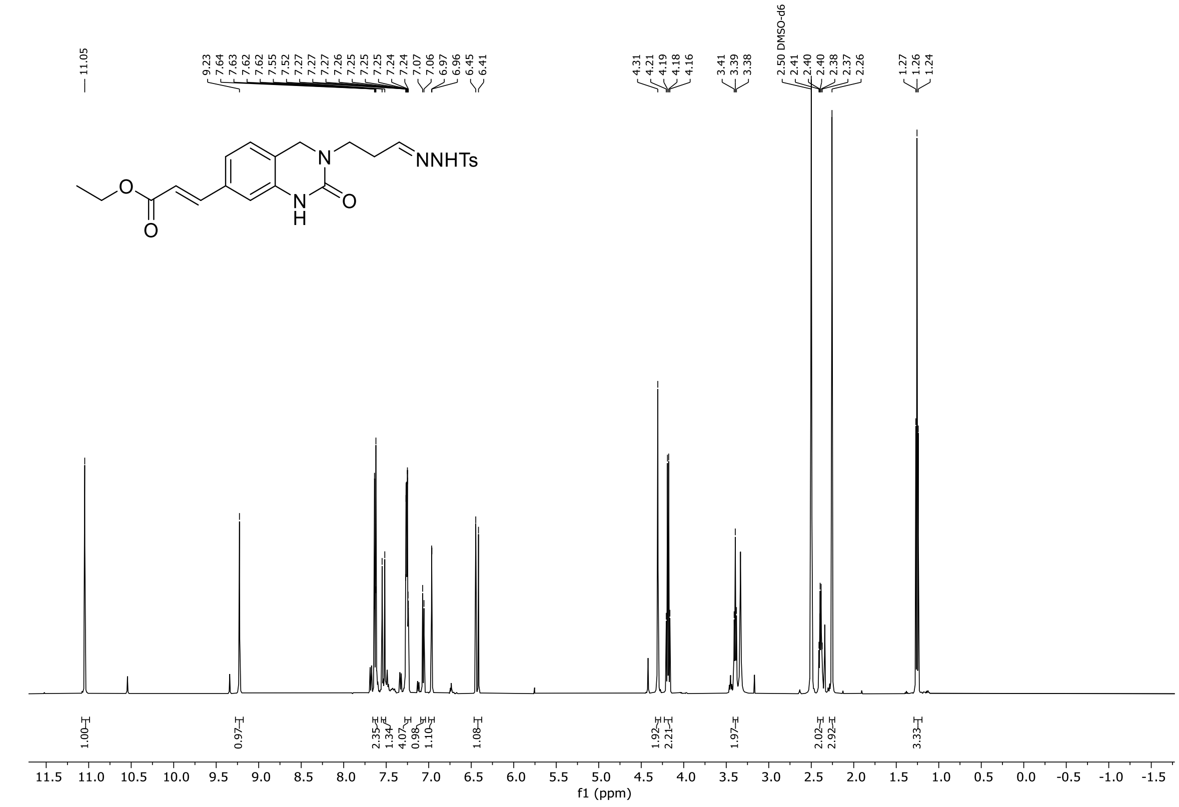

^{13}C NMR (126 MHz, DMSO-d_6):

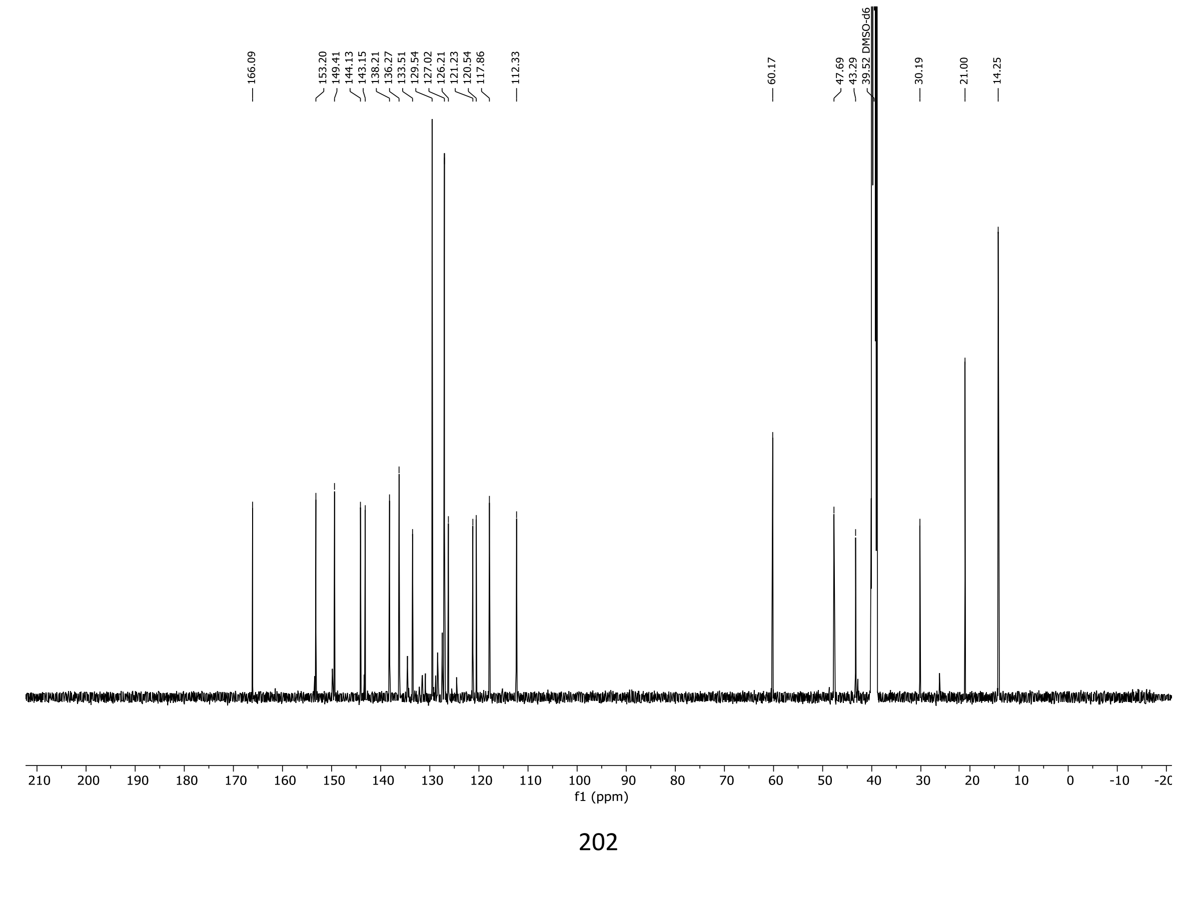

*10.3.19. N'-(3-(6-(4-Methoxyphenyl)-2-oxo-1,4-dihydroquinazolin-3(2H)-yl)propylidene)-4-methylbenzenesulfonohydrazide (**4s**)*

^{1}H NMR (500 MHz, DMSO-d_6):

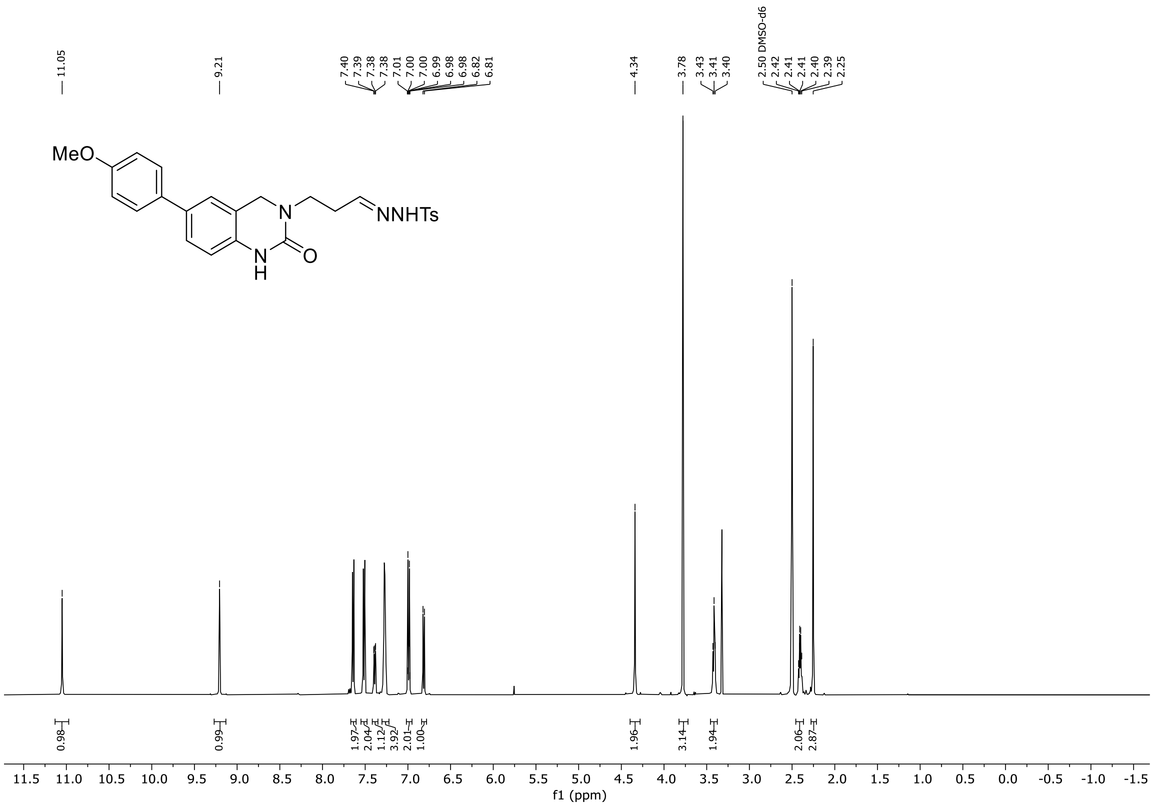

^{13}C NMR (126 MHz, DMSO-d_6):

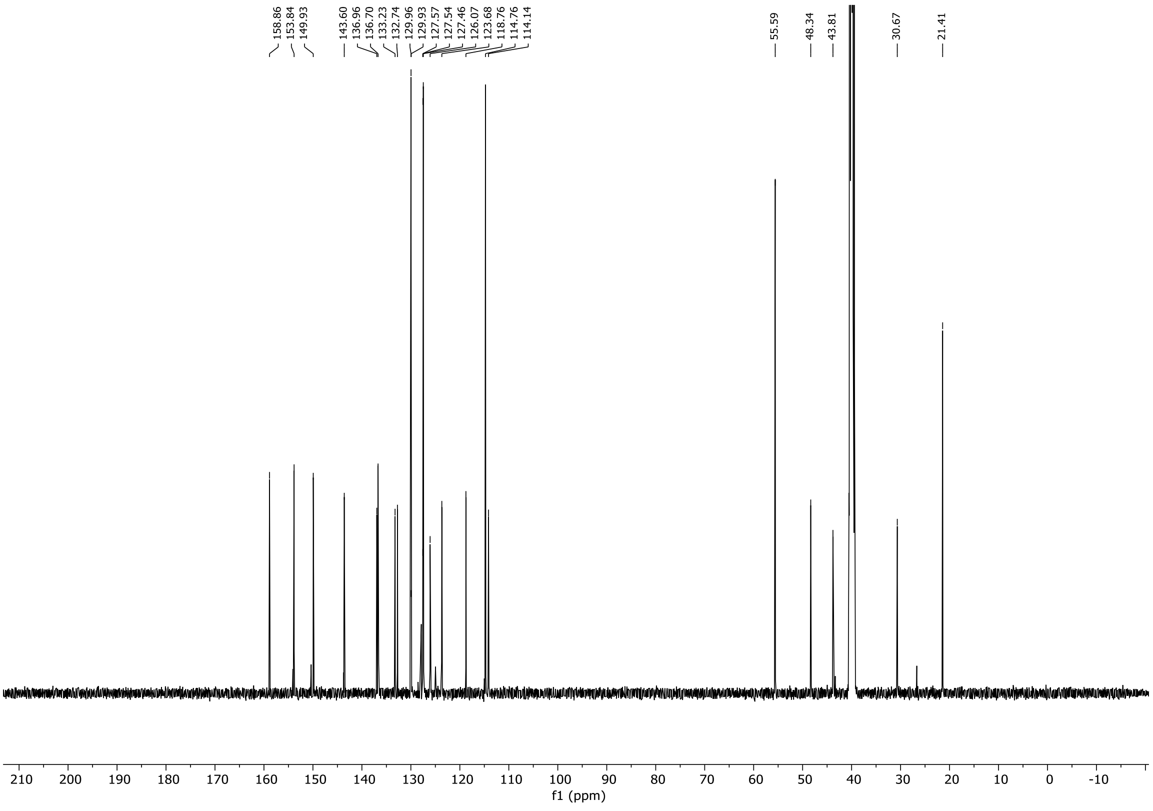

*10.3.20. N'-(3-(2-Oxo-1,4-dihydropyrido[3,2-d]pyrimidin-3(2H)-yl)propylidene)-4-methylbenzenesulfonohydrazide (**4t**)*

^{1}H NMR (500 MHz, DMSO-d_6):

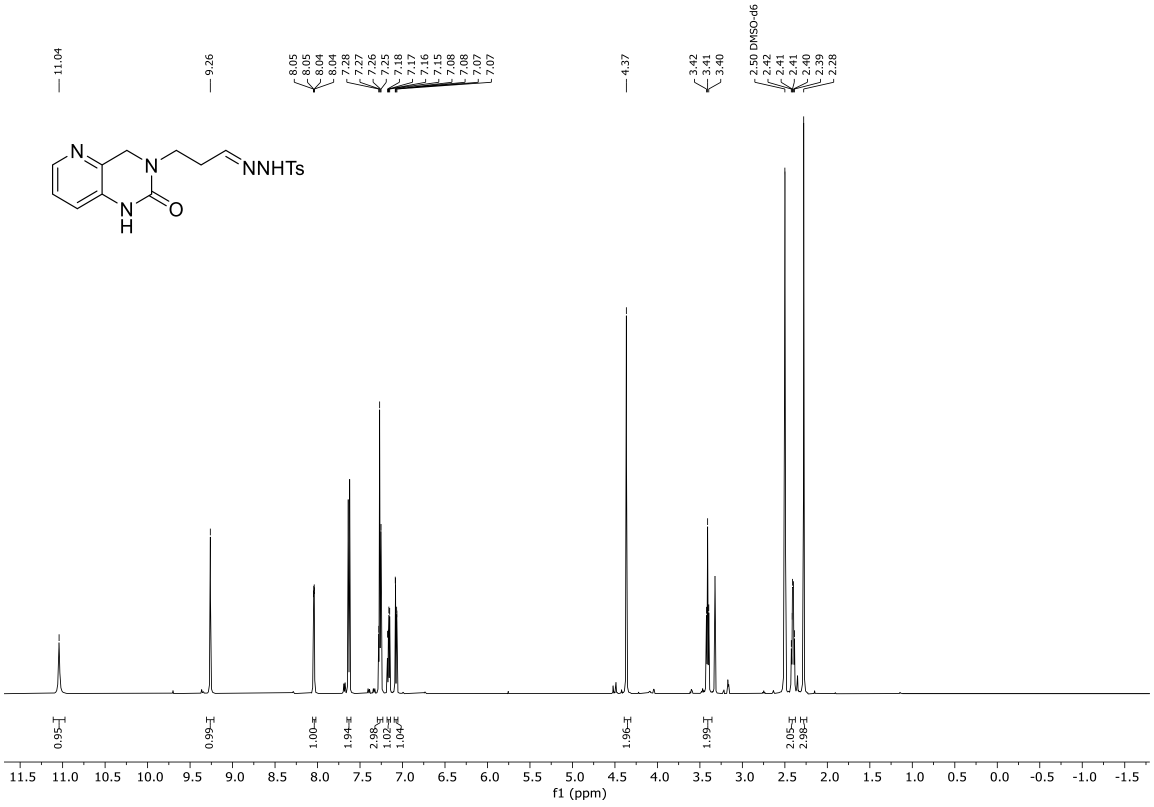

^{13}C NMR (126 MHz, DMSO-d_6):

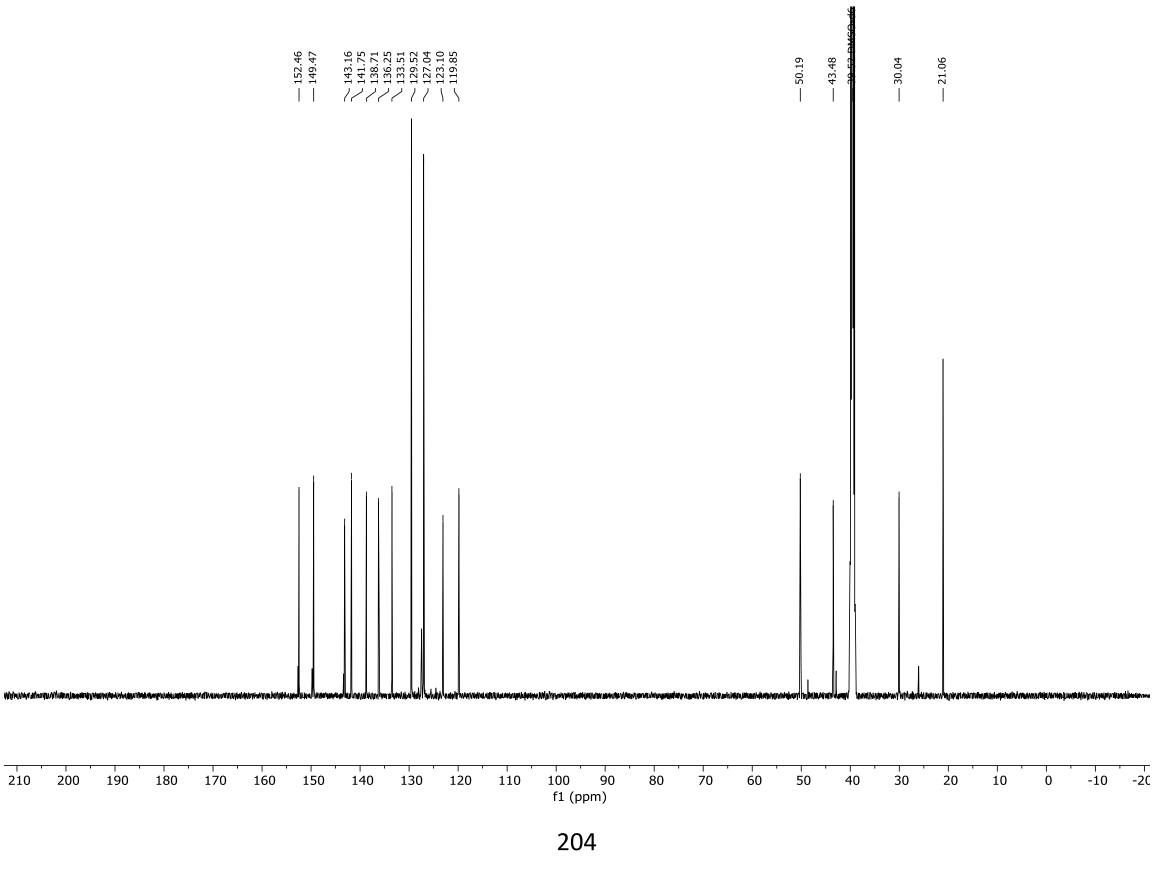

*10.3.21. N'-(2,2-Dimethyl-3-(2-oxo-1,4-dihydroquinazolin-3(2H)-yl)propylidene)-4-methylbenzenesulfonohydrazide (**4u**)*

^{1}H NMR (500 MHz, DMSO-d_6):

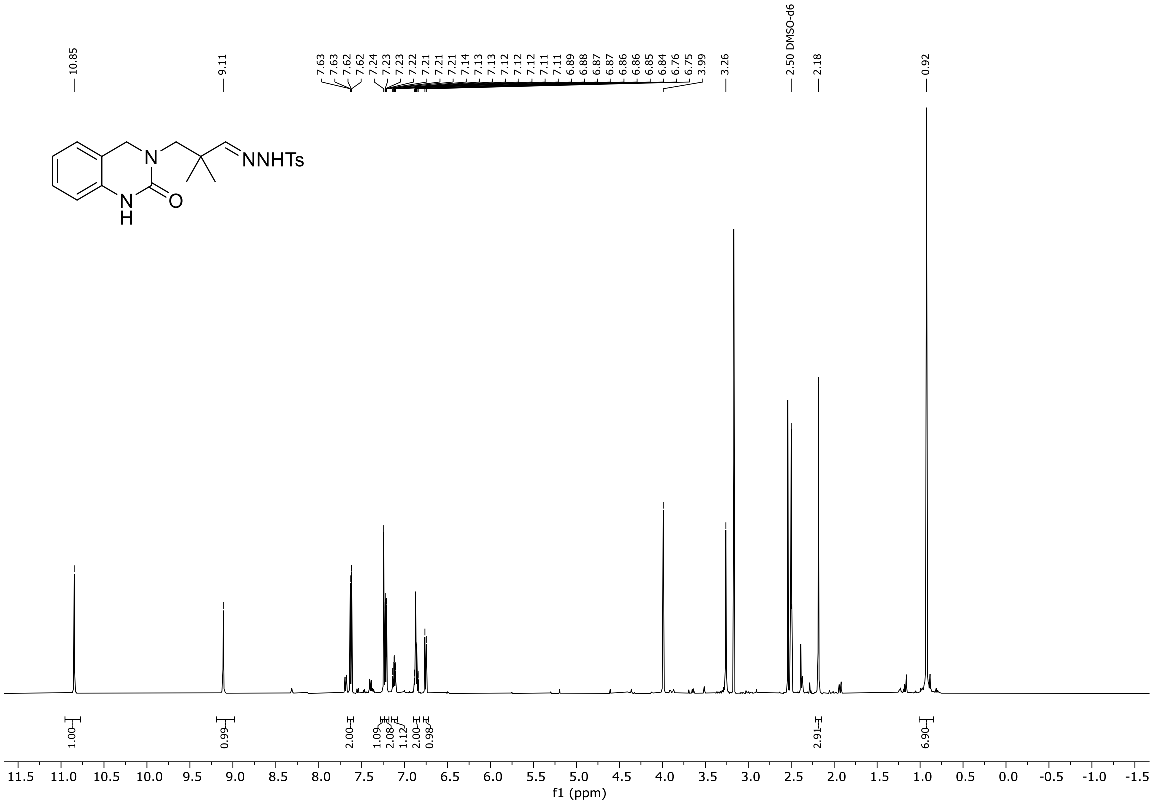

^{13}C NMR (126 MHz, DMSO-d_6):

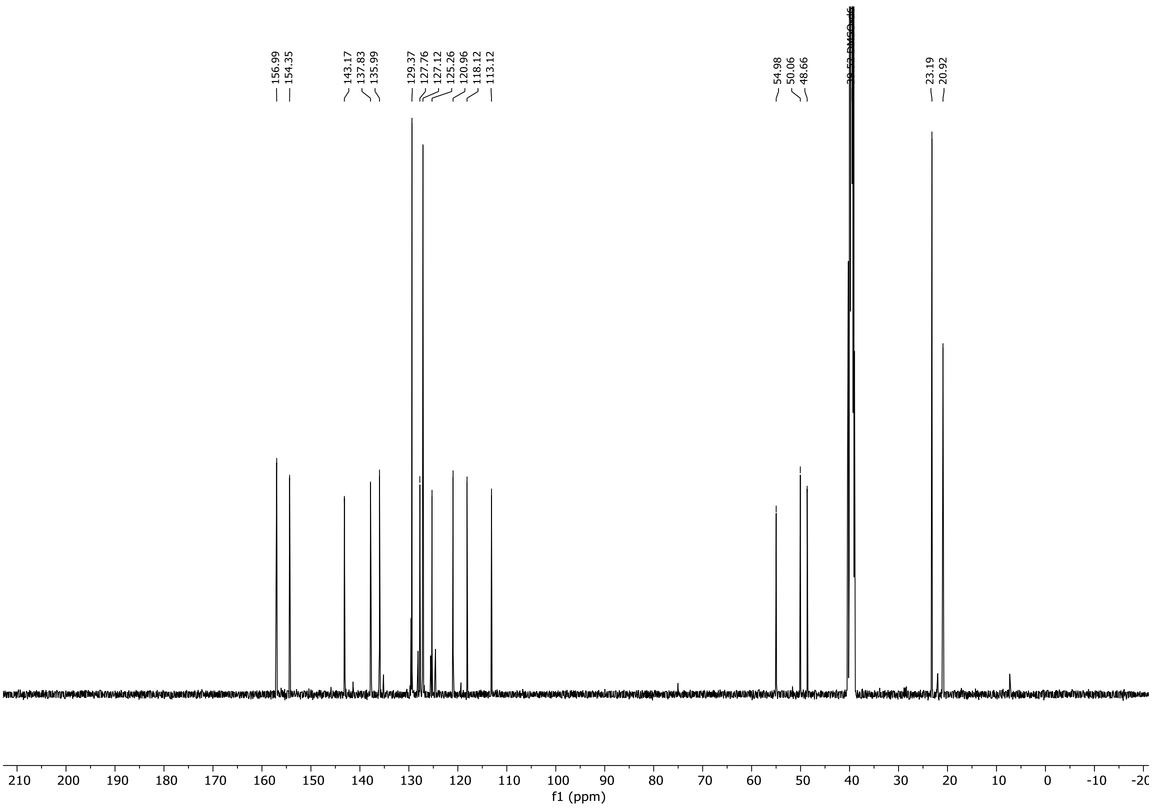

*10.3.22. N'-(3-(2-Oxo-1,4-dihydropyrido[2,3-d]pyrimidin-3(2H)-yl)propylidene)-4-methylbenzenesulfonohydrazide (**4v**)*

^{1}H NMR (500 MHz, DMSO-d_6):

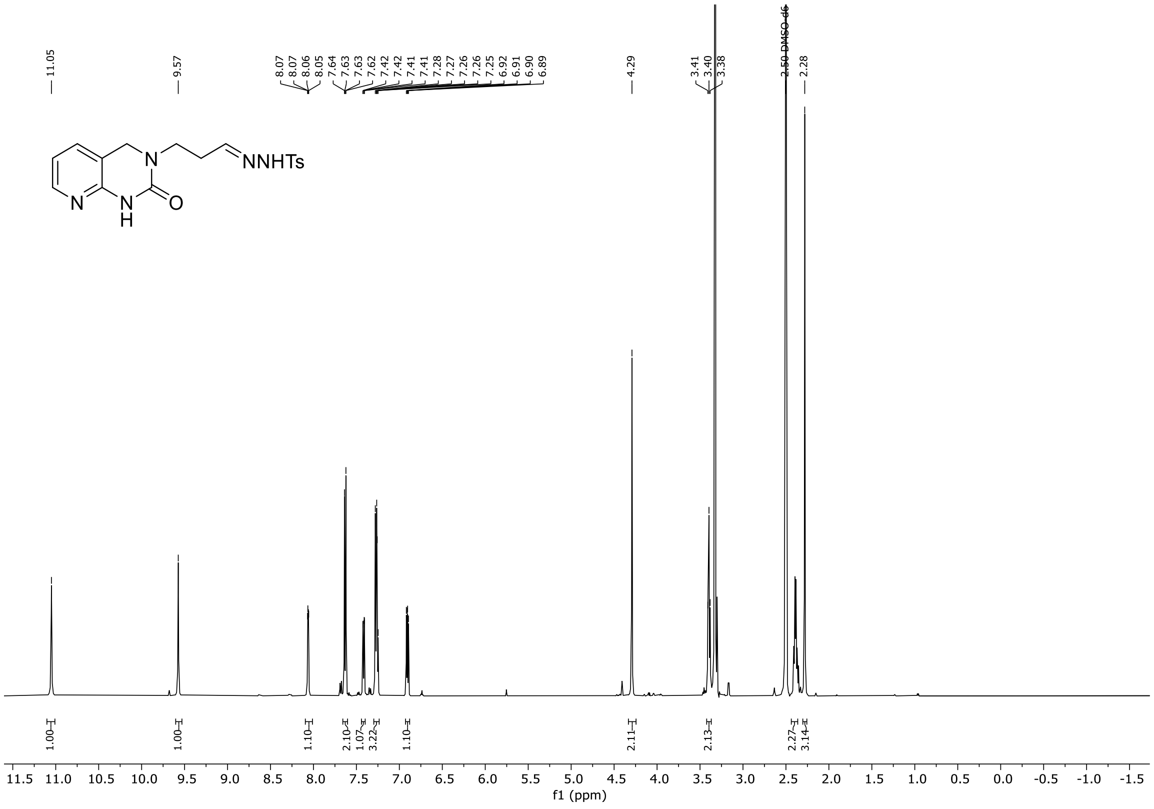

^{13}C NMR (126 MHz, DMSO-d_6):

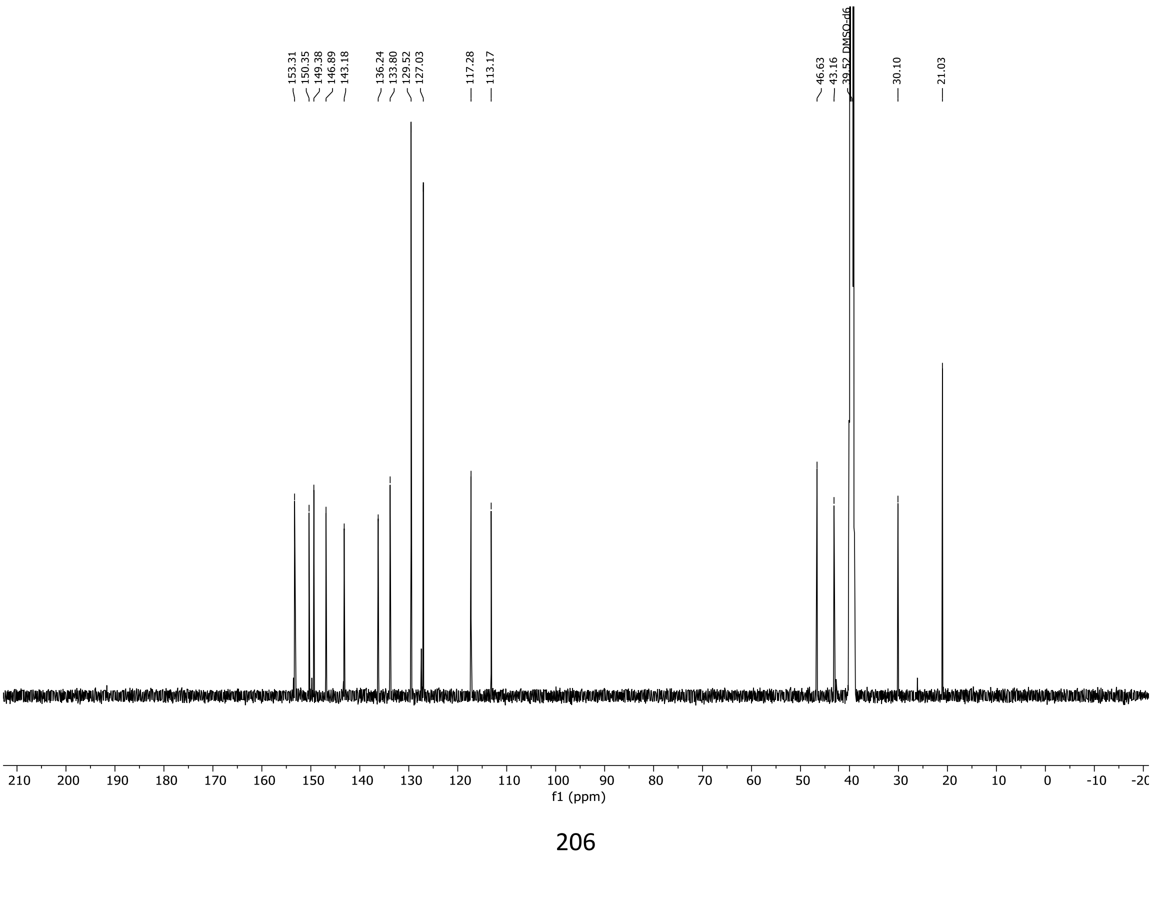

*10.3.23. N'-(2-(2-Oxo-1,4-dihydroquinazolin-3(2H)-yl)benzylidene)-4-methylbenzenesulfonohydrazide (**4w**)*

^{1}H NMR (500 MHz, DMSO-d_6):

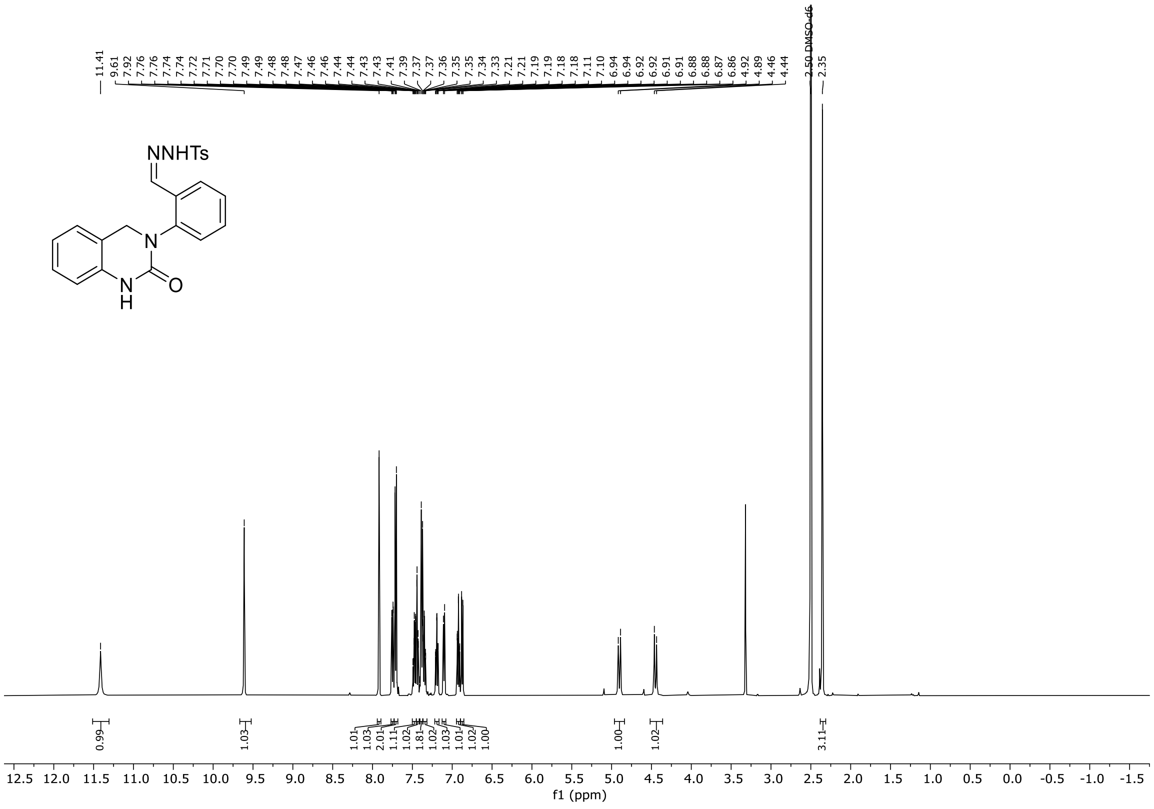

^{13}C NMR (126 MHz, DMSO-d_6):

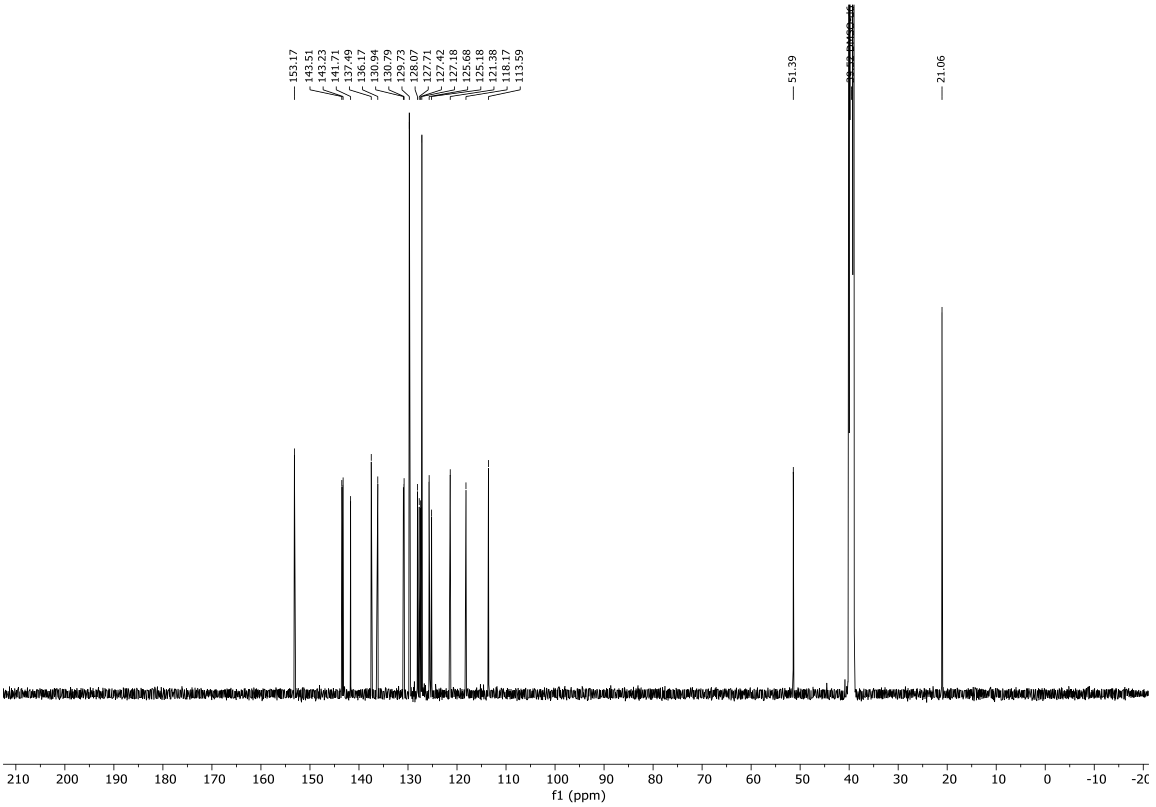

*10.3.24. N'-(4-(2-Oxo-1,4-dihydroquinazolin-3(2H)-yl)butylidene)-4-methylbenzenesulfonohydrazide (**4aa**)*

^{1}H NMR (500 MHz, DMSO-d_6):

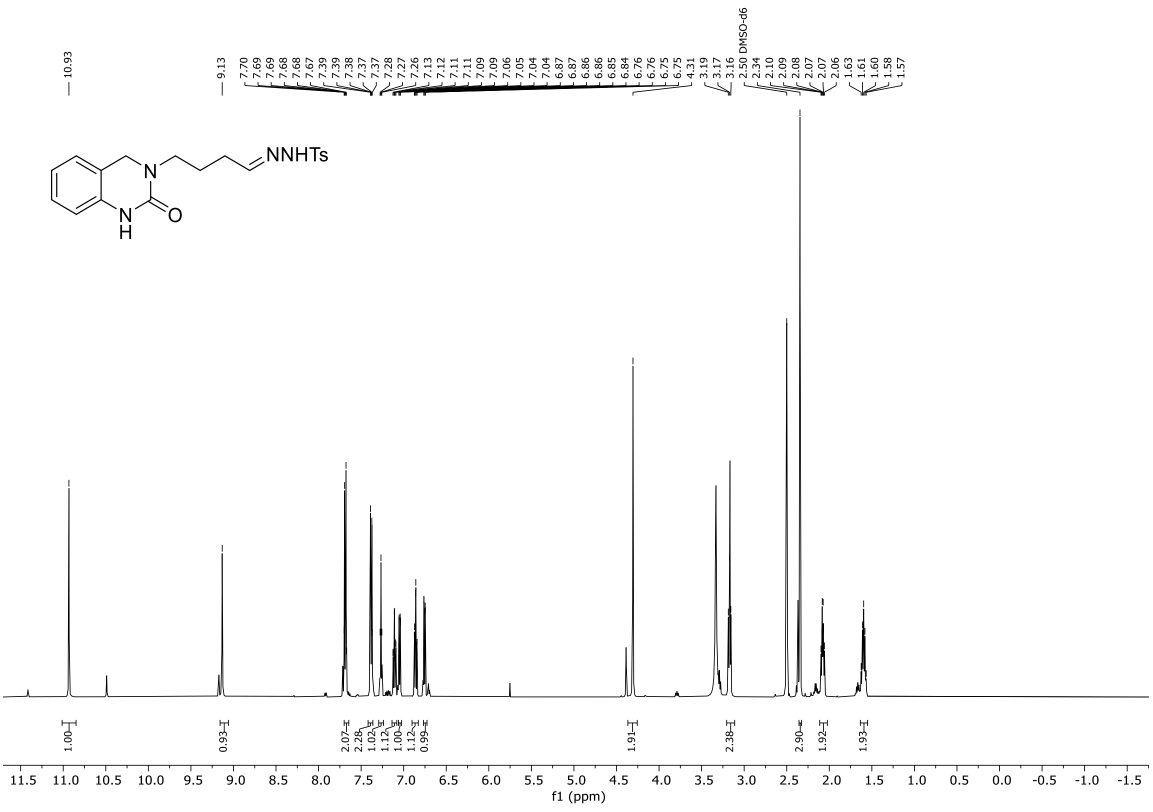

^{13}C NMR (126 MHz, DMSO-d_6):

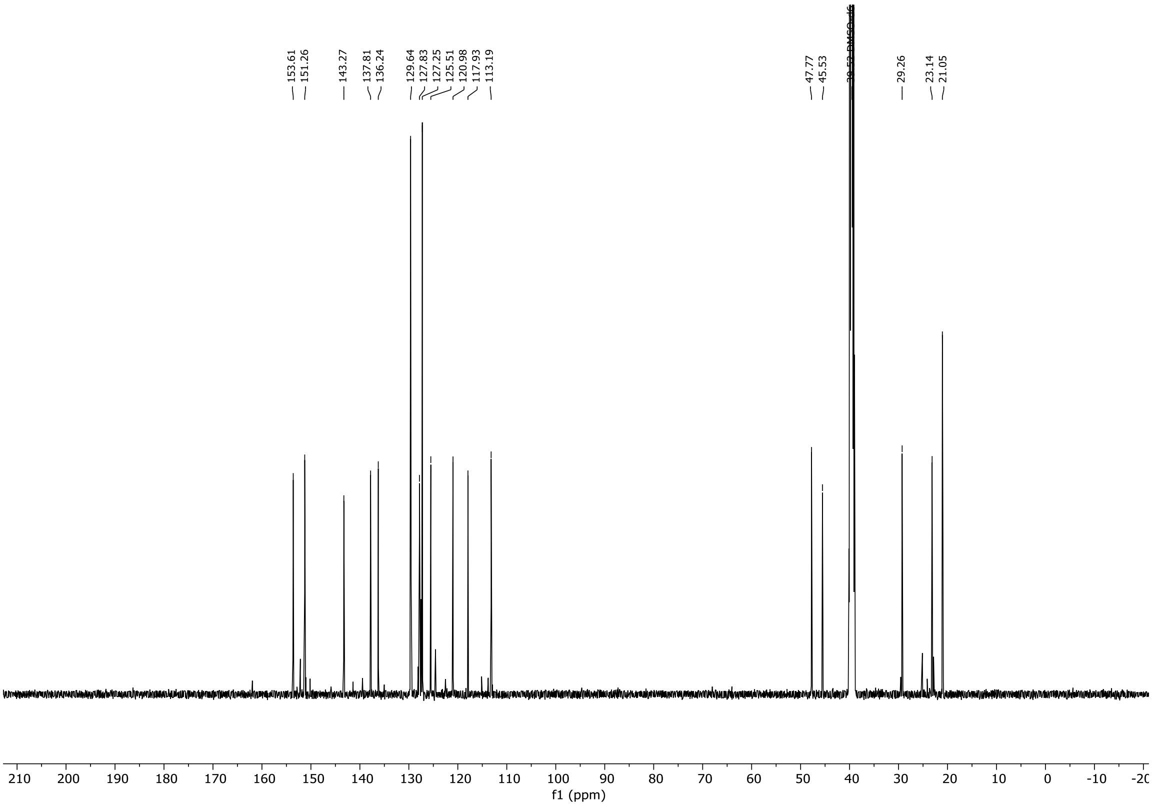

10.3.25. N'-(2-((2-Oxo-1,4-dihydroquinazolin-3(2H)-yl)methyl)benzylidene)-4-methylbenzenesulfonohydrazide ***(4ab)***

^{1}H NMR (500 MHz, DMSO-d_6):

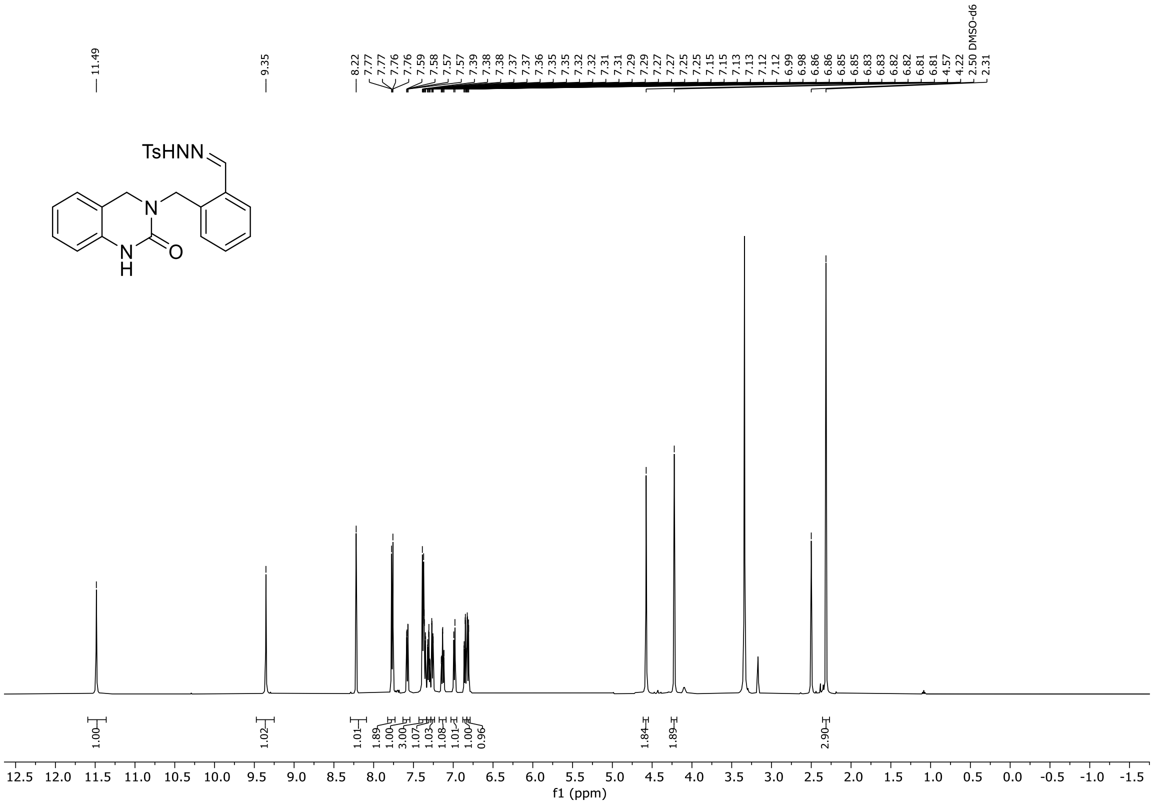

^{13}C NMR (126 MHz, DMSO-d_6):

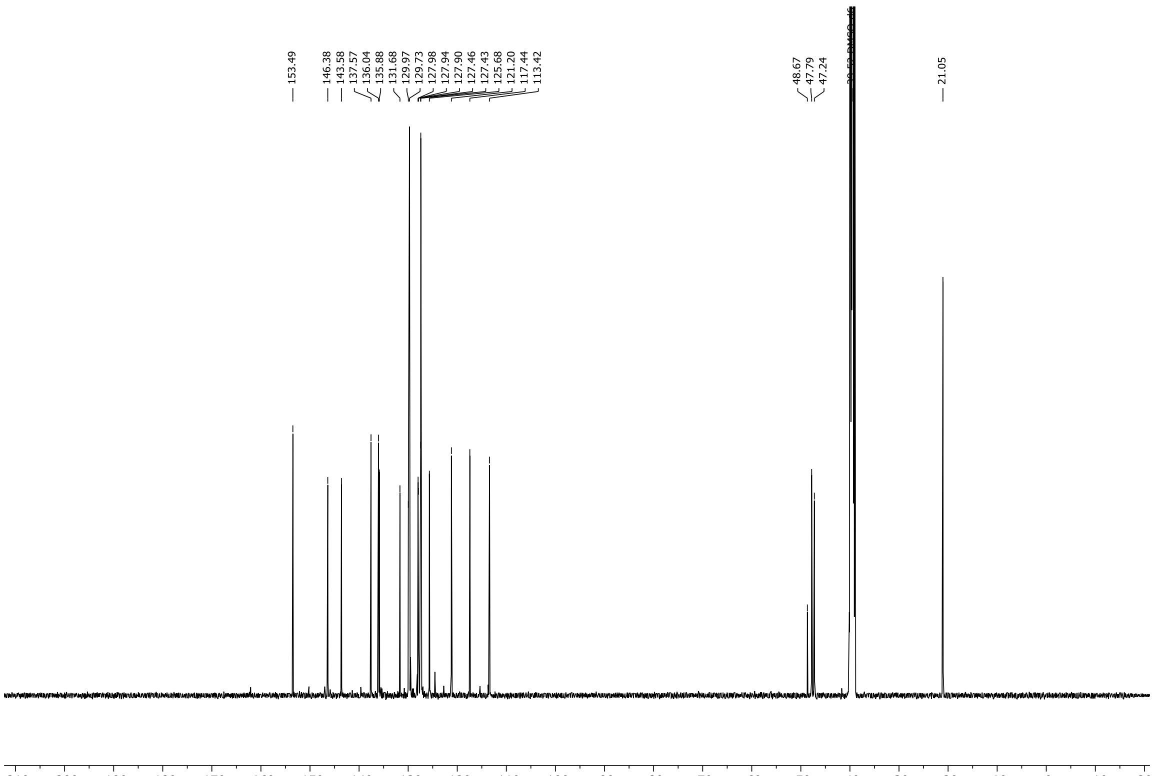

*10.3.26. N'-(3,3-Dimethyl-4-(2-oxo-1,4-dihydroquinazolin-3(2H)-yl)butylidene)-4-methylbenzenesulfonohydrazide (**4ac**)*

^{1}H NMR (500 MHz, DMSO-d_6):

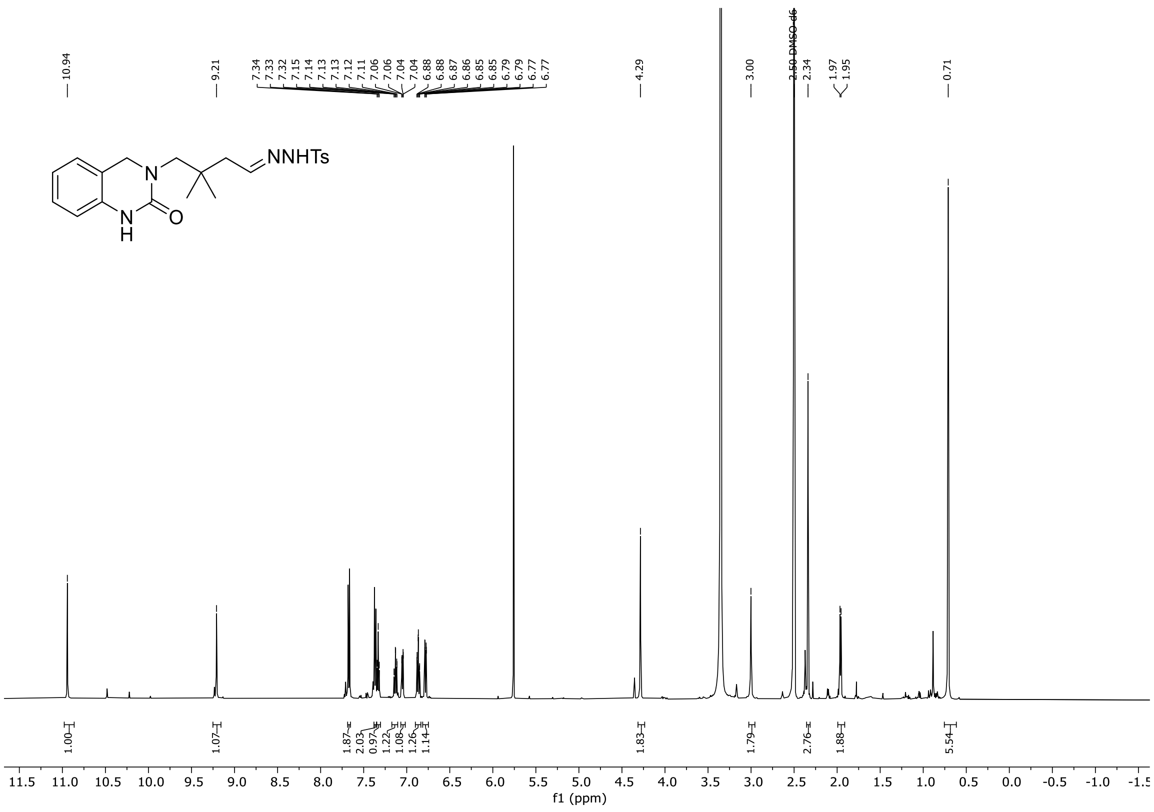

^{13}C NMR (126 MHz, DMSO-d_6):

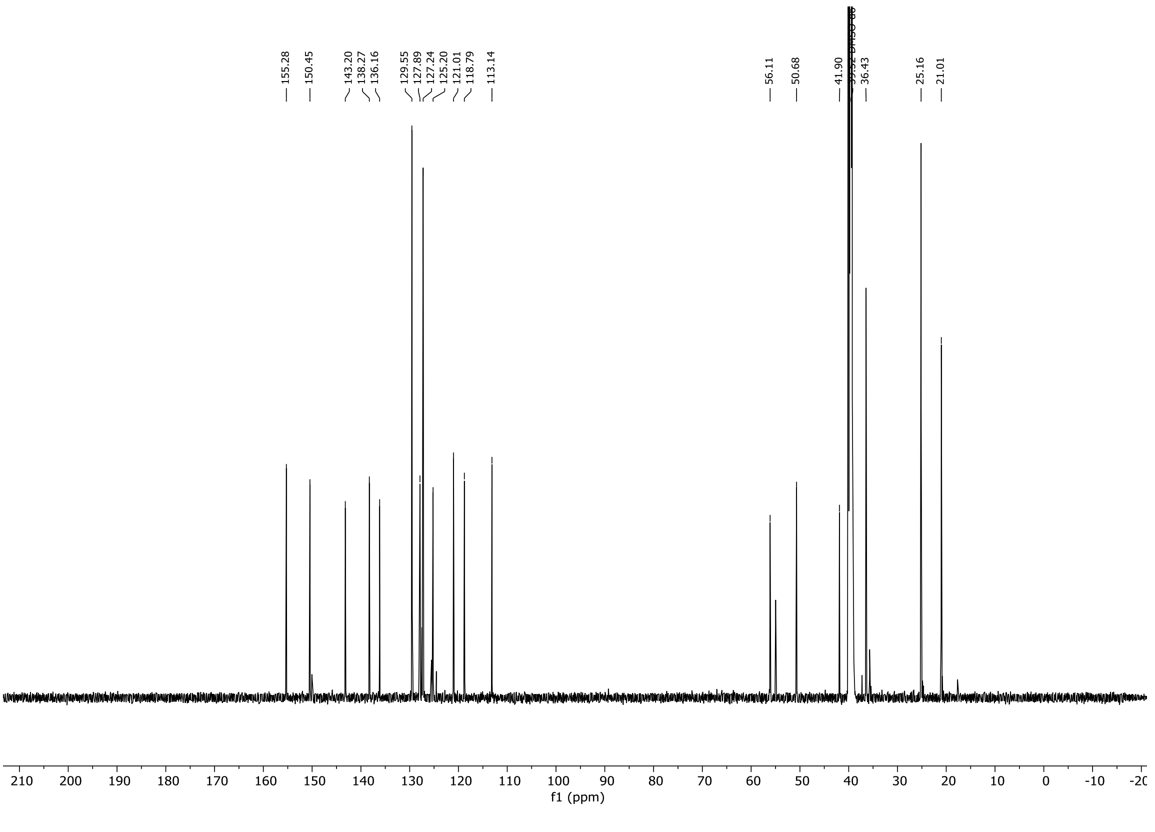

*10.3.27. N'-(2-(2-(2-Oxo-1,4-dihydroquinazolin-3(2H)-yl)ethyl)benzylidene)-4-methylbenzenesulfonohydrazide **(4ba)***

^{1}H NMR (500 MHz, DMSO-d_6):

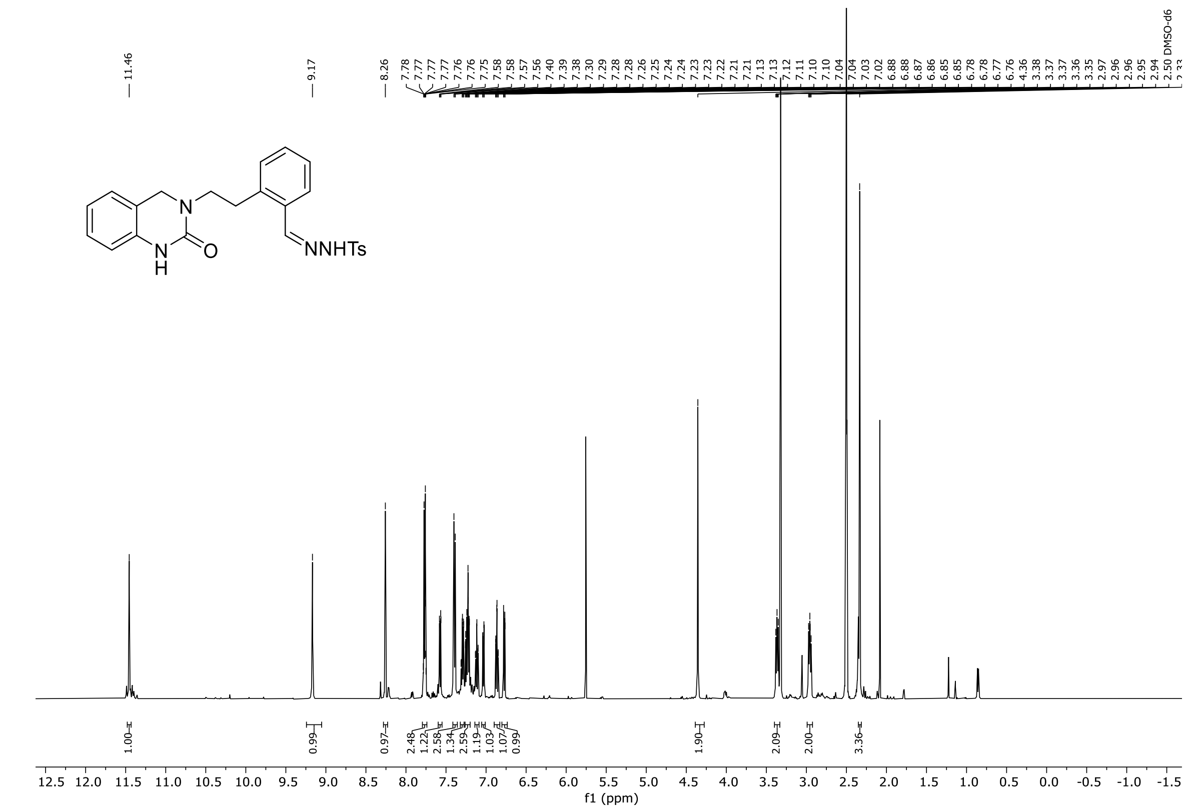

^{13}C NMR (126 MHz, DMSO-d_6):

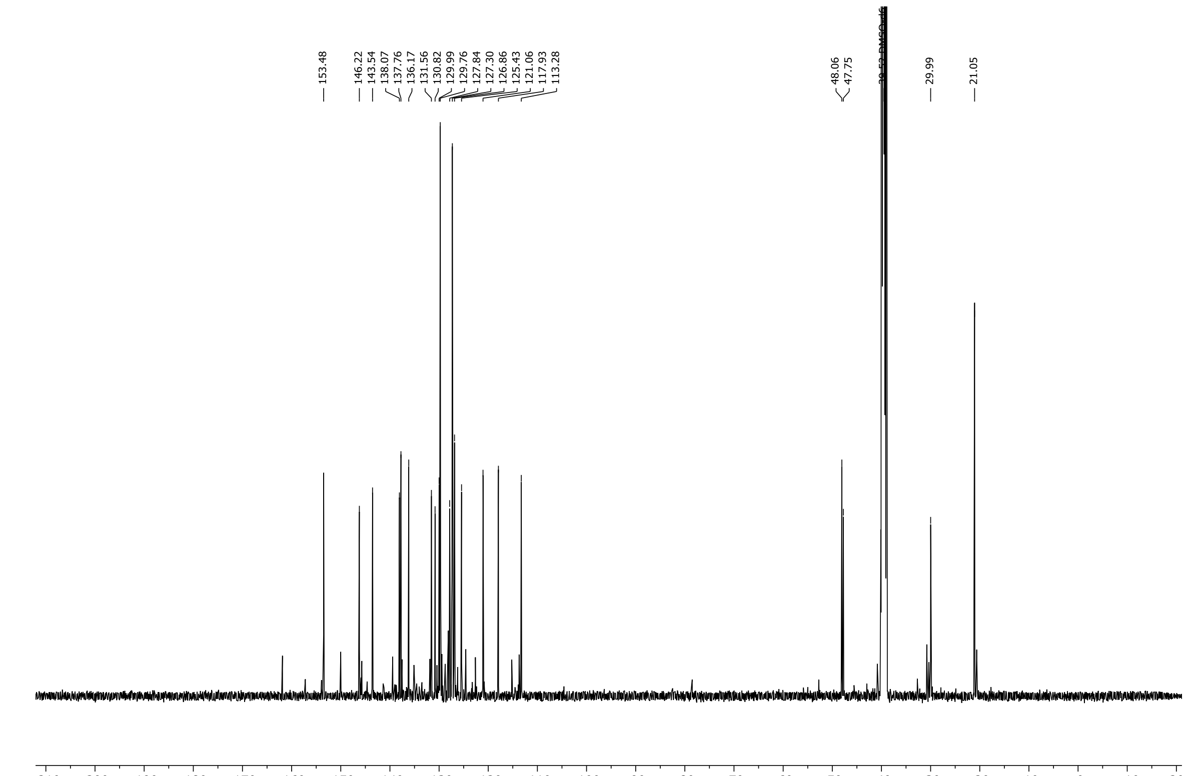

*10.3.28. N'-((6-(2-(2-Oxo-1,4-dihydroquinazolin-3(2H)-yl)ethyl)benzo[d][1,3]dioxol-5-yl)methylene)-4-methylbenzenesulfonohydrazide (**4bb**)*

^{1}H NMR (500 MHz, DMSO-d_6):

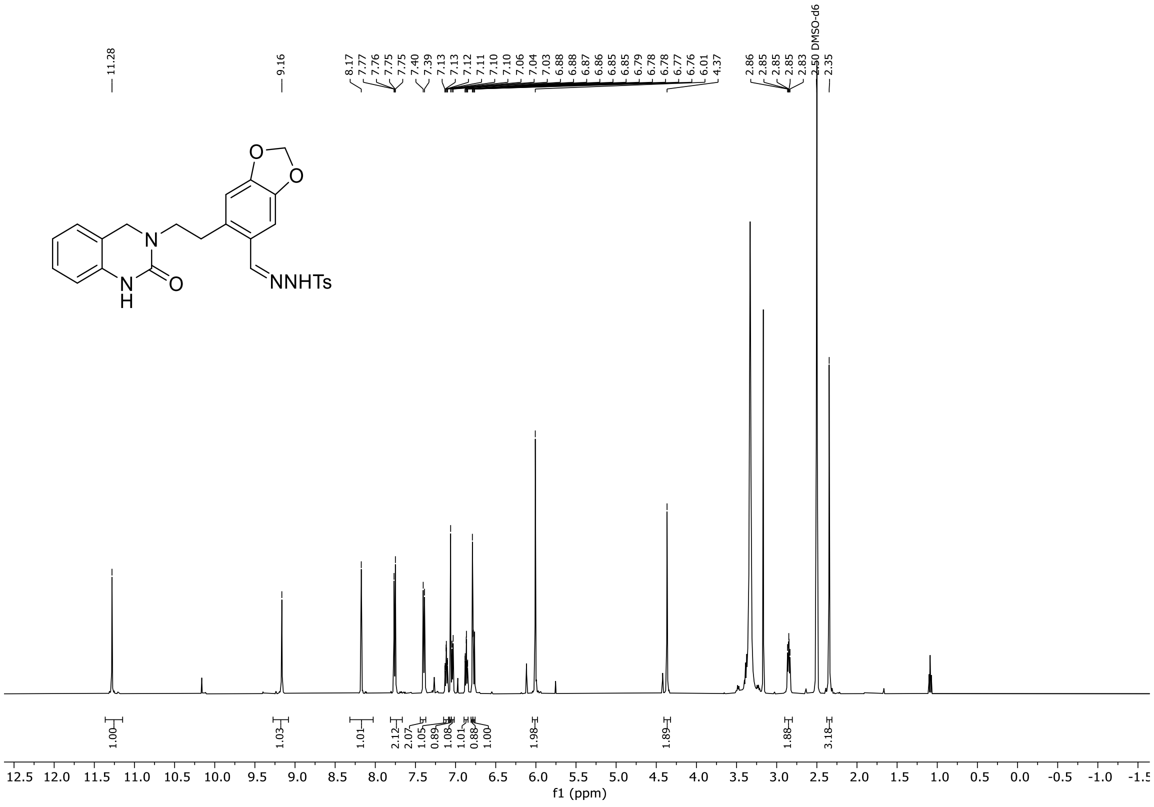

^{13}C NMR (126 MHz, DMSO-d_6):

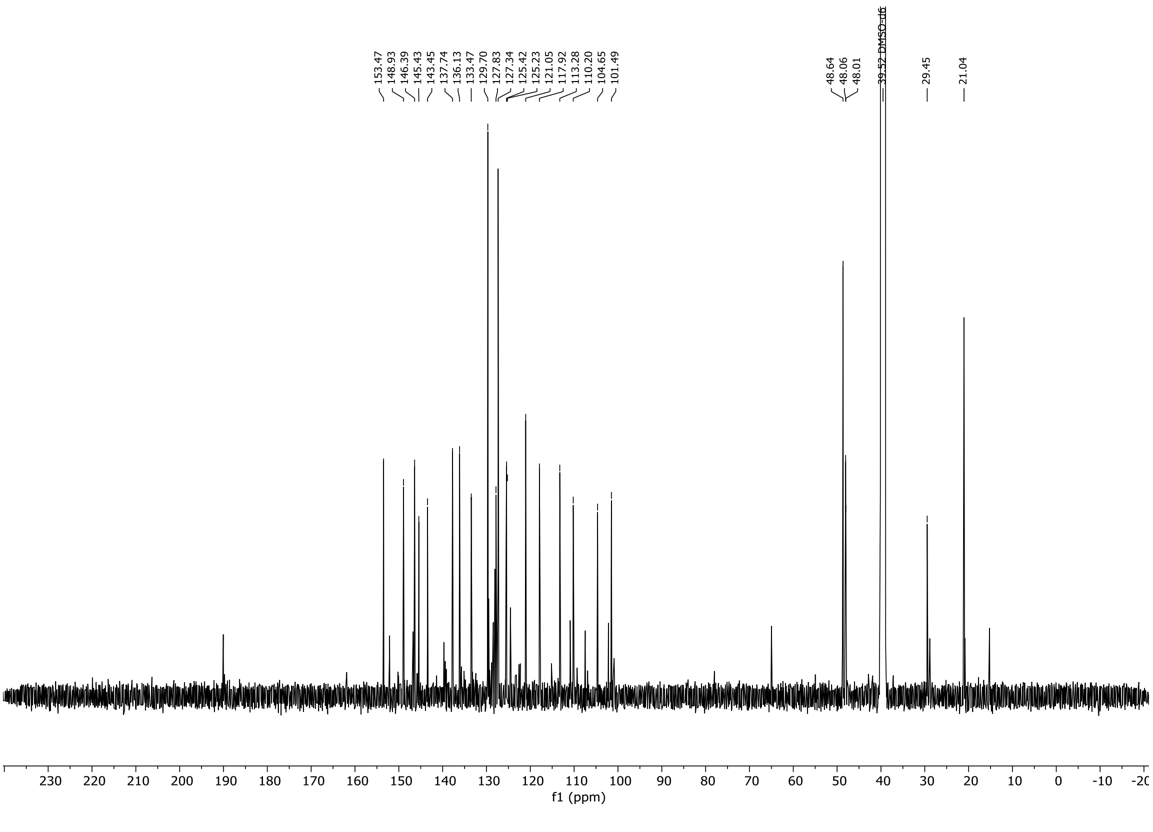

*10.3.29. N'-(4,5-Dimethoxy-2-(2-(2-oxo-1,4-dihydroquinazolin-3(2H)-yl)ethyl)benzylidene)-4-methylbenzenesulfonohydrazide (**4bc**)*

^{1}H NMR (500 MHz, DMSO-d_6):

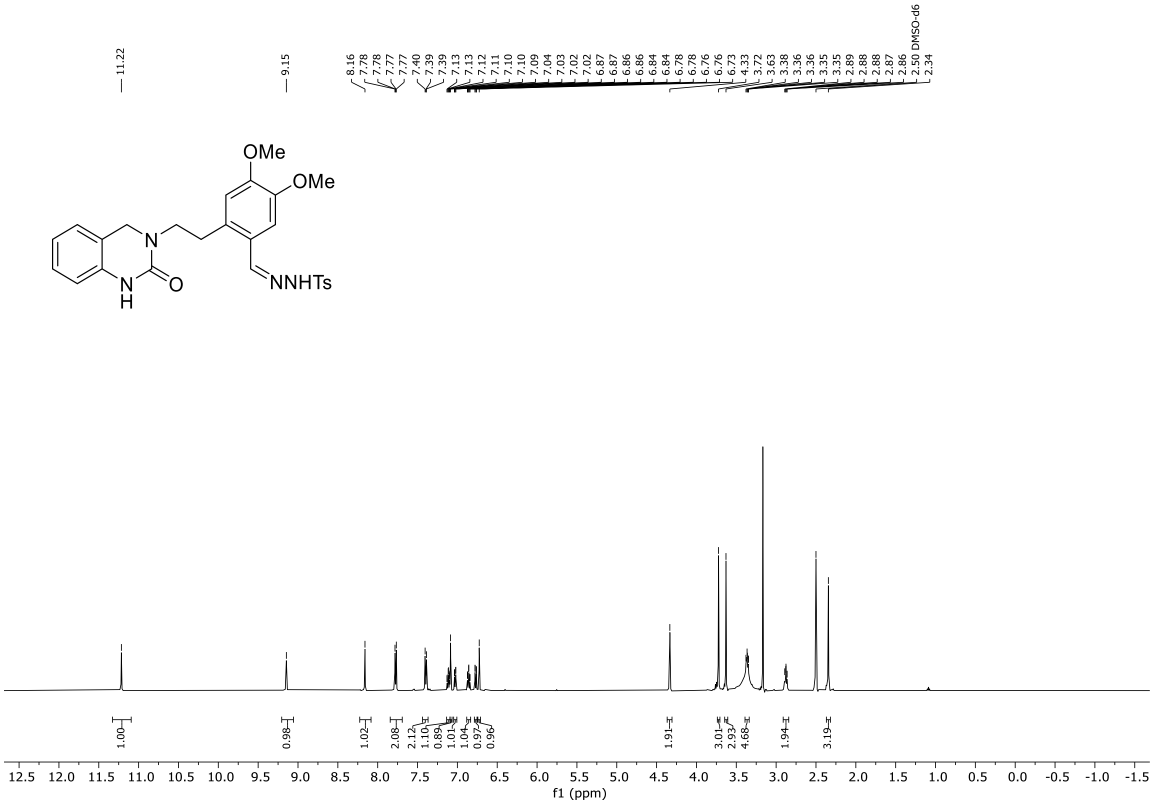

^{13}C NMR (126 MHz, DMSO-d_6):

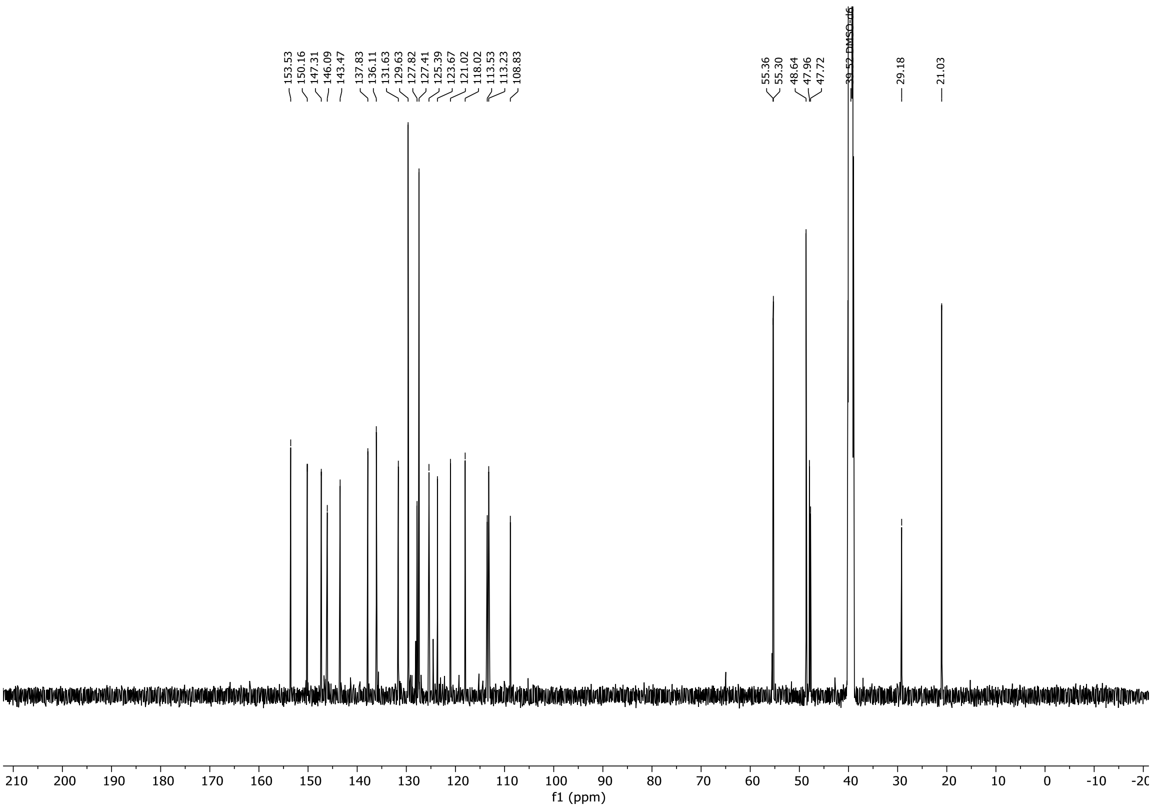

*10.3.30. N'-(2-(2-Oxo-1,4-dihydroquinazolin-3(2H)-yl)ethylidene)-4-methylbenzenesulfonohydrazide (**4ca**)*

^{1}H NMR (500 MHz, DMSO-d_6):

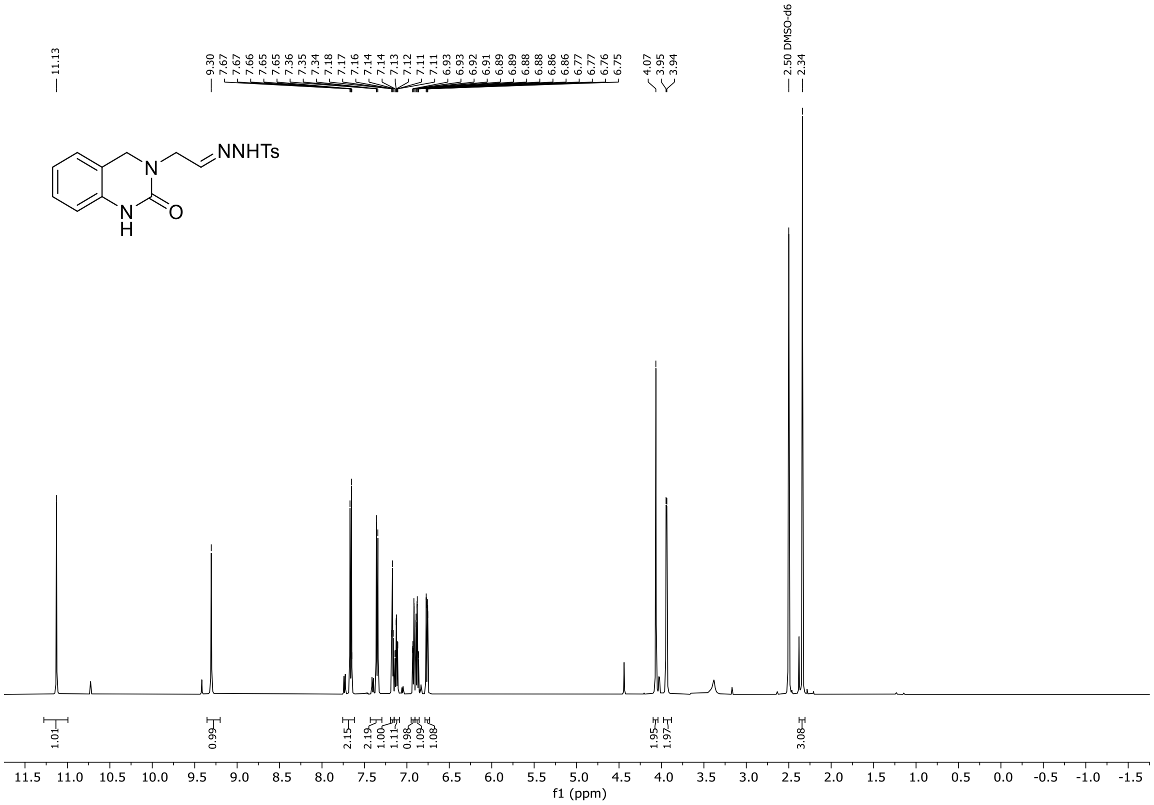

^{13}C NMR (126 MHz, DMSO-d_6):

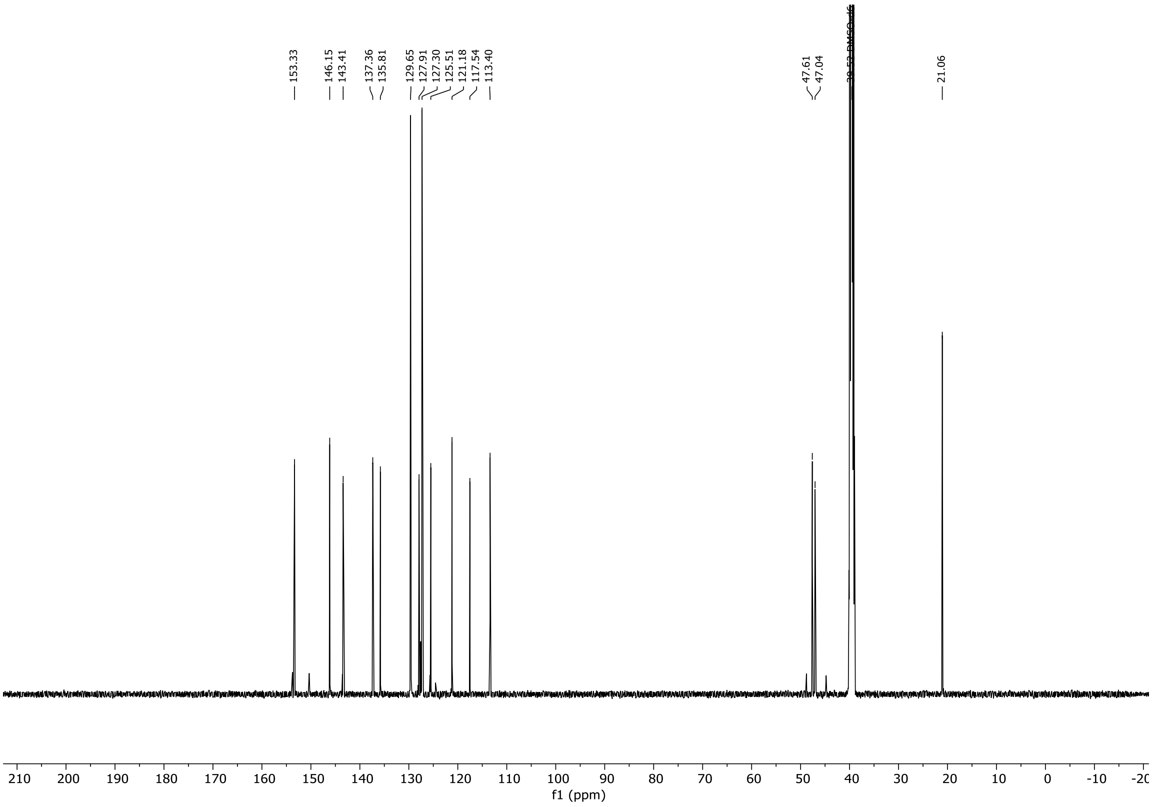

10.4. Individual Substrates

*10.4.1. 2-(1,3-Dioxolan-2-yl)benzonitrile (**SI-9**)*

^{1}H NMR (500 MHz, $CDCl_3$):

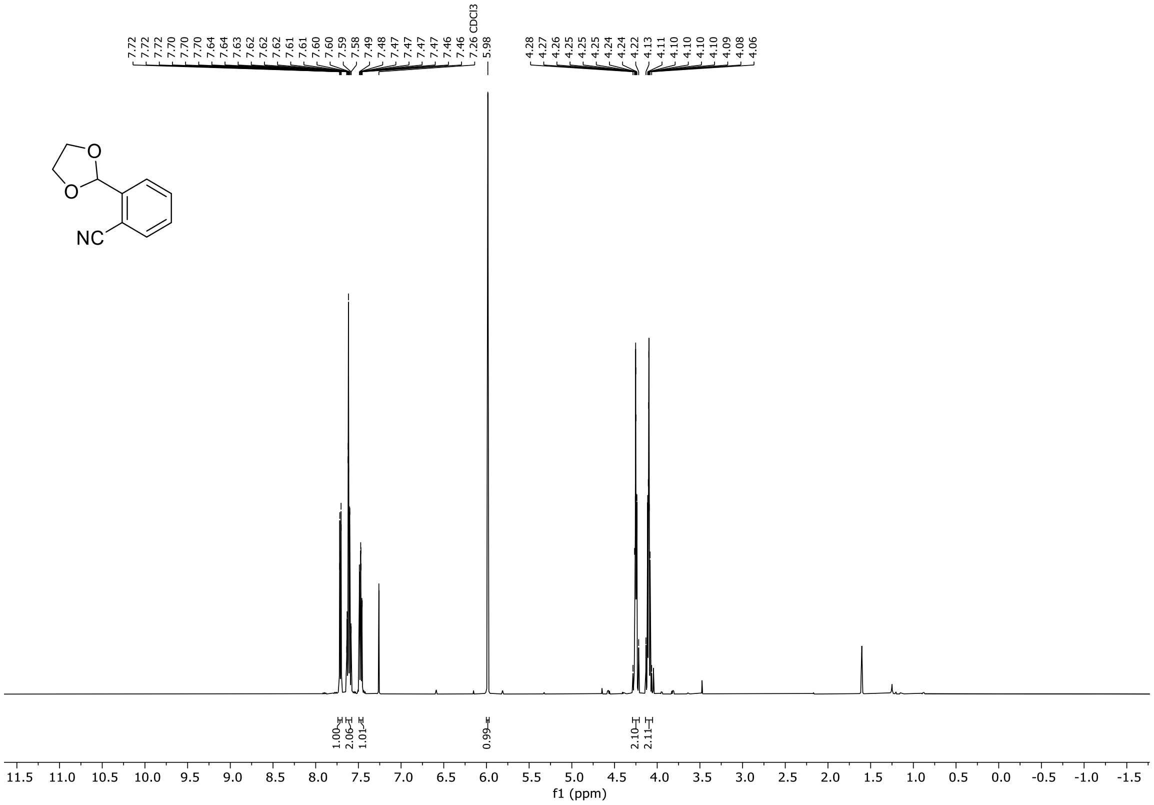

^{13}C NMR (126 MHz, $CDCl_3$):

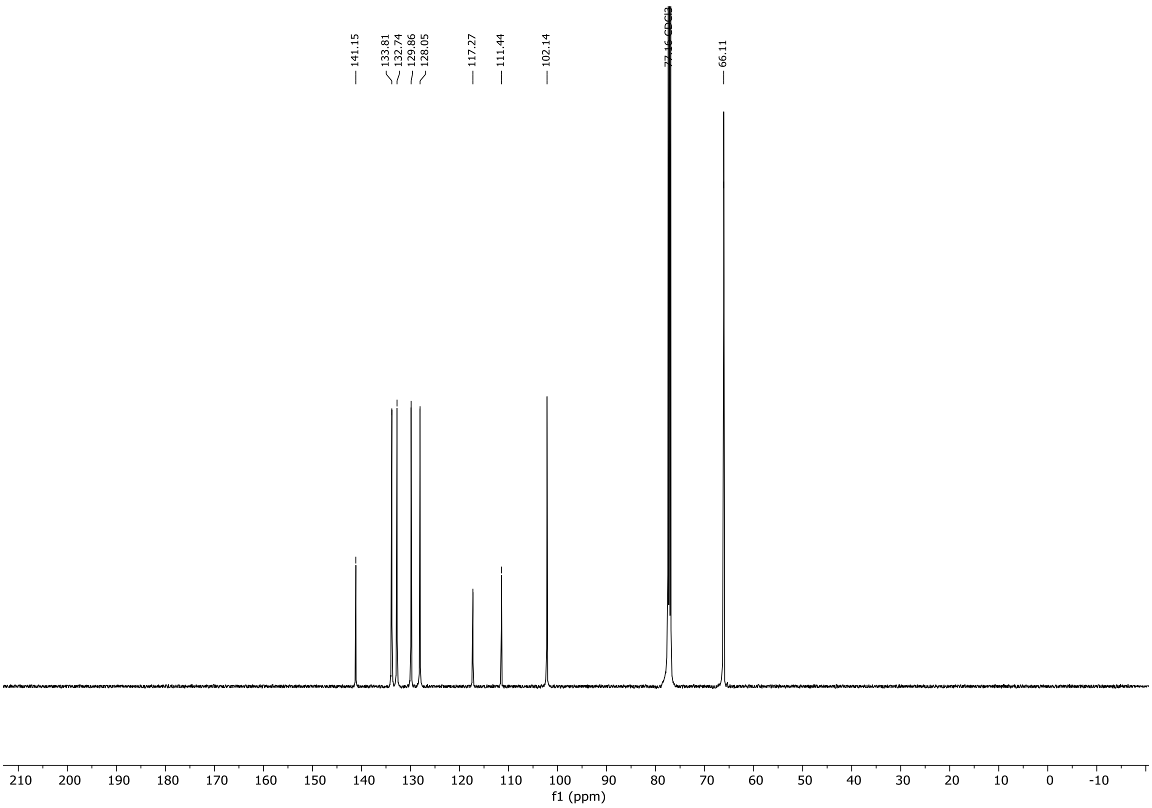

*10.4.2. 2-(1,3-Dioxolan-2-yl)benzonitrile (**9ca**)*

^{1}H NMR (500 MHz, CD_2Cl_2):

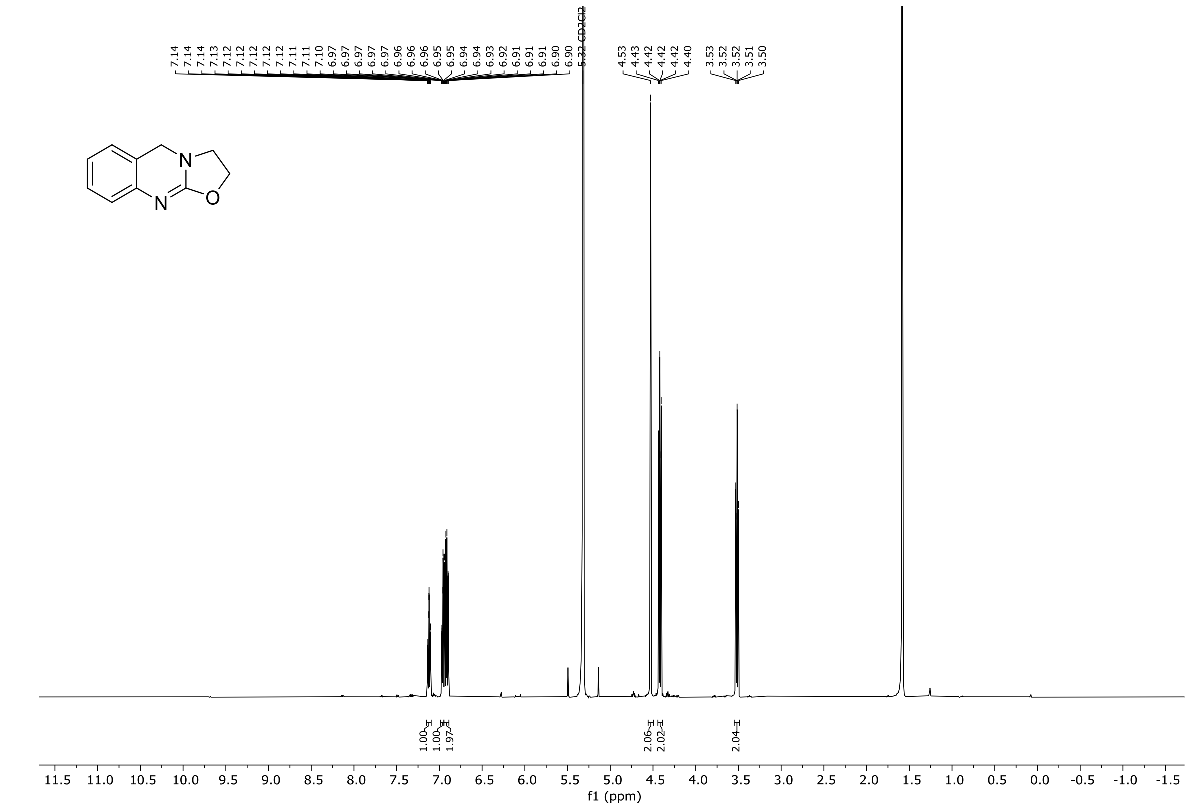

^{13}C NMR (126 MHz, CD_2Cl_2):

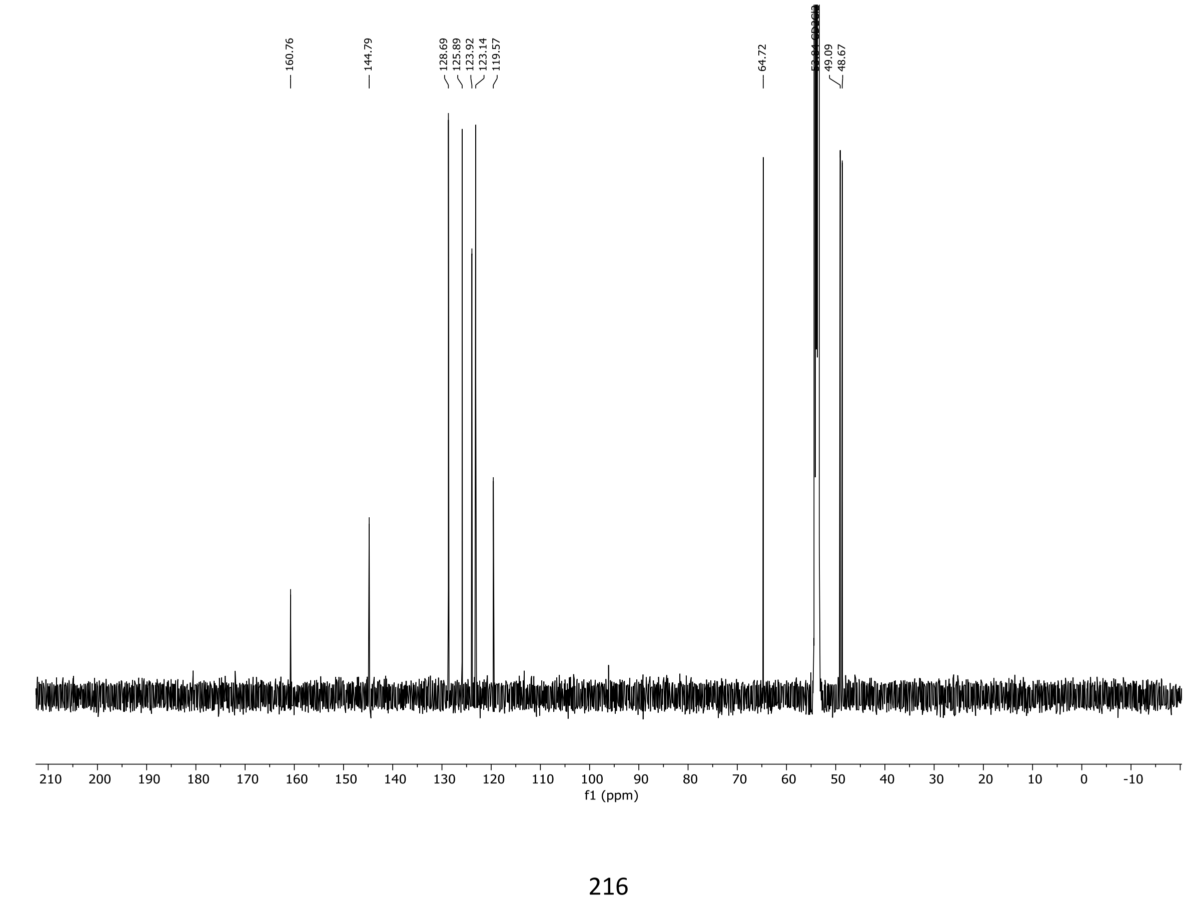

10.5. Catalysis Products

*10.5.1. 2,3,6,10b-Tetrahydropyrrolo[1,2-c]quinazolin-5(1H)-one (**5a**)*

^{1}H NMR (500 MHz, $CDCl_3$):

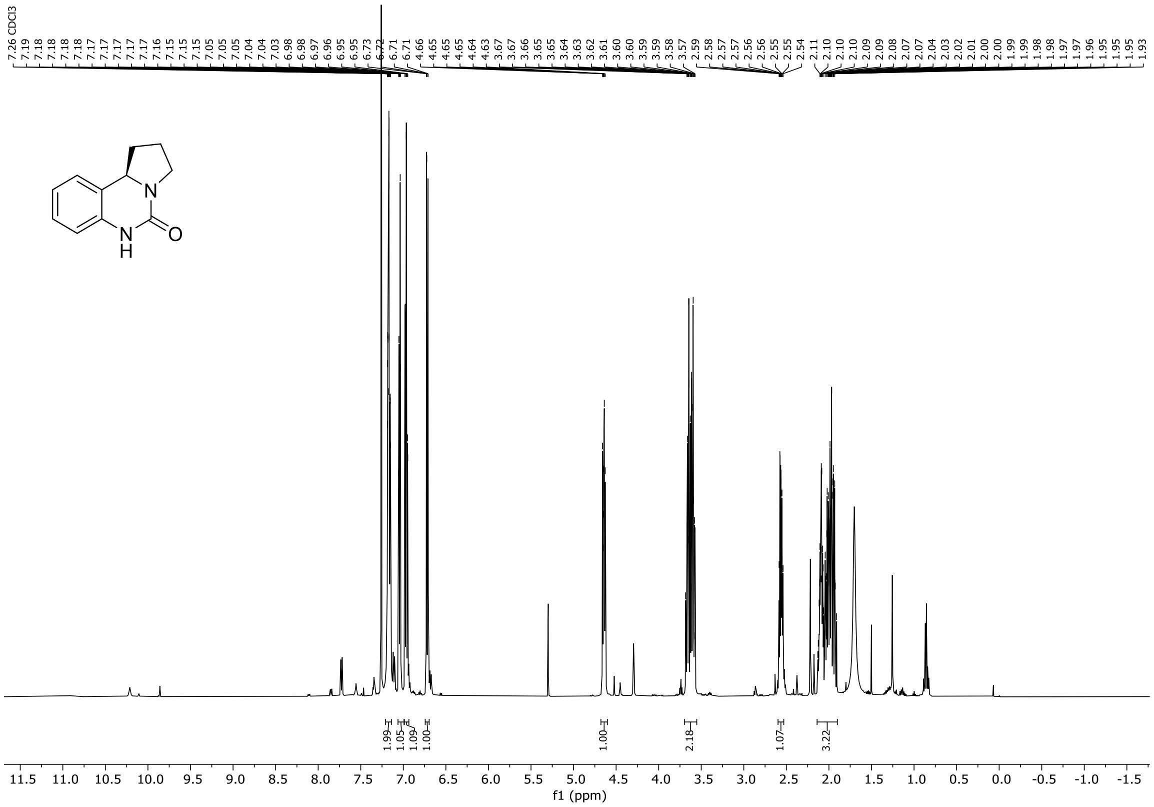

^{13}C NMR (126 MHz, $CDCl_3$):

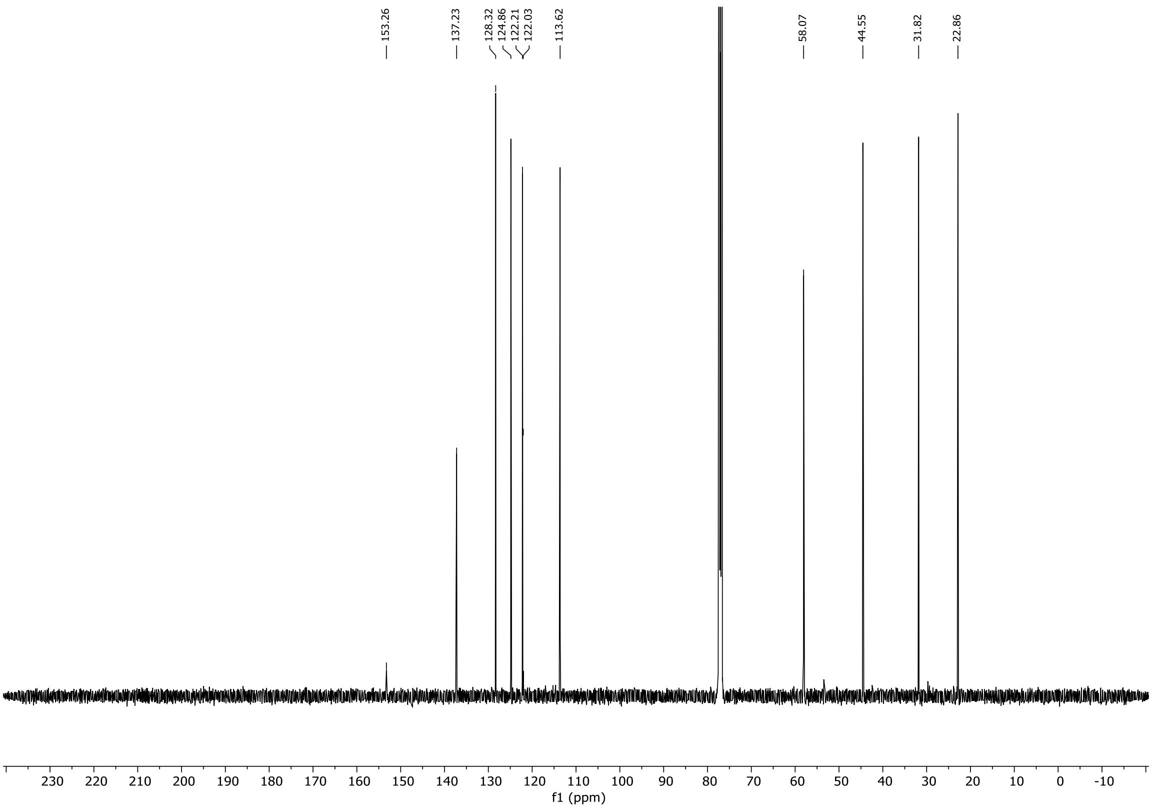

*10.5.2. 10-Bromo-2,3,6,10b-tetrahydropyrrolo[1,2-c]quinazolin-5(1H)-one (**5b**)*

^{1}H NMR (500 MHz, $CDCl_3$):

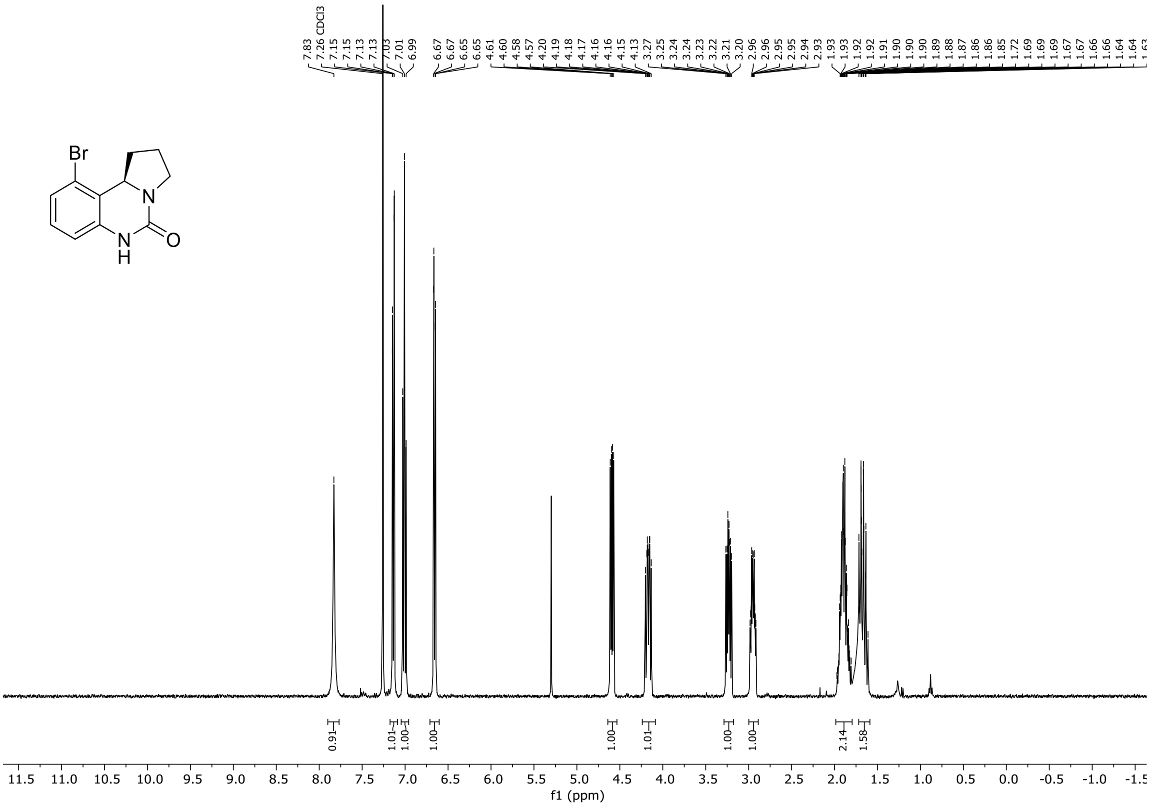

^{13}C NMR (126 MHz, $CDCl_3$):

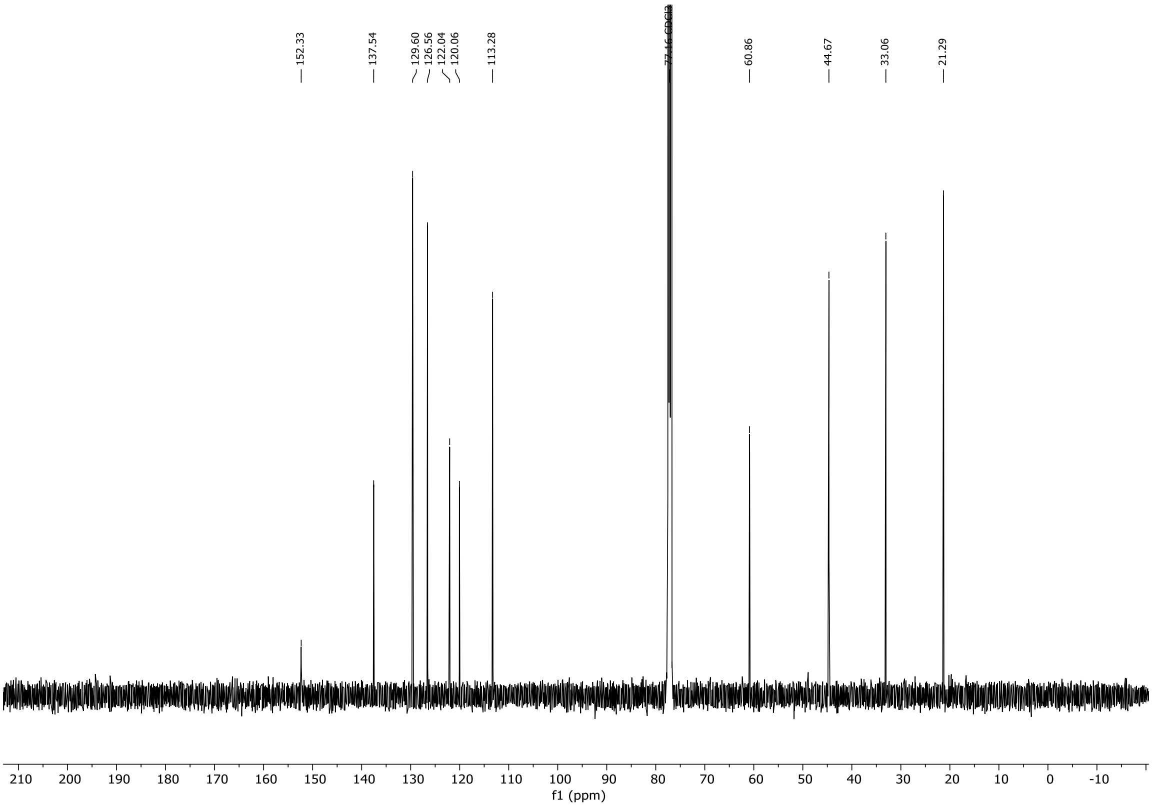

*10.5.3. 9-Bromo-2,3,6,10b-tetrahydropyrrolo[1,2-c]quinazolin-5(1H)-one (**5c**)*

^{1}H NMR (500 MHz, $CDCl_3$):

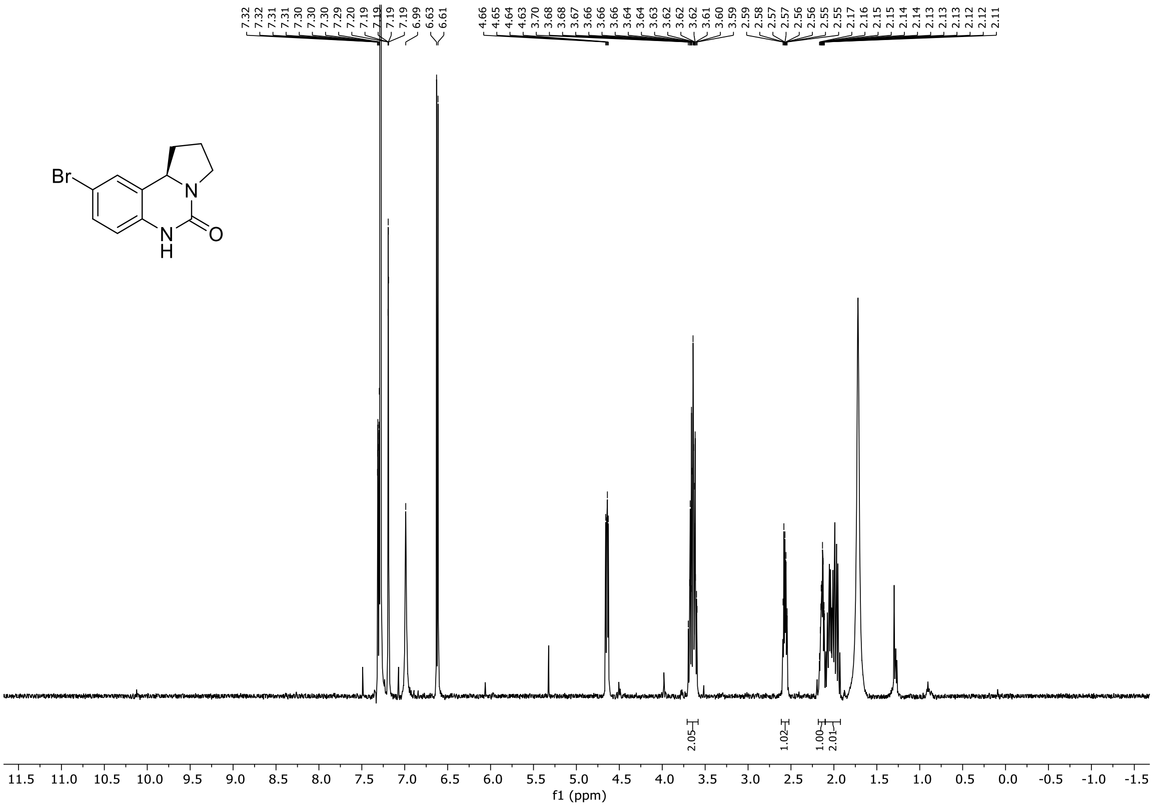

^{13}C NMR (126 MHz, $CDCl_3$):

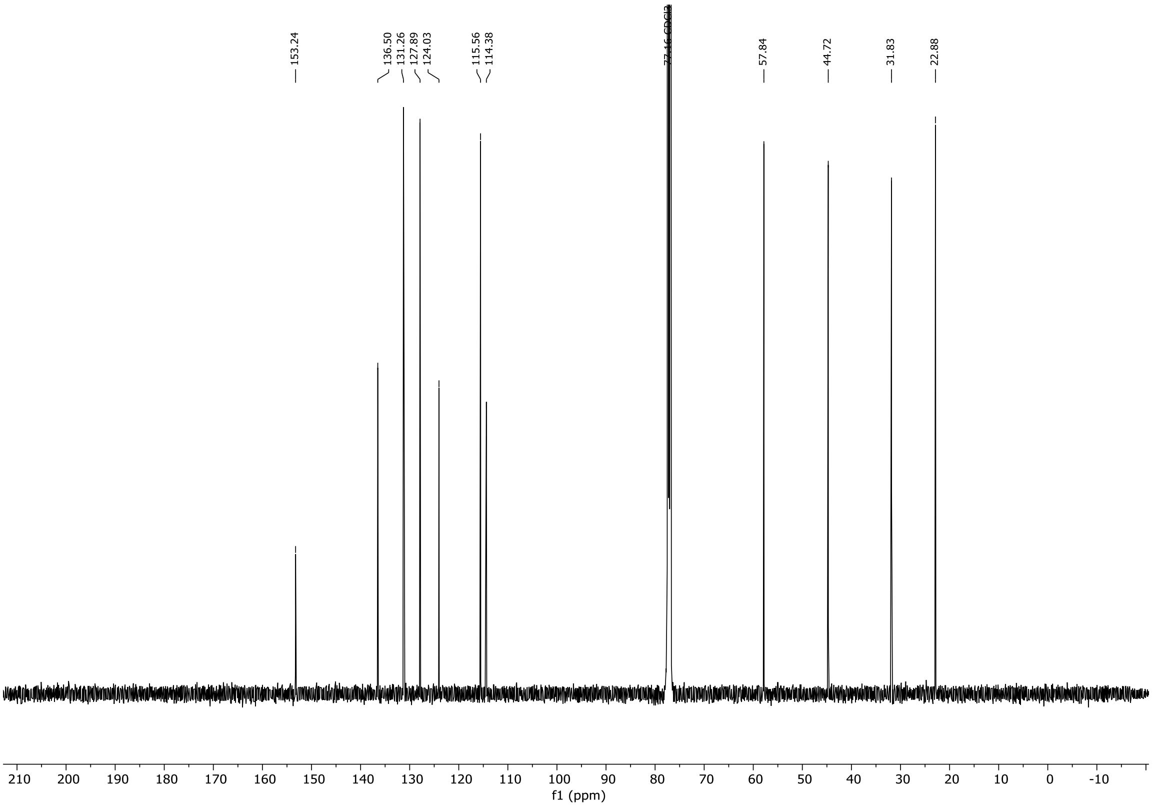

*10.5.4. 8-Bromo-2,3,6,10b-tetrahydropyrrolo[1,2-c]quinazolin-5(1H)-one (**5d**)*

^{1}H NMR (500 MHz, $CDCl_3$):

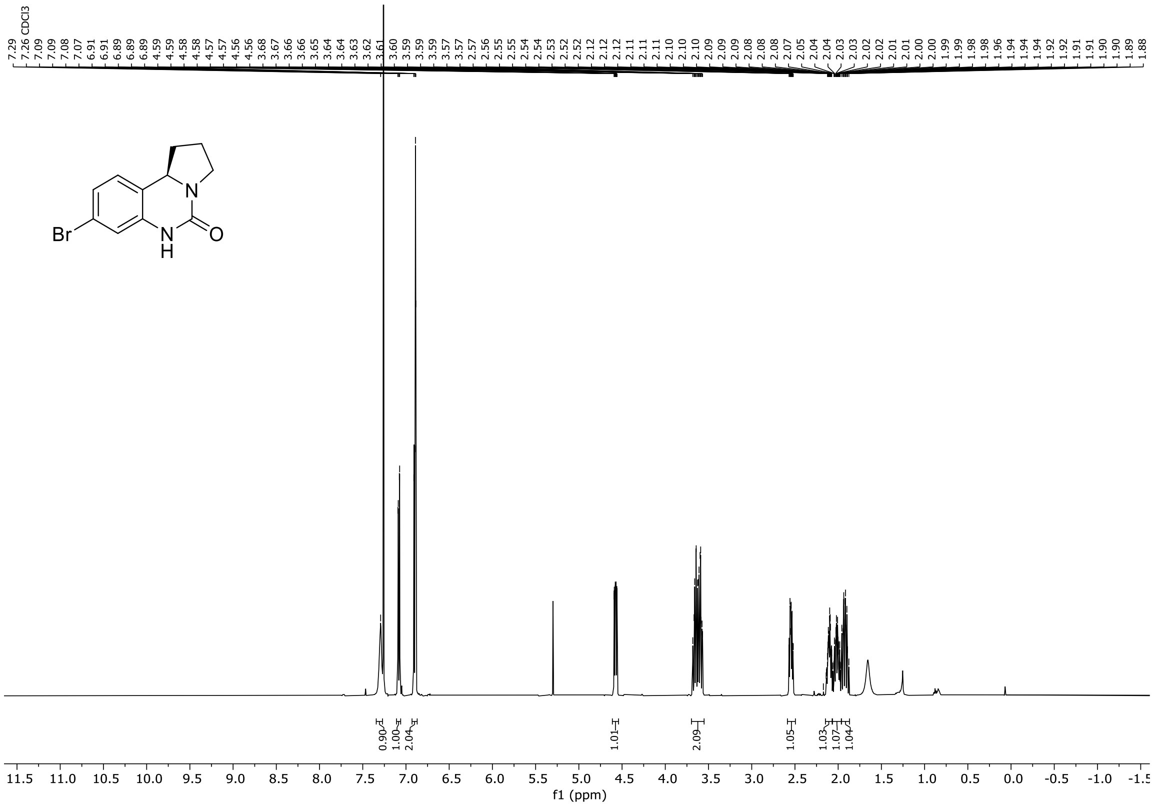

^{13}C NMR (126 MHz, $CDCl_3$):

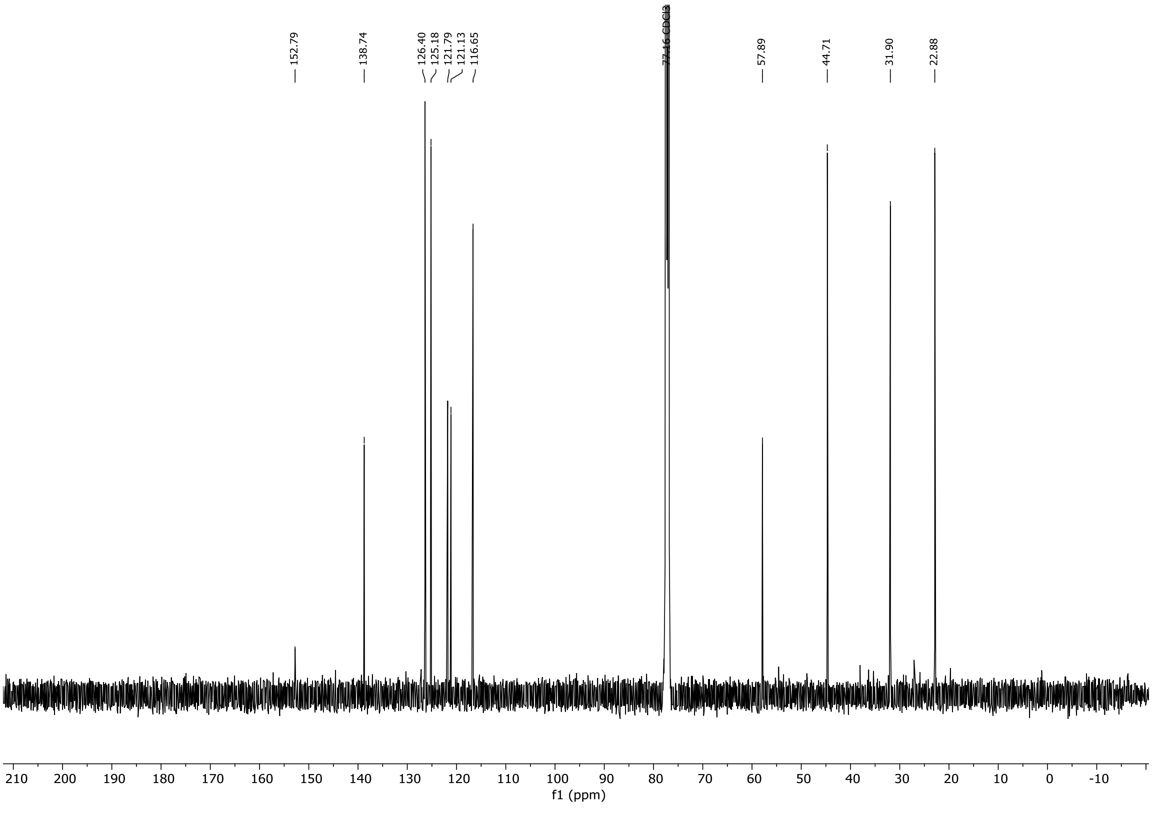

*10.5.5. 7-Bromo-2,3,6,10b-tetrahydropyrrolo[1,2-c]quinazolin-5(1H)-one (**5e**)*

^{1}H NMR (500 MHz, $CDCl_3$):

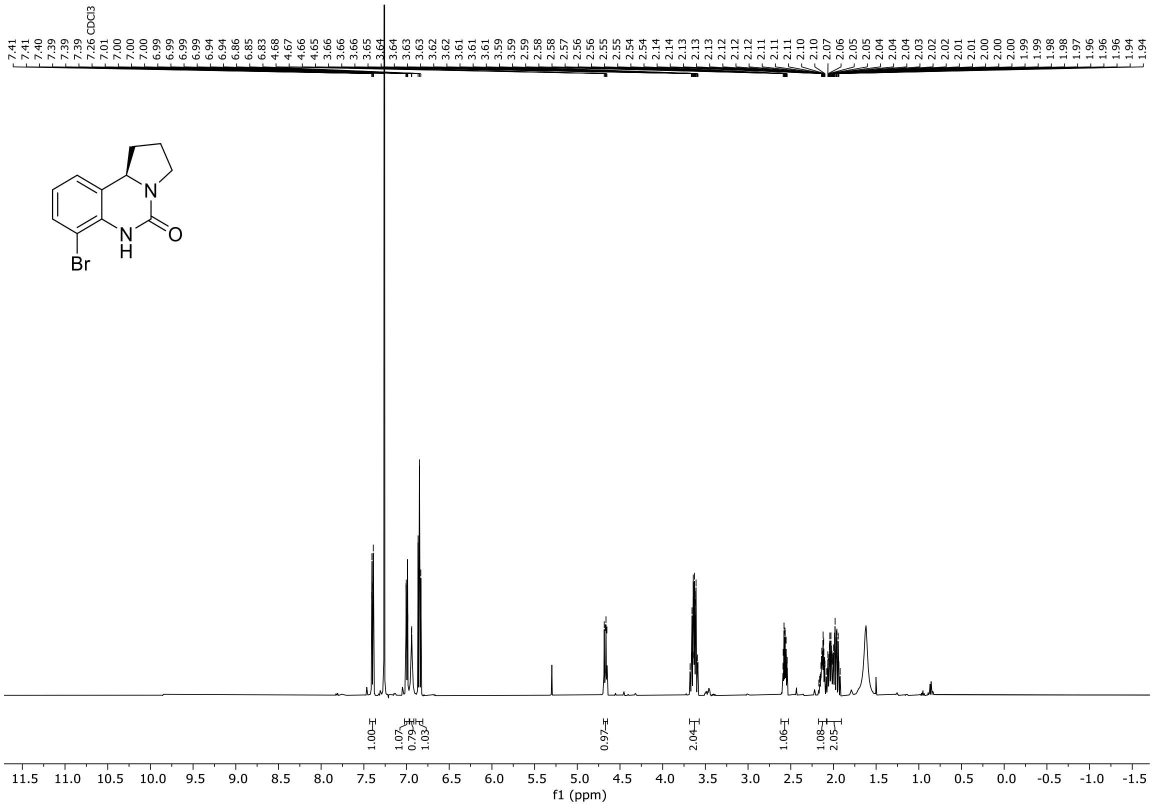

^{13}C NMR (126 MHz, $CDCl_3$):

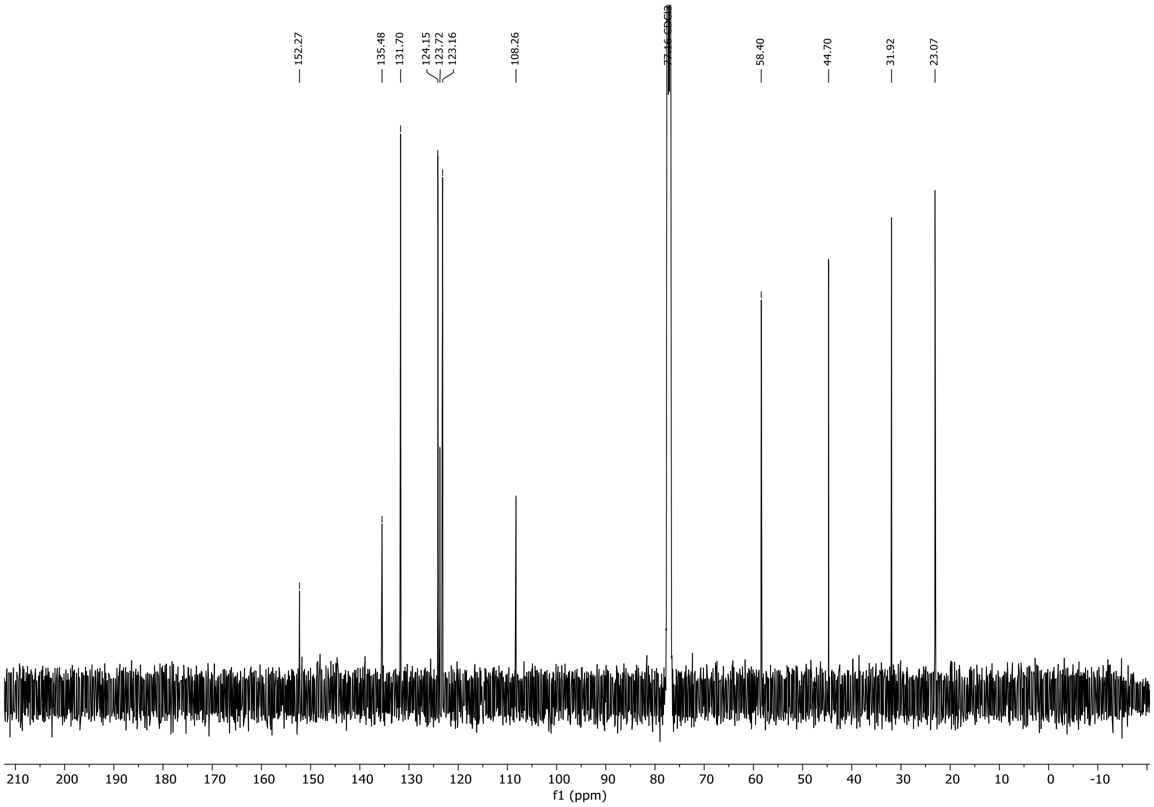

*10.5.6. 9-Chloro-2,3,6,10b-tetrahydropyrrolo[1,2-c]quinazolin-5(1H)-one (**5f**)*

^{1}H NMR (500 MHz, $CDCl_3$):

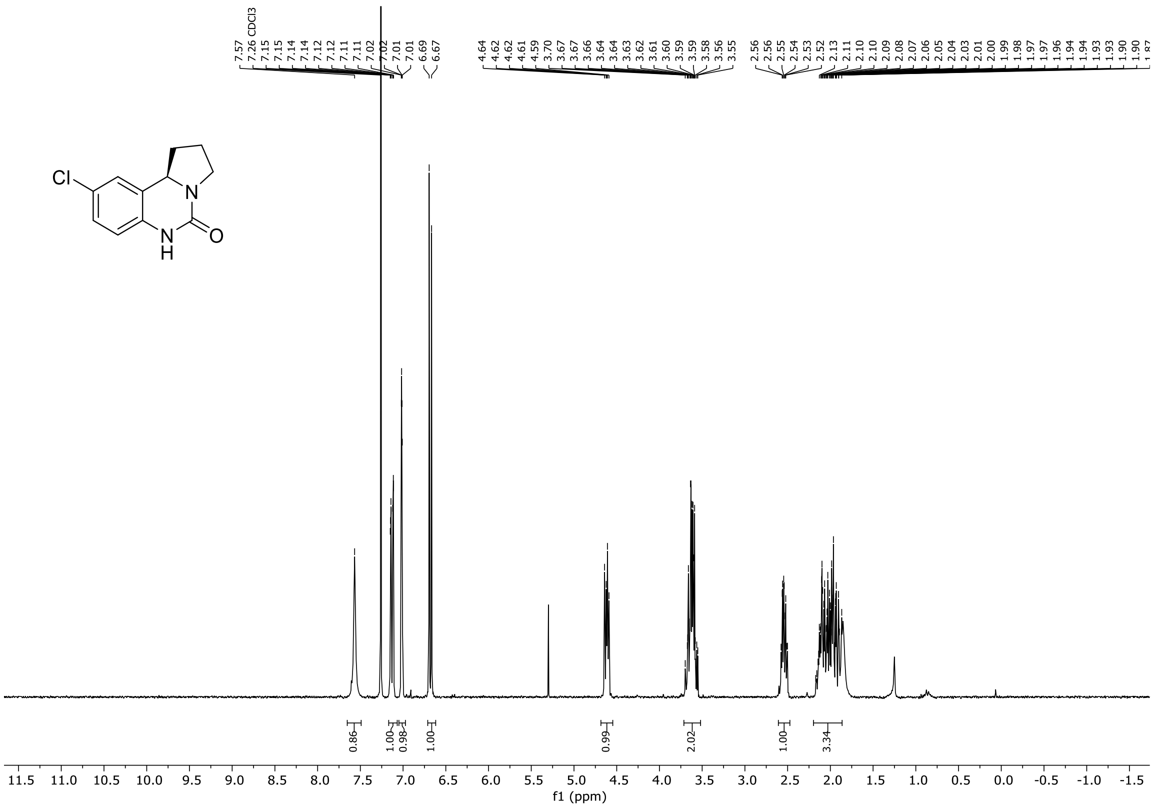

^{13}C NMR (126 MHz, $CDCl_3$):

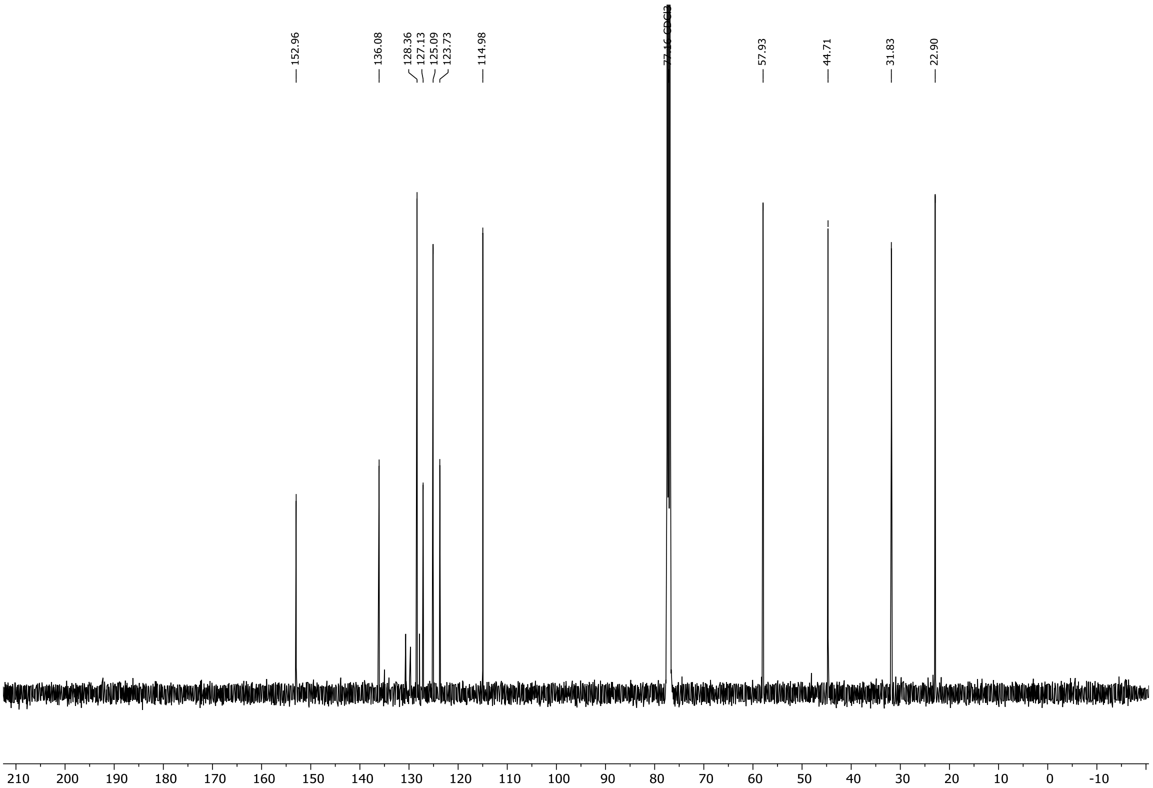

*10.5.7. 8-Fluoro-2,3,6,10b-tetrahydropyrrolo[1,2-c]quinazolin-5(1H)-one (**5g**)*

^{1}H NMR (500 MHz, $CDCl_3$):

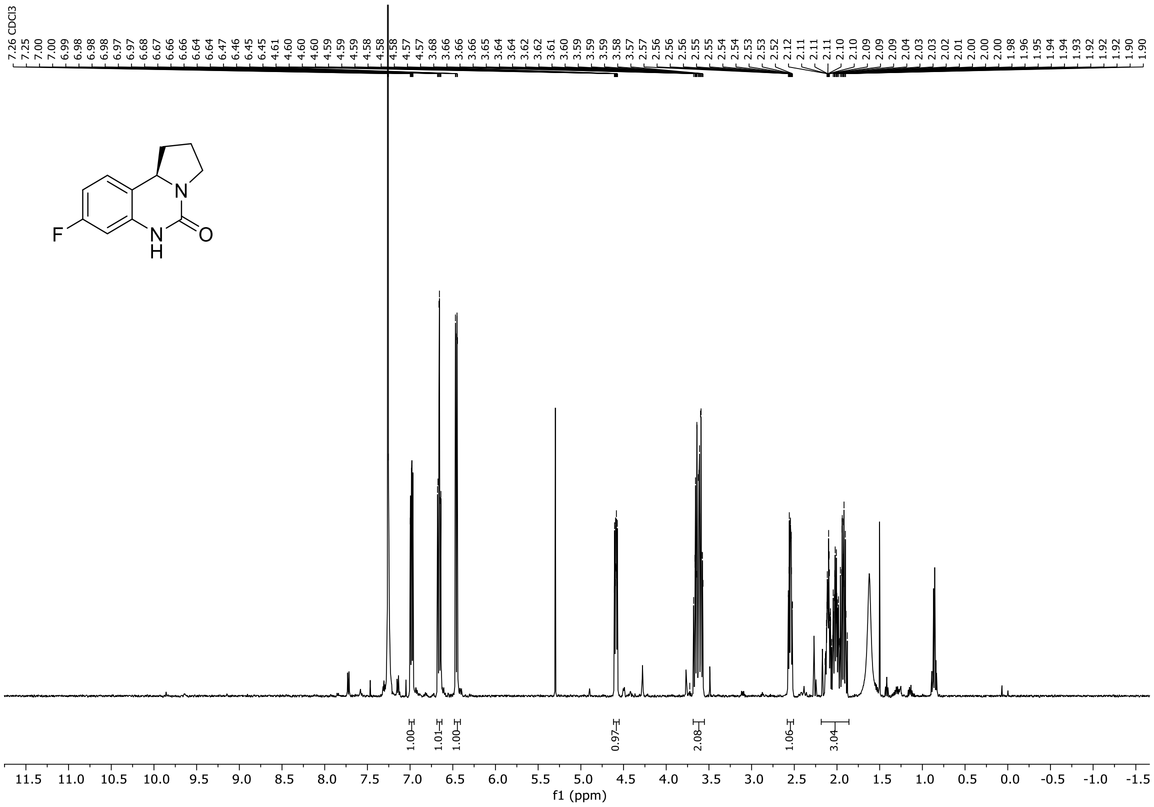

^{13}C NMR (126 MHz, $CDCl_3$):

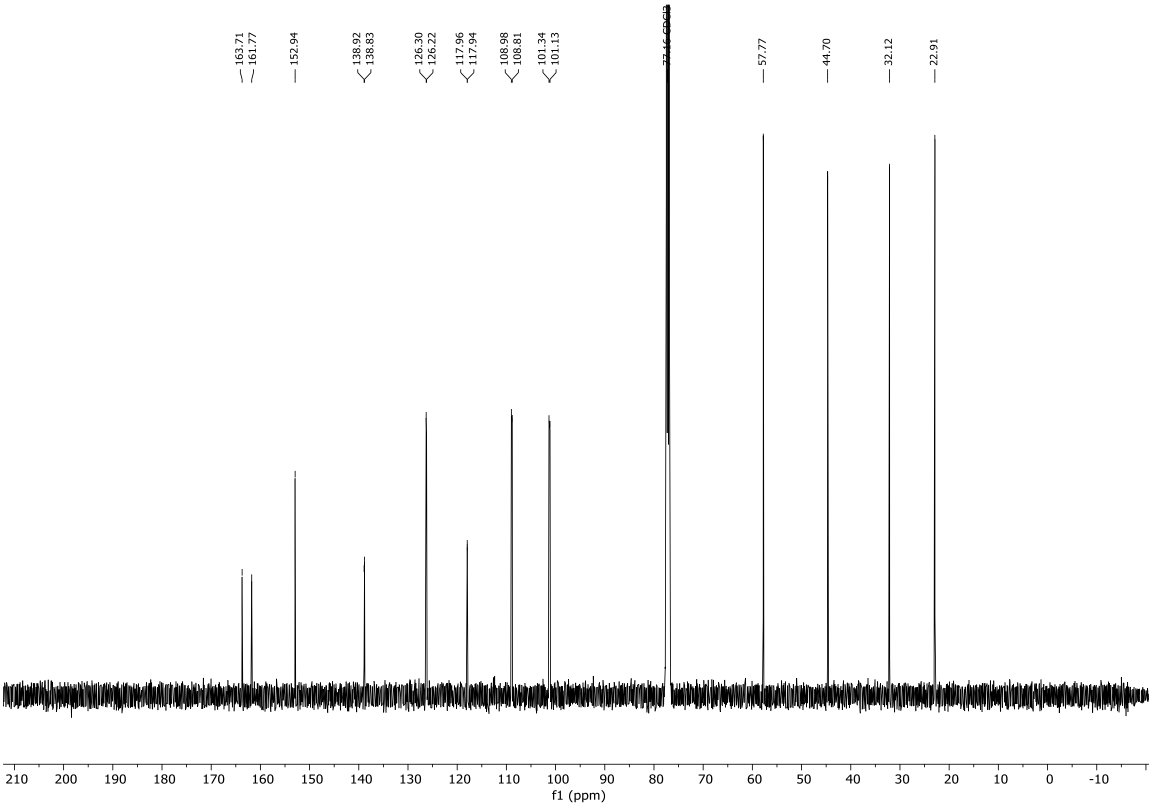

^{19}F NMR (471 MHz, $CDCl_3$):

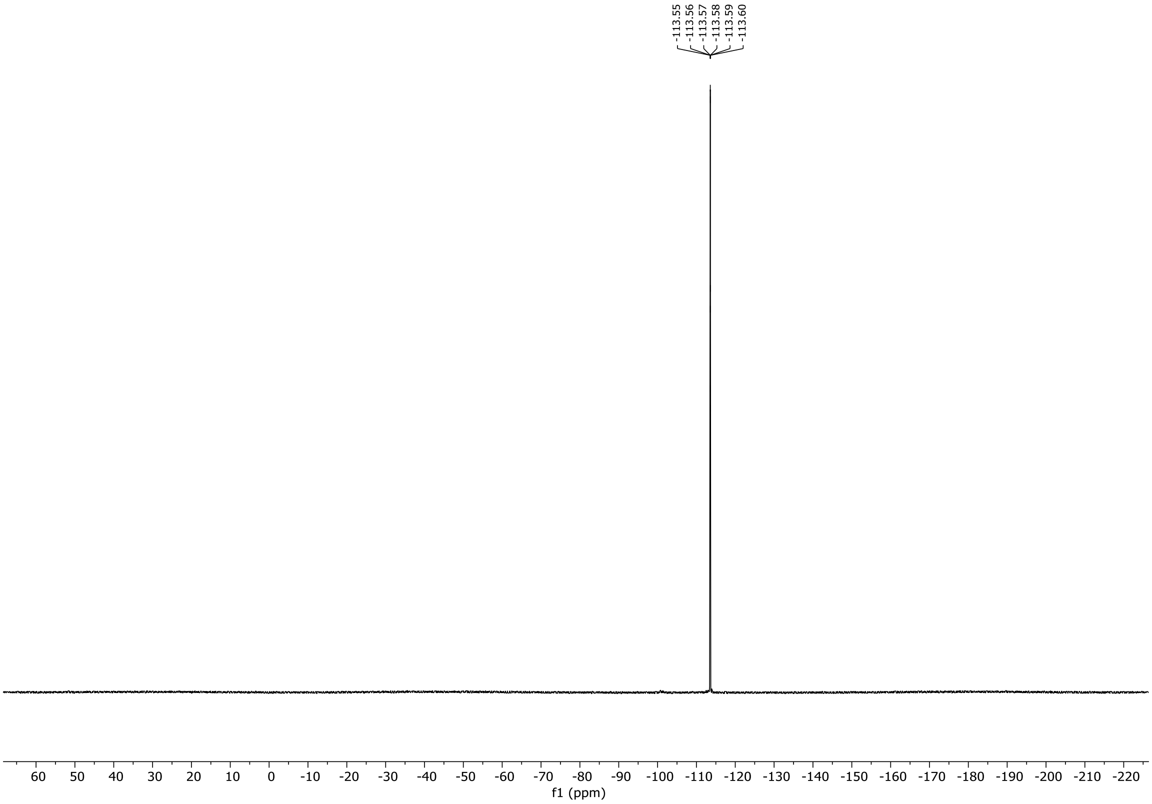

*10.5.8. 8-(Trifluoromethyl)-2,3,6,10b-tetrahydropyrrolo[1,2-c]quinazolin-5(1H)-one (**5h**)*

^{1}H NMR (500 MHz, $CDCl_3$):

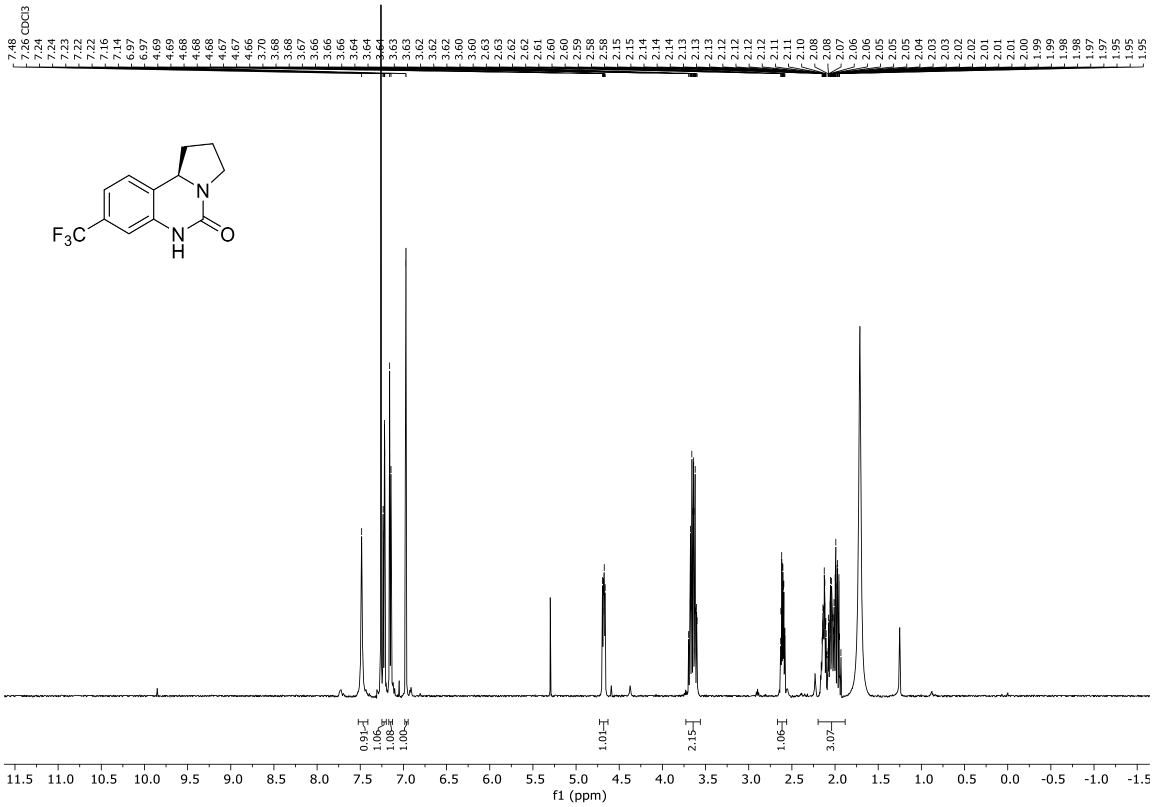

^{13}C NMR (126 MHz, $CDCl_3$):

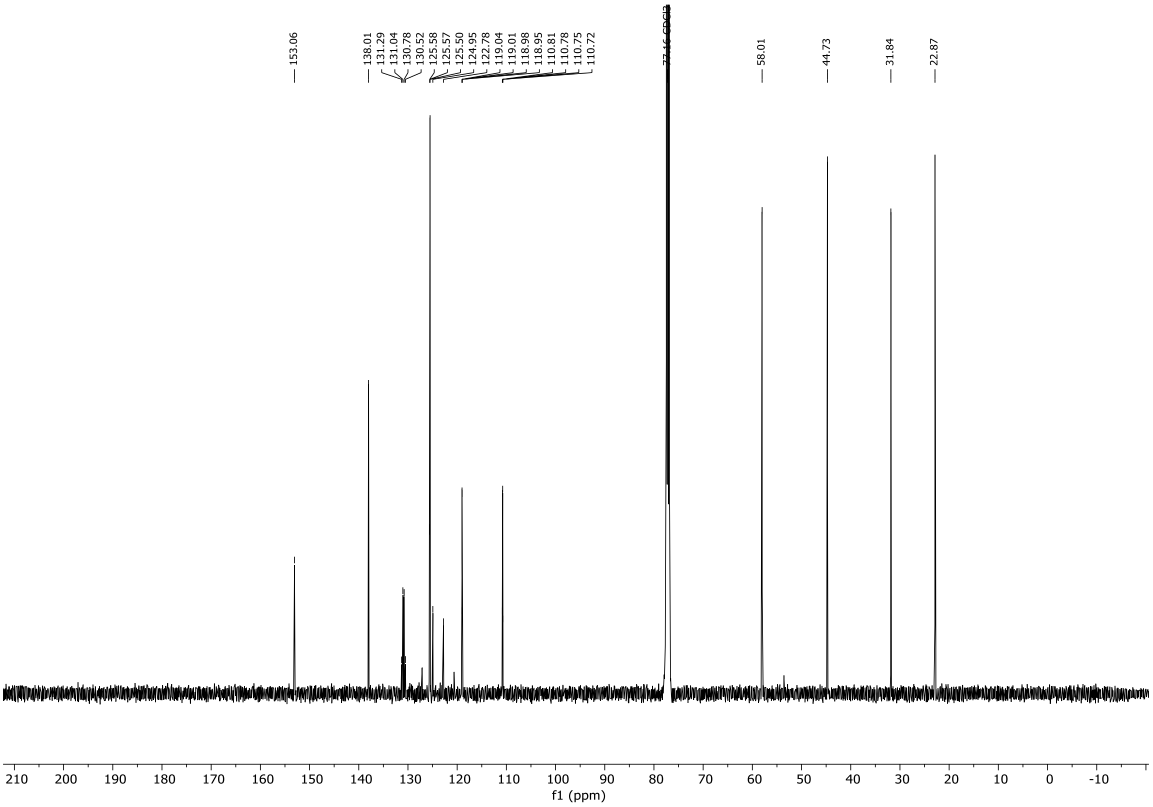

^{19}F NMR (471 MHz, $CDCl_3$):

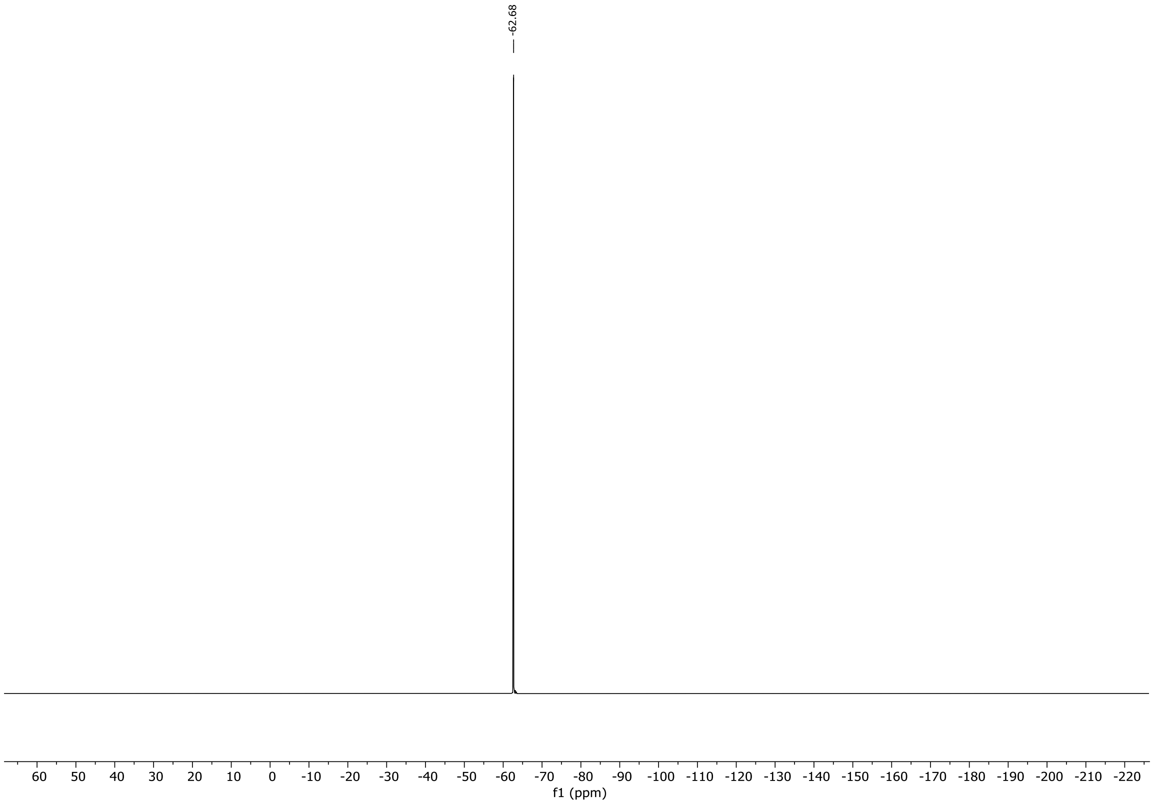

*10.5.9. 9-Vinyl-2,3,6,10b-tetrahydropyrrolo[1,2-c]quinazolin-5(1H)-one (**5i**)*

^{1}H NMR (500 MHz, $CDCl_3$):

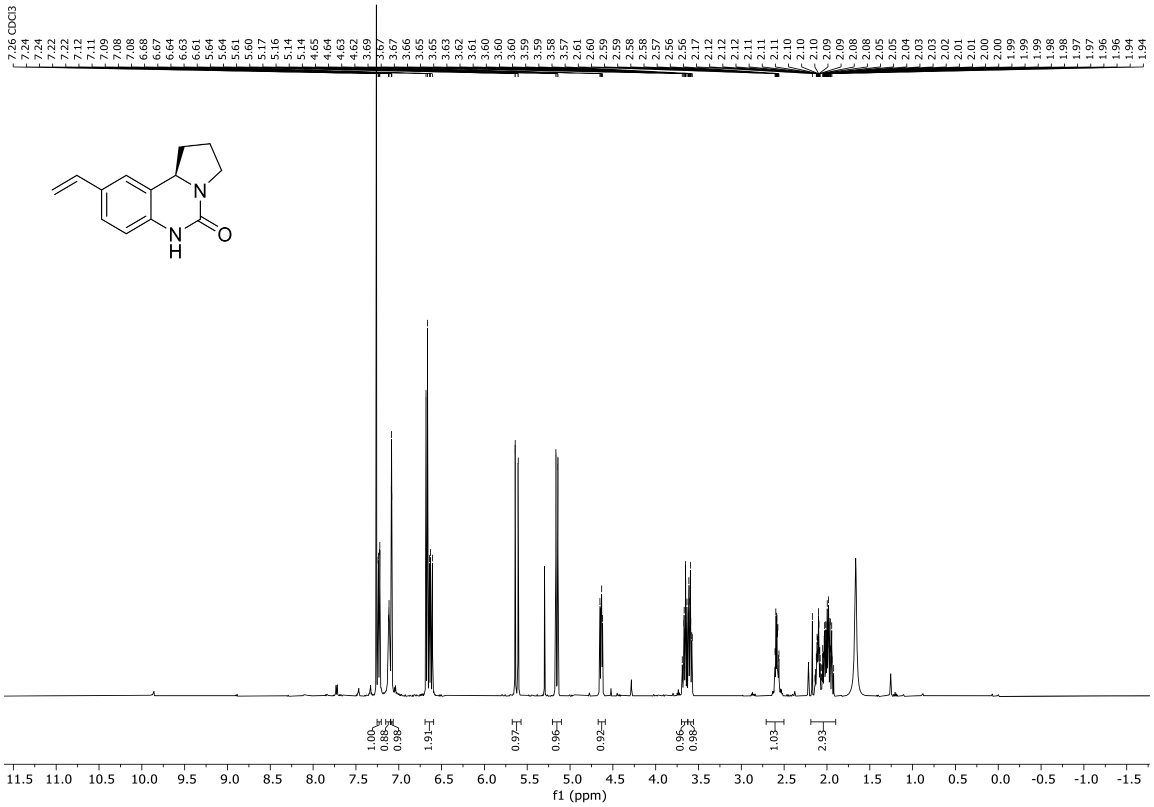

^{13}C NMR (126 MHz, $CDCl_3$):

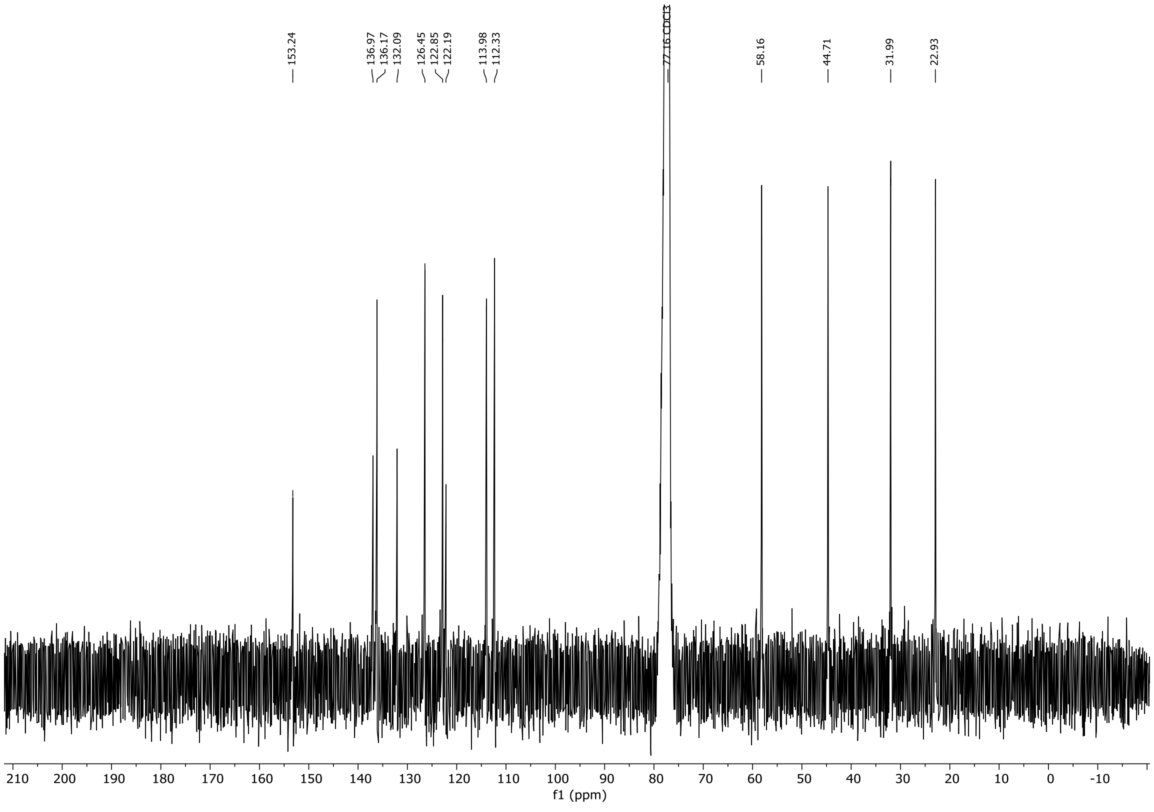

*10.5.10. 9-Allyl-2,3,6,10b-tetrahydropyrrolo[1,2-c]quinazolin-5(1H)-one (**5j**)*

^{1}H NMR (500 MHz, $CDCl_3$):

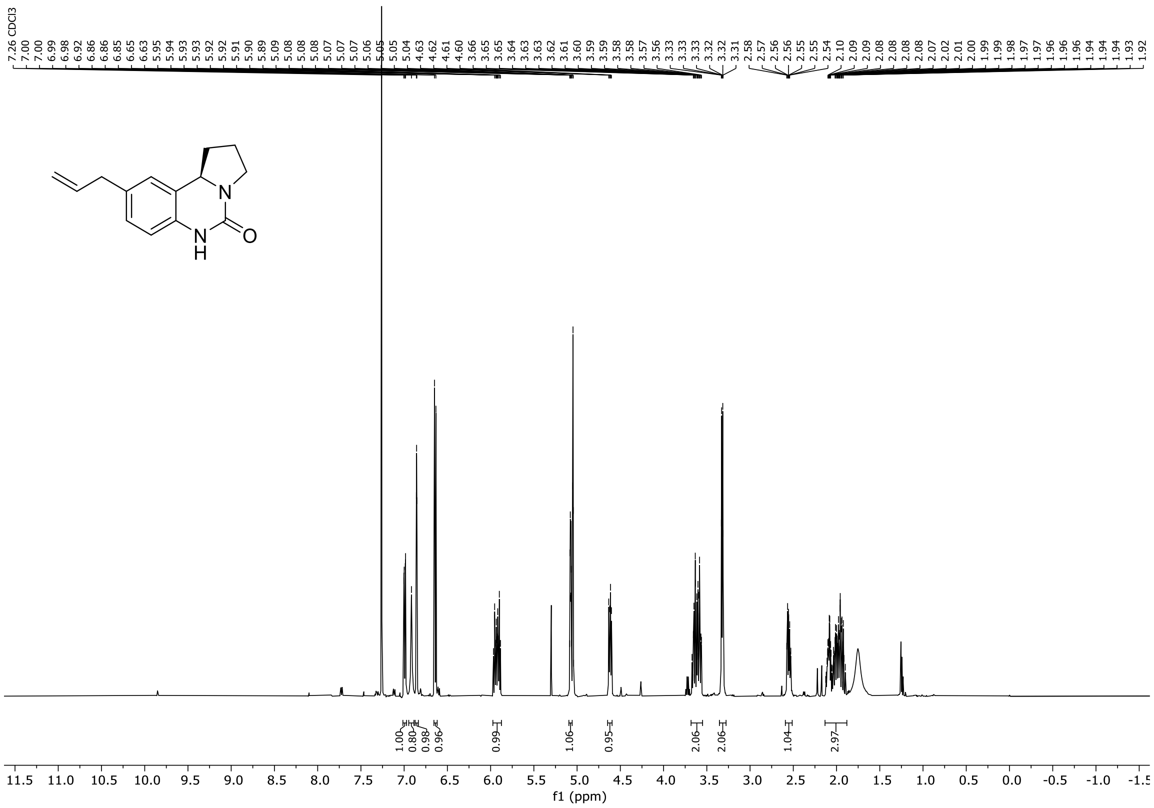

^{13}C NMR (126 MHz, $CDCl_3$):

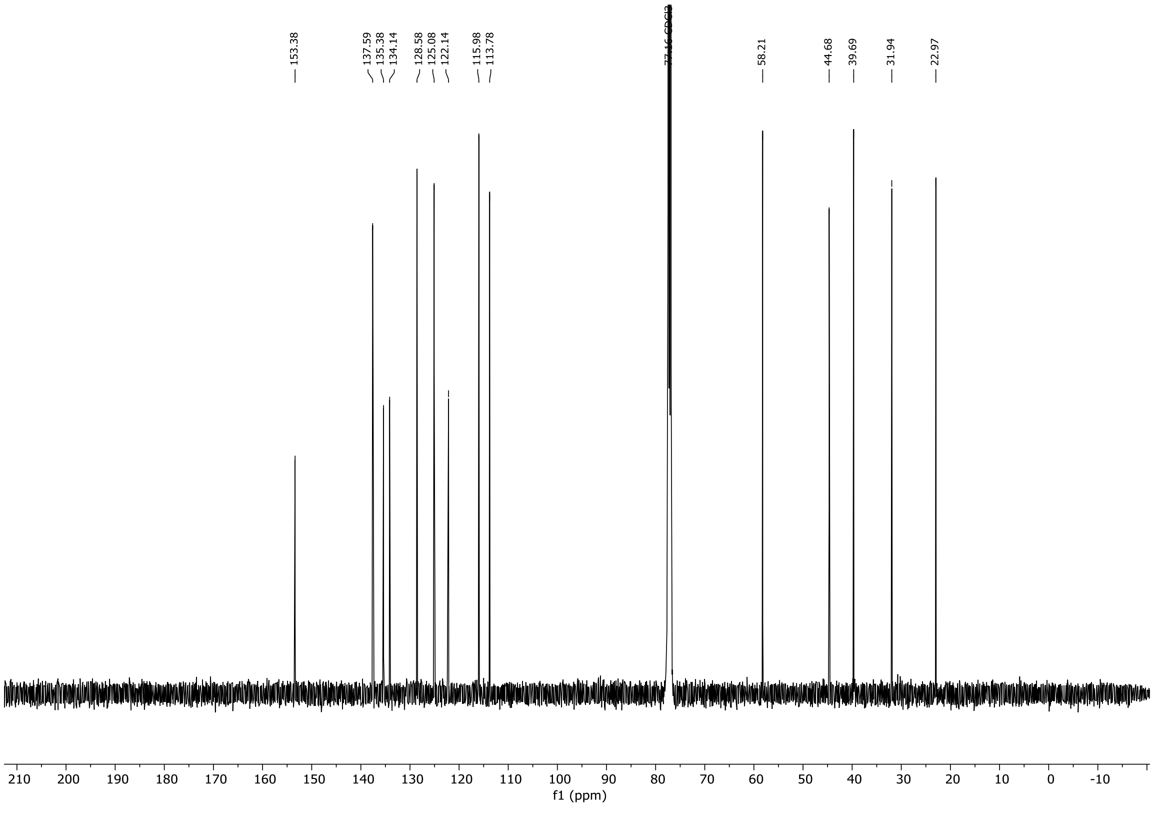

*10.5.11. 7-Methyl-2,3,6,10b-tetrahydropyrrolo[1,2-c]quinazolin-5(1H)-one (**5k**)*

^{1}H NMR (500 MHz, $CDCl_3$):

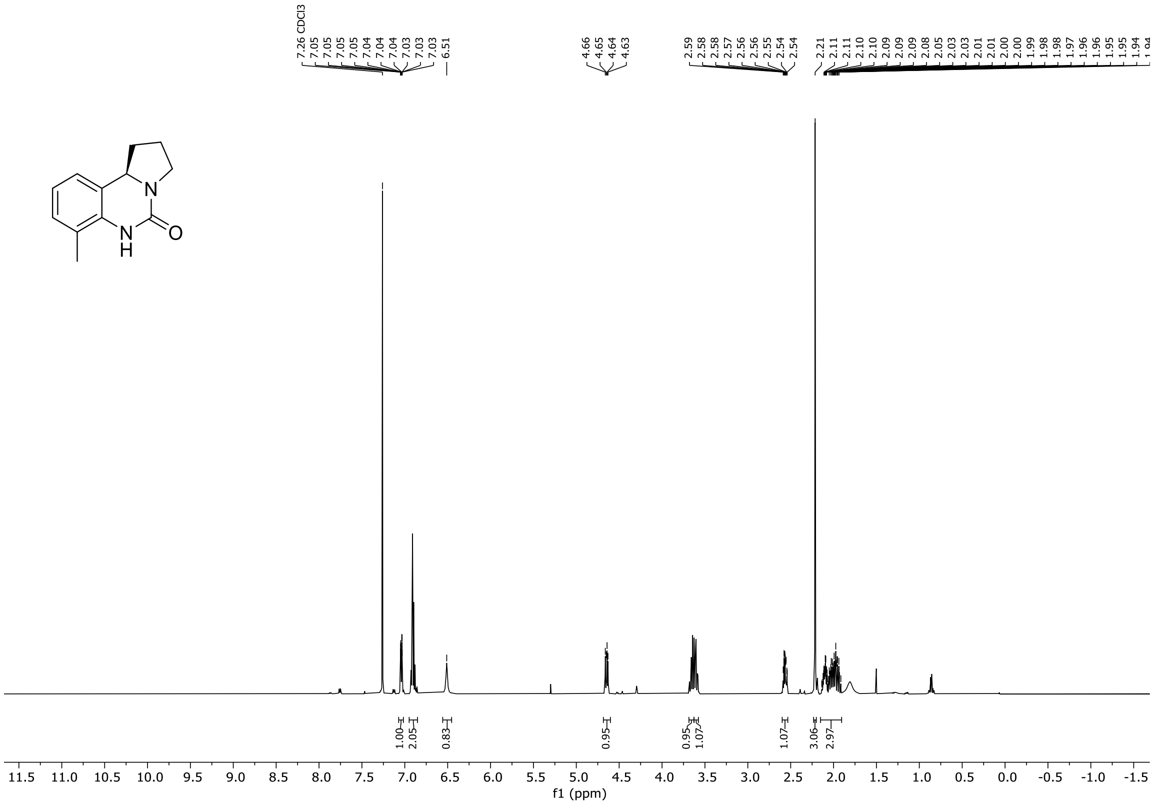

^{13}C NMR (126 MHz, $CDCl_3$):

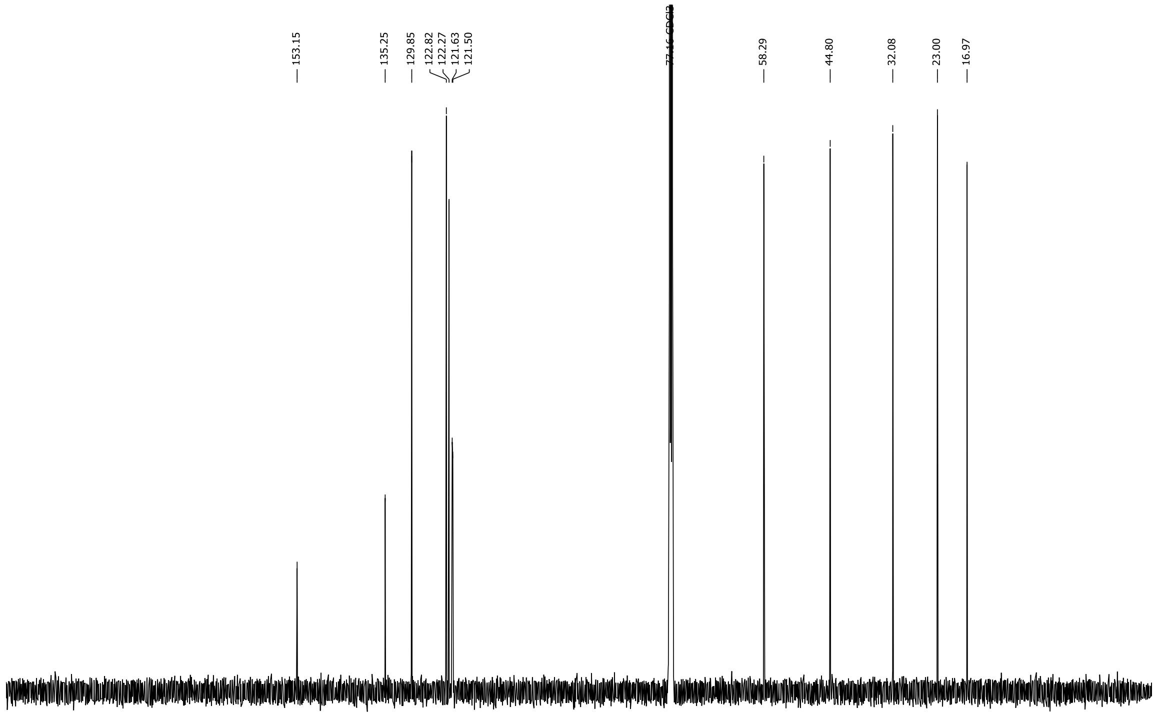

*10.5.12. 9-Methoxy-2,3,6,10b-tetrahydropyrrolo[1,2-c]quinazolin-5(1H)-one (**5l**)*

^{1}H NMR (500 MHz, $CDCl_3$):

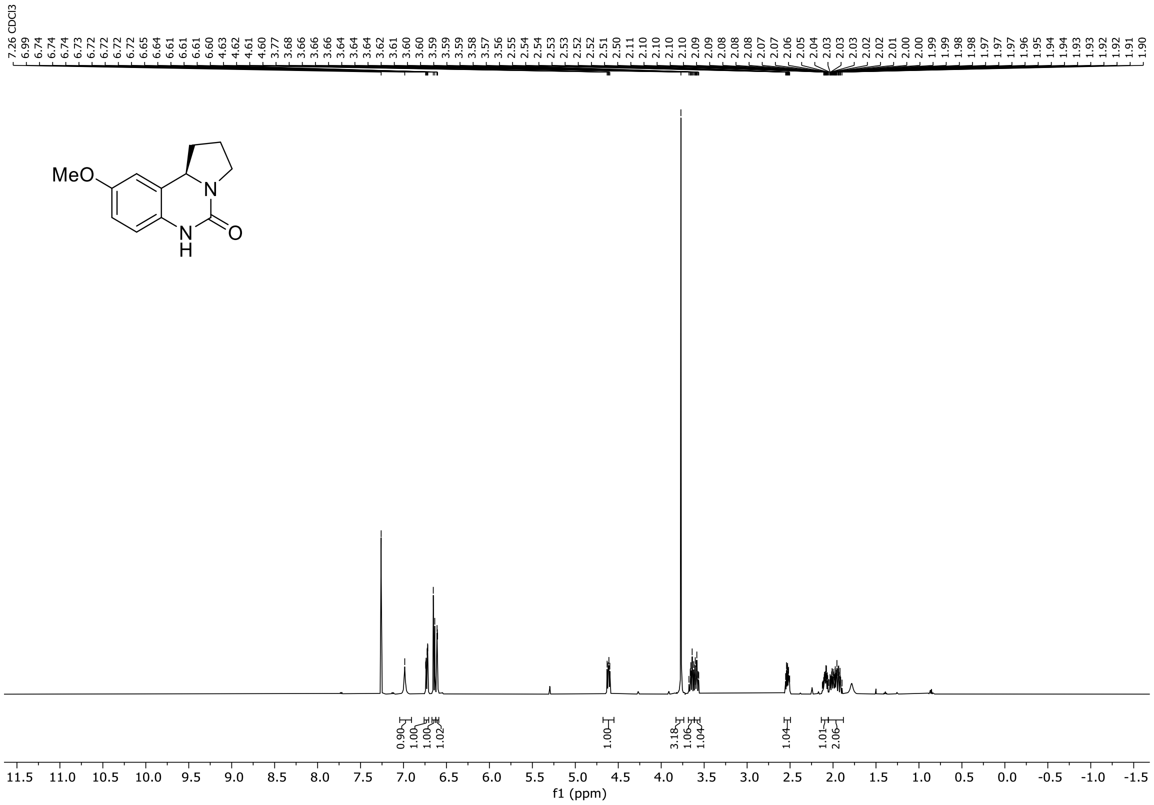

^{13}C NMR (126 MHz, $CDCl_3$):

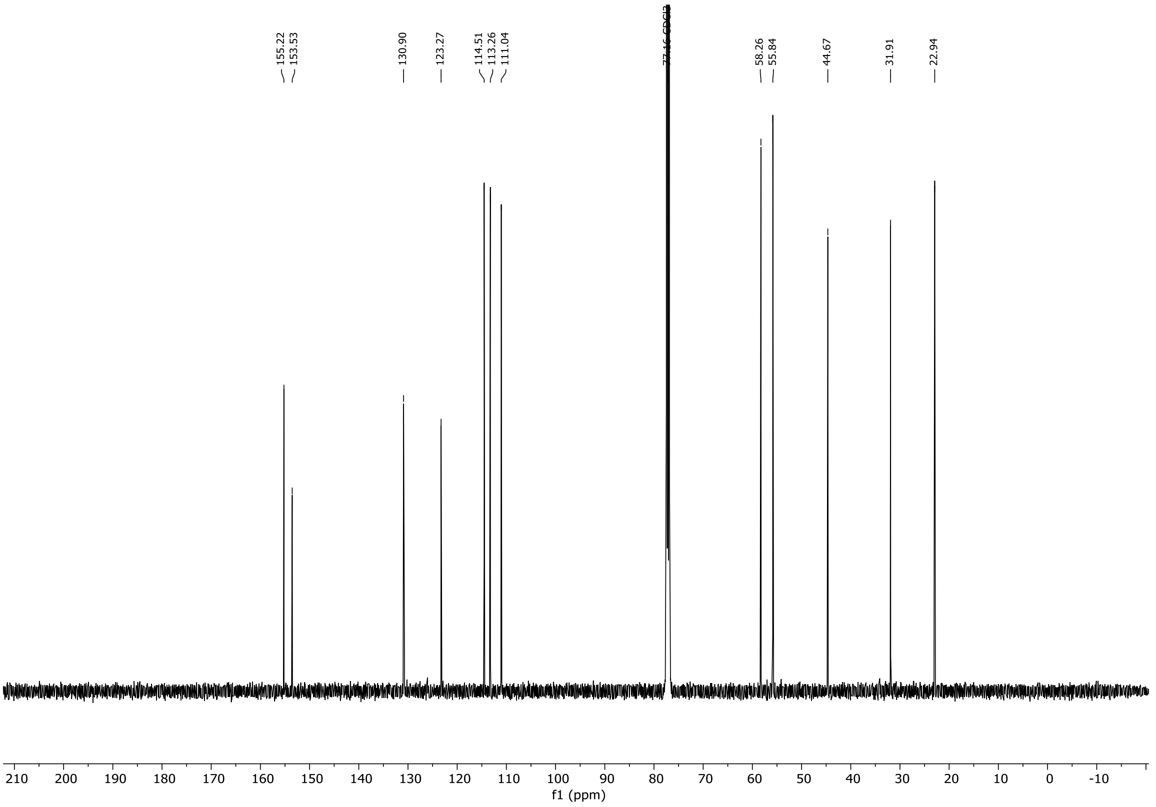

*10.5.13. 7-Methoxy-2,3,6,10b-tetrahydropyrrolo[1,2-c]quinazolin-5(1H)-one (**5m**)*

^{1}H NMR (500 MHz, $CDCl_3$):

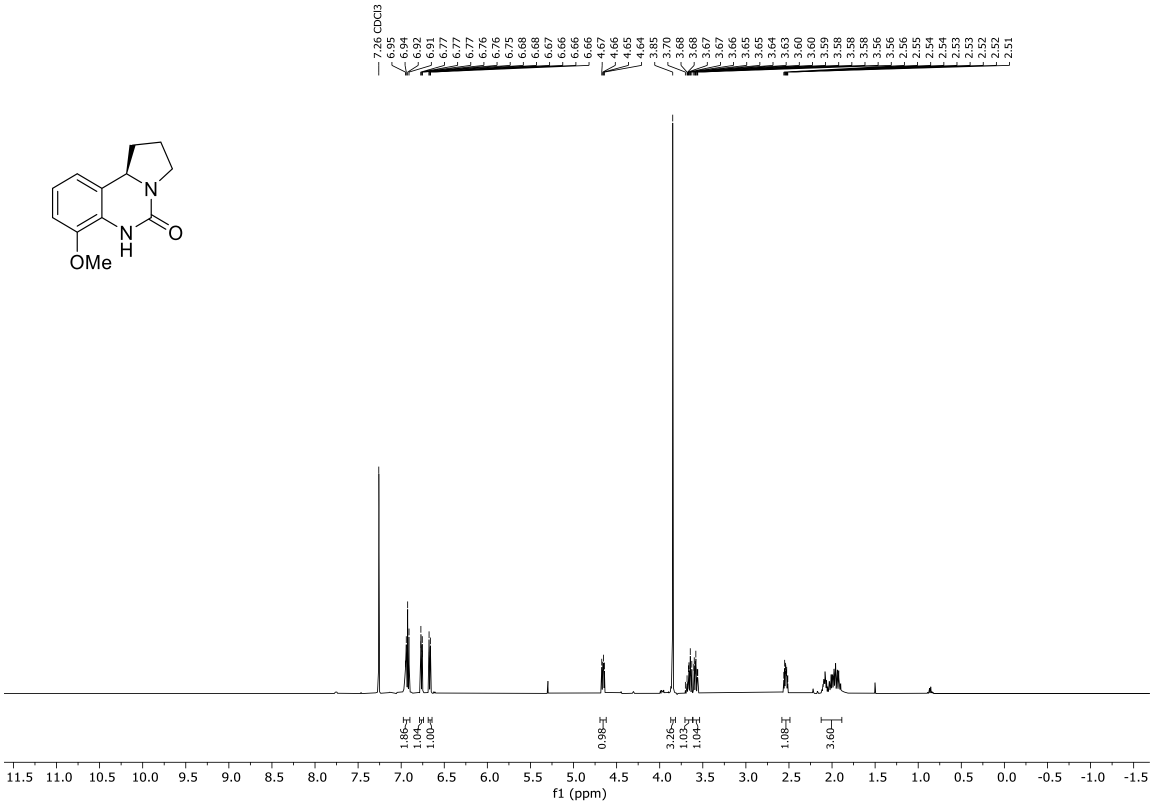

^{13}C NMR (126 MHz, $CDCl_3$):

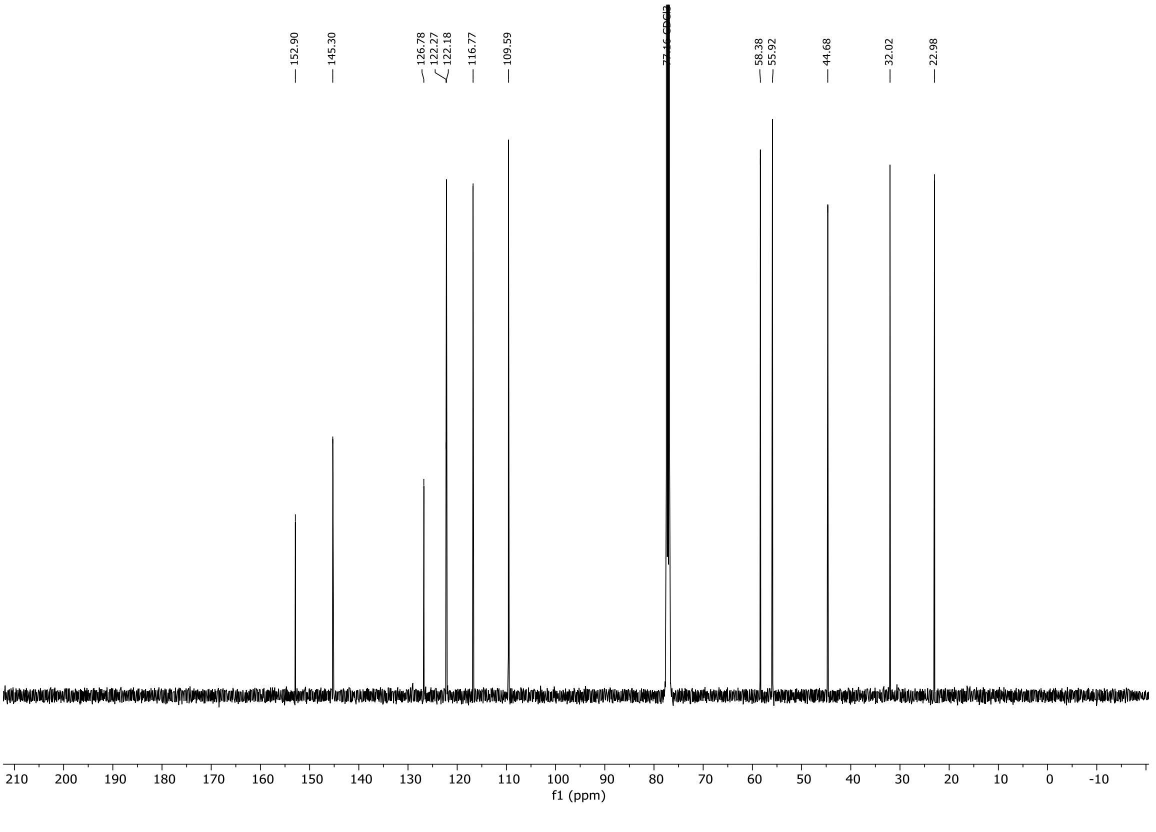

*10.5.14. 8-(Furan-3-yl)-2,3,6,10b-tetrahydropyrrolo[1,2-c]quinazolin-5(1H)-one (**5n**)*

^{1}H NMR (500 MHz, $CDCl_3$):

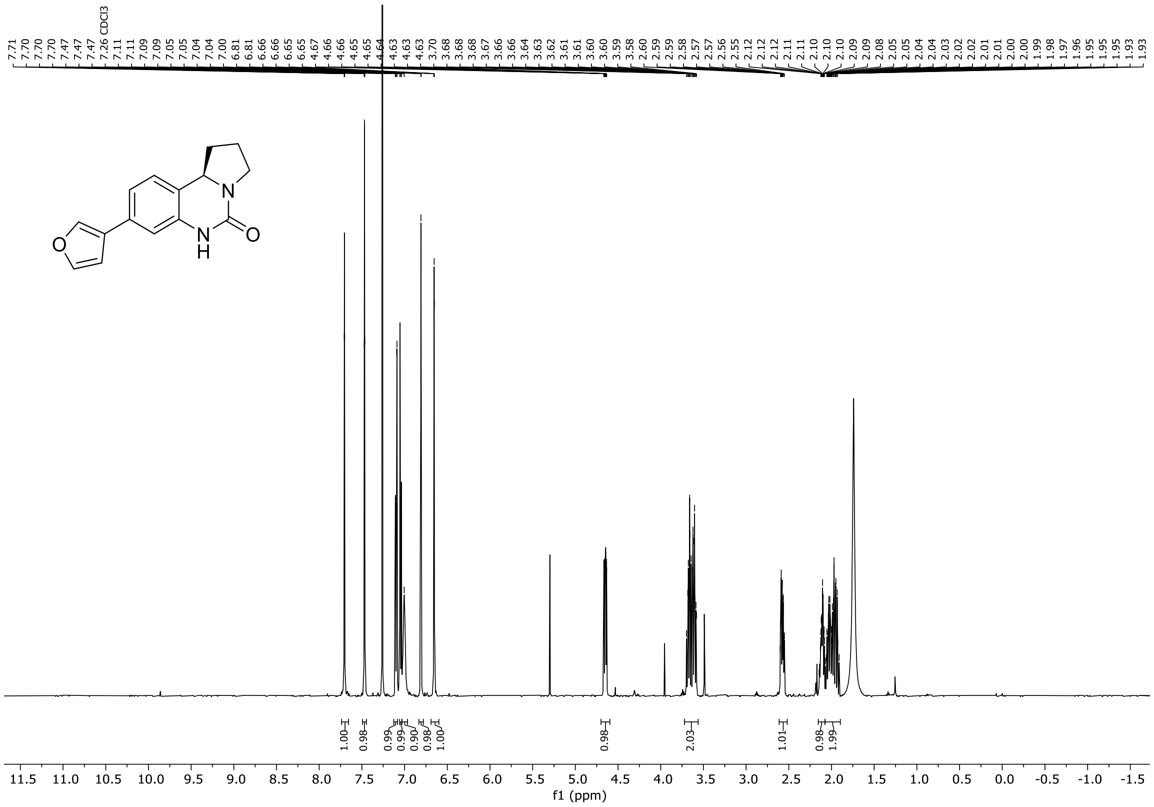

^{13}C NMR (126 MHz, $CDCl_3$):

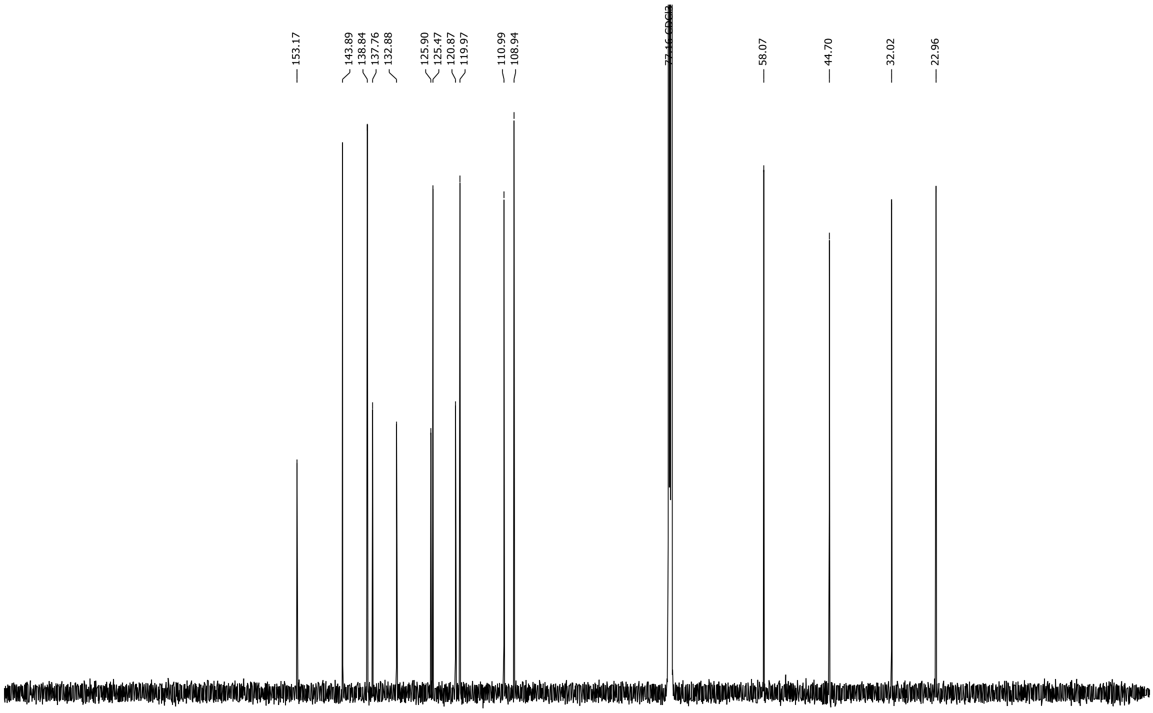

*10.5.15. 8-(Thiophen-3-yl)-2,3,6,10b-tetrahydropyrrolo[1,2-c]quinazolin-5(1H)-one (**5o**)*

^{1}H NMR (500 MHz, $CDCl_3$):

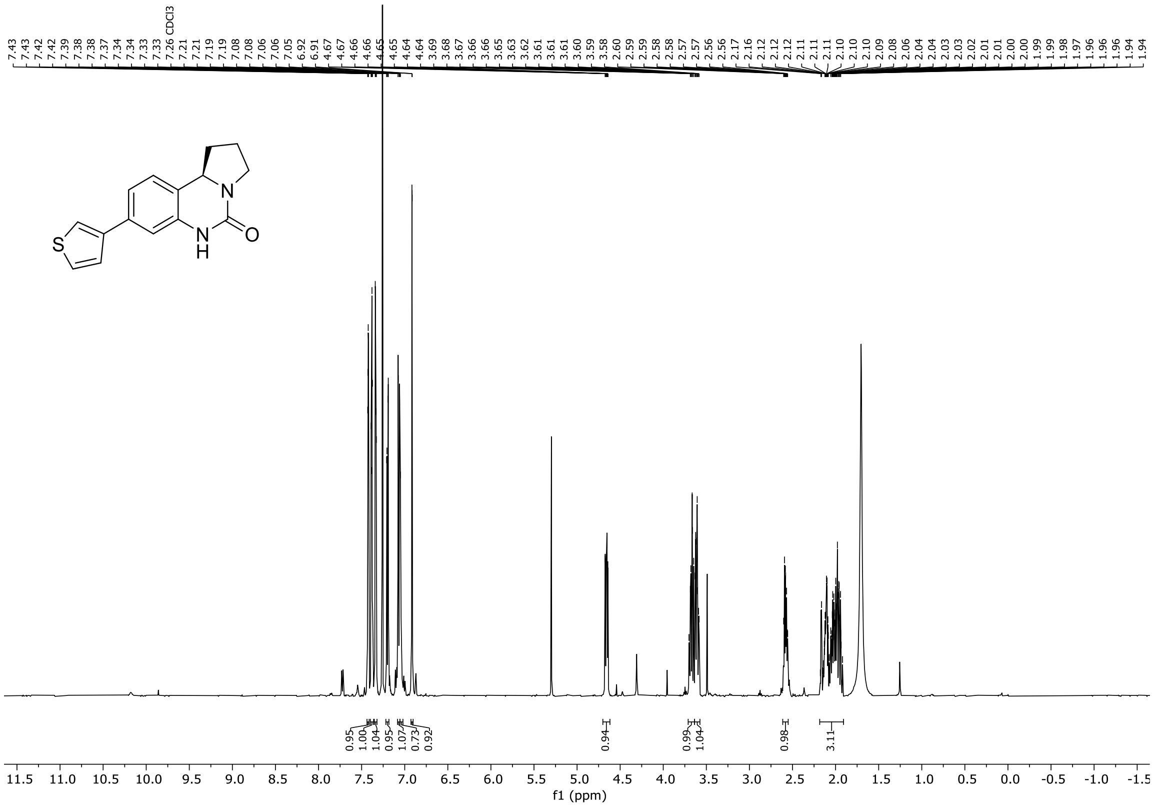

^{13}C NMR (126 MHz, $CDCl_3$):

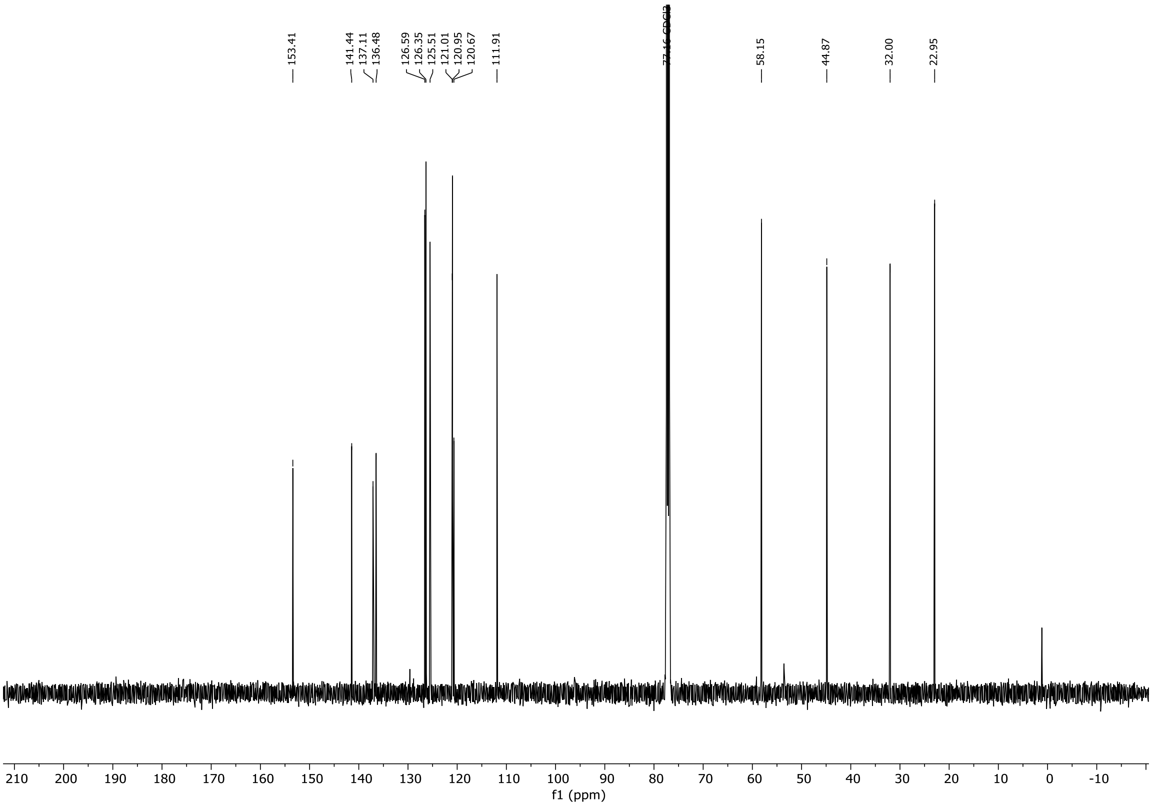

10.5.16. tert-Butyl 2-(5-oxo-1,2,3,5,6,10b-hexahydropyrrolo[1,2-c]quinazolin-8-yl)-1H-pyrrole-1-carboxylate (5p)

^{1}H NMR (500 MHz, $CDCl_3$):

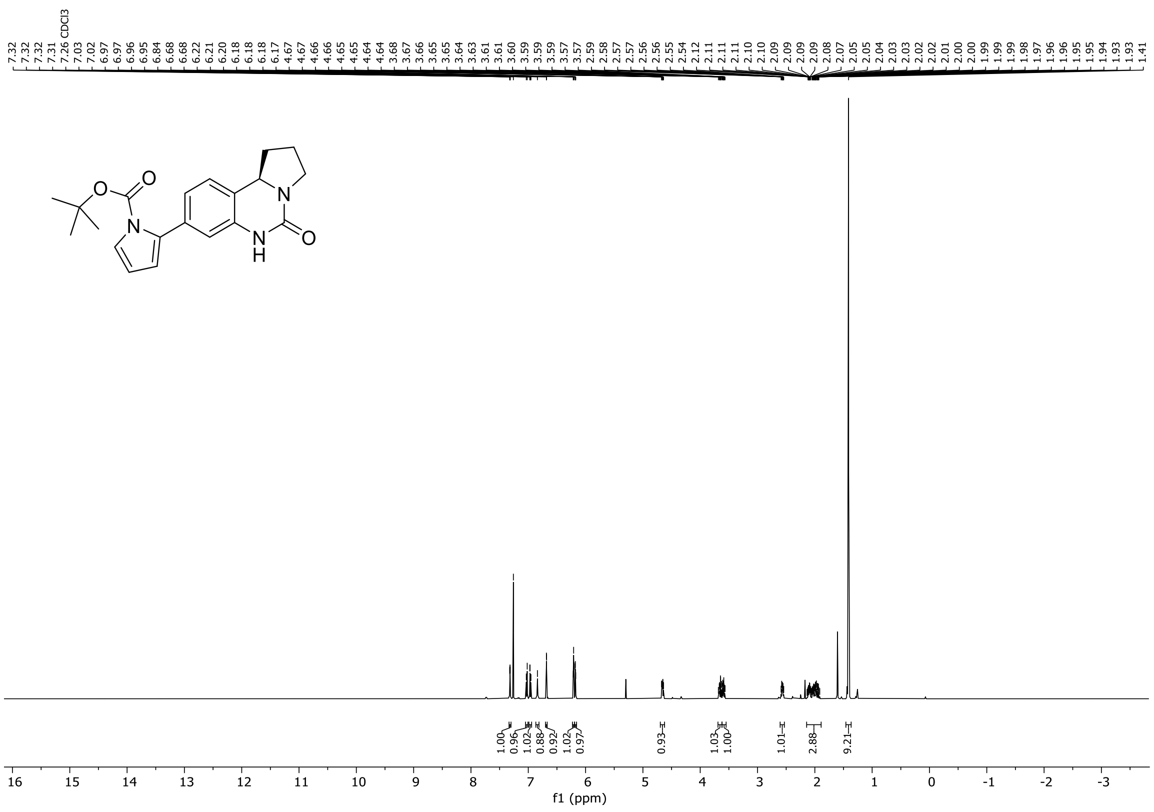

^{13}C NMR (126 MHz, $CDCl_3$):

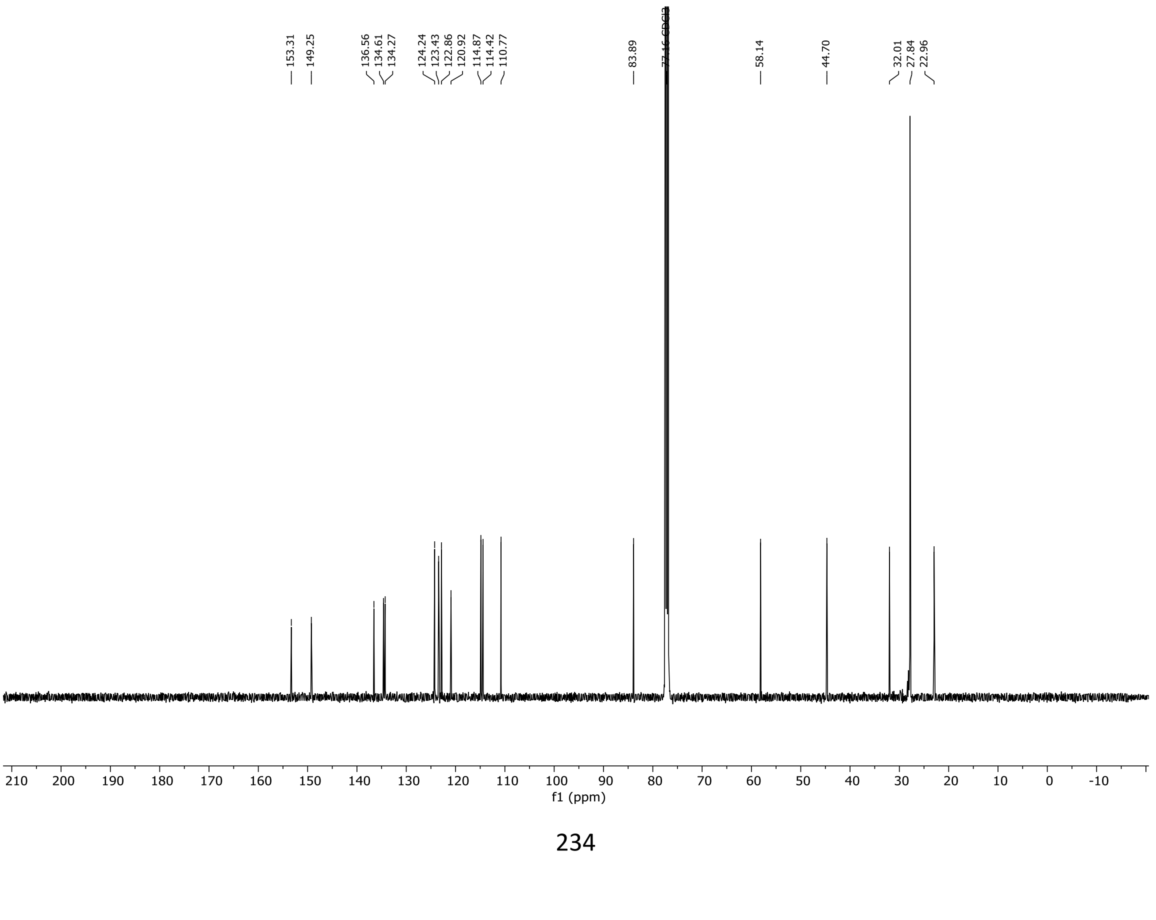

*10.5.17. 8-(Pyridin-3-yl)-2,3,6,10b-tetrahydropyrrolo[1,2-c]quinazolin-5(1H)-one (**5q**)*

^{1}H NMR (500 MHz, $CDCl_3$):

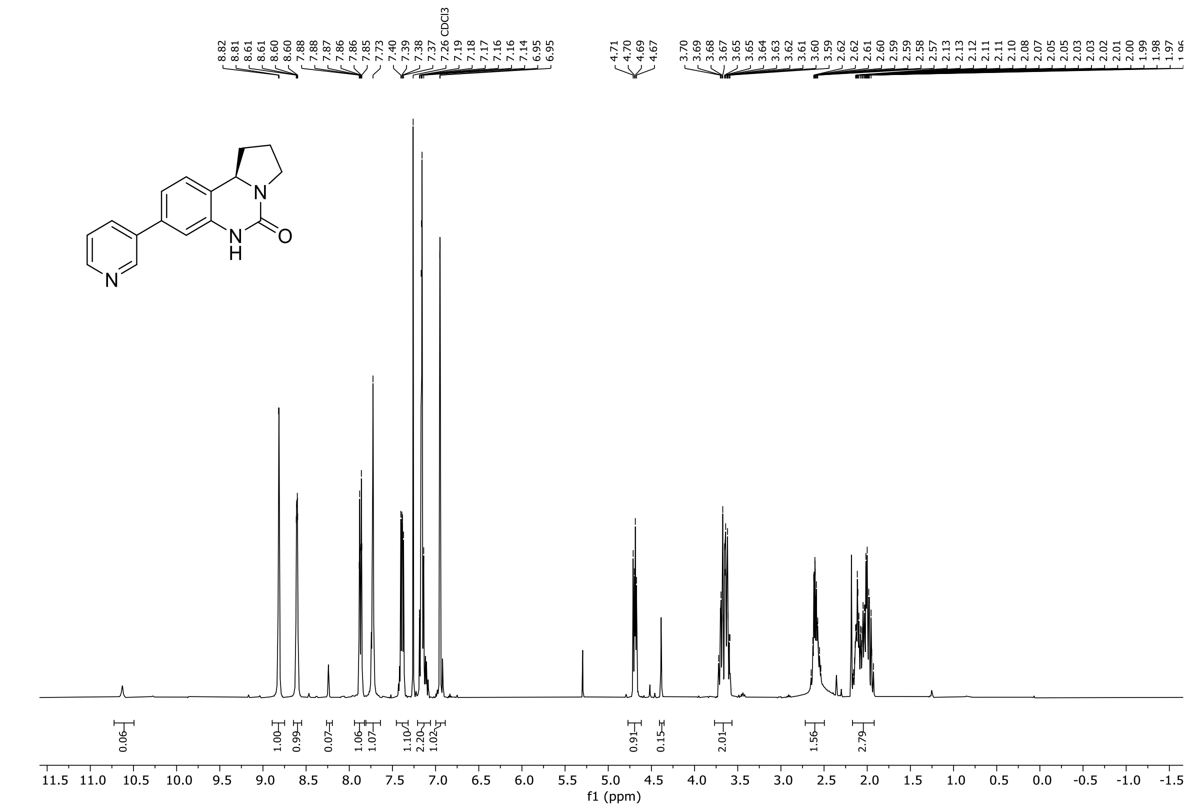

^{13}C NMR (126 MHz, $CDCl_3$):

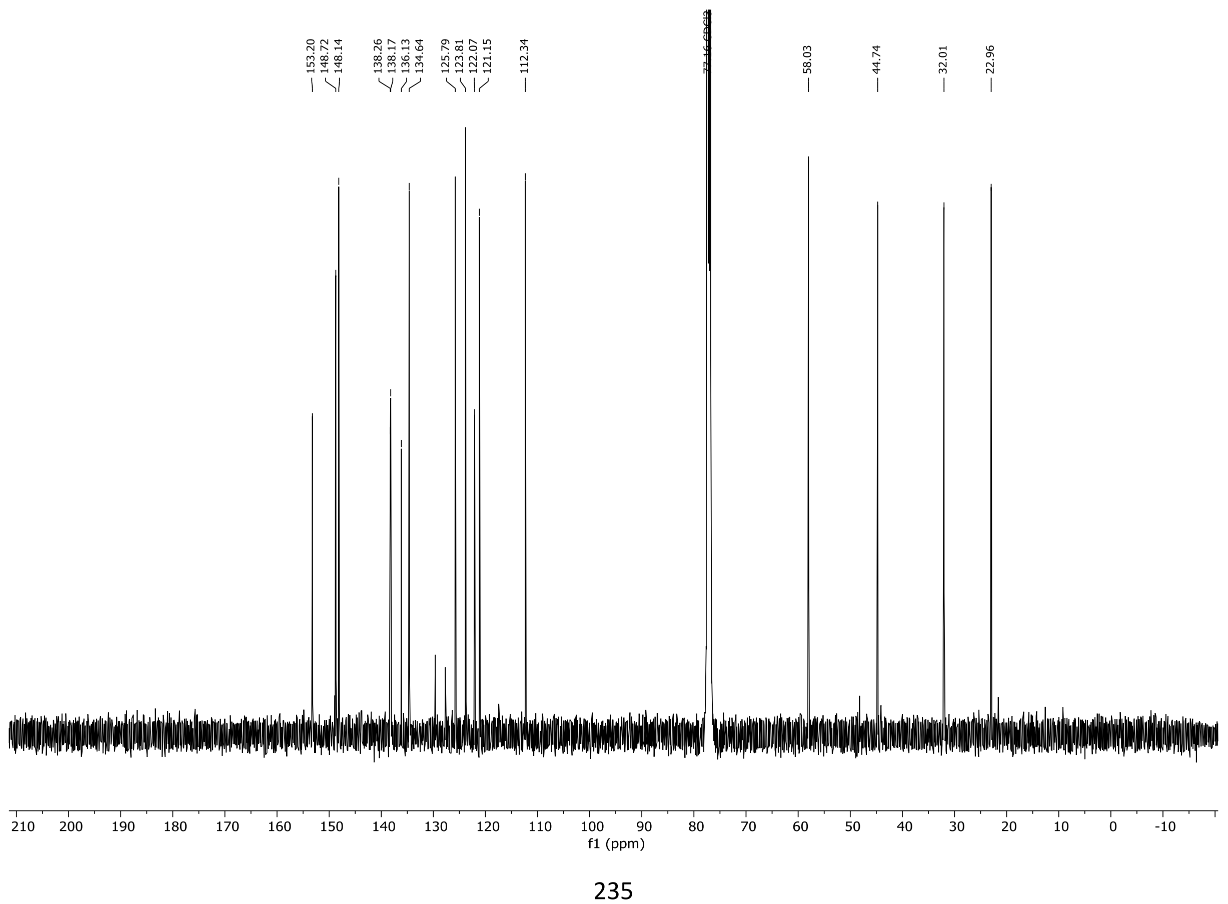

*10.5.18. Ethyl (E)-3-(5-oxo-1,2,3,5,6,10b-hexahydropyrrolo[1,2-c]quinazolin-8-yl)acrylate (**5r**)*

^{1}H NMR (500 MHz, $CDCl_3$):

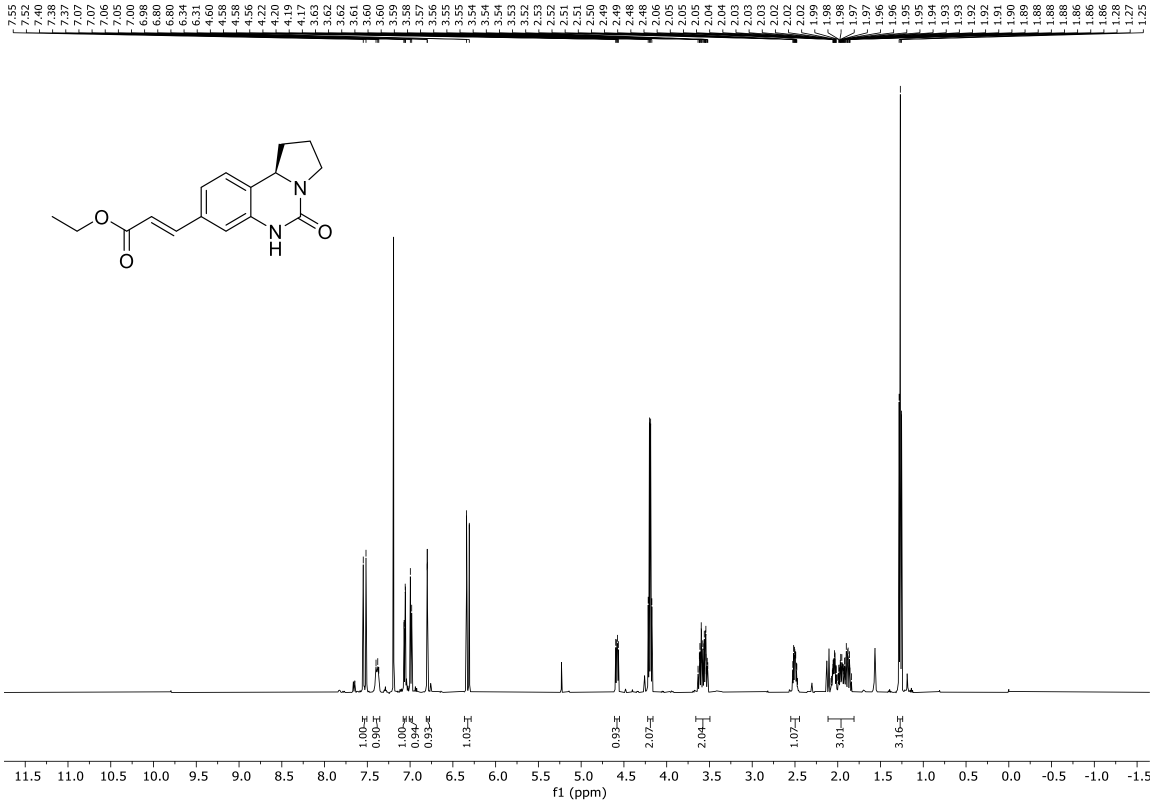

^{13}C NMR (126 MHz, $CDCl_3$):

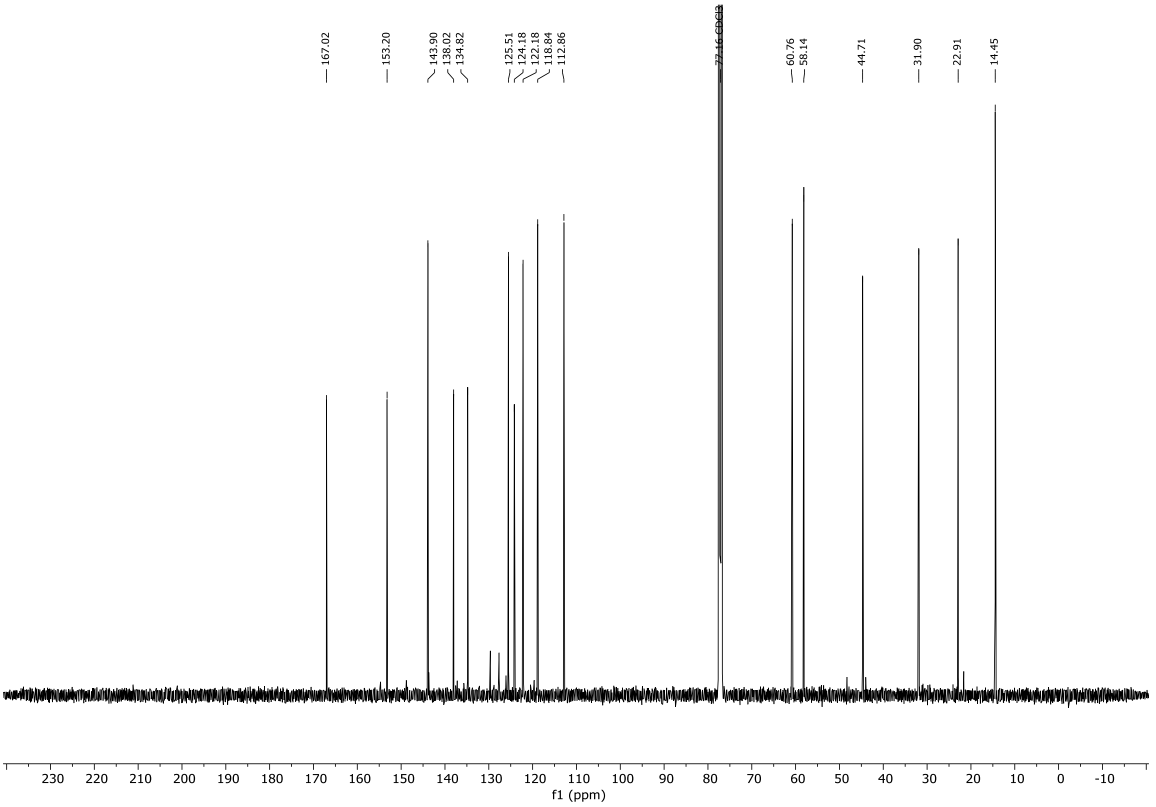

*10.5.19. 9-(4-Methoxyphenyl)-2,3,6,10b-tetrahydropyrrolo[1,2-c]quinazolin-5(1H)-one (**5s**)*

^{1}H NMR (500 MHz, $CDCl_3$):

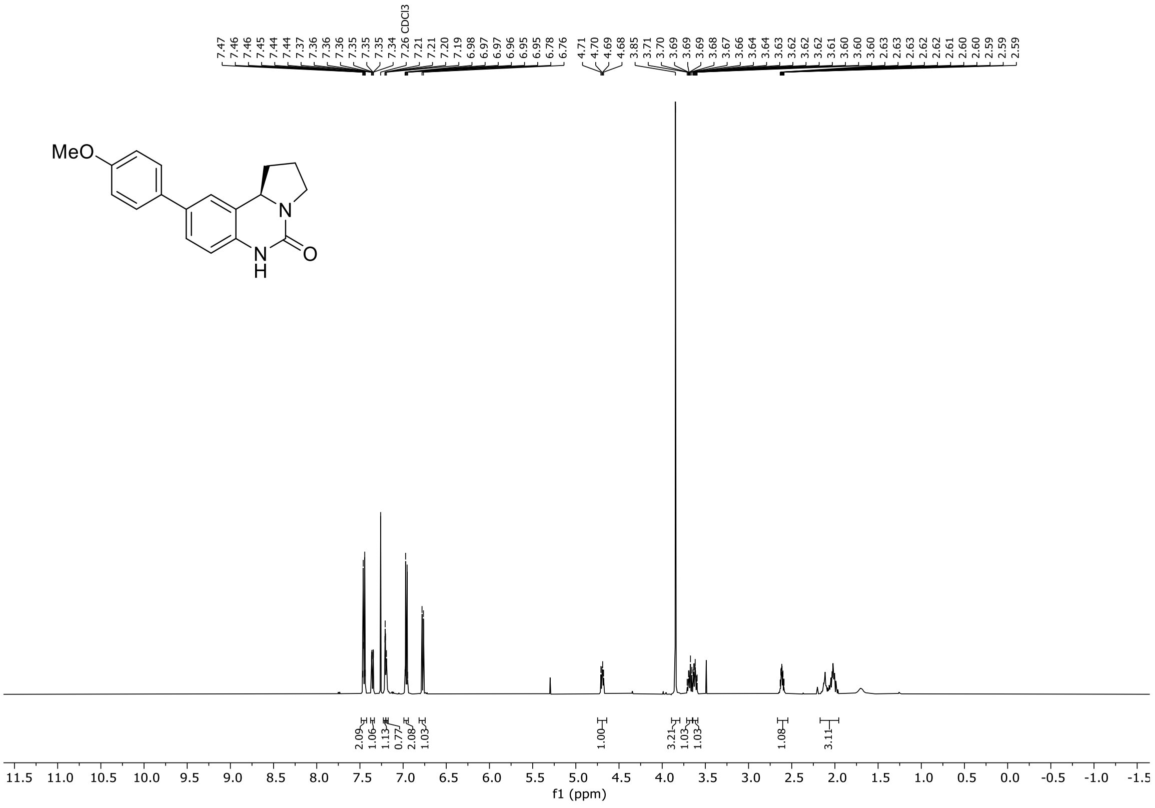

^{13}C NMR (126 MHz, $CDCl_3$):

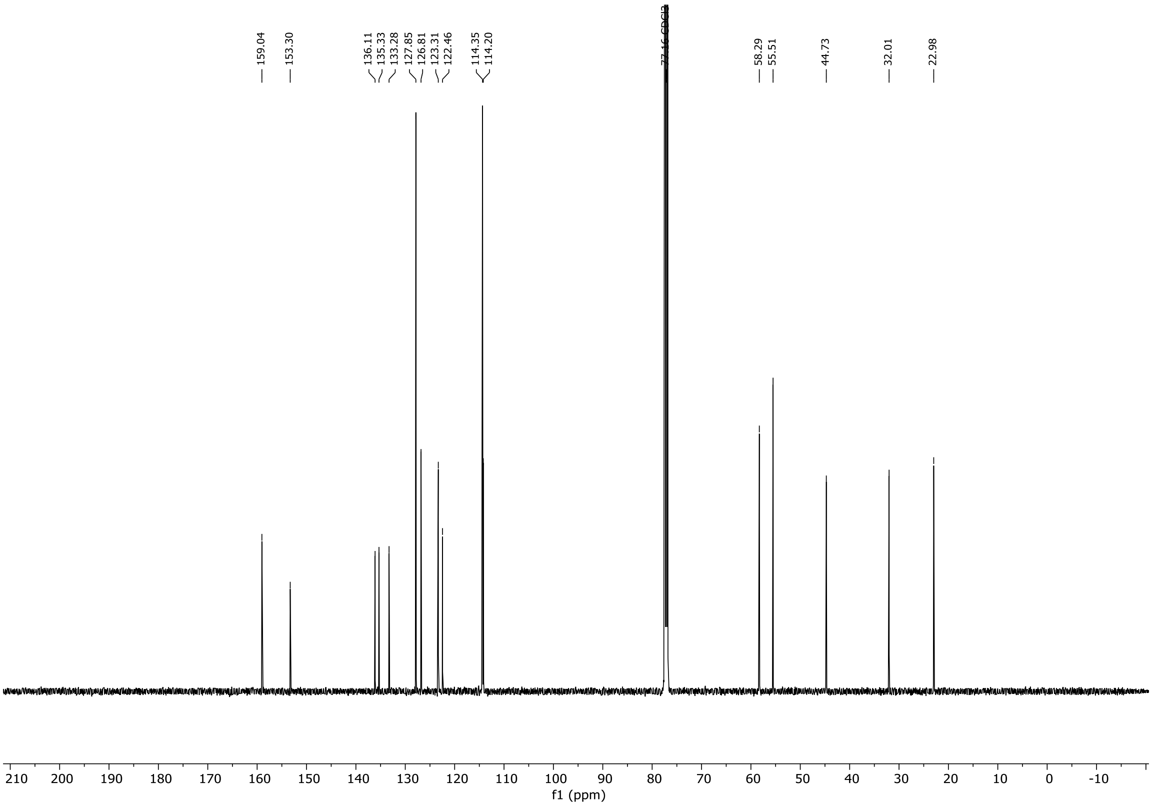

*10.5.20. 8,9,10,10a-Tetrahydropyrido[2,3-e]pyrrolo[1,2-c]pyrimidin-6(5H)-one (**5t**)*

^{1}H NMR (500 MHz, $CDCl_3$):

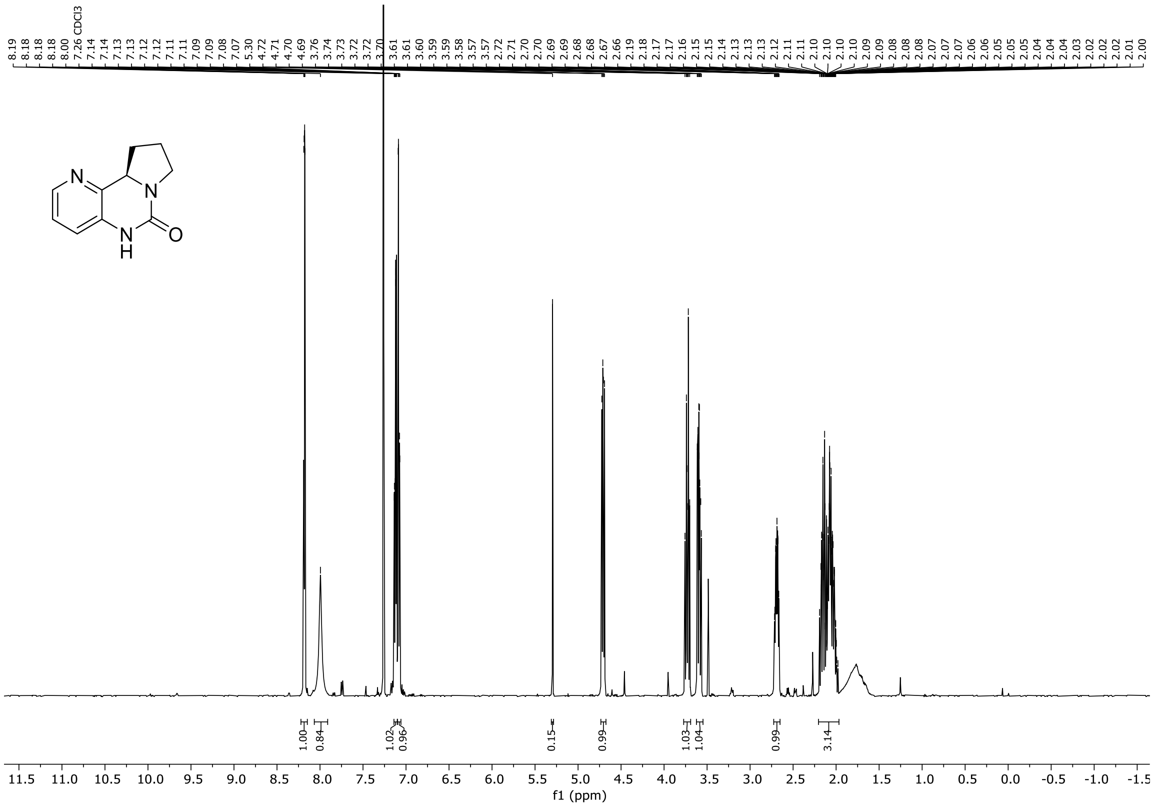

^{13}C NMR (126 MHz, $CDCl_3$):

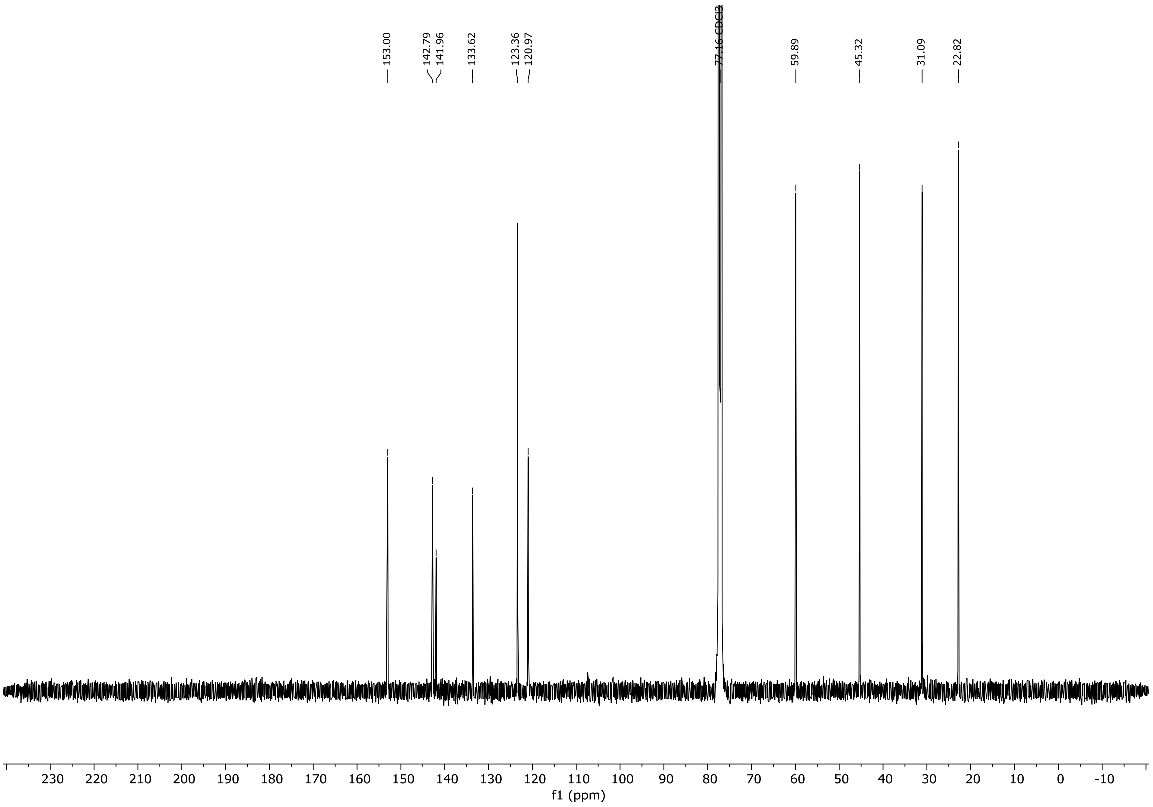

*10.5.21. 2,2-Dimethyl-2,3,6,10b-tetrahydropyrrolo[1,2-c]quinazolin-5(1H)-one (**5u**)*

^{1}H NMR (500 MHz, $CDCl_3$):

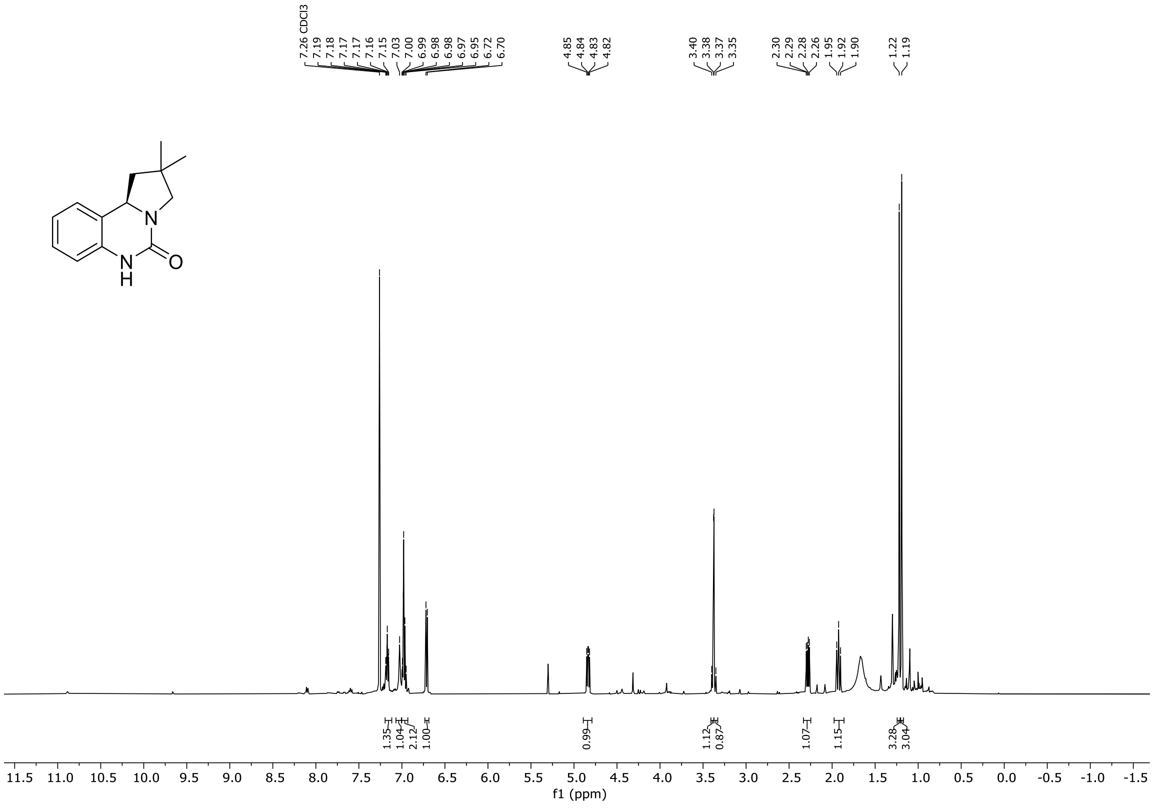

^{13}C NMR (126 MHz, $CDCl_3$):

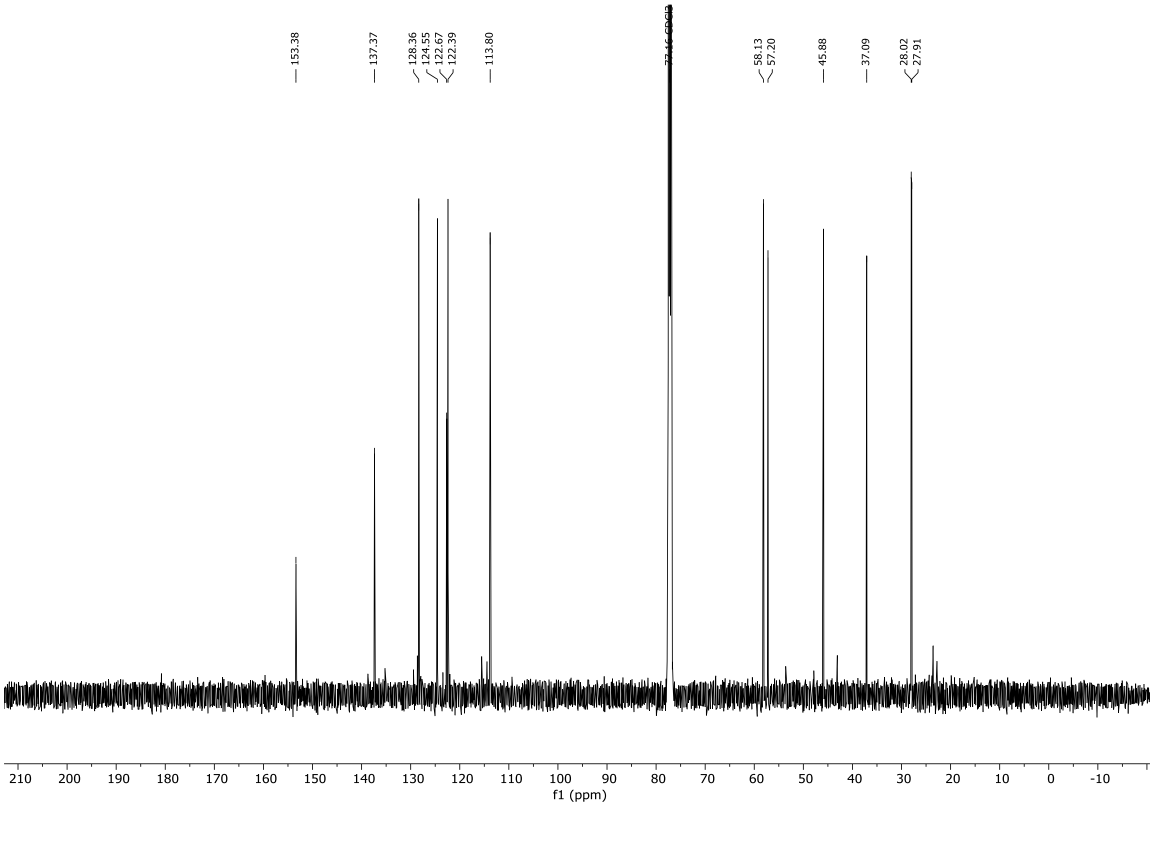

*10.5.22. 8,9,10,10a-Tetrahydropyrido[3,2-e]pyrrolo[1,2-c]pyrimidin-6(5H)-one (**5v**)*

^{1}H NMR (500 MHz, $CDCl_3$):

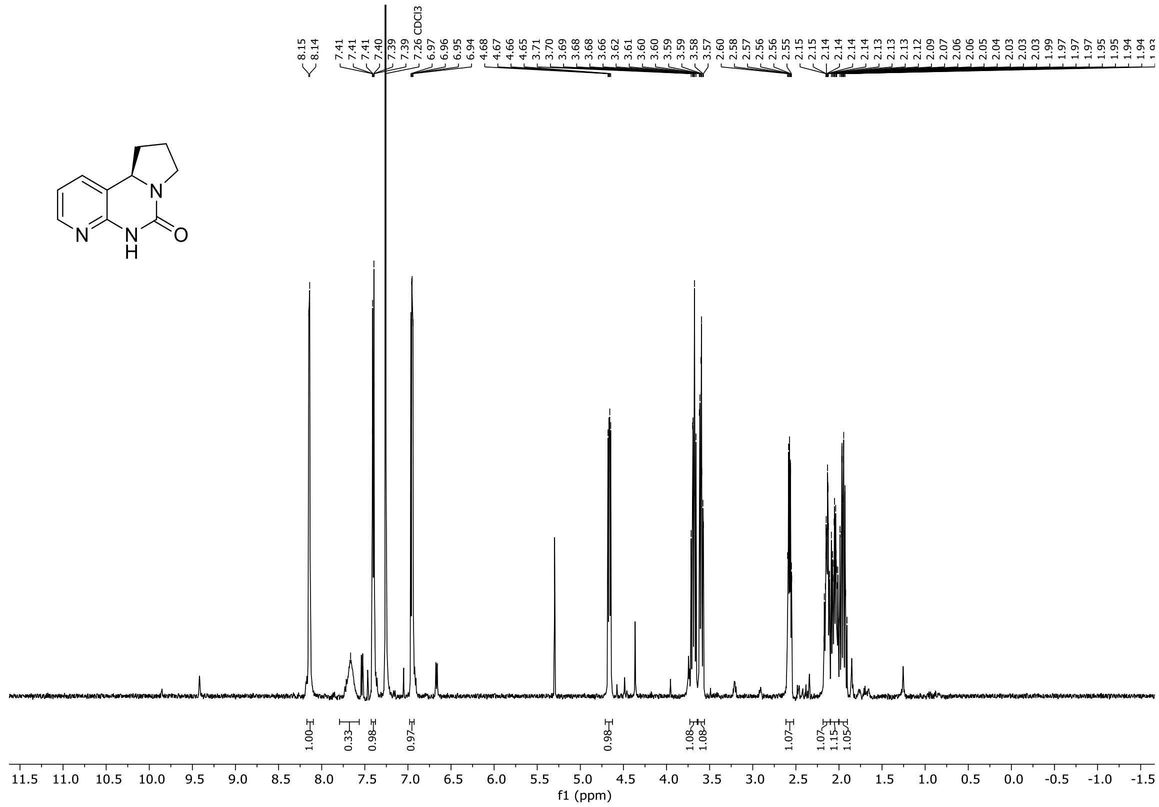

^{13}C NMR (126 MHz, $CDCl_3$):

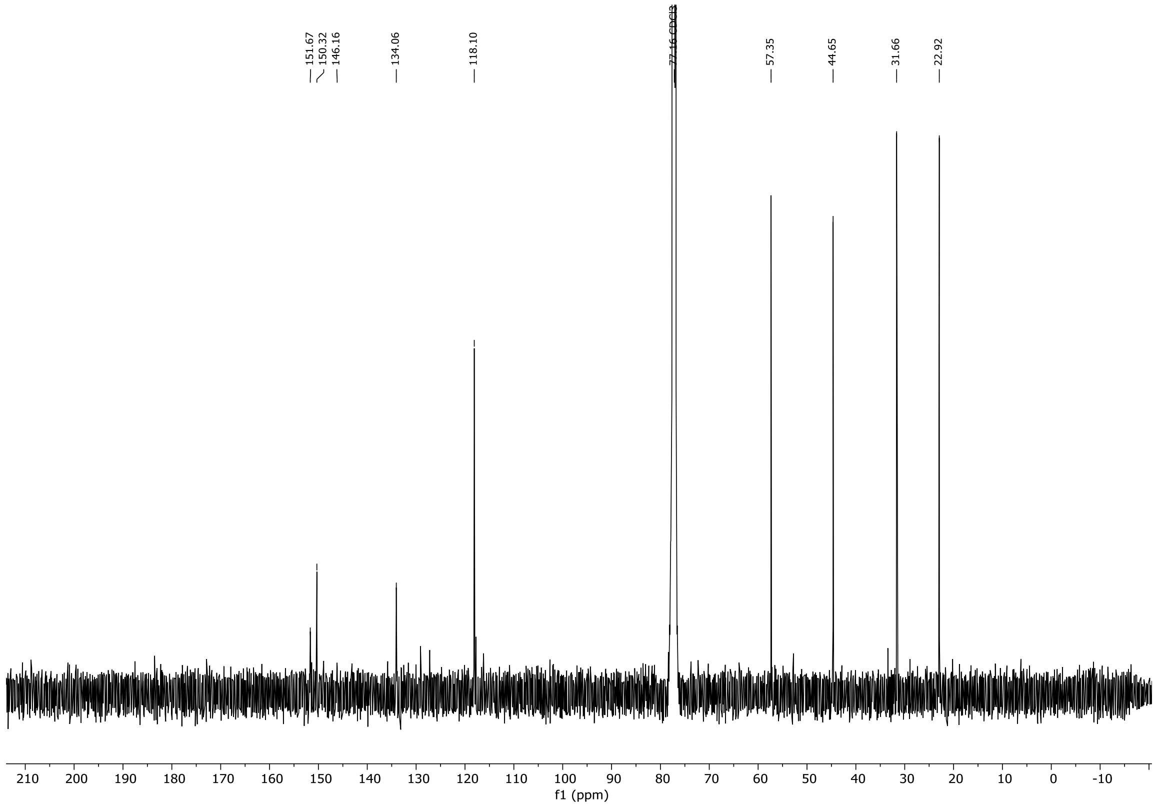

*10.5.23. 5,8,9,10,11,11a-Hexahydro-6H-pyrido[1,2-c]quinazolin-6-one (**5aa**)*

^{1}H NMR (500 MHz, $CDCl_3$):

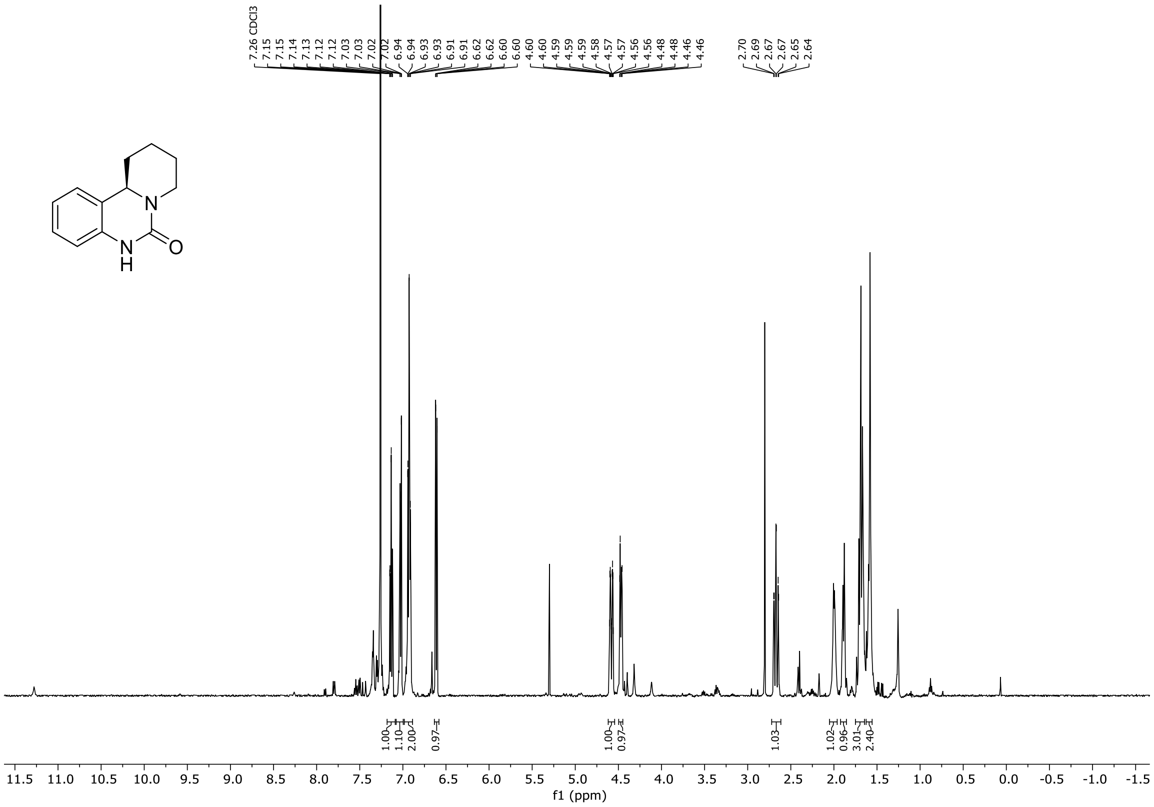

^{13}C NMR (126 MHz, $CDCl_3$):

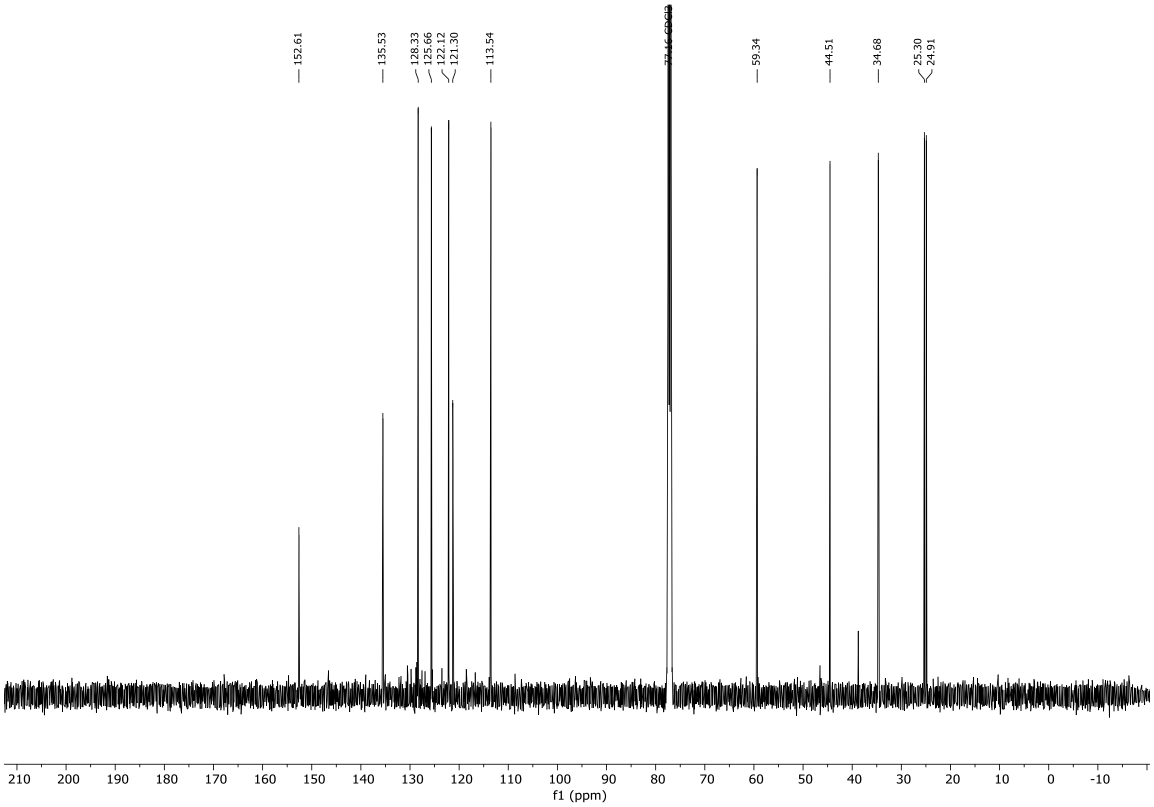

*10.5.24. 5,8,13,13a-Tetrahydro-6H-isoquinolino[2,3-c]quinazolin-6-one (**5ab**)*

^{1}H NMR (500 MHz, $CDCl_3$):

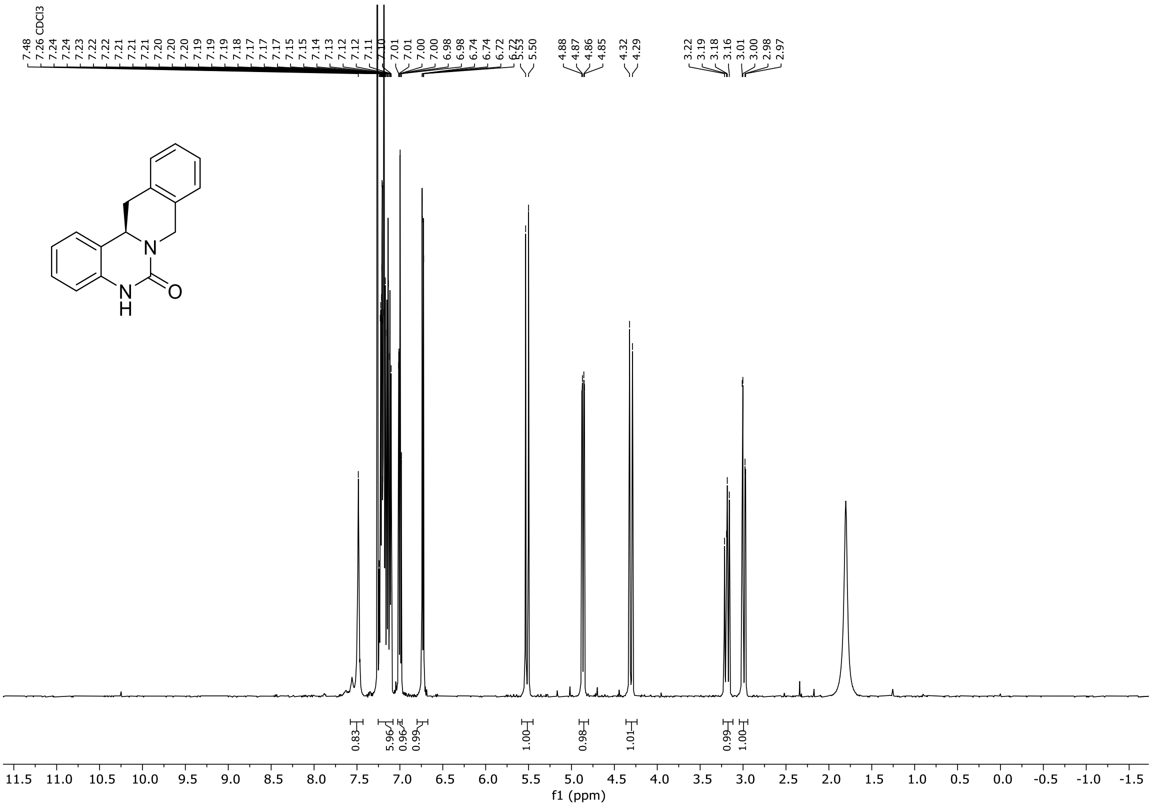

^{13}C NMR (126 MHz, $CDCl_3$):

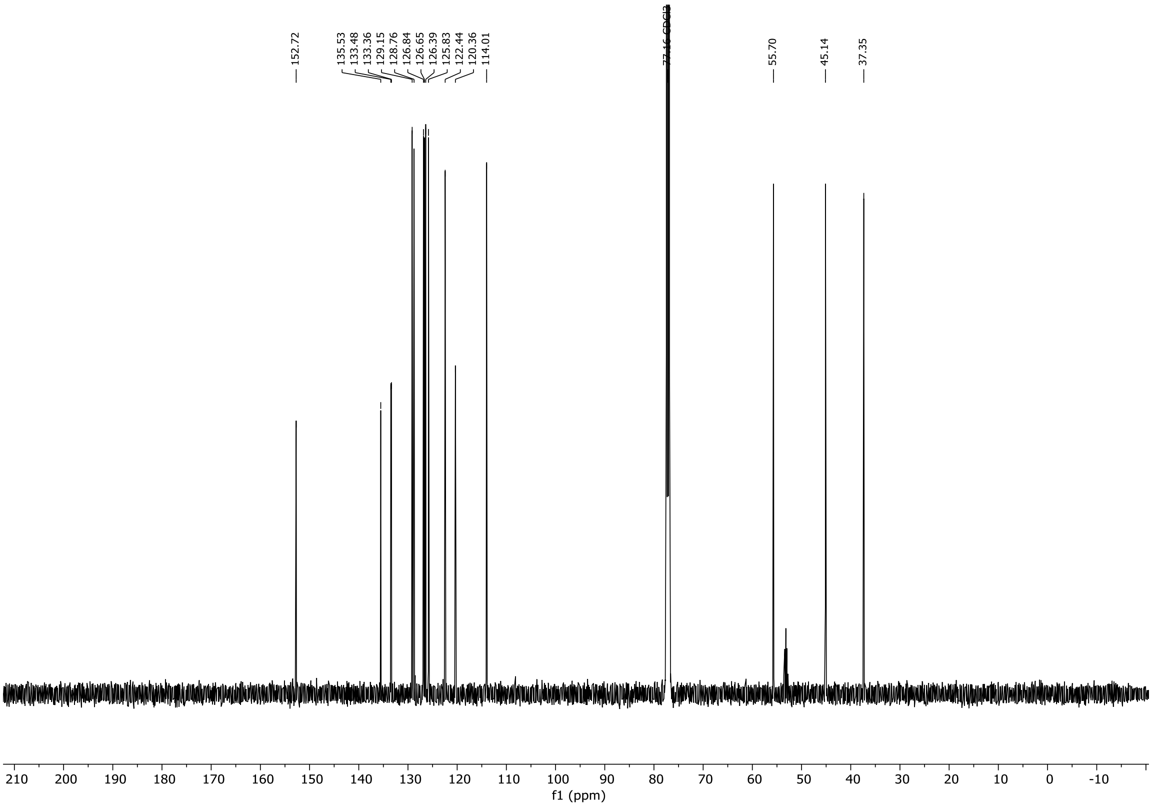

*10.5.25. 9,9-Dimethyl-5,8,9,10,11,11a-hexahydro-6H-pyrido[1,2-c]quinazolin-6-one (**5ac**)*

^{1}H NMR (500 MHz, $CDCl_3$):

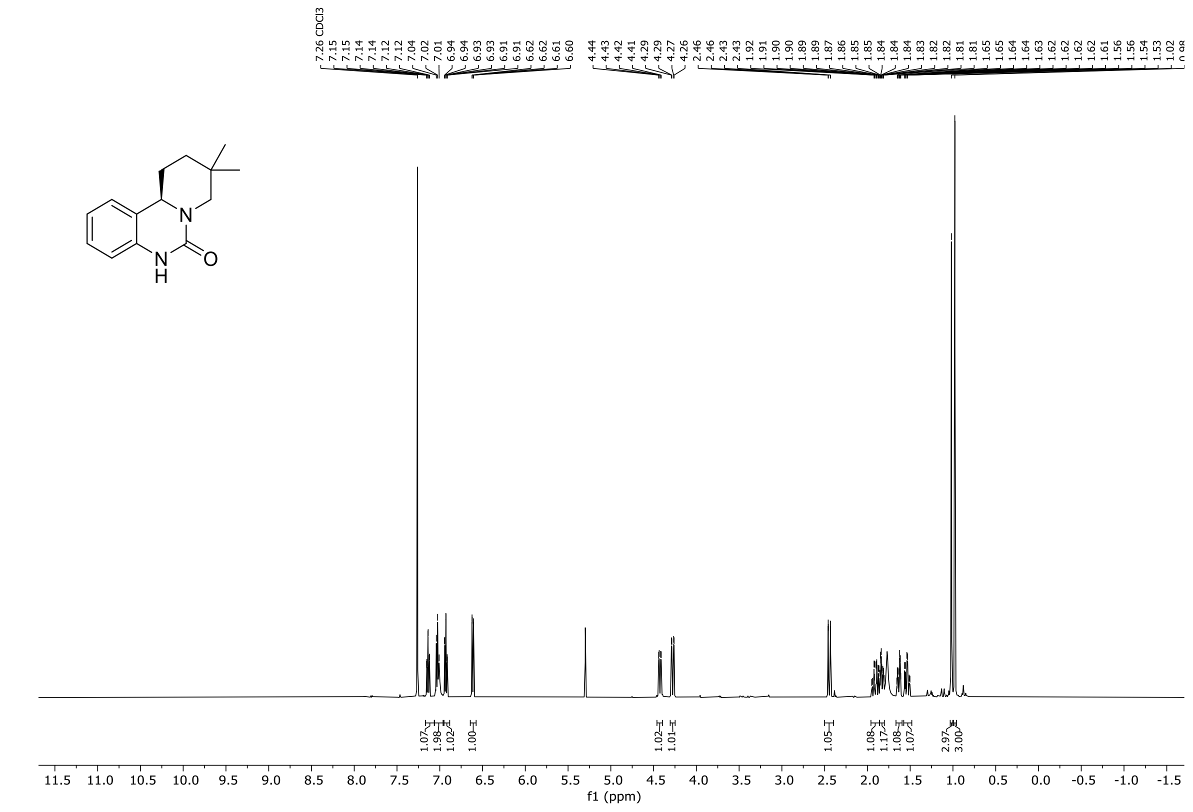

^{13}C NMR (126 MHz, $CDCl_3$):

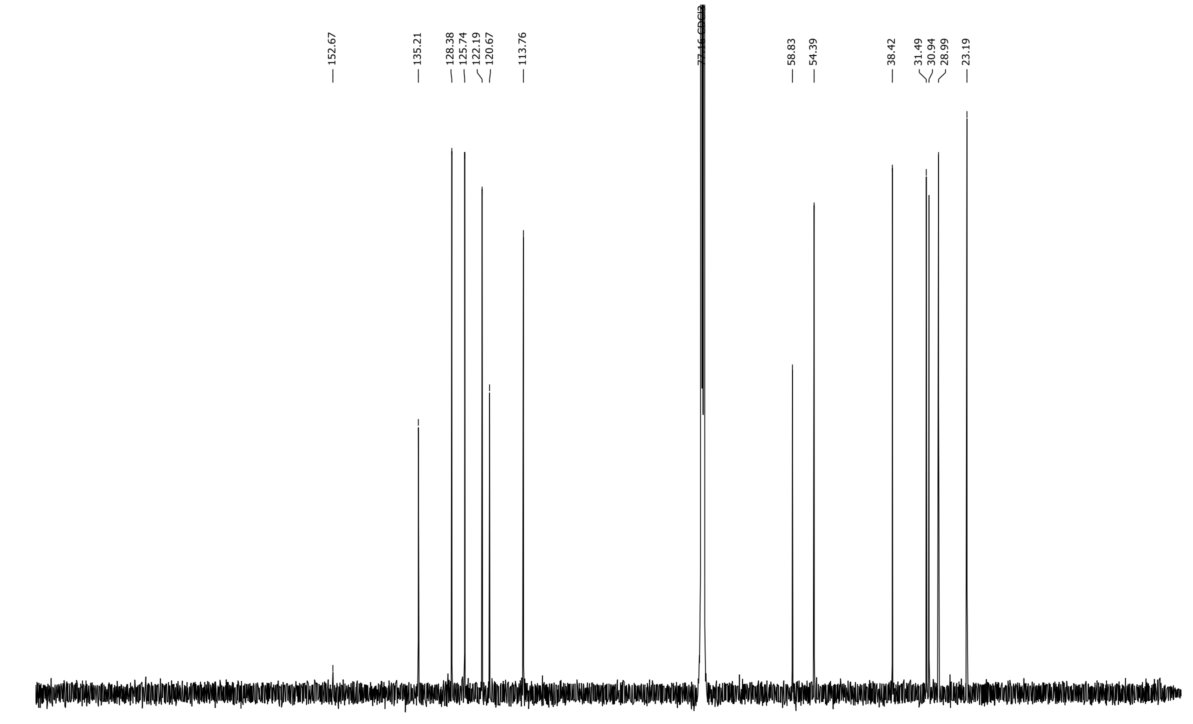

*10.5.26. 8,9,14,14a-Tetrahydrobenzo[4,5]azepino[1,2-c]quinazolin-6(5H)-one (**5ba**)*

^{1}H NMR (500 MHz, $CDCl_3$):

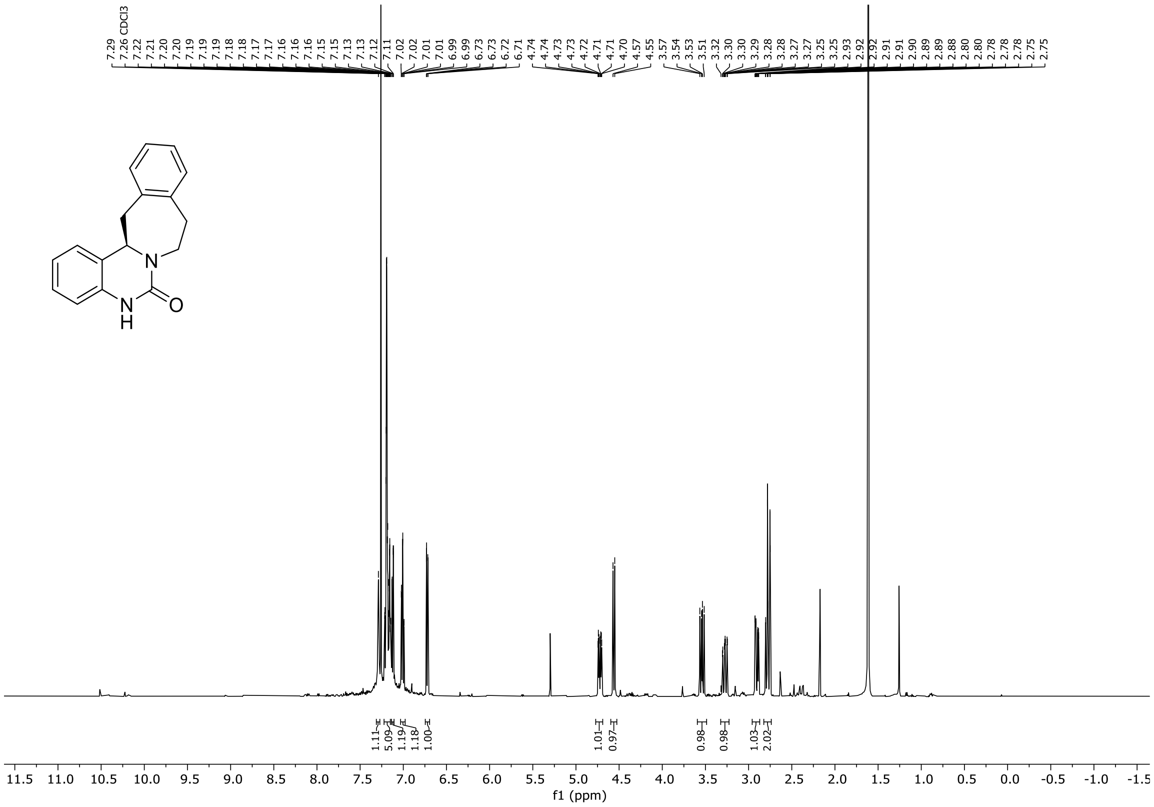

^{13}C NMR (126 MHz, $CDCl_3$):

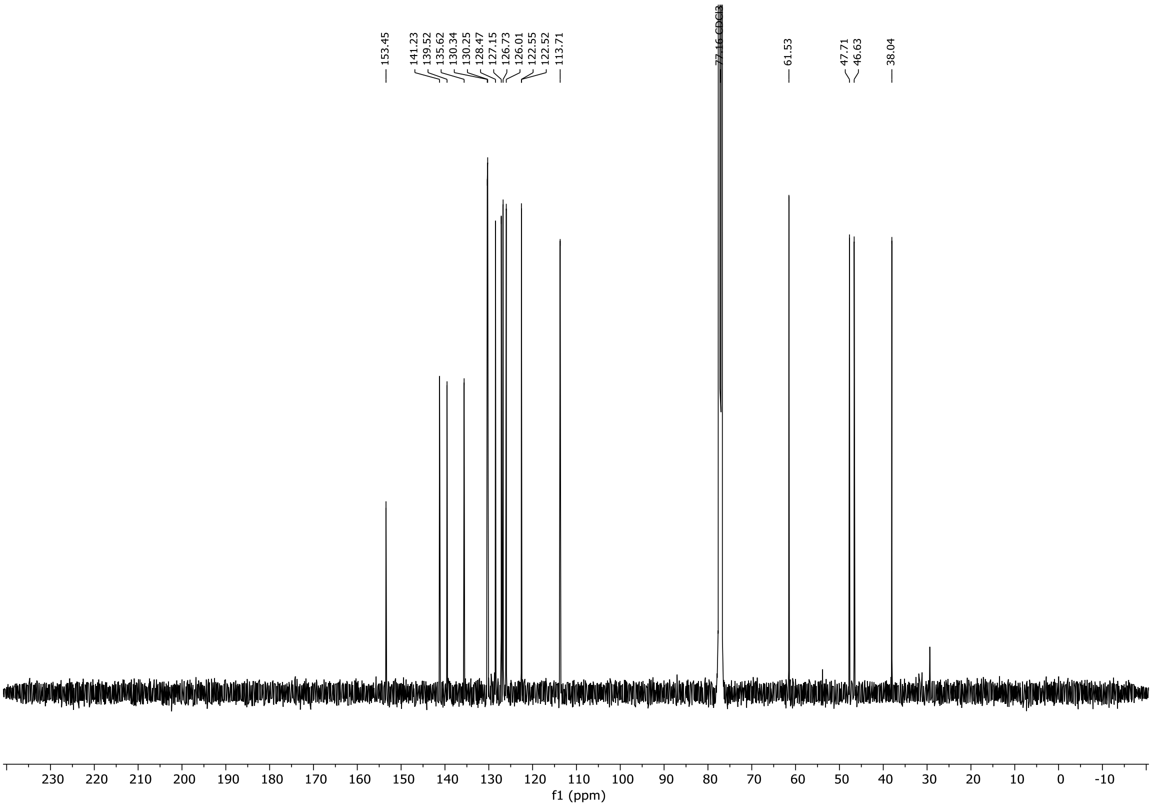

*10.5.27. 8,9,15,15a-Tetrahydro-[1,3]dioxolo[4,5:4,5]benzo[1,2:4,5]azepino[1,2-c]quinazolin-6(5H)-one (**5bb**)*

^{1}H NMR (500 MHz, $CDCl_3$):

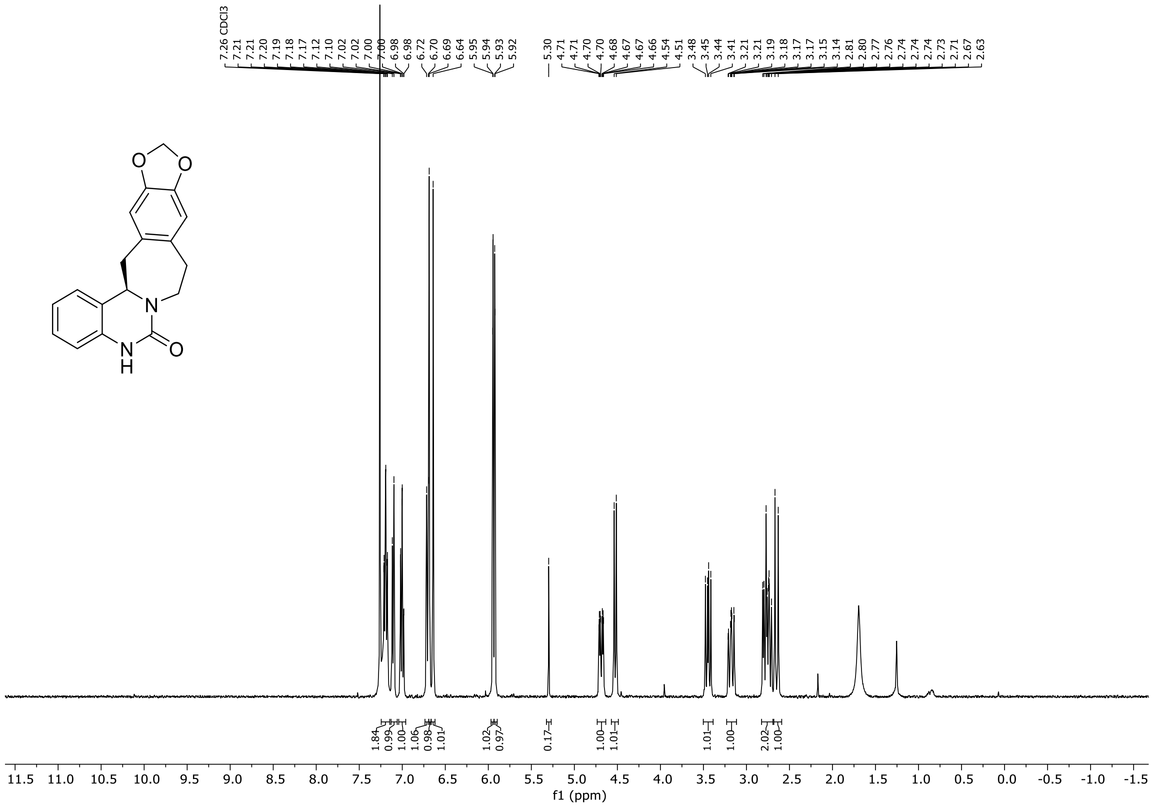

^{13}C NMR (126 MHz, $CDCl_3$):

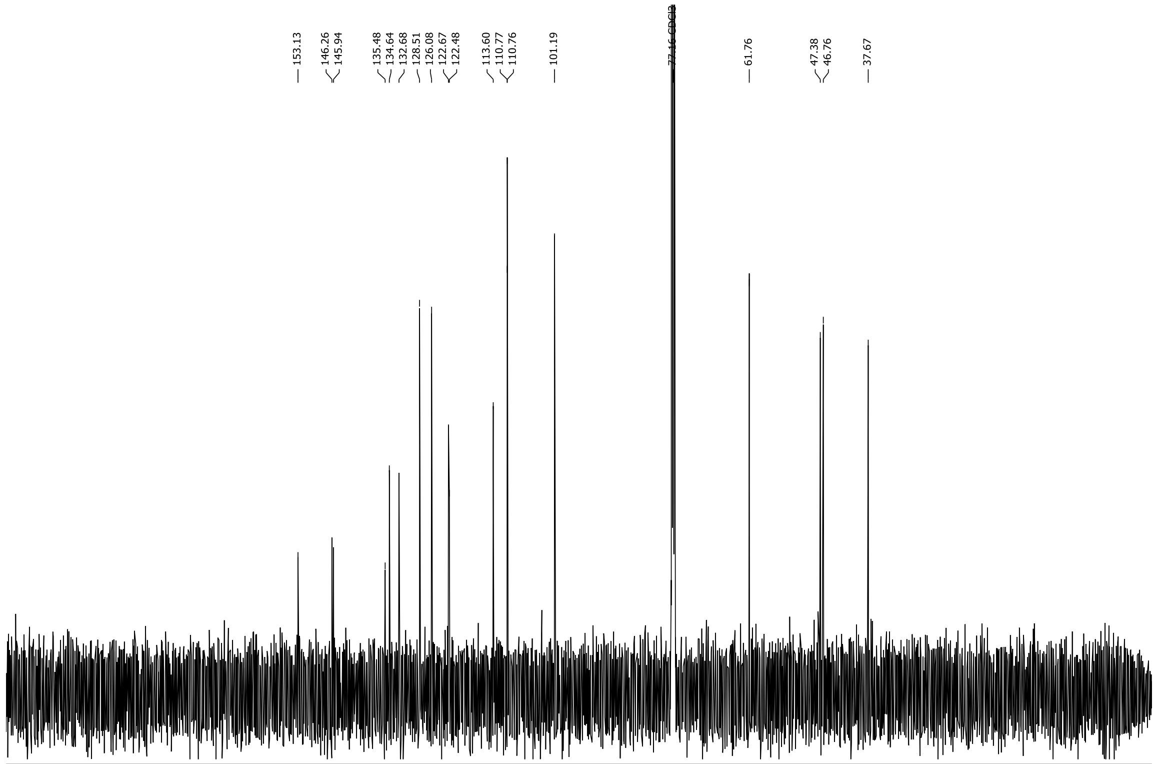

*10.5.28. 11,12-Dimethoxy-8,9,14,14a-tetrahydrobenzo[4,5]azepino[1,2-c]quinazolin-6(5H)-one (**5bc**)*

^{1}H NMR (500 MHz, $CDCl_3$):

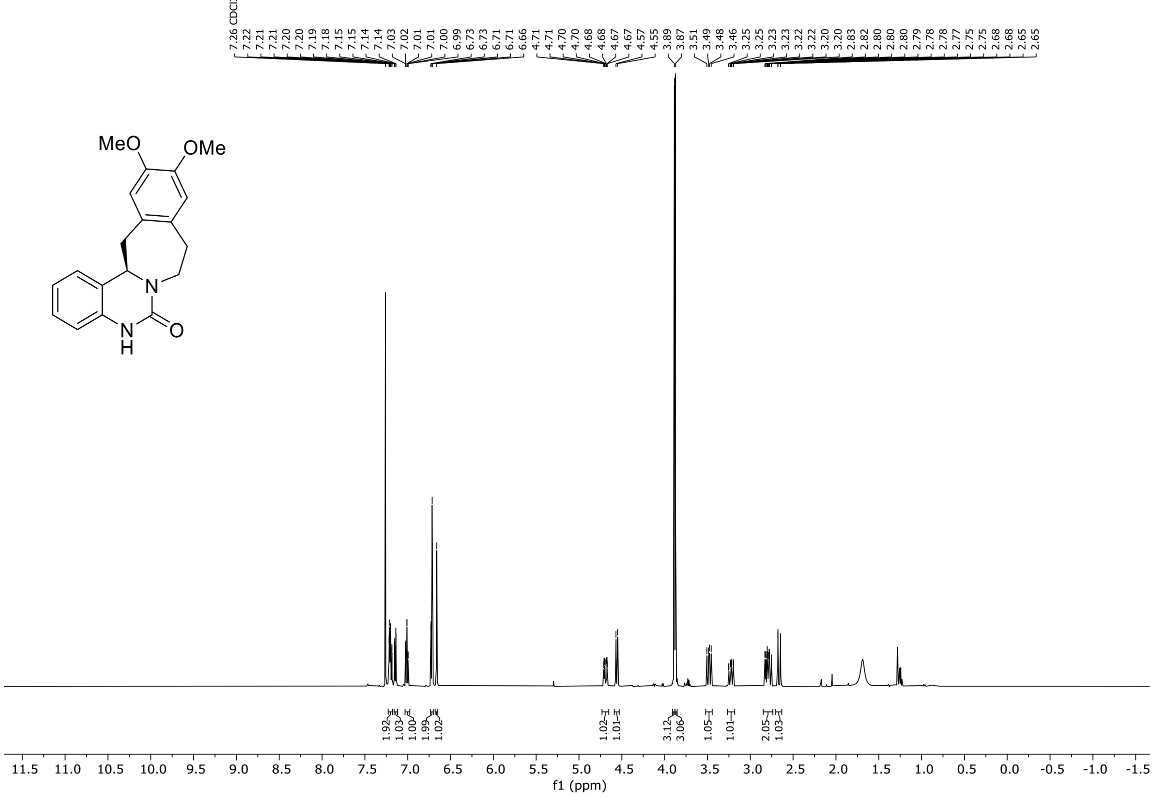

^{13}C NMR (126 MHz, $CDCl_3$):

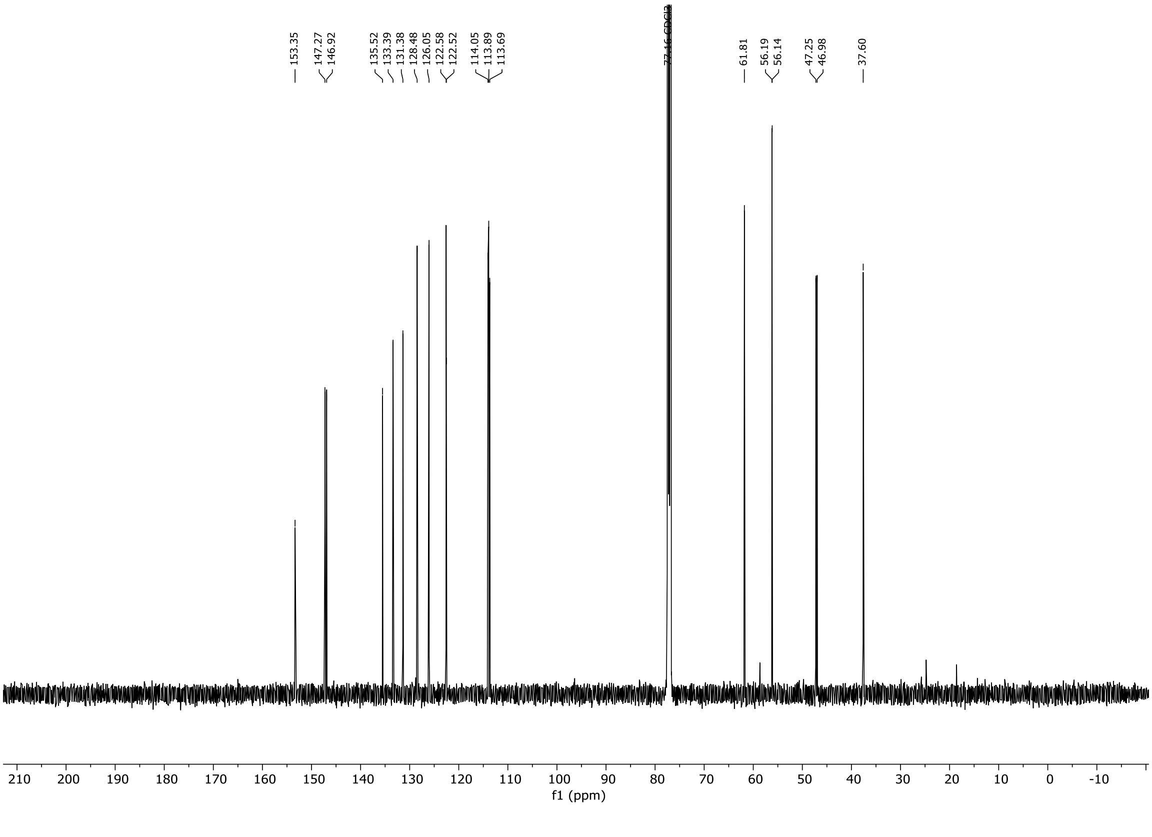

10.6. Deuteration Experiments

10.6.1. 3-(3,3-Diethoxypropyl)-3,4-dihydroquinazolin-2(1H)-one-4,4-d$_2$ ***(SI-8a-d***$_2$***)***

^{1}H NMR (500 MHz, CDCl$_3$):

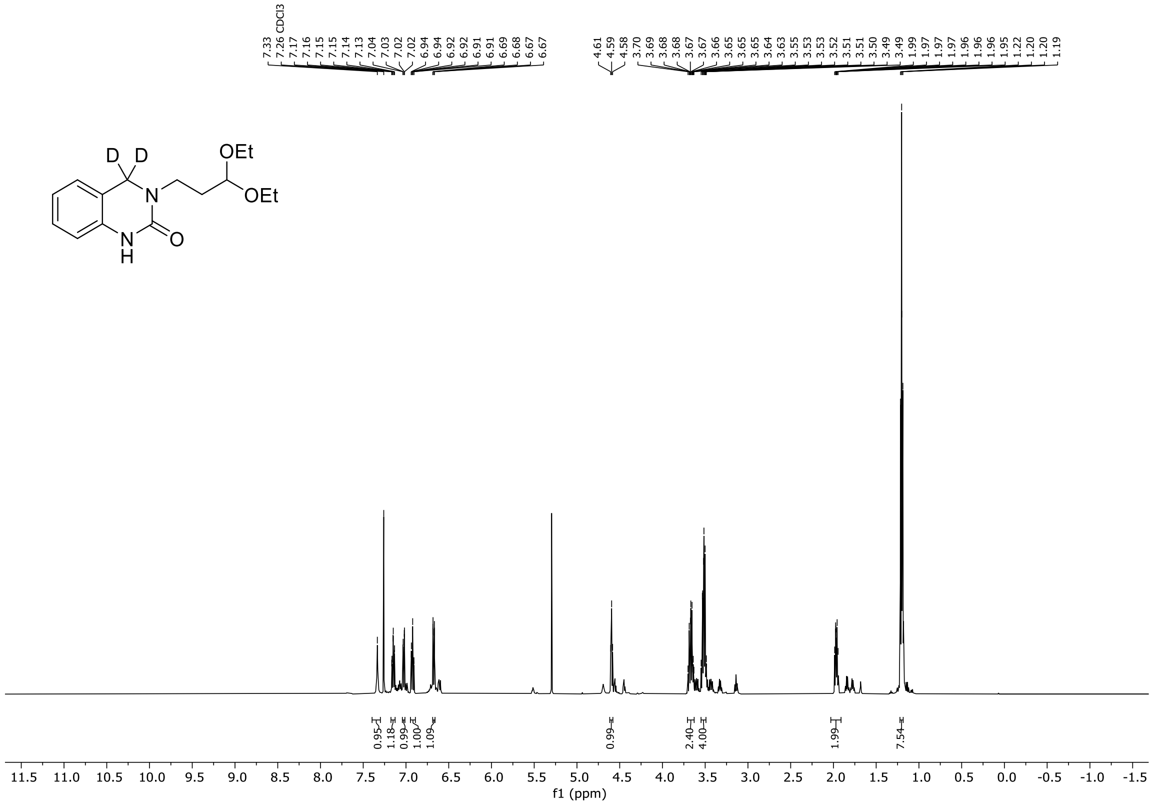

^{13}C NMR (126 MHz, CDCl$_3$):

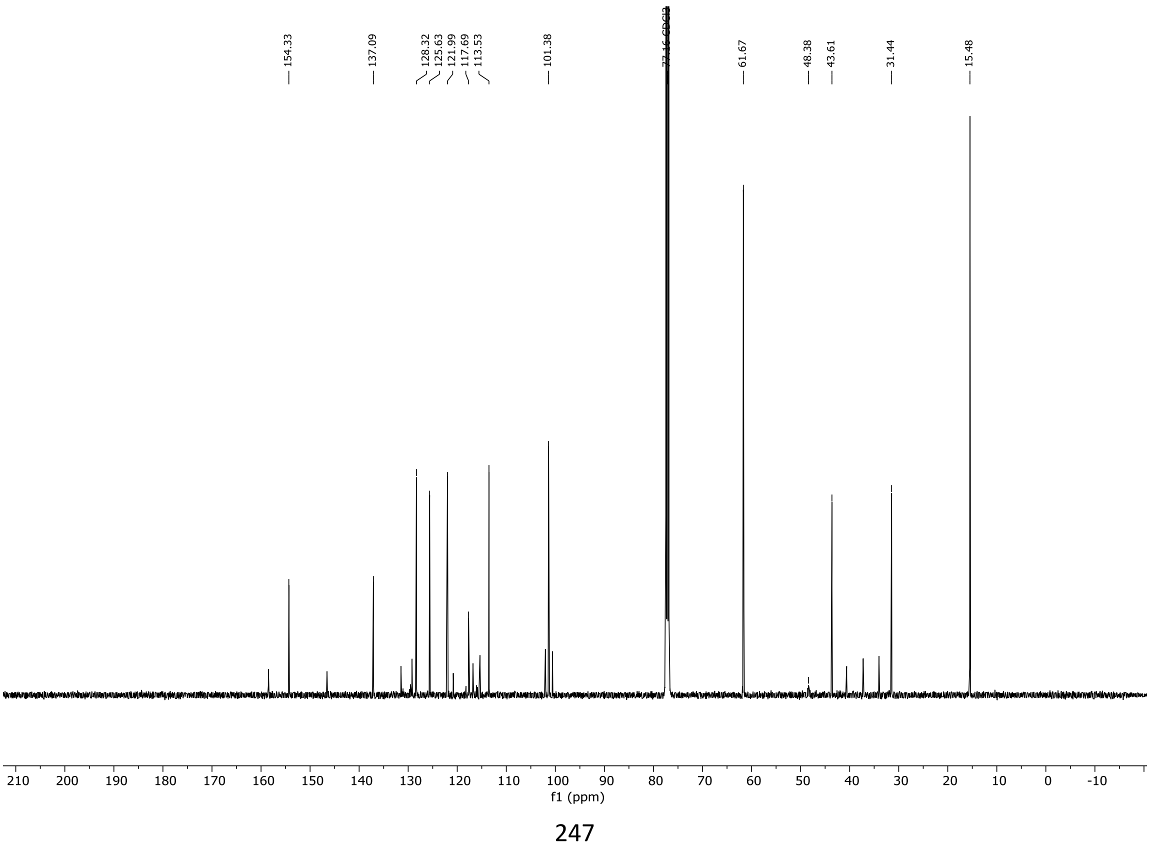

*10.6.2. N'-(3-(2-Oxo-1,4-dihydroquinazolin-3(2H)-yl-4,4-d_2)propylidene)-4-methylbenzenesulfonohydrazide (**4a**-d_2)*

^{1}H NMR (500 MHz, $CDCl_3$):

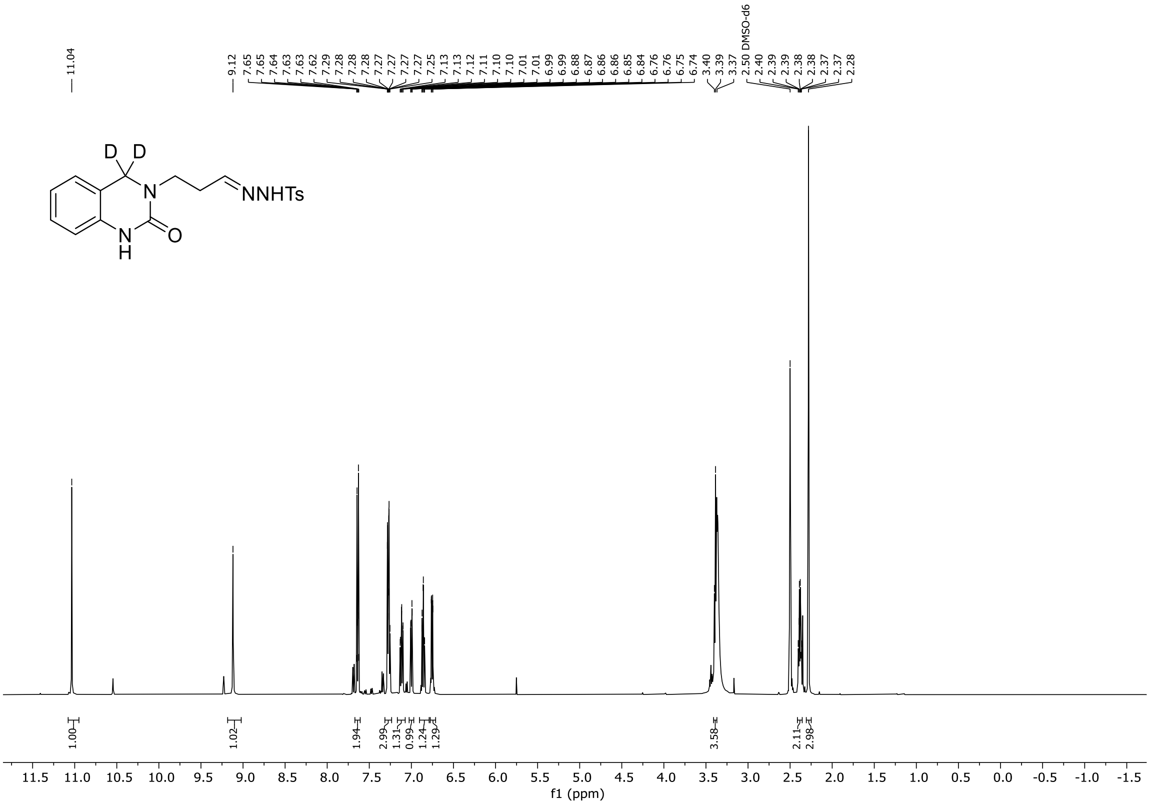

^{13}C NMR (126 MHz, $CDCl_3$):

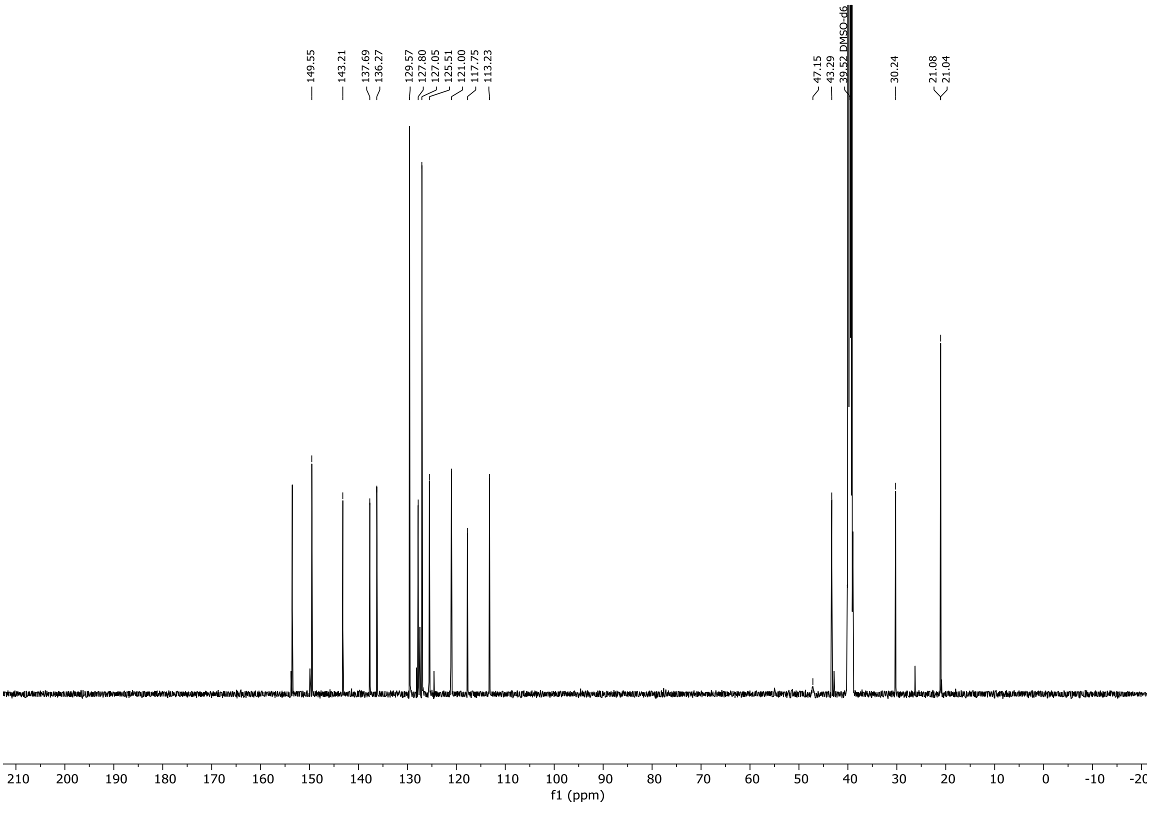

*10.6.3. 2,3,6,10b-Tetrahydropyrrolo[1,2-c]quinazolin-5(1H)-one-1,10b-d2 (rac-**5a**-d_2)*

^{1}H NMR (500 MHz, $CDCl_3$):

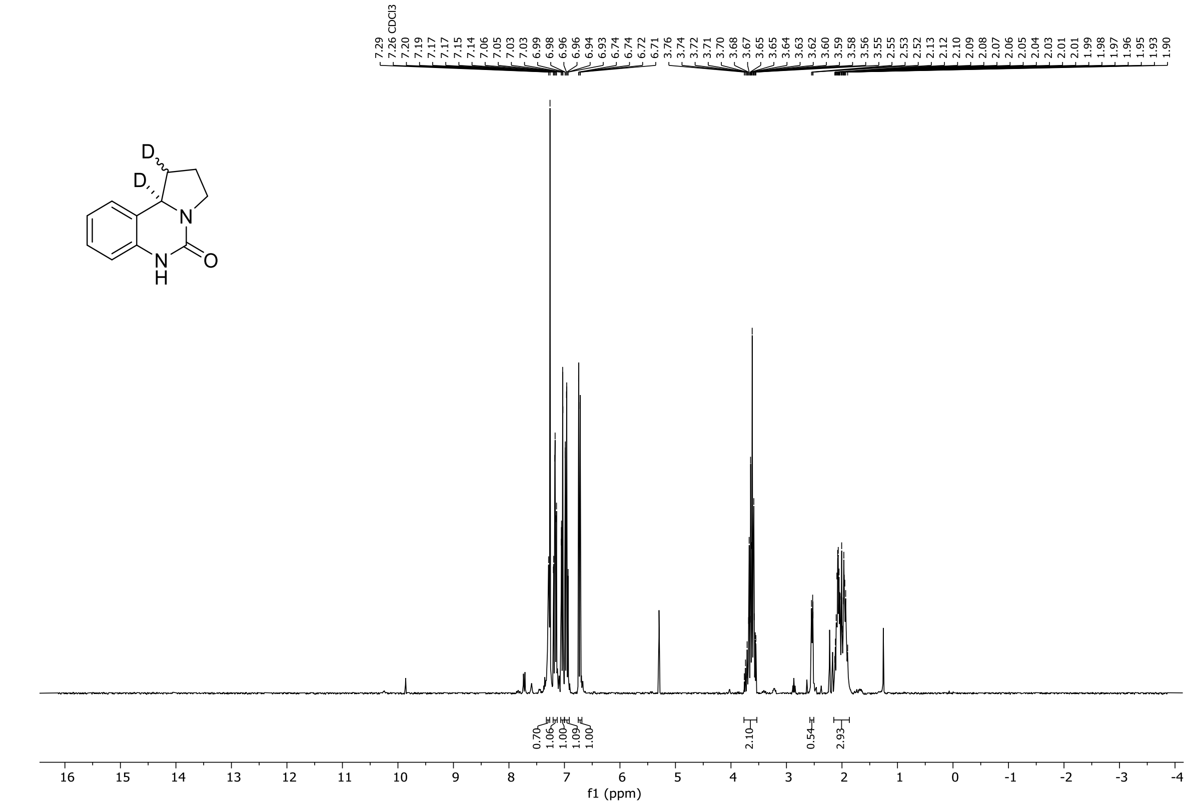

^{13}C NMR (126 MHz, $CDCl_3$):

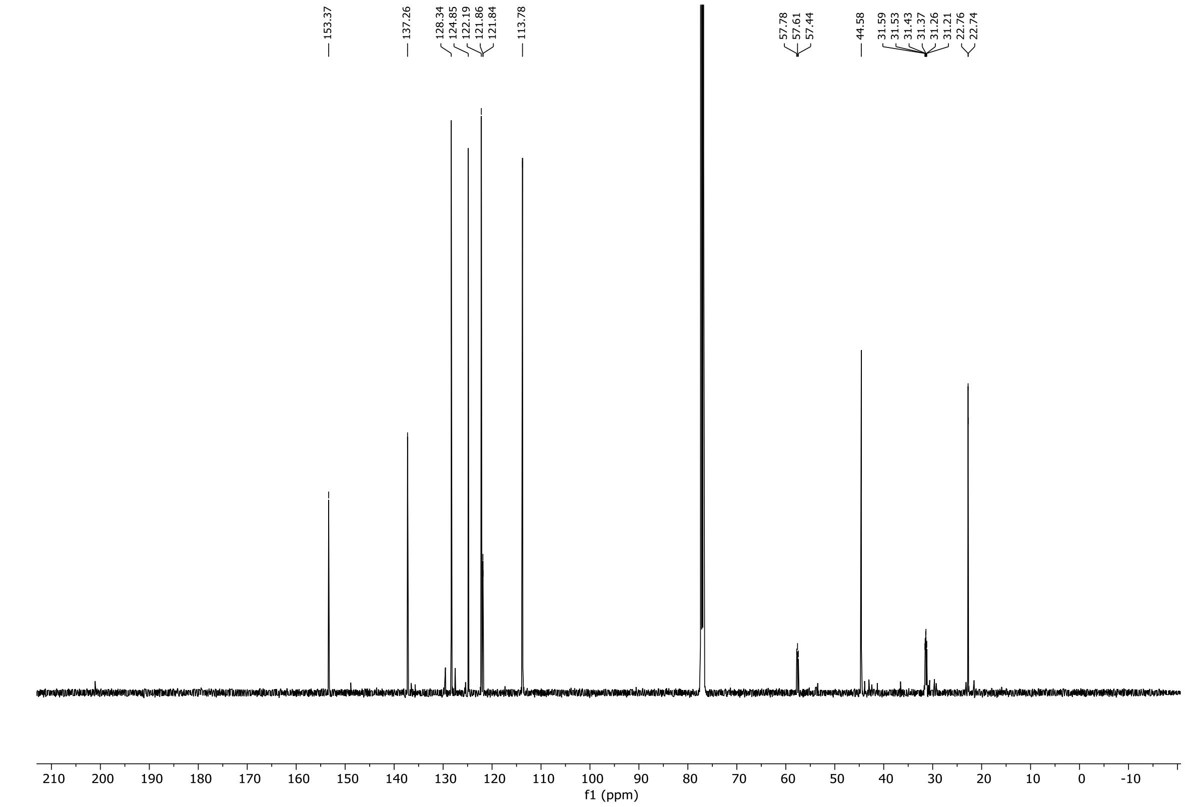

^{2}H NMR (500 MHz, $CHCl_3$):

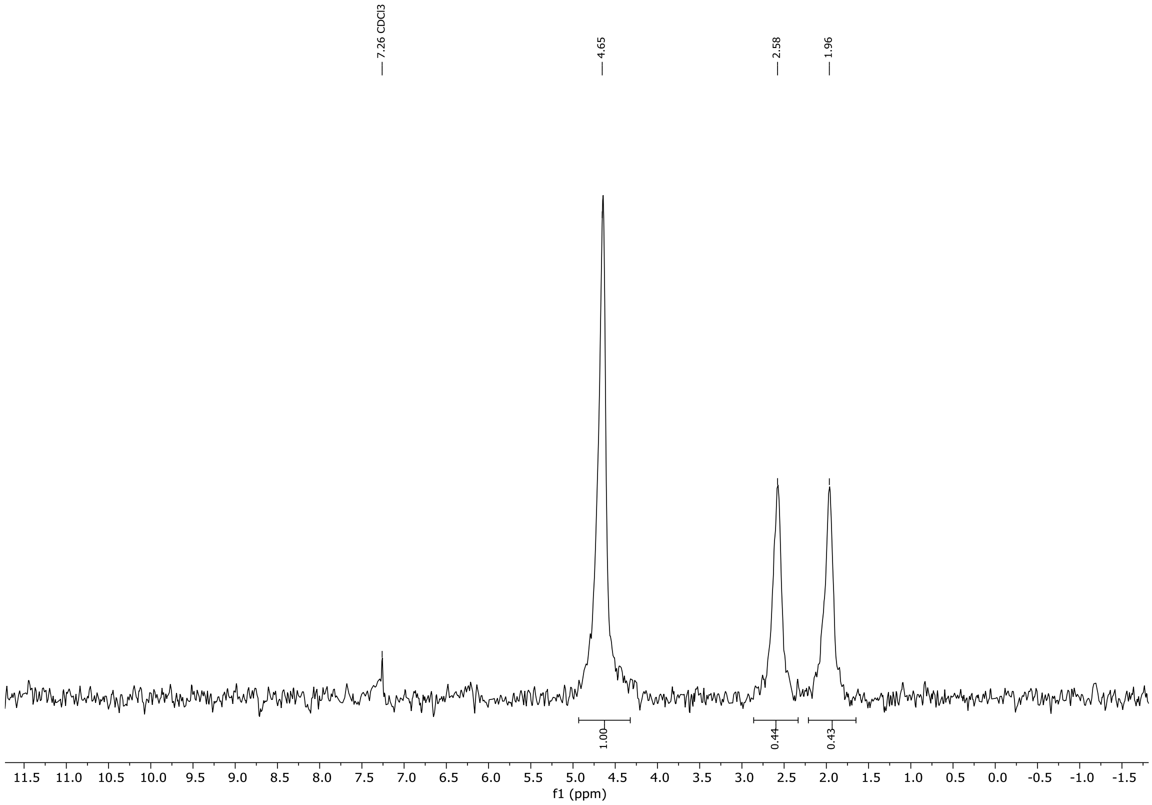

*10.6.4. 2,3,6,10b-Tetrahydropyrrolo[1,2-c]quinazolin-5(1H)-one-1,10b-d2 (**5a-**d_2)*

^{1}H NMR (500 MHz, $CDCl_3$):

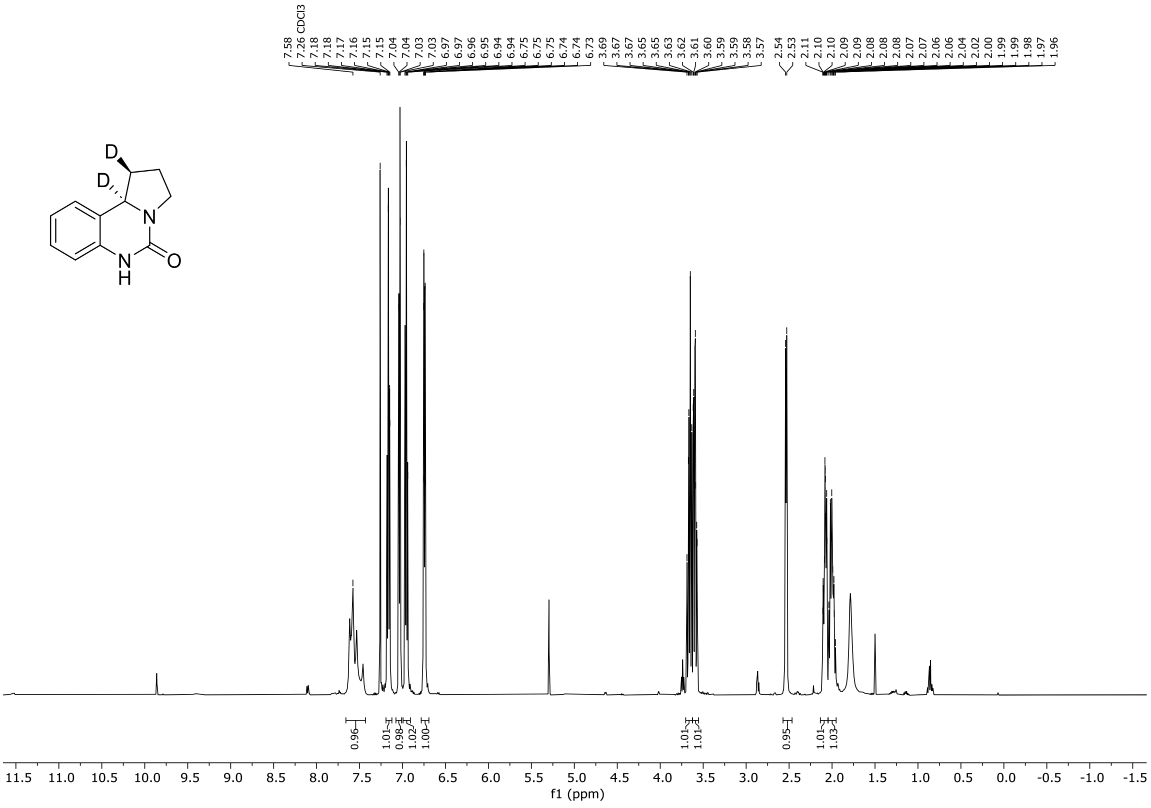

^{2}H NMR (500 MHz, $CHCl_3$):

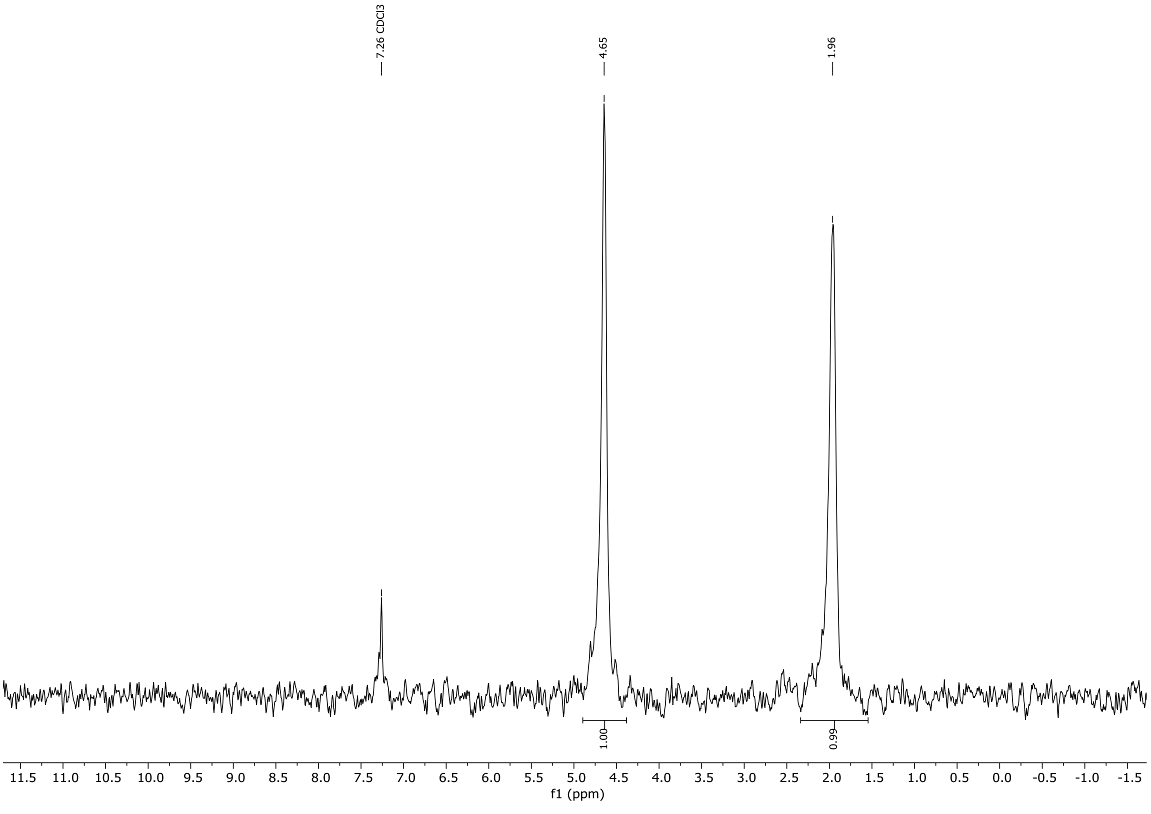

11. HPLC Traces

11.1. 2,3,6,10b-Tetrahydropyrrolo[1,2-c]quinazolin-5(1H)-one (5a)

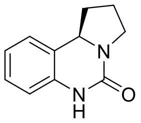

95% *ee*

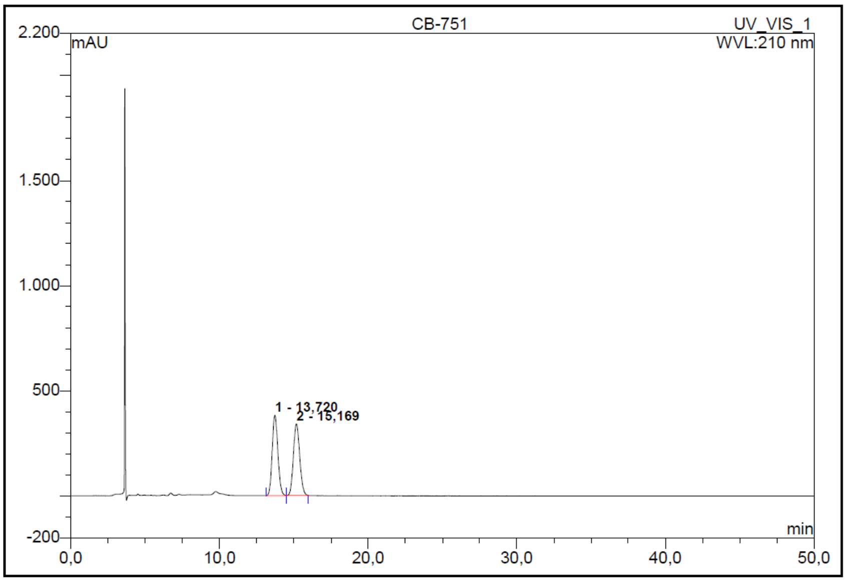

No.	Ret.Time min	Peak Name	Height mAU	Area mAU*min	Rel.Area %	Amount	Type
1	13,72	n.a.	381,947	169,273	50,12	n.a.	BM
2	15,17	n.a.	340,827	168,486	49,88	n.a.	MB
Total:			722,774	337,758	100,00	0,000	

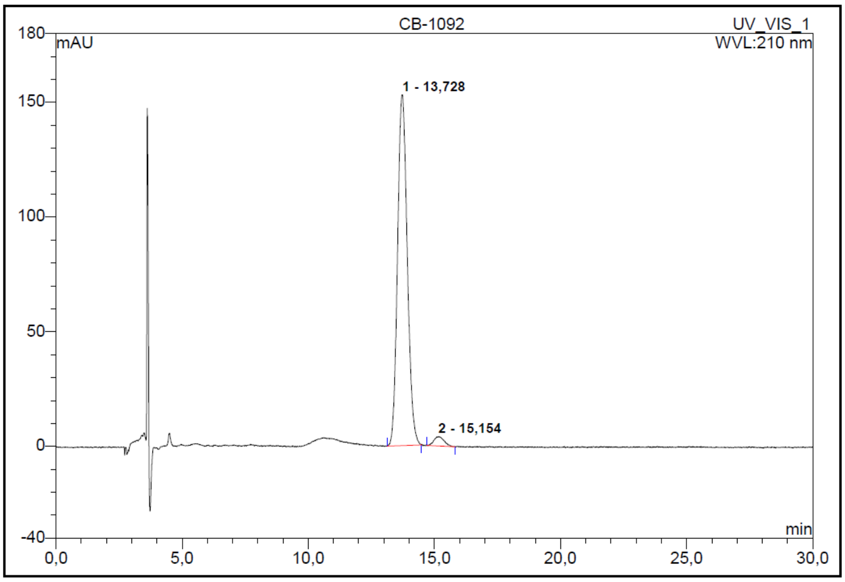

No.	Ret.Time min	Peak Name	Height mAU	Area mAU*min	Rel.Area %	Amount	Type
1	13,73	n.a.	153,067	68,364	97,24	n.a.	BMB
2	15,15	n.a.	4,156	1,938	2,76	n.a.	BMB*
Total:			157,223	70,302	100,00	0,000	

Scale-up Experiment:

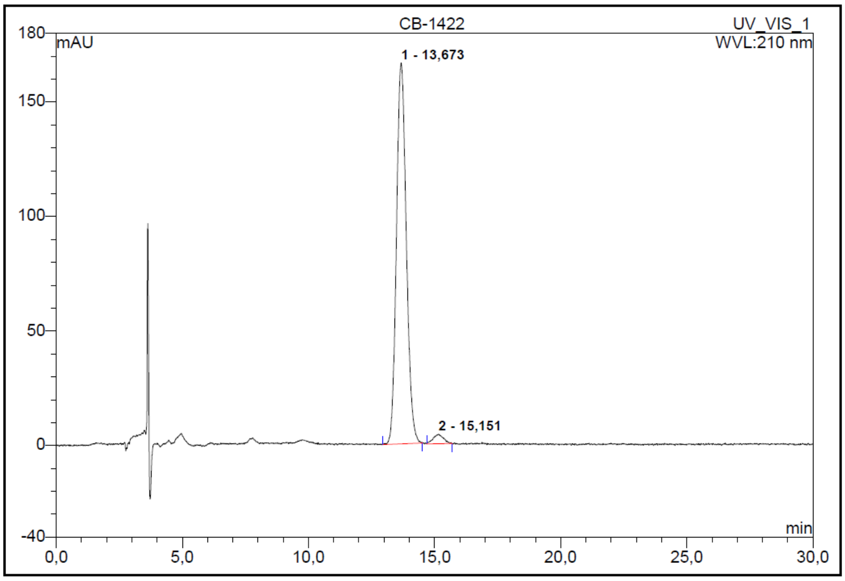

No.	Ret.Time min	Peak Name	Height mAU	Area mAU*min	Rel.Area %	Amount	Type
1	13,67	n.a.	166,399	75,211	97,49	n.a.	BMB*
2	15,15	n.a.	4,130	1,938	2,51	n.a.	BMB*
Total:			170,529	77,149	100,00	0,000	

One-pot Experiment:

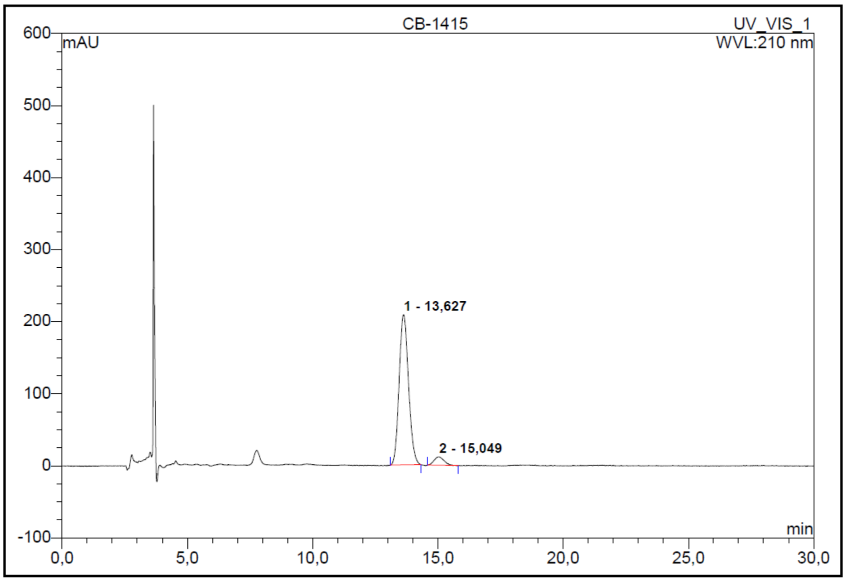

No.	Ret.Time min	Peak Name	Height mAU	Area mAU*min	Rel.Area %	Amount	Type
1	13,63	n.a.	208,610	90,773	94,54	n.a.	BMB
2	15,05	n.a.	11,628	5,240	5,46	n.a.	BMB*
Total:			220,238	96,013	100,00	0,000	

11.2. 10-Bromo-2,3,6,10b-tetrahydropyrrolo[1,2-c]quinazolin-5(1H)-one (5b)

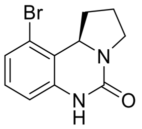

91% *ee*

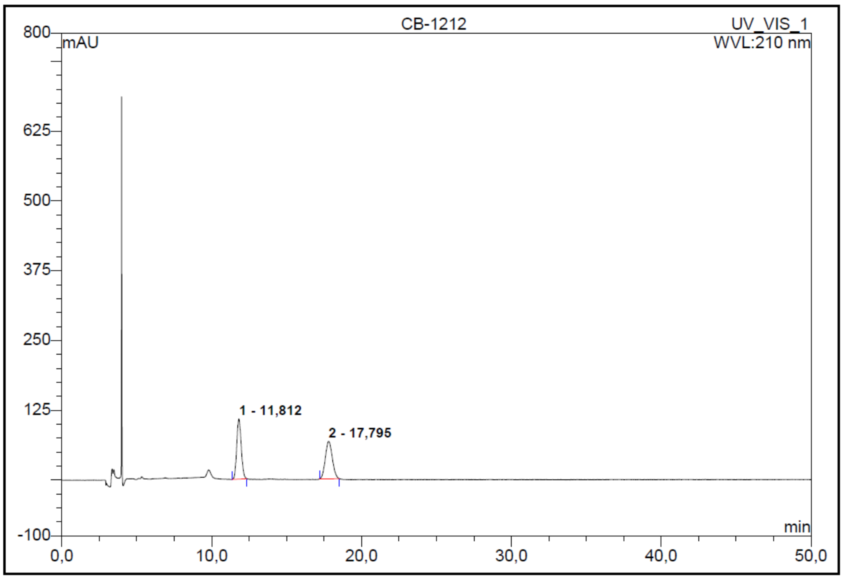

No.	Ret.Time min	Peak Name	Height mAU	Area mAU*min	Rel.Area %	Amount	Type
1	11,81	n.a.	107,798	37,279	50,69	n.a.	BMB
2	17,79	n.a.	67,175	36,264	49,31	n.a.	BMB
Total:			174,972	73,543	100,00	0,000	

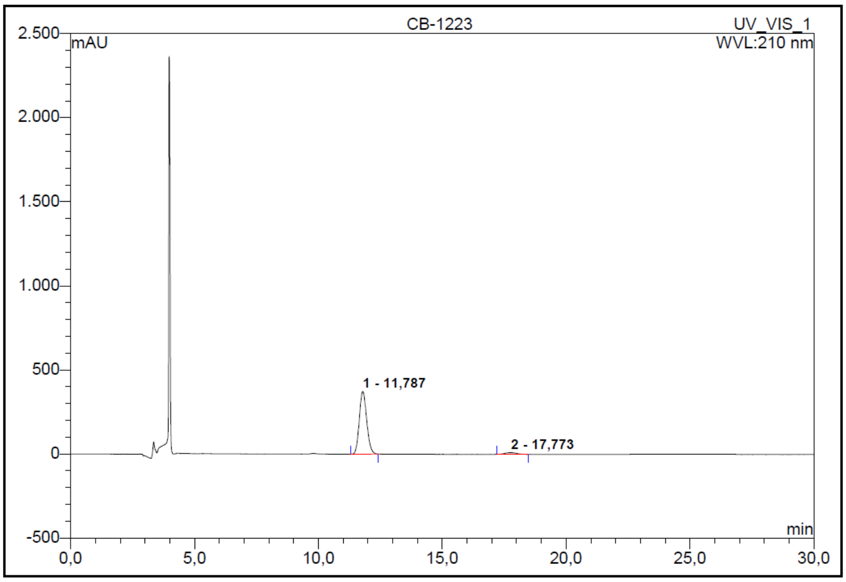

No.	Ret.Time min	Peak Name	Height mAU	Area mAU*min	Rel.Area %	Amount	Type
1	11,79	n.a.	373,687	130,206	95,53	n.a.	BMB
2	17,77	n.a.	11,708	6,092	4,47	n.a.	BMB*
Total:			385,395	136,298	100,00	0,000	

Preparative HPLC for crystallography:

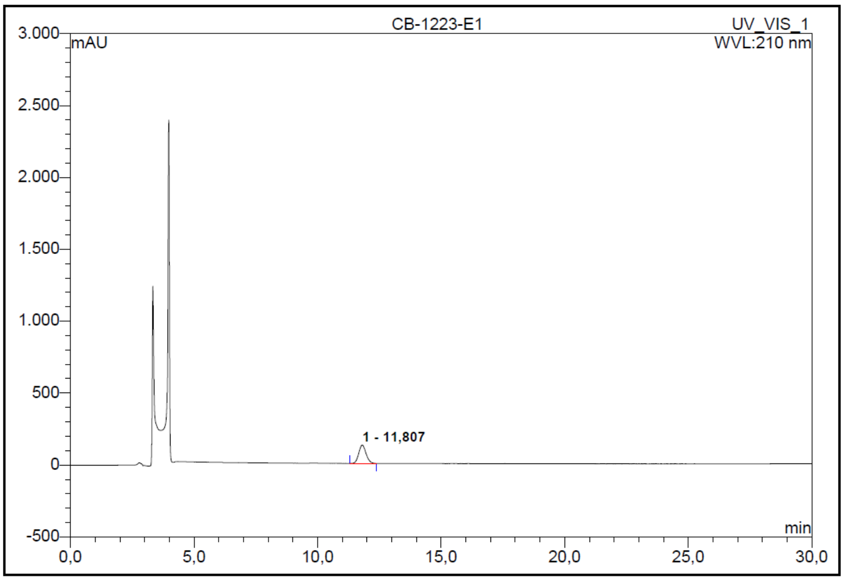

No.	Ret.Time min	Peak Name	Height mAU	Area mAU*min	Rel.Area %	Amount	Type
1	11,81	n.a.	127,387	43,873	100,00	n.a.	BMB
Total:			127,387	43,873	100,00	0,000	

11.3. 9-Bromo-2,3,6,10b-tetrahydropyrrolo[1,2-c]quinazolin-5(1H)-one (5c)

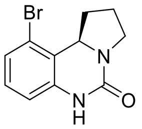

94% *ee*

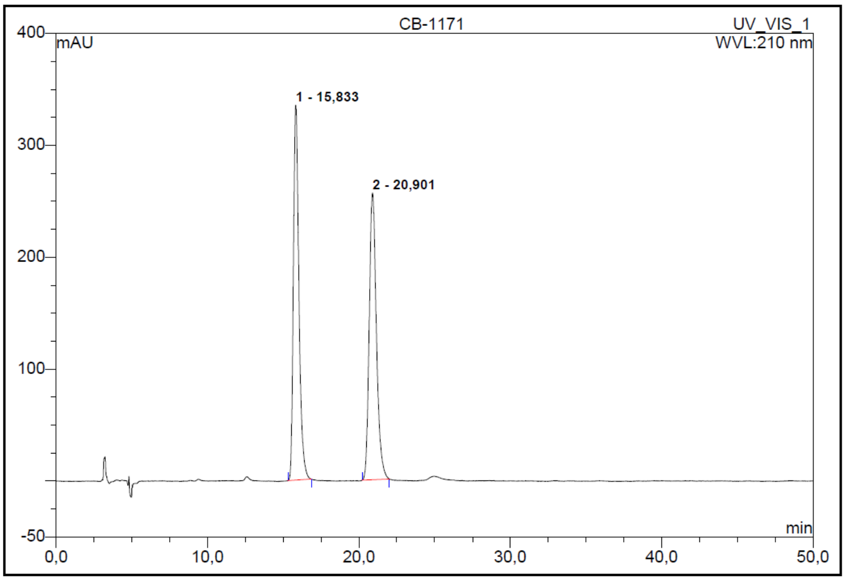

No.	Ret.Time min	Peak Name	Height mAU	Area mAU*min	Rel.Area %	Amount	Type
1	15,83	n.a.	335,306	141,220	50,04	n.a.	BMB
2	20,90	n.a.	256,381	141,006	49,96	n.a.	BMB
Total:			591,687	282,226	100,00	0,000	

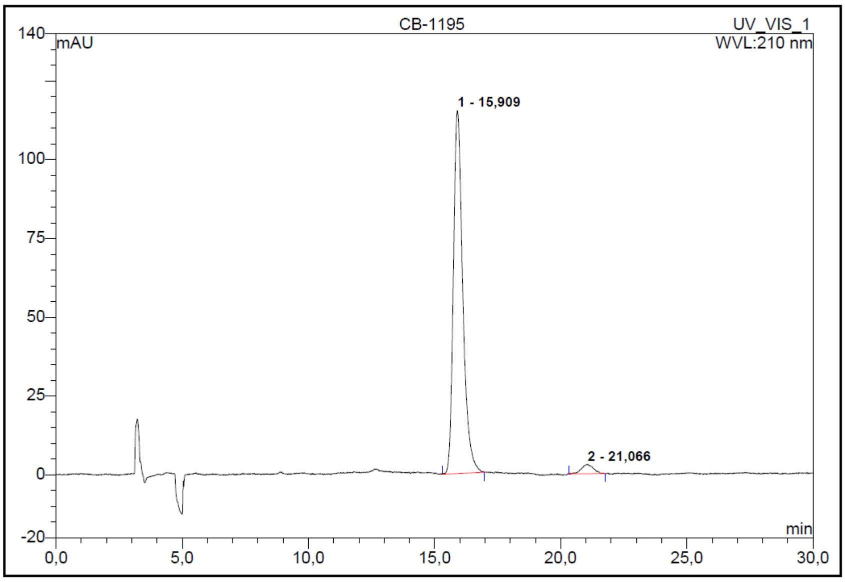

No.	Ret.Time min	Peak Name	Height mAU	Area mAU*min	Rel.Area %	Amount	Type
1	15,91	n.a.	115,194	49,672	96,91	n.a.	BMB*
2	21,07	n.a.	2,949	1,583	3,09	n.a.	BMB*
Total:			118,143	51,255	100,00	0,000	

11.4. 8-Bromo-2,3,6,10b-tetrahydropyrrolo[1,2-c]quinazolin-5(1H)-one (5d)

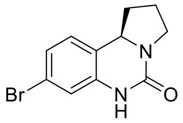

89% *ee*

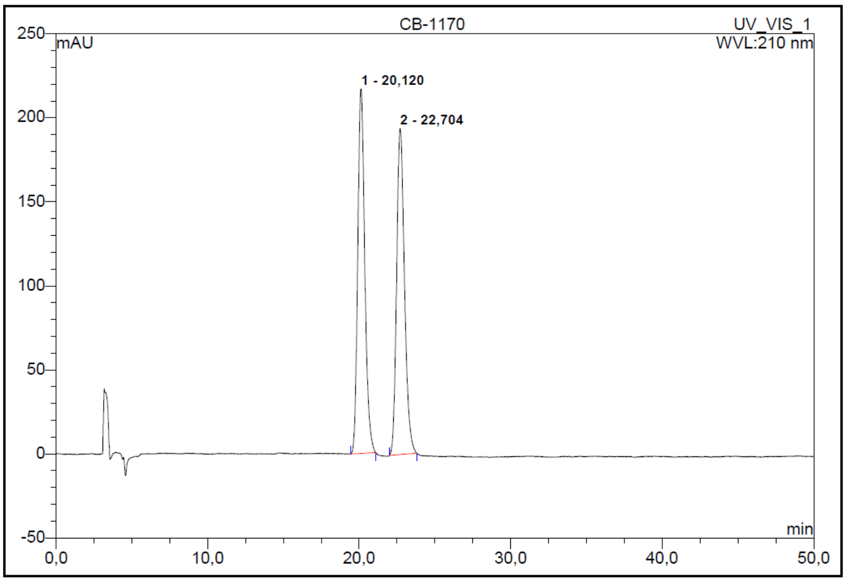

No.	Ret.Time min	Peak Name	Height mAU	Area mAU*min	Rel.Area %	Amount	Type
1	20,12	n.a.	216,960	116,671	50,01	n.a.	BMB
2	22,70	n.a.	193,958	116,629	49,99	n.a.	BMB
Total:			410,918	233,300	100,00	0,000	

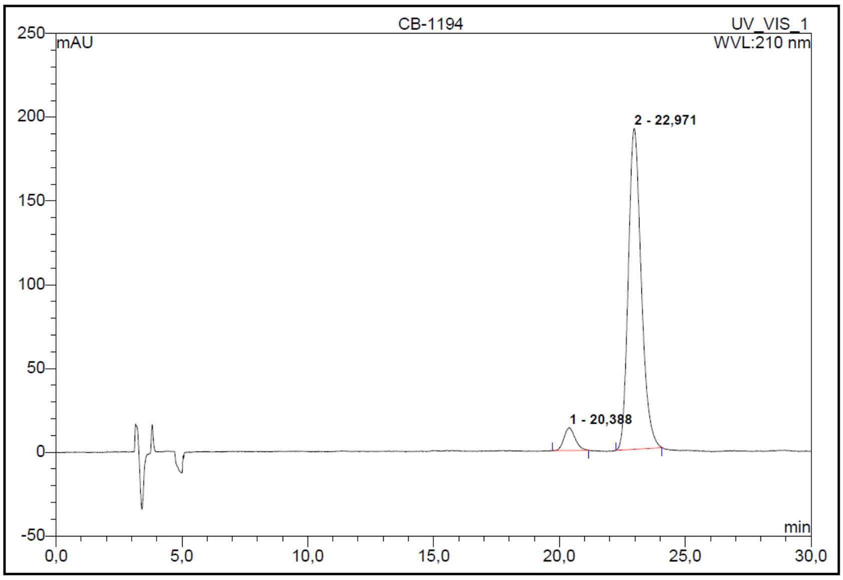

No.	Ret.Time min	Peak Name	Height mAU	Area mAU*min	Rel.Area %	Amount	Type
1	20,39	n.a.	13,609	6,832	5,65	n.a.	BMB*
2	22,97	n.a.	191,635	114,131	94,35	n.a.	BMB
Total:			205,244	120,963	100,00	0,000	

11.5. 7-Bromo-2,3,6,10b-tetrahydropyrrolo[1,2-c]quinazolin-5(1H)-one (5e)

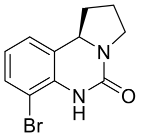

47% *ee*

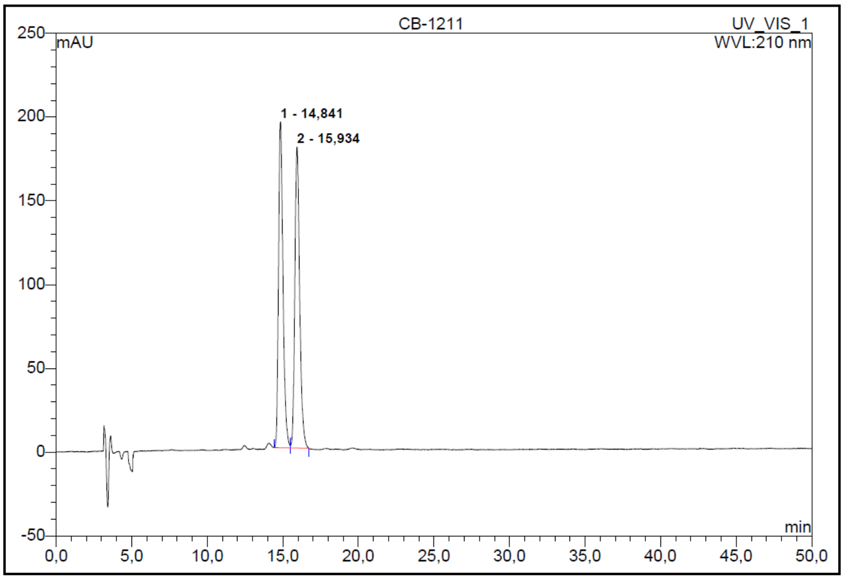

No.	Ret.Time min	Peak Name	Height mAU	Area mAU*min	Rel.Area %	Amount	Type
1	14,84	n.a.	194,571	66,703	49,93	n.a.	BM
2	15,93	n.a.	179,732	66,892	50,07	n.a.	MB
Total:			374,303	133,595	100,00	0,000	

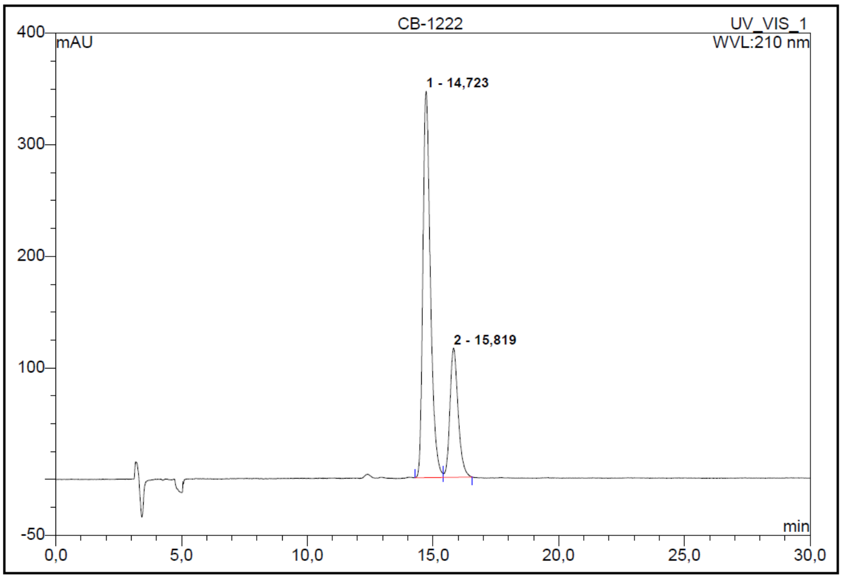

No.	Ret.Time min	Peak Name	Height mAU	Area mAU*min	Rel.Area %	Amount	Type
1	14,72	n.a.	346,171	120,381	73,38	n.a.	BM
2	15,82	n.a.	115,720	43,670	26,62	n.a.	MB
Total:			461,891	164,051	100,00	0,000	

11.6. 9-Chloro-2,3,6,10b-tetrahydropyrrolo[1,2-c]quinazolin-5(1H)-one (5f)

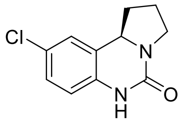

95% *ee*

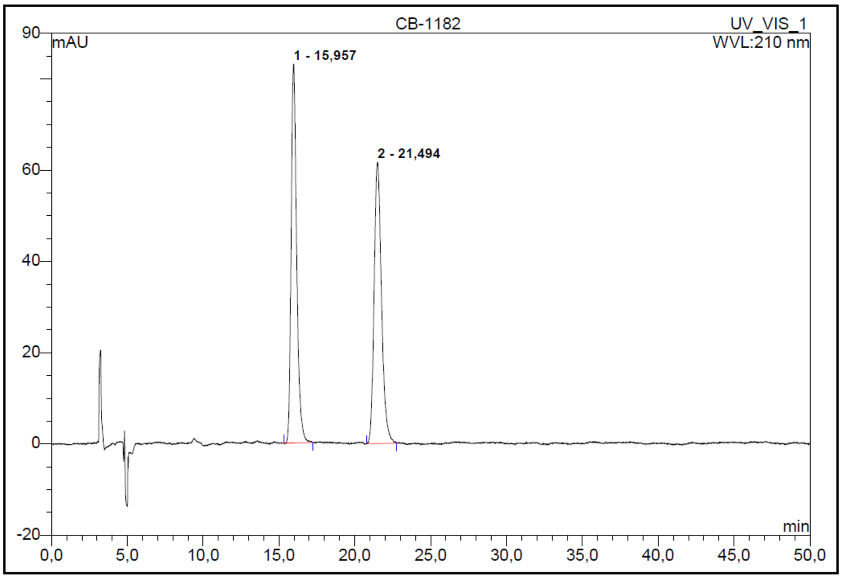

No.	Ret.Time min	Peak Name	Height mAU	Area mAU*min	Rel.Area %	Amount	Type
1	15,96	n.a.	83,028	34,924	50,13	n.a.	BMB*
2	21,49	n.a.	61,702	34,745	49,87	n.a.	BMB*
Total:			144,730	69,669	100,00	0,000	

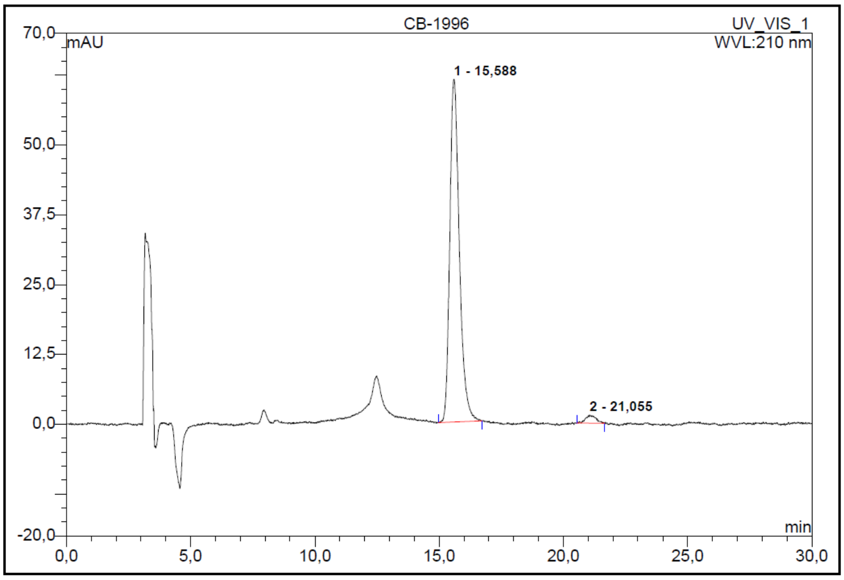

No.	Ret.Time min	Peak Name	Height mAU	Area mAU*min	Rel.Area %	Amount	Type
1	15,59	n.a.	61,388	27,344	97,46	n.a.	BMB*
2	21,06	n.a.	1,492	0,713	2,54	n.a.	BMB*
Total:			62,880	28,057	100,00	0,000	

11.7. 8-Fluoro-2,3,6,10b-tetrahydropyrrolo[1,2-c]quinazolin-5(1H)-one (5g)

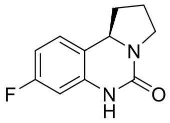

99% *ee*

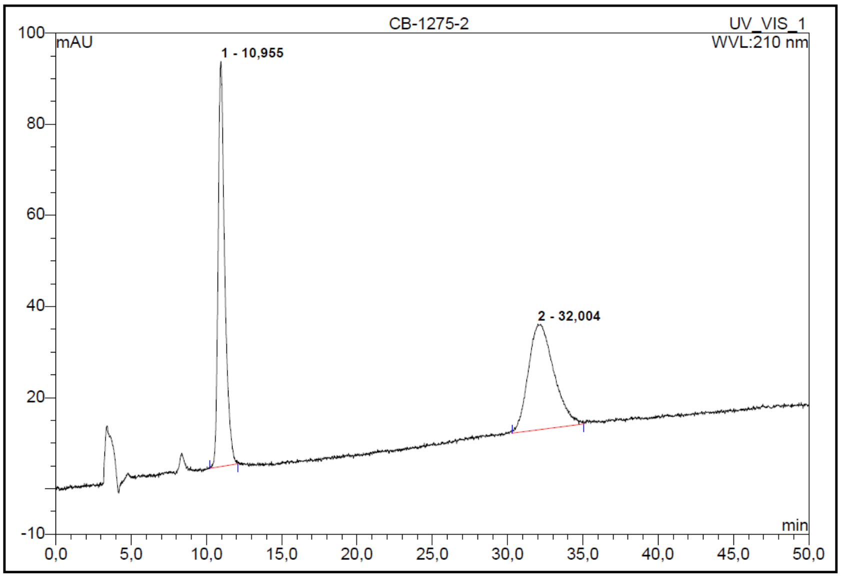

No.	Ret.Time min	Peak Name	Height mAU	Area mAU*min	Rel.Area %	Amount	Type
1	10,95	n.a.	88,944	45,517	49,97	n.a.	BMB*
2	32,00	n.a.	23,240	45,575	50,03	n.a.	BMB*
Total:			112,185	91,092	100,00	0,000	

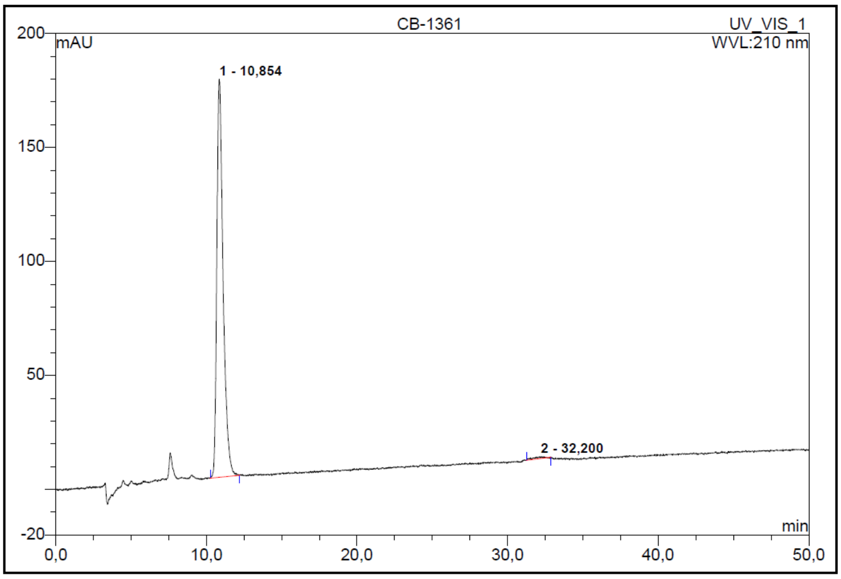

No.	Ret.Time min	Peak Name	Height mAU	Area mAU*min	Rel.Area %	Amount	Type
1	10,85	n.a.	174,705	84,500	99,26	n.a.	BMB*
2	32,20	n.a.	0,868	0,626	0,74	n.a.	BMB*
Total:			175,573	85,126	100,00	0,000	

11.8. 8-(Trifluoromethyl)-2,3,6,10b-tetrahydropyrrolo[1,2-c]quinazolin-5(1H)-one (5h)

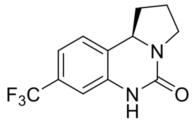

95% *ee*

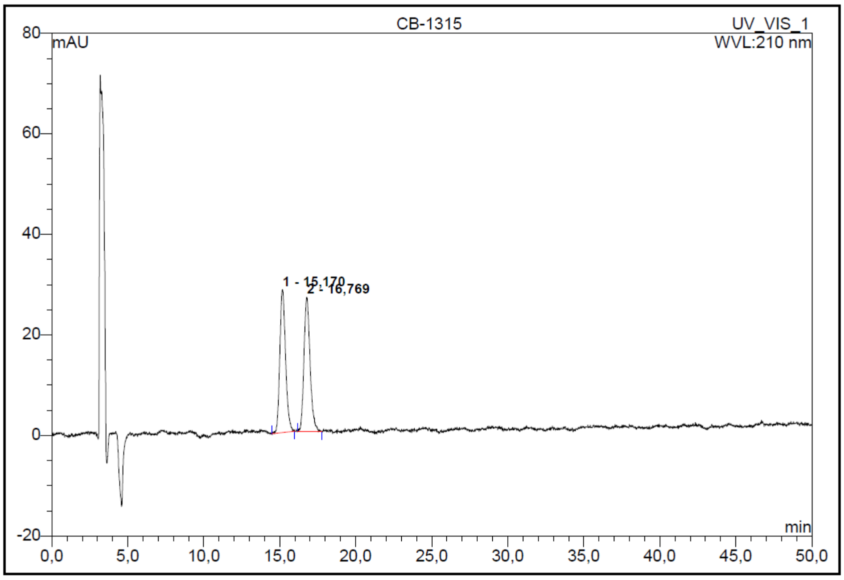

No.	Ret.Time min	Peak Name	Height mAU	Area mAU*min	Rel.Area %	Amount	Type
1	15,17	n.a.	28,432	12,478	50,21	n.a.	BMB*
2	16,77	n.a.	26,659	12,374	49,79	n.a.	BMB*
Total:			55,091	24,852	100,00	0,000	

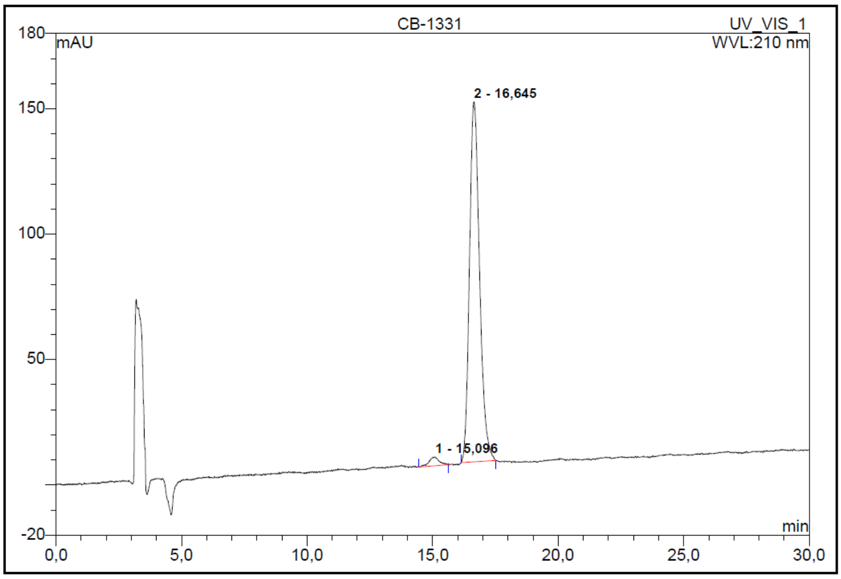

No.	Ret.Time min	Peak Name	Height mAU	Area mAU*min	Rel.Area %	Amount	Type
1	15,10	n.a.	3,549	1,666	2,43	n.a.	BMB*
2	16,65	n.a.	143,644	66,788	97,57	n.a.	BMB
Total:			147,193	68,454	100,00	0,000	

11.9. 9-Vinyl-2,3,6,10b-tetrahydropyrrolo[1,2-c]quinazolin-5(1H)-one (5i)

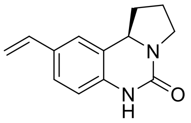

96% *ee*

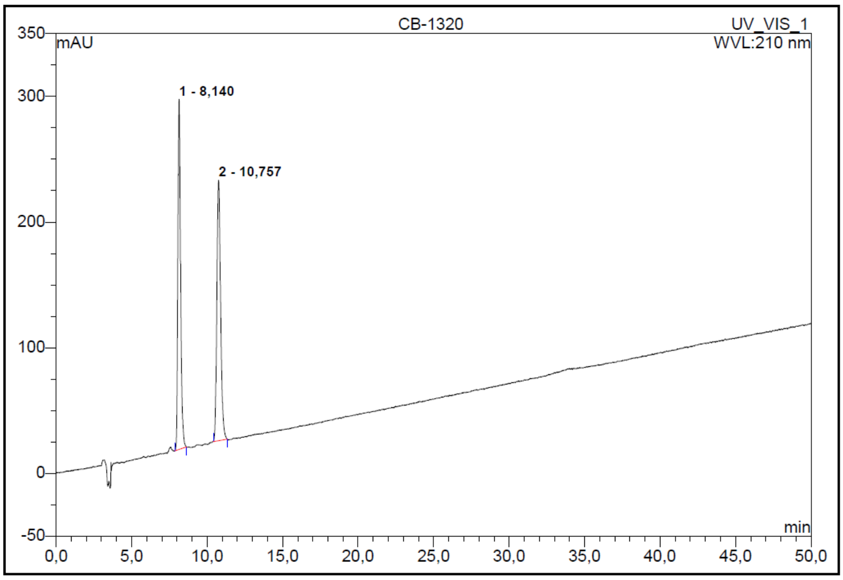

No.	Ret.Time min	Peak Name	Height mAU	Area mAU*min	Rel.Area %	Amount	Type
1	8,14	n.a.	278,640	59,055	49,82	n.a.	BMB
2	10,76	n.a.	207,282	59,479	50,18	n.a.	BMB
Total:			485,922	118,533	100,00	0,000	

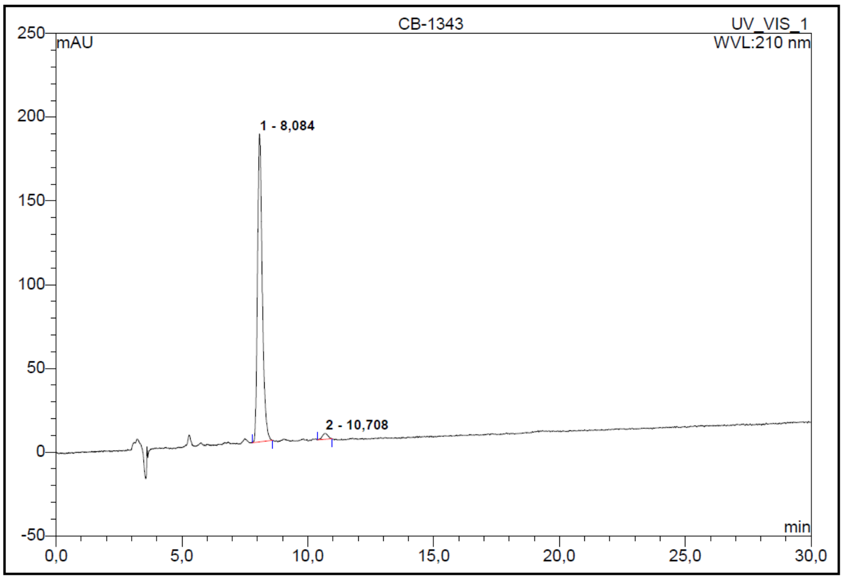

No.	Ret.Time min	Peak Name	Height mAU	Area mAU*min	Rel.Area %	Amount	Type
1	8,08	n.a.	183,976	40,230	97,84	n.a.	BMB
2	10,71	n.a.	3,440	0,888	2,16	n.a.	BMB*
Total:			187,416	41,118	100,00	0,000	

11.10. 9-Allyl-2,3,6,10b-tetrahydropyrrolo[1,2-c]quinazolin-5(1H)-one (5j)

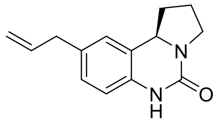

93% *ee*

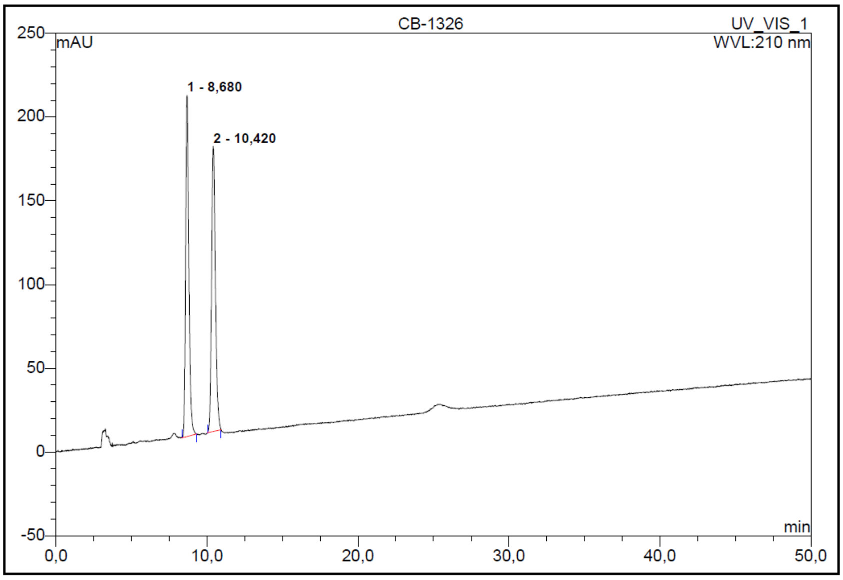

No.	Ret.Time min	Peak Name	Height mAU	Area mAU*min	Rel.Area %	Amount	Type
1	8,68	n.a.	203,765	50,744	50,29	n.a.	BMB
2	10,42	n.a.	170,255	50,154	49,71	n.a.	BMB
Total:			374,019	100,899	100,00	0,000	

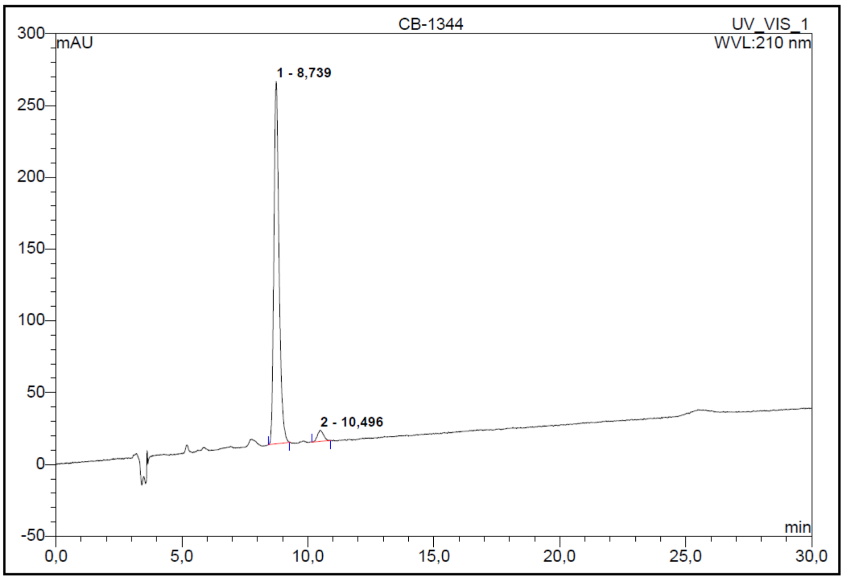

No.	Ret.Time min	Peak Name	Height mAU	Area mAU*min	Rel.Area %	Amount	Type
1	8,74	n.a.	252,398	59,902	96,51	n.a.	BMB*
2	10,50	n.a.	7,708	2,164	3,49	n.a.	BMB*
Total:			260,106	62,066	100,00	0,000	

11.11. 7-Methyl-2,3,6,10b-tetrahydropyrrolo[1,2-c]quinazolin-5(1H)-one (5k)

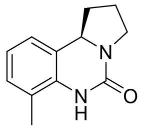

92% *ee*

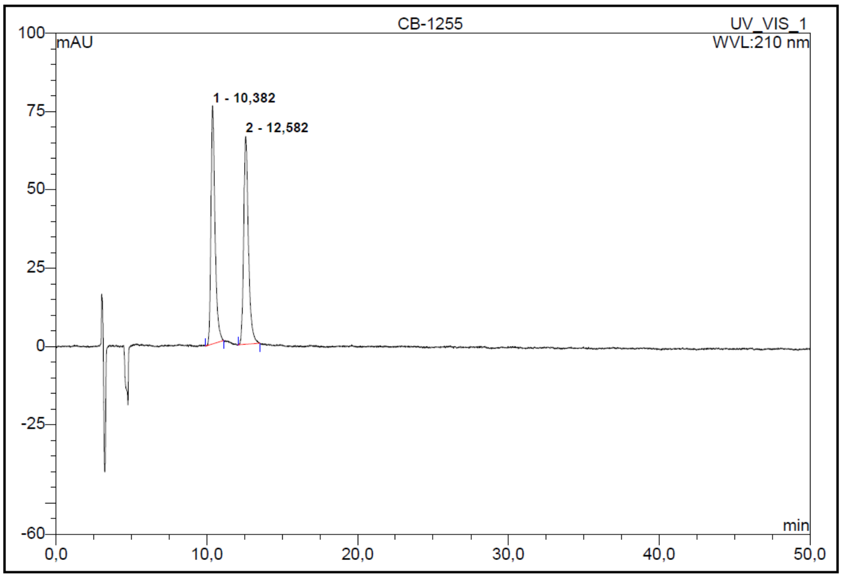

No.	Ret.Time min	Peak Name	Height mAU	Area mAU*min	Rel.Area %	Amount	Type
1	10,38	n.a.	76,087	23,851	49,63	n.a.	BMB*
2	12,58	n.a.	66,412	24,202	50,37	n.a.	BMB*
Total:			142,498	48,052	100,00	0,000	

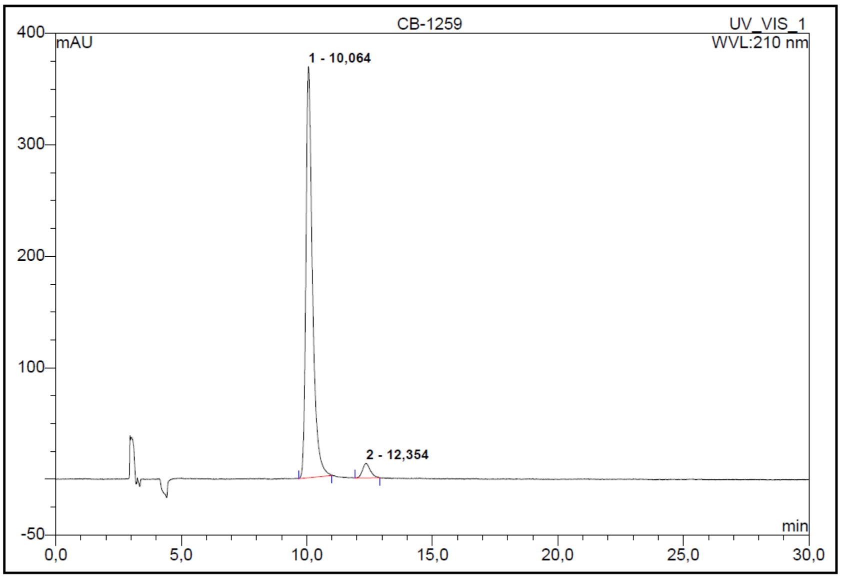

No.	Ret.Time min	Peak Name	Height mAU	Area mAU*min	Rel.Area %	Amount	Type
1	10,06	n.a.	368,947	111,759	96,05	n.a.	BMB
2	12,35	n.a.	12,911	4,595	3,95	n.a.	BMB*
Total:			381,858	116,355	100,00	0,000	

11.12. 9-Methoxy-2,3,6,10b-tetrahydropyrrolo[1,2-c]quinazolin-5(1H)-one (5l)

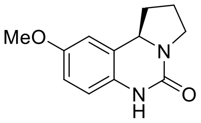

94% *ee*

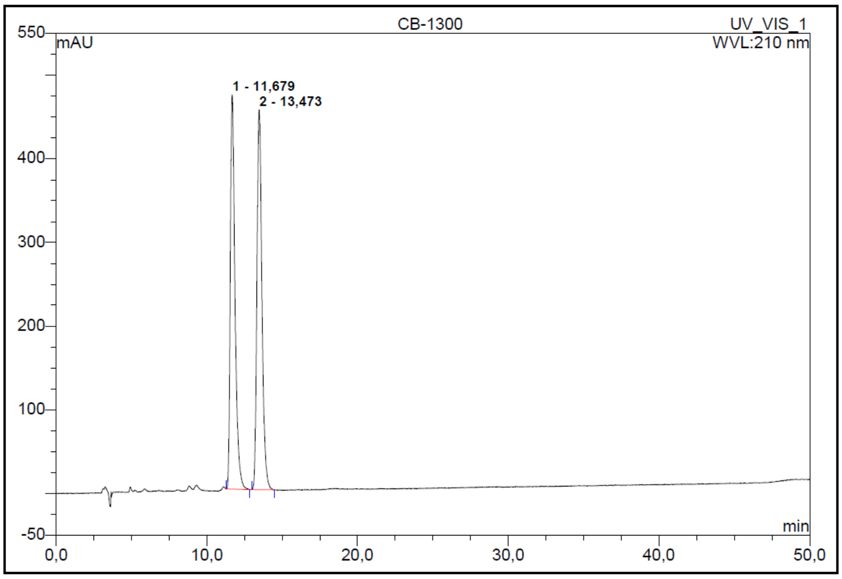

No.	Ret.Time min	Peak Name	Height mAU	Area mAU*min	Rel.Area %	Amount	Type
1	11,68	n.a.	471,794	171,460	49,55	n.a.	BMB*
2	13,47	n.a.	454,430	174,580	50,45	n.a.	BMB*
Total:			926,223	346,041	100,00	0,000	

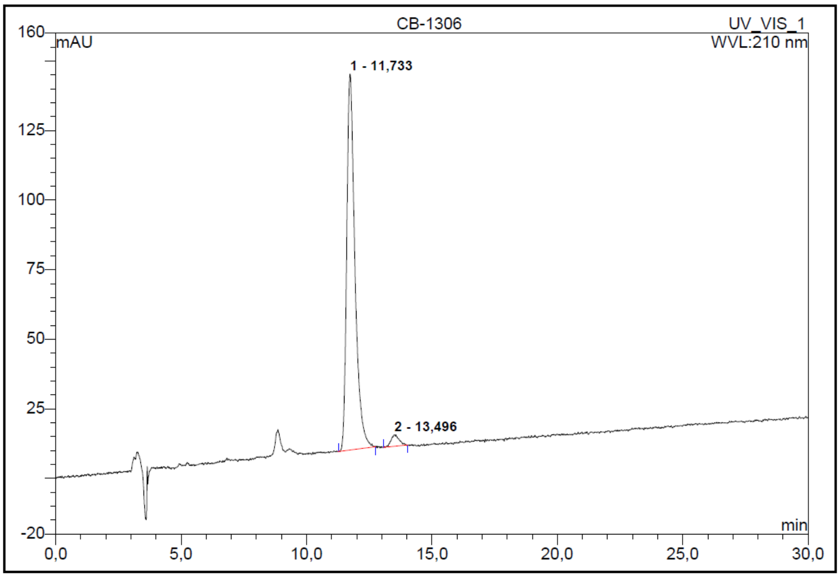

No.	Ret.Time min	Peak Name	Height mAU	Area mAU*min	Rel.Area %	Amount	Type
1	11,73	n.a.	135,147	51,116	97,14	n.a.	BMB*
2	13,50	n.a.	4,156	1,506	2,86	n.a.	BMB*
Total:			139,302	52,622	100,00	0,000	

11.13. 7-Methoxy-2,3,6,10b-tetrahydropyrrolo[1,2-c]quinazolin-5(1H)-one (5m)

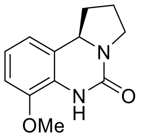

58% *ee*

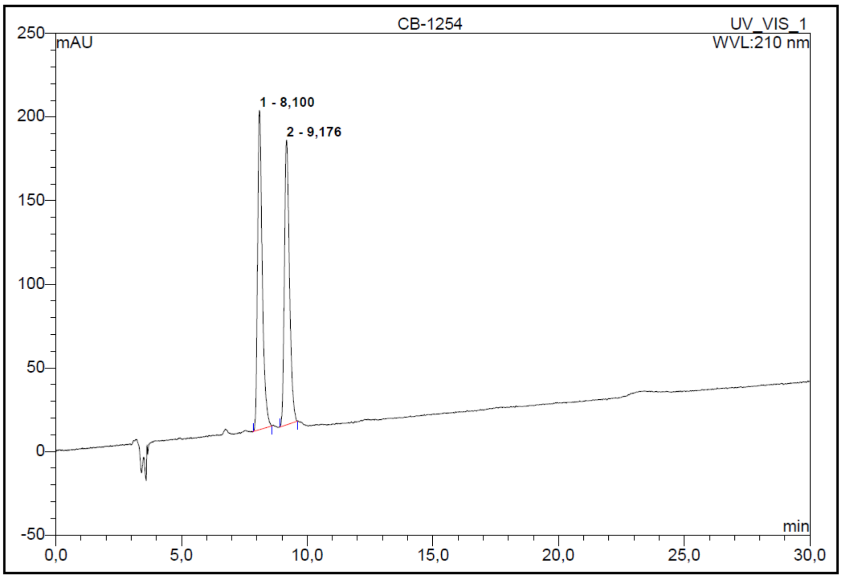

No.	Ret.Time min	Peak Name	Height mAU	Area mAU*min	Rel.Area %	Amount	Type
1	8,10	n.a.	190,988	41,135	50,81	n.a.	BMB
2	9,18	n.a.	170,255	39,819	49,19	n.a.	BMB
Total:			361,243	80,954	100,00	0,000	

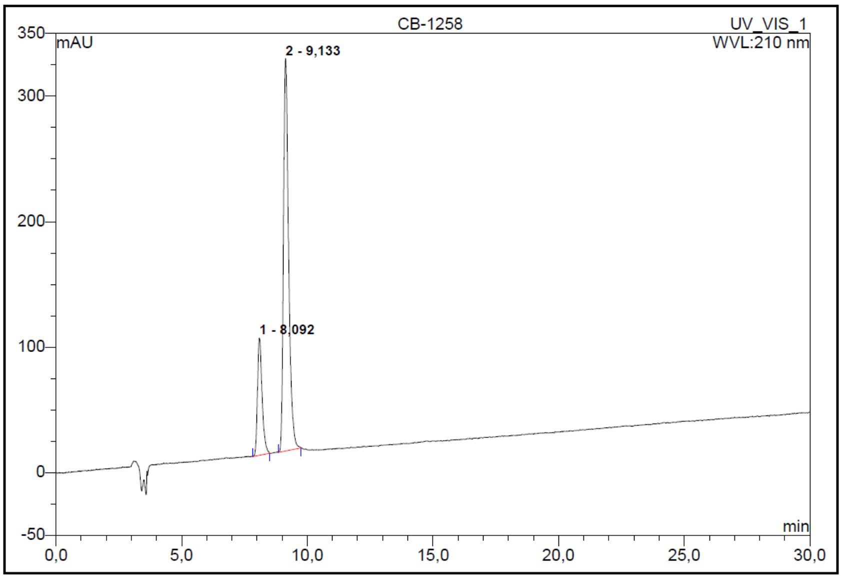

No.	Ret.Time min	Peak Name	Height mAU	Area mAU*min	Rel.Area %	Amount	Type
1	8,09	n.a.	93,430	19,811	21,00	n.a.	BMB
2	9,13	n.a.	312,687	74,535	79,00	n.a.	BMB
Total:			406,118	94,347	100,00	0,000	

11.14. 8-(Furan-3-yl)-2,3,6,10b-tetrahydropyrrolo[1,2-c]quinazolin-5(1H)-one (5n)

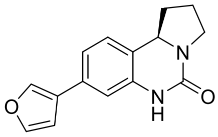

98% *ee*

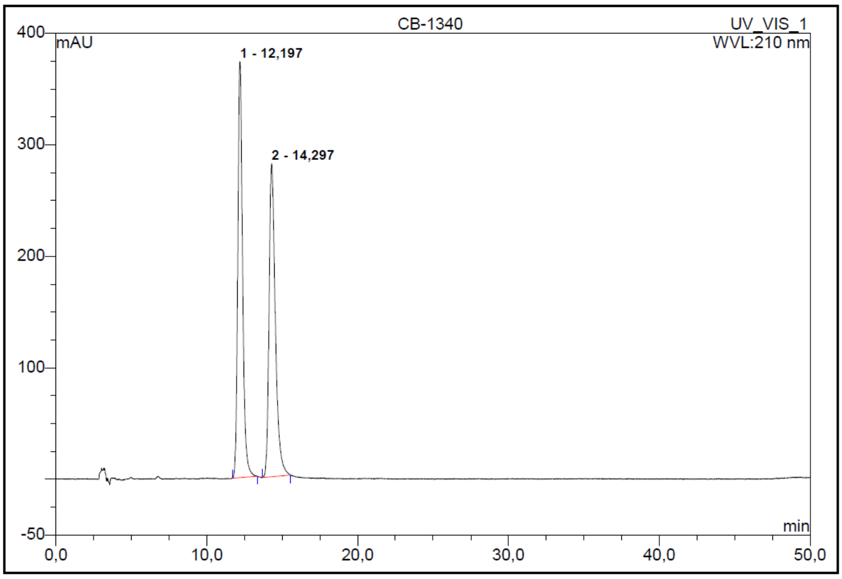

No.	Ret.Time min	Peak Name	Height mAU	Area mAU*min	Rel.Area %	Amount	Type
1	12,20	n.a.	373,472	137,012	50,40	n.a.	BMB*
2	14,30	n.a.	280,858	134,863	49,60	n.a.	BMB
Total:			654,330	271,874	100,00	0,000	

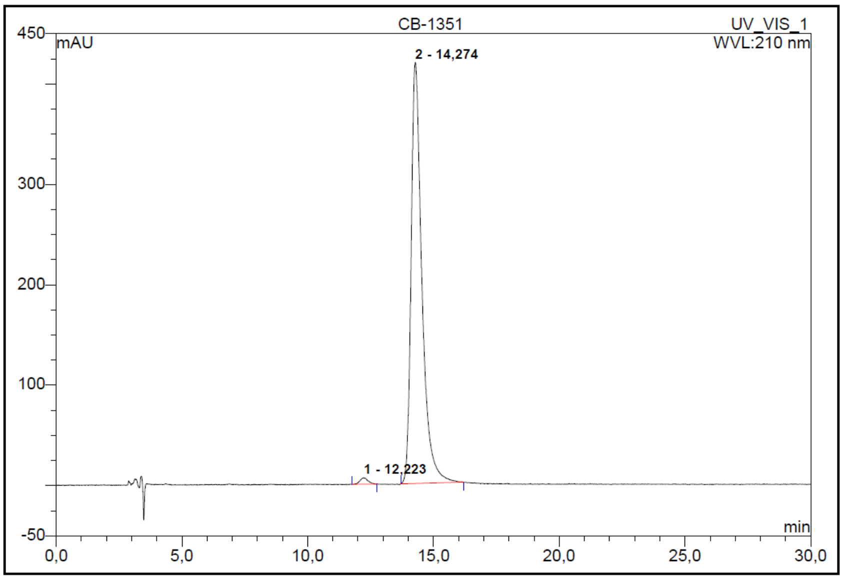

No.	Ret.Time min	Peak Name	Height mAU	Area mAU*min	Rel.Area %	Amount	Type
1	12,22	n.a.	6,518	2,346	1,11	n.a.	BMB*
2	14,27	n.a.	419,410	208,322	98,89	n.a.	BMB*
Total:			425,928	210,667	100,00	0,000	

11.15. 8-(Thiophen-3-yl)-2,3,6,10b-tetrahydropyrrolo[1,2-c]quinazolin-5(1H)-one (5o)

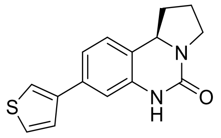

97% *ee*

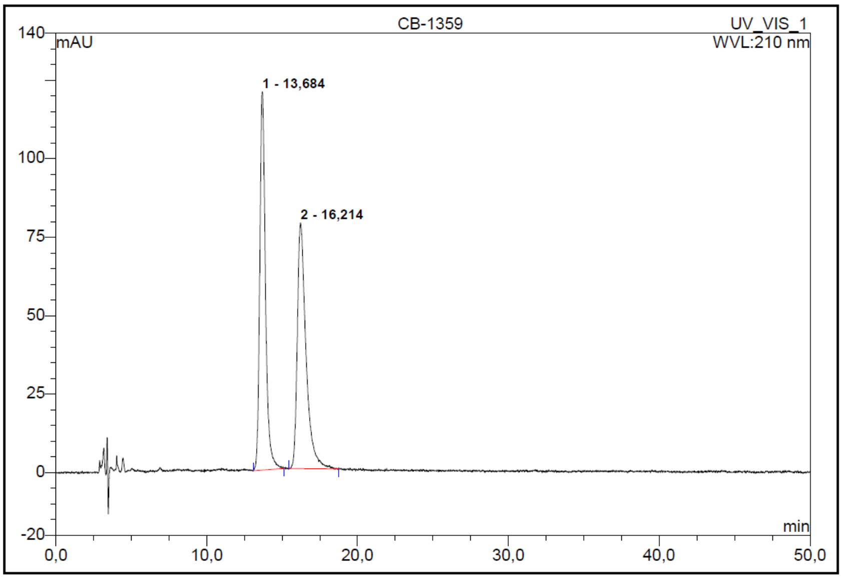

No.	Ret.Time min	Peak Name	Height mAU	Area mAU*min	Rel.Area %	Amount	Type
1	13,68	n.a.	120,557	52,929	50,28	n.a.	BMB*
2	16,21	n.a.	78,426	52,337	49,72	n.a.	BMB*
Total:			198,984	105,266	100,00	0,000	

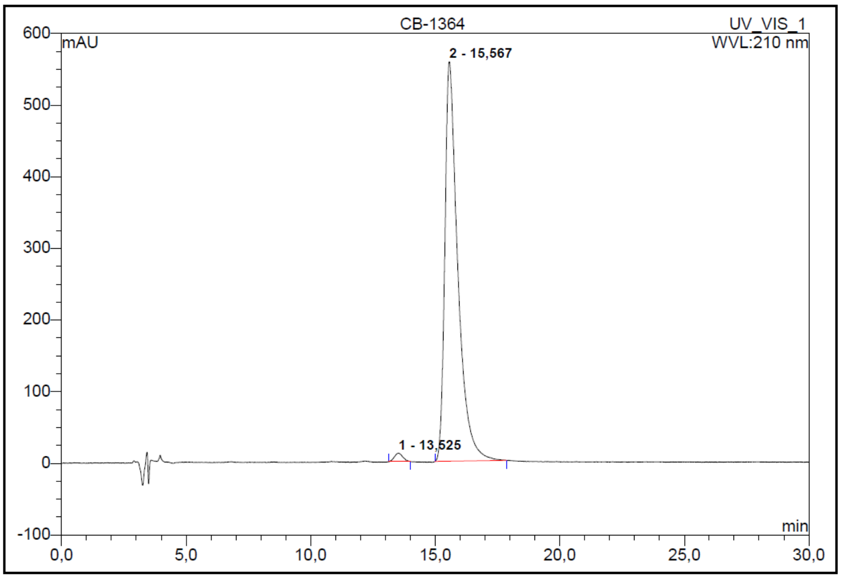

No.	Ret.Time min	Peak Name	Height mAU	Area mAU*min	Rel.Area %	Amount	Type
1	13,52	n.a.	11,688	4,562	1,34	n.a.	BMB*
2	15,57	n.a.	557,684	336,027	98,66	n.a.	BMB*
Total:			569,371	340,589	100,00	0,000	

11.16. tert-Butyl 2-(5-oxo-1,2,3,5,6,10b-hexahydropyrrolo[1,2-c]quinazolin-8-yl)-1H-pyrrole-1-carboxylate (5p)

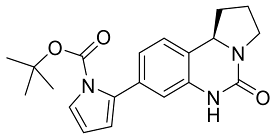

97% *ee*

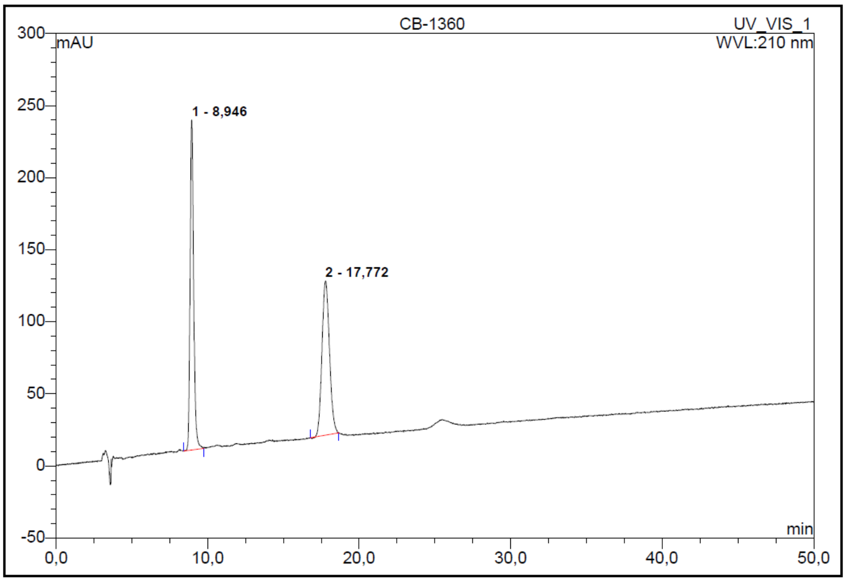

No.	Ret.Time min	Peak Name	Height mAU	Area mAU*min	Rel.Area %	Amount	Type
1	8,95	n.a.	229,045	62,279	50,54	n.a.	BMB*
2	17,77	n.a.	107,100	60,941	49,46	n.a.	BMB*
Total:			336,144	123,219	100,00	0,000	

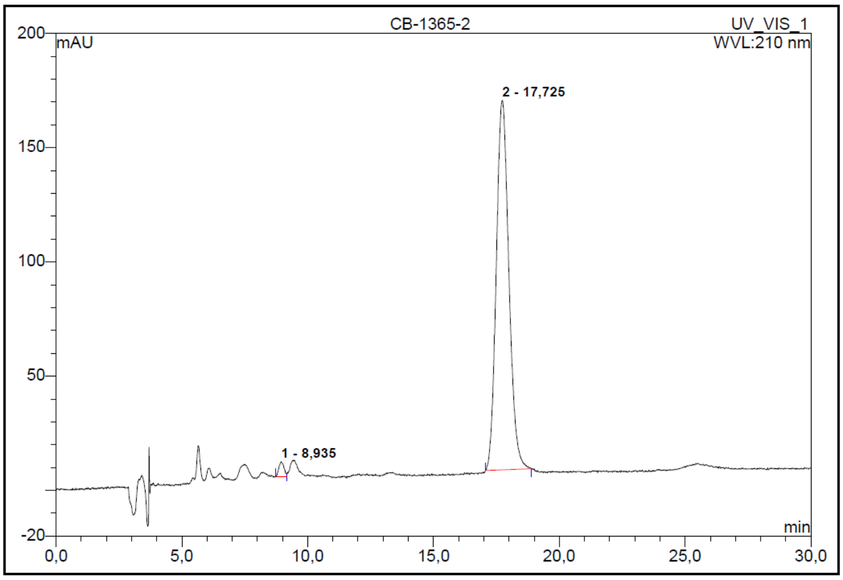

No.	Ret.Time min	Peak Name	Height mAU	Area mAU*min	Rel.Area %	Amount	Type
1	8,94	n.a.	6,570	1,542	1,62	n.a.	BM *
2	17,72	n.a.	161,731	93,752	98,38	n.a.	BMB*
Total:			168,301	95,294	100,00	0,000	

11.17. 8-(Pyridin-3-yl)-2,3,6,10b-tetrahydropyrrolo[1,2-c]quinazolin-5(1H)-one (5q)

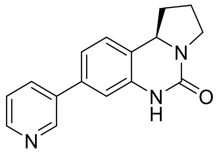

97% *ee*

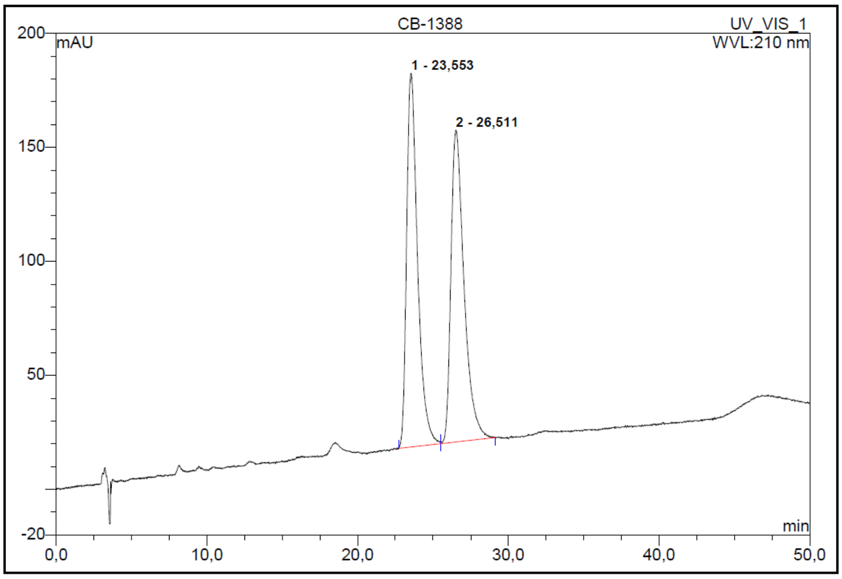

No.	Ret.Time min	Peak Name	Height mAU	Area mAU*min	Rel.Area %	Amount	Type
1	23,55	n.a.	163,864	137,541	50,02	n.a.	BM *
2	26,51	n.a.	136,785	137,424	49,98	n.a.	MB*
Total:			300,649	274,965	100,00	0,000	

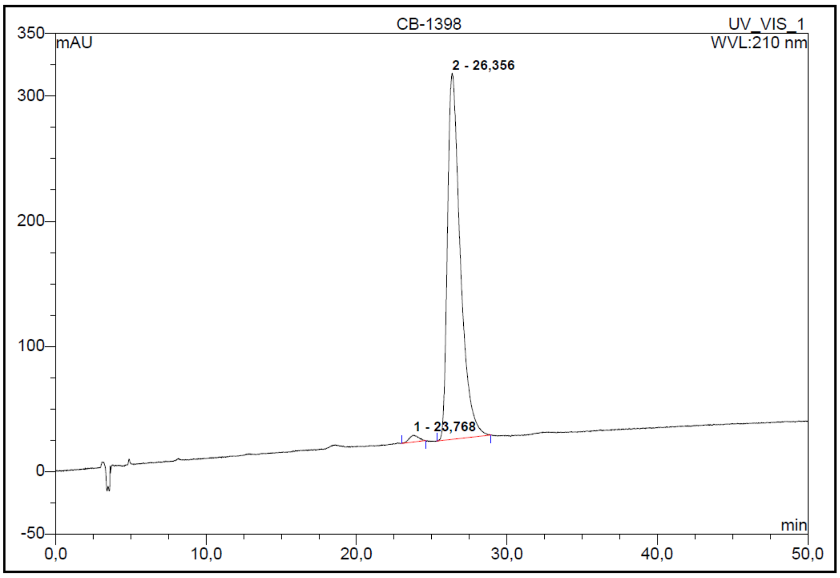

No.	Ret.Time min	Peak Name	Height mAU	Area mAU*min	Rel.Area %	Amount	Type
1	23,77	n.a.	5,438	3,796	1,30	n.a.	BMB*
2	26,36	n.a.	292,683	288,518	98,70	n.a.	BMB*
Total:			298,121	292,314	100,00	0,000	

11.18. Ethyl (E)-3-(5-oxo-1,2,3,5,6,10b-hexahydropyrrolo[1,2-c]quinazolin-8-yl)acrylate (5r)

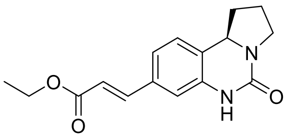

96% *ee*

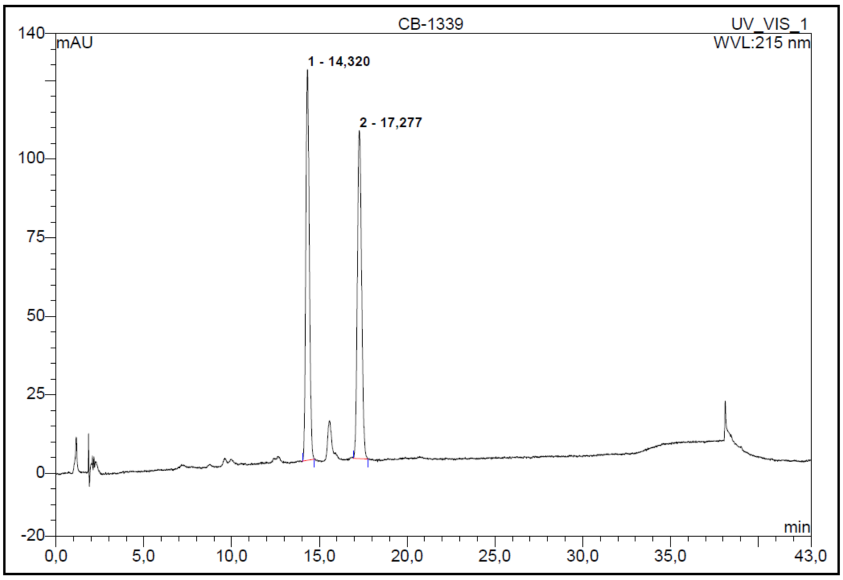

No.	Ret.Time min	Peak Name	Height mAU	Area mAU*min	Rel.Area %	Amount	Type
1	14,32	n.a.	124,417	28,908	49,80	n.a.	BMB
2	17,28	n.a.	104,527	29,134	50,20	n.a.	BMB
Total:			228,944	58,041	100,00	0,000	

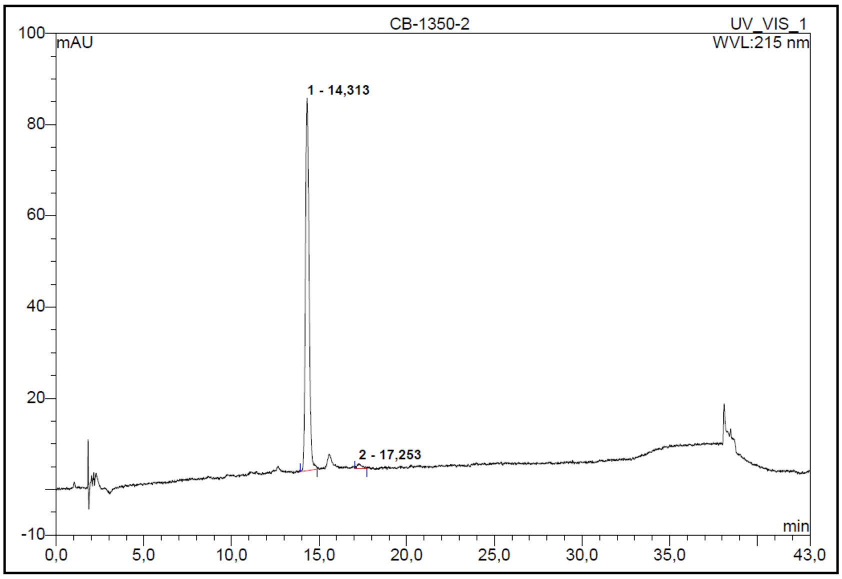

No.	Ret.Time min	Peak Name	Height mAU	Area mAU*min	Rel.Area %	Amount	Type
1	14,31	n.a.	81,690	19,519	97,99	n.a.	BMB*
2	17,25	n.a.	1,162	0,401	2,01	n.a.	BMB*
Total:			82,852	19,920	100,00	0,000	

11.19. 9-(4-Methoxyphenyl)-2,3,6,10b-tetrahydropyrrolo[1,2-c]quinazolin-5(1H)-one (5s)

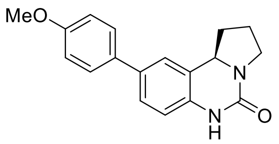

91% *ee*

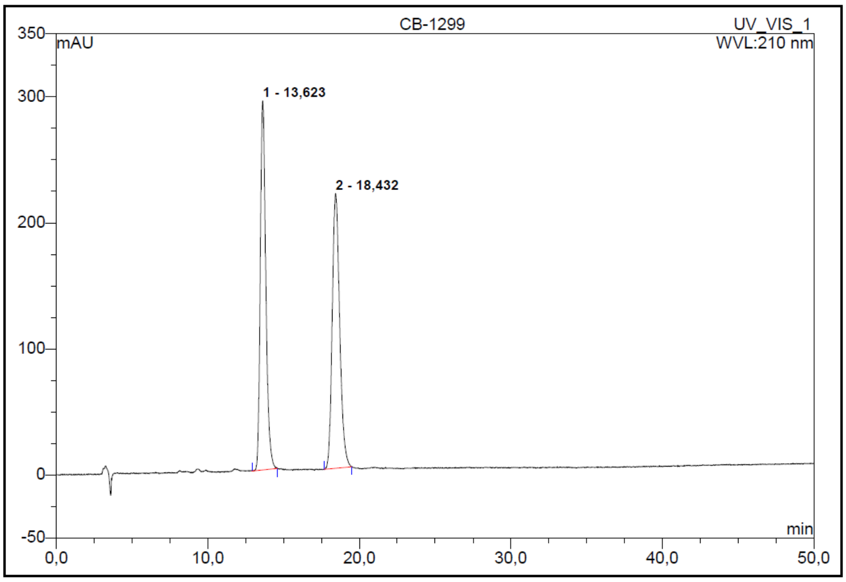

No.	Ret.Time min	Peak Name	Height mAU	Area mAU*min	Rel.Area %	Amount	Type
1	13,62	n.a.	292,837	123,618	49,92	n.a.	BMB*
2	18,43	n.a.	218,049	124,005	50,08	n.a.	BMB*
Total:			510,886	247,624	100,00	0,000	

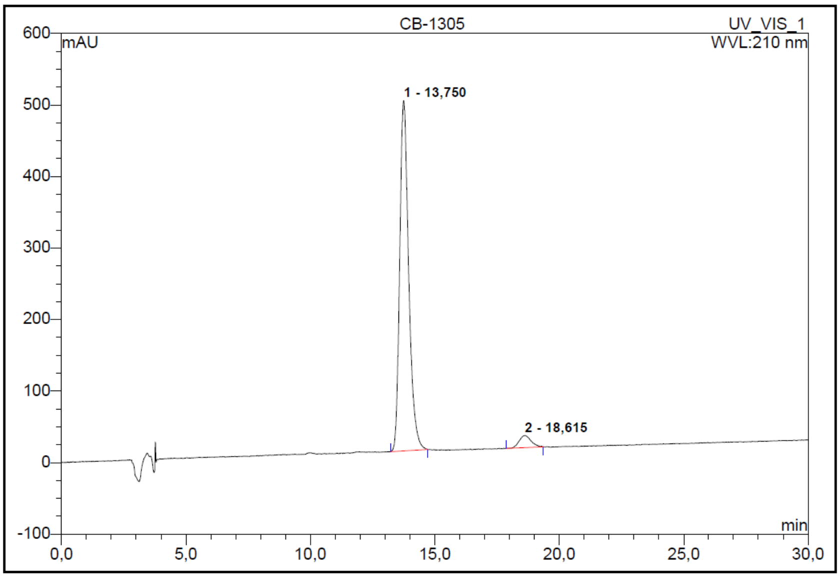

No.	Ret.Time min	Peak Name	Height mAU	Area mAU*min	Rel.Area %	Amount	Type
1	13,75	n.a.	489,607	204,519	95,59	n.a.	BMB
2	18,62	n.a.	17,038	9,426	4,41	n.a.	BMB*
Total:			506,646	213,945	100,00	0,000	

11.20. 8,9,10,10a-Tetrahydropyrido[2,3-e]pyrrolo[1,2-c]pyrimidin-6(5H)-one (5t)

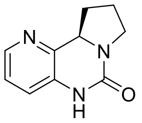

81% *ee*

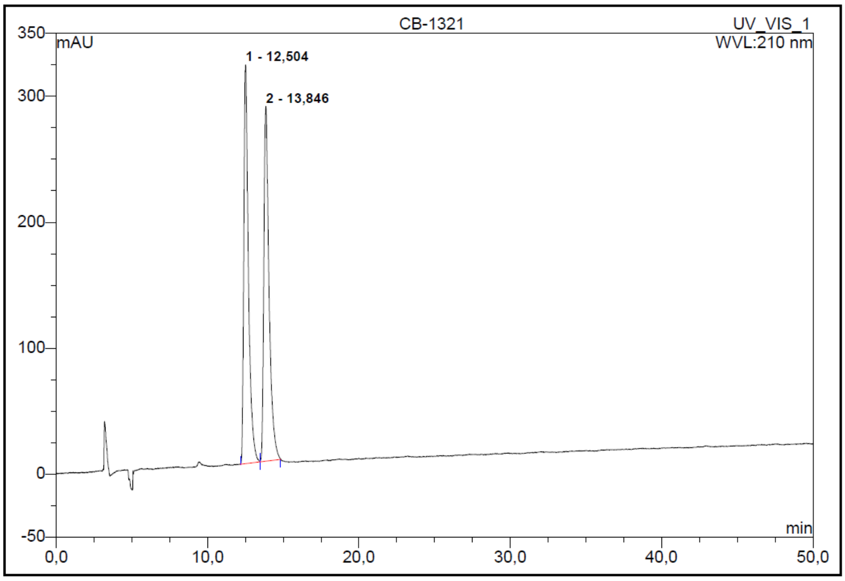

No.	Ret.Time min	Peak Name	Height mAU	Area mAU*min	Rel.Area %	Amount	Type
1	12,50	n.a.	316,598	112,717	49,99	n.a.	BM
2	13,85	n.a.	281,728	112,775	50,01	n.a.	MB
Total:			598,326	225,492	100,00	0,000	

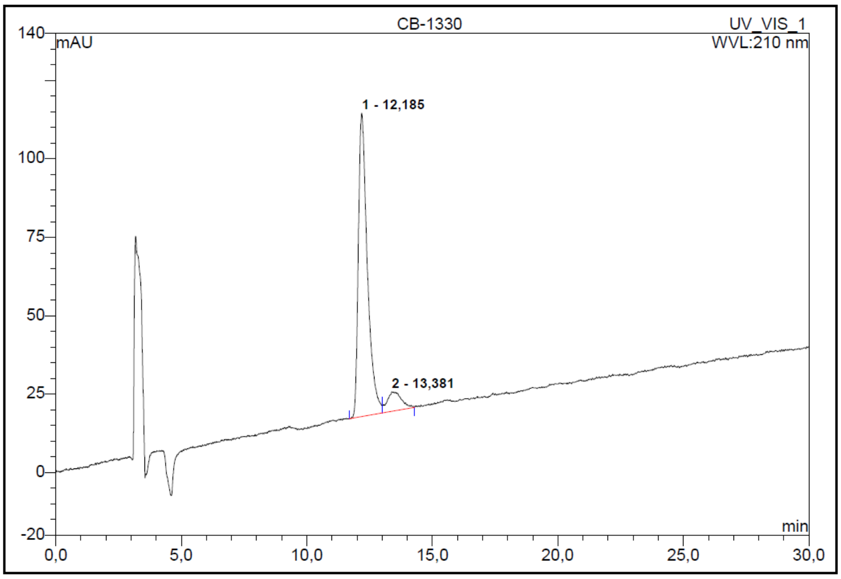

No.	Ret.Time min	Peak Name	Height mAU	Area mAU*min	Rel.Area %	Amount	Type
1	12,18	n.a.	96,780	40,279	90,70	n.a.	BM *
2	13,38	n.a.	6,276	4,131	9,30	n.a.	MB*
Total:			103,056	44,410	100,00	0,000	

11.21. 2,2-Dimethyl-2,3,6,10b-tetrahydropyrrolo[1,2-c]quinazolin-5(1H)-one (5u)

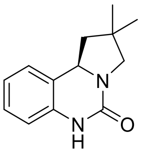

85% *ee*

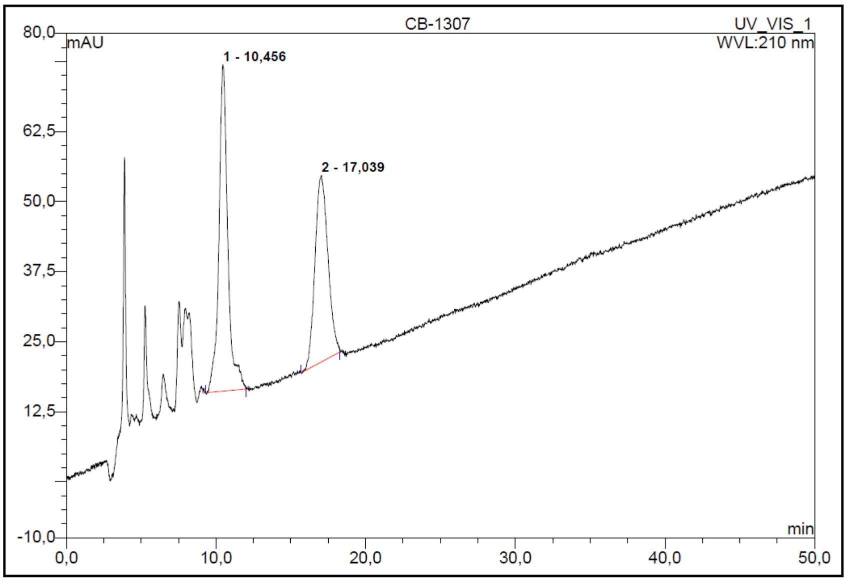

No.	Ret.Time min	Peak Name	Height mAU	Area mAU*min	Rel.Area %	Amount	Type
1	10,46	n.a.	58,306	40,134	54,65	n.a.	BMB*
2	17,04	n.a.	33,329	33,299	45,35	n.a.	BMB*
Total:			91,635	73,433	100,00	0,000	

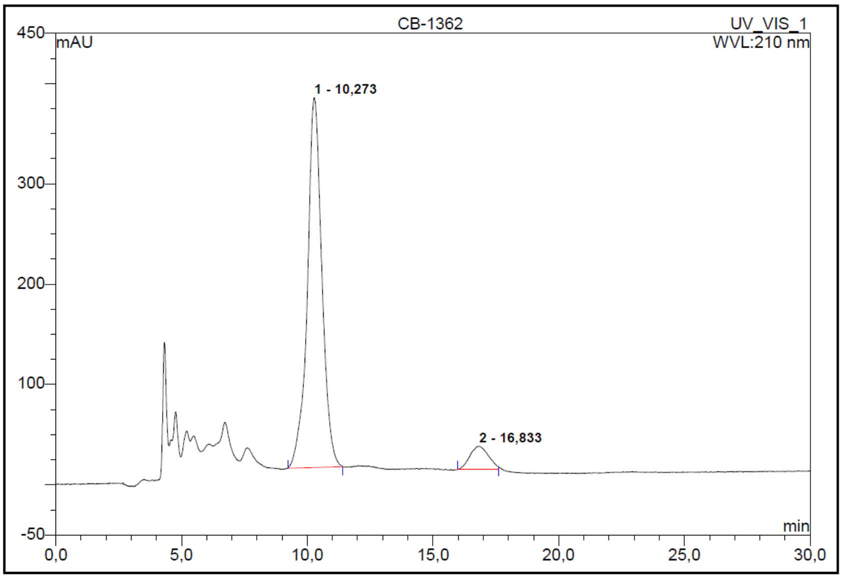

No.	Ret.Time min	Peak Name	Height mAU	Area mAU*min	Rel.Area %	Amount	Type
1	10,27	n.a.	368,750	247,372	92,51	n.a.	BMB
2	16,83	n.a.	23,258	20,014	7,49	n.a.	BM *
Total:			392,007	267,386	100,00	0,000	

11.22. 8,9,10,10a-Tetrahydropyrido[3,2-e]pyrrolo[1,2-c]pyrimidin-6(5H)-one (5v)

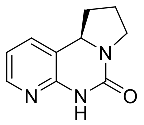

49% *ee*

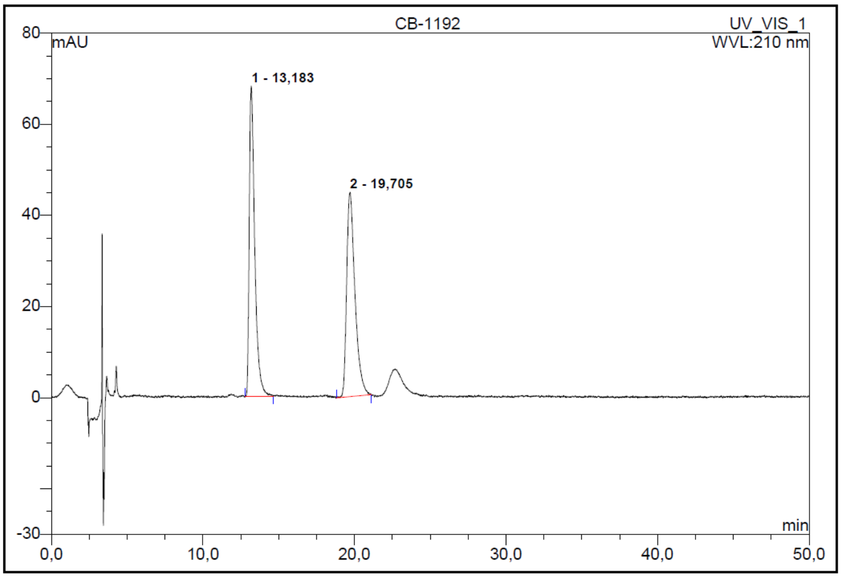

No.	Ret.Time min	Peak Name	Height mAU	Area mAU*min	Rel.Area %	Amount	Type
1	13,18	n.a.	68,040	28,527	50,05	n.a.	BMB*
2	19,70	n.a.	44,870	28,468	49,95	n.a.	BMB*
Total:			112,910	56,995	100,00	0,000	

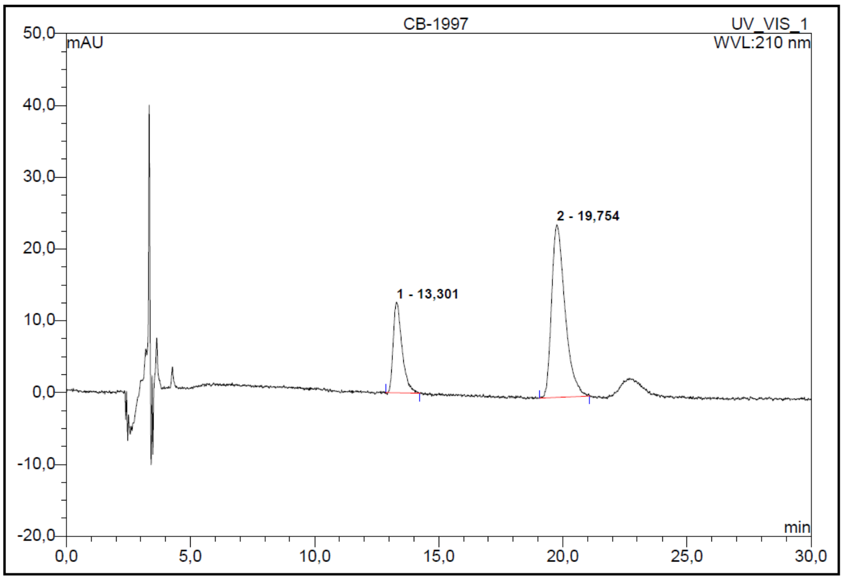

No.	Ret.Time min	Peak Name	Height mAU	Area mAU*min	Rel.Area %	Amount	Type
1	13,30	n.a.	12,610	5,193	25,60	n.a.	BMB*
2	19,75	n.a.	24,069	15,090	74,40	n.a.	BMB*
Total:			36,680	20,283	100,00	0,000	

11.23. 5,8,9,10,11,11a-Hexahydro-6H-pyrido[1,2-c]quinazolin-6-one (5aa)

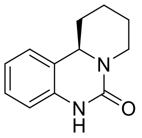

98% *ee*

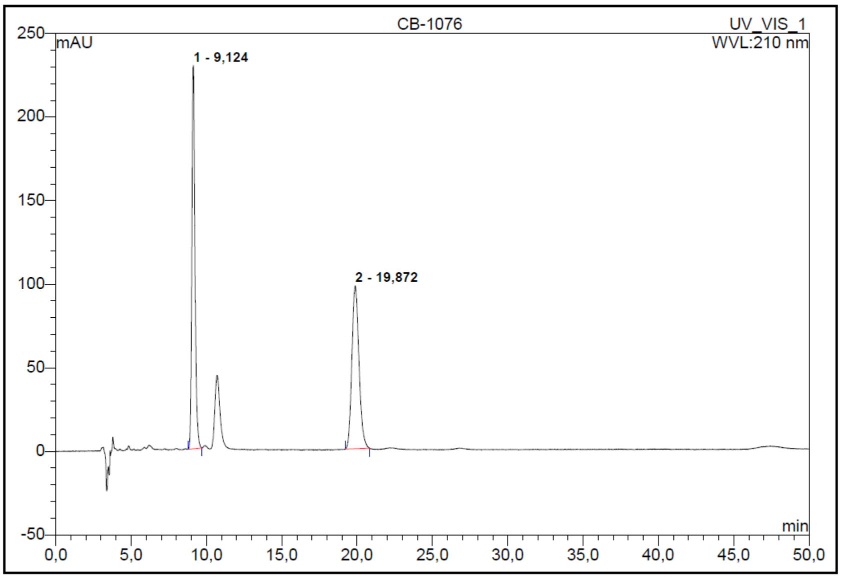

No.	Ret.Time min	Peak Name	Height mAU	Area mAU*min	Rel.Area %	Amount	Type
1	9,12	n.a.	229,441	54,685	50,75	n.a.	BMB
2	19,87	n.a.	97,519	53,070	49,25	n.a.	BMB
Total:			326,959	107,755	100,00	0,000	

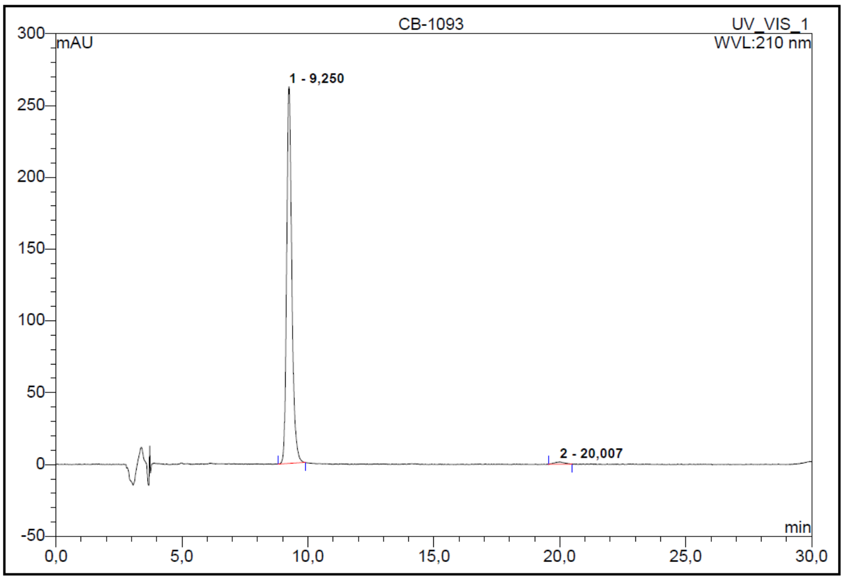

No.	Ret.Time min	Peak Name	Height mAU	Area mAU*min	Rel.Area %	Amount	Type
1	9,25	n.a.	262,336	62,623	98,86	n.a.	BMB*
2	20,01	n.a.	1,625	0,721	1,14	n.a.	BMB*
Total:			263,961	63,344	100,00	0,000	

11.24. 5,8,13,13a-Tetrahydro-6H-isoquinolino[2,3-c]quinazolin-6-one (5ab)

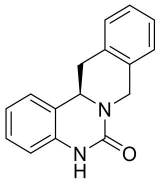

96% *ee*

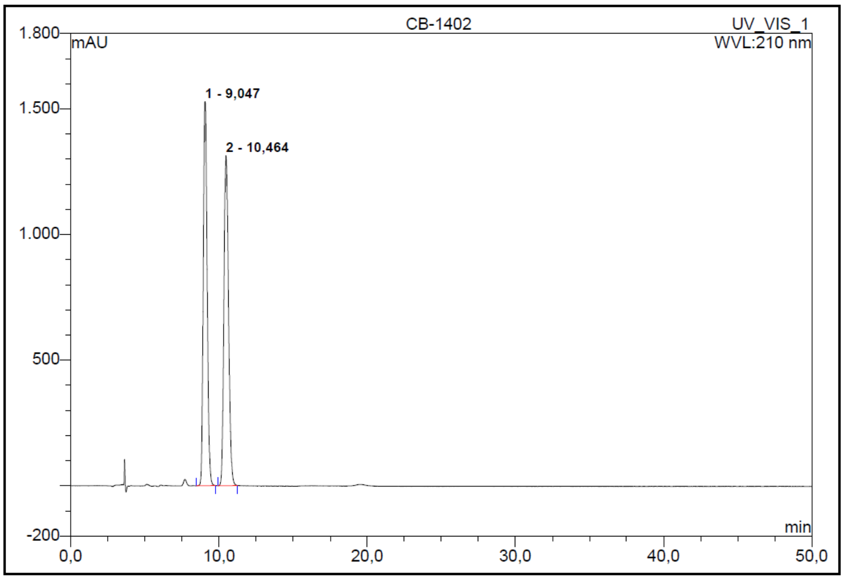

No.	Ret.Time min	Peak Name	Height mAU	Area mAU*min	Rel.Area %	Amount	Type
1	9,05	n.a.	1529,062	465,427	49,60	n.a.	BMB*
2	10,46	n.a.	1313,326	472,890	50,40	n.a.	BMB
Total:			2842,388	938,317	100,00	0,000	

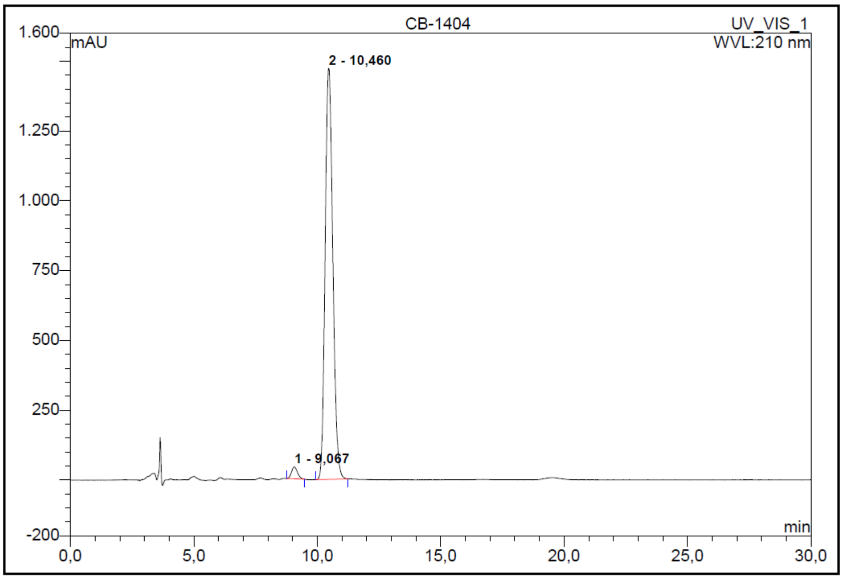

No.	Ret.Time min	Peak Name	Height mAU	Area mAU*min	Rel.Area %	Amount	Type
1	9,07	n.a.	42,014	11,759	2,16	n.a.	BMB
2	10,46	n.a.	1472,617	531,580	97,84	n.a.	BMB
Total:			1514,630	543,339	100,00	0,000	

11.25. 9,9-Dimethyl-5,8,9,10,11,11a-hexahydro-6H-pyrido[1,2-c]quinazolin-6-one (5ac)

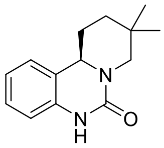

94% *ee*

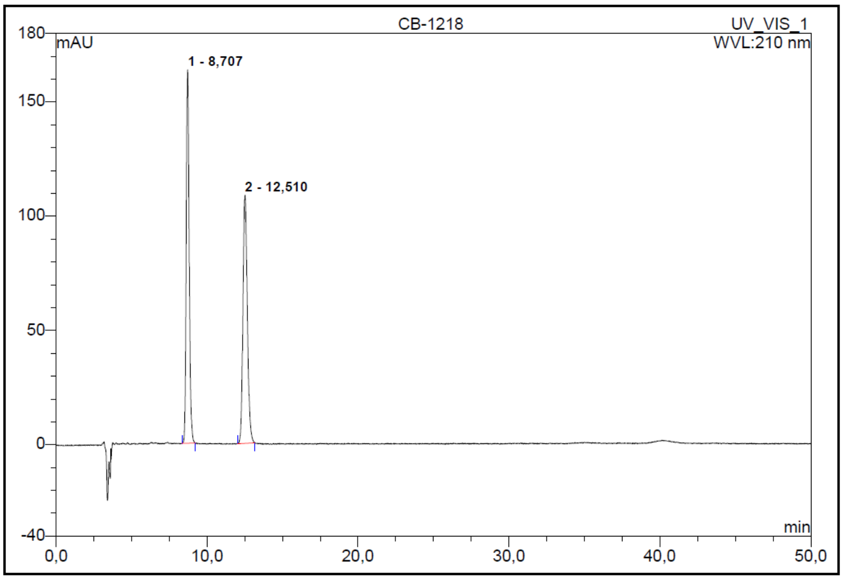

No.	Ret.Time min	Peak Name	Height mAU	Area mAU*min	Rel.Area %	Amount	Type
1	8,71	n.a.	163,516	37,010	50,12	n.a.	BMB
2	12,51	n.a.	108,655	36,828	49,88	n.a.	BMB
Total:			272,171	73,838	100,00	0,000	

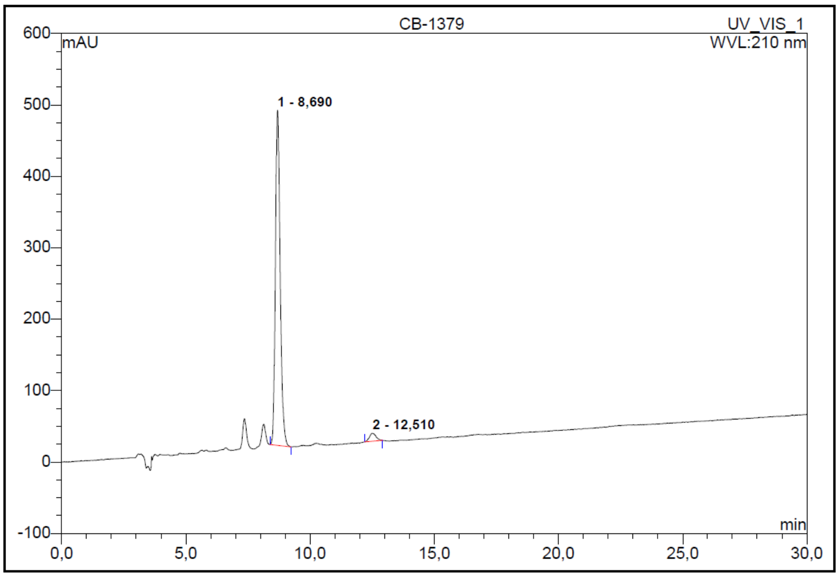

No.	Ret.Time min	Peak Name	Height mAU	Area mAU*min	Rel.Area %	Amount	Type
1	8,69	n.a.	469,120	105,035	96,79	n.a.	BMB
2	12,51	n.a.	11,223	3,485	3,21	n.a.	BMB*
Total:			480,343	108,519	100,00	0,000	

11.26. 8,9,14,14a-Tetrahydrobenzo[4,5]azepino[1,2-c]quinazolin-6(5H)-one (5ba)

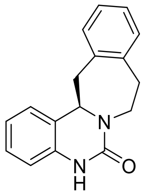

75% *ee*

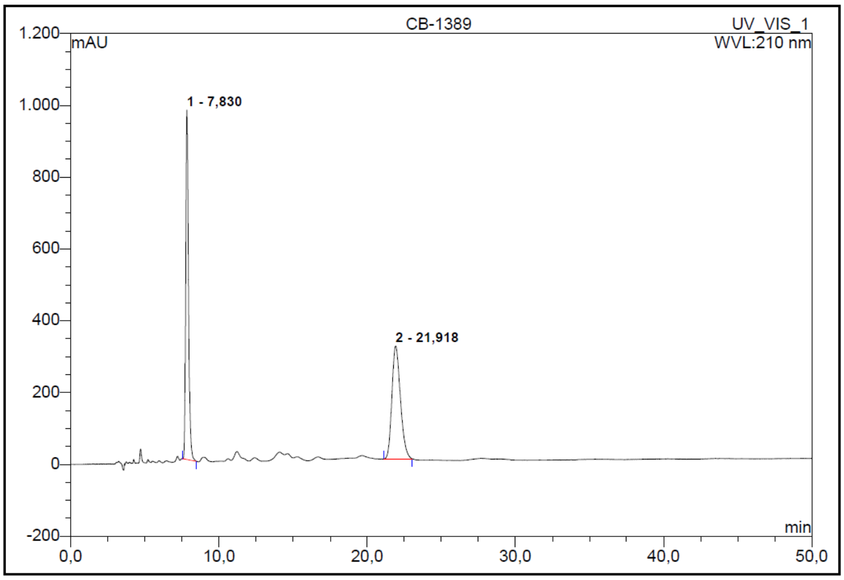

No.	Ret.Time min	Peak Name	Height mAU	Area mAU*min	Rel.Area %	Amount	Type
1	7,83	n.a.	973,300	209,988	49,98	n.a.	BMB
2	21,92	n.a.	314,392	210,181	50,02	n.a.	BMB
Total:			1287,692	420,169	100,00	0,000	

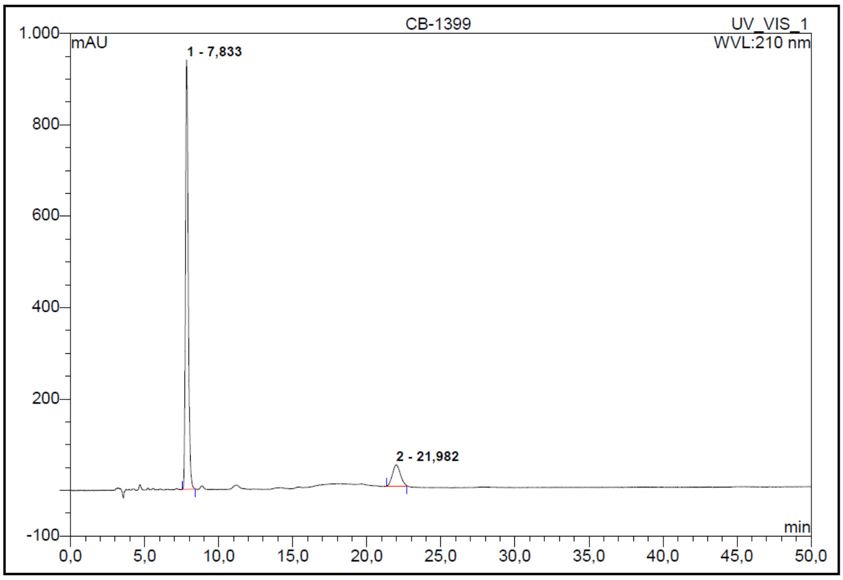

No.	Ret.Time min	Peak Name	Height mAU	Area mAU*min	Rel.Area %	Amount	Type
1	7,83	n.a.	939,245	202,103	87,57	n.a.	BMB
2	21,98	n.a.	46,456	28,687	12,43	n.a.	BMB
Total:			985,700	230,790	100,00	0,000	

11.27. 8,9,15,15a-Tetrahydro-[1,3]dioxolo[4,5:4,5]benzo[1,2:4,5]azepino[1,2-c]quinazolin-6(5H)-one (5bb)

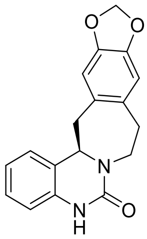

78% *ee*

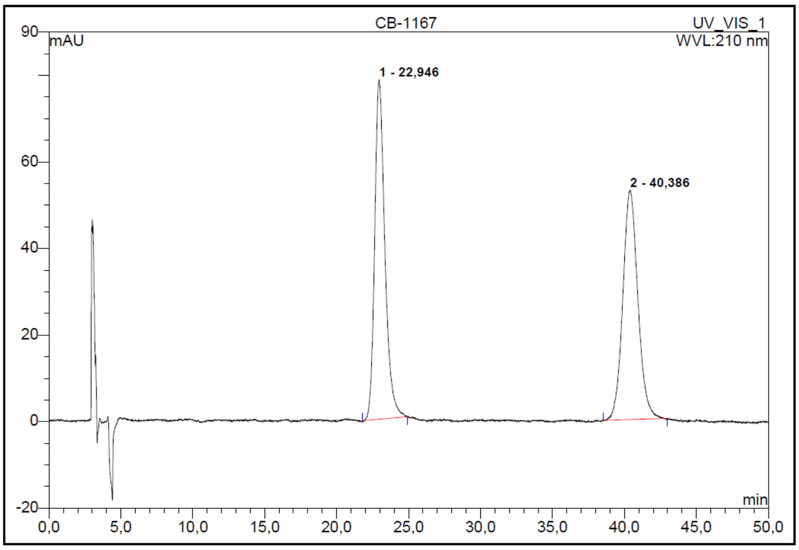

No.	Ret.Time min	Peak Name	Height mAU	Area mAU*min	Rel.Area %	Amount	Type
1	22,95	n.a.	78,469	64,254	50,03	n.a.	BMB*
2	40,39	n.a.	52,971	64,177	49,97	n.a.	BMB*
Total:			131,440	128,430	100,00	0,000	

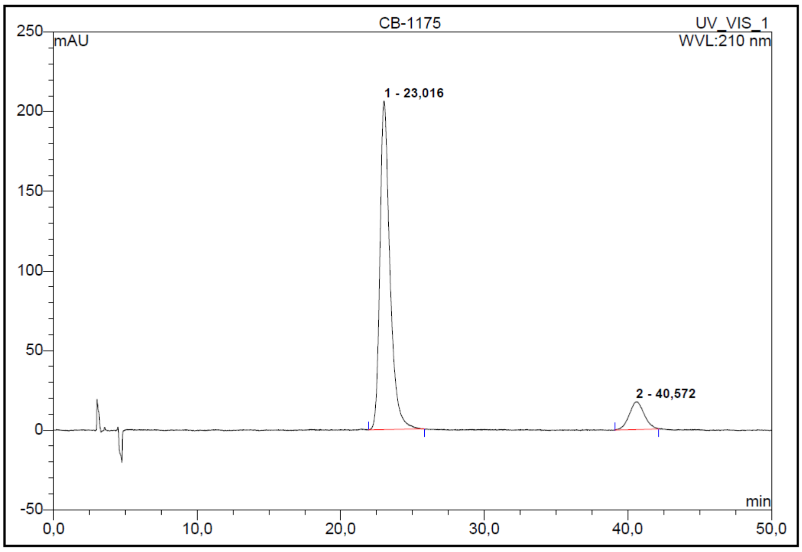

No.	Ret.Time min	Peak Name	Height mAU	Area mAU*min	Rel.Area %	Amount	Type
1	23,02	n.a.	206,377	166,621	88,77	n.a.	BMB*
2	40,57	n.a.	17,560	21,084	11,23	n.a.	BMB*
Total:			223,937	187,706	100,00	0,000	

11.28. 11,12-Dimethoxy-8,9,14,14a-tetrahydrobenzo[4,5]azepino[1,2-c]quinazolin-6(5H)-one (5bc)

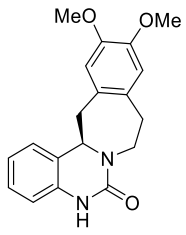

74% *ee*

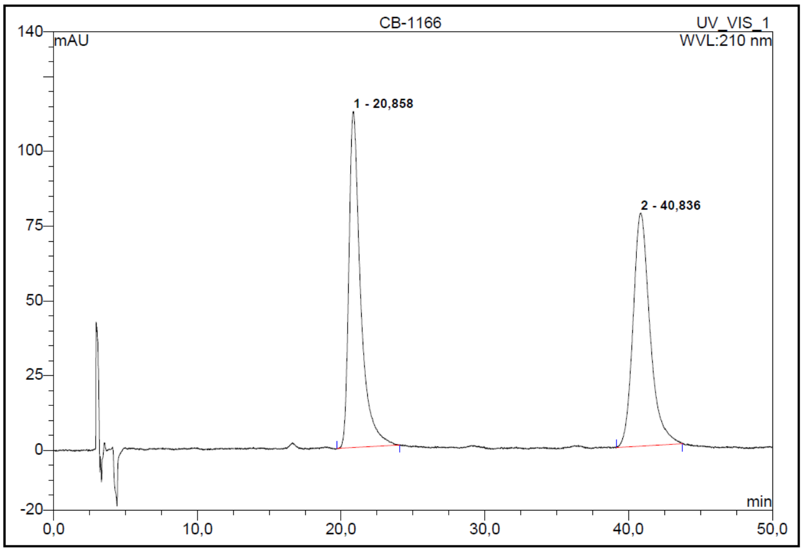

No.	Ret.Time min	Peak Name	Height mAU	Area mAU*min	Rel.Area %	Amount	Type
1	20,86	n.a.	112,546	106,197	49,87	n.a.	BMB*
2	40,84	n.a.	78,049	106,759	50,13	n.a.	BMB*
Total:			190,595	212,956	100,00	0,000	

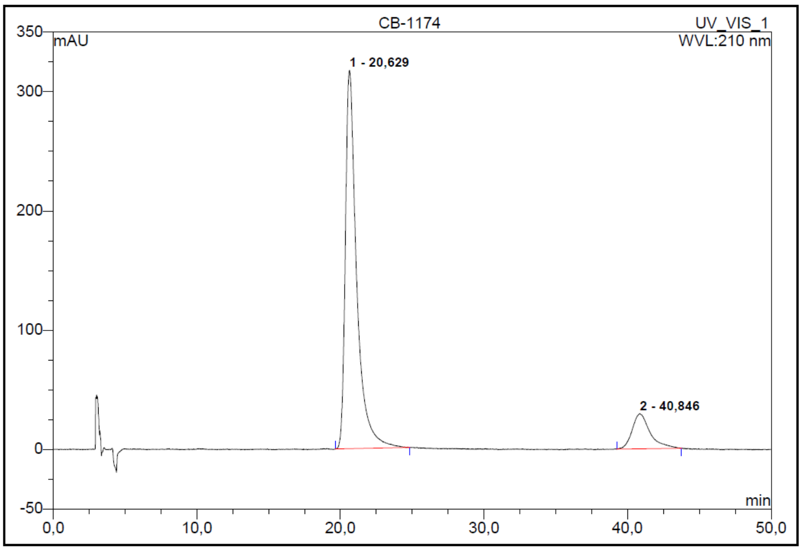

No.	Ret.Time min	Peak Name	Height mAU	Area mAU*min	Rel.Area %	Amount	Type
1	20,63	n.a.	317,369	289,109	87,20	n.a.	BMB*
2	40,85	n.a.	29,576	42,453	12,80	n.a.	BMB*
Total:			346,945	331,562	100,00	0,000	

12. References

[1] Buchelt, C.; Zuber, J.; Bach, T., Intramolecular Cobalt Porphyrin-Catalyzed Alkylation of 1-Isoindolinones by Site-Selective Insertion into a C(sp(3))-H Bond. *Org. Lett.* **2024,** *26*, 7302-7306.

[2] Frost, J. R.; Huber, S. M.; Breitenlechner, S.; Bannwarth, C.; Bach, T., Enantiotopos-selective C-H oxygenation catalyzed by a supramolecular ruthenium complex. *Angew. Chem. Int. Ed.* **2015,** *54*, 691-695.

[3] Schissler, C.; Schneider, E. K.; Felker, B.; Weis, P.; Nieger, M.; Kappes, M. M.; Brase, S., A Synthetic Strategy for Cofacial Porphyrin-Based Homo- and Heterobimetallic Complexes. *Chemistry* **2021,** *27*, 3047-3054.

[4] Banfi, S.; Caruso, E.; Buccafurni, L.; Murano, R.; Monti, E.; Gariboldi, M.; Papa, E.; Gramatica, P., Comparison between 5,10,15,20-tetraaryl- and 5,15-diarylporphyrins as photosensitizers: synthesis, photodynamic activity, and quantitative structure-activity relationship modeling. *J. Med. Chem.* **2006,** *49*, 3293-3304.

[5] Plunkett, S.; Dahms, K.; Senge, M. O., Synthesis and Reactivity of Allenylporphyrins. *Eur. J. Org. Chem.* **2013,** *2013*, 1566-1579.

[6] Fackler, P.; Huber, S. M.; Bach, T., Enantio- and regioselective epoxidation of olefinic double bonds in quinolones, pyridones, and amides catalyzed by a ruthenium porphyrin catalyst with a hydrogen bonding site. *J. Am. Chem. Soc.* **2012,** *134*, 12869-12878.

[7] Burg, F.; Gicquel, M.; Breitenlechner, S.; Pothig, A.; Bach, T., Site- and Enantioselective C-H Oxygenation Catalyzed by a Chiral Manganese Porphyrin Complex with a Remote Binding Site. *Angew. Chem. Int. Ed.* **2018,** *57*, 2953-2957.

[8] Fackler, P.; Berthold, C.; Voss, F.; Bach, T., Hydrogen-bond-mediated enantio- and regioselectivity in a Ru-catalyzed epoxidation reaction. *J. Am. Chem. Soc.* **2010,** *132*, 15911-15913.

[9] Wang, Y.; Wen, X.; Cui, X.; Zhang, X. P., Enantioselective Radical Cyclization for Construction of 5-Membered Ring Structures by Metalloradical C-H Alkylation. *J. Am. Chem. Soc.* **2018,** *140*, 4792-4796.

[10] Pechulis, A. D.; Beck, J. P.; Curry, M. A.; Wolf, M. A.; Harms, A. E.; Xi, N.; Opalka, C.; Sweet, M. P.; Yang, Z.; Vellekoop, A. S.; Klos, A. M.; Crocker, P. J.; Hassler, C.; Laws, M.; Kitchen, D. B.; Smith, M. A.; Olson, R. E.; Liu, S.; Molino, B. F., 4-Phenyl tetrahydroisoquinolines as dual norepinephrine and dopamine reuptake inhibitors. *Bioorg. Med. Chem. Lett.* **2012,** *22*, 7219-7222.

[11] Thanigaimalai, P.; Lee, K. C.; Bang, S. C.; Lee, J. H.; Yun, C. Y.; Roh, E.; Hwang, B. Y.; Kim, Y.; Jung, S. H., Evaluation of 3,4-dihydroquinazoline-2(1H)-thiones as inhibitors of alpha-MSH-induced melanin production in melanoma B16 cells. *Bioorg. Med. Chem.* **2010,** *18*, 1555-1562.

[12] Ryabukhin, S. V.; Volochnyuk, D. M.; Iermolenko, I. A.; Kolosov, O. S.; Ostapchuk, E. N.; Lega, D. A.; Derkach, N. O.; Levchenko, K. V.; Makhankova, V. G.; Rozhenko, A. B., Practical Multigram Approach to Conformationally Constrained α-Proline-Based Building Blocks with γ-Spiro Conjunction. *Synthesis* **2024,** *57*, 664-674.

[13] Cortez, A.; Li, Y.; Miller, A. T.; Zhang, X.; Yue, K.; Maginnis, J.; Hampton, J.; Hall de, S.; Shapiro, M.; Nayak, B.; D'Oro, U.; Li, C.; Skibinski, D.; Mbow, M. L.; Singh, M.; O'Hagan, D. T.; Cooke, M. P.; Valiante, N. M.; Wu, T. Y., Incorporation of Phosphonate into Benzonaphthyridine Toll-like Receptor 7 Agonists for Adsorption to Aluminum Hydroxide. *J. Med. Chem.* **2016,** *59*, 5868-5878.

[14] Omura, K.; Swern, D., Oxidation of alcohols by “activated” dimethyl sulfoxide. a preparative, steric and mechanistic study. *Tetrahedron* **1978,** *34*, 1651-1660.

[15] Lu, Y.; Lin, H.; Xu, Y.; Shen, Z.; Guo, Y.; Jin, Y.; Shi, Q.; Chen, H.; Zhuang, Y.; Huang, W.; Che, J.; Dai, H.; Dong, X., Discovery of orally bioavailable phenyltetrazolium derivatives for the acute treatment and the secondary prevention of ischemic stroke. *Eur. J. Med. Chem.* **2024,** *275*, 116542.

[16] Nichols, D.; Cueva, J., A Novel and Efficient Synthesis of Dihydrexidine. *Synthesis* **2009,** *2009*, 715-720.

[17] Gruber, N.; Diaz, J. E.; Orelli, L. R., Synthesis of dihydroquinazolines from 2-aminobenzylamine: N (3) -aryl derivatives with electron-withdrawing groups. *Beilstein J. Org. Chem.* **2018,** *14*, 2510-2519.

[18] Zhang, C.; De, C. K.; Mal, R.; Seidel, D., Alpha-amination of nitrogen heterocycles: ring-fused aminals. *J. Am. Chem. Soc.* **2008,** *130*, 416-417.

[19] Kiyokawa, K.; Kawanaka, K.; Minakata, S., Amino-lambda(3) -iodane-Enabled Electrophilic Amination of Arylboronic Acid Derivatives. *Angew. Chem. Int. Ed.* **2024,** *63*, e202319048.

[20] Frisch, M. J.; Trucks, G. W.; Schlegel, H. B.; Scuseria, G. E.; Robb, M. A.; Cheeseman, J. R.; Scalmani, G.; Barone, V.; Petersson, G. A.; Nakatsuji, H.; Li, X.; Caricato, M.; Marenich, A. V.; Bloino, J.; Janesko, B. G.; Gomperts, R.; Mennucci, B.; Hratchian, H. P.; Ortiz, J. V.; Izmaylov, A. F.; Sonnenberg, J. L.; Williams; Ding, F.; Lipparini, F.; Egidi, F.; Goings, J.; Peng, B.; Petrone, A.; Henderson, T.; Ranasinghe, D.; Zakrzewski, V. G.; Gao, J.; Rega, N.; Zheng, G.; Liang, W.; Hada, M.; Ehara, M.; Toyota, K.; Fukuda, R.; Hasegawa, J.; Ishida, M.; Nakajima, T.; Honda, Y.; Kitao, O.; Nakai, H.; Vreven, T.; Throssell, K.; Montgomery Jr., J. A.; Peralta, J. E.; Ogliaro, F.; Bearpark, M. J.; Heyd, J. J.; Brothers, E. N.; Kudin, K. N.; Staroverov, V. N.; Keith, T. A.; Kobayashi, R.; Normand, J.; Raghavachari, K.; Rendell, A. P.; Burant, J. C.; Iyengar, S. S.; Tomasi, J.; Cossi, M.; Millam, J. M.; Klene, M.; Adamo, C.; Cammi, R.; Ochterski, J. W.; Martin, R. L.; Morokuma, K.; Farkas, O.; Foresman, J. B.; Fox, D. J. *Gaussian 16 Rev. C.01*, Wallingford, CT, 2016.

[21] Zhao, Y.; Truhlar, D. G., A new local density functional for main-group thermochemistry, transition metal bonding, thermochemical kinetics, and noncovalent interactions. *J. Chem. Phys.* **2006,** *125*, 194101.

[22] Grimme, S.; Antony, J.; Ehrlich, S.; Krieg, H., A consistent and accurate ab initio parametrization of density functional dispersion correction (DFT-D) for the 94 elements H-Pu. *J. Chem. Phys.* **2010,** *132*, 154104.

[23] Rappoport, D.; Furche, F., Property-optimized gaussian basis sets for molecular response calculations. *J. Chem. Phys.* **2010,** *133*, 134105.

[24] Weigend, F.; Ahlrichs, R., Balanced basis sets of split valence, triple zeta valence and quadruple zeta valence quality for H to Rn: Design and assessment of accuracy. *Phys. Chem. Chem. Phys.* **2005,** *7*, 3297-3305.

[25] Grimme, S., Supramolecular binding thermodynamics by dispersion-corrected density functional theory. *Chemistry* **2012,** *18*, 9955-9964.

[26] Paton, R., Kinisot. py, version 2.0. 2. Zenodo: 2023.

[27] Rzepa, H. S., KINISOT. A basic program to calculate kinetic isotope effects using normal coordinate analysis of transition state and reactants. Zenodo: 2015.

[28] Bakowski, A.; Dressel, M.; Bauer, A.; Bach, T., Enantioselective radical cyclisation reactions of 4-substituted quinolones mediated by a chiral template. *Org. Biomol. Chem.* **2011,** *9*, 3516-3529.

[29] Bach, T.; Bergmann, H.; Grosch, B.; Harms, K., Highly enantioselective intra- and intermolecular [2 + 2] photocycloaddition reactions of 2-quinolones mediated by a chiral lactam host: host-guest interactions, product configuration, and the origin of the stereoselectivity in solution. *J. Am. Chem. Soc.* **2002,** *124*, 7982-7990.

[30] Plaza, M.; Grosskopf, J.; Breitenlechner, S.; Bannwarth, C.; Bach, T., Photochemical Deracemization of Primary Allene Amides by Triplet Energy Transfer: A Combined Synthetic and Theoretical Study. *J. Am. Chem. Soc.* **2021,** *143*, 11209-11217.

[31] Kratz, T.; Steinbach, P.; Breitenlechner, S.; Storch, G.; Bannwarth, C.; Bach, T., Photochemical deracemization of chiral alkenes via triplet energy transfer. *J. Am. Chem. Soc.* **2022,** *144*, 10133-10138.

[32] Kutta, R. J.; Großkopf, J.; van Staalduinen, N.; Seitz, A.; Pracht, P.; Breitenlechner, S.; Bannwarth, C.; Nuernberger, P.; Bach, T., Multifaceted view on the mechanism of a photochemical deracemization reaction. *J. Am. Chem. Soc.* **2023,** *145*, 2354-2363.

[33] Pflaum, N.; Pauls, M.; Kumar, A.; Kutta, R. J.; Nuernberger, P.; Hauer, J. r.; Bannwarth, C.; Bach, T., Oxetane Cleavage Pathways in the Excited State: Photochemical Kinetic Resolution as an Approach to Enantiopure Oxetanes. *J. Am. Chem. Soc.* **2025,** *147*, 13893-13904.

[34] Thordarson, P., Determining association constants from titration experiments in supramolecular chemistry. *Chem. Soc. Rev.* **2011,** *40*, 1305-1323.

[35] Alper, J. S.; Gelb, R. I., Standard errors and confidence intervals in nonlinear regression: comparison of Monte Carlo and parametric statistics. *J. Phys. Chem.* **2002,** *94*, 4747-4751.
